# Experimental data from gas burner fires in residential structure with HVAC system

**DOI:** 10.1016/j.dib.2022.108825

**Published:** 2022-12-14

**Authors:** Craig Weinschenk, Shruti Ghanekar, Keith Stakes, Adam Quiat, Richard M. Kessler, Tonghun Lee

**Affiliations:** a UL Fire Safety Research Institute, Columbia, MD, USA; b University of Illinois Urbana-Champaign, Champaign, IL, USA; c Rhino Fire Protection Engineering, Reston, VA, USA

**Keywords:** Fire model CFD, Validation gas burner, Residential structures ventilation, HVAC

## Abstract

The fire modeling community currently lacks full-scale experi- mental data from fires in residential-style structures with heat- ing, ventilation, and air conditioning (HVAC) systems. Further, there is an absence of data quantifying the generation of H_2_O due to combustion and the subsequent transport of those gases with a structure. Propane gas burner fire experiments were con- ducted in a purpose-built two-story structure instrumented to measure temperature, pressure, velocity, and gas concentrations. Experiments were conducted to assess heat and gas species trans- fer due to HVAC operating status (off vs. on), fire location and heat release rate, and bedroom door position (open vs. closed).

**Specifications Table**

**Table:** 

Subject	Engineering
Specific subject area	Experimental Thermal and Fluid Sciences and Fire Safety Engineering
Type of data	Tabular
How data were acquired	Type K Thermocouples Bi-directional Probes Setra Model 264 Capacitive Differential Pressure Sensors Siemens ULTRAMAT 23 non-dispersive infrared (NDIR) gas analyzer Siemens OxyMat 6 paramagnetic oxygen sensor Infrared tunable diode laser absorption spectroscopy (IR-TDLAS) sensor
Data format	Raw and Analyzed
Description of data collection	The gas burner was supplied with a flow rate of propane gas to produce heat release rates that ranged from 110 kW to 360 kW. Thermocouple arrays with sensors arranged from the ceiling to the floor were used to measure temperatures. Arrays of bi-directional probes were used to measure gas flow velocities. Arrays of differential pressure sensors were used to measure static pressure relative to atmospheric pressure external to the structure. Gas concentrations (O_2_, CO_2_, and CO) were measured in four locations and H_2_O concen- trations were measured in three locations. Experiments varied fire location, interior and exterior ventilation, and operating status of HVAC system.
Data source location	Experiments were conducted at the Delaware County Emergency Services Training Cen- ter, Sharon Hill, PA United States Latitude and longitude for collected data: 39.891135, -75.26595
Data accessibility	The data is archived in a github repository at: https://github.com/ulfsri/fsri-hvac-gasburner-2019
Related research article	S. Ghanekar, C. Weinschenk, G. P. Horn, K. Stakes, R. M. Kesler, T. Lee, Effects of HVAC on combustion-gas transport in residential structures, Fire Safety Journal. 128 (2022) 103534. https://doi.org/10.1016/j.firesaf.2022.103534. A. Quiat. Analysis of Propane Gas Burner Experiments and FDS Simulations in a Two-Story Residential Structure with HVAC, [Master's Thesis] College Park: University of Maryland; 2020. https://drum.lib.umd.edu/handle/1903/26719.

## Value of the Data

• These data provide a complete picture of fluid flow and gas-phase heat transfer in residential- style structures given a well-defined fire source and a residential heating, ventilation, and air conditioning (HVAC) system.
• These data may benefit fire model developers and practitioners, fire protection engineers, investigators, and firefighters.
• These data may be used to validate fire models and improve understanding of flow paths generated due to changing ventilation conditions and HVAC system operating status in residential structures.

## 1 Objective

Heat and combustion-gas exposures are two considerable hazards associated with fires in res- idential structures for potentially trapped occupants and responding firefighters. The measure- ment data generated from this series of experiments quantify how heat and smoke move through a multi-story residential structure with a heating, ventilation, and air conditioning (HVAC) sys- tem under a range of initial ventilation and fire conditions. The ultimate objective is to provide data that increases the understanding of how a typical residential HVAC system impacts the flow of fire gases within a structure.

## 2 Data Description

The github repository contains the data and supporting files from gas burner experiments conducted in single-story ranch-style structure with an above ground basement. The repository contains directories for the raw data and event timestamps, supporting information for the exper- iments, and python scripts for visualizing the data.

### 2.1 Data

The data directory (01_Data) contains two comma-separated value (CSV) plaintext files for each experiment. The name of the file containing the time series measurement data from each experiment matches the name of the experiment. The second file for each experiment includes the experiment name followed by Events and contains the timing information of notable events from the experiment.

### 2.2 Information

The information directory (02_Info/) contains CSV files that provide high-level informa- tion about the experiments and support the plotting script contained in the 03_Scripts/ di- rectory. The info file (Info_Gas_Burner) provides information regarding the fire location, the status of the HVAC system, the position of interior vents, and axis scales for plotting. Additional CSV files (Channel_List_Gas_Burner and Channel_List_Gas_Burner_2) list the details about each of the sensors including the location of the sensor, data type measured, and the ar- ray with which each sensor is grouped. Two files are used to account for the movement of gas concentrations measurement locations within a subset of the living room burner location exper- iments. A subdirectory labeled ‘Floorplans’ is included which includes dimensioned floorplans of the structure, dimensioned floorplans of the heating, ventilation, and air conditioning (HVAC) supply and return vents, and dimensioned floorplans of instrumentation locations.

### 2.3 Scripts

The scripts directory (03_Scripts/) contains a single Python script that, when executed, generates time history charts of sensor data from each experiment. The script creates a charts directory (04_Charts/) on the same level as the scripts directory. Directories corresponding to the different experiments are created within the charts directory and then populated with portable document format (PDF) files that contain the experimental data plots from the various sensor groups.

## 3 Experimental Design, Materials and Methods

Twenty-nine propane gas burner experiments conducted in a single-story ranch-style resi- dential structure with an above ground basement originally built to study firefighter suppression tactics [1]. For this experimental series, the structure was gutted and retrofitted to include an HVAC system. The experiments were conducted to determined how the HVAC system status (i.e., operating versus off), fire location, fire heat release rate, and bedroom door status (i.e., open vs. closed) would or would not impact heat and gas species transfer within the structure.

### 3.1 Fire Source

A gas burner, supplied by two 420 lb/120 gal propane tanks, was used as the fire source for these experiments. The burner was 0.6 m (2.0 ft) by 0.6 m (2.0 ft) with the top surface 0.5 m (20 in.) above the floor. A mass flow controller was used to set the heat release rate of the fires. Three mass flow rates of propane were used — 75 LPM, 190 LPM, and 245 LPM, which corresponded to approximate heat release rates of 110 kW, 280 kW, and 360 kW, respectively. Combining the contributing uncertainties in the experimental setup, yielded estimated expanded uncertainties in heat release rates that ranged from ± 19% at 110 kW to ± 7% at 360 kW.

### 3.2 Experimental Structure

The structure was a ranch-style house with an above ground basement. To simulate the insu- lation of a basement, the perimeter wall of the ground level (i.e., the basement) was composed of interlocking concrete blocks that were 0.6 m (2.0 ft) wide, 0.6 m (2.0 ft) high, and 1.2 m (4.0 ft) long. Gaps where the blocks joined together were filled with high-temperature spray insulation. The basement was framed with dimensional lumber, 3.81 cm by 8.89 cm (2 × 4 studs) that were lined with 1.27 cm (0.5 in.) drywall. R-13 fiberglass insulation was added to the stud bays be- tween the concrete block and drywall. The resulting interior dimensions of the basement were 13.18 m (43.2 ft) wide, 7.07 m (23.0 ft) long, and 2.74 m (9.0 ft) high.

**Fig. 1:**
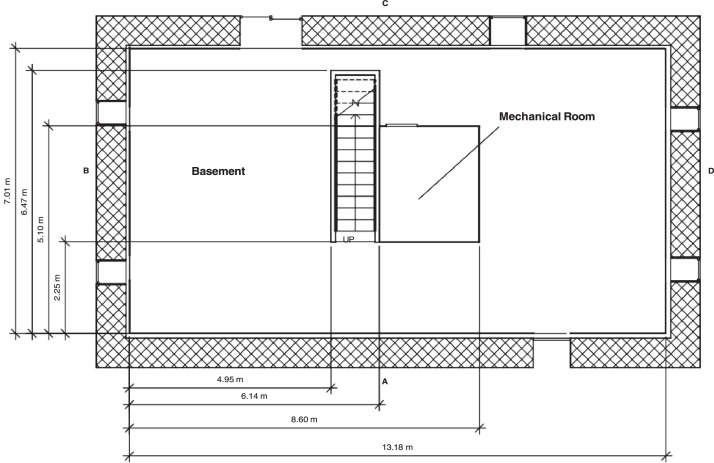
Dimensions of basement [1,2].

Note: In Fig. 1 through 6, dimensions are given with respect to the interior A/B corner. The first-floor interior space overhung the interior of the concrete block basement wall. As a result, the first-floor interior A/B corner is offset 29.5 cm (11.6 in.) in the x axis and 34.5 cm (13.6 in.) in the y axis.

The first floor was built on top of engineered lumber I-joists that had 40.6 cm (16 in.) joint depths. On the bottom side of the joists was a single layer of 1.27 cm (0.5 in.) drywall that formed the basement ceiling. On top of the I-joists was a layer of 1.27 cm (0.5 in.) oriented strand board (OSB) and 1.27 cm (0.5 in.) cement board which served as floor for the first story. Though closed for all experiments, the basement had two exterior vents. The side C basement window was 1.0 m (39 in.) wide by 0.85 m (33 in.) tall with a 1.52 m (60 in) sill height and the side C basement slider was 1.83 m (72 in.) wide by 2.05 m (81 in.) tall. The layout and dimensions of the basement are included in Fig. 1.

Similar the basement walls, the first-floor walls were constructed from nominal dimension lumber, 3.81 cm by 8.89 cm (2 × 4 studs). The exterior of the first-floor walls were finished with 8 mm (0.31 in.) thick fiber cement board siding on top of a layer of olefin home wrap and 1.27 cm (0.5 in.) OSB. The interior of the studs were lined with 1.27 cm (0.5 in.) drywall with R-13 fiberglass insulation in the stud bays. The resulting interior dimensions of the first floor measured 13.77 m (45.2 ft) by 7.70 m (25.3 ft) with a 2.44 m (8 ft) ceiling.

**Fig. 2:**
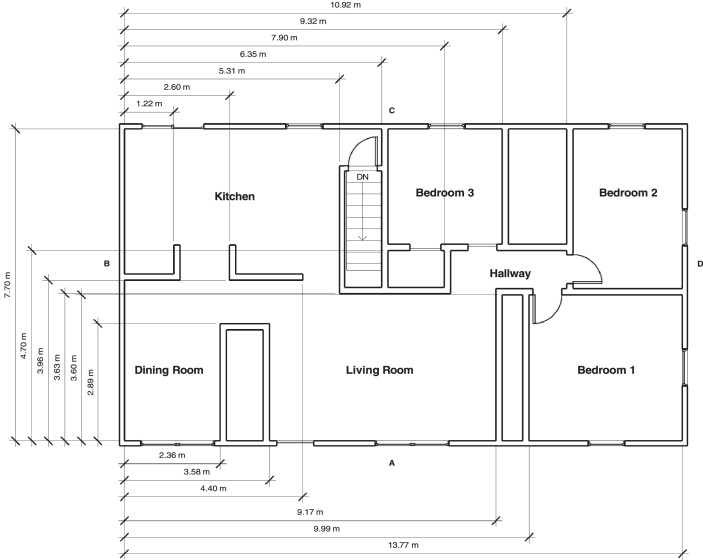
Dimensions of first floor [1,2].

The first-floor ceiling had a single layer of 1.27 cm (0.5 in.) drywall over nominal dimension lumber wood trusses with R-38 fiberglass insulation between the trusses. The layout and dimen- sions of the first floor are included in Fig. 2. The front door was 0.91 m (36 in.) by 2.03 m (80 in.) and was toggled open for a subset of the experiments. The remaining first-floor vents remained closed until after the fire was extingished, when they were opened to aid in post-fire ventilation. The single windows in the kitchen and bedrooms were 0.91 m (36 in.) wide by 1.5 m (60 in.) tall with a sill height of 0.61 m (24 in.). The double windows in the dining room and living room were 1.83 m (72 in.) wide by 1.5 m (60 in.) tall with a sill height of 0.61 m (24 in.). The kitchen slider was 1.83 m (72 in.) wide by 2.05 m (81 in.) tall.

To characterize the structure's leakage, ASTM E 779, “Standard Test Method for Determin- ing Air Leakage Rate by Fan Pressurization,” was followed. With all exterior vents closed, the structure had 8.3 air changes per hour (ACPH) at 50 Pa (0.007 psi) and an equivalent leakage area of approximately 0.14 m^2^ (1.5 ft^2^).

#### 3.2.1 Heating, Ventilation, and Air Conditioning System

The HVAC system added to the structure for these experiments used metal and flex ductwork to transport gases throughout the structure. The system included an 18 kW heater with a heating/cooling capacity of 10.5 kW (36,000 BTU/hr) using R410A refrigerant with a 0.37 kW (1/2 horsepower) motor capable of moving 2040 m^3^/hr (1200 scfm) or air. The condensing unit was located at ground level along the exterior side D wall of the structure and the furnace was in the basement mechanical room.

**Fig. 3:**
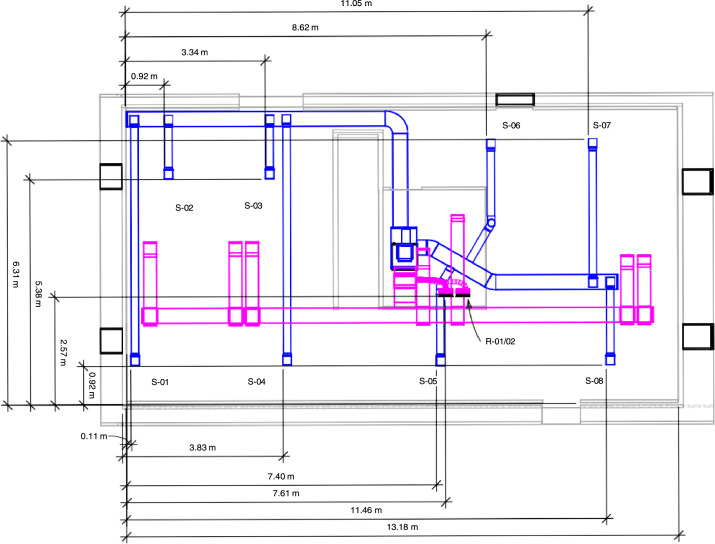
Basement HVAC supply and return vent layout [2]. The vents are sequentially numbered using S-XX and R-YY for supply (blue) and return (pink) vents, respectively. Numbering is initiated in the basement and carries over to the first floor.

The system supply, which originated in the basement mechanical room, split into three sep- arate ducts: two fed the basement and one fed the first floor. Two basement branches, which measured 36 cm by 20 cm (14 in. by 8 in.), fed eight, 18 cm (7 in.) diameter ducts that led to the basement supply vents installed in the ceiling. Except for two supply vents in the C/D quadrant of the basement that measured 28 cm by 28 cm (11 in. by 11 in.), the basement supply vents measured 24 cm by 24 cm (9-3/8 in. by 9-3/8 in). Labeled and dimensioned basement vents are provided in Fig. 3.

**Fig. 4:**
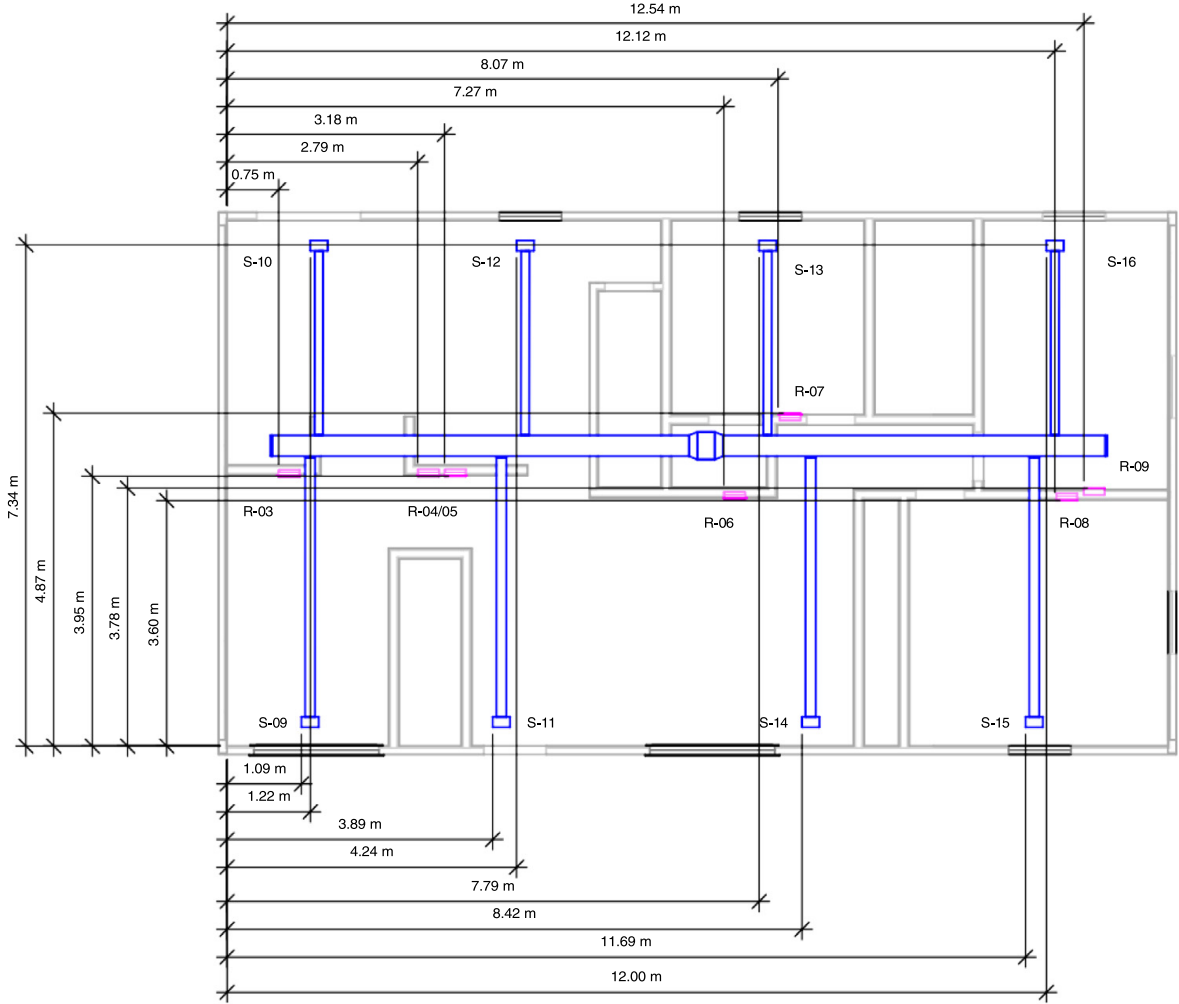
First floor HVAC supply and return vent layout [2]. The vents are sequentially numbered using S-XX and R-YY for supply (blue) and return (pink) vents, respectively. Numbering is initiated in the basement and carries over to the first floor.

The first-floor supply duct ran from the basement through the bedroom 3 closet on the first floor and into the attic. The 40 cm by 20 cm (16 in. by 8 in.) duct split into two branches in the attic; each measured 30 cm by 20 cm (12 in. by 8 in.). There were four 15 cm (6 in.) diameter ducts that connected to each of the two branches in the attic. These ducts connected the eight first-floor supply vents that were installed in the ceiling. Each first-floor supply vent was 30 cm by 15 cm (12 in. by 6 in.). Labeled and dimensioned first floor vents are provided in Fig. 4.

There were seven return vents in the structure, two of which were double returns (R-01/02 in Fig. 3 and R-04/05 in Fig. 4). The basement return vent was 1.6 m (63 in.) above the floor and the first-floor return vents were 21.75 cm (8.6 in.) above the floor. All single return vents measured 36 cm by 25 cm (14 in. by 10 in.). The double return vents measured 66 cm by 25 cm (26 in. by 10 in.). Each return vent fed into a plenum space consisting of either a floor joist space or a wall stud space. The plenum spaces connected to return ductwork that measured 36 cm by 20 cm (14 in. by 8 in.). All return ducts ran back to single duct in the basement mechanical room (51 cm by 25 cm (20 in. by 10 in.) that connected to the furnace.

### 3.3 Instrumentation

**Fig. 5:**
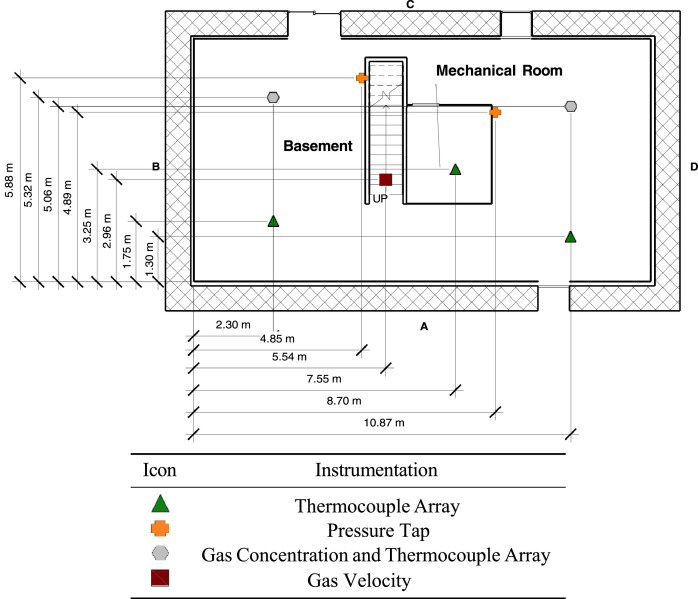
Basement instrumentation layout [2].

**Fig. 6:**
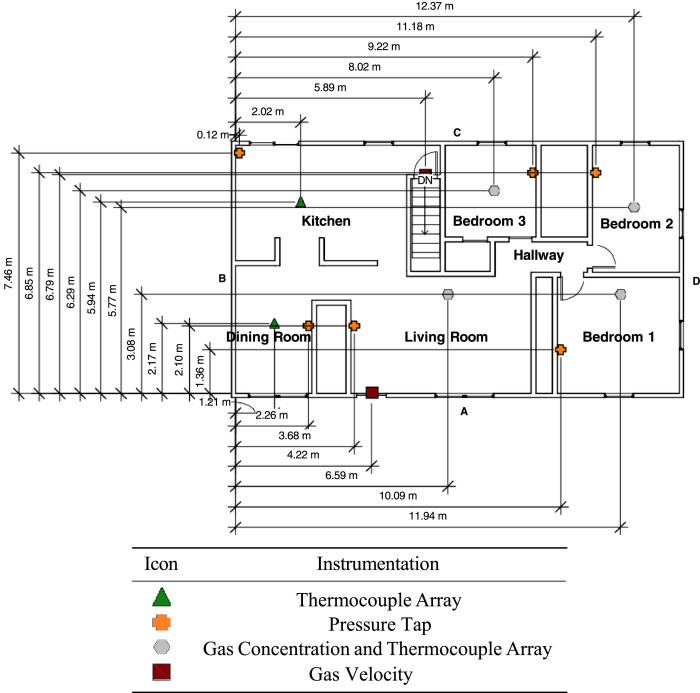
First floor instrumentation layout [2].

Measurements of gas temperature, gas pressure, gas velocity, and gas concentrations were made within the structure using instrumentation specifically designed for the hazards of a typical fire environment. Fig. 5 provides the layout of the basement and Fig. 6 provides a layout of the first-floor instrumentation. The xyz coordinate triple for the location of each instrument is included in the channel list files in the 02_Info/ directory. The coordinates are based on ground level at A/B corner (front left) of each respective floor being set to (0,0,0).

Gas temperatures were measured with 1.3 mm diameter bare-bead Chromel-Alumel (type- K) thermocouples. Five bare-bead thermocouple arrays were installed in the basement of the structure. The basement was subdivided into four corners, or quadrants, and a thermocouple tree was placed in the middle of each corner. One thermocouple tree was also placed in the mechanical room. Each thermocouple tree in the basement consisted of nine thermocouples. The top thermocouple was 2.54 cm (1 in.) below the ceiling and subsequent thermocouples were spaced 30.5 cm (1 ft) apart descending toward the floor. The bottom thermocouple was 2.44 m (8 ft) below the ceiling.

Six thermocouple arrays were installed on the first floor of the structure. Each thermocouple tree on the first floor consisted of eight thermocouples. Similar to the basement, the top ther- mocouple was 2.54 cm below (1 in.) the ceiling. The subsequent thermocouples were spaced 30.5 cm (1 ft) apart descending toward the floor. The bottom thermocouple was 2.13 m (7 ft) below the ceiling.

In addition to the thermocouple arrays, single bare-bead thermocouples were installed at the supply and return registers in the basement and first floor, at the thermostat on the hallway wall outside of bedroom 3 (1.2 m (4 ft) above the floor), and in the first-floor HVAC supply trunk that ran through the bedroom 3 closet (1.52 m (5 ft) above the first floor).

Differential pressure transducers were used to measure the pressure increase or decrease relative to the exterior (i.e., ambient). The high side of the differential pressure transducers (measurement range of ± 125 Pa) via polyvinyl tubing were connected to copper sampling probes throughout the structure (Figs. 5 and 6). The low side of transducer was connected to polyvinyl tubing ran to the exterior of the structure. Pressure taps were installed in two locations in the basement and at six locations on the first floor. Each location had three pressure taps, the first was 30.5 cm (1 ft) below the ceiling, the second at 1.22 m (4 ft) below the ceiling, and the third at 2.13 m (7 ft) below the ceiling. The taps extended from the wall by approximately 15 cm.

Sheathed thermocouples were used in conjunction with bi-directional probes for gas velocity measurements. To determine magnitude and direction of the flow, the bi-directional probes were connected to both the high and low input of a differential pressure transducer. Velocity was mea- sured at three locations: front door, top basement stairwell, and bottom of basement stairwell. Arrays of five probes were installed at the top of the stairwell and the front door. The bottom probe at both locations was installed 0.36 m (1.2 ft) above the floor and were spaced 30.5 cm (1 ft) apart. At the bottom of the stairwell, an array of six probes were installed starting at 1 m (3 ft) above the floor and spaced 30.5 cm (1 ft) apart.

Carbon dioxide (CO_2_), oxygen (O_2_), and water vapor (H_2_O) gas concentrations were mea- sured at multiple locations based on the specific experiments. The locations of the gas mea- surements as a function of experiment number are included in Table 1. For CO_2_ and O_2_ mea- surements, gases were extracted, cooled, and filtered to remove moisture and particulates before being analyzed using a non-dispersive infrared (NDIR) gas analyzer and paramagnetic oxygen sensor, respectively. Both analyzers had a range of 0 to 25% by volume and resolution of 0.01%. H_2_O measurements were made in-situ using an infrared tunable diode laser absorption spec- troscopy (IR-TDLAS) based sensor. All gases were measured 1.22 m (4 ft) above the floor.

**Table 1:** Gas measurement locations.

Exp #s Fire Room	CO_2_ & O_2_	H_2_O
01–14 Bedroom (BR) 1	BRs 1, 2, 3 and BSMT Side D	BRs 2, 3 and BSMT Side D
15–18 Living Room (LR)	LR, BRs 2, 3 and BSMT Side D	BRs 2, 3 and BSMT Side D
19–21 Living Room (LR)	BRs 1, 2, 3 and BSMT Side D	BRs 2, 3 and BSMT Side D
22–26 Basement (BSMT)	BRs 1, 2, 3 and BSMT Side B	BRs 2, 3 and BSMT Side B
27–31 Basement (BSMT)	BRs 1, 2, 3 and BSMT Side B	—

Numerical data was recorded with a National Instruments data acquisition system. The sys- tem consisted of a PXIe-1085 chassis, a PXIe-8840 controller, PXIe-4353 temperature input modules, and PXIe-6365 multifunction I/O modules (± 10 V). The PXIe-4353 modules were connected to TC-4353 rack mount adapters that provided built-in cold junction compensation for thermocouples. The PXIe-6365 modules were connected to SCB-68A connector blocks that allowed for interfacing with the input voltage signals. Data were sampled at 1 Hz for all experi- ments.

A full description of each experiment can be found in work performed by Quiat [2].

## Ethics Statements

The authors declare that there are no ethical issues with the data presented. This project does not involve human subjects, animal experiments, or data collected from social media platforms.

## CRediT authorship contribution statement

**Craig Weinschenk:** Writing – original draft, Data curation, Investigation, Formal analysis. **Shruti Ghanekar:** Investigation, Formal analysis. **Keith Stakes:** Conceptualization, Investigation, Resources. **Adam Quiat:** Investigation. **Richard M. Kessler:** Investigation, Resources. **Tonghun Lee:** Supervision.

## Declaration of Competing Interest

The authors declare that they have no known competing financial interests or personal relation- ships which have, or could be perceived to have, influenced the work reported in this article.

## Data Availability

2019 Gas Burner HVAC Experimental Data (Original data) (Github).

## Appendix Supplementary materials

Image, application 1

## References

[1] Madryzkowski D., Weinschenk C. (2018).

[2] Quiat A. (2020). https://drum.lib.umd.edu/handle/1903/26719.

