# Supplementary material for: Experimental data from gas burner fires in residential structure with HVAC system
Source: Data Brief. 2022 Dec 14;46:108825. doi: 10.1016/j.dib.2022.108825 (PMC9792339; doi:10.1016/j.dib.2022.108825)
Supplement: Supplementary file 1 [file mmc1.pdf]

ABSTRACT

Title of Thesis: Analysis of Propane Gas Burner Experiments and FDS Simulations in a Two-Story Residential Structure with HVAC

Adam Quiat
Masters of Science, 2020

Thesis directed by: Dr. James A Milke
Department of Fire Protection Engineering

To further our knowledge of fire dynamics in a residential structure with a heating, venitilation, and air conditioning (HVAC) system, a series of live fire gas burner experiments were conducted and modeling simulations were completed to compare the results. 29 full-scale experiments were conducted in a single story ranch structure on a basement with fire from a single propane fed gas burner. The structure was instrumented to measure temperatures, oxygen and carbon dioxide concentrations, water vapor concentrations, and gas velocities. In addition to the full-scale experiments, six experiments were selected and modeled for comparison. A numerical simulation program, known as a Fire Dynamics Simulator (FDS) (version 6.7.10), was used to complete the computational fluid dynamics (CFD) calculations. The FDS HVAC submodel was incorporated into the modeled experiments to compare how heat and fire gases transversed the modeled HVAC system as compared

to the experimental results. Results from experimental and simulated data showed that heat and fire gases were transferred through the HVAC system during the experiments. Comparison of simulation data to the experimental data showed that FDS over predicted heat transfer through the HVAC system. However, there was sufficient agreement between data points to support FDS modeling as a more severe set of outcomes in the event of a fire.

Analysis of Propane Gas Burner Experiments and FDS Simulations in a Two-Story Residential Structure with HVAC

by

Adam Quiat

Thesis submitted to the Faculty of the Graduate School of the
University of Maryland, College Park in partial fulfillment
of the requirements for the degree of
Masters of Science
2020

Advisory Committee:
Dr. James A Milke, Chair/Advisor
Dr. Arnaud Trouvé, Professor/Defense Committee
Dr. Craig Weinschenk, UL FSRI/Defense Committee

© Copyright by
Adam Quiat
2020

Acknowledgments

There are several individuals and groups that deserve recognition. First, I need to thank the entire team at Underwriters Laboratories Firefighter Safety Research Institute for their help with planning, executing and guiding me through my thesis. Second, I need to thank my advisory committee including Professor James Milke, Professor Arnaud Trouvé and Dr. Craig Weinschenk from UL FSRI. A special thanks goes to Dr. Randall McDermott from the National Institute of Standards and Technology for his wisdom and advice on the subject of modeling. Finally, I would like to thank the College Park Volunteer Fire Department for their continued support and motivation throughout this process.

Table of Contents

Acknowledgments ii

List of Tables vi

List of Figures viii

List of Abbreviations xiv

1 Introduction 1
- 1.1 Experimentation 2
- 1.2 HVAC Basics 2
- 1.3 Computational Modeling 4
- 1.4 Objectives and Overview 7

2 Experimental Setup 9
- 2.1 Experimental Structure 9
  - 2.1.1 HVAC 11
- 2.2 Instrumentation 15
  - 2.2.1 Basement 17
  - 2.2.2 First Floor 20
- 2.3 Gas Burner Setup 21
- 2.4 Experimental Scenarios 24

3 Experimental Procedure 27

4 Modeling Setup 34

5 Discussion of Experimental Results 41
- 5.1 Bedroom 1 Experiments 41
  - 5.1.1 Experiment 1 42
  - 5.1.2 Experiment 2 52
  - 5.1.3 Experiment 3 64
  - 5.1.4 Experiment 4 73

5.1.5 Experiment 5 84
5.1.6 Experiment 6 91
5.1.7 Experiment 7 99
5.1.8 Experiment 8 108
5.1.9 Experiment 9 118
5.1.10 Experiment 10 127
5.1.11 Experiment 11 136
5.1.12 Experiment 12 144
5.1.13 Experiment 13 153
5.1.14 Experiment 14 162
5.2 Living Room Experiments 171
5.2.1 Experiment 15 172
5.2.2 Experiment 16 182
5.2.3 Experiment 17 192
5.2.4 Experiment 18 202
5.2.5 Experiment 19 211
5.2.6 Experiment 20 220
5.2.7 Experiment 21 228
5.3 Basement Experiments 236
5.3.1 Experiment 23 237
5.3.2 Experiment 24 248
5.3.3 Experiment 25 258
5.3.4 Experiment 26 269
5.3.5 Experiment 27 280
5.3.6 Experiment 28 290
5.3.7 Experiment 29 299
5.4 HVAC Status Comparison 308

6 Discussion of Modeling Results 316
6.1 Cold Flow Analysis 316
6.2 Experimental Simulation Analysis 318
6.2.1 Experiment 1 318
6.2.2 Experiment 12 328
6.2.3 Experiment 15 338
6.2.4 Experiment 16 349
6.2.5 Experiment 23 359
6.2.6 Experiment 25 369

7 Conclusions 380
7.1 Future Work 382

A Cold Flow Losses 384

B DelCo Test Structure 418

C FDS Experiment 1 Script 430

Bibliography 477

List of Tables

2.1 List of Bedroom 1 Experiments Performed . . . 24
2.2 List of Living Room Experiments Performed . . . 25
2.3 List of Basement Experiments Performed . . . 26

4.1 Experiment Events of Interest, time in seconds . . . 40

5.1 Experiment 1 Events . . . 42
5.2 Experiment 2 Events . . . 53
5.3 Experiment 3 Events . . . 65
5.4 Experiment 4 Events . . . 74
5.5 Experiment 5 Events . . . 85
5.6 Experiment 6 Events . . . 92
5.7 Experiment 7 Events . . . 100
5.8 Experiment 8 Events . . . 109
5.9 Experiment 9 Events . . . 119
5.10 Experiment 10 Events . . . 128
5.11 Experiment 11 Events . . . 137
5.12 Experiment 12 Events . . . 145
5.13 Experiment 13 Events . . . 154
5.14 Experiment 14 Events . . . 163
5.15 Experiment 15 Events . . . 172
5.16 Experiment 16 Events . . . 183
5.17 Experiment 17 Events . . . 193
5.18 Experiment 18 Events . . . 203
5.19 Experiment 19 Events . . . 212
5.20 Experiment 20 Events . . . 221
5.21 Experiment 21 Events . . . 229
5.22 Experiment 23 Events . . . 237
5.23 Experiment 24 Events . . . 249
5.24 Experiment 25 Events . . . 258
5.25 Experiment 26 Events . . . 270
5.26 Experiment 27 Events . . . 281
5.27 Experiment 28 Events . . . 291

5.28 Experiment 29 Events . . . 300
5.29 Bedroom 1 HVAC Status Comparison . . . 310
5.30 Living Room HVAC Status Comparison . . . 312
5.31 Basement HVAC Status Comparison . . . 314

6.1 Experiment 1 Comparisons . . . 328
6.2 Experiment 12 Comparisons . . . 337
6.3 Experiment 15 Comparisons . . . 348
6.4 Experiment 16 Comparisons . . . 358
6.5 Experiment 23 Comparisons . . . 369
6.6 Experiment 25 Comparisons . . . 379

7.1 FDS Simulation Differential Comparisons . . . 382

A.1 Flow Hood Supply Vent Results . . . 385
A.2 Flow Hood Return Vent Results . . . 386
A.3 ASHRAE 1 Supply System . . . 388
A.4 ASHRAE 1 Return System . . . 389
A.5 ASHRAE 1 Supply Results . . . 390
A.6 ASHRAE 1 Return Results . . . 391
A.7 Minor Supply Losses ASHRAE 1 & 2 . . . 393
A.8 Minor Return Losses ASHRAE 1 & 2 . . . 394
A.9 ASHRAE 2 Supply Results . . . 395
A.10 ASHRAE 2 Return Results . . . 396
A.11 Minor Supply Losses ASHRAE 1, 2, & 3 . . . 397
A.12 Minor Return Losses ASHRAE 1, 2, & 3 . . . 398
A.13 Minor Supply Losses ASHRAE 4 . . . 400
A.14 Minor Return Losses ASHRAE 4 . . . 401
A.15 ASHRAE 4 Supply Results . . . 403
A.16 ASHRAE 4 Return Results . . . 404
A.17 Minor Supply Losses ASHRAE 5 . . . 405
A.18 Minor Return Losses ASHRAE 5 . . . 406
A.19 Minor Supply Losses ASHRAE 6 . . . 408
A.20 Minor Return Losses ASHRAE 6 . . . 409
A.21 Minor Supply Losses ASHRAE 7 . . . 410
A.22 Minor Return Losses ASHRAE 7 . . . 411
A.23 ASHRAE 7 Supply Results . . . 412
A.24 ASHRAE 7 Return Results . . . 413

B.1 TC Tree List . . . 425
B.2 Single Thermalcouple List . . . 426
B.3 Gas Concentration List . . . 427
B.4 Bi-Directional Probe List . . . 428
B.5 Pressure Tap List . . . 429

List of Figures

2.1 Basement Plan . . . 10
2.2 First Floor Plan . . . 11
2.3 Basement HVAC . . . 13
2.4 First Floor HVAC . . . 15
2.5 Basement Instrumentation . . . 19
2.6 First Floor Instrumentation . . . 21
2.7 Top: Basement Gas Burner, Middle: First Floor Gas Burner Locations, Bottom: Gas Burner Legend . . . 23

3.1 Experiment Instrumentation . . . 28
3.2 Propane Path and Safety Features . . . 30
3.3 Propane Pilot Line . . . 31

4.1 FDS Smokeview Outputs . . . 36

5.1 Experiment 1 First Floor Temperatures . . . 44
5.2 Experiment 1 Basement Temperatures . . . 45
5.3 Experiment 1 Pressures . . . 47
5.4 Experiment 1 Gases . . . 49
5.5 Experiment 1 Vent Temperatures . . . 50
5.6 Experiment 1 Supply Duct Temperature and Velocity . . . 52
5.7 Experiment 2 First Floor Temperatures . . . 55
5.8 Experiment 2 Basement Temperatures . . . 56
5.9 Experiment 2 Pressures . . . 58
5.10 Experiment 2 Gases . . . 60
5.11 Experiment 2 Vent Temperatures . . . 62
5.12 Experiment 2 Supply Duct Temperature and Velocity . . . 64
5.13 Experiment 3 First Floor Temperatures . . . 66
5.14 Experiment 3 Basement Temperatures . . . 67
5.15 Experiment 3 Pressures . . . 69
5.16 Experiment 3 Gases . . . 71
5.17 Experiment 3 Vent Temperatures . . . 72
5.18 Experiment 3 Supply Duct Temperature and Velocity . . . 73

5.19 Experiment 4 First Floor Temperatures 76
5.20 Experiment 4 Basement Temperatures 77
5.21 Experiment 4 Pressures 79
5.22 Experiment 4 Gases 81
5.23 Experiment 4 Vent Temperatures 82
5.24 Experiment 4 Supply Duct Temperature and Velocity 84
5.25 Experiment 5 First Floor Temperatures 86
5.26 Experiment 5 Basement Temperatures 87
5.27 Experiment 5 Pressures 88
5.28 Experiment 5 Gases 89
5.29 Experiment 5 Vent Temperatures 90
5.30 Experiment 5 Supply Duct Temperature and Velocity 91
5.31 Experiment 6 First Floor Temperatures 93
5.32 Experiment 6 Basement Temperatures 94
5.33 Experiment 6 Pressures 95
5.34 Experiment 6 Gases 97
5.35 Experiment 6 Vent Temperatures 98
5.36 Experiment 6 Supply Duct Temperature and Velocity 99
5.37 Experiment 7 First Floor Temperatures 101
5.38 Experiment 7 Basement Temperatures 102
5.39 Experiment 7 Pressures 104
5.40 Experiment 7 Gases 106
5.41 Experiment 7 Vent Temperatures 107
5.42 Experiment 7 Supply Duct Temperature and Velocity 108
5.43 Experiment 8 First Floor Temperatures 110
5.44 Experiment 8 Basement Temperatures 111
5.45 Experiment 8 Pressures 113
5.46 Experiment 8 Gases 115
5.47 Experiment 8 Vent Temperatures 117
5.48 Experiment 8 Supply Duct Temperature and Velocity 118
5.49 Experiment 9 First Floor Temperatures 120
5.50 Experiment 9 Basement Temperatures 121
5.51 Experiment 9 Pressures 123
5.52 Experiment 9 Gases 125
5.53 Experiment 9 Vent Temperatures 126
5.54 Experiment 9 Supply Duct Temperature and Velocity 127
5.55 Experiment 10 First Floor Temperatures 129
5.56 Experiment 10 Basement Temperatures 130
5.57 Experiment 10 Pressures 132
5.58 Experiment 10 Gases 133
5.59 Experiment 10 Vent Temperatures 135
5.60 Experiment 10 Supply Duct Temperature and Velocity 136
5.61 Experiment 11 First Floor Temperatures 138
5.62 Experiment 11 Basement Temperatures 139
5.63 Experiment 11 Pressures 141

5.64 Experiment 11 Gases 142
5.65 Experiment 11 Vent Temperatures 143
5.66 Experiment 11 Supply Duct Temperature and Velocity 144
5.67 Experiment 12 First Floor Temperatures 146
5.68 Experiment 12 Basement Temperatures 147
5.69 Experiment 12 Pressures 149
5.70 Experiment 12 Gases 151
5.71 Experiment 12 Vent Temperatures 152
5.72 Experiment 12 Supply Duct Temperature and Velocity 153
5.73 Experiment 13 First Floor Temperatures 155
5.74 Experiment 13 Basement Temperatures 156
5.75 Experiment 13 Pressures 158
5.76 Experiment 13 Gases 159
5.77 Experiment 13 Vent Temperatures 161
5.78 Experiment 13 Supply Duct Temperature and Velocity 162
5.79 Experiment 14 First Floor Temperatures 164
5.80 Experiment 14 Basement Temperatures 165
5.81 Experiment 14 Pressures 167
5.82 Experiment 14 Gases 168
5.83 Experiment 14 Vent Temperatures 170
5.84 Experiment 14 Supply Duct Temperature and Velocity 171
5.85 Experiment 15 First Floor Temperatures 174
5.86 Experiment 15 Basement Temperatures 175
5.87 Experiment 15 Pressures 177
5.88 Experiment 15 Gases 179
5.89 Experiment 15 Vent Temperatures 181
5.90 Experiment 15 Supply Duct Temperature and Velocity 182
5.91 Experiment 16 First Floor Temperatures 184
5.92 Experiment 16 Basement Temperatures 185
5.93 Experiment 16 Pressures 187
5.94 Experiment 16 Bedroom Gases 189
5.95 Experiment 16 Vent Temperatures 191
5.96 Experiment 16 Supply Duct Temperature and Velocity 192
5.97 Experiment 17 First Floor Temperatures 194
5.98 Experiment 17 Basement Temperatures 195
5.99 Experiment 17 Pressures 197
5.100 Experiment 17 Bedroom Gases 199
5.101 Experiment 17 Vent Temperatures 201
5.102 Experiment 17 Supply Duct Temperature and Velocity 202
5.103 Experiment 18 First Floor Temperatures 204
5.104 Experiment 18 Basement Temperatures 205
5.105 Experiment 18 Pressures 207
5.106 Experiment 18 Bedroom Gases 209
5.107 Experiment 18 Vent Temperatures 210
5.108 Experiment 18 Supply Duct Temperature and Velocity 211

5.109Experiment 19 First Floor Temperatures 213
5.110Experiment 19 Basement Temperatures 214
5.111Experiment 19 Pressures 216
5.112Experiment 19 Bedroom Gases 218
5.113Experiment 19 Vent Temperatures 219
5.114Experiment 19 Supply Duct Temperature and Velocity 220
5.115Experiment 20 First Floor Temperatures 222
5.116Experiment 20 Basement Temperatures 223
5.117Experiment 20 Pressures 224
5.118Experiment 20 Bedroom Gases 226
5.119Experiment 20 Vent Temperatures 227
5.120Experiment 20 Supply Duct Temperature and Velocity 228
5.121Experiment 21 First Floor Temperatures 230
5.122Experiment 21 Basement Temperatures 231
5.123Experiment 21 Pressures 232
5.124Experiment 21 Bedroom Gases 234
5.125Experiment 21 Vent Temperatures 235
5.126Experiment 21 Supply Duct Temperature and Velocity 236
5.127Experiment 23 Basement Temperatures 239
5.128Experiment 23 First Floor Temperatures 241
5.129Experiment 23 Pressures 243
5.130Experiment 23 Bedroom Gases 245
5.131Experiment 23 Vent Temperatures 246
5.132Experiment 23 Supply Duct Temperature and Velocity 248
5.133Experiment 24 Basement Temperatures 250
5.134Experiment 24 First Floor Temperatures 252
5.135Experiment 24 Pressures 254
5.136Experiment 24 Bedroom Gases 255
5.137Experiment 24 Vent Temperatures 257
5.138Experiment 24 Supply Duct Temperature and Velocity 258
5.139Experiment 25 Basement Temperatures 260
5.140Experiment 25 First Floor Temperatures 262
5.141Experiment 25 Pressures 264
5.142Experiment 25 Bedroom Gases 266
5.143Experiment 25 Vent Temperatures 267
5.144Experiment 25 Supply Duct Temperature and Velocity 269
5.145Experiment 26 Basement Temperatures 271
5.146Experiment 26 First Floor Temperatures 273
5.147Experiment 26 Pressures 275
5.148Experiment 26 Bedroom Gases 277
5.149Experiment 26 Vent Temperatures 279
5.150Experiment 26 Supply Duct Temperature and Velocity 280
5.151Experiment 27 Basement Temperatures 282
5.152Experiment 27 First Floor Temperatures 284
5.153Experiment 27 Pressures 286

5.154 Experiment 27 Bedroom Gases . . . 287
5.155 Experiment 27 Vent Temperatures . . . 289
5.156 Experiment 27 Supply Duct Temperature and Velocity . . . 290
5.157 Experiment 28 Basement Temperatures . . . 292
5.158 Experiment 28 First Floor Temperatures . . . 294
5.159 Experiment 28 Pressures . . . 296
5.160 Experiment 28 Bedroom Gases . . . 297
5.161 Experiment 28 Vent Temperatures . . . 298
5.162 Experiment 28 Supply Duct Temperature and Velocity . . . 299
5.163 Experiment 29 Basement Temperatures . . . 301
5.164 Experiment 29 First Floor Temperatures . . . 303
5.165 Experiment 29 Pressures . . . 305
5.166 Experiment 29 Bedroom Gases . . . 306
5.167 Experiment 29 Vent Temperatures . . . 307
5.168 Experiment 29 Supply Duct Temperature and Velocity . . . 308

6.1 FDS Experiment 1 First Floor Temperatures . . . 320
6.2 FDS Experiment 1 Basement Temperatures . . . 321
6.3 FDS Experiment 1 Pressures . . . 323
6.4 FDS Experiment 1 Gases . . . 325
6.5 FDS Experiment 1 Vent Temperatures . . . 327
6.6 FDS Experiment 12 First Floor Temperatures . . . 329
6.7 FDS Experiment 12 Basement Temperatures . . . 330
6.8 FDS Experiment 12 Pressures . . . 332
6.9 FDS Experiment 12 Gases . . . 334
6.10 FDS Experiment 12 Vent Temperatures . . . 336
6.11 FDS Experiment 12 Supply Duct Temperature and Velocity . . . 337
6.12 FDS Experiment 15 First Floor Temperatures . . . 340
6.13 FDS Experiment 15 Basement Temperatures . . . 341
6.14 FDS Experiment 15 Pressures . . . 343
6.15 FDS Experiment 15 Gases . . . 345
6.16 FDS Experiment 15 Vent Temperatures . . . 347
6.17 FDS Experiment 15 Supply Duct Temperature and Velocity . . . 348
6.18 FDS Experiment 16 First Floor Temperatures . . . 350
6.19 FDS Experiment 16 Basement Temperatures . . . 351
6.20 FDS Experiment 16 Pressures . . . 353
6.21 FDS Experiment 16 Gases . . . 355
6.22 FDS Experiment 16 Vent Temperatures . . . 357
6.23 FDS Experiment 16 Supply Duct Temperature and Velocity . . . 358
6.24 FDS Experiment 23 Basement Temperatures . . . 360
6.25 FDS Experiment 23 First Floor Temperatures . . . 362
6.26 FDS Experiment 23 Pressures . . . 364
6.27 FDS Experiment 23 Gases . . . 366
6.28 FDS Experiment 23 Vent Temperatures . . . 367
6.29 FDS Experiment 23 Supply Duct Temperature and Velocity . . . 368

6.30 FDS Experiment 25 Basement Temperatures 370
6.31 FDS Experiment 25 First Floor Temperatures 372
6.32 FDS Experiment 25 Pressures 374
6.33 FDS Experiment 25 Gases 376
6.34 FDS Experiment 25 Vent Temperatures 377
6.35 FDS Experiment 25 Supply Duct Temperature and Velocity 378

A.1 ASHRAE Losses 415
A.2 Supply Flow Comparison 416
A.3 Return Flow Comparison 417

B.1 Basement Plan 419
B.2 Basement Vents Detailed 419
B.3 Basement HVAC Plan 420
B.4 Basement HVAC Isometric 420
B.5 First Floor Plan 421
B.6 First Floor Vents 422
B.7 First Floor HVAC Plan 423
B.8 First Floor HVAC Isometric 423

List of Abbreviations

A/C	Air conditioning
ACPH	Air changes per hour
AFG	Assistance to Firefighters Grant
BTU	British Thermal Unit
CFD	Computational Fluid Dynamics
DelCo	Delaware County Emergency Services Training Center
FDS	Fire Dynamics Simulator
FSRI	UL Firefighter Safety Research Institute
HVAC	Heating, Ventilation and Air Conditioning
NFPA	National Fire Protection Association
NIST	National Institute Standards and Technology
OSB	Oriented Strand Board
UL	Underwriters Laboratories

Chapter 1: Introduction

Prior research has shown that ventilation conditions within a structure play an important role in fire development and spread [1, 2]. One aspect of ventilation that has not been studied under fire conditions is the effect that residential heating, ventilation and air conditioning (HVAC) may have on a fire.

Firefighters have become aware of the need to check HVAC units in the event of a commercial fire. It is common for a truck company to be sent to the roof of a structure to check the HVAC units for smoke and heat. Firefighters are aware that products of combustion can be transported through an HVAC system, but the extent and importance of particle movement through an HVAC system has yet to be determined. The movement of smoke and fire gases in a structure is important for firefighters to help guide their search for occupants. According to Stephen Marsar's article 'Survivability Profiling: Are the Victims Savable? [3]', firefighters are dying at a disproportionately high rate compared to civilians at incidents where firefighters are killed. Marsar stresses the importance of reading the conditions inside a structure and understand how the fire will progress before committing to interior operations such as search and rescue. Recognizing when occupant survival chances have disappeared should alter how fireground operations

and tactics are prioritized.

1.1 Experimentation

A one-story ranch style structure with a basement previously utilized for the study of basement fires was retrofitted with an HVAC system similar to that installed in a typical house. Experiments were conducted to see how an HVAC system would affect the spread of combustion products through a one-story ranch structure on a basement. The fire source for these experiments was provided by a single gas burner controlled by a high-precision turn valve. The gas flow for these experiments were controlled with a mass flow controller, designed for propane flow. A variety of measurements were collected during these experiments including gas concentrations of oxygen, carbon monoxide, carbon dioxide and water vapors. Data on the gas velocity, temperature and pressures within the structure were also recorded. The gas burner experiments referenced in this report are a subset of a larger project. The test names used in this report are the same as the test names utilized in the overarching project. The primary experiments of interest are Experiments 1, 8, 15, 16, 23, & 25.

1.2 HVAC Basics

HVAC in the residential structure has been present since the middle of the 20th century [4]. The role of HVAC systems in the movement of smoke and gas has not been studied in depth. Similarly, no validation work has been performed

to check the performance of the HVAC sub-model in FDS [5]. Having experiments focused specifically on HVAC will allow for a better understanding of how products of combustion disperse through a structure. A better understanding of smoke and gas movement in residential HVAC systems can additionally highlight areas of concern for both firefighters and HVAC designers.

A basic understanding of the components in an HVAC system and how it functions is necessary to understand how the model should perform. Airflow through a system is generated by a fan housed within the furnace in our structure. The furnace filters and conditions the air prior to distribution through the structure via the duct system. Ductwork is split into two separate systems. A supply system distributes the air from the furnace throughout the conditioned space of the structure. The return system pulls air from the rooms in the structure and returns it to the furnace to be filtered and conditioned [6].

Ductwork is typically made of galvanized steel or similar materials formed in rectangular or circular sections. Ductwork can be run above or below a ceiling and through unconditioned spaces when wrapped in insulation. A main duct trunk can split off into separate branches at junctions called tees. When air enters or exits a room it will pass through a vent that can be mounted on a wall, ceiling or floor. A vent is located at the end of a duct branch and typically has vanes to slow and direct the airflow into the room. Typically, duct size will decrease in a branch as you move further from the furnace. The furnace function is controlled by a thermostat that is centrally located. The thermostat can be set to turn the furnace on or off depending on temperature settings and can determine when heat needs to be added or removed

from the conditioned air to reach a desired temperature at the thermostat's location. A condensing unit is needed to facilitate the heating and cooling of air within the furnace. The condensing unit circulates a refrigerant coolant through tubing and coils inside the furnace and within the condenser unit on the exterior of the structure to allow heat transfer from the coolant to the exterior air [6].

1.3 Computational Modeling

Modeling of the experiments was performed using a Fire Dynamics Simulator (FDS)(version 6.7.3) and Smokeview (version 6.7.10) was used for visual representation of the results. The simulations were completed by the computational fluid dynamics (CFD) coding within FDS that models thermally-driven fluid flows. The software packages are maintained by the National Institute of Standards and Technology (NIST). For low speed thermally-driven flows (Ma¡0.3), FDS numerically solves a form of the Navier-Stokes equations. A main point of emphasis FDS is how smoke and heat produced by a fire are transported around the model. A complete description of the modeling capabilities of FDS, including equations and numerical algerithms utilized, can be found in the Technical Reference Guide [7]. Information on how to construct an input file for FDS can be referenced in the FDS User's Guide [8].

Mathematical verification of the equations utilized within FDS can be found in the Verification Guide [9]. Validation of FDS [5] is continually being performed with comparisons to a ever growing database stocked with data from various fire scenario

experiments. Verification of FDS is achieved by ensuring the equations used within FDS are being solved correctly. Validation, as defined by the developers of FDS, is the process of checking and ensuring the governing equations of the mathematical models are appropriately being applied. To achieve this, comparisons are typically made between experimental data and model results.

It is important to differentiate between validation and verification. Validation is used to relate FDS capabilities to real world situations. Verification confirms that the calculation process within the FDS program is being applied appropriately. Validation of the HVAC coding in FDS is important for several reasons. If simulations and experiments can yield similar results, then further studies can be conducted without the need for experiments as often. Fewer experiments means less cost when researching different fire scenarios. These costs include time, labor, material and structural damage as a result of the increased heat and smoke. Simulations provide more versatility in a shorter time frame. Multiple fire scenarios can be simulated and building layouts can be adjusted as desired in a short amount of time. By modeling the propane gas fire experiments in FDS, analysis can be performed to compare the outputs of the model to the data collected in the experiments. Results of this comparison can hopefully then be used to validate the HVAC coding in FDS as it is applied to a one-story structure on a basement. Prior validation work has compared FDS simulations to real world incidents [10–15].

CFD modeling is a tool commonly utilized by fire protection engineers in various diciplines. The ability to predict fire dynamics and smoke behavior allows an engineer to better prepare safety designs for a structure. It is important that

CFD models are continually validated against different fire scenarios to ensure that the models being created are applicable. FDS is a commonly used program for these models. Within the FDS Validation Guide are several prior studies that look at smoke and gas flow over multiple stories, through doorways and through HVAC vents. While the list experiments within the FDS Validation Guide continues to grow, there have not been any experiments published that investigate the effect of a full scale HVAC system in a multi-level structure. Successful completion of these gas burner experiments and FDS modeling for comparison will provide a valuable database that can be contributed to the FDS validation space.

It is addressed in the FDS Technical Refenence Guide that FDS itself is not able to adequately model an HVAC network. To overcome this limitation, an HVAC submodel is included within FDS. The HVAC submodel works as a linkage between the FDS field model and the HVAC network model. A network model for the HVAC submodel implies that uniform parameters are present in any cross sectional view of the ductwork. This imbedded assumption is appropriate for HVAC because at any discrete point along the ductwork, a uniform field would be expected. Ralph and Carvel ([16], 2018) provide a detailed review of various coupled hybrid modelling approaches. From the Technical Reference Guide, it is stated that the HVAC solver is based on a computer program called MELCOR, which is utilized for simulating accidents within nuclear power plant containment buildings. The MELCOR program [17] is a thermal hydraulic solver which models the spread of fire and smoke in complex ventilation systems. The MELCOR program was utilized in FDS with implimentation similar to the implementation of MELCOR in the Fire and Smoke

SIMulator ((FSSIM) [18]).

1.4 Objectives and Overview

- Establish experimental baseline for heat and gas movement through a residential HVAC system

  Observe how changes to fire size, location, and HVAC system compare to baseline

- Model experiments in FDS

  Compare variation of FDS resolution

  Contribute data and code to validation space

  Emphasize the contributions to the HVAC submodel

  Highlight and compare water vapor measurements

As stated in the list above, there are several objectives that these experiments hope to achieve. Establishing a baseline from the experiments for how the heat and gases produced by the fire spread through the structure is a key objective. The data for how heat and gases travel through the HVAC within the structure will enable comparisons to be made when changes are made. The changes for comparison include varying the HRR, fire location, and status of the HVAC system. After the collection of data from experiments, the next objective is to recreate the experimental fires within the constraints of FDS. Successful simulations will enable a comparison of experimental fires to simulated fires. Should the model data agree

with the experimental data, a case can be made to use the findings to strengthen the validation of FDS and the HVAC submodel.

A baseline knowledge of how gases move through the structure will also enable further research into where an occupant has the best chances for surviving inside a structure. Being able to apply the data found within these experiments to victim survivability will hopefully draw a map of tenable spaces within a structure. Further research on this matter should include the use of real fuels and firefighting tactics as well as an analysis of how fire suppression affects gas flows.

This thesis contains detailed descriptions of the setup of the experiments including the construction of the test structure (see Chapter 2 and Appendix B), the HVAC within the structure (Subsection 2.1.1 and Appendix B), and the instrumentation that accompanied the experiments (Section 2.2). The testing procedures for the experiments are also detailed with descriptions of the simulated fire characteristics as well as the HVAC control statuses that accompanied the different experiments (Chapter 3). The information provided in the experimental setup and procedures were then incorporated into FDS input scripts which were utilized to simulate the experimental burns (Chapter 4). The output results were then compared to the data collected during the experiments. Analysis of the experiments and models examined several factors including: hot gas travel through the HVAC system; gas concentrations of oxygen, carbon monoxide, carbon dioxide and water vapors; gas velocity; and pressures within the structure (Chapter 5 and Chapter 6).

Chapter 2: Experimental Setup

2.1 Experimental Structure

Gas burner experiments were conducted in a structure located at the Delaware County Emergency Services Training Center in Sharon Hill, PA. The structure is a ranch-style house with an above ground basement. The outer wall of the basement was composed of interlocking concrete blocks 0.6 m wide, 0.6 m high, and 1.2 m long. The concrete blocks act as insulation to simulate the basement being below ground level. The interior dimensions of the basement were 13.3 m wide, 7.28 m long, and 2.74 m high. The joints and gaps between the blocks were filled with high-temperature insulation. The walls were constructed from dimensional lumber, 3.81 cm by 8.89 cm. The studs were lined with 1.27 cm drywall. The ceiling had a single layer of 1.27 cm drywall over engineered lumber I-joists. The exterior walls of the first floor were protected by 8 mm thick fiber cement board siding, a layer of olefin home wrap, and 1.27 cm oriented strand board (OSB). The walls were constructed from nominal dimension lumber, 3.81 cm by 8.89 cm . The studs were lined with 1.27 cm drywall. The interior dimensions of the first floor measured 13.77 m by 7.68 m with a 2.44 m ceiling. For the duration of this report, the two floors in the structure will be referred to as the basement and the first floor. The sides of the

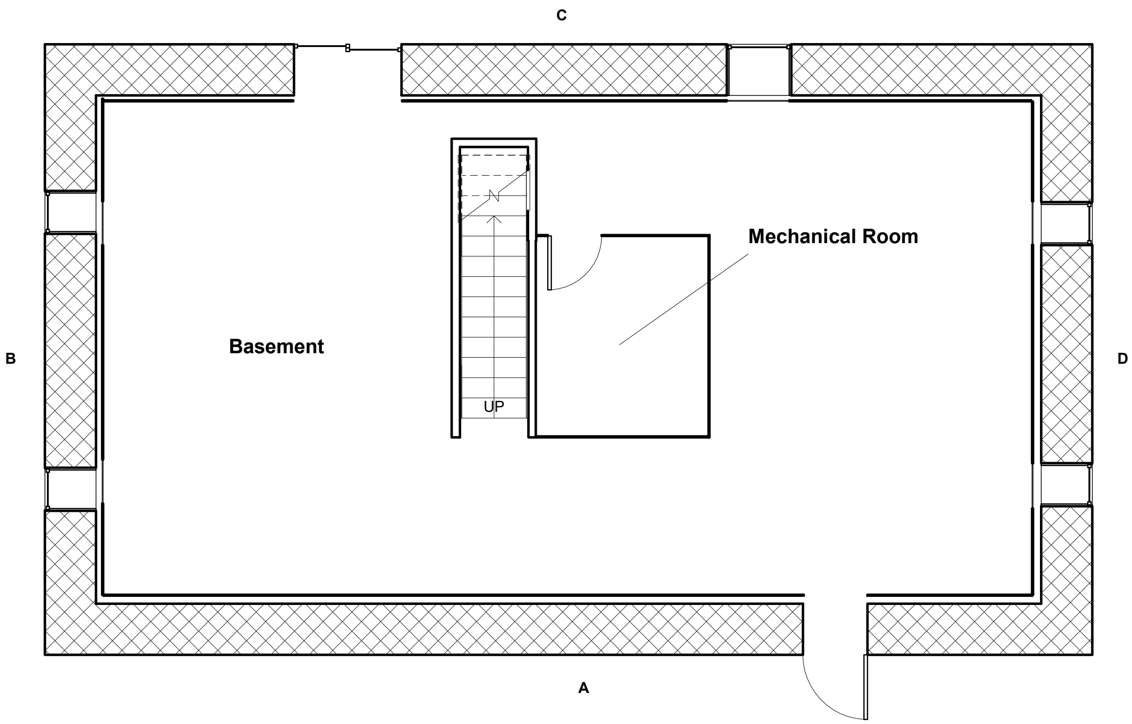

Figure 2.1: Basement Plan

structure were assigned a letter designation starting with the front door side being referred to as the A side and continuing around the structure clockwise there are the B, C and D sides. Figure 2.1 shows the basement floorplan and Figure 2.2 shows the first floor plan. Both plans show the letter designation that refers to each side of the structure. Further detailed drawings of the structure are included in Appendix B.

A leakage test was performed with all exterior vents closed to determine the relative tightness of the structure. To calculate the leakage from the structure ASTM E779 was followed [19]. Based on the results of this test, the leakage in the structure was determined to be 8.3 air changes per hour (ACPH) at 50 Pa. The equivalent leakage area was calculated to be 0.137 m^2 at 10 Pa. Equivalent leakage area is defined as the area of a sharp-edged hole that would have the same leakage flow

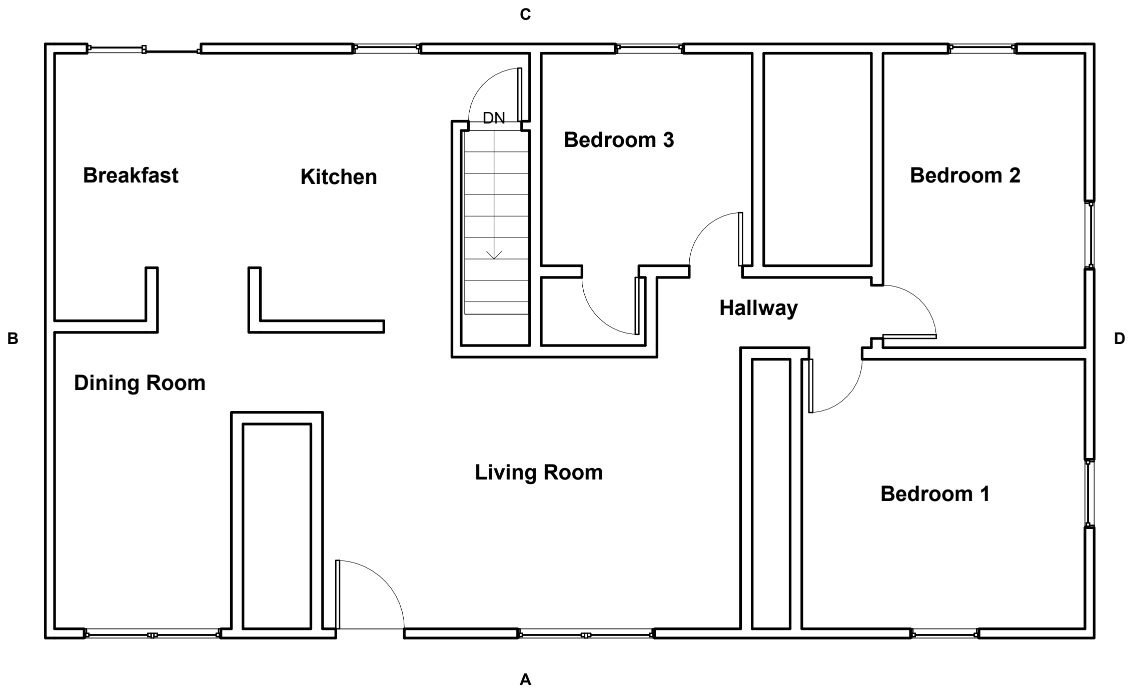

Figure 2.2: First Floor Plan

rate as the building if both were subjected to a 10 Pa pressure difference. For a single story residential structure, a tight house would have 3.5 ACPH50 (air changes per hour at 50 Pa), a moderately tight house would have 8.8 ACPH50, a typical house would have 17.5 ACPH50, and a leaky house would have 35 ACPH50 [20]. Based on the test performed, this structure would be classified as a moderately tight structure.

2.1.1 HVAC

A fully functioning residential HVAC system was installed in the structure. The HVAC system was constructed to replicate a system that would typically be found in a house in the testing region. A local contractor was utilized to install the system and to ensure that every aspect of the HVAC system was within all

codes and standards for the region. The system installed had a 18 kW heater, with standard efficiency and a heating capacity of 36,000 British Thermal Units per hour (BTU/H). The system had a 3 ton capacity with a 25.4 cm x 20.3 cm 1/2 horsepower 5 speed motor. The motor is 44.5 cm wide which allows for a capacity of approximately 17.0 m^3/min to 34.0 m^3/min. R410A refrigerant was used as the cooling fluid that conditions the air in a single stage air handler [21]. The system installed did not have an exterior air return for drawing fresh air in from outside the structure.

Galvanized steel ductwork was run through the house to create the main trunk of airflow. The HVAC system was split into two, a supply and a return. The supply system originated in the basement mechanical room and extended out through the top of the furnace unit. The system then split into three separate ducts, two that fed both sides of the basement and one that fed up to the first floor. The ducts that supplied the basement branches measured 35.6 cm x 20.3 cm. There were eight supply vents in the basement, each being fed by a circular duct with a diameter of 17.8 cm branching off of the main supply ducts. With the exception of supply vents S-06 and S-07 in the C/D corner, which measured 27.9 cm x 27.9 cm, the basement supply vents measured 23.8 cm 23.8 cm. The supply vents were labeled based on location in the basement. The basement was split into a B side and a D side for the purposes of labeling vents. S-01 through S-04 were located in the B side of the basement. S-01 was in the A/B corner of the B side, S-02 in the B/C corner of the B side, S-03 in the C/D corner of the B side and S-04 in the A/D corner of the B side. S-05 through S-08 were located in the D side of the basement. S-05 was in the

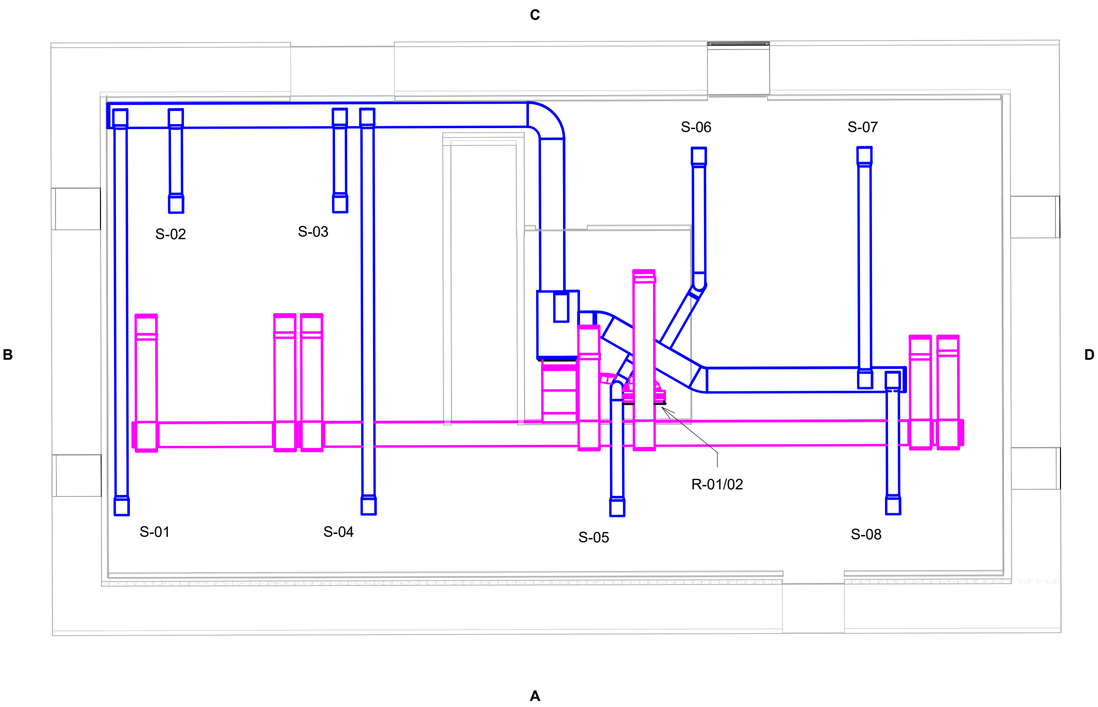

Figure 2.3: Basement HVAC

A/B corner of the D side, S-06 in the B/C corner of the D side, S-07 in the C/D corner of the D side and S-08 in the A/D corner of the D side. Labeled basement vents are provided in Figure 2.3.

The first floor of the structure also has eight supply vents split evenly between the B side and D side. The single duct that ran from the basement to the first floor measured 40.6 cm x 20.3 cm. The single duct split into two ducts in the attic space measuring 30.5 cm x 20.3 cm each that fed both sides of the structure. The branches that supplied the first floor vents were circular with a radius of 15.2 cm. The supply vents on the first floor all measured 30.5 cm x 15.2 cm. S-09 through S-12 were located on the B side and S-13 through S-16 were on the D side. S-09 supplied air to the dining room, S-10 to the kitchen by the sliding door, S-11 to the living room by the front door and S-12 to the kitchen by the top of the basement

staircase. S-13 was in bedroom 3, S-14 in the living room toward the D side, S-15 in bedroom 1 and S-16 in bedroom 2.

The return system started at every return vent and terminated in the bottom of the furnace in the basement mechanical room. There were a total of seven return vents in the structure with two of the vents being considered ‘double returns’ because they comprised two joist spaces. Each return vent fed into a plenum space consisting of either a floor joist space or a wall stud space. The plenum spaces then fed into the return ductwork that measured 35.6 cm x 20.3 cm. Two branches of the return ductwork combined all of the return air from the entire structure then fed into the furnace through a single duct that measured 50.8 cm x 25.4 cm. The floor joist plenum space measured 30.5 cm wide, 35.6 cm deep and 1.83 m long. The double return in the basement fed into two wall stud spaces each measuring 36.8 cm wide, 7.6 cm deep and 1.83 m tall. The return vent in the basement measured 66.0 cm x 25.4 cm and was labeled R-01/02. This vent was located on the exterior of the A side mechanical room wall. All single return vents on the first floor measured 35.6 cm x 25.4 cm while the double return on the first floor measured 66.0 cm x 25.4 cm. R-03 was a single return in the dining room C side wall. R-04/05 was a double return in the short hallway between the dining room and the living room on the C side wall. R-06 was a single return located in the living room on the C side wall toward the D side. R-07 was a single return in bedroom three on the A side wall. R-08 was a single return in bedroom one on the C side wall. R-09 was a single return in bedroom 2 on the A side wall. Labeled first floor vents are provided in Figure 2.4.

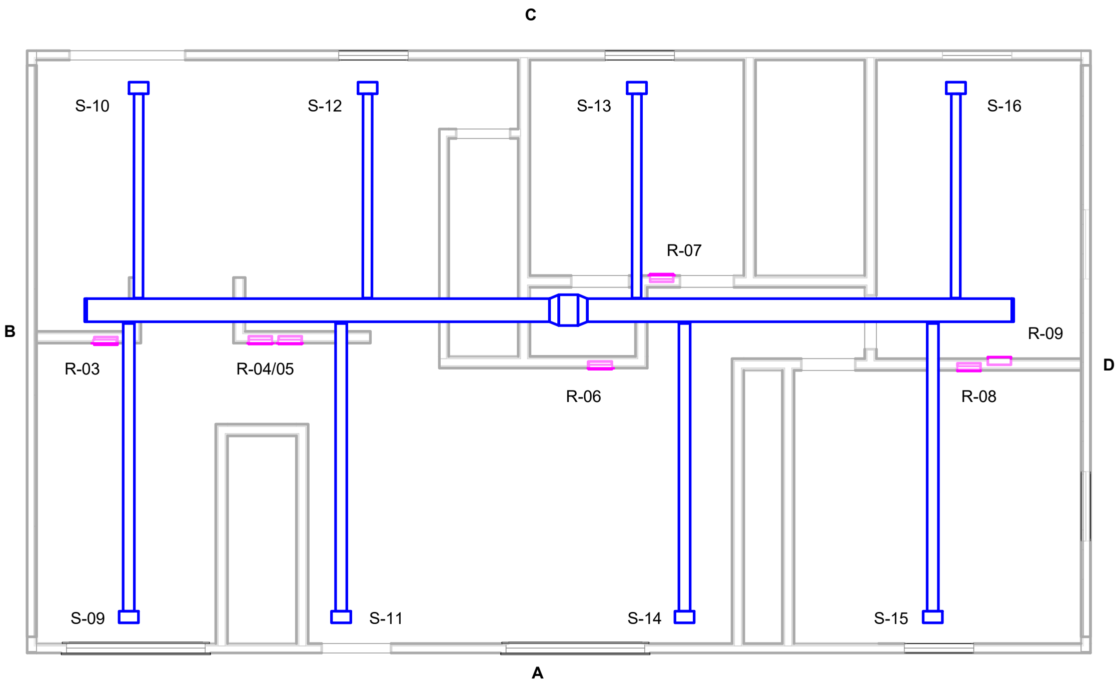

Figure 2.4: First Floor HVAC

The condensing unit for the HVAC system was located along the exterior D side wall of the structure. The condensing unit used was a heat pump type model utilizing R410A refrigerant, a single stage motor and a capacity of 36,000 BTU [22].

Prior to the start of experiments, a flow hood was utilized to capture the flow rates out of each supply vent and in through each return vent. This collected data was then utilized in FDS to determine losses in the HVAC ductwork. For further information on the usage of the collected flow rates see Appendix A for cold flow modeling.

2.2 Instrumentation

The test structure was outfitted with instrumentation for gas temperature, gas velocity, gas concentration and pressure measurements. Gas temperatures were

measured with 1.3 mm. bare-bead, chromel-alumel (type K) thermocouples and 1.6 mm inconel-sheathed thermocouples. Small-diameter thermocouples were used during these experiments to limit the impact of radiative heating and cooling. The expanded uncertainty associated with the temperature measurements from these experiments is estimated to be ± 15% as reported by researchers at NIST [23, 24].

Sheathed thermocouples were used with bi-directional probes and were used for measuring gas velocity. The pressure measurements were made using differential pressure sensors, with an operating range of ±125 Pa. The pressure sensors were connected to each side of the bi-directional probe. From prior research investigating gas velocity flow through doorways in pre-flashover compartment fires, an expanded uncertainties range from ±14% to ±22% was established for measurements from bi-directional probes, similar to those used during these experiments [25].

Gas concentration measurements collected include oxygen, carbon dioxide, and carbon monoxide. To measure these concentrations, sampling ports were installed in various locations in the structure. A sampling port consisted of 9.5 mm stainless steel tubing within the structure, with an open end in the room of interest. The steel tubing transported gases to an area outside of the heated environment. Once outside the structure, the sample was filtered through a coarse, 2 micron paper filter before being drawn through a condensing trap to remove moisture. At the condensate trap exit, the sample line transitioned from stainless steel to polyethylene tubing for flexibility. Upstream of the analyzer, the sample passed through a fine, 1 micron filter. To minimize transport time through the system, samples were pulled from the structure with the use of a vacuum/pressure diaphragm pump rated

at 0.75 CFM. Gas samples were analyzed through oxygen analyzers (paramagnetic alternating pressure) and combination carbon monoxide/carbon dioxide analyzers (non-dispersive infrared). The gas sampling instruments used throughout the series of tests discussed in this report have demonstrated an expanded uncertainty of ±1% when compared to span gas volume fractions [26]. Given the non-uniformities and movement of the fire gas environment and the limited set of sampling points in these experiments, an estimated expanded uncertainty of ±12% is applied to the results [27]. Each sampling port was installed 1.2 m above the respective floor in the room of interest.

Pressure measurements were made using differential pressure sensors, effectively determining pressure rise or drop relative to ambient (external) conditions. The pressure taps were made up of 6.35 mm. copper tubing secured into a wall. One side of the tubing was open to the room, approximately 15 cm away from the wall. The other side was connected to a differential transducer. The range of the differential pressure sensors is ± 250 Pa and the uncertainty is estimated at ± 10% [28].

Detailed drawings and further information regarding instrument channel listings can be found in Appendix B.

2.2.1 Basement

A legend for the instrumentation used in the basement and a basement instrumentation plan can be seen in Figure 2.5. Five thermocouple arrays, also referred

to as trees (TC tree), were installed in the basement of the test structure. Each TC tree in the basement consisted of nine thermocouples starting approximately 2.54 cm below the ceiling and were spaced approximately 30.5 cm apart descending down toward the floor with the last thermocouple being approximately 2.44 m below the ceiling. The heights for all basement instrumentation are noted in the legend in Figure 2.5. The basement was subdivided into four corners, or quadrants, and a TC tree was placed in the middle of each corner. One TC tree was also placed in the mechanical room.

Two locations were used to collect data via pressure taps, one for the B side and one for the D side of the basement. Each location had three pressure taps, the first at a height of 0.3 m, the second at 1.22 m and the third at 2.13 m. The taps extended from the wall by approximately 15 cm. A gas concentration measurement was taken at an elevation of 1.22 m. The gas concentration measurement was located by 3TC in the C/D corner for all first floor burner experiments and was then moved to an alternate location next to 2TC in the B/C corner for the basement burner experiments. The alternate location for gas concentration measurements is denoted in Figure 2.5 as '1GAS*'.

The combined bi-directional probe and thermocouple arrays were located near the base of the staircase in the center of the stairwell. Six velocity and temperature probes were used in this array with their heights noted in the legend.

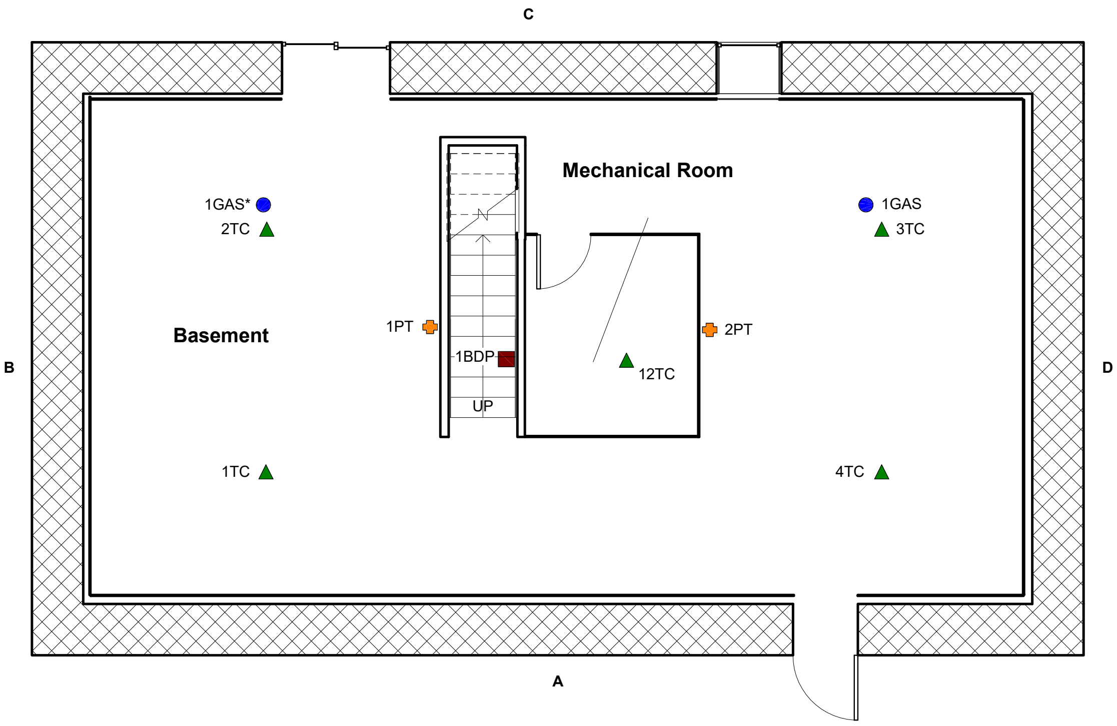

Symbol	Description
▲	TC Tree (0.3m-2.72m)
●	GAS Sensor (1.22m)
■	Bi-Directional Probe and Thermocouple Array (0.99m, 1.27m, 1.55m, 1.88m, 2.18m, 2.49m)
✚	Pressure Taps (0.3m, 1.22m, 2.13m)

Figure 2.5: Basement Instrumentation

2.2.2 First Floor

A legend for instrumentation used in the first floor and a first floor instrumentation plan is shown in Figure 2.6. Six thermocouple arrays were installed on the first floor of the structure. Each thermocouple tree on the first floor consisted of eight thermocouples starting approximately 2.54 cm below the ceiling and were spaced approximately 30.5 cm apart descending down toward the floor with the last thermocouple being approximately 2.13 m below the ceiling. The thermocouple trees were installed centrally in the room that they were installed in.

Six pressure taps were installed, as shown in Figure 2.6, at the same heights as those in the basement, 0.3 m, 1.22 m and 2.13 m. The taps extended from the wall by approximately 15 cm. Five gas concentration measurement locations were installed. During experiments, not all gas concentration measurement locations were utilized due to a limited number of gas channels available. A combination of three of the first floor gas concentration measurement locations were used along with the single gas concentration location in the basement. First floor locations were chosen based on burner location and interior door positions. Gas concentration measurements were taken at an elevation of 1.22 m.

Two locations were used for the combined bi-directional probe and thermocouple arrays. One array was located at the front door on the exterior side of the door to measure flows in and out of the front door when open. The second array was located at the top of the stairwell above the top step before reaching the first floor. Each array consisted of five measurement probes, located at the heights listed

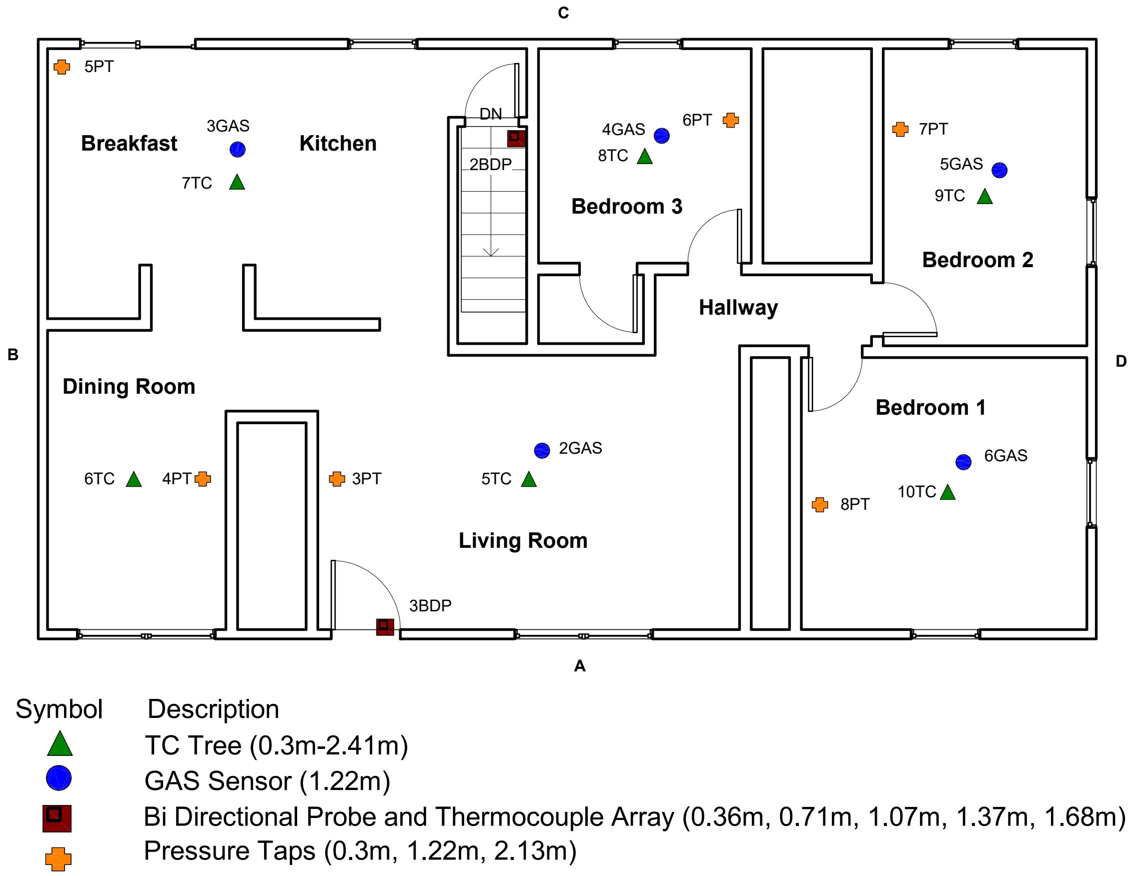

Figure 2.6: First Floor Instrumentation

above the first floor level.

2.3 Gas Burner Setup

For the gas burner experiments, two large propane tanks were utilized. The tanks were set up at a location far from the structure and a series of flexible pipes were used to route the propane to the burner. To control the flow of propane into the structure, several control valves were installed between the propane tanks and the gas burner. The first control valve that the propane passed through was the control valve at the propane tanks which opened and closed the tanks. Propane

traveled from the tanks to the exterior of the structure through a 4.5 cm flexible metal pipe to a quarter turn valve which acted as the emergency shutoff valve for the experiments. Propane then flowed through a 5 cm steel braided flexible pipe section to the next control valve, the electronic flow controller. The electronic flow controller determined the quantity of propane that was allowed to pass through based on a voltage set at the control device. Propane flowed from the flow control device through another section of braided steel pipe to a needle valve. The needle valve was used to slowly allow gas to enter the structure during ignition to prevent a large buildup of propane inside the structure. The needle valve helped to ensure a smooth ignition that did not result in a large pressure increase inside the structure due to rapid ignition of excess propane. After the needle valve the propane traveled through more sections of braided steel piping and 90 ° metal elbows before arriving at the burner.

A pilot line was installed to run from the exterior of the structure to the burner and was fuelled by a small portable propane bottle. The pilot line was constructed with a quarter inch diameter copper tube that ran from the portable propane bottle to the top of the gas burner. Igniting the pilot line prior to opening the higher flow propane tanks allowed for a safe ignition of the larger gas burner that did not require anyone to be inside the structure.

The location of the gas burner for the basement experiments and first floor experiments can be found in Figure 2.7 along with a legend for the figures. A strict sequence of events was created to ensure safe operation of the gas burner. For more information see Chapter 3.

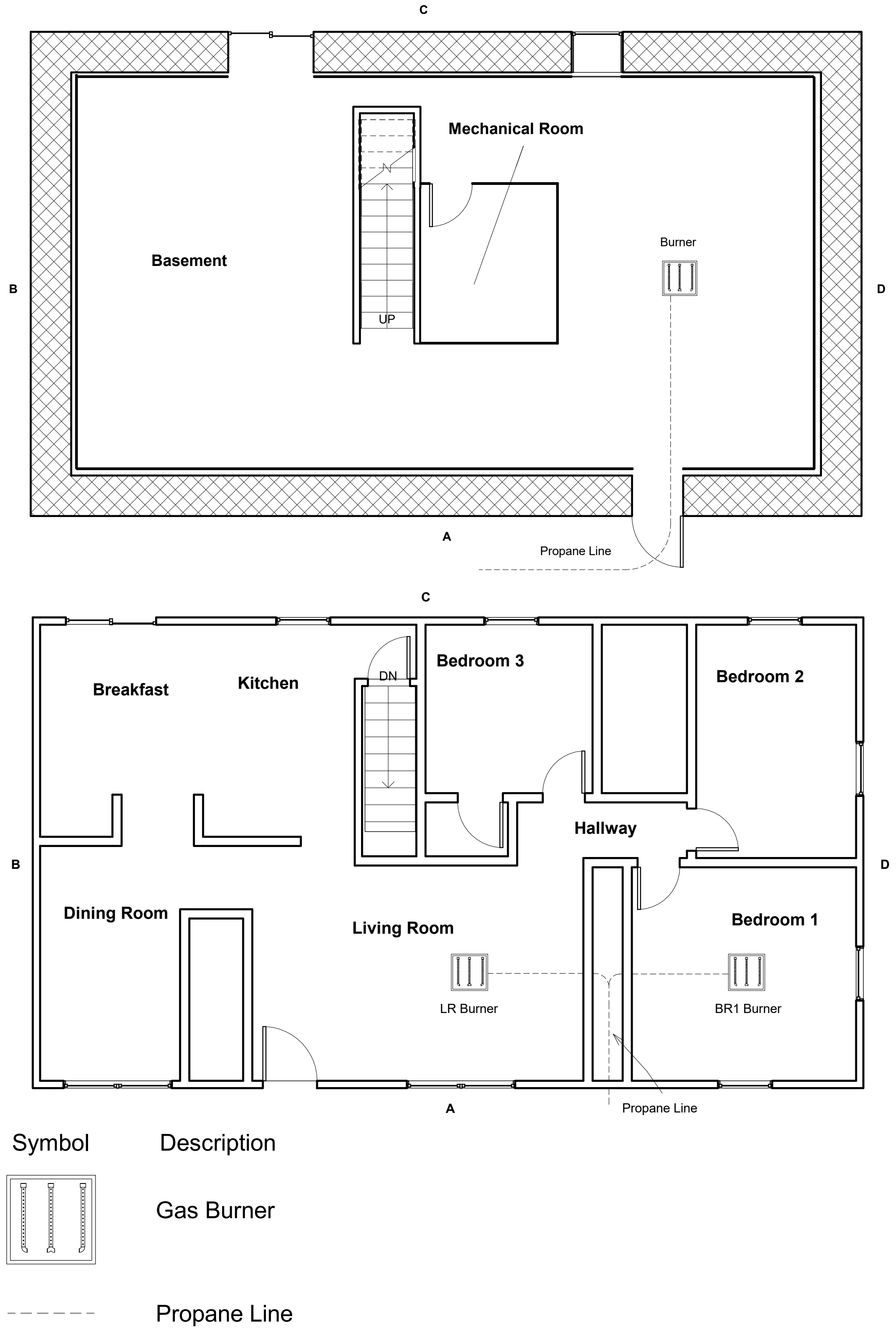

Figure 2.7: Top: Basement Gas Burner, Middle: First Floor Gas Burner Locations, Bottom: Gas Burner Legend

2.4 Experimental Scenarios

Three fire locations were utilized for the gas burner experiments. The first fire location was bedroom 1. A list of experiments for bedroom 1 can be seen in Table 2.1. Bedroom 1 was selected to simulate a typical fire in a bedroom. With the exception of Experiment 7, a HRR of 121 kW was set for all bedroom 1 experiments. For Experiment 7 a HRR of 302 kW was used to see the effect of a larger fire in the bedroom. The status of the HVAC was varied between passive, on in A/C and on in heat. The bedroom door was also changed between open and closed to see the effect of ventilation on the spread of fire gases.

Table 2.1: List of Bedroom 1 Experiments Performed

Experiment	HRR (kW)	HVAC Status	Bedroom Door
1	121	Passive	Open
2	121	A/C	Open
3	121	A/C	Open
4	121	Heat to Off	Open
5	121	A/C	Open
6	121	Heat to Off	Open
7	302	Passive	Open
8	121	Passive	Closed
9	121	A/C	Closed
10	121	Passive	Closed
11	121	Passive	Closed
12	121	Passive	Open
13	121	A/C	Open
14	121	Off to A/C	Open

The second set of burner experiments took place in the living room. A list of experiments for the living room can be seen in Table 2.2. Living room experiments were performed to see how a fire in a larger room on the first floor would spread fire

gases throughout the structure. Three HRR were used in the living room, increasing from 121 kW to 302 kW, and finally up to 400 kW. HVAC status was set to either passive or A/C during the living room experiments. The HVAC heat setting was not used in the living room after analysis of the bedroom 1 heat experiments yielded no notable differences from the baseline. With the exception of Experiment 15, all living room experiments were performed with the door to the basement staircase closed.

Table 2.2: List of Living Room Experiments Performed

Experiment Number	HRR (kW)	HVAC Status
15	121	Passive
16	121	Passive
17	121	A/C
18	302	Passive
19	302	A/C
20	393	Passive
21	393	A/C

The third set of experiments were performed with the gas burner in the basement on the D side of the structure. A list of basement experiments can be seen in Table 2.3. Basement experiments were performed to see how a fire in a larger volume would spread fire gases through the structure. Basement fire scenarios also enabled observation of fires for longer at higher HRRs because the basement ceiling was further from the burner than on the first floor. The two HRR utilized in the basement were 302 kW 393 kW. Experiments were performed with the HVAC system in passive, A/C and heat. The basement staircase door for these experiments was varied between open and closed to see the effect that it had on fire gas spread to the first floor.

Table 2.3: List of Basement Experiments Performed

Experiment Number	HRR (kW)	HVAC Status	Basement Door
22	Scrubbed	Scrubbed	Scrubbed
23	393	Passive	Open
24	302	A/C	Open
25	302	Passive	Closed
26	302	A/C	Closed
27	302	Heat	Closed
28	393	Passive	Closed
29	393	Passive	Open

Chapter 3: Experimental Procedure

Once construction and instrumentation was complete, a testing procedure was established to reduce risks during live fire experiments and increase reliability of the experiments. Prior to starting an experiment, all data collecting equipment was evaluated for proper function and connection during what were called channel checks. Channel checks were completed using two different tools; a heat gun to check thermocouples, bi-directional probes and pressure taps, and a specific gas combination to determine the time delay in gas concentration measurements from the sampling point to the analyzer. Bullet cameras and infrared cameras were checked for good connections and appropriate placement. After ensuring all data and video collection sources were properly set up, a new filter was installed in the HVAC system. The HVAC system was then set according to the plan for that experiment. If the HVAC system was set in active cooling or heating then the system was given adequate time to condition the space to a temperature of approximately 70 $^\circ$ C to 90 $^\circ$ C prior to the start of background data collection.

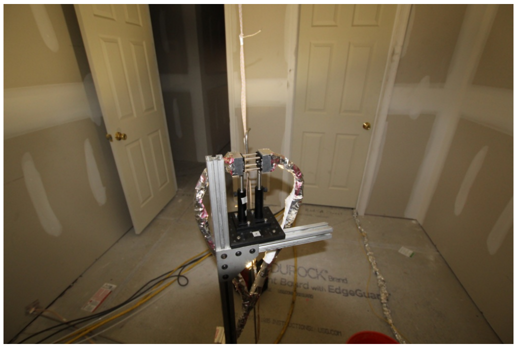

(a) Bedroom 3 Water Vapor

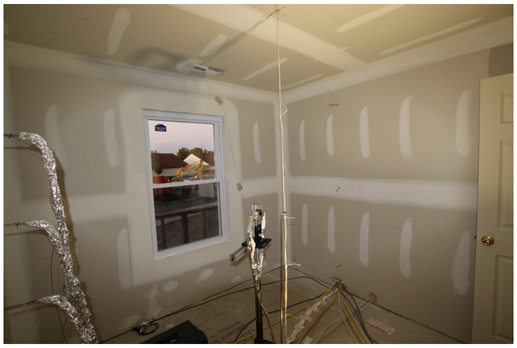

(b) Bedroom 3 Instrumentation

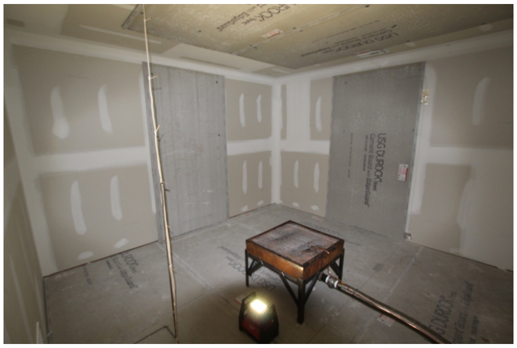

(c) Bedroom 1 Propane Burner and Thermocouple Tree

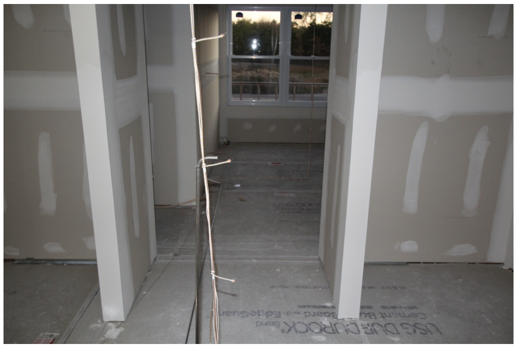

(d) Kitchen Gas and Thermocouple Tree

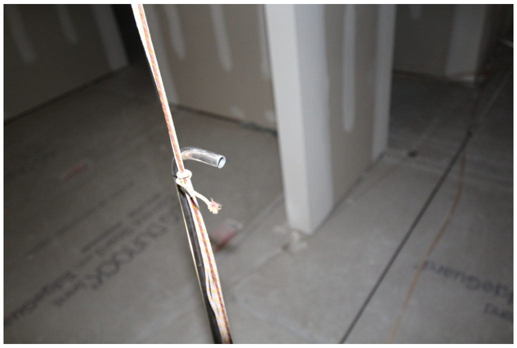

(e) Kitchen Gas Intake

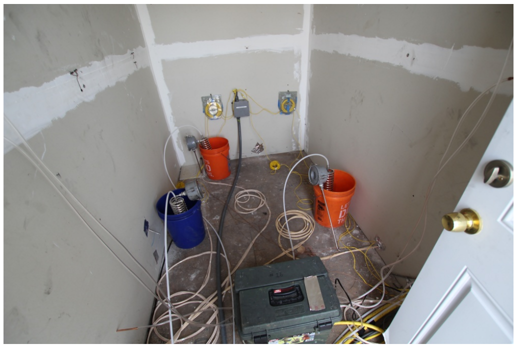

(f) Instrumentation Closet

Figure 3.1: Experiment Instrumentation

Prior to ignition, safety precautions had to be established. To control the flow of propane into the structure. Several control valves were installed between the

propane tanks and the gas burner. The first control valve that the propane passed through was the valve at the tanks which opened and closed the tanks. After the propane tanks were opened propane traveled down the gas line to a quarter turn ball valve which acted as the emergency shutoff valve for the experiments. One team member was always positioned at the emergency shutoff valve whenever the propane tanks were open. The next control valve that the propane flowed through was the electronic flow controller. The electronic flow controller determined the quantity of propane that passed through using a voltage controlled device. The last control valve between the electronic flow controller and the gas burner inside the structure was a needle valve that was used to slowly allow gas to enter the structure during ignition to prevent a large buildup of propane inside the structure. The needle valve helped to ensure a smooth ignition that did not result in a large pressure increase inside the structure.

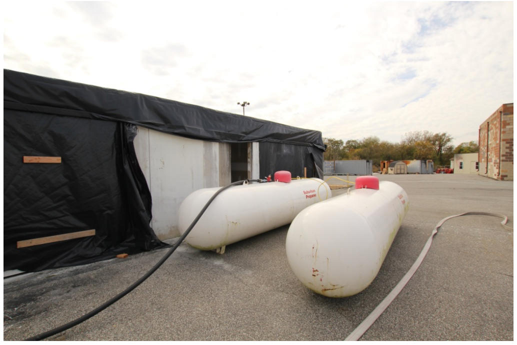

(a) Propane Tanks

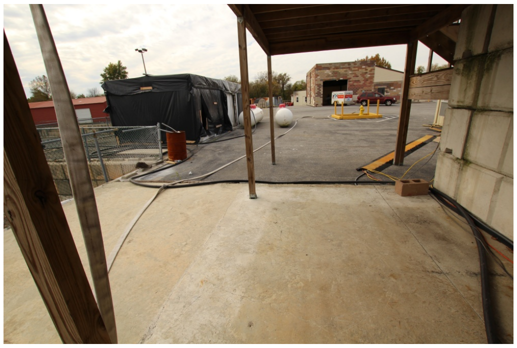

(b) Propane Path

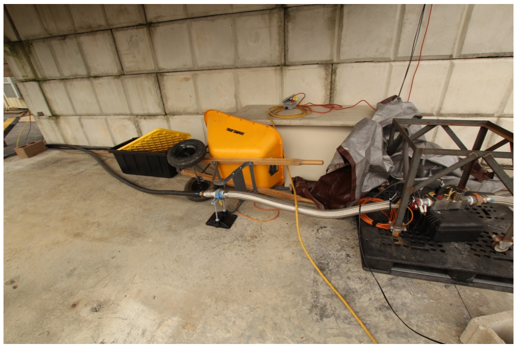

(c) Propane Flowmeter and Ball Valve

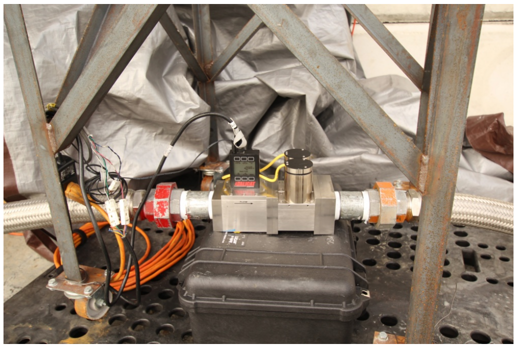

(d) Propane Flowmeter

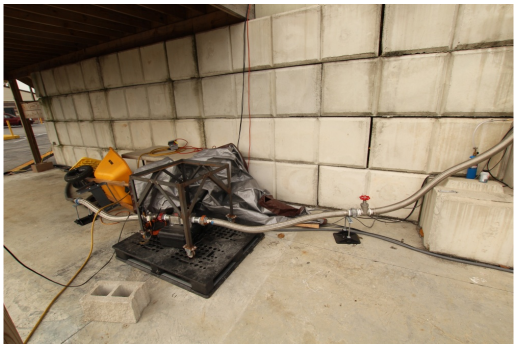

(e) Propane Flowmeter and Needle Valve

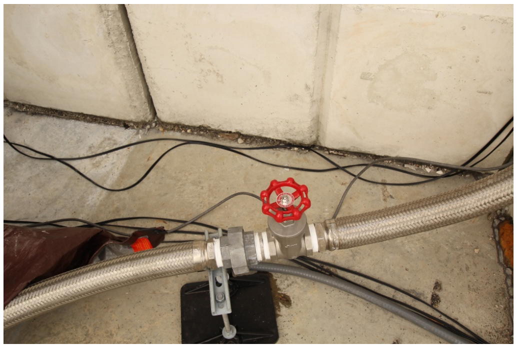

(f) Needle Valve

Figure 3.2: Propane Path and Safety Features

A pilot line was run from the exterior of the structure to the burner and was fueled by a small portable propane bottle. After background data collection had been occurring for a few minutes the fire suppression firefighter in full turnout gear

and SCBA would enter the structure and ensure that the structure was ready for the experiment. This included opening and closing doors as planned and checking the thermostat for proper function. Once the structure was deemed ready to test, the pilot line gas would be opened outside of the structure and the fire suppression firefighter would ignite the discharged propane at the burner using a lighter. After confirmation of the pilot gas ignition the fire suppression firefighter would confirm that no one was inside the structure and exit the structure. The fire suppression firefighter then positioned at the front door with a safety hose line in the event that the fire spread from the propane burner and had to be extinguished.

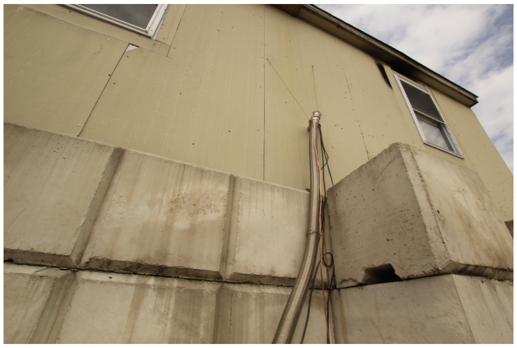

(a) Propane Pilot Line

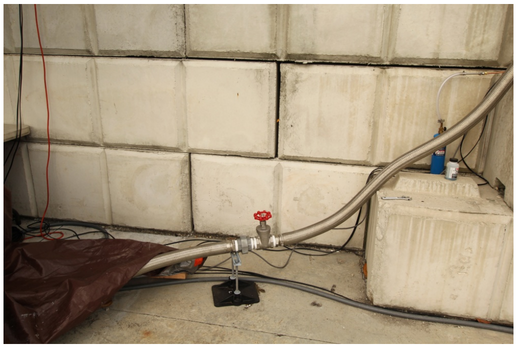

(b) Propane Pilot Tank

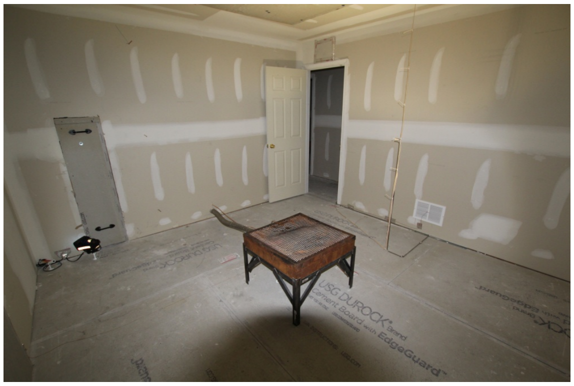

(c) Bedroom 1 Propane Burner

Figure 3.3: Propane Pilot Line

One team member oversaw each experiment from the data collection hub and had control of the electronic flow meter from that hub. This team member was referred to as command. Once the pilot light was ignited, command instructed that the propane tanks be opened. Gas then flowed up to the quarter turn valve. The flow meter voltage was then set to a predetermined voltage that matched the propane flowrate that was desired for the experiment. On command's directive the quarter turn valve was opened followed by the needle valve. Command then confirmed the ignition of the propane burner by cameras and data sensors. Once ignition was confirmed the pilot line was shut off. If the experiment being performed had a planned increase or decrease in the heat release rate, the electronic flow meter would be adjusted by command at a predetermined time and the mass flow rate would then be confirmed by a team member at the flow meter.

The experiments would then be run with command watching the gas concentrations within the fire room to prevent the experiment from reaching a ventilation limited state. The experiments would be terminated once a desired experiment length had been reached or the oxygen concentrations approached a ventilation limited state. On command's direction, the propane tanks would be the first thing to be closed. Closing at the propane tanks allowed for any gas in the line to be expelled relieving pressure on the gas line. Once the flow control meter had a no flow reading the quarter turn valve was closed. Around this time, command would note that all visible flames at the gas burner were extinguished by looking at the IR camera feed. The electronic flow control was then turned off and the needle valve was closed once the pressure in the gas line had decreased.

A few minutes after the termination of propane flow, the fire suppression firefighter would enter the structure at command's direction through the front door wearing full PPE and SCBA. The firefighter would close the front door after entering and would proceed to the fire room to confirm no active fire was in the structure. Ventilation would then occur in the structure at the direction of command. The firefighter would open doors and windows to allow heat and gases to escape the structure. After ventilation was complete data would continue to be collected until command determined that the experiment was complete. Upon completion of the experiment the HVAC filter was removed and sealed in a bag for later analysis. The structure was then given appropriate time to cool down to nominal temperatures prior to the start of another experiment. When multiple experiments were performed on the same day, channel checks were only performed prior to the start of the first experiment of the day.

Chapter 4: Modeling Setup

As stated in the Introduction [1], modeling for this project was performed using a Fire Dynamics Simulator (FDS)(version 6.7.4) program and Smokeview (version 6.7.14) for visual representation created and maintained by the National Institute of Standards and Technology (NIST).

The FDS software utilized performed the heat transfer and fluid flow calculations. There were several user defined fields that needed to be completed prior to the initiation of a simulation. Among the most important of the inputs was the mesh field, or grid spacing, that defined the space that would be utilized. A mesh field is a specified number of grid points in an X, Y and Z coordinate system that generated a three dimensional space. These grid points formed the basis for the construction of every element within the model. Defining a practical grid space was critical to the model performance. If the grid spacing was too large, then calculations would not be as accurate as desired. If the spacing was too small, then simulations would require extensive amounts of time and processing power to complete a simulation. Depending on the size of a model domain, several mesh fields could be established. These fields could be stacked to simulate multiple floors or could be established next to another field to have finer resolution of one level. Calculations were able to be

performed incorporating data from grid points within different mesh fields allowing for large spaces to be modeled accurately. Multiple mesh fields were established to improve processing of a model on a server. A computer with multiple processors could model multiple mesh fields simultaneously to reduce the time it took to complete a simulation [7, 8].

For our modeling purposes, mesh fields were established to complete simulations using either a grid spacing of 10 cm or 20 cm in the X, Y & Z coordinates. The 20 cm grid was used during initial modeling runs to check for errors. The domain for these models simulated a space that was 16.2 meters long, 10.2 meters wide and 8 meters tall, fully containing our structure. When simulations were completed in the 20 cm grid spacing, two mesh fields were established to construct the structure. Each mesh field represented a three-dimensional space measuring 16.2 meters long, 10.2 meters wide and 4 meters tall. Each mesh field consisted of 80 grid points in the X-direction, 50 grid points in the Y-direction and 20 grid points in the Z-direction. In this situation the mesh fields were stacked in the model domain. The first mesh field represented the basement level and the second mesh field represented the first floor. 20 cm grid resolution resulted in 160,000 cubic grid squares. When simulations were completed in the 10 cm grid spacing, 12 mesh fields were established. Each mesh field measured 5.4 meters long, 5 meters wide and 4 meters tall. Each mesh field contained 54 grid points in the X-direction, 50 grid points in the Y-direction and 40 grid points in the Z-direction. Each floor contained six mesh fields, arranged three long and two wide. The 10 cm grid resolution resulted in 1,296,000 cubic grid squares.

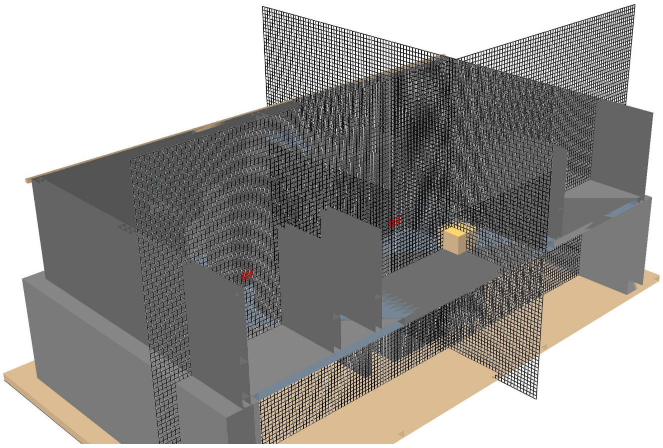

(a) Grid Resolution

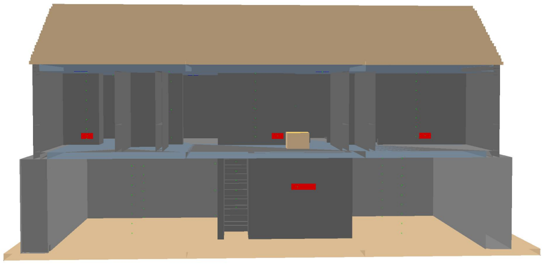

(b) Interior Structure View with Living Room Burner

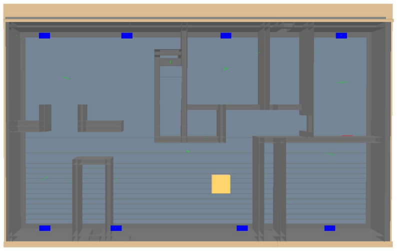

(c) First Floor Aerial View With Supply Vents

Figure 4.1: FDS Smokeview Outputs

To check the applicability of the mesh resolution, an equation provided in the FDS Users Guide [8] was used to calculate the non-dimensional characteristic fire diameter, D. D was calculated for a heat release rate of 121 kW and 393 kW to investigate the values at the range of heat release rates used in these experiments. For the lower HRR, D was calculated to be 0.409 and for the higher HRR, D equaled 0.655. Dividing these values by the nominal size of a mesh grid yields the number of cells spanning the characteristic diameter of the fire. The greater the number of cells spanning the characteristic fire, the greater the resolution. Dividing our D values by 0.1 m yields 4.09 for the 121 kW fire and 6.55 for the 393 kW fire. These calculated values correlate to a rough grid size for the size of the fires specified [5]. In future work, the significance of these values and their effect on results should be investigated.

Once grid scaling was established, the layout of the structure needed to be modeled. Measurements were taken in the DelCo structure and transferred to the model grid space by rounding to the nearest 10 cm increment. To construct the layout, obstructions were created to simulate walls, floors and ceilings. Surface properties were assigned to obstructions to represent drywall and concrete block. Doorways and windows were created by defining holes in the obstructions. The propane gas burner was simulated by using a series of obstructions to create a box platform. A surface property that simulated the heat release rate of the propane burner was assigned to the top of the box platform. The fuel source was then specified to represent propane.

After laying out the structure and gas burner the HVAC system needed to

be modeled. FDS utilizes an HVAC submodel that will compute all HVAC flows separate from the domain of the structure. In the submodel, grid spacing does not need to be defined. Necessary information about the HVAC system is coded into the original file and read in by the HVAC submodel to calculate losses and heat transfer. Several components were needed to define the HVAC system in FDS. Three of the defining HVAC categories are vents, nodes and ducts. A vent is any location that connects the domain to the HVAC submodel. A node is any specific point in the HVAC domain that is associated with a component of the HVAC system. A duct is an element defined in the original file and utilized by the submodel to calculate losses and heat transfer. The first elements placed were the vents. Supply and return vent locations were modeled on surfaces in the structure and given unique IDs for reference. Supply and return vents were distinguished in the model by color, blue for supply and red for return. In our structure all the supply vents were located on the ceilings and all return vents were located on walls. Nodes were placed next to be able to connect vents and ductwork. Every aspect of the ductwork, whether it be where a vent connects to the ductwork or where a tee splits one duct into several, was assigned a specific node ID. After establishing all the nodes, the ductwork was added. To place a section of ductwork, the following information was needed: nodes that were on either end, area of the duct, minor losses in the duct, length, roughness, and how many computational spaces are in the duct.

A fan had to be placed in the ductwork to simulate air flow generated within the HVAC system. A fan curve was determined based on information obtained in the guide for the furnace model used in the DELCO structure [21].

Another feature of FDS that was heavily utilized in these simulations was the ability to place measurement instrumentation in the model. Every piece of instrumentation that was utilized in the DELCO structure was measured and modeled into FDS. These devices included temperature, pressure, gas concentrations and velocities at select locations. By modeling all the devices, comparisons between the model simulations and the gas burner experiments were able to be analyzed for similarities and differences. In addition to all the instrumentation included in the real test structure, FDS was able to place measurement devices within every duct segment in the structure. These measurements included velocity, volume flow, temperature and pressure.

The pressure solver utilized within the FDS simulations was changed from the default, CrayFishpak FFT, to an optional solver called UGLMAT. This was accomplished by setting the 'Solver' equal to 'UGLMat' on the pressure line. Per the FDS Users Guide ([8]), UGLMAT addresses pressures by solving the Poisson equation in the global discretization matrix. As discussed in the manual's example case dealing with duct flow, the UGLMAT solver provided a quicker and more accurate simulation for the provided duct flow scenario. UGLMAT enforces impermeability at solid boundaries, thus preventing leakage through objects such as walls and doors. The impermiability of these objects makes it necessary to provide pressure relief holes in the structure to attempt to match the natural leakage of the real structure.

For all of the experiments that were represented by models, key events from that experiment were noted. The time of each event was then related to the start of data collection so that a total run time of the experiment could be provided in

seconds for FDS to use.

Experimental times of interest are shown in Table 4.1 using the pilot confirmation time as a common zero point. These six experiments were selected to model because the HVAC system was off for all of them. Experiments 1 and 12 are bedroom 1 experiments, experiments 15 and 16 are living room experiments and experiments 23 and 25 are basement experiments. The paired experiments are similar but have one doorway altered between them. While Experiment 1 had all exterior doors closed, Experiment 12 was performed with the front door open. Experiment 15 was conducted with the basement stair door open, while Experiment 16 had the door closed. Simlarly, Experiment 23 was conducted with the basement stair door open and Experiment 25 with the doorway closed.

Table 4.1: Experiment Events of Interest, time in seconds

Test Number	Pilot Confirmation	Ignition	Burner Out
1	0	136	977
12	0	97	1071
15	0	94	1860
16	0	85	1857
23	0	94	1279
25	0	97	1048

A complete FDS script is provided for Experiment 1 in Appendix C.

Chapter 5: Discussion of Experimental Results

This section reviews the transport of products of combustion through the HVAC system to various parts of the structure. Four events common to all experiments were identified on the graphs. These four events were as follows: the confirmation of the pilot line (Pilot Confirmed), the ignition of the gas burner (Ignition), turning off the propane tanks (Stop Gas), and finally the extinction of flames at the gas burner (Burner Out). To standardize time scales across all experiments, each experimental graph begins with the confirmation of ignition of the gas burner pilot line at a time of zero seconds.

5.1 Bedroom 1 Experiments

Gas Burner Experiments 1 through 14 were performed with the gas burner located in bedroom 1. For all bedroom 1 experiments, the door to bedroom 2 was open and the door to bedroom 3 was closed. Three gas concentration measurements were obtained on the first floor and one measurement in the basement. The basement gas concentration measurement was located on the D side of the structure. To protect the windows in bedroom 1 from failure due to repeated heat exposure and to reduce rehab time between experiments, durarock was placed inside bedroom 1 to

protect the windows. The durarock was replaced as needed to protect the windows during all bedroom 1 experiments.

5.1.1 Experiment 1

Gas Burner Experiment 1 was performed with the HVAC system off. The flow rate of propane to the gas burner was controlled so that a heat release rate of approximately 121 kW was produced. The door to bedroom 1 was open as well as the staircase door to the basement. All exterior vents to the structure were closed. For this experiment, gas concentration measurements on the first floor were taken in bedroom 1, bedroom 2, bedroom 3. Experiment 1 was performed to develop a baseline for how heat and fire gases were transported within the HVAC ductwork with the system turned off. The events of interest for Experiment 1 are presented in Table 5.1.

Table 5.1: Experiment 1 Events

Event	Elapsed Time (seconds)
Pilot Confirmed	0
Ignition	136
Stop Gas	857
Burner Out	977

The time versus temperature graphs from the three bedrooms, the living room, the dining room and the kitchen are provided in Figure 5.1. Temperatures in bedroom 1 increased approximately 250 °C at the ceiling and approximately 50 °C at the 0.3 m height while the gas burner was on. Temperatures in bedroom 2 increased approximately 100 °C at the ceiling and approximately 20 °C at the 0.3 m height

while the gas burner was on. Temperatures in bedroom 3 increased approximately 20 °C at the ceiling and had minor changes at the 0.3 m height while the gas burner was on. Temperatures in the living room increased approximately 100 °C at the ceiling and approximately 20 °C at the 0.3 m height while the gas burner was on. Temperatures in the dining room increased approximately 70 °C at the ceiling and approximately 15 °C at the 0.3 m height. Temperatures in the kitchen increased approximately 60 °C at the ceiling and 15 °C at the 0.3 m height while the gas burner was on.

The temperatures in bedroom 2 and the living room increased with a similar trend to that of bedroom 1 due to convective heating through the open bedroom doorway. The magnitude of the temperature increase in bedroom 2 was less than bedroom 1 due to the distance away from the burner. The similar increase in temperature between the living room and bedroom 2 was due to the thermocouple trees being a similar distance away from the burner. The temperature increases in the dining room and the kitchen increased with a similar trend and a lower magnitude to the living room because of the further distance from the burner. The temperature increase in bedroom 3 was due to the convective movement of heat through the HVAC duct network. Both bedroom 2 and bedroom 3 were in close proximity to the burner in bedroom 1, but the main difference that limited the convective heat flow into bedroom 3 was the closed bedroom 3 door.

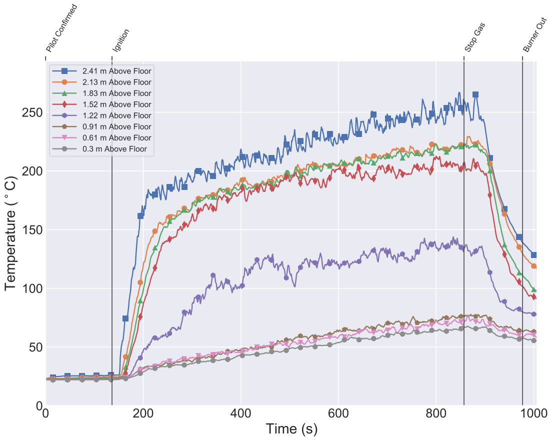

(a) Bedroom 1 Temperatures

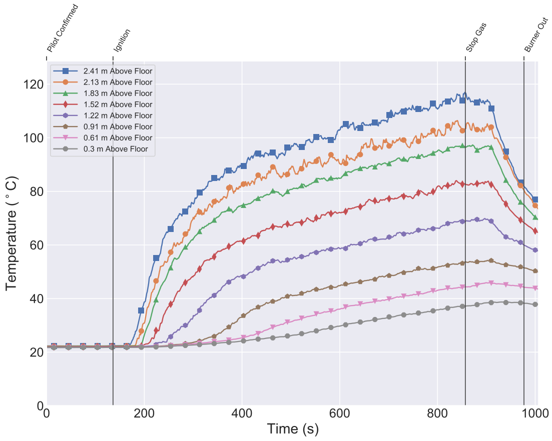

(b) Bedroom 2 Temperatures

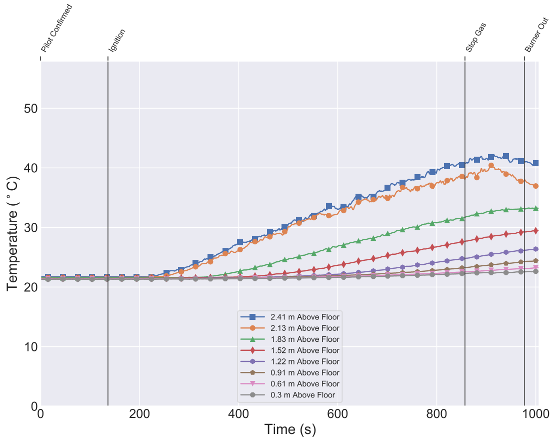

(c) Bedroom 3 Temperatures

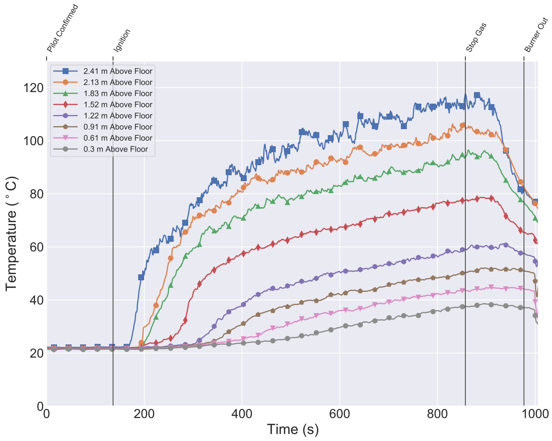

(d) Living Room Temperatures

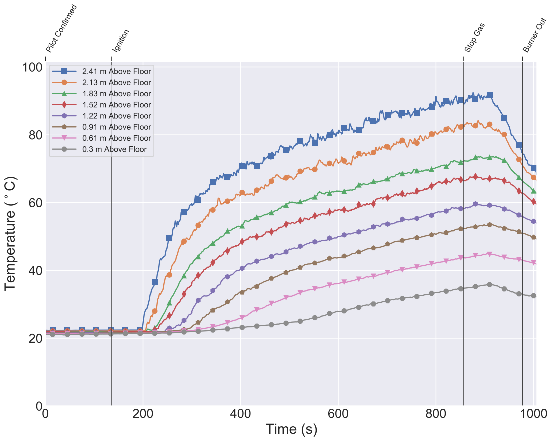

(e) Dining Room Temperatures

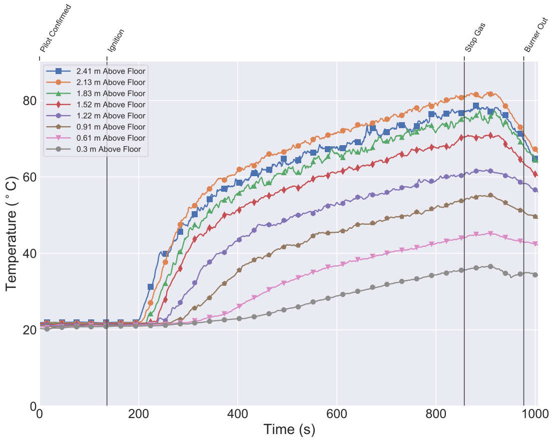

(f) Kitchen Temperatures

Figure 5.1: Experiment 1 First Floor Temperatures

The time versus temperature graphs for the four basement quadrants are provided in Figure 5.2. From the figure it can be seen that with a fire in bedroom 1, there was no notable temperature increase in the basement.

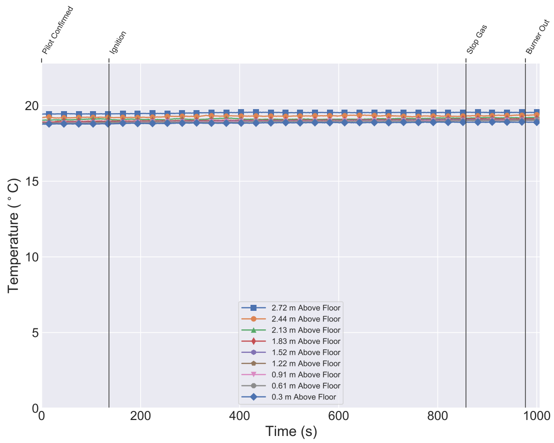

(a) Basement A Quadrant Temperatures

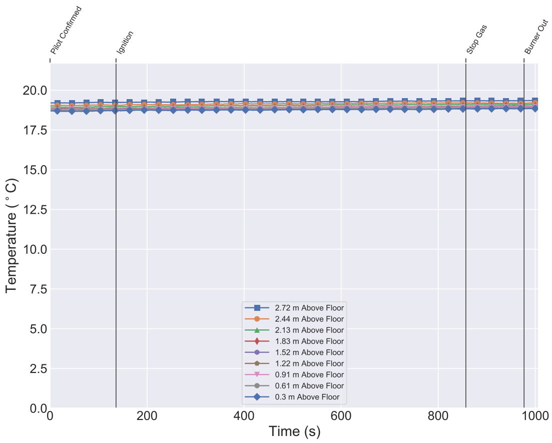

(b) Basement B Quadrant Temperatures

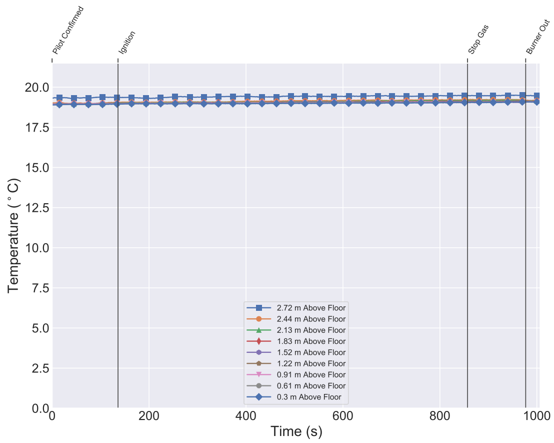

(c) Basement C Quadrant Temperatures

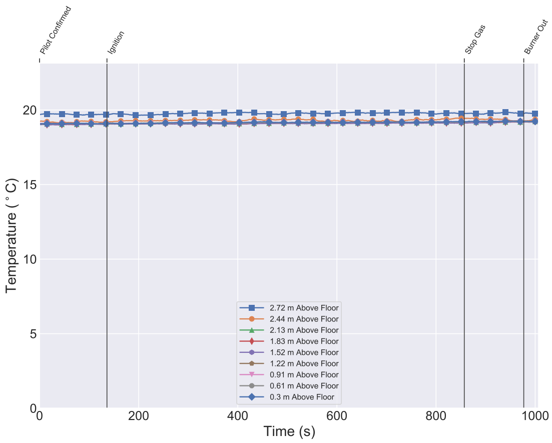

(d) Basement D Quadrant Temperatures

Figure 5.2: Experiment 1 Basement Temperatures

The pressures within each room are shown in Figure 5.3. Pressure in bedroom 1 at 2.13 m above the floor exceeded the ambient pressure by approximately 5 Pa while the burner was on. At 1.2 m above the floor pressure exceeded the ambient by

approximately 2 Pa. At 0.3 m above the floor pressure remained at approximately ambient with a small spike following ignition. Pressures in bedroom 2 followed a similar trend with the exception of the 0.3 m height fluctuating between 1 to 2 Pa above ambient. Pressures in bedroom 3 were similar at all height measurements with a peak of 3 to 4 Pa above ambient shortly after ignition. Pressures in bedroom 3 mostly fluctuated between 1 to 2 Pa above the ambient pressure while the gas burner was on. Pressures in the living room and kitchen were similar to those recorded in bedroom 2. Pressures in the basement remained in the 1 to 2 pascal range for the majority of the time that the burner was on with the exception of a positive spike shortly after ignition and a negative spike after the gas was shut off.

The highest pressures were recorded close to the ceiling in a room. This is a result of the following fundamentals of physics. Hot gases produced by the fire were driven to the ceiling due to buoyancy. As the gases were heated they expanded within the confinements of the structure. Applying the ideal gas law to a fixed volume structure it can be seen that as the temperature of a gas increases, there is a direct correlation to the pressure. Gases will naturally flow from areas of high pressure to areas of low pressure. A negative pressure measurement indicates that air and gases are being pulled into that area from other parts of the structure. The spike in pressure in bedroom 3, the basement and the kitchen shortly after ignition shows that there was a measurable pressure surge in the HVAC ducts that spread through the strucure. This is important to note because it highlights how interconnected the structure is due to the HVAC ducts.

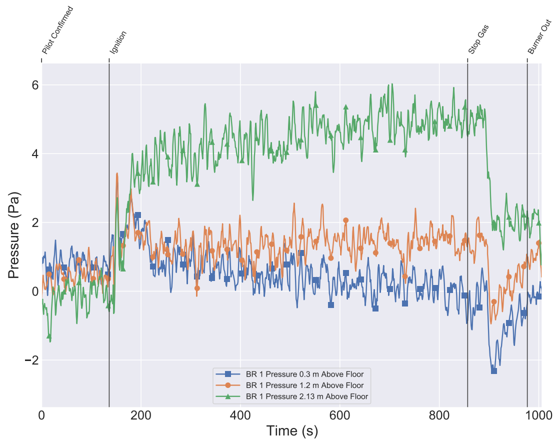

(a) Bedroom 1 Pressures

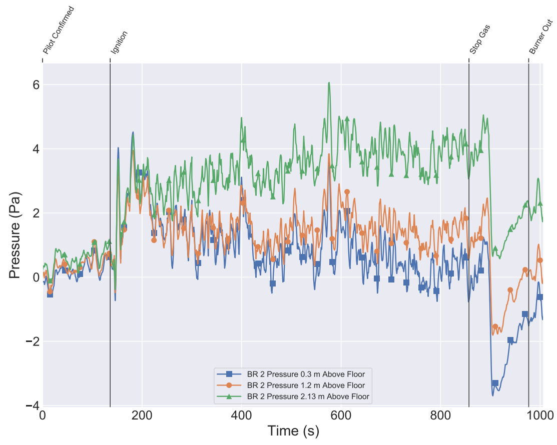

(b) Bedroom 2 Pressures

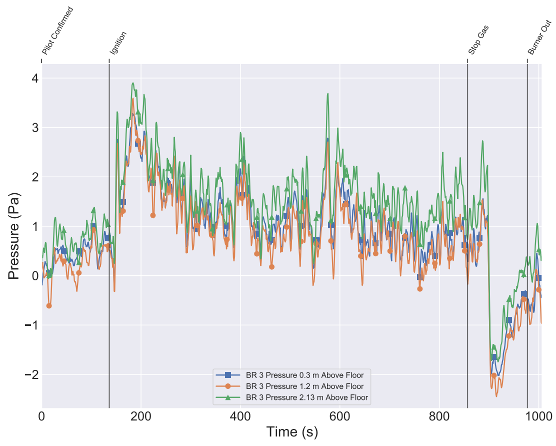

(c) Bedroom 3 Pressures

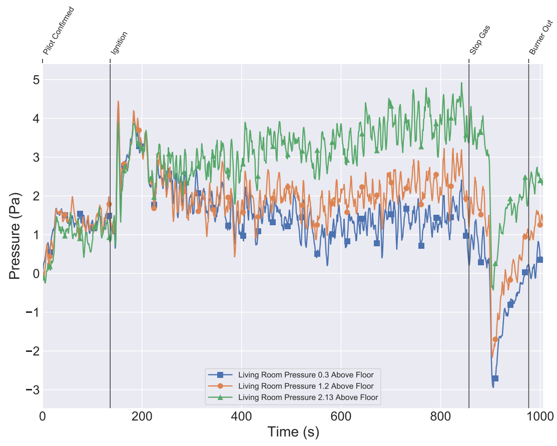

(d) Living Room Pressures

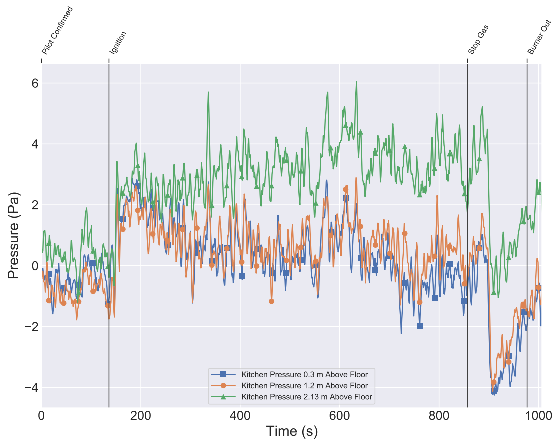

(e) Kitchen Pressures

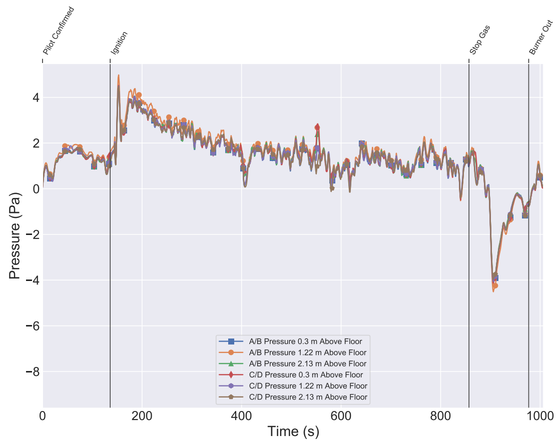

(f) Basement Pressures

Figure 5.3: Experiment 1 Pressures

The concentrations of oxygen and carbon dioxide are provided for the three bedrooms and the basement in Figure 5.4. Water vapor concentrations are included for bedroom 2, bedroom 3 and the basement. Gas concentrations in bedrooms 1 and 2 followed a similar trend with oxygen concentrations dropping to around 17% while the burner was on. Carbon dioxide concentrations in bedrooms 1 and 2 reached approximately 2%. Water vapor concentrations in bedroom 2 increased approximately 3% while the burner was on. Bedroom 3 displays a slight change in both oxygen and carbon dioxide concentrations prior to the gas burner going out. Water vapor concentrations in bedroom 3 showed no notable change. While the gas concentrations in bedroom 3 do not change as much as the other bedrooms in magnitude, there is still a change that could be attributed to gas flow through the HVAC ductwork. Gas and water vapor concentrations in the basement did not show any notable changes while the gas burner was on.

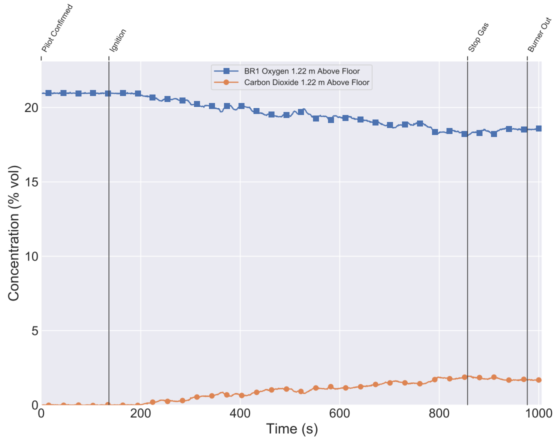

(a) Bedroom 1 Gases

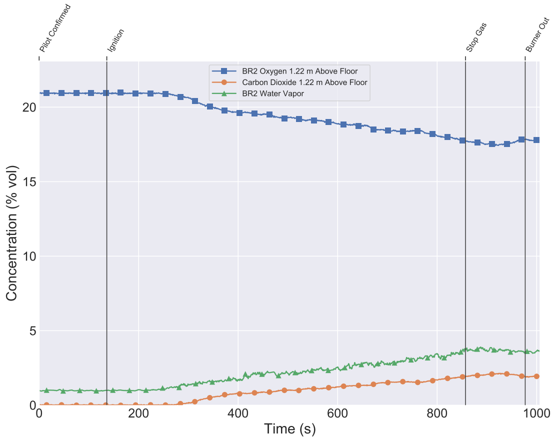

(b) Bedroom 2 Gases

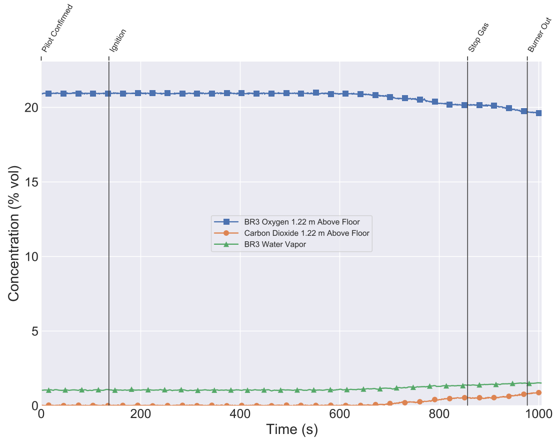

(c) Bedroom 3 Gases

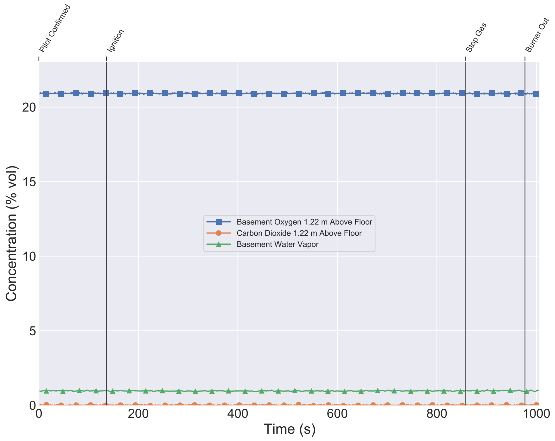

(d) Basement Gases

Figure 5.4: Experiment 1 Gases

Figure 5.5 shows the temperatures of the supply and return vents in the structure. It can be seen that the temperature at the supply vent in bedroom 1 is the same as the ceiling temperature in bedroom 1. The supply vent temperature in bedroom 2 follows the same trend as the ceiling temperature in bedroom 2. The supply vent temperature for bedroom 3 tells a different story than the supply vent of bedroom 2. The temperature of the supply vent in bedroom 3 rises at a rate

much faster than the ceiling temperature in bedroom 3 and increases by a temperature of approximately 80 °C. This supply vent temperature being greater than the ceiling temperature in bedroom 3 shows that the heat increase in the room is due to heat transfer through the HVAC supply ducts. Looking at the return vent temperatures, it can be seen that no measurable heat flow occurred through the return ductwork because all return temperatures trended with the room temperatures at their respective heights.

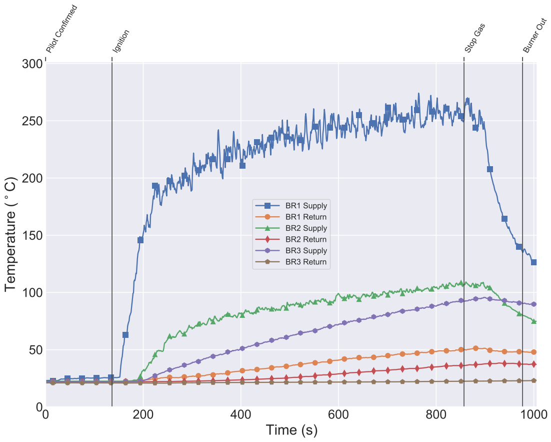

(a) Bedroom Vent Temperatures

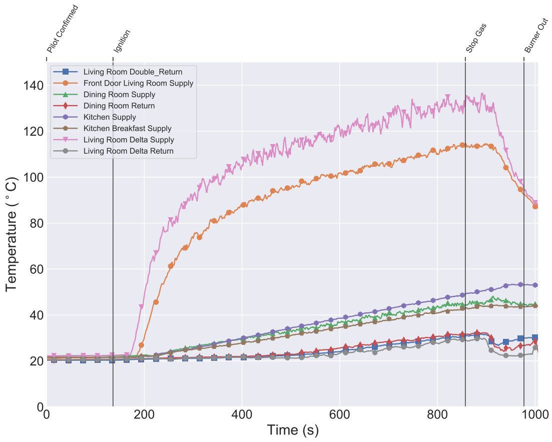

(b) First Floor Vent Temperatures

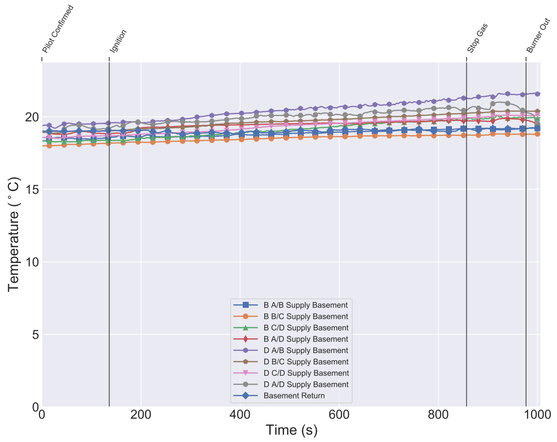

(c) Basement Vent Temperatures

Figure 5.5: Experiment 1 Vent Temperatures

Comparison of the Living Room supply vents show that both follow a similar trend to that of the ceiling temperature in that room. The supply vent temperatures for both Kitchen vents and the Dining Room vent increase at a rate slower than that of the ceiling temperature in their respective room. Similar to the bedroom return vents, the return ductwork does not apprear to contribute any heat transfer to the return vents in the rest of the structure.

By observation of the basement supply vent temperatures it appears that there could be a small amount of heat transfer through the supply ductwork into the basement. The temperatures of the supply vents appear to be a few degrees Centigrade greater than the ceiling temperatures recorded in the basement.

The temperature and velocity within the supply duct are provided in Figure 5.6. From this figure it can be seen that there is a temperature increase within the supply duct of approximately 25 °C while the gas burner was on. The increase in temperature is consistent with what was observed from the supply vent temperatures in the basement but with greater magnitude. The supply vent velocity appears to show that there is a constant flow of air in the supply vent from the first floor to the basement, however this held constant from before ignition to after termination of the burner.

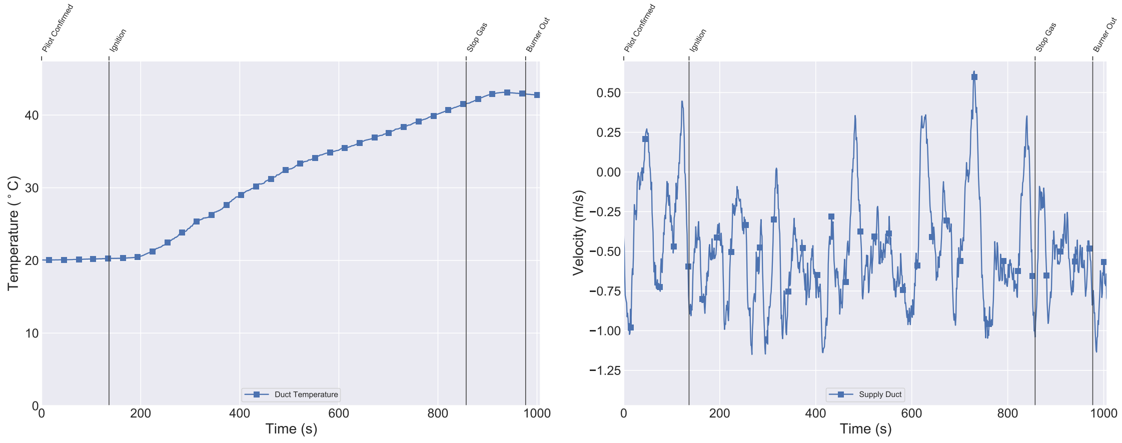

(a) Supply Duct Temperature

(b) Supply Duct Velocity

Figure 5.6: Experiment 1 Supply Duct Temperature and Velocity

5.1.2 Experiment 2

Gas Burner Experiment 2 was performed with the HVAC system set to A/C. The flow rate of propane to the gas burner was controlled so that a heat release rate of approximately 121 kW was produced. The door to bedroom 1 was open as well as the staircase door to the basement. All exterior vents to the structure were closed. For this experiment, gas concentration measurements on the first floor were taken in bedroom 1, bedroom 2, bedroom 3. Experiment 2 was performed to develop a baseline for how heat and fire gases were transported within the HVAC ductwork with the system turned to A/C. The events of interest for Experiment 2 are presented in Table 5.2.

Table 5.2: Experiment 2 Events

Event	Elapsed Time (seconds)
Pilot Confirmed	0
Ignition	114
Stop Gas	1076
Burner Out	1150

The time versus temperature graphs from the three bedrooms, the living room, the dining room and the kitchen are provided in Figure 5.7. Temperatures in bedroom 1 increased approximately 275 °C at the ceiling and approximately 35 °C at the 0.3 m height while the gas burner was on. Temperatures in bedroom 2 increased approximately 100 °C at the ceiling and approximately 25 °C at the 0.3 m height while the gas burner was on. Temperatures in bedroom 3 increased approximately 20 °C at the ceiling and showed a slight increase at the 0.3 m height while the gas burner was on. Temperatures in the living room increased approximately 100 °C at the ceiling and approximately 25 °C at the 0.3 m height while the gas burner was on. Temperatures in the dining room increased approximately 80 °C at the ceiling and approximately 20 °C at the 0.3 m height. Temperatures in the kitchen increased approximately 70 °C at the ceiling and 20 °C at the 0.3 m height while the gas burner was on.

The temperatures on the first floor reach higher maximum values than those reported in Experiment 1, but this is due to a longer run time of the gas burner. One point of interest to note from Figure 5.7(c) is that the temperature increase in bedroom 3 appears to be slower for most of the experiment than compared to Experiment 1. The rapid increase that occurs towards the end of the experiment

could indicate that the fire had grown to a size where it could overpower the outward flow of air from the supply trunk and push into the branch to bedroom 3.

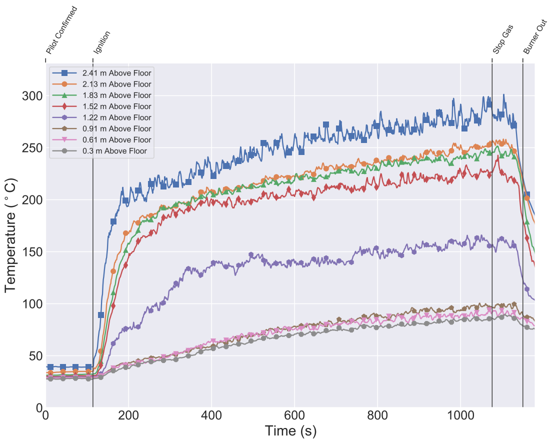

(a) Bedroom 1 Temperatures

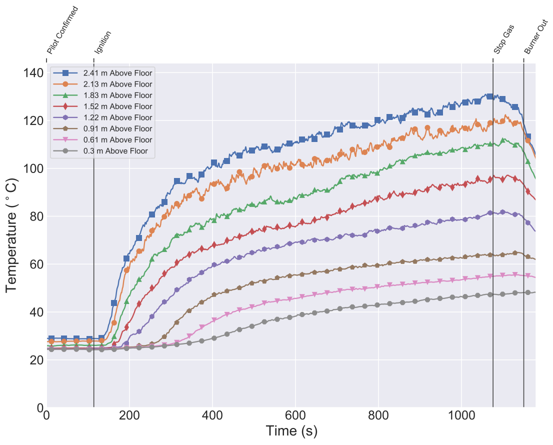

(b) Bedroom 2 Temperatures

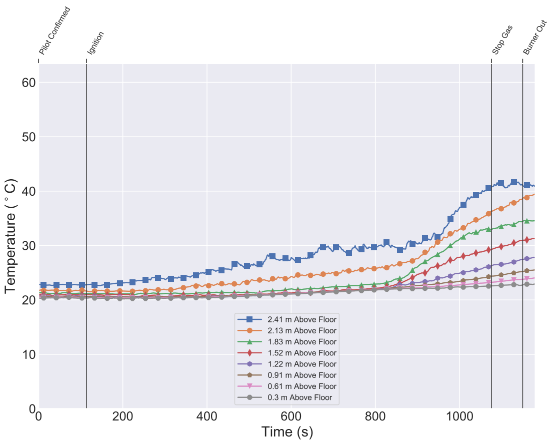

(c) Bedroom 3 Temperatures

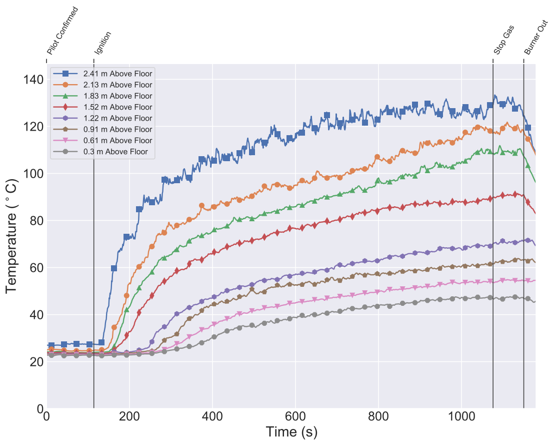

(d) Living Room Temperatures

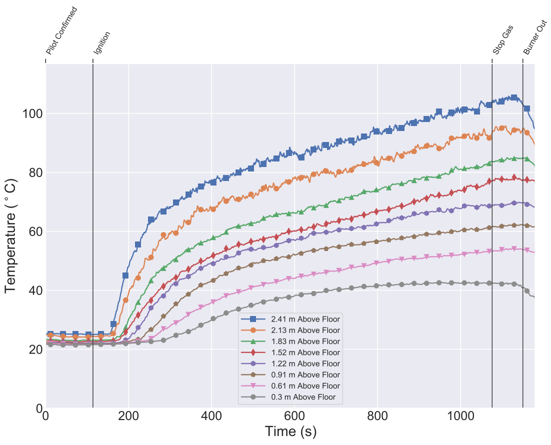

(e) Dining Room Temperatures

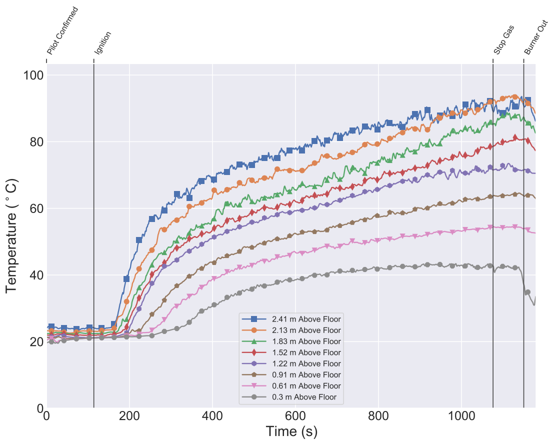

(f) Kitchen Temperatures

Figure 5.7: Experiment 2 First Floor Temperatures

The time versus temperature graphs for the four basement quadrants are provided in Figure 5.8. From the figure it can be seen that with a fire in bedroom 1, there was no notable temperature increase in the basement with the HVAC system on.

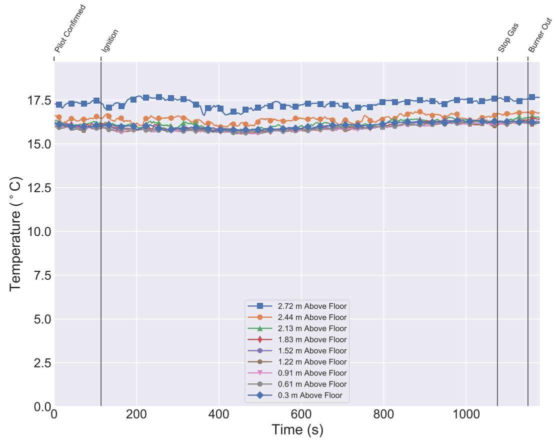

(a) Basement A Quadrant Temperatures

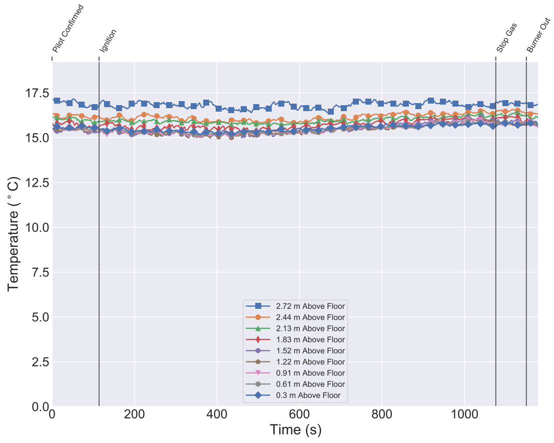

(b) Basement B Quadrant Temperatures

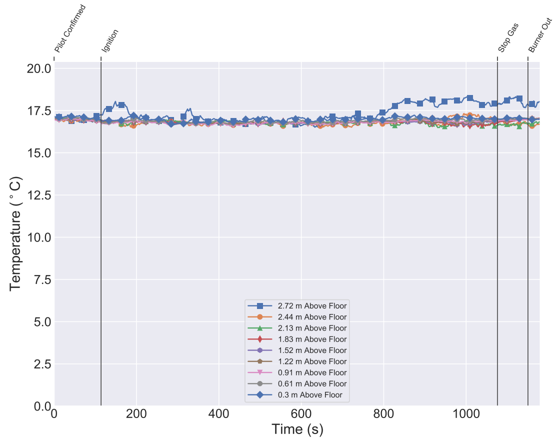

(c) Basement C Quadrant Temperatures

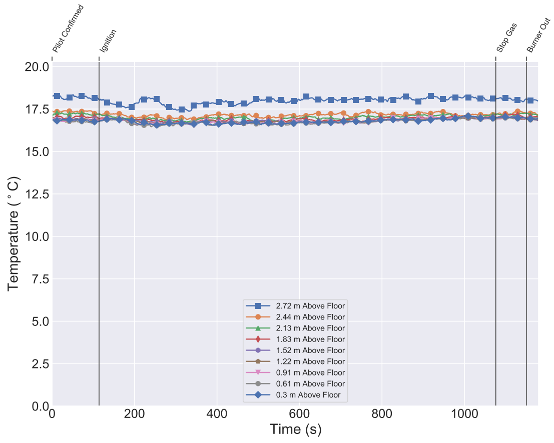

(d) Basement D Quadrant Temperatures

Figure 5.8: Experiment 2 Basement Temperatures

The pressures within each room are shown in Figure 5.9. Pressure in bedroom 1 at 2.13 m above the floor exceeded the ambient pressure by approximately 6 Pa

while the burner was on. At 1.2 m above the floor pressure exceeded the ambient by approximately 1 Pa. At 0.3 m above the floor pressure remained at approximately 1 Pa below the ambient with a small spike above the ambient pressure following ignition. Pressures in bedroom 2 followed a similar trend with a maximum at 2.13 m exceeding the ambient by approximately 4 Pa and at the 0.3 m height the pressure fluctuated between 0 and 1 Pa above the ambient. Pressures in bedroom 3 were similar at all height measurements with a peak of 3 to 4 Pa above ambient shortly after ignition. Pressures in bedroom 3 mostly fluctuated between 0 and 2 Pa above the ambient pressure while the gas burner was on. Pressures in the living room and kitchen were similar to those recorded in bedroom 2. Pressures in the basement remained close to zero pascals for the majority of the time that the burner was on with the exception of a positive spike up to 4 Pa shortly after ignition.

Pressures in Experiment 2 followed similar trends to those of Experiment 1. From this comparison it would appear that the HVAC system being on in the A/C mode did not affect the pressures while the burner was on.

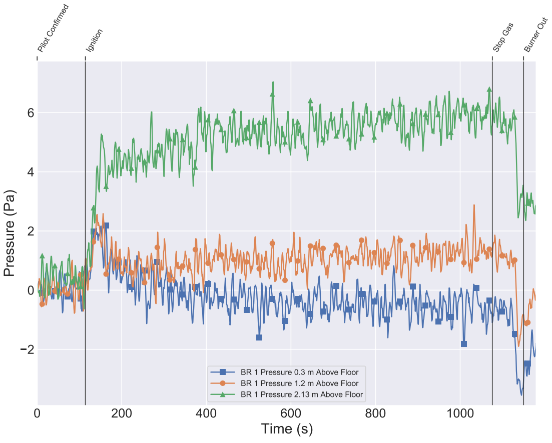

(a) Bedroom 1 Pressures

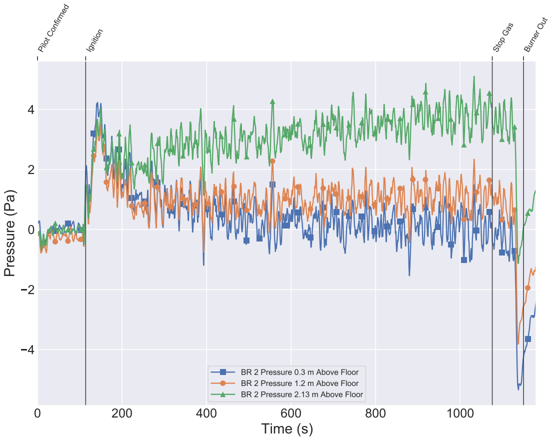

(b) Bedroom 2 Pressures

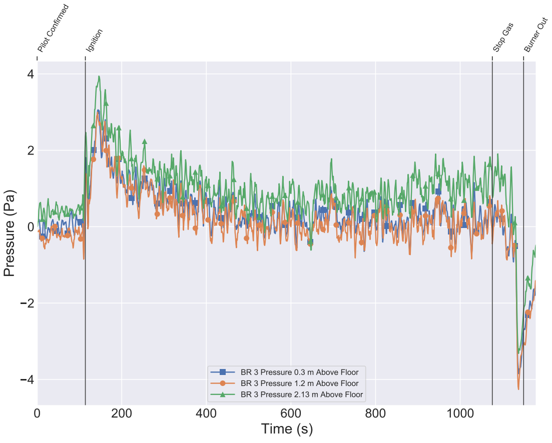

(c) Bedroom 3 Pressures

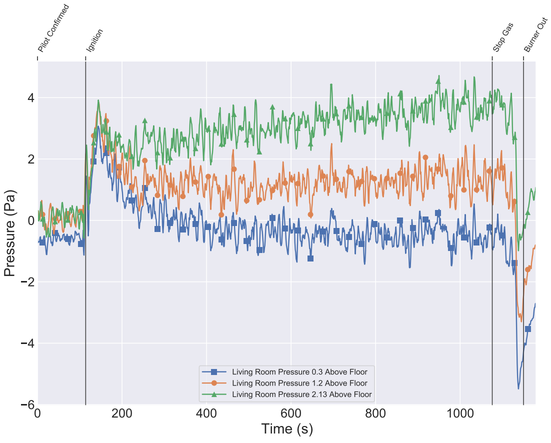

(d) Living Room Pressures

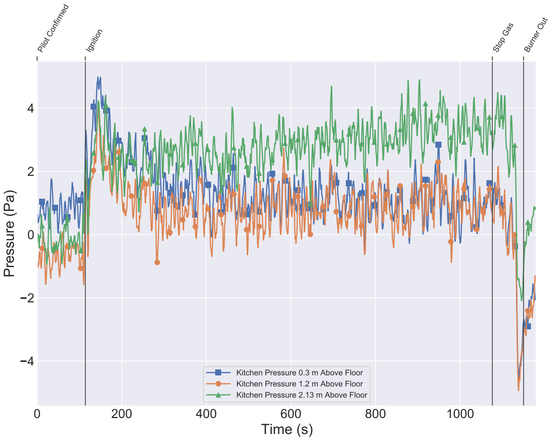

(e) Kitchen Pressures

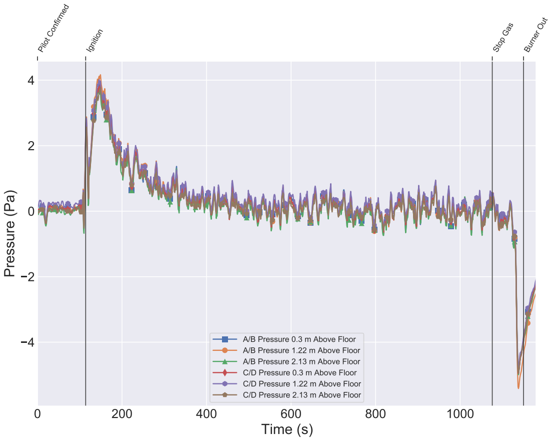

(f) Basement Pressures

Figure 5.9: Experiment 2 Pressures

The concentrations of oxygen and carbon dioxide are provided for the three bedrooms and the basement in Figure 5.10. Water vapor concentrations are included for bedroom 2, bedroom 3 and the basement. Oxygen concentrations in bedroom 1 dropped to around 17% while the burner was on. Carbon dioxide concentrations in bedroom 1 increased approximately 3%. Oxygen concentrations in bedroom 2 dropped to around 16% while the burner was on. Carbon dioxide concentrations in bedroom 2 increased approximately 3%. Water vapor concentrations in bedroom 2 increased approximately 4% while the burner was on. Oxygen concentrations in bedroom 3 dropped approximately 1% while the burner was on but continued to drop after the burner was shut off. Carbon dioxide concentrations in bedroom 3 increased approximately 1%. Water vapor concentrations in bedroom 3 showed a slight increase during the experiment. Gas and water vapor concentrations in the basement did not show any notable changes while the gas burner was on.

The continued decrease of oxygen and increase of carbon dioxide in bedroom 3 following the termination of the gas burner could be due to a buildup of gases in the HVAC system being distributed throughout the structure.

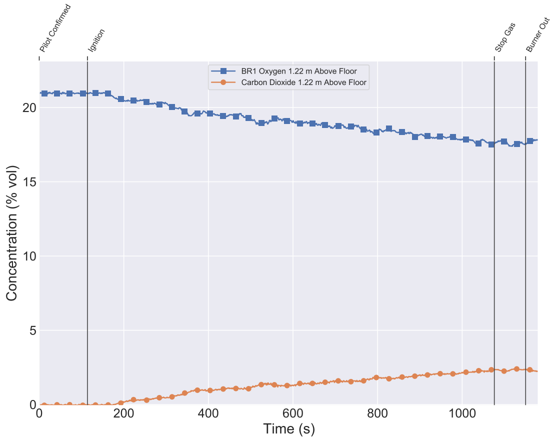

(a) Bedroom 1 Gases

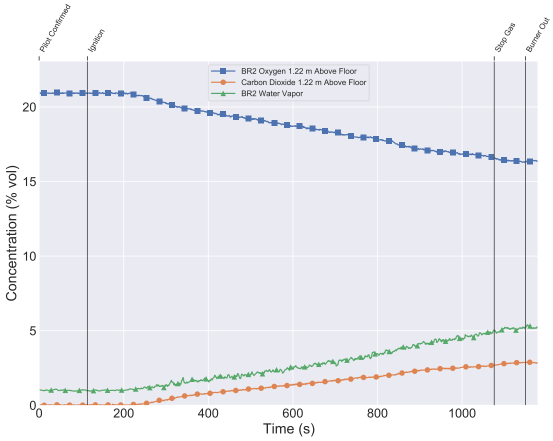

(b) Bedroom 2 Gases

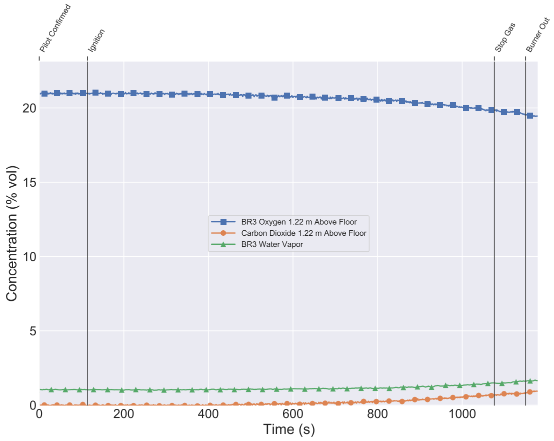

(c) Bedroom 3 Gases

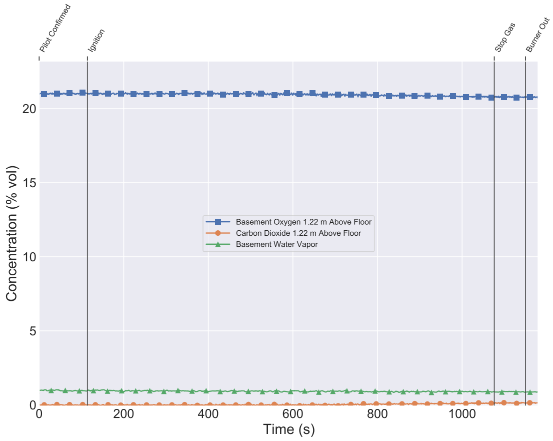

(d) Basement Gases

Figure 5.10: Experiment 2 Gases

Figure 5.11 shows the temperatures of the supply and return vents in the structure. Figure 5.11(a) shows that the supply vent temperatures in the bedrooms are held at a constant and cool temperature for several hundred seconds following ignition. In comparison to the vent temperatures in Experiment 1, it can be seen that the HVAC system being set to A/C keeps the temperatures at the vents cool for a long time before being overpowered by the fire gases and smoke temperature.

In bedroom 1 the supply vent temperature holds constant for almost 500 seconds before climbing to similar temperatures as those measured at the ceiling in the bedroom. In bedroom 2 the supply vent temperature holds constant for a similar time before slowly staggering up to the ceiling temperature. In bedroom 3 the supply vent temperature holds constant for approximately 700 seconds before gradually increasing. For bedroom 3, the temperature increase at the ceiling is caused by heated gases travelling through the HVAC system. Temperatures at the supply duct increase prior to the rest of the room, thus indicating heat transfer from the supply vent to the bedroom.

The return vent temperatures appear to show a similar trend to that seen in Experiment 1. The temperatures slowing increase at a similar rate to that near the floor in each bedroom.

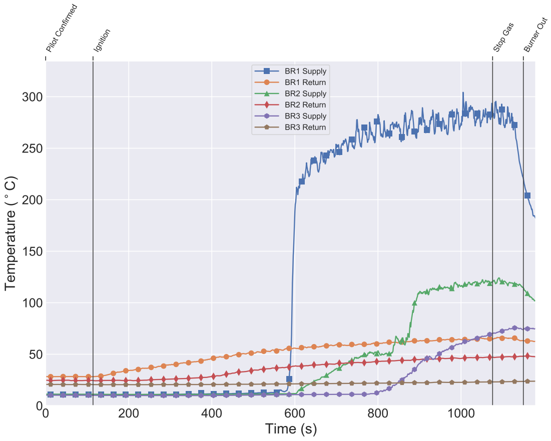

(a) Bedroom Vent Temperatures

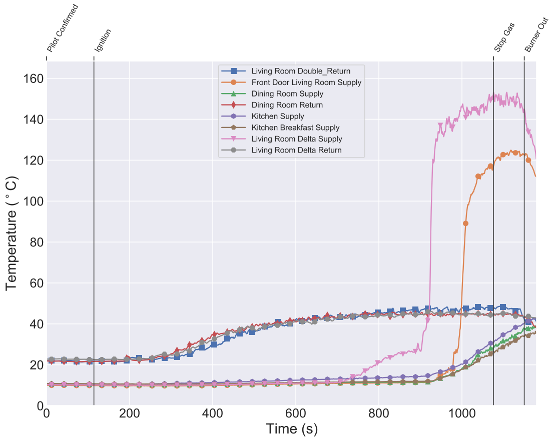

(b) First Floor Vent Temperatures

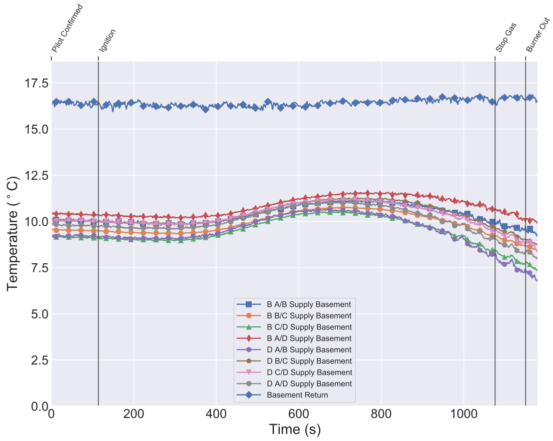

(c) Basement Vent Temperatures

Figure 5.11: Experiment 2 Vent Temperatures

Figure 5.11(b) shows the vent temperatures for the remainder of the first floor. Similar to what was observed in the bedrooms, the supply vent temperatures all stay steady at a constant temperature for a majority of the experiment. Comparison of the living room supply vents show that the supply vent closer to bedroom 1, the delta living room supply, increases sooner than the front door supply. The living room delta supply vent is located on the bedroom 1 side of the vertical supply duct

riser, as compared to the rest of the supply vents displayed in this figure. The influx of conditioned air from the supply riser could be a major contribution to the lower temperatures seen in the supply vents located on the B side of the first floor. The return vents for the remainder of the first floor appear to follow the temperature trends of the thermalcouple probes near the floor in their respective rooms.

Figure 5.11(c) shows the basement vent temperatures. The basement supply vents are continually kept at a temperature lower than the surrounding ceiling temperatures. What is interesting to observe from this graph is that there is a uniform increase in temperature at all supply vents followed by a decrease. This could the result of the system trying to compensate for the increased temperature at the thermostat. The return vent temperature in the basement did not appear to change during this experiment.

The temperature and velocity within the supply duct are provided in Figure 5.12. From this figure it can be seen that the temperature increases slowly over the course of the experiment but never exceeds 15 °C. Similar to the temperatures seen in the basement supply vents, there is a slow increase in temperature within the supply riser followed by a decrease in temperature. This supports the thought that the HVAC system attempted to correct for the increasing temperatures on the first floor. For the majority of this experiment there appears to be a negative velocity within the supply riser which would indicate flow from the first floor to the basement, however that type of flow is not supported by the temperatures recorded in the duct or the basement.

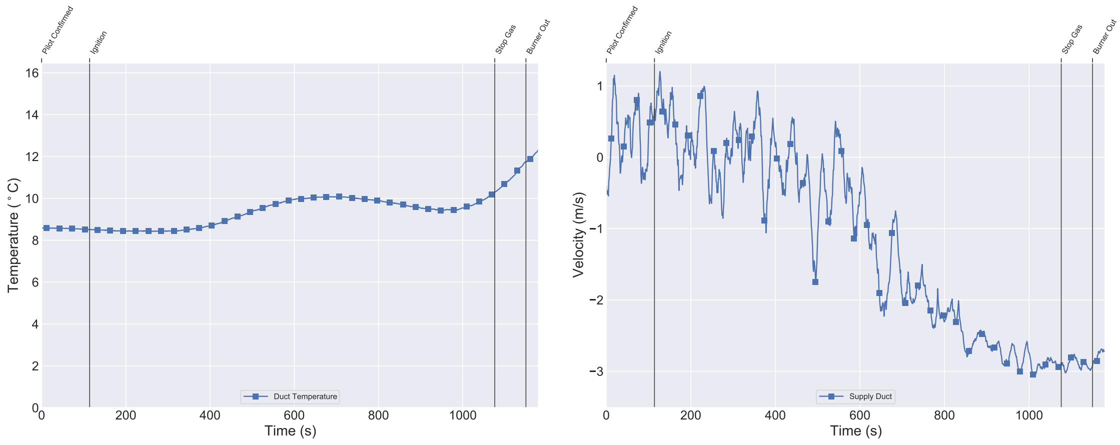

(a) Supply Duct Temperature

(b) Supply Duct Velocity

Figure 5.12: Experiment 2 Supply Duct Temperature and Velocity

5.1.3 Experiment 3

Gas Burner Experiment 3 was performed with the HVAC system set to A/C. The flow rate of propane to the gas burner was controlled so that a heat release rate of approximately 121 kW was produced. The door to bedroom 1 was open as well as the staircase door to the basement. All exterior vents to the structure were closed. For this experiment, gas concentration measurements on the first floor were taken in bedroom 1, bedroom 2, bedroom 3. Experiment 3 was performed as a duplicate to Experiment 2 to see how heat and fire gases were transported within the HVAC ductwork with the system turned to A/C. The events of interest for Experiment 3 are presented in Table 5.3.

Table 5.3: Experiment 3 Events

Event	Elapsed Time (seconds)
Pilot Confirmed	0
Ignition	119
Stop Gas	840
Burner Out	915

The time versus temperature graphs from the three bedrooms, the living room, the dining room and the kitchen are provided in Figure 5.13. Temperatures in bedroom 1 increased approximately 250 °C at the ceiling and approximately 50 °C at the 0.3 m height while the gas burner was on. Temperatures in bedroom 2 increased approximately 100 °C at the ceiling and approximately 20 °C at the 0.3 m height while the gas burner was on. Temperatures in bedroom 3 increased less than 10 °C at the ceiling and showed a slight increase at the 0.3 m height while the gas burner was on. Temperatures in the living room increased approximately 100 °C at the ceiling and approximately 25 °C at the 0.3 m height while the gas burner was on. Temperatures in the dining room increased approximately 75 °C at the ceiling and approximately 20 °C at the 0.3 m height. Temperatures in the kitchen increased approximately 65 °C at the ceiling and 20 °C at the 0.3 m height while the gas burner was on.

In comparison to Experiment 2, the behavior of all temperatures on the first floor trend with the data reported in the previous experiment.

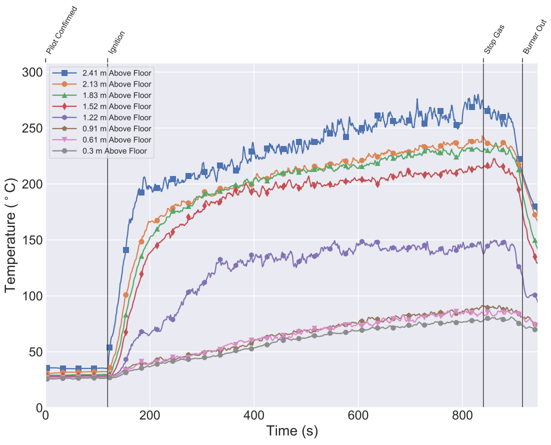

(a) Bedroom 1 Temperatures

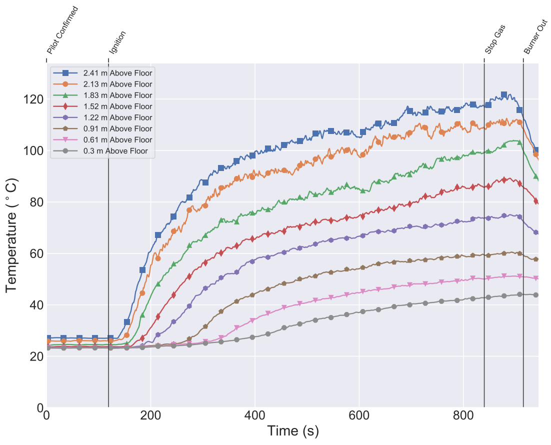

(b) Bedroom 2 Temperatures

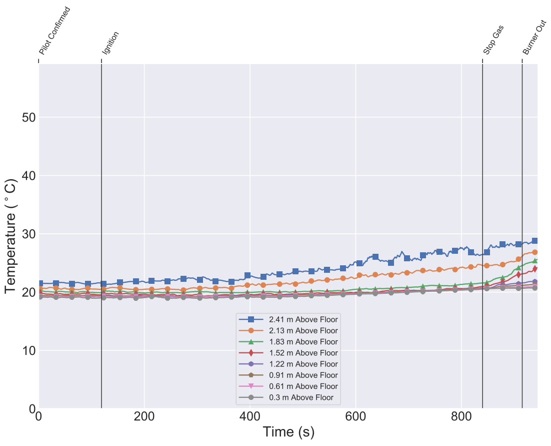

(c) Bedroom 3 Temperatures

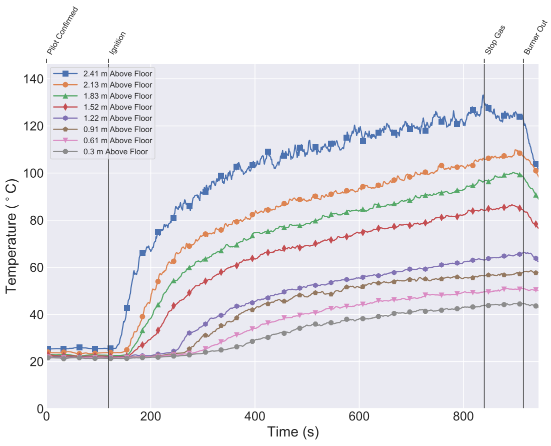

(d) Living Room Temperatures

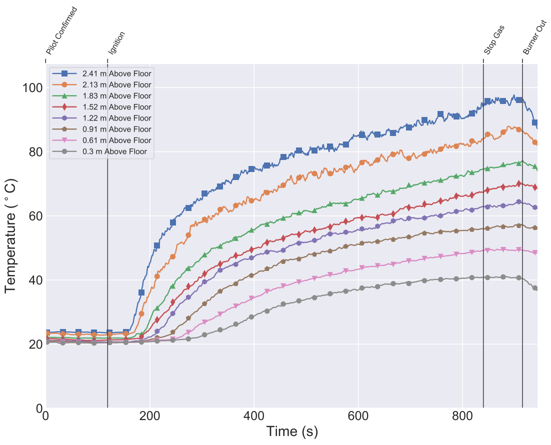

(e) Dining Room Temperatures

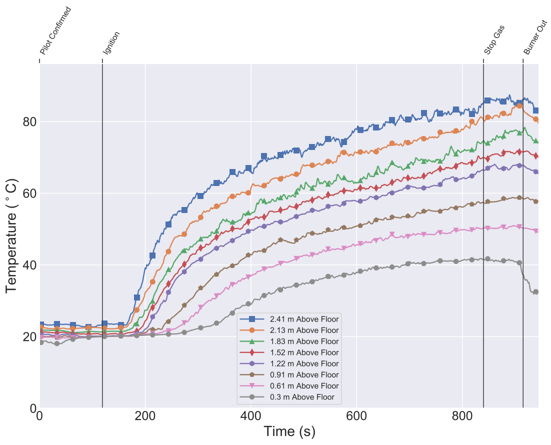

(f) Kitchen Temperatures

Figure 5.13: Experiment 3 First Floor Temperatures

The time versus temperature graphs for the four basement quadrants are provided in Figure 5.8. As was the case in Experiment 2, there was no notable temperature increase in the basement with the HVAC system on.

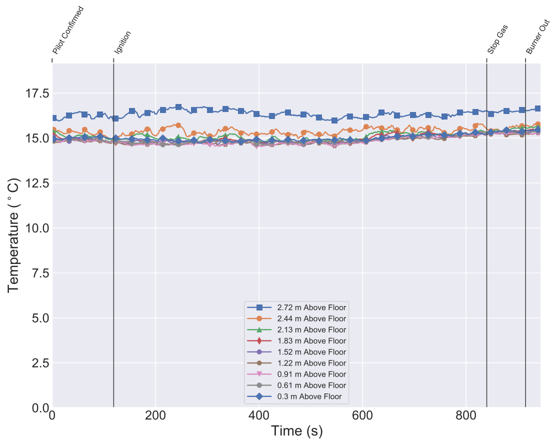

(a) Basement A Quadrant Temperatures

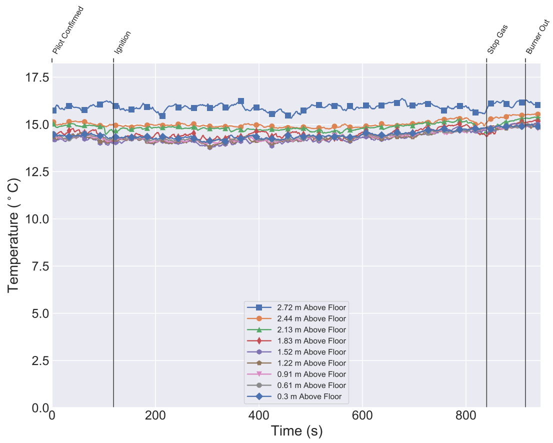

(b) Basement B Quadrant Temperatures

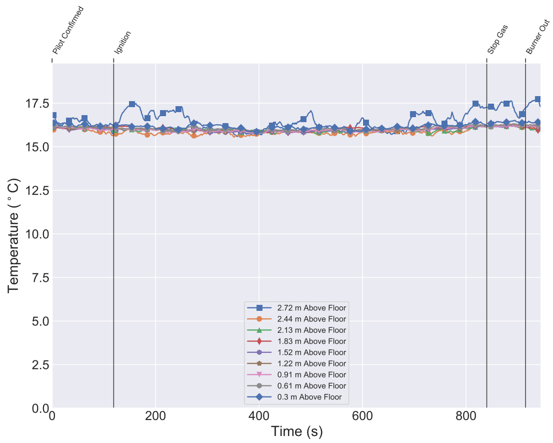

(c) Basement C Quadrant Temperatures

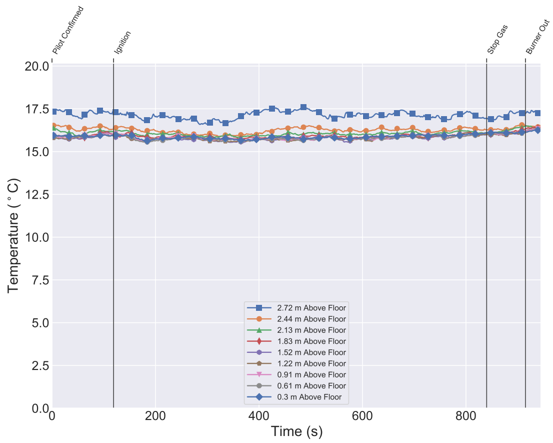

(d) Basement D Quadrant Temperatures

Figure 5.14: Experiment 3 Basement Temperatures

The pressures within each room are shown in Figure 5.15. Pressure in bedroom 1 at 2.13 m above the floor exceeded the ambient pressure by approximately 5 Pa while the burner was on. At 1.2 m above the floor pressure exceeded the ambient by

approximately 1 Pa. At 0.3 m above the floor pressure remained slightly below the ambient pressure with a small spike above the ambient pressure following ignition. Pressures in bedroom 2 followed a similar trend with a maximum at 2.13 m exceeding the ambient by approximately 3 to 4 Pa and at the 0.3 m height the pressure fluctuated between 0 and 1 Pa above the ambient. Pressures in bedroom 3 were similar at all height measurements with a peak of 2 to 3 Pa above ambient shortly after ignition. Pressures in bedroom 3 mostly fluctuated between 1 Pa below the ambient pressure and 1 Pa above the ambient pressure while the gas burner was on. Pressures in the living room and kitchen were similar to those recorded in bedroom 2. Pressures in the basement remained close to zero pascals for the majority of the time that the burner was on with the exception of a positive spike up to 4 Pa shortly after ignition.

For Experiment 3, the pressures followed the same trends as those reported in Experiment 2.

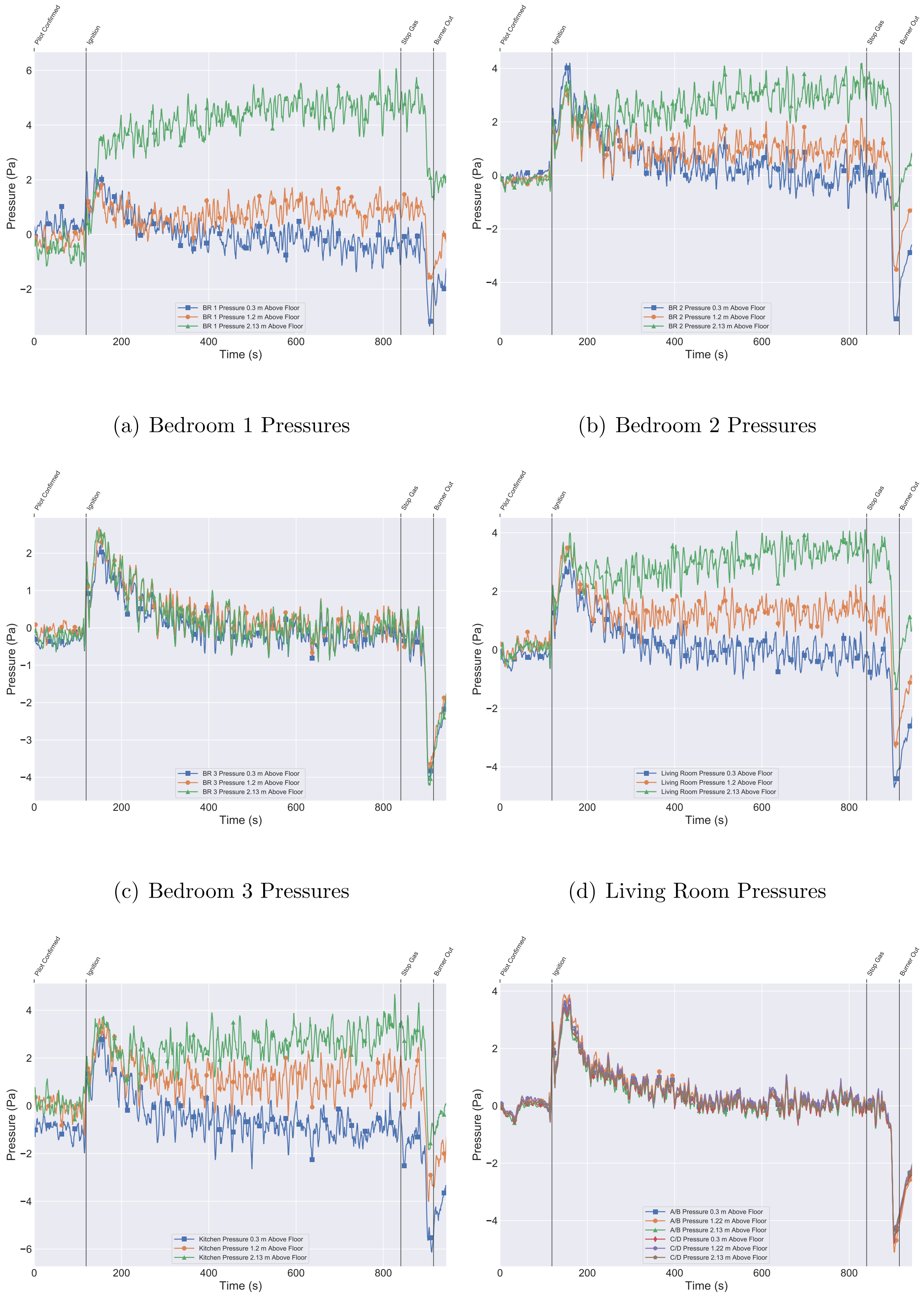

(a) Bedroom 1 Pressures

(b) Bedroom 2 Pressures

(c) Bedroom 3 Pressures

(d) Living Room Pressures

(e) Kitchen Pressures

(f) Basement Pressures

Figure 5.15: Experiment 3 Pressures

The concentrations of oxygen and carbon dioxide are provided for the three bedrooms and the basement in Figure 5.16. Water vapor concentrations are included for bedroom 2, bedroom 3 and the basement. Oxygen concentrations in bedroom 1 dropped to around 18% while the burner was on. Carbon dioxide concentrations in bedroom 1 increased approximately 2%. Oxygen concentrations in bedroom 2 dropped to around 17% while the burner was on. Carbon dioxide concentrations in bedroom 2 increased approximately 3%. Water vapor concentrations in bedroom 2 increased approximately 3% while the burner was on. Oxygen concentrations in bedroom 3 dropped less than 1% while the burner was on. Carbon dioxide concentrations in bedroom 3 showed a slight increase less than 1% during this experiment. Water vapor concentrations in bedroom 3 showed a slight increase during the experiment. Gas and water vapor concentrations in the basement did not show any notable changes while the gas burner was on.

The gas concentrations for Experiment 3 trended similarly to those reported in Experiment 2.

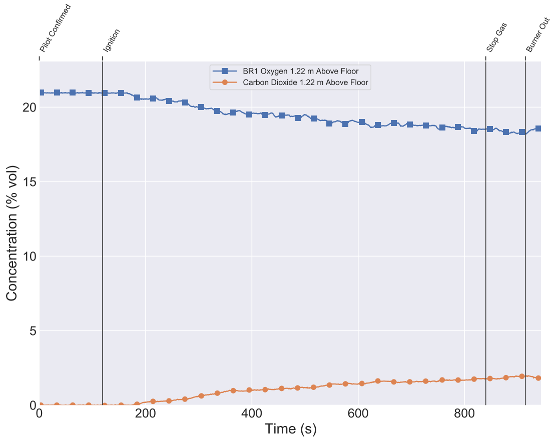

(a) Bedroom 1 Gases

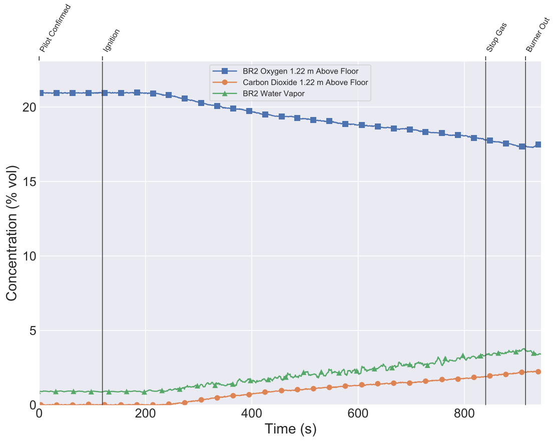

(b) Bedroom 2 Gases

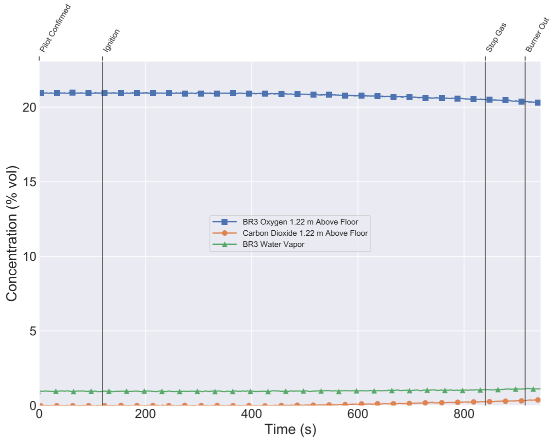

(c) Bedroom 3 Gases

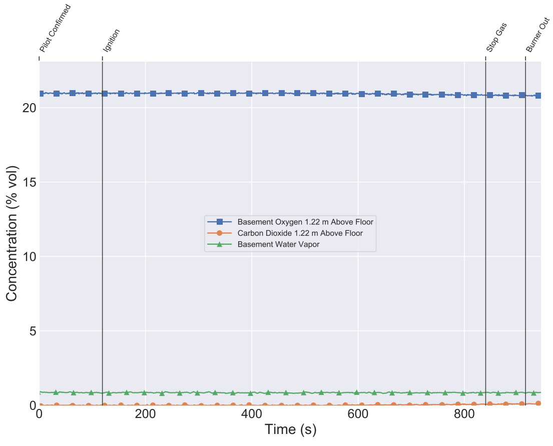

(d) Basement Gases

Figure 5.16: Experiment 3 Gases

Figure 5.17 shows the temperatures of the supply and return vents in the structure. From Figure 5.17(a) it can be seen that the vent temperatures in the bedrooms follow the same trends discussed for Experiment 2. Figure 5.17(b) shows that the vent temperatures for the remainder of the first floor also behave similar to Experiment 2. The same can be said for the basement, as seen in Figure 5.17(c).

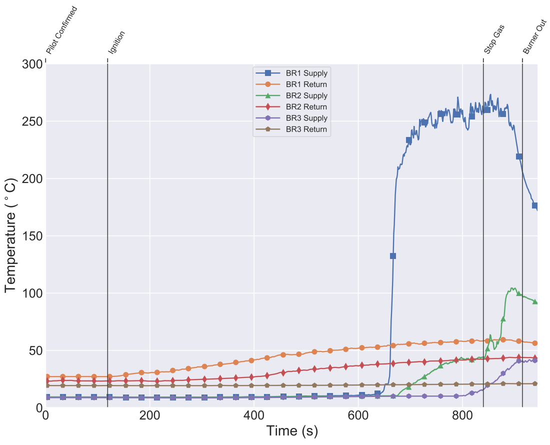

(a) Bedroom Vent Temperatures

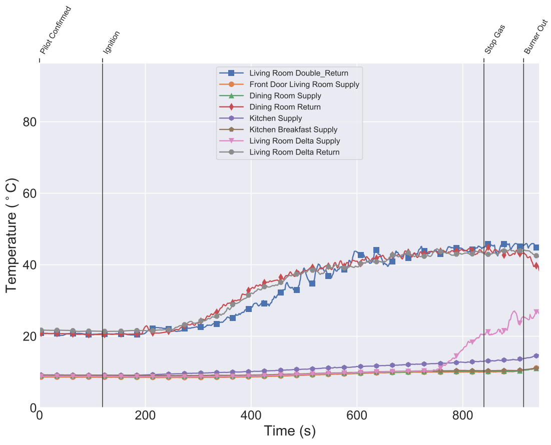

(b) First Floor Vent Temperatures

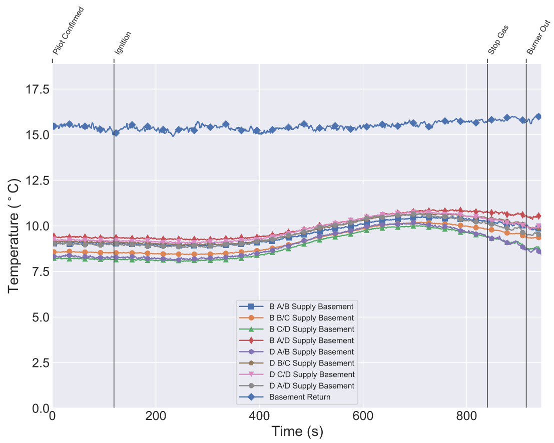

(c) Basement Vent Temperatures

Figure 5.17: Experiment 3 Vent Temperatures

The temperature and velocity within the supply duct are provided in Figure 5.18. As with the vent temperatures, the data recorded for the supply duct riser is similar to that observed in Experiment 2.

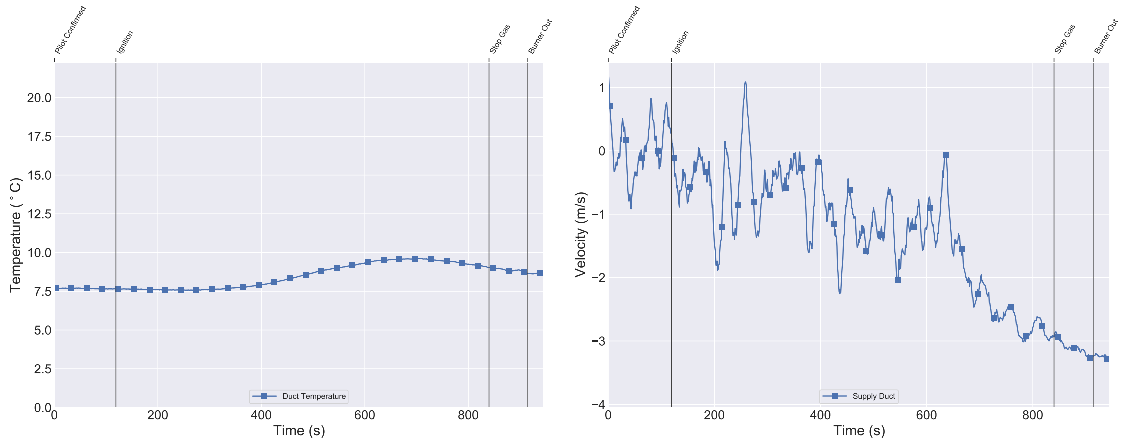

(a) Supply Duct Temperature

(b) Supply Duct Velocity

Figure 5.18: Experiment 3 Supply Duct Temperature and Velocity

5.1.4 Experiment 4

Gas Burner Experiment 4 was performed with the HVAC system set to heat. The flow rate of propane to the gas burner was controlled so that a heat release rate of approximately 121 kW was produced. The door to bedroom 1 was open as well as the staircase door to the basement. All exterior vents to the structure were closed. For this experiment, gas concentration measurements on the first floor were taken in bedroom 1, bedroom 2, bedroom 3. Experiment 4 was performed to develop a baseline for how heat and fire gases were transported within the HVAC ductwork with the system turned to heat. The events of interest for Experiment 4 are presented in Table 5.4.

Table 5.4: Experiment 4 Events

Event	Elapsed Time (seconds)
Pilot Confirmed	0
Ignition	93
Stop Gas	814
Burner Out	886

The time versus temperature graphs from the three bedrooms, the living room, the dining room and the kitchen are provided in Figure 5.19. Temperatures in bedroom 1 increased approximately 225 °C at the ceiling and approximately 50 °C at the 0.3 m height while the gas burner was on. Temperatures in bedroom 2 increased approximately 95 °C at the ceiling and approximately 20 °C at the 0.3 m height while the gas burner was on. Temperatures in bedroom 3 increased approximately 15 °C at the ceiling and had minor changes at the 0.3 m height while the gas burner was on. Temperatures in the living room increased approximately 95 °C at the ceiling and approximately 20 °C at the 0.3 m height while the gas burner was on. Temperatures in the dining room increased approximately 70 °C at the ceiling and approximately 15 °C at the 0.3 m height. Temperatures in the kitchen increased approximately 60 °C at the ceiling and 15 °C at the 0.3 m height while the gas burner was on.

Temperatures and the rates at which temperatures changed in the rooms on the first floor did not show any significant difference to the data collected in Experiment 1. By comparison of Experiment 4 to Experiment 1, setting the HVAC system to heat did not appear to have an effect on temperatures. This conclusion makes sense because the heat setting on HVAC is designed to increase the temperature in the

structure to a set point at the thermostat and will then turn the system off once that temperature is achieved. The thermostat in this structure was located in the hallway that connected all three bedrooms to the living room. With the fire being in bedroom 1 with the door open, it would not take long for the heat to travel from bedroom 1 to the thermostat. Once the temperature at the thermostat reached its designated heating temperature, the HVAC system then returned to a passive system.

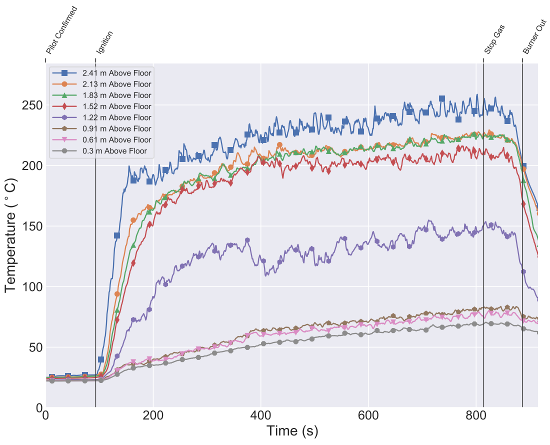

(a) Bedroom 1 Temperatures

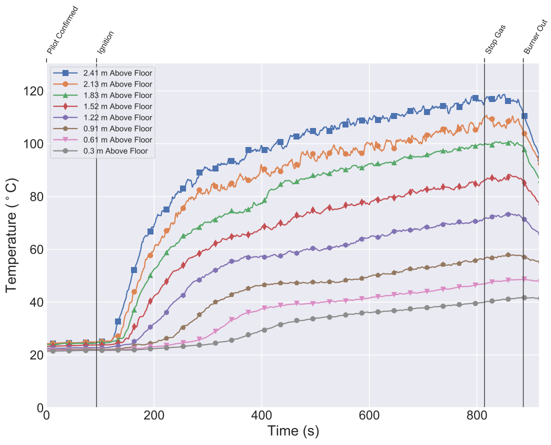

(b) Bedroom 2 Temperatures

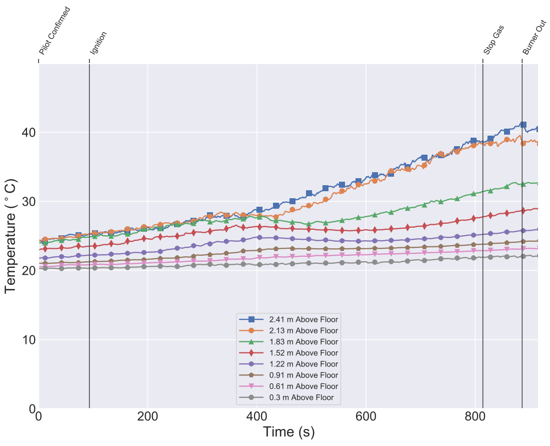

(c) Bedroom 3 Temperatures

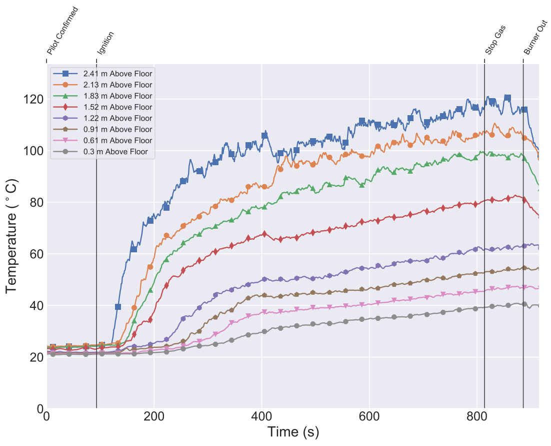

(d) Living Room Temperatures

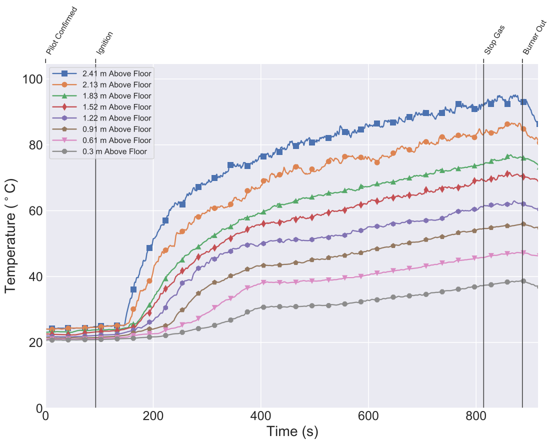

(e) Dining Room Temperatures

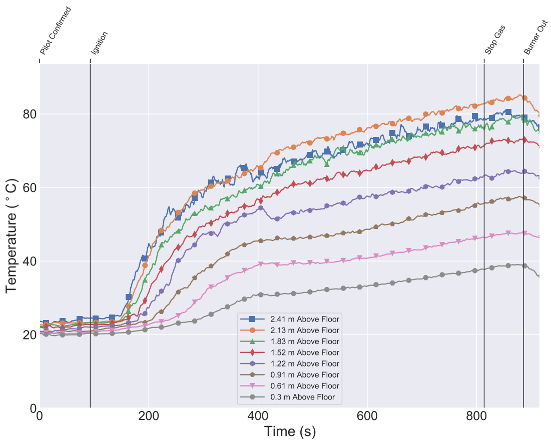

(f) Kitchen Temperatures

Figure 5.19: Experiment 4 First Floor Temperatures

The time versus temperature graphs for the four basement quadrants are provided in Figure 5.20. From the figure it can be seen that with a fire in bedroom 1, there was a notable temperature decrease in the basement of approximately 5 °C near the ceiling. One possible reason for this decrease in temperature is that as the fire grew in size and pressure, cooler air in the return system was forced from the first floor down into the basement. This theory is supported by the vent temperatures in Figure 5.23.

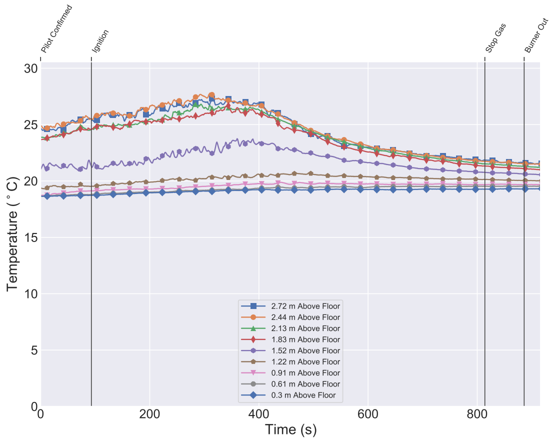

(a) Basement A Quadrant Temperatures

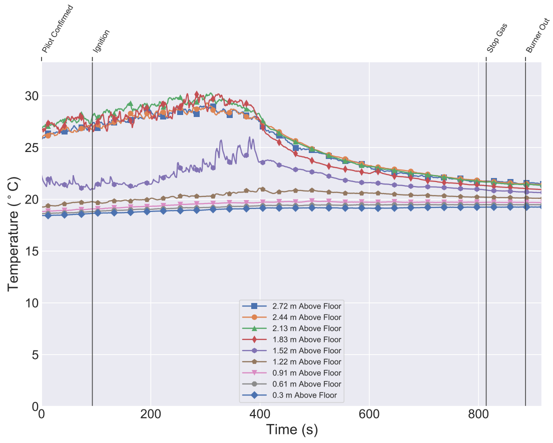

(b) Basement B Quadrant Temperatures

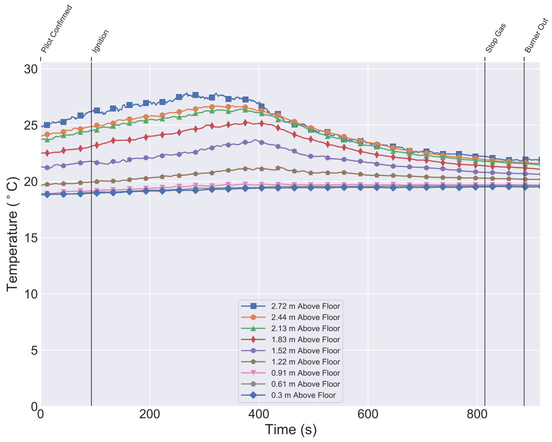

(c) Basement C Quadrant Temperatures

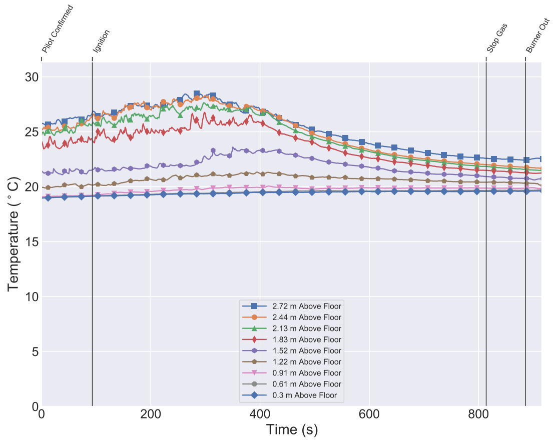

(d) Basement D Quadrant Temperatures

Figure 5.20: Experiment 4 Basement Temperatures

The pressures within each room are shown in Figure 5.21. Pressure in bedroom 1 at 2.13 m above the floor exceeded the ambient pressure by approximately 4 Pa while the burner was on. At 1.2 m above the floor pressure remained approximately equal to the ambient. At 0.3 m above the floor pressure was approximately 2 Pa below the ambient with a small positive spike following ignition. Pressures in bedrooms 2 and 3 fluctuated between 5 Pa above and below the ambient during this experiment. Pressures in the living room fluctuated between 2 Pa above and below the ambient. Pressures in the kitchen fluctuated widely between 15 Pa below the ambient and 5 Pa above. Pressures in the basement remained between 2 and 4 Pa below the ambient.

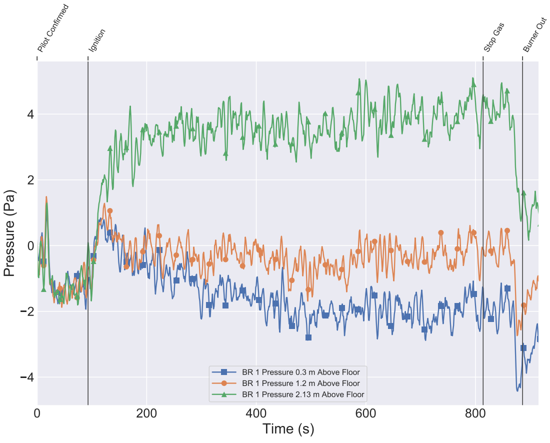

(a) Bedroom 1 Pressures

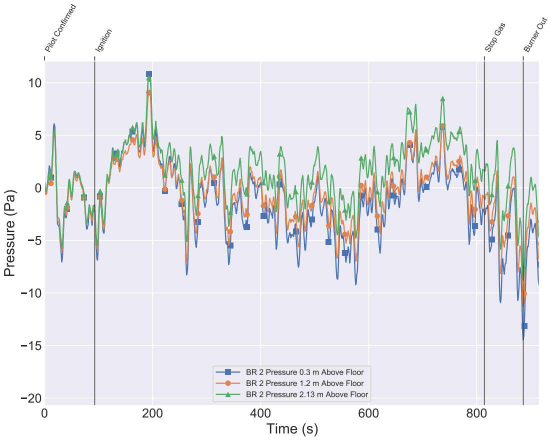

(b) Bedroom 2 Pressures

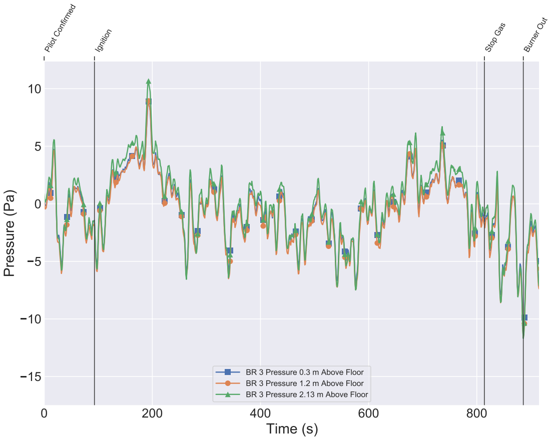

(c) Bedroom 3 Pressures

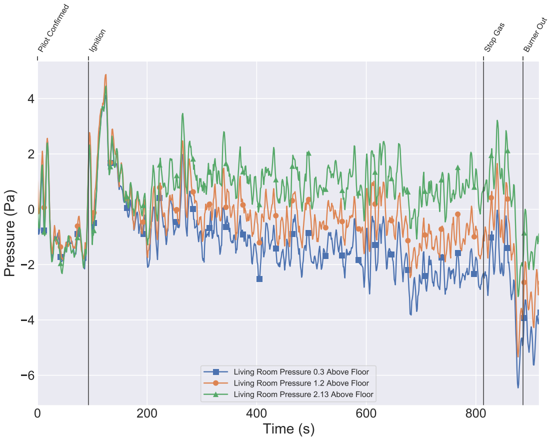

(d) Living Room Pressures

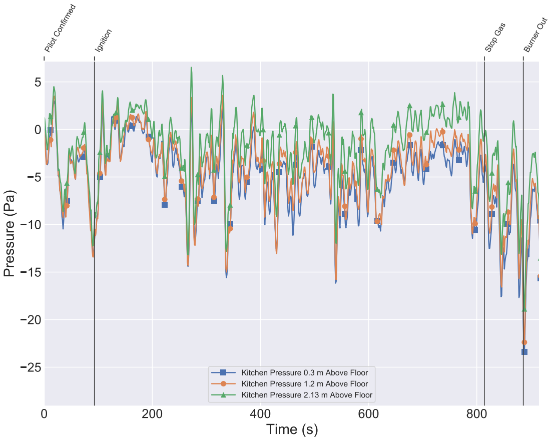

(e) Kitchen Pressures

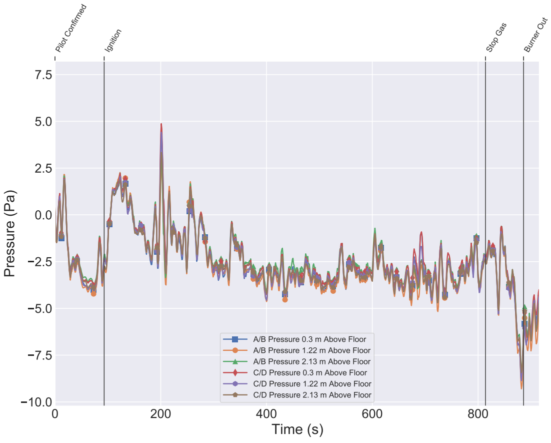

(f) Basement Pressures

Figure 5.21: Experiment 4 Pressures

The concentrations of oxygen and carbon dioxide are provided for the three bedrooms and the basement in Figure 5.22. Water vapor concentrations are included for bedroom 2, bedroom 3 and the basement. Gas concentrations in bedrooms 1 and 2 followed a similar trend with oxygen concentrations dropping to around 18% while the burner was on. Carbon dioxide concentrations in bedrooms 1 and 2 reached approximately 2%. Water vapor concentrations in bedroom 2 increased in an exponential manner up to 20% while the burner was on. Bedroom 3 displays no notable change in both oxygen and carbon dioxide concentrations prior to the gas burner going out. Water vapor concentrations in bedroom 3 showed no notable change as well. Gas and water vapor concentrations in the basement did not show any notable changes while the gas burner was on.

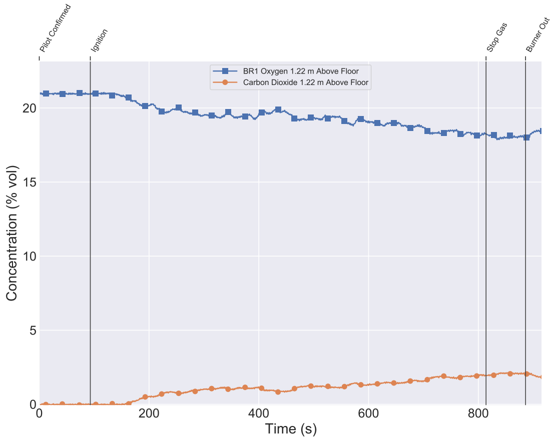

(a) Bedroom 1 Gases

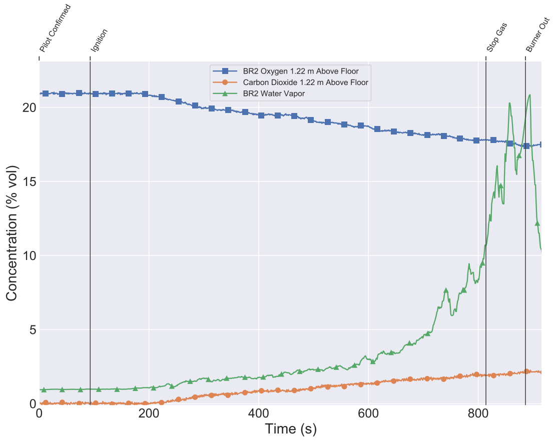

(b) Bedroom 2 Gases

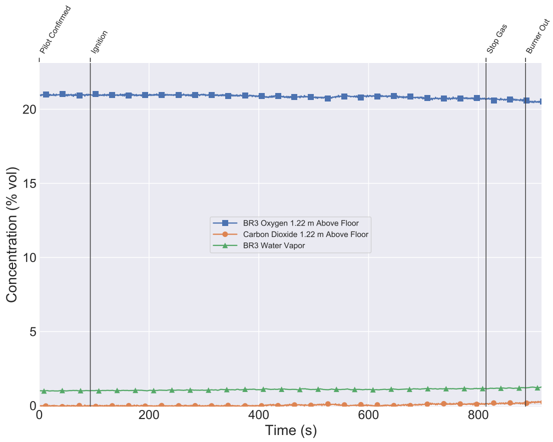

(c) Bedroom 3 Gases

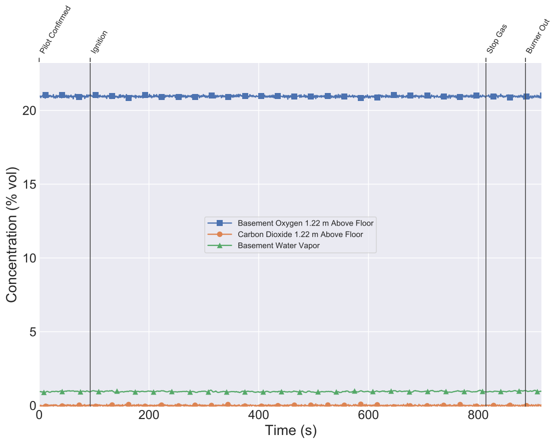

(d) Basement Gases

Figure 5.22: Experiment 4 Gases

Figure 5.23 shows the temperatures of the supply and return vents in the structure. From Figure 5.23(a) it can be seen that the supply vent temperatures in all three bedrooms are held relatively constant below 50 °C for approximately 300 seconds following ignition. The rapid temperature spike at the bedroom 1 vent displays the moment that the pressure in the fire room exceeded the pressure in the bedroom 1 branch of the supply duct. Similarly in bedroom 2 it can be seen

that shortly after the spike in bedroom 1, the pressure in bedroom 2 also exceeded that in the supply duct. The steady temperature increase in bedroom 3 following these spikes shows that the hot gases are being pushed through the supply duct in the opposite direction that is intended, similar to what was seen in Experiment 2. Return vent temperatures trended with the temperatures at that height in each bedroom.

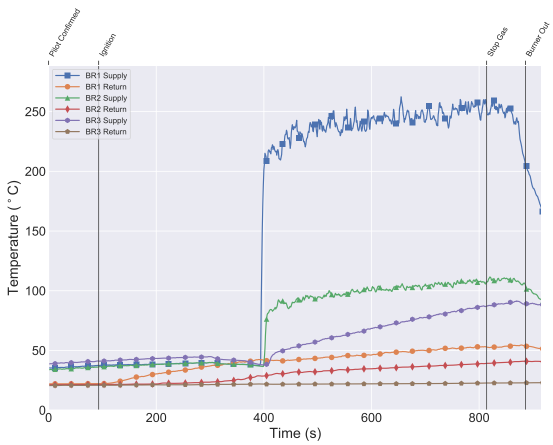

(a) Bedroom Vent Temperatures

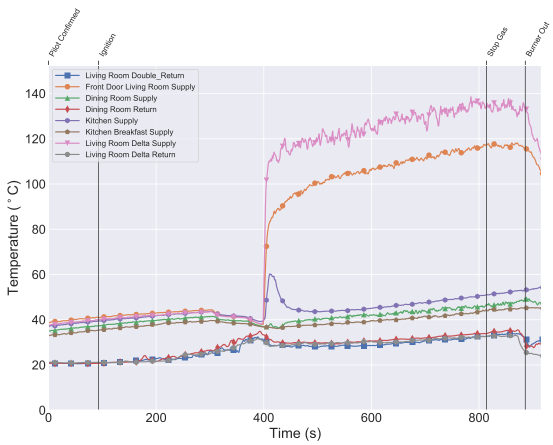

(b) First Floor Vent Temperatures

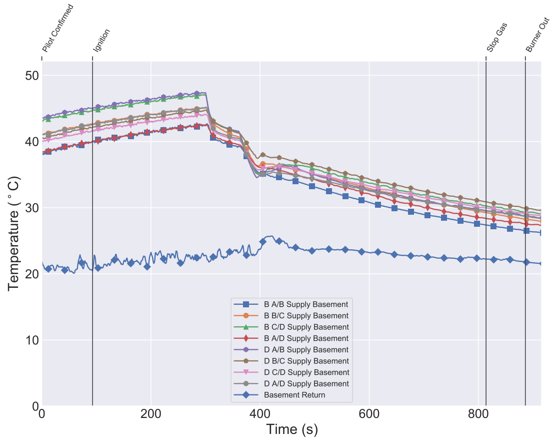

(c) Basement Vent Temperatures

Figure 5.23: Experiment 4 Vent Temperatures

Figure 5.23(b) shows the vent temperatures for the remainder of the first floor. Similar to what was observed in the bedrooms, the supply vent temperatures all stay steady at a constant temperature for a few hundred seconds following ignition. Comparison of the living room supply vents show that the supply vent closer to bedroom 1, the delta living room supply, increases to a higher temperature than the front door supply, but both spike at the same time. The kitchen and dining room supply vents appear to maintain a positive pressure within the ducts, thus maintaining a temperature lower than the ambient temperature in the room around it. The return vents for the remainder of the first floor appear to follow the temperature trends of the thermalcouple probes near the floor in their respective rooms.

Figure 5.23(c) shows the basement vent temperatures. The basement supply vents follow an increasing trend for the first 200 seconds following ignition but then display a negative trend for the remainder of the experiment. A possible reason for this decrease is that at approximately 200 seconds following ignition the thermostat reached its designated shut off temperature for heating. If the system shut down at that time, the basement temperature was still well below the temperature of the air in the supply ducts. Once the HVAC system shut off, the cooler air in the basement was able to gradually dissipate the heat that was still in the basement ductwork. The basement return vent appeared to show some unsteady heat flow possibly induced by heat being pulled or pushed through the return ductwork. While the temperature never increased by more than a few degrees, the unsteady nature of the measurement indicates turbulent mixing of warm and cool air.

The temperature and velocity within the supply duct are provided in Fig-

ure 5.24. The temperature in the supply duct increases in a similar manner to the basement supply vents and then has a sudden decrease approximately 200 seconds after ignition. This data supports the theory that the HVAC system shut off at a certain temperature, thus allowing cooler unconditioned air in the return vent to transfer into the supply duct and equalize temperature. Temperature in the supply duct riser decreased sharply before gradually increasing again as hot gases from the first floor were able to penetrate the first floor supply duct network. The velocity within the supply duct appears to show a continuous airflow from the first floor to the basement with a sharp increase in downward flow at the same time that the temperature within the duct began to slowly increase again.

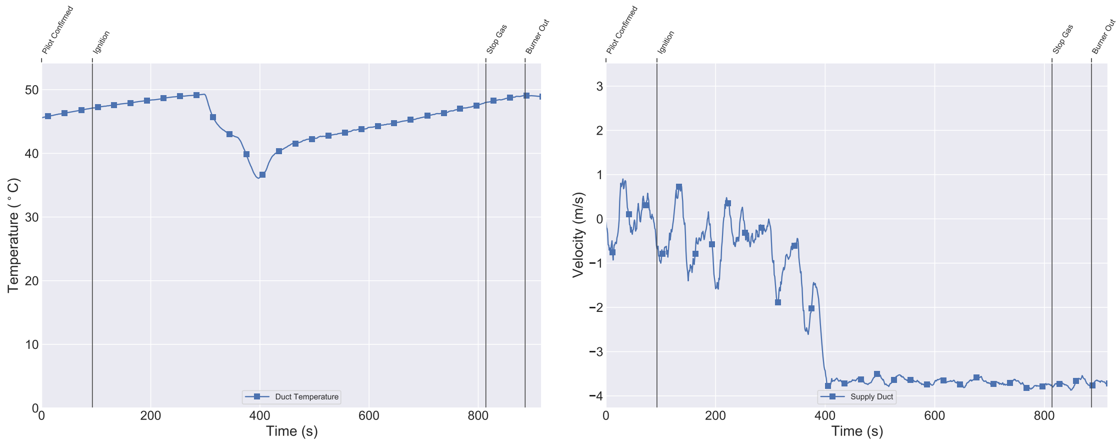

(a) Supply Duct Temperature

(b) Supply Duct Velocity

Figure 5.24: Experiment 4 Supply Duct Temperature and Velocity

5.1.5 Experiment 5

Gas Burner Experiment 5 was performed with the HVAC system set to A/C. The flow rate of propane to the gas burner was controlled so that a heat release rate

of approximately 121 kW was produced. The door to bedroom 1 was open as well as the staircase door to the basement. All exterior vents to the structure were closed. For this experiment, gas concentration measurements on the first floor were taken in bedroom 1, bedroom 2, bedroom 3. Experiment 5 was performed as another duplicate to Experiments 2 and 3 to see how heat and fire gases were transported within the HVAC ductwork with the system turned to A/C. The events of interest for Experiment 5 are presented in Table 5.5.

Table 5.5: Experiment 5 Events

Event	Elapsed Time (seconds)
Pilot Confirmed	0
Ignition	77
Stop Gas	799
Burner Out	864

The time versus temperature graphs from the three bedrooms, the living room, the dining room and the kitchen are provided in Figure 5.25. All temperatures on the first floor followed the trends that were established in Experiments 2 and 3.

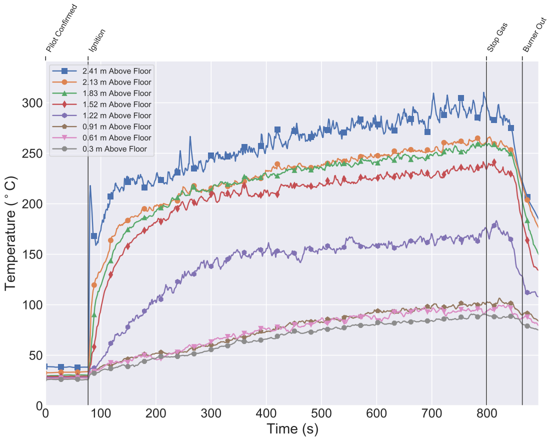

(a) Bedroom 1 Temperatures

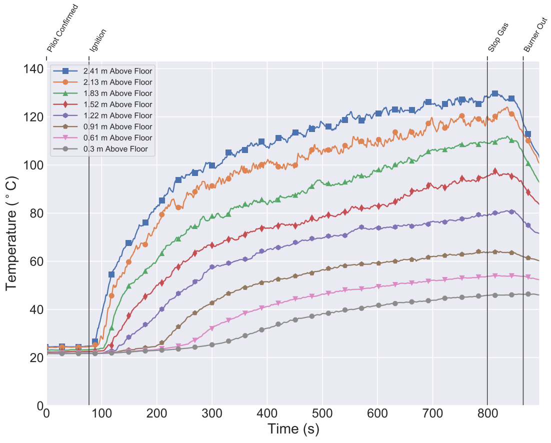

(b) Bedroom 2 Temperatures

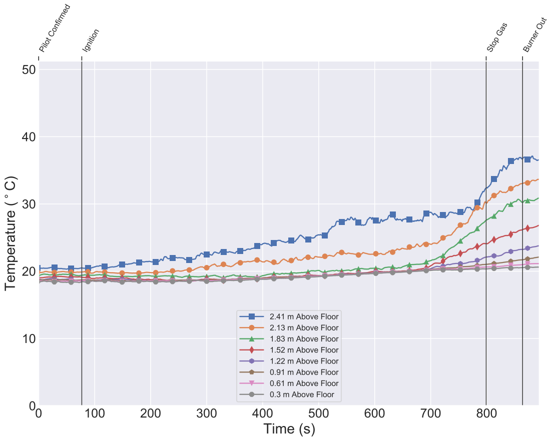

(c) Bedroom 3 Temperatures

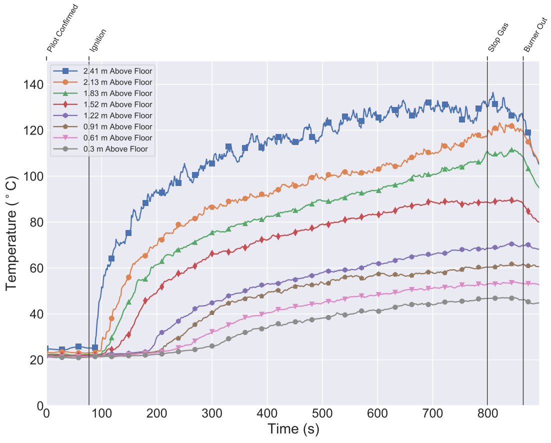

(d) Living Room Temperatures

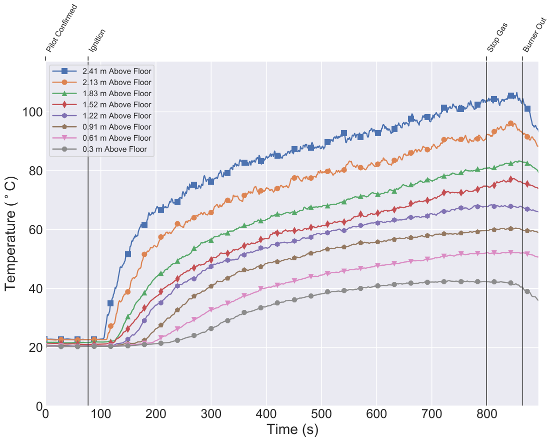

(e) Dining Room Temperatures

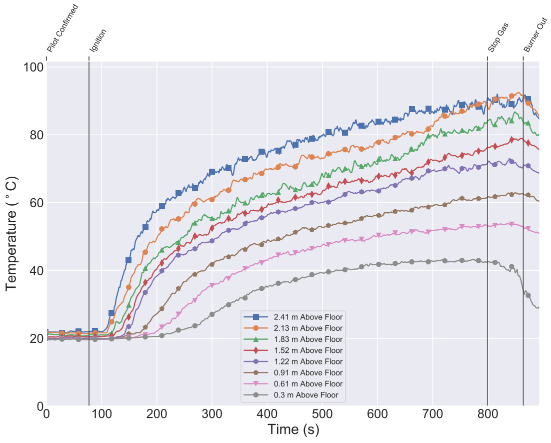

(f) Kitchen Temperatures

Figure 5.25: Experiment 5 First Floor Temperatures

The time versus temperature graphs for the four basement quadrants are provided in Figure 5.26. As was the case with Experiments 2 and 3, there were no changes to the temperatures in the basement.

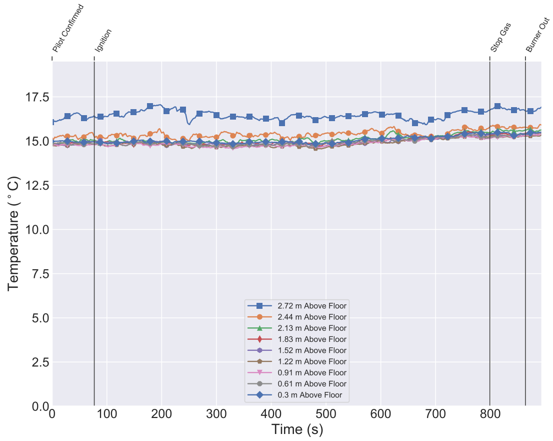

(a) Basement A Quadrant Temperatures

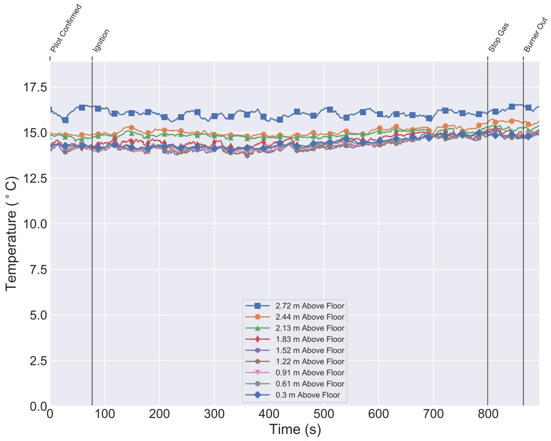

(b) Basement B Quadrant Temperatures

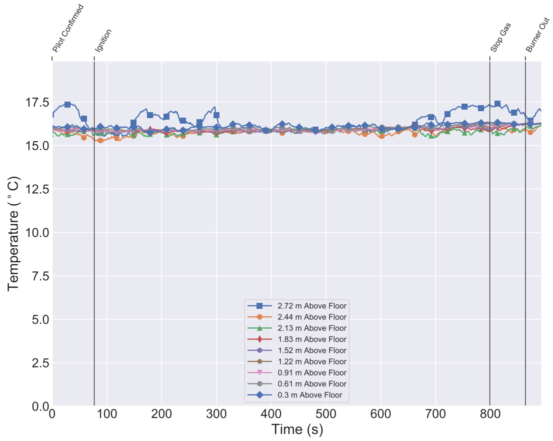

(c) Basement C Quadrant Temperatures

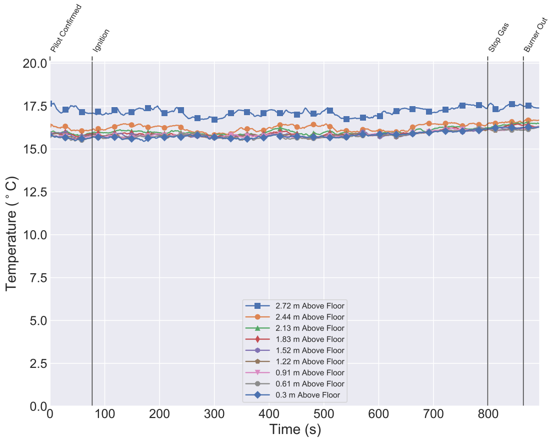

(d) Basement D Quadrant Temperatures

Figure 5.26: Experiment 5 Basement Temperatures

The pressures within each room are shown in Figure 5.27. Pressures appeared to follow similar trends established in Experiment 2 with the exception of all rooms recording a spike greater than 15 Pa following ignition.

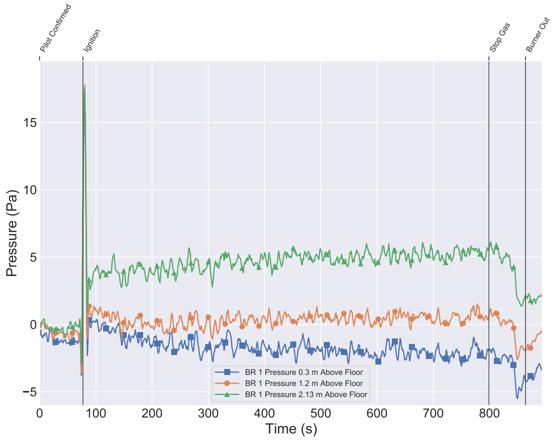

(a) Bedroom 1 Pressures

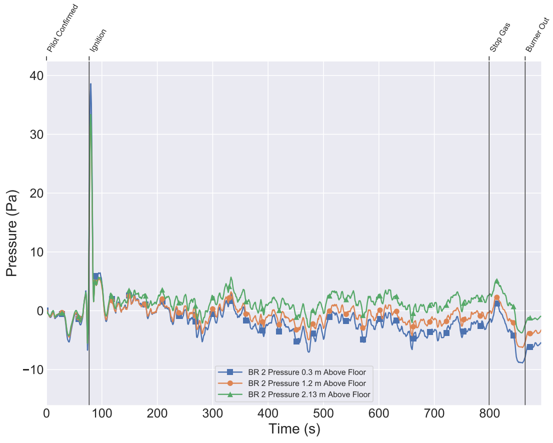

(b) Bedroom 2 Pressures

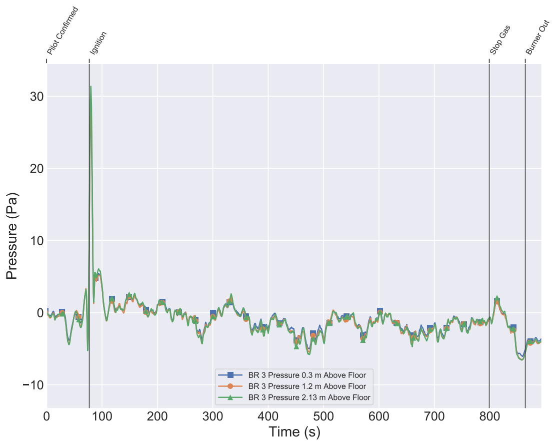

(c) Bedroom 3 Pressures

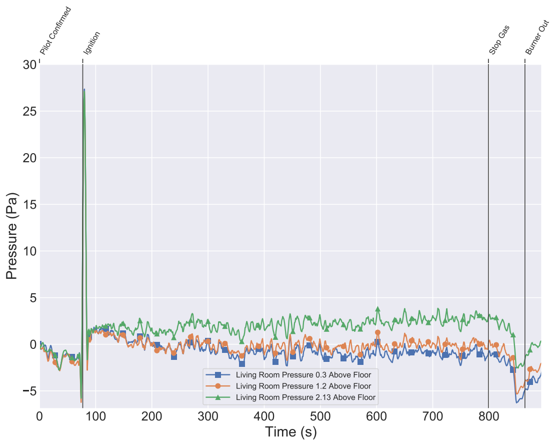

(d) Living Room Pressures

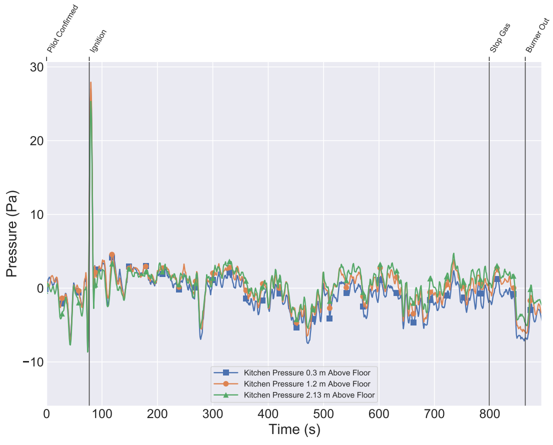

(e) Kitchen Pressures

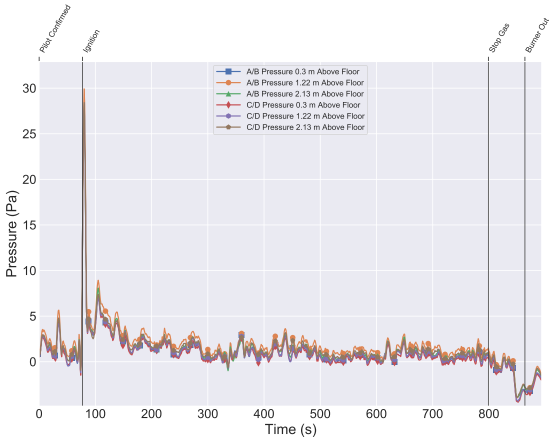

(f) Basement Pressures

Figure 5.27: Experiment 5 Pressures

The concentrations of oxygen and carbon dioxide are provided for the three bedrooms and the basement in Figure 5.28. Water vapor concentrations are included for bedroom 2, bedroom 3 and the basement. Gas and water vapor concentrations recorded in Experiment 5 followed the same trends that were discussed in Experiments 2 and 3.

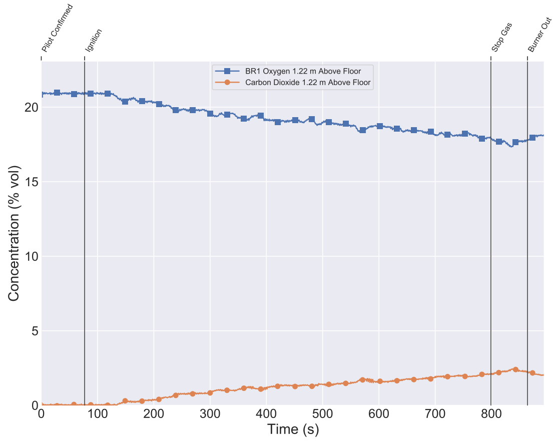

(a) Bedroom 1 Gases

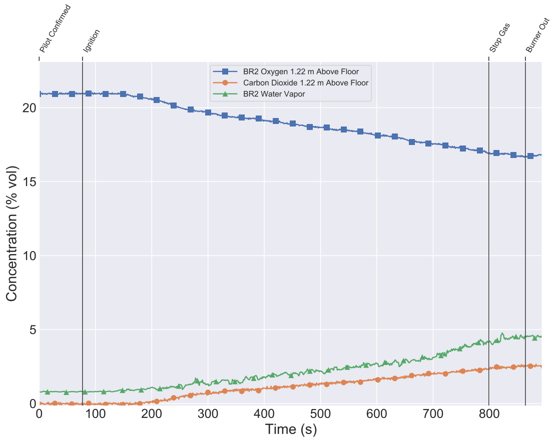

(b) Bedroom 2 Gases

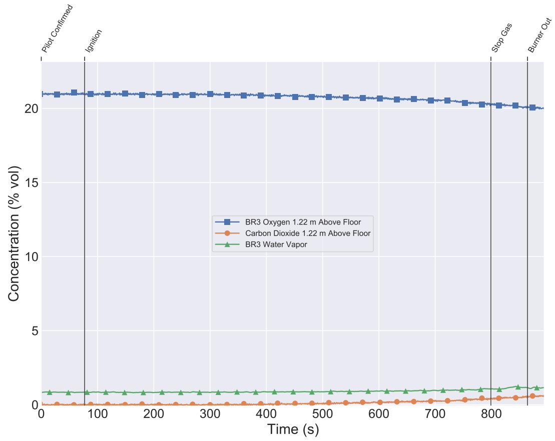

(c) Bedroom 3 Gases

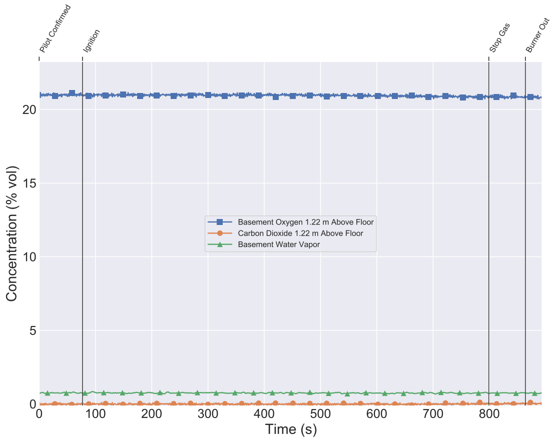

(d) Basement Gases

Figure 5.28: Experiment 5 Gases

Figure 5.29 shows the temperatures of the supply and return vents in the struc-

ture. As was the case with Experiment 3, the vent temperature data recorded for Experiment 5 is similar to that reported in Experiment 2 throughout the structure.

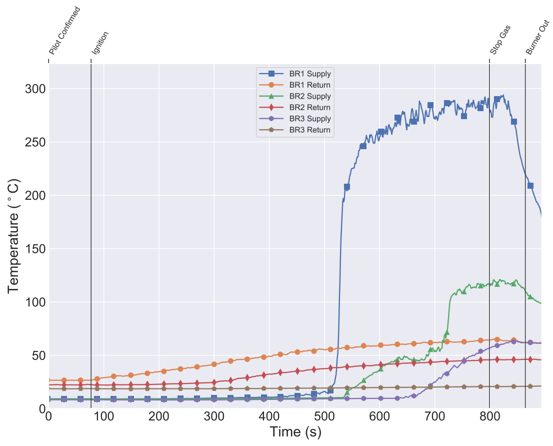

(a) Bedroom Vent Temperatures

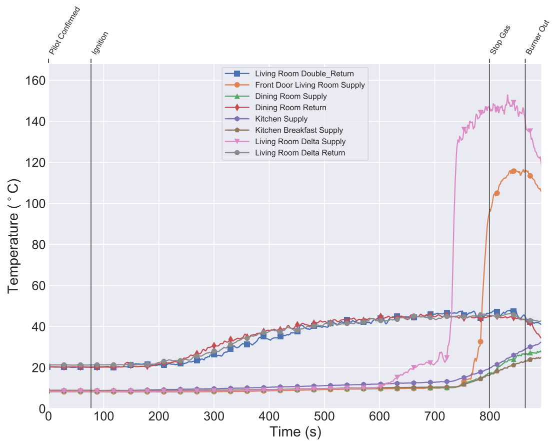

(b) First Floor Vent Temperatures

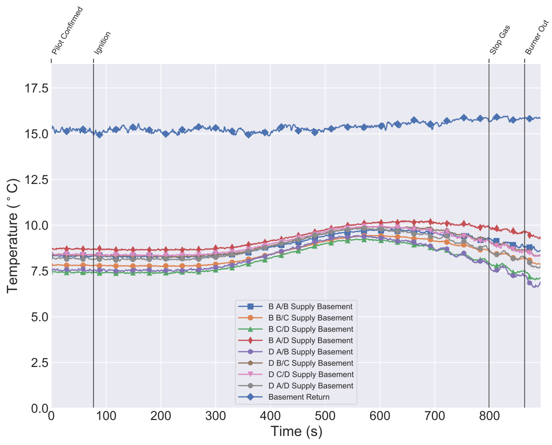

(c) Basement Vent Temperatures

Figure 5.29: Experiment 5 Vent Temperatures

The temperature and velocity within the supply duct are provided in Figure 5.30. The supply duct temperature and velocity follow the trends of Experiments 2 and 3.

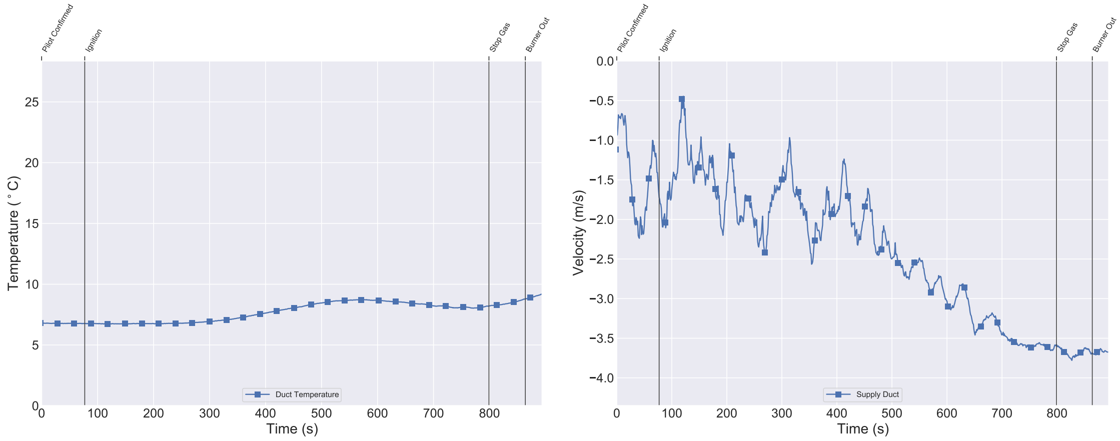

(a) Supply Duct Temperature

(b) Supply Duct Velocity

Figure 5.30: Experiment 5 Supply Duct Temperature and Velocity

Analysis of Experiment 5 provided repetative data that was similar to what was recorded in Experiments 2 and 3. Experiment 5 provided reassurance and successfully completed its objective of supporting the data and trends identified in the prior experiments utilizing the same setup.

5.1.6 Experiment 6

Gas Burner Experiment 6 was performed with the HVAC system set to heat. The flow rate of propane to the gas burner was controlled so that a heat release rate of approximately 121 kW was produced. The door to bedroom 1 was open as well as the staircase door to the basement. All exterior vents to the structure were closed. For this experiment, gas concentration measurements on the first floor were taken in bedroom 1, bedroom 2, bedroom 3. Experiment 6 was performed as a duplicate to Experiment 4 to see how heat and fire gases were transported within the HVAC

ductwork with the system turned to heat. The events of interest for Experiment 6 are presented in Table 5.6.

Table 5.6: Experiment 6 Events

Event	Elapsed Time (seconds)
Pilot Confirmed	0
Ignition	103
Stop Gas	824
Burner Out	901

The time versus temperature graphs from the three bedrooms, the living room, the dining room and the kitchen are provided in Figure 5.31. The temperatures recorded in Experiment 6 show similar values and trends to those identified in Experiment 4.

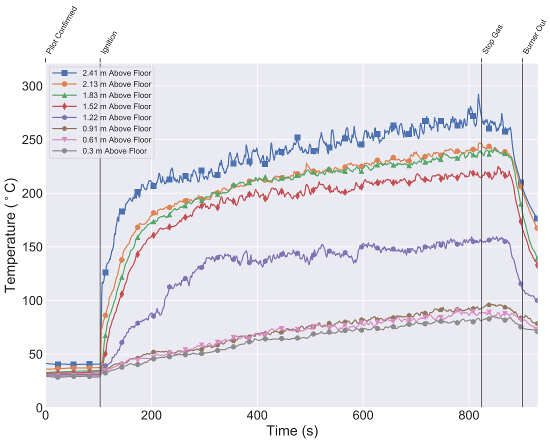

(a) Bedroom 1 Temperatures

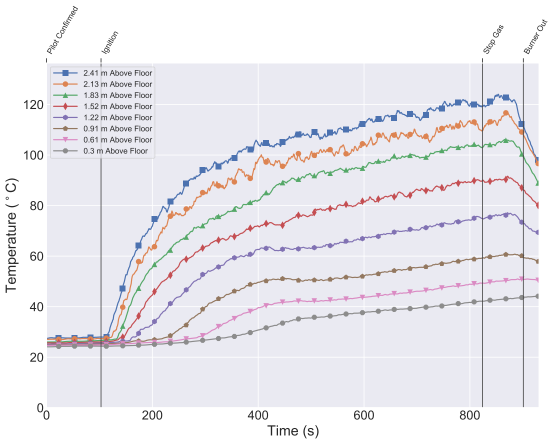

(b) Bedroom 2 Temperatures

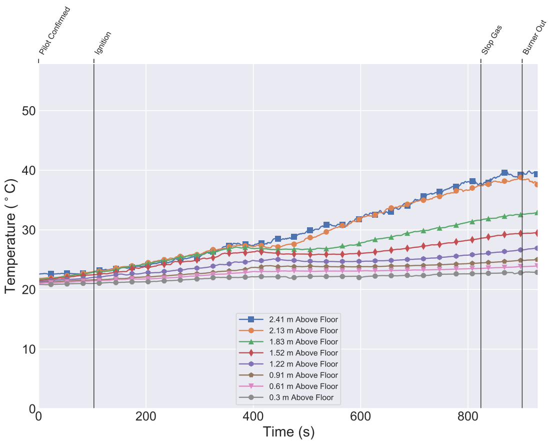

(c) Bedroom 3 Temperatures

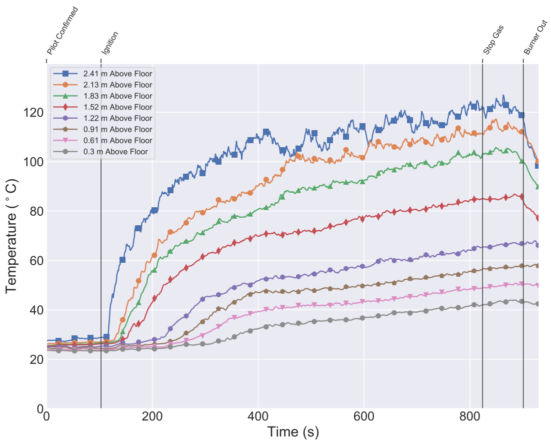

(d) Living Room Temperatures

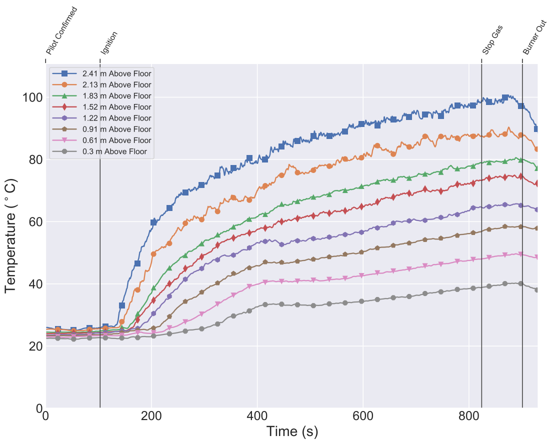

(e) Dining Room Temperatures

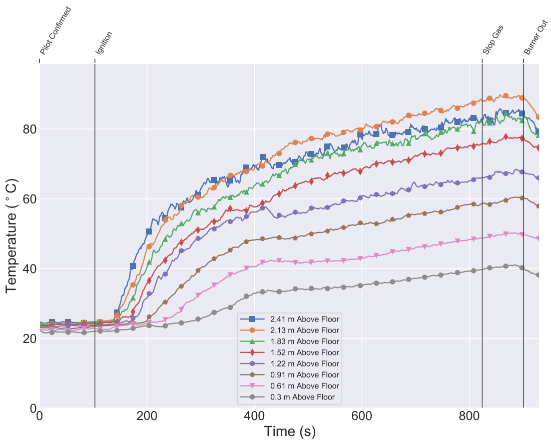

(f) Kitchen Temperatures

Figure 5.31: Experiment 6 First Floor Temperatures

The time versus temperature graphs for the four basement quadrants are provided in Figure 5.32. Comparison of the Experiment 6 to those of Experiment 4 show the same increase and decrease discussed in Experiment 4.

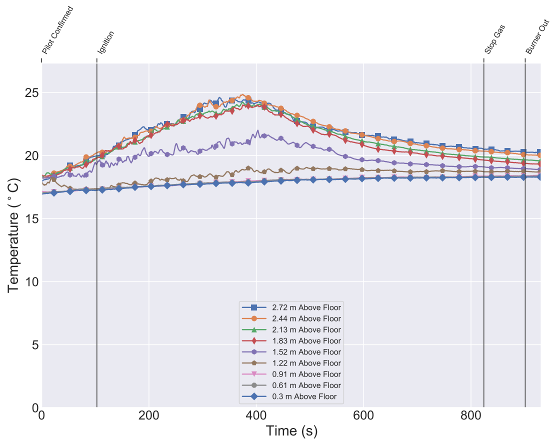

(a) Basement A Quadrant Temperatures

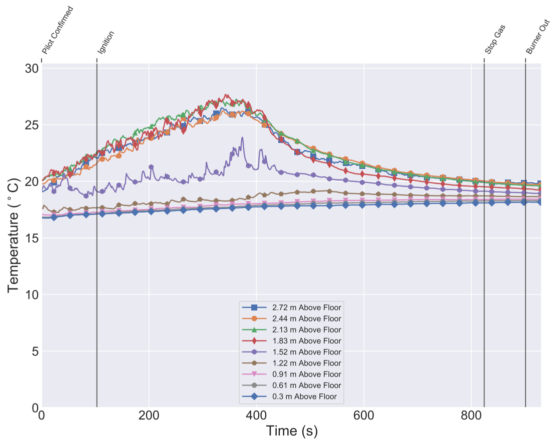

(b) Basement B Quadrant Temperatures

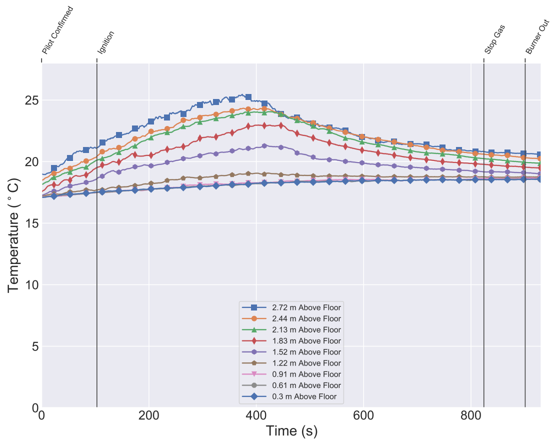

(c) Basement C Quadrant Temperatures

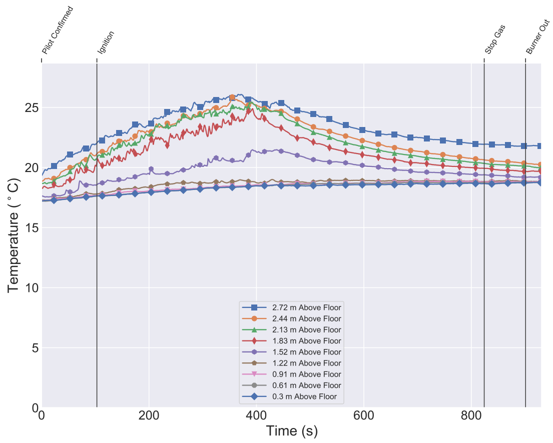

(d) Basement D Quadrant Temperatures

Figure 5.32: Experiment 6 Basement Temperatures

The pressures within each room are shown in Figure 5.33. The pressures recorded in Experiment 6 are similar to those of Experiment 4 with the exception of a pressure spike greater than 10 Pa following ignition in all rooms.

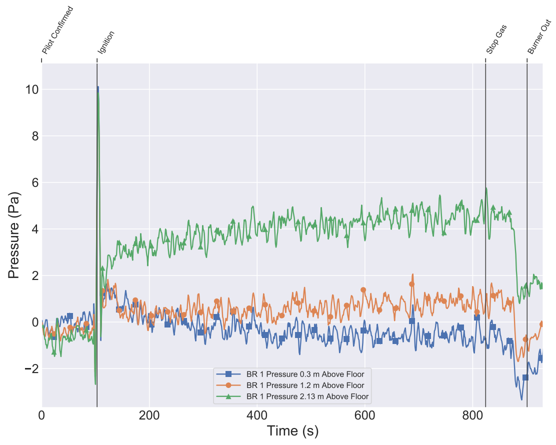

(a) Bedroom 1 Pressures

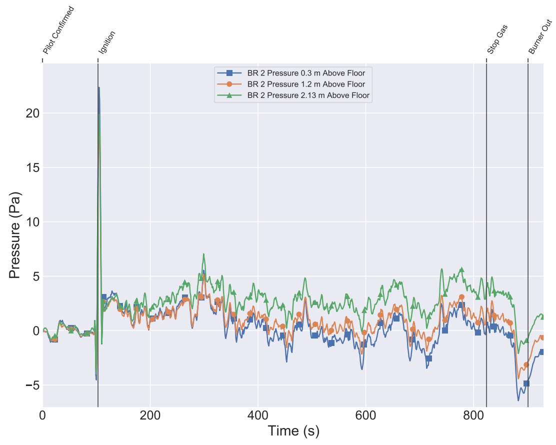

(b) Bedroom 2 Pressures

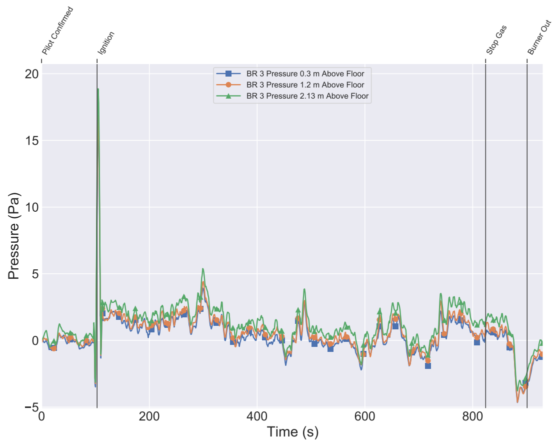

(c) Bedroom 3 Pressures

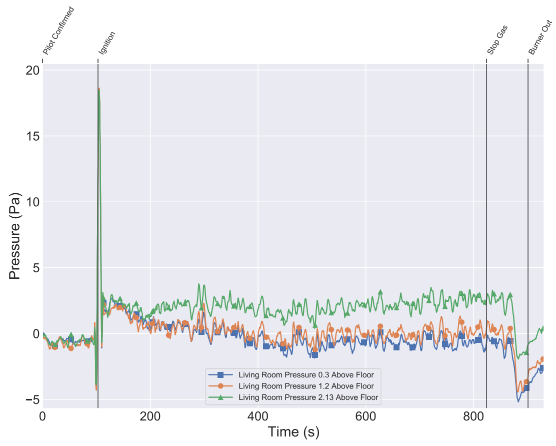

(d) Living Room Pressures

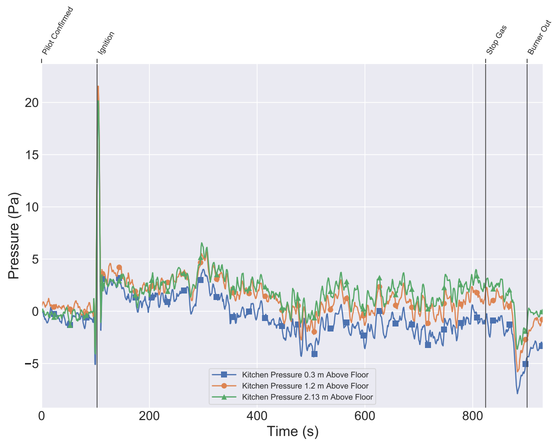

(e) Kitchen Pressures

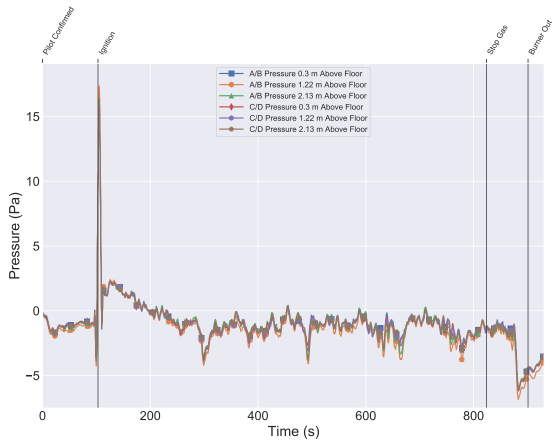

(f) Basement Pressures

Figure 5.33: Experiment 6 Pressures

The concentrations of oxygen and carbon dioxide are provided for the three bedrooms and the basement in Figure 5.34. Water vapor concentrations are included for bedroom 2, bedroom 3 and the basement. Gas concentrations in all locations show a similar trend to those reported in Experiment 4. Water vapor concentrations in bedroom 3 and the basement showed no change, similar to Experiment 4. The water vapor data collected in bedroom 2 yielded an increase of approximately 3% during this experiment, whereas in Experiment 4 the water vapor in bedroom 2 increased over five times that amount.

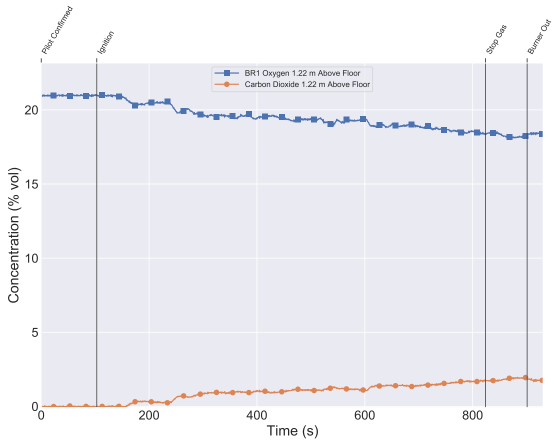

(a) Bedroom 1 Gases

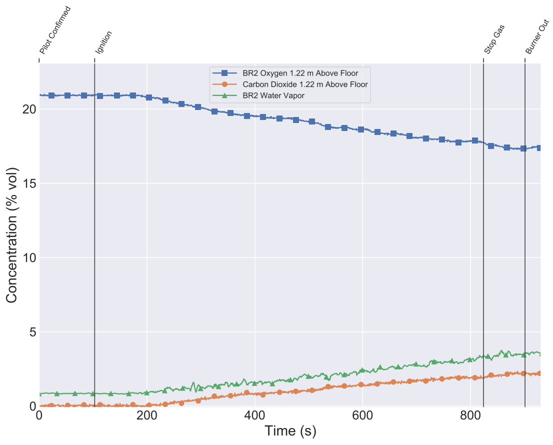

(b) Bedroom 2 Gases

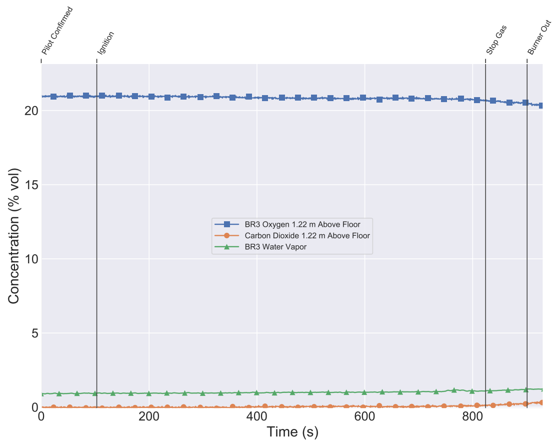

(c) Bedroom 3 Gases

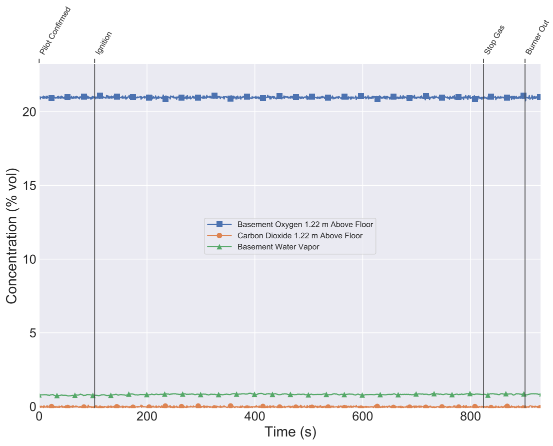

(d) Basement Gases

Figure 5.34: Experiment 6 Gases

Figure 5.35 shows the temperatures of the supply and return vents in the structure. Comparion of Experiment 6 vent data to that of Experiment 4 show nearly identical results, supporting the trends identified in Experiment 4.

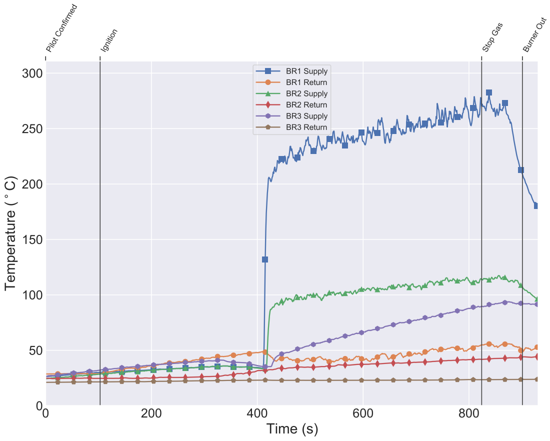

(a) Bedroom Vent Temperatures

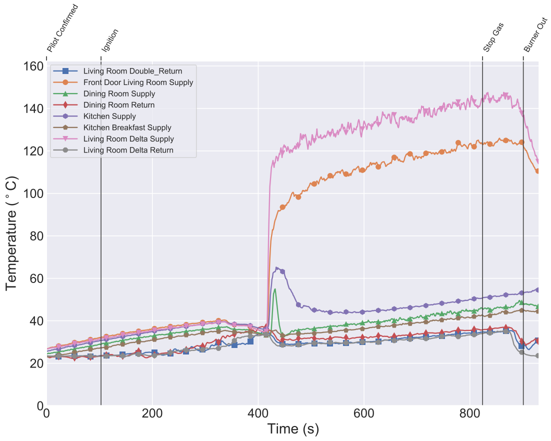

(b) First Floor Vent Temperatures

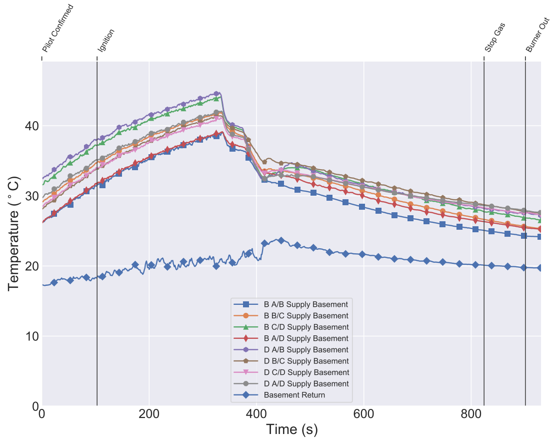

(c) Basement Vent Temperatures

Figure 5.35: Experiment 6 Vent Temperatures

The temperature and velocity within the supply duct are provided in Figure 5.36. The supply duct data collected for Experiment 6 is again nearly identical to that recorded in Experiment 4.

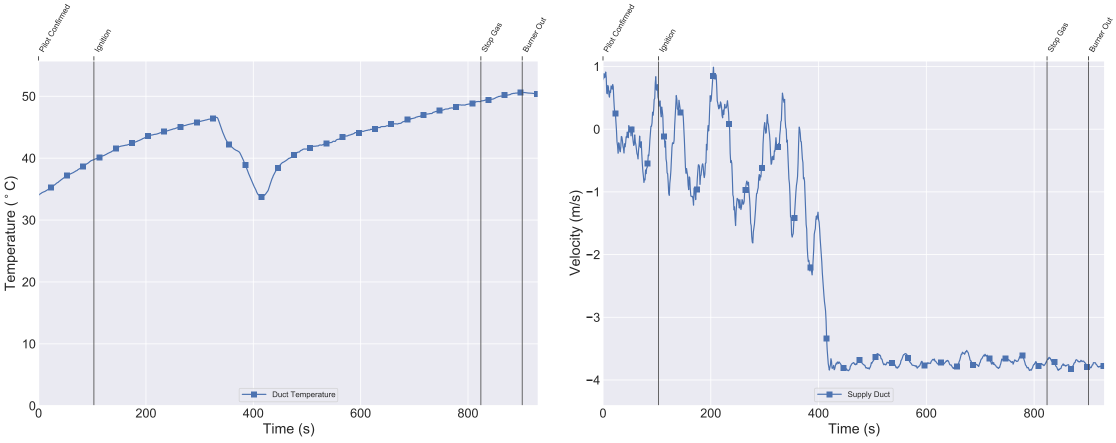

(a) Supply Duct Temperature

(b) Supply Duct Velocity

Figure 5.36: Experiment 6 Supply Duct Temperature and Velocity

The data collected in Experiment 6 supports the trends identified in Experiment 4 and provide data to support the repeatability of the experiments performed.

5.1.7 Experiment 7

Gas Burner Experiment 7 was performed with the HVAC system off. The flow rate of propane to the gas burner was controlled so that a heat release rate of approximately 121 kW was produced initially and was then increased to 302 kW shortly thereafter. The door to bedroom 1 was open as well as the staircase door to the basement. All exterior vents to the structure were closed. For this experiment, gas concentration measurements on the first floor were taken in bedroom 1, bedroom 2, bedroom 3. Experiment 7 was performed to develop a comparison to Experiment 1 for how a higher heat release rate would transport heat and fire gases within the HVAC ductwork with the system turned off. The events of interest for Experiment

7 are presented in Table 5.7.

Table 5.7: Experiment 7 Events

Event	Elapsed Time (seconds)
Pilot Confirmed	0
Ignition	112
Increased HRR	233
Stop Gas	893
Burner Out	943

The time versus temperature graphs from the three bedrooms, the living room, the dining room and the kitchen are provided in Figure 5.37. Temperatures in bedroom 1 increased approximately 600 °C at the ceiling and approximately 125 °C at the 0.3 m height while the gas burner was on. Temperatures in bedroom 2 increased approximately 150 °C at the ceiling and approximately 35 °C at the 0.3 m height while the gas burner was on. Temperatures in bedroom 3 increased approximately 30 °C at the ceiling and had minor changes at the 0.3 m height while the gas burner was on. Temperatures in the living room increased approximately 150 °C at the ceiling and approximately 35 °C at the 0.3 m height while the gas burner was on. Temperatures in the dining room increased approximately 115 °C at the ceiling and approximately 40 °C at the 0.3 m height. Temperatures in the kitchen increased approximately 100 °C at the ceiling and 40 °C at the 0.3 m height while the gas burner was on.

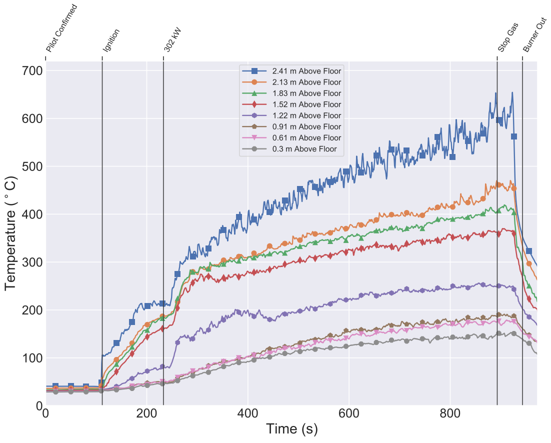

(a) Bedroom 1 Temperatures

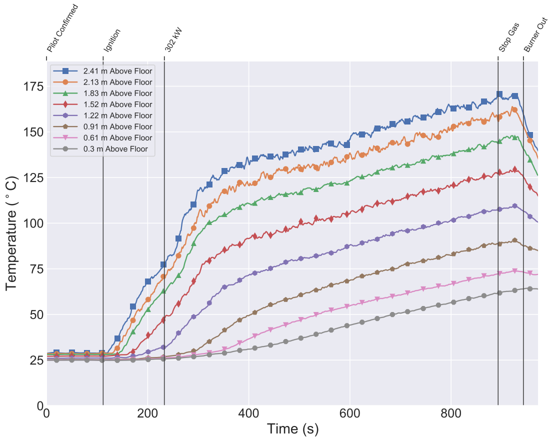

(b) Bedroom 2 Temperatures

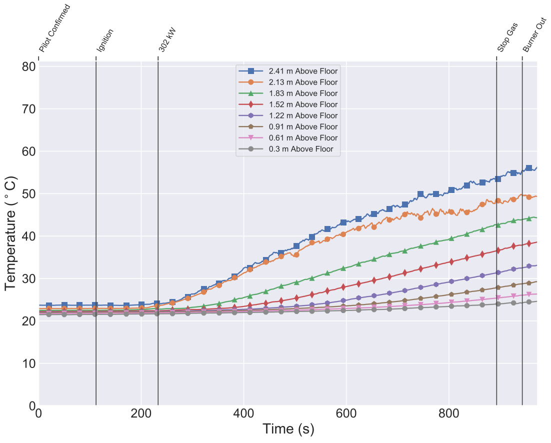

(c) Bedroom 3 Temperatures

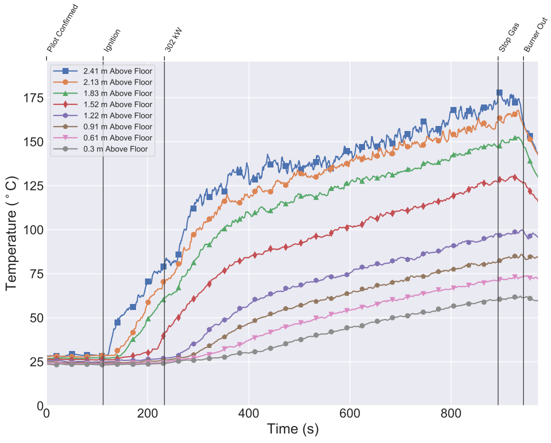

(d) Living Room Temperatures

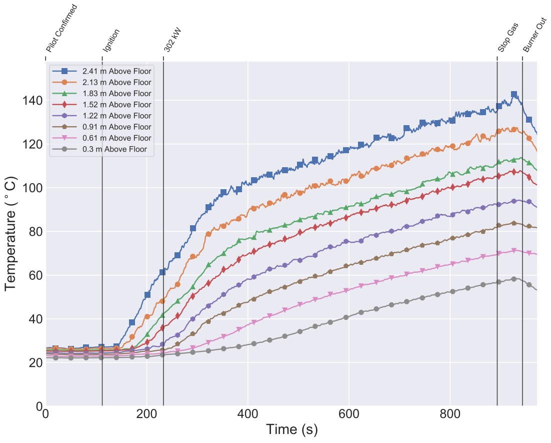

(e) Dining Room Temperatures

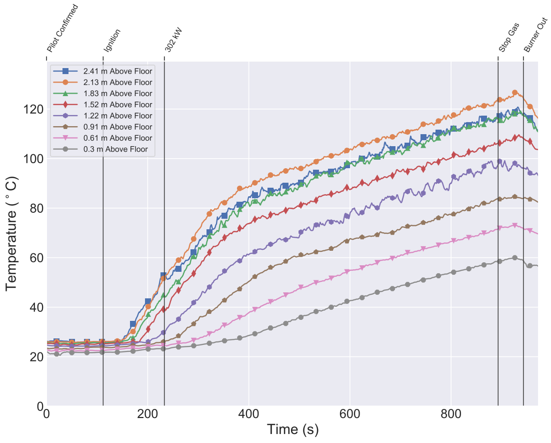

(f) Kitchen Temperatures

Figure 5.37: Experiment 7 First Floor Temperatures

The time versus temperature graphs for the four basement quadrants are provided in Figure 5.38. Similar to what has been observed in all bedroom 1 burner experiments, there was no notable change in basement temperatures.

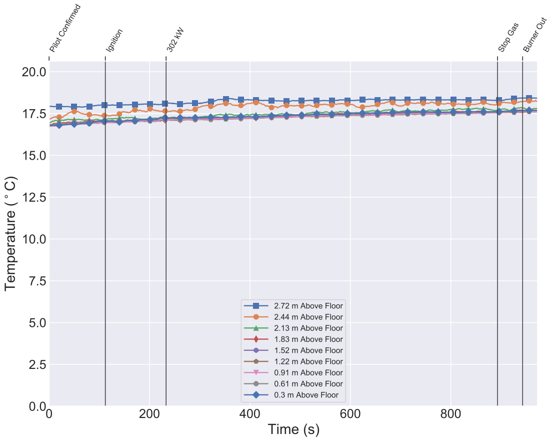

(a) Basement A Quadrant Temperatures

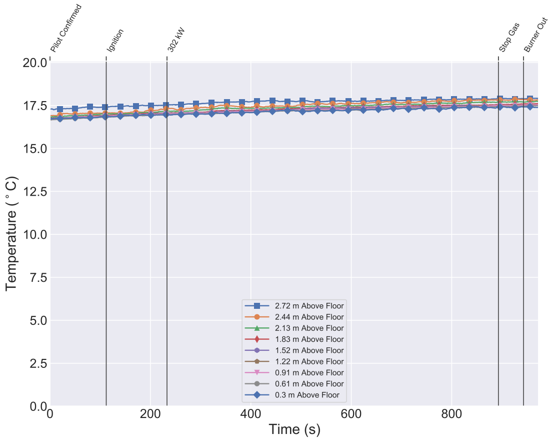

(b) Basement B Quadrant Temperatures

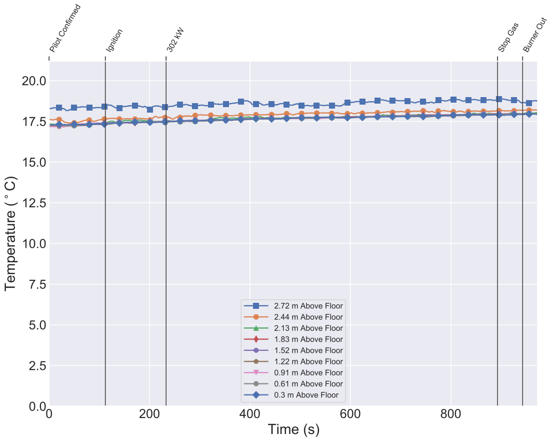

(c) Basement C Quadrant Temperatures

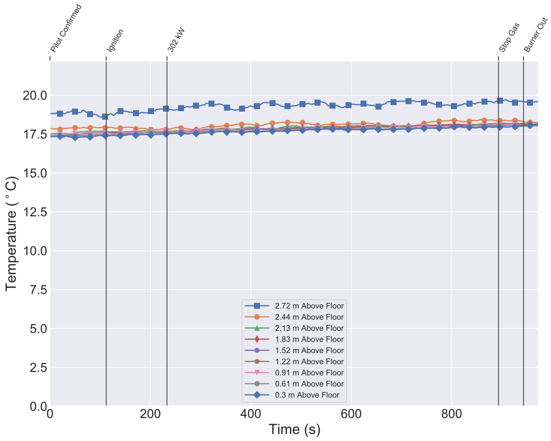

(d) Basement D Quadrant Temperatures

Figure 5.38: Experiment 7 Basement Temperatures

The pressures within each room are shown in Figure 5.39. Pressure in bedroom 1 at 2.13 m above the floor exceeded the ambient pressure by approximately 8 Pa while the burner was on. At 1.2 m above the floor pressure exceeded the ambient by

approximately 3 Pa. At 0.3 m above the floor pressure remained at approximately ambient with a small spike following ignition. Pressures in bedroom 2 followed a similar trend with the exception of the 0.3 m height which trended similar to the 1.2 m height. Pressures in bedroom 3 were similar at all height measurements with a peak of 8 Pa above ambient shortly after the HRR was increased. Pressures in bedroom 3 mostly fluctuated between 2 to 3 Pa above the ambient pressure while the gas burner was on. Pressures in the living room and kitchen were similar to those recorded in bedroom 2. Pressures in the basement remained around 0 Pa for the majority of the time that the burner was on with the exception of a positive spike of approximately 8 Pa shortly after the HRR was increased.

Comparison to Experiment 1 shows that the pressures all trended in a similar manner with greater values being achieved by the higher HRR in this Experiment.

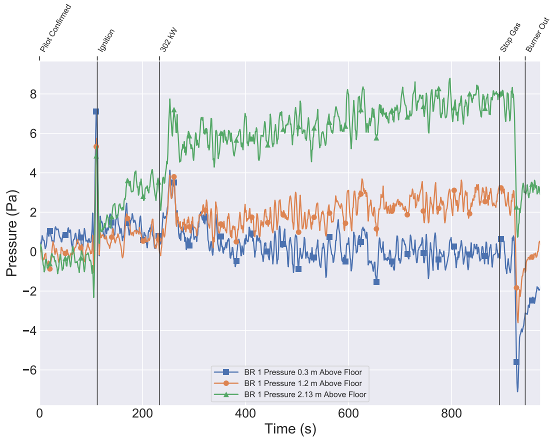

(a) Bedroom 1 Pressures

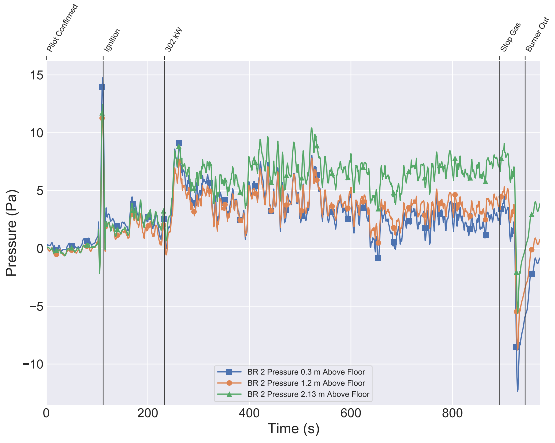

(b) Bedroom 2 Pressures

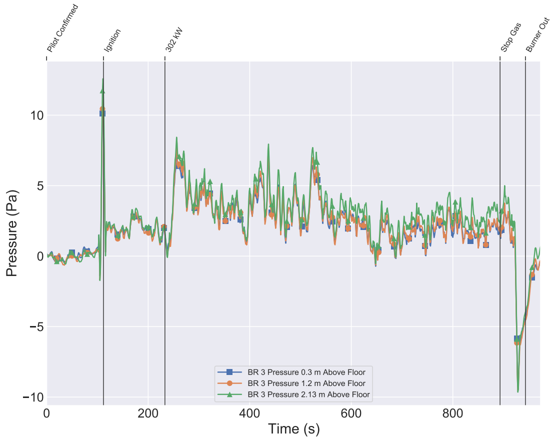

(c) Bedroom 3 Pressures

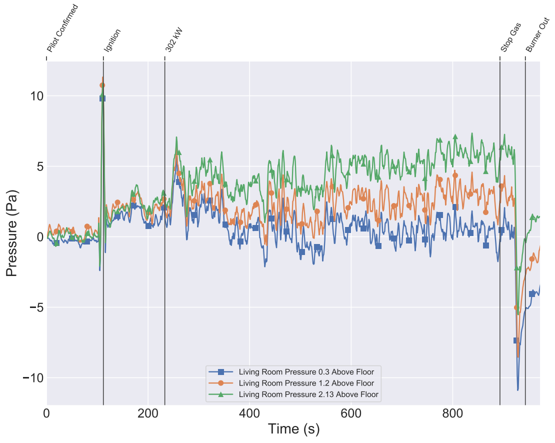

(d) Living Room Pressures

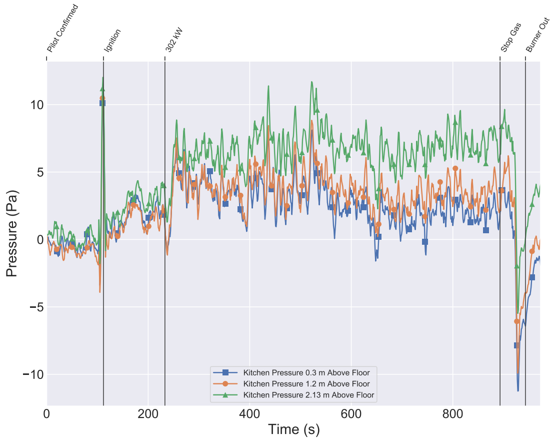

(e) Kitchen Pressures

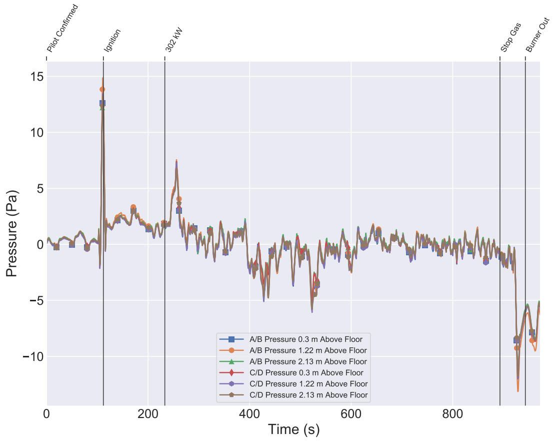

(f) Basement Pressures

Figure 5.39: Experiment 7 Pressures

The concentrations of oxygen and carbon dioxide are provided for the three bedrooms and the basement in Figure 5.40. Water vapor concentrations are included for bedroom 2, bedroom 3 and the basement. Oxygen concentrations in bedroom 1 dropped to around 15% while the burner was on. Carbon dioxide concentrations in bedroom 1 reached approximately 4%. Oxygen concentrations in bedroom 2 dropped to approximately 13% while carbon dioxide concentrations increased to approximately 5%. Water vapor concentrations in bedroom 2 increased approximately 7% while the burner was on. Oxygen concentrations in bedroom 3 dropped to approximately 17% prior to the gas burner going out. Carbon dioxide concentrations increased to approximately 2%. Water vapor concentrations in bedroom 3 increased by approximately 1% while the gas burner was on. Gas and water vapor concentrations in the basement did not show any notable changes while the gas burner was on.

Comparison of Experiment 7 gas and water vapor concentrations shows that over a similar time frame, there is a greater change in concentrations due to a larger fire, which is expected as a result of more propane being combusted.

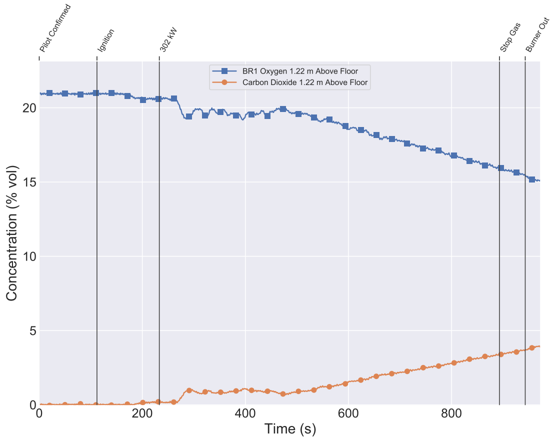

(a) Bedroom 1 Gases

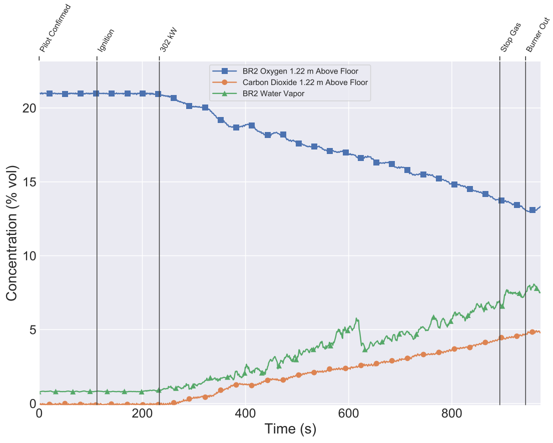

(b) Bedroom 2 Gases

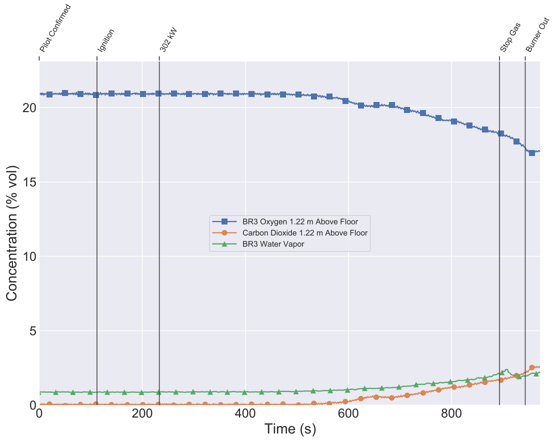

(c) Bedroom 3 Gases

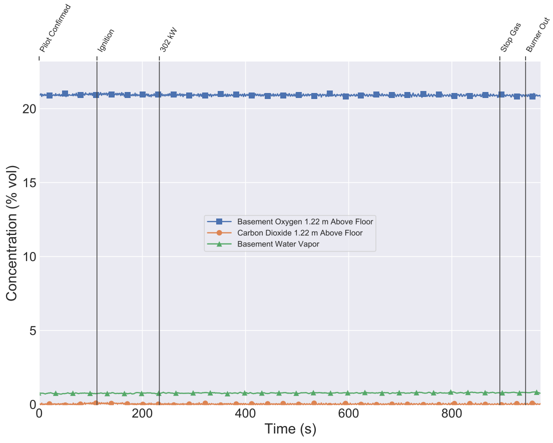

(d) Basement Gases

Figure 5.40: Experiment 7 Gases

Figure 5.41 shows the temperatures of the supply and return vents in the structure. The temperatures recorded in Experiment 7 yield similar trends to those observed in Experiment 1 but with greater magnitudes of temperature change on the first floor. The basement vent temperatures showed only a slight increase over the course of the experiment, similar to what was seen in Experiment 1.

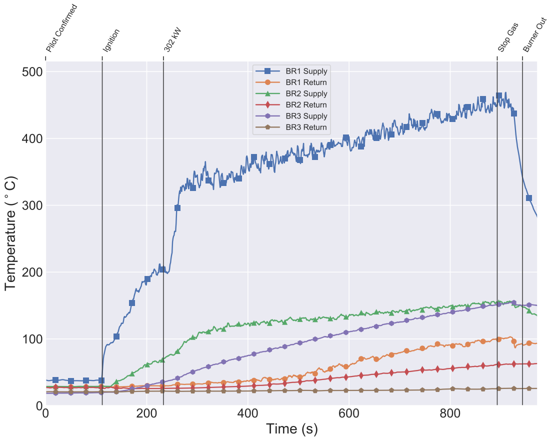

(a) Bedroom Vent Temperatures

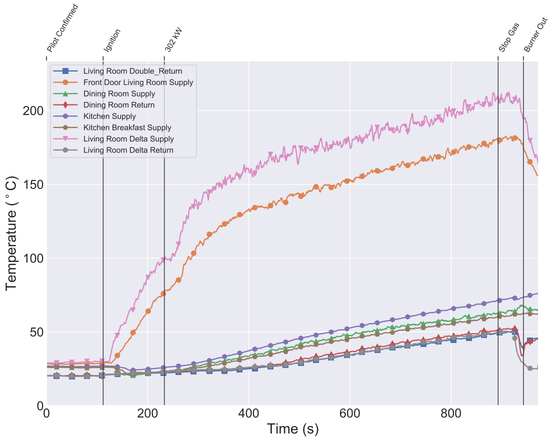

(b) First Floor Vent Temperatures

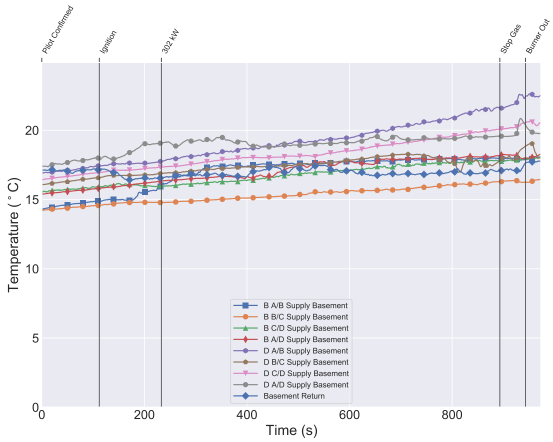

(c) Basement Vent Temperatures

Figure 5.41: Experiment 7 Vent Temperatures

The temperature and velocity within the supply duct are provided in Figure 5.42. From this figure it can be seen that there is a temperature increase within the supply duct of approximately 40 °C while the gas burner was on. The supply vent velocity appears to show that there is a constant flow of air in the supply vent from the basement to the first floor, however this held constant from before ignition to after termination of the burner. The trend in supply duct temperature is consis-

tent with what was observed in Experiment 1 but with greater magnitude due to the increased HRR.

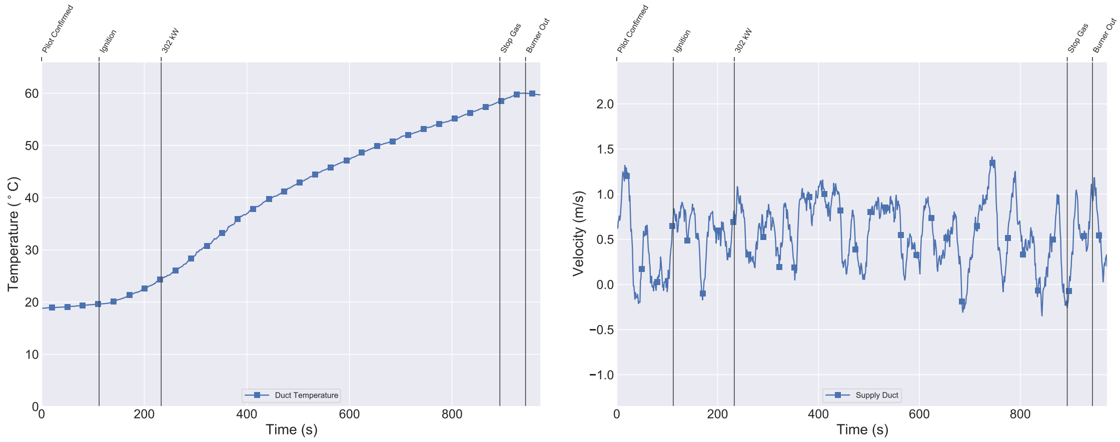

(a) Supply Duct Temperature

(b) Supply Duct Velocity

Figure 5.42: Experiment 7 Supply Duct Temperature and Velocity

5.1.8 Experiment 8

Gas Burner Experiment 8 was performed with the HVAC system off. The flow rate of propane to the gas burner was controlled so that a heat release rate of approximately 121 kW was produced. The doors to bedroom 1 and 3 were closed. The door to bedroom 2 and the staircase door to the basement were open. All exterior vents to the structure were closed. For this experiment, gas concentration measurements on the first floor were taken in bedroom 1, bedroom 2, and bedroom 3. Experiment 8 was performed to observe how heat and fire gases were transported within the HVAC ductwork with the system turned off and flow being restricted out of bedroom 1 with a closed door. The events of interest for Experiment 8 are presented in Table 5.8.

Table 5.8: Experiment 8 Events

Event	Elapsed Time (seconds)
Pilot Confirmed	0
Ignition	111
Stop Gas	553
Burner Out	582

The time versus temperature graphs for the first floor are provided in Figure 5.43. Temperatures in bedroom 1 increased approximately 280 °C at the ceiling and approximately 50 °C at the 0.3 m height while the gas burner was on. Temperatures outside bedroom 1, including in the basement, showed little change if any during this experiment. The lack of rapid temperature increase in bedroom 2 and the living room is due to the closed door preventing convective heat transfer through the doorway to bedroom 1. The temperature in bedroom 3 did not have the same increases that were observed in Experiment 1. This could indicate that there was not enough airflow into bedroom 1 to push hot gases out through the HVAC Supply system and into other parts of the structure.

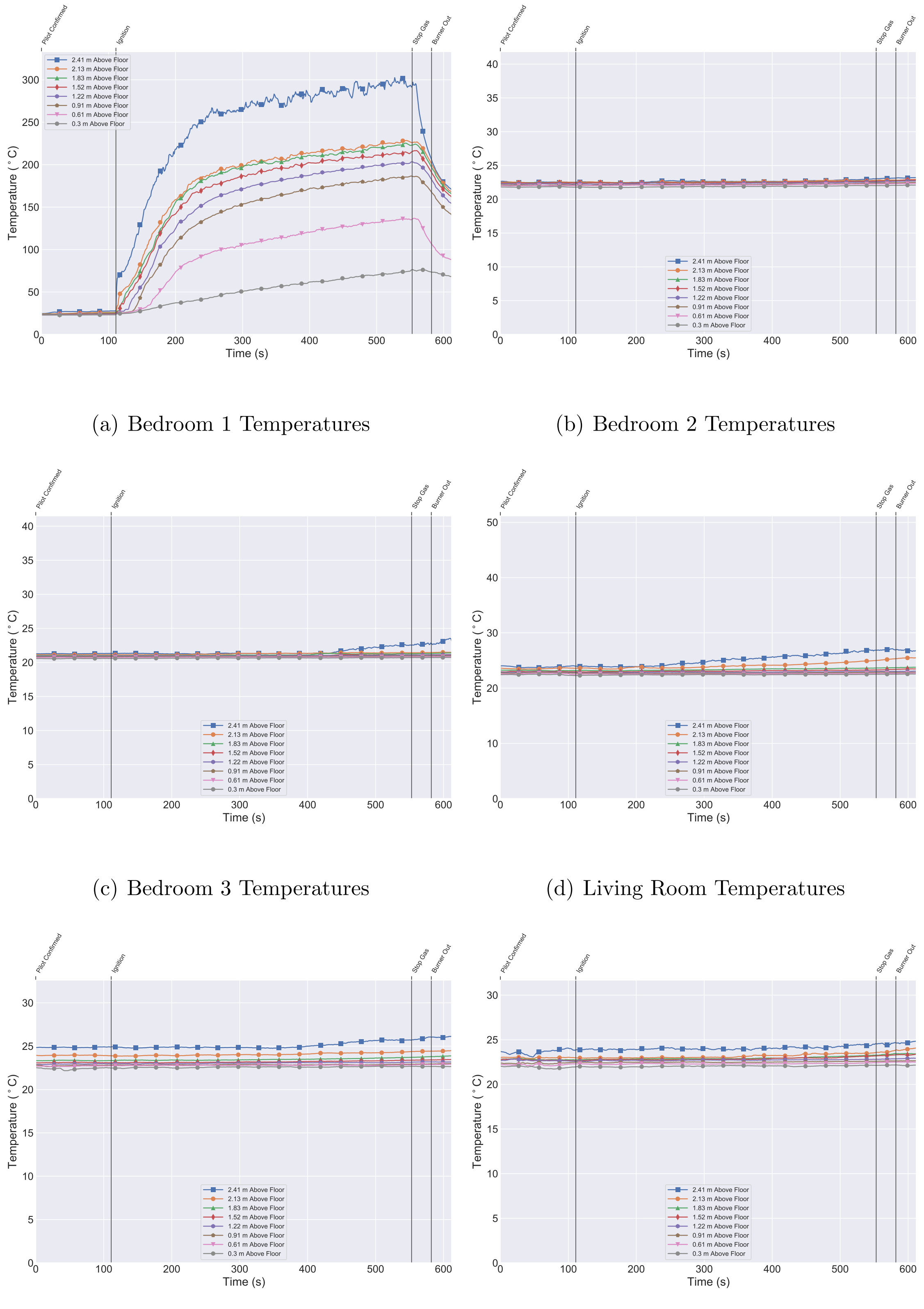

(a) Bedroom 1 Temperatures

(b) Bedroom 2 Temperatures

(c) Bedroom 3 Temperatures

(d) Living Room Temperatures

(e) Dining Room Temperatures

(f) Kitchen Temperatures

Figure 5.43: Experiment 8 First Floor Temperatures

The time versus temperature graphs for the four basement quadrants are provided in Figure 5.44. Similar to what has been observed in all bedroom 1 burner experiments, there was no notable change in basement temperatures.

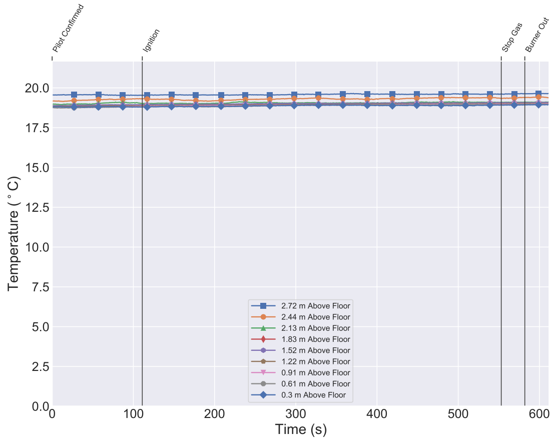

(a) Basement A Quadrant Temperatures

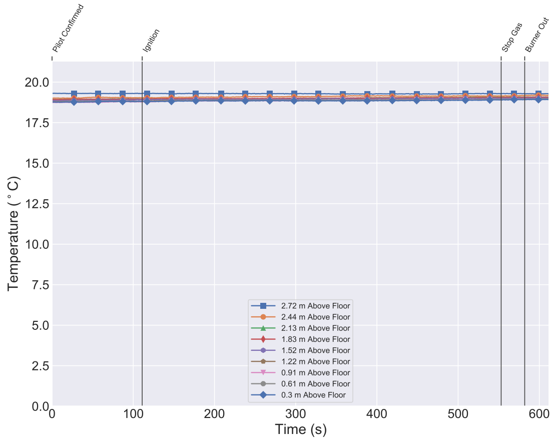

(b) Basement B Quadrant Temperatures

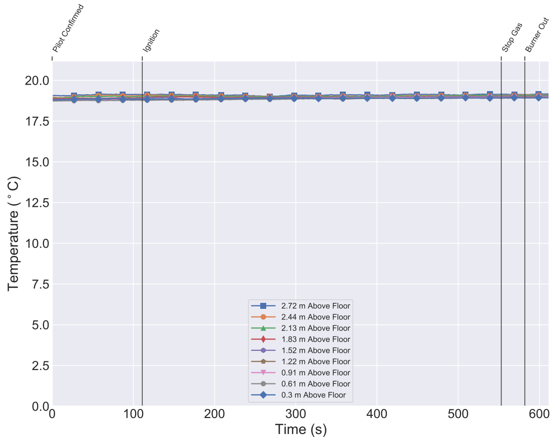

(c) Basement C Quadrant Temperatures

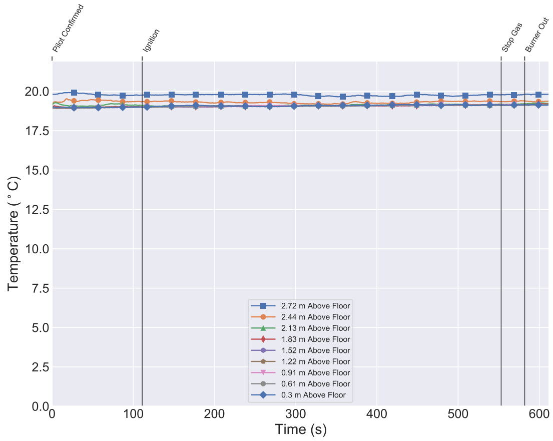

(d) Basement D Quadrant Temperatures

Figure 5.44: Experiment 8 Basement Temperatures

The pressures within each room are shown in Figure 5.45. Pressure in bedroom 1 at 2.13 m above the floor exceeded the ambient pressure by approximately 8 Pa while the burner was on. At 1.2 m above the floor pressure exceeded the ambient by

approximately 4 Pa. At 0.3 m above the floor pressure remained at approximately ambient with the exception of a spike following ignition. Pressures in bedroom 2 at all heights experienced a spike following ignition and then remained between 1 to 2 Pa greater than the ambient pressure. Pressures in bedroom 3 were similar to bedroom 2 with a steady pressure between 1 to 2 Pa following the spike at ignition. Pressures in the basement did not show any significant changes throughout the entire experiment. Pressures in the living room were similar to those recorded in bedroom 2 and 3. The pressures in the kitchen spiked after ignition and then continued to fluctuate with a greater magnitude than other rooms in the structure. Pressures at 2.13 m and 1.2 m above the floor in the kitchen peaked above 4 Pa and then fluctuated between 0 and 4 Pa during the experiment. Pressures at 0.3 m above the floor in the kitchen peaked at 3 Pa and fluctuated between -1 and 3 Pa for the duration of the experiment.

The ceiling pressure in bedroom 1 exceeded that of experiment 1 by approximately 2 Pa. The greater pressure within bedroom 1 was caused by closing the door to bedroom 1 for this experiment. Closing the door reduced the volume that smoke and gases were able to expand into, therefore increasing pressure.

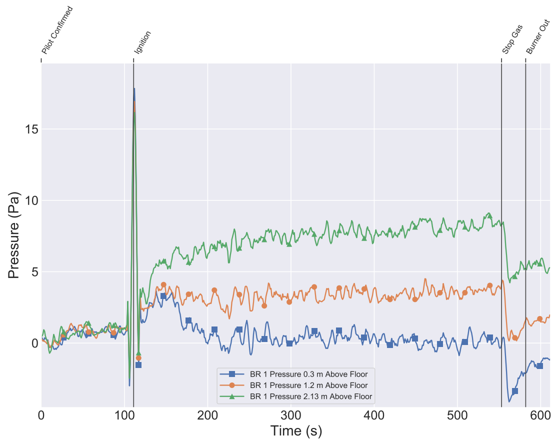

(a) Bedroom 1 Pressures

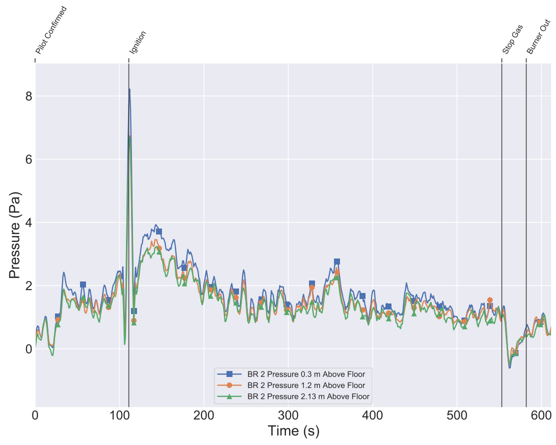

(b) Bedroom 2 Pressures

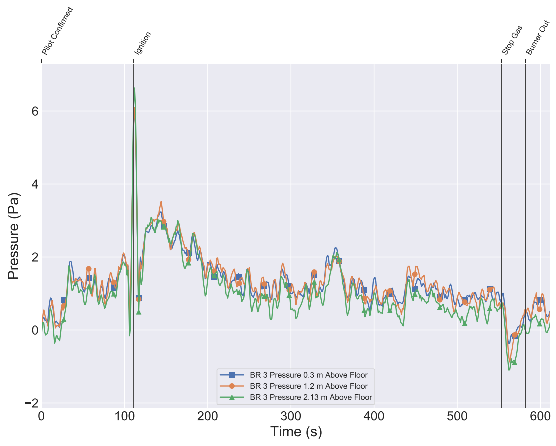

(c) Bedroom 3 Pressures

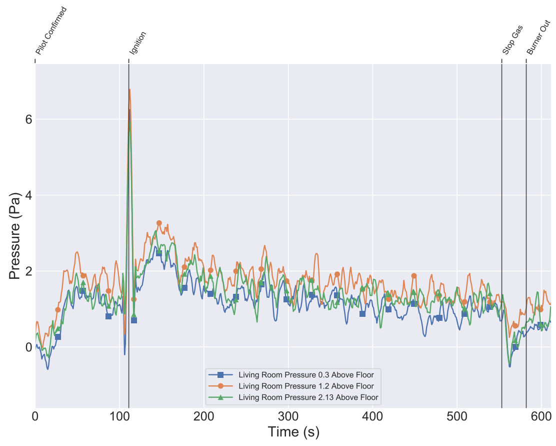

(d) Living Room Pressures

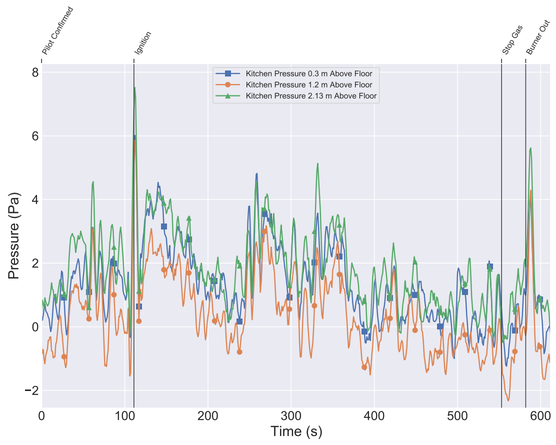

(e) Kitchen Pressures

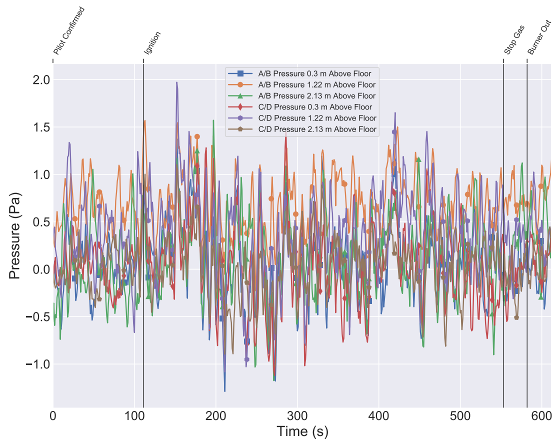

(f) Basement Pressures

Figure 5.45: Experiment 8 Pressures

The concentrations of oxygen and carbon dioxide are provided for the three bedrooms and the basement in Figure 5.46. Water vapor concentrations are included for bedroom 2, bedroom 3 and the basement. Bedroom 1 was the only bedroom to display significant changes in gas concentrations. This is because the smoke and fire gases were mostly contained within bedroom 1 by the closed door. Oxygen levels in bedroom 1 dropped to approximately 11% before gas flow was terminated. Carbon dioxide levels in bedroom 1 rose to approximately 7% during the experiment. Gas and water vapor concentrations in the rest of the structure did not display any notable changes during this experiment. Significant quanitities of smoke and fire gases were not able to traverse the supply ductwork to produce changes in other parts of the structure.

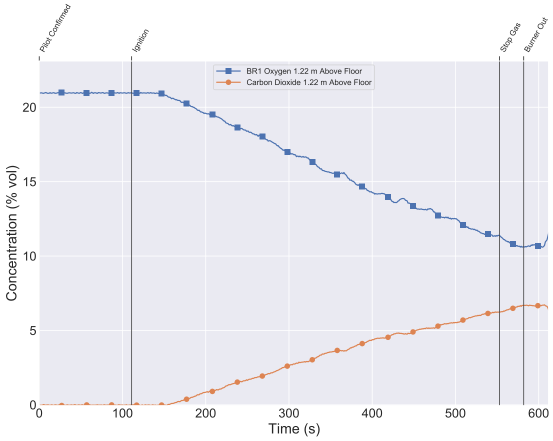

(a) Bedroom 1 Gases

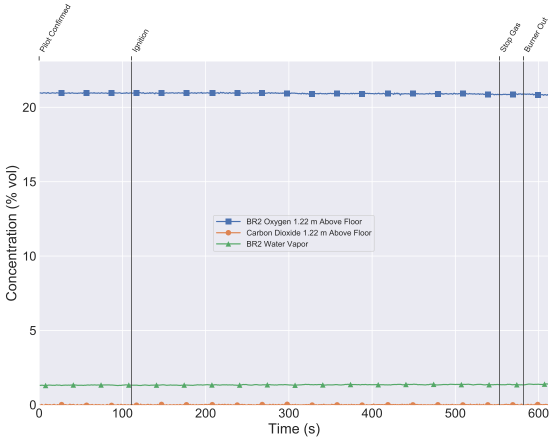

(b) Bedroom 2 Gases

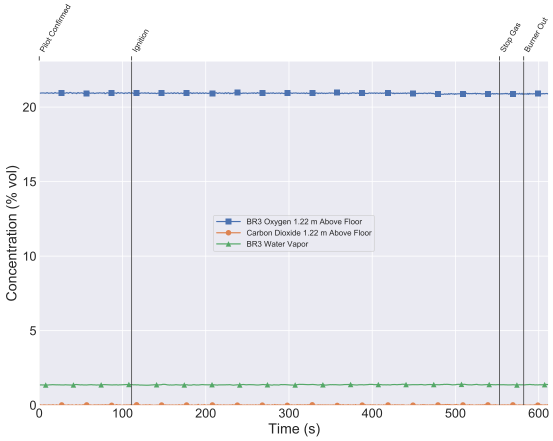

(c) Bedroom 3 Gases

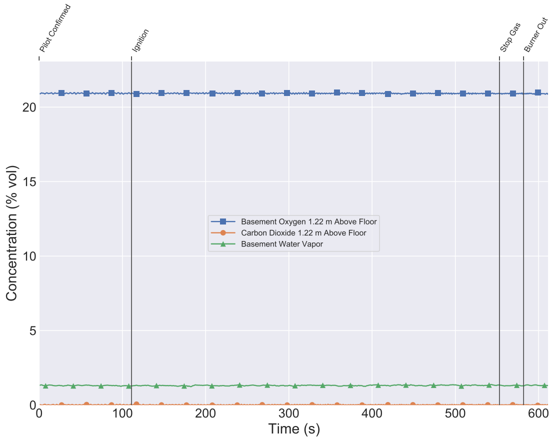

(d) Basement Gases

Figure 5.46: Experiment 8 Gases

Figure 5.47 shows the temperatures of the supply and return vents in the structure. Just as in experiment 1, it can be seen from Figure 5.47(a) that the temperature at the supply vent in bedroom 1 is the same as the ceiling temperature in bedroom 1. The supply vent temperatures in bedrooms 2 and 3 rose at a similar rate increasing approximately 30 °C over the course of the experiment. This similar rise was expected because the shortest path of least resistance to both bedrooms

was through the supply ductwork. Gases had to travel a similar distance through the ductwork to go from bedroom 1 to bedrooms 2 and 3.

Looking at the first floor vent temperatures in Figure 5.47(b), it can be seen that all supply vents rose at a similar rate, however the further the distance the gases had to travel to a vent, the longer it took for the temperature increase to register at that vent. Figure 5.47(c) shows that the temperatures at the supply vents in the basement did not increase by more than a couple of degrees celcius over the course of the experiment. This constant slight increase in basement supply vent temperatures could show that there is some heat transfer flowing from the first floor supply duct system down into the basement via the supply trunk.

The return vents in Figure 5.47, with the exception of bedroom 1, all show that there was no measurable temperature increase within the return duct system. The increase in the bedroom 1 return vent is similar to the temperature increase in bedroom 1 at 0.3 m above the floor.

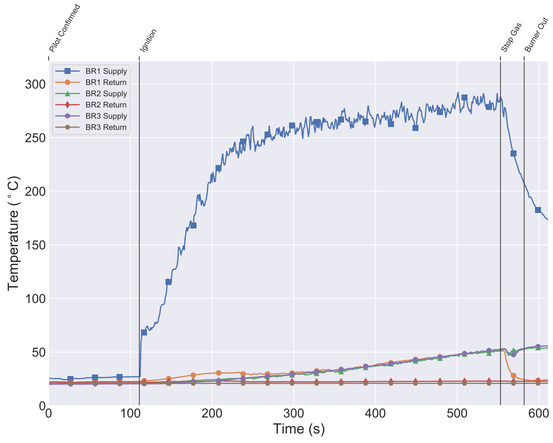

(a) Bedroom Vent Temperatures

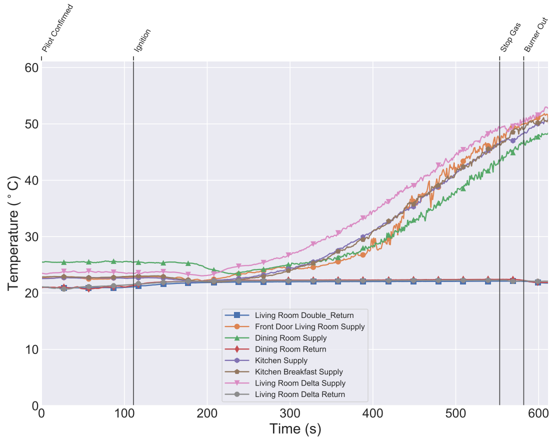

(b) First Floor Vent Temperatures

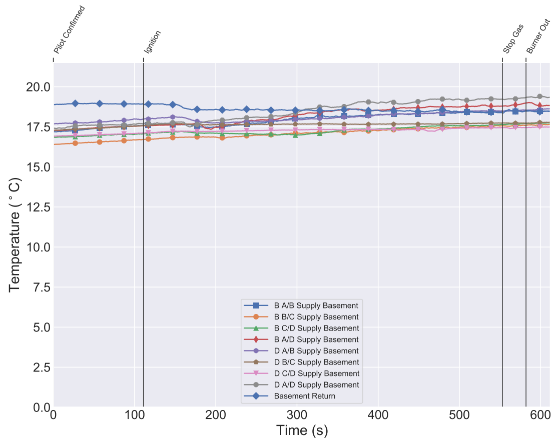

(c) Basement Vent Temperatures

Figure 5.47: Experiment 8 Vent Temperatures

The temperature and velocity within the supply duct for this experiment are provided in Figure 5.48. From Figure 5.48(a) it can be seen that there is a slight temperature increase within the supply duct of approximately 5 °C while the gas burner was on. The increase in temperature is consistent with what was observed from the supply vent temperatures in the basement but with greater magnitude, thus driving the increase in temperature of the supply ducts in the basement. The

supply vent velocity appears to show a constant flow of air in the supply vent from the basement to the first floor. While the magnitude of this flow varies, it appears to fluctuate mostly between 0 and 1.5 m/s.

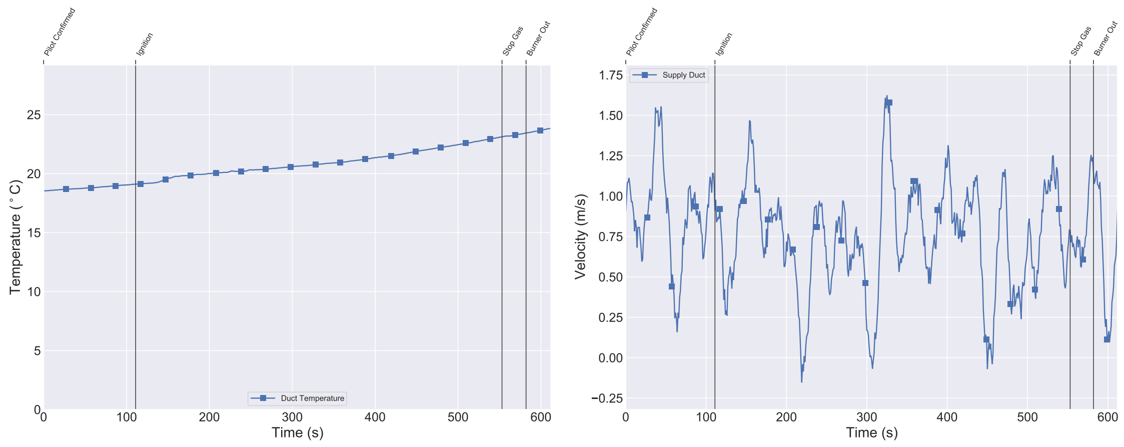

(a) Supply Duct Temperature

(b) Supply Duct Velocity

Figure 5.48: Experiment 8 Supply Duct Temperature and Velocity

5.1.9 Experiment 9

Gas Burner Experiment 9 was performed with the HVAC system set to A/C. The flow rate of propane to the gas burner was controlled so that a heat release rate of approximately 121 kW was produced. The doors to bedroom 1 and 3 were closed. The door to bedroom 2 and the staircase door to the basement were open. All exterior vents to the structure were closed. For this experiment, gas concentration measurements on the first floor were taken in bedroom 1, bedroom 2, and bedroom 3. Experiment 9 was performed to observe how heat and fire gases were transported within the HVAC ductwork with the system turned to A/C and flow being restricted out of bedroom 1 with a closed door. Comparisons of Experiment 9 to Experiment

8 are made to observe the effects of HVAC on combustion product transfer via the HVAC system. The events of interest for Experiment 9 are presented in Table 5.9.

Table 5.9: Experiment 9 Events

Event	Elapsed Time (seconds)
Pilot Confirmed	0
Ignition	126
Stop Gas	447
Burner Out	480

The time versus temperature graphs for the first floor are provided in Figure 5.49. Observation of the data collected in Experiment 9 shows that with the exception of bedroom 2, all trends identified in Experiment 8 are present in Experiment 9. In bedroom 2 there is a greater increase in temperature near the ceiling that was not observed in Experiment 8.

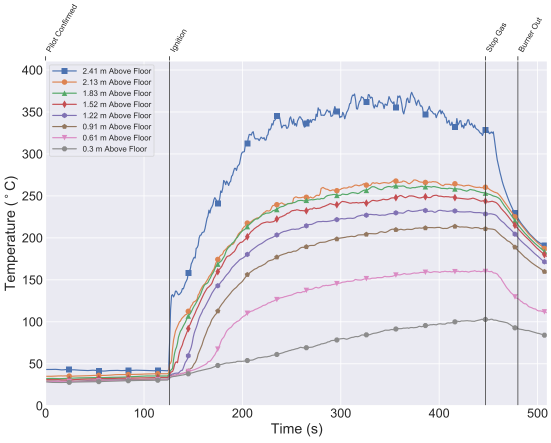

(a) Bedroom 1 Temperatures

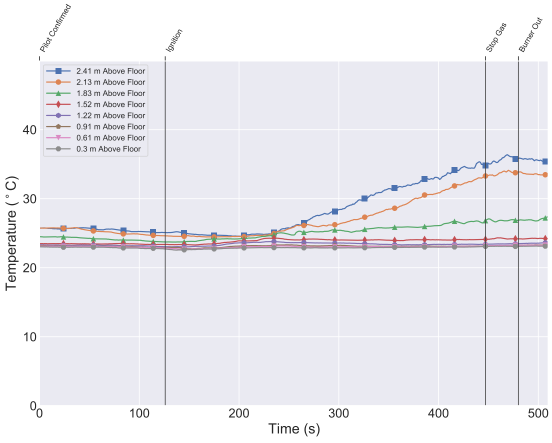

(b) Bedroom 2 Temperatures

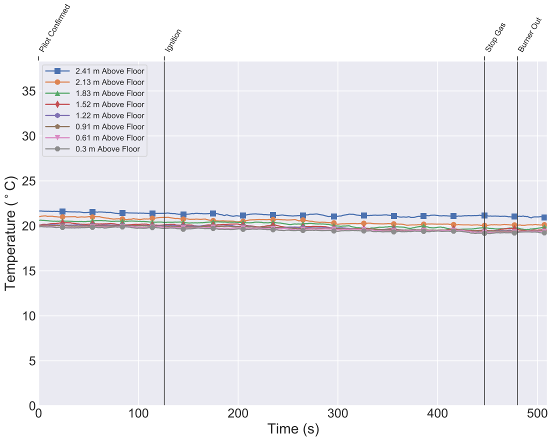

(c) Bedroom 3 Temperatures

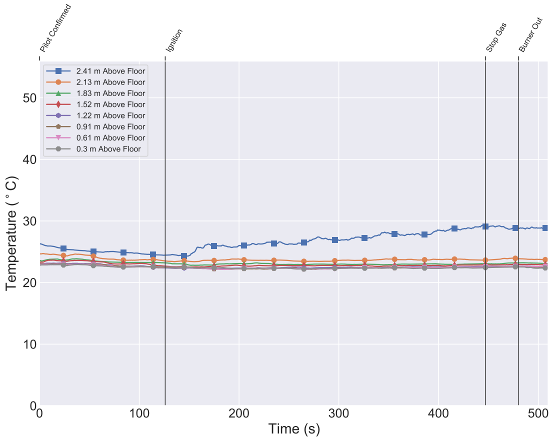

(d) Living Room Temperatures

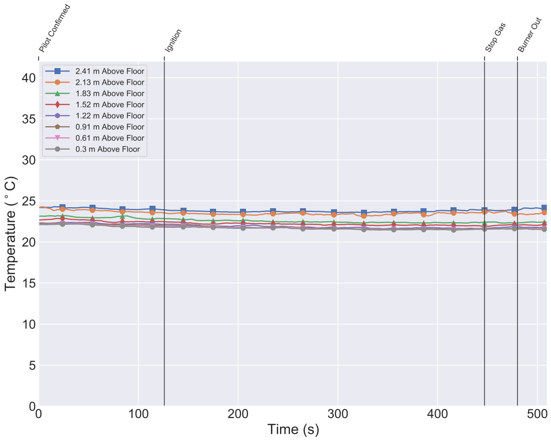

(e) Dining Room Temperatures

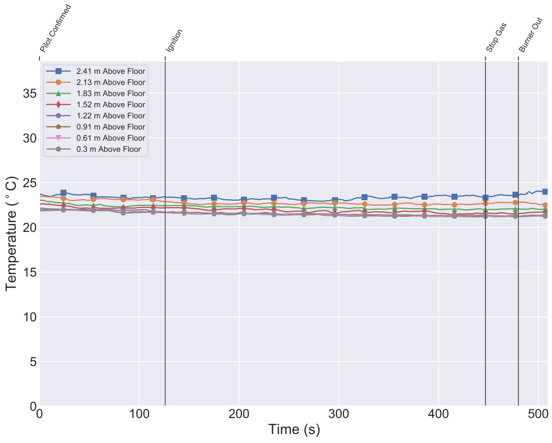

(f) Kitchen Temperatures

Figure 5.49: Experiment 9 First Floor Temperatures

The time versus temperature graphs for the four basement quadrants are provided in Figure 5.50. As was the case with Experiment 8, there was no notable change in basement temperatures.

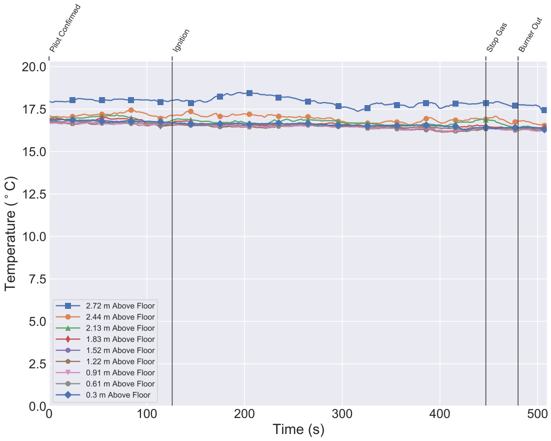

(a) Basement A Quadrant Temperatures

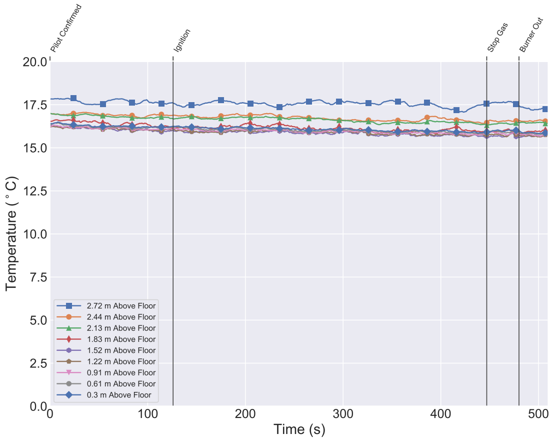

(b) Basement B Quadrant Temperatures

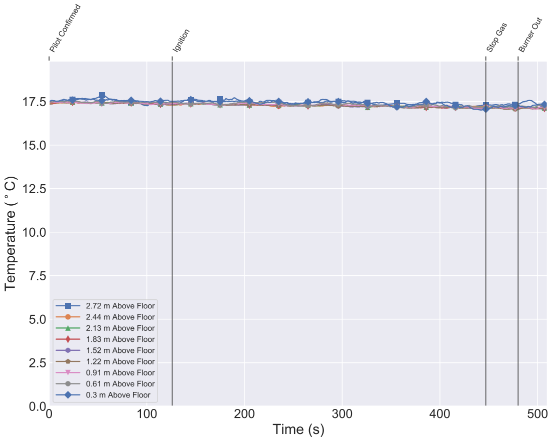

(c) Basement C Quadrant Temperatures

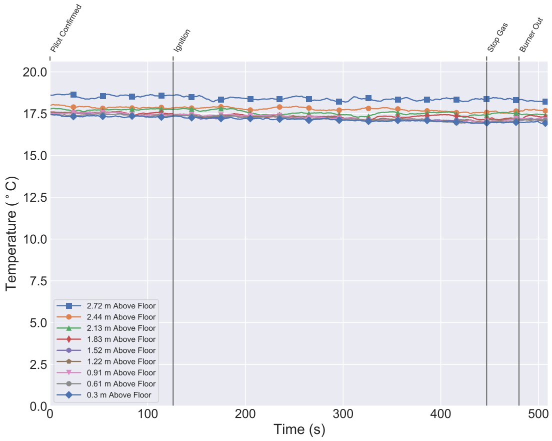

(d) Basement D Quadrant Temperatures

Figure 5.50: Experiment 9 Basement Temperatures

The pressures within each room are shown in Figure 5.51. With the exception of the pressure spike immediately following ignition, comparison of recorded pressures in bedroom 1 between Experiment 8 and Experiment 9 yield similar values and

trends. Post ignition pressure spikes across the structure were almost double the values observed in Experiment 8. Pressures in bedroom 2, bedroom 3 and the living room remained around ambient for the majority of the experiment. Pressures in the kitchen and basement behaved similar to that of the data presented in Experiment 8.

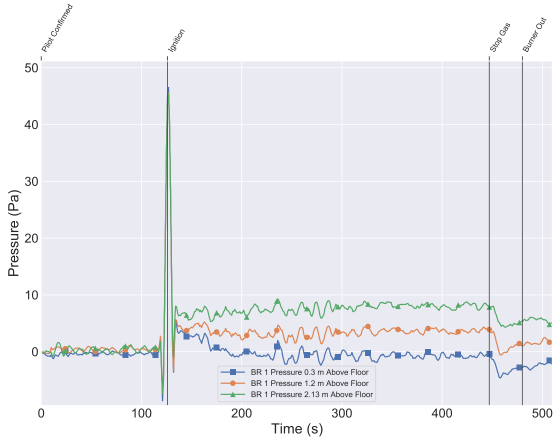

(a) Bedroom 1 Pressures

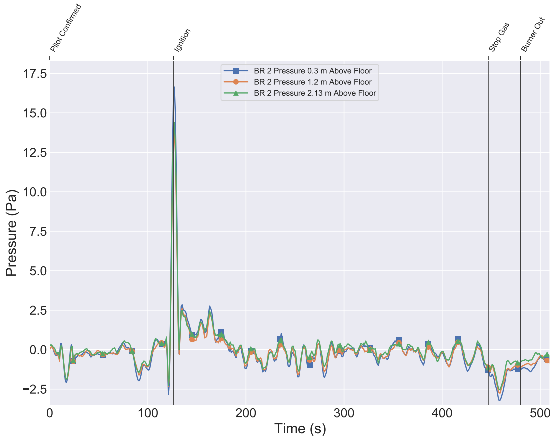

(b) Bedroom 2 Pressures

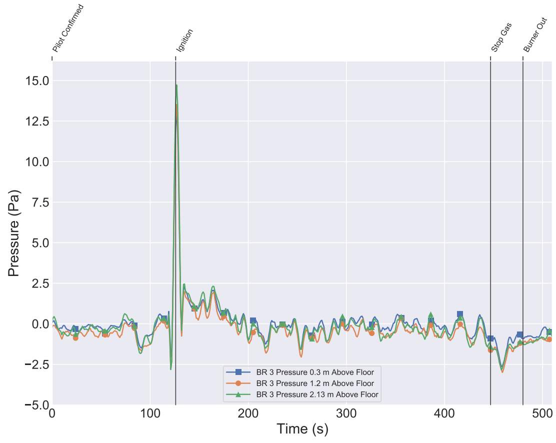

(c) Bedroom 3 Pressures

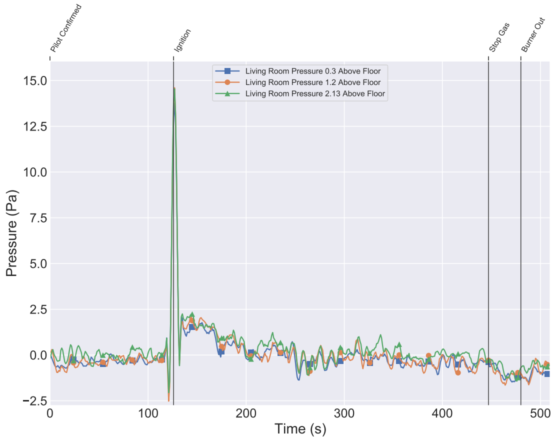

(d) Living Room Pressures

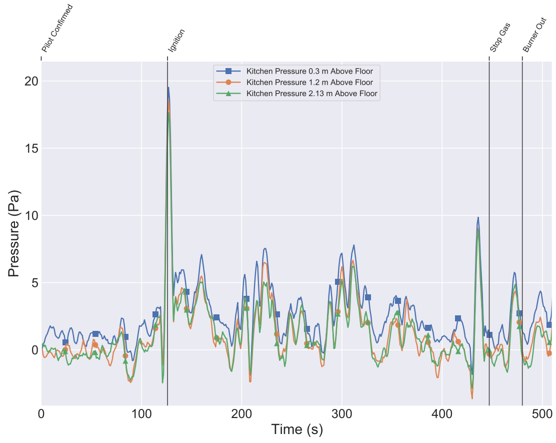

(e) Kitchen Pressures

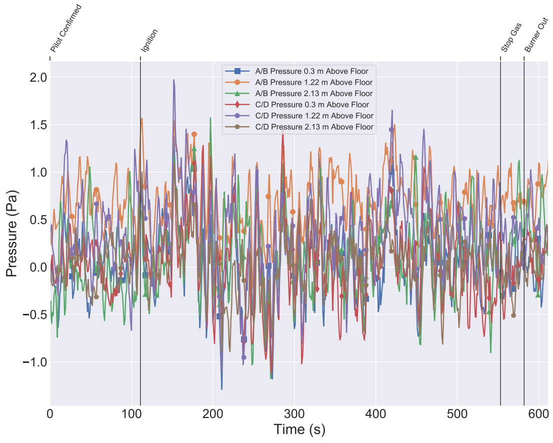

(f) Basement Pressures

Figure 5.51: Experiment 9 Pressures

The concentrations of oxygen and carbon dioxide are provided for the three bedrooms and the basement in Figure 5.52. Water vapor concentrations are included for bedroom 2, bedroom 3 and the basement. Similar to Experiment 8, Bedroom 1 was the only bedroom to display significant changes in gas concentrations. Oxygen levels in bedroom 1 dropped to approximately 8% before gas flow was terminated. Carbon dioxide levels in bedroom 1 rose to approximately 8% during the experiment. Gas and water vapor concentrations in the rest of the structure did not display any notable changes during this experiment.

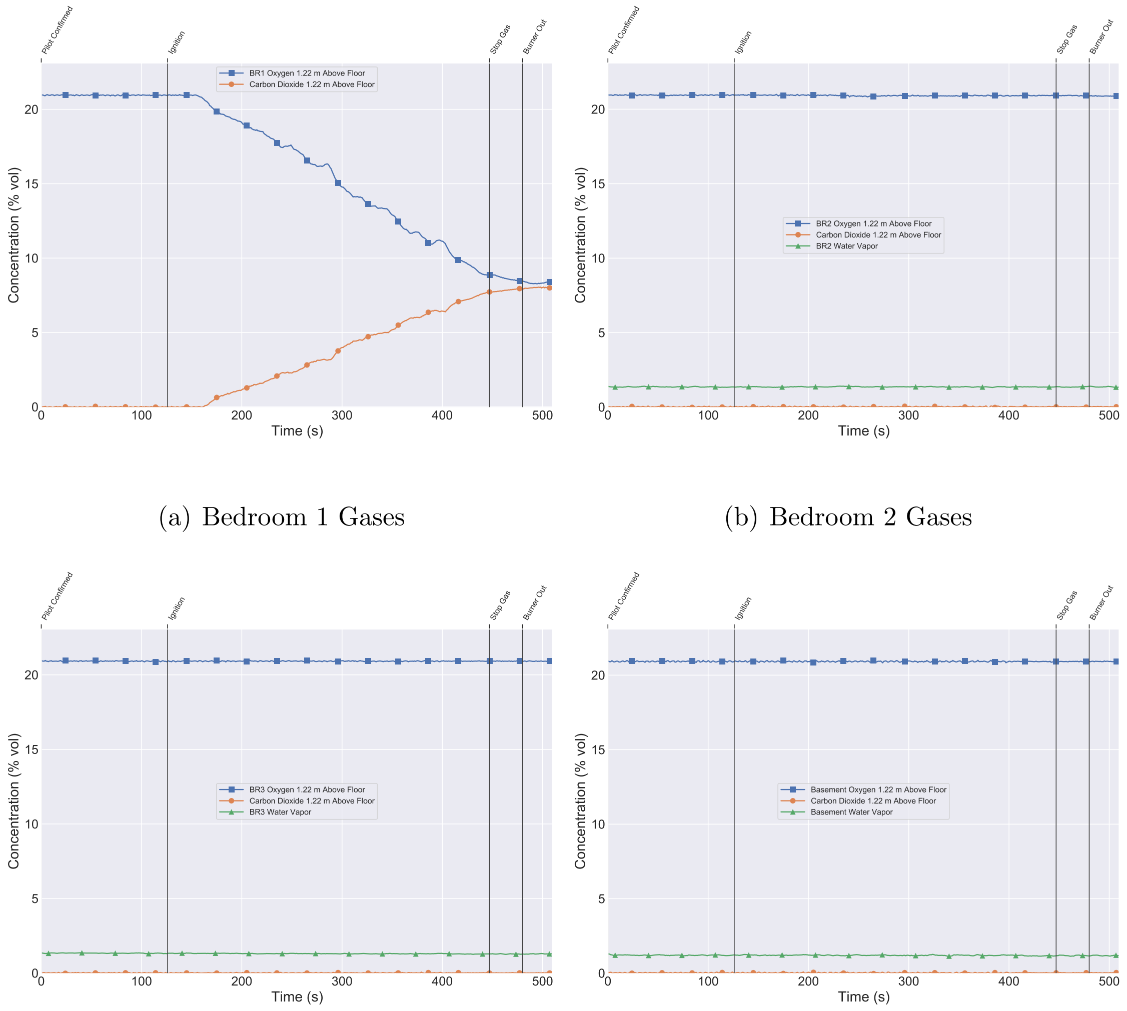

(a) Bedroom 1 Gases

(b) Bedroom 2 Gases

(c) Bedroom 3 Gases

(d) Basement Gases

Figure 5.52: Experiment 9 Gases

Figure 5.53 shows the temperatures of the supply and return vents in the structure. Just as in experiment 1, it can be seen from Figure 5.53(a) that the temperatures in the bedrooms behaved in a similar to manner to the data reported in Experiment 8. Looking at the rest of the first floor vent temperatures in Figure 5.53(b), there is no increase in temperature at any of the supply or return vents. From this experiment, it would appear that the HVAC system set in A/C was able

to prevent the hot gases from spreading through the HVAC system to the rest of the first floor vents. Figure 5.53(c) shows that the temperatures at the supply and return vents in the basement did not increase over the course of the experiment.

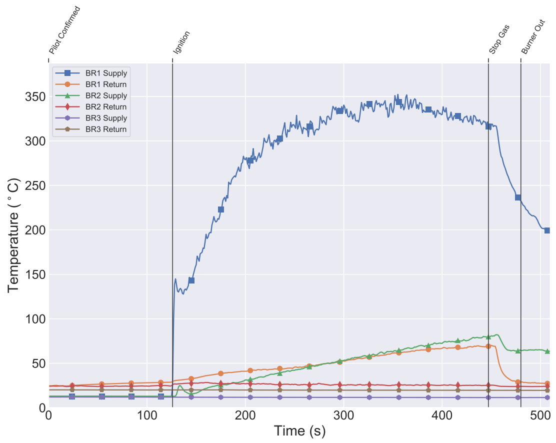

(a) Bedroom Vent Temperatures

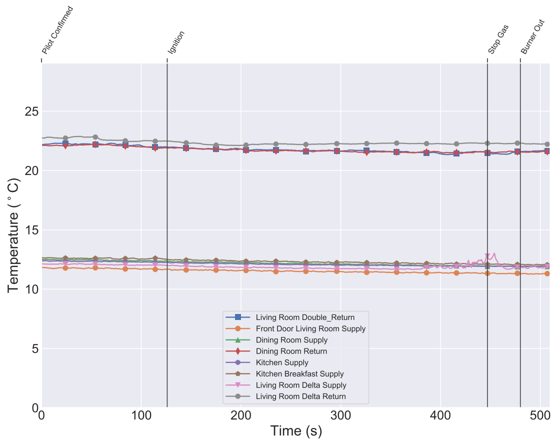

(b) First Floor Vent Temperatures

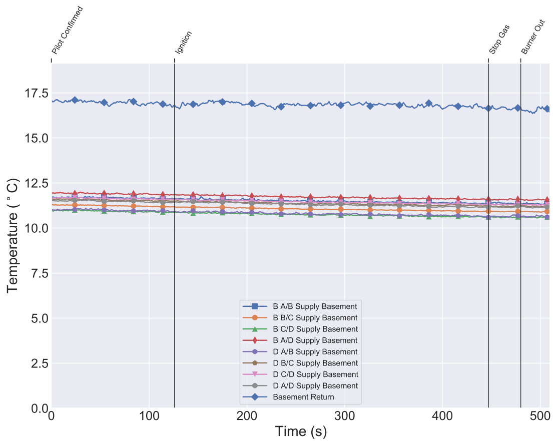

(c) Basement Vent Temperatures

Figure 5.53: Experiment 9 Vent Temperatures

The temperature and velocity within the supply duct for this experiment are provided in Figure 5.54. From Figure 5.54(a) it can be seen that there is a no increase in temperature within the supply duct while the gas burner was on. The

supply vent velocity appears to show a flow of air in the supply vent from the first floor to the basement, however that would not support the constant temperature that was supplied from the AHU in the basement.

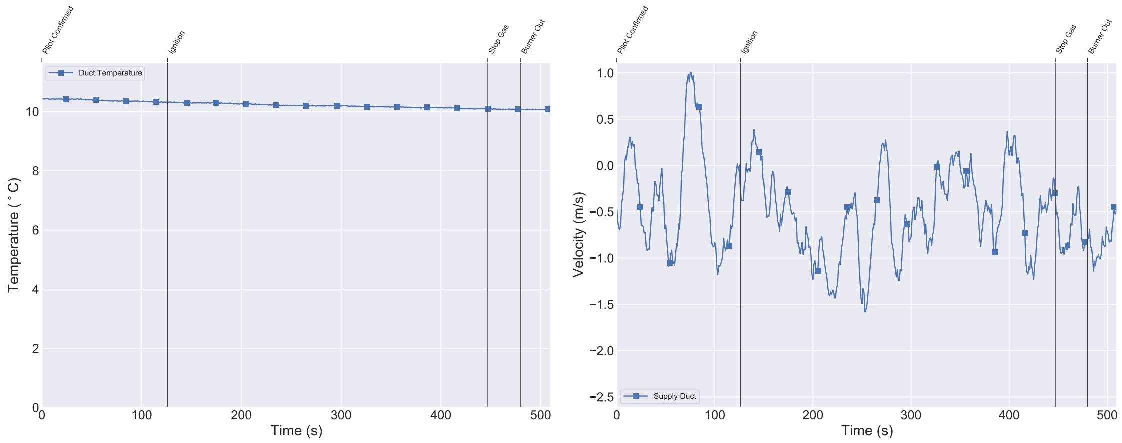

(a) Supply Duct Temperature

(b) Supply Duct Velocity

Figure 5.54: Experiment 9 Supply Duct Temperature and Velocity

5.1.10 Experiment 10

Gas Burner Experiment 10 was performed with the HVAC system off. The flow rate of propane to the gas burner was controlled so that a heat release rate of approximately 121 kW was produced. The doors to bedroom 1 and 3 were closed. The door to bedroom 2 and the staircase door to the basement were open. All exterior vents to the structure were closed. A louver vent was installed above the bedroom 1 door. For this experiment, gas concentration measurements on the first floor were taken in bedroom 1, bedroom 2, and bedroom 3. Experiment 10 was performed to observe how heat and fire gases were transported throughout the structure with the addition of the louver vent. Comparisons will be made to

Experiments 8 and 9. The events of interest for Experiment 10 are presented in Table 5.10.

Table 5.10: Experiment 10 Events

Event	Elapsed Time (seconds)
Pilot Confirmed	0
Ignition	106
Stop Gas	527
Burner Out	611

The time versus temperature graphs for the first floor are provided in Figure 5.55. Comparison of the data collected in Experiment 10 to that of Experiment 8 shows that the addition of the louver vent allowed for more heat to be transferred out of bedroom 1 and spread across the first floor. Temperatures in bedroom 3 rose at a similar rate to that of Experiment 8. Comparison to Experiment 1 shows that the temperatures across the first floor did not increase as much as was the case with the bedroom 1 door open, but the louver vent did allow for heat transfer out of bedroom 1.

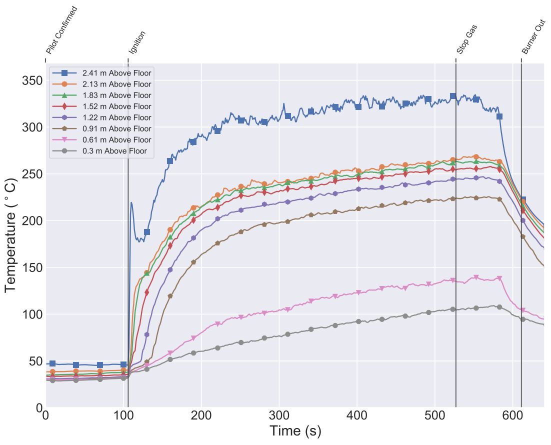

(a) Bedroom 1 Temperatures

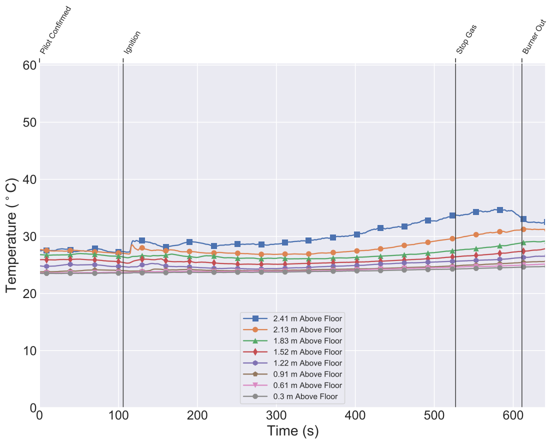

(b) Bedroom 2 Temperatures

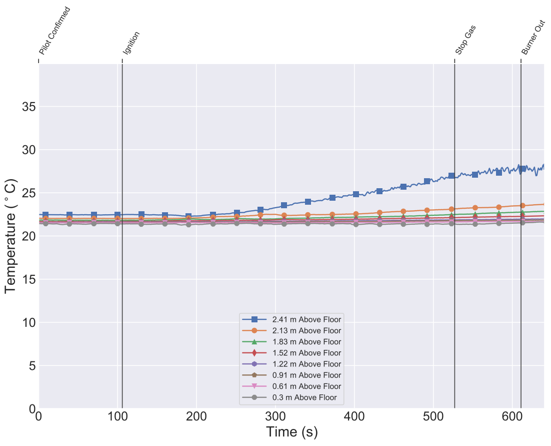

(c) Bedroom 3 Temperatures

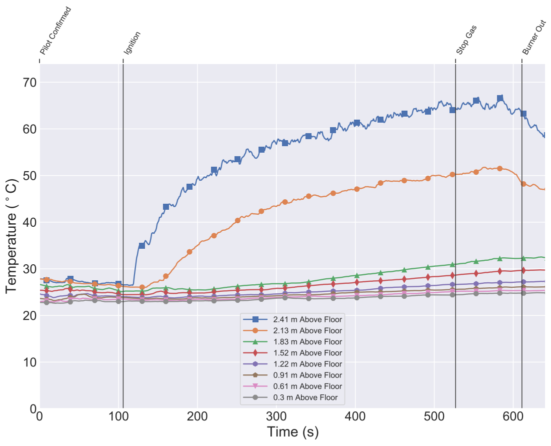

(d) Living Room Temperatures

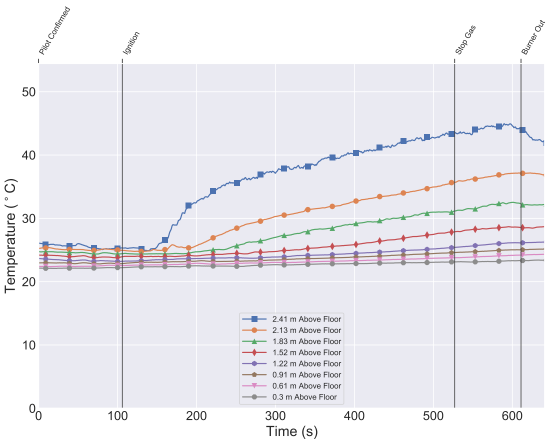

(e) Dining Room Temperatures

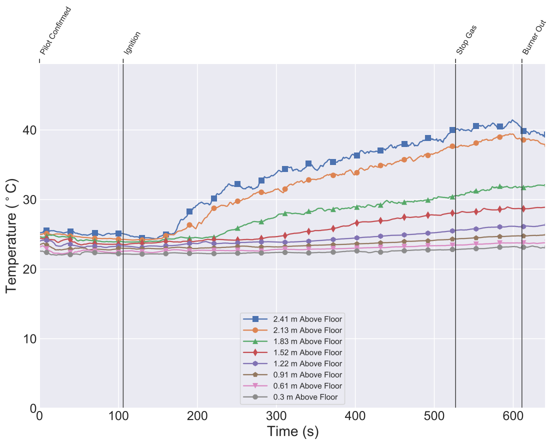

(f) Kitchen Temperatures

Figure 5.55: Experiment 10 First Floor Temperatures

The time versus temperature graphs for the four basement quadrants are provided in Figure 5.56. No notable temperature changes occurred in the basement during this experiment.

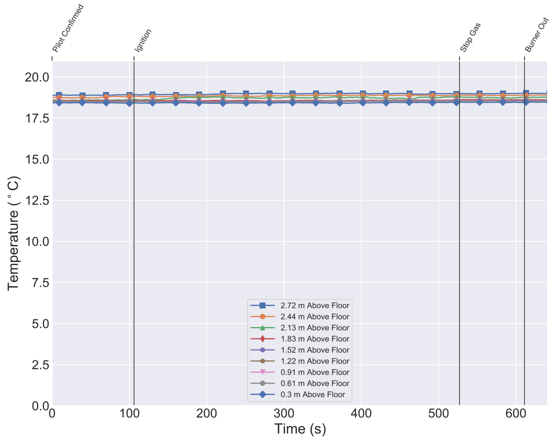

(a) Basement A Quadrant Temperatures

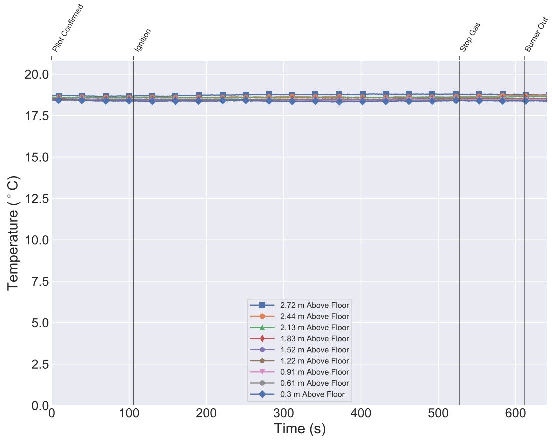

(b) Basement B Quadrant Temperatures

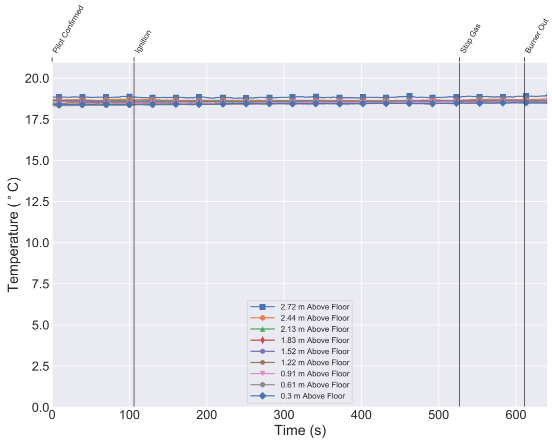

(c) Basement C Quadrant Temperatures

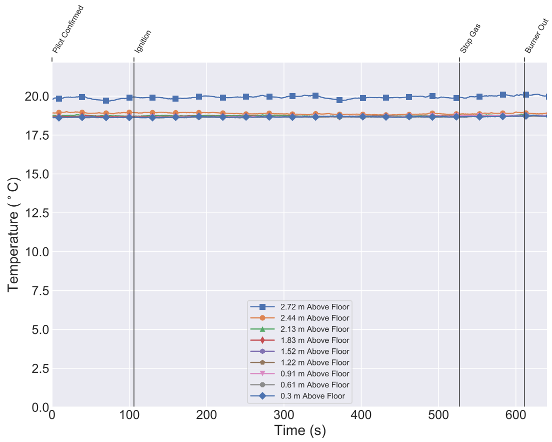

(d) Basement D Quadrant Temperatures

Figure 5.56: Experiment 10 Basement Temperatures

The pressures within each room are shown in Figure 5.57. Pressure measurments from Experiment 10 are similar to the pressures recorded in Experiment 9, including the large pressure spikes following ignition. One location where the pres-

sure varied drastically from the previous experiment was the kitchen. Pressures in the kitchen appeared to fluctuate between 20 Pa below and above the ambient for the duration of the experiment.

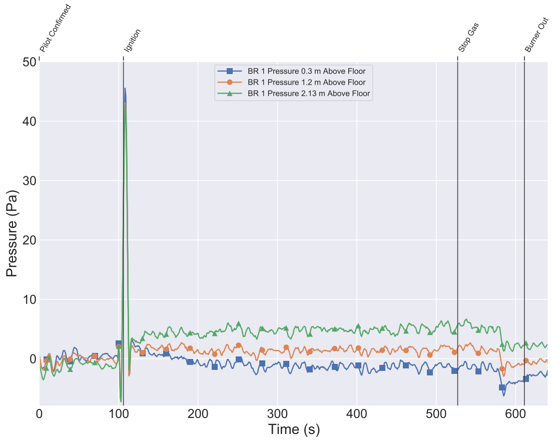

(a) Bedroom 1 Pressures

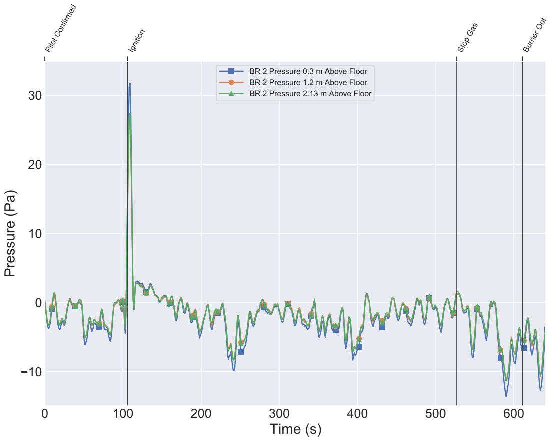

(b) Bedroom 2 Pressures

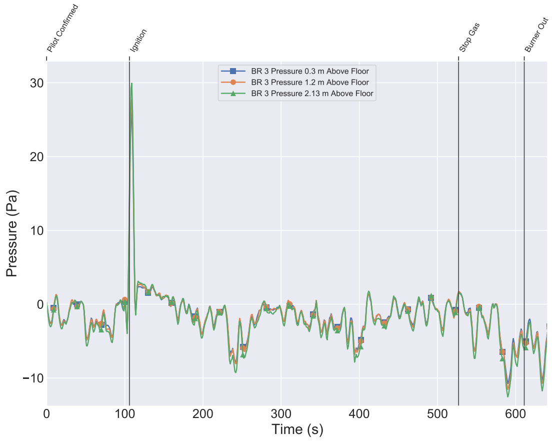

(c) Bedroom 3 Pressures

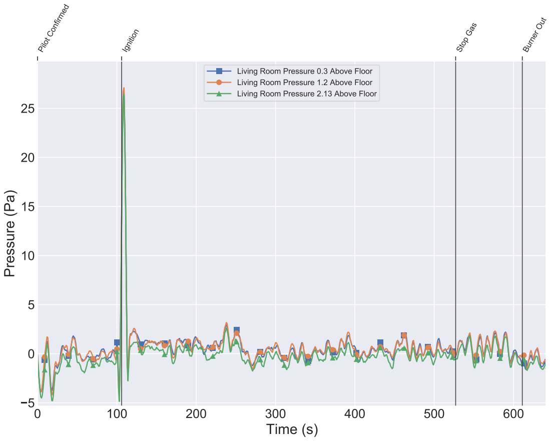

(d) Living Room Pressures

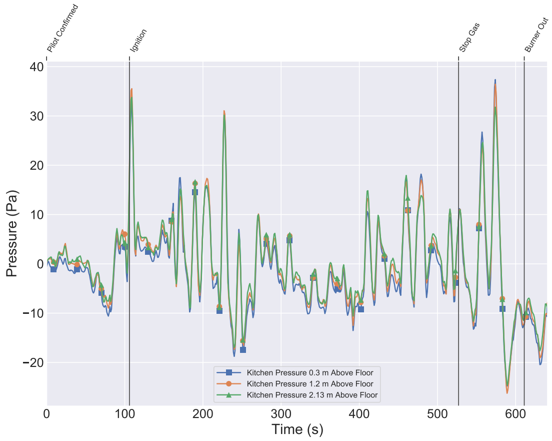

(e) Kitchen Pressures

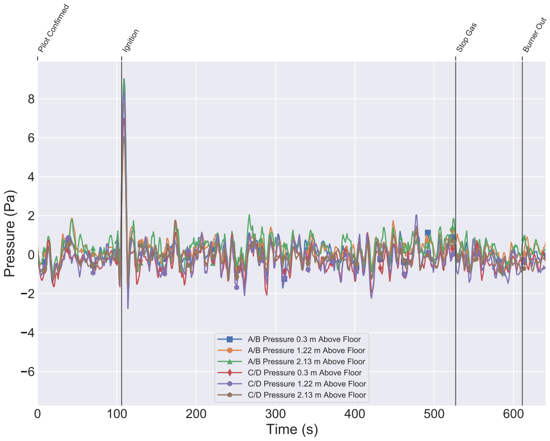

(f) Basement Pressures

Figure 5.57: Experiment 10 Pressures

The concentrations of oxygen and carbon dioxide are provided for the three bedrooms and the basement in Figure 5.58. Water vapor concentrations are included for bedroom 2, bedroom 3 and the basement. The data collected in Experiment 10 is similar to the trends observed in Experiment 8.

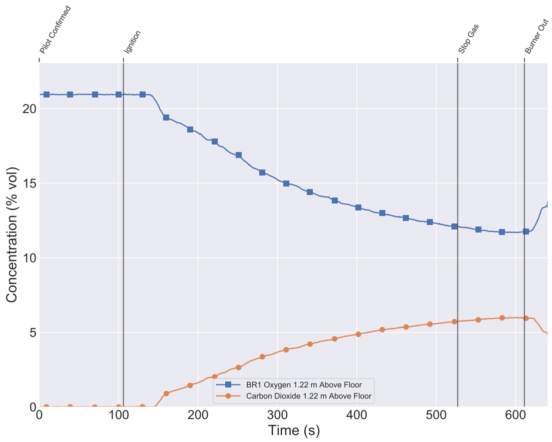

(a) Bedroom 1 Gases

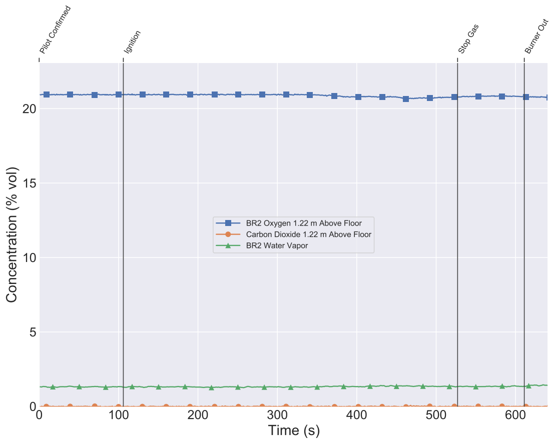

(b) Bedroom 2 Gases

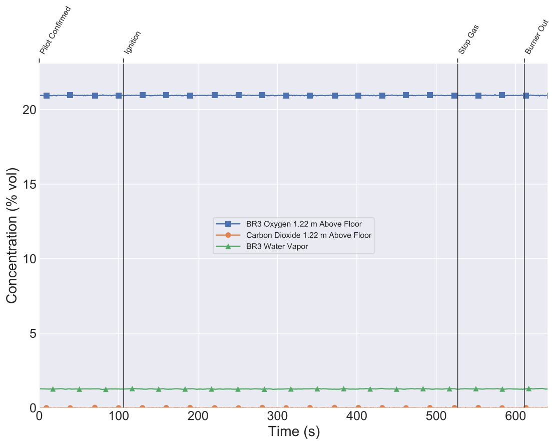

(c) Bedroom 3 Gases

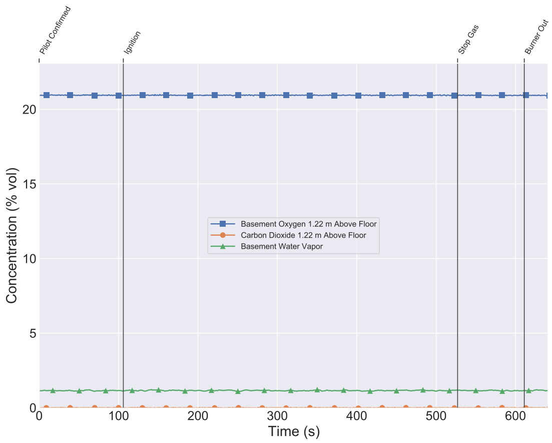

(d) Basement Gases

Figure 5.58: Experiment 10 Gases

Figure 5.59 shows the temperatures of the supply and return vents in the structure. By observation of Figure 5.59(a), it can be seen that the bedroom vents

behave the same as those of Experiment 8. In contrast to Experiment 8, the living room supply vents were heated by the convective air flowing from the louver vent. Towards the end of the experiment, it is observed that the living room delta supply temperature decreases as the pressure within the vents surpasses that of the living room. The larger pressure in the vents pushed the cooler air that was still in the supply ducts out into the living, thus cooling the vent temperature. Apart from the living room supply vents, the remaining vents on the first floor and in the basement displayed similar data to that observed in Experiment 8.

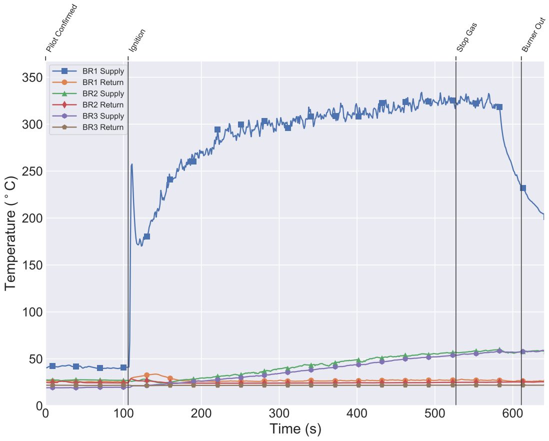

(a) Bedroom Vent Temperatures

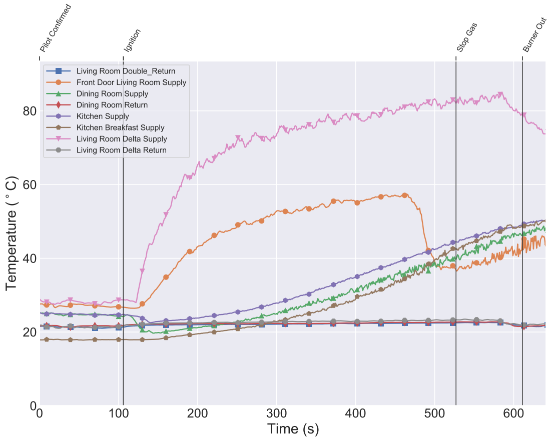

(b) First Floor Vent Temperatures

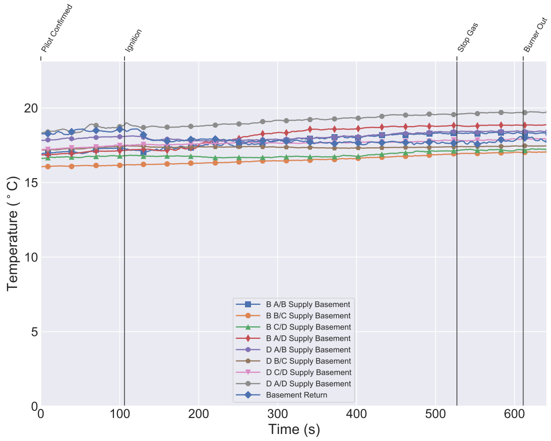

(c) Basement Vent Temperatures

Figure 5.59: Experiment 10 Vent Temperatures

The temperature and velocity within the supply duct for this experiment are provided in Figure 5.60. The supply duct measurements presented similar trends to those observed in Experiment 8.

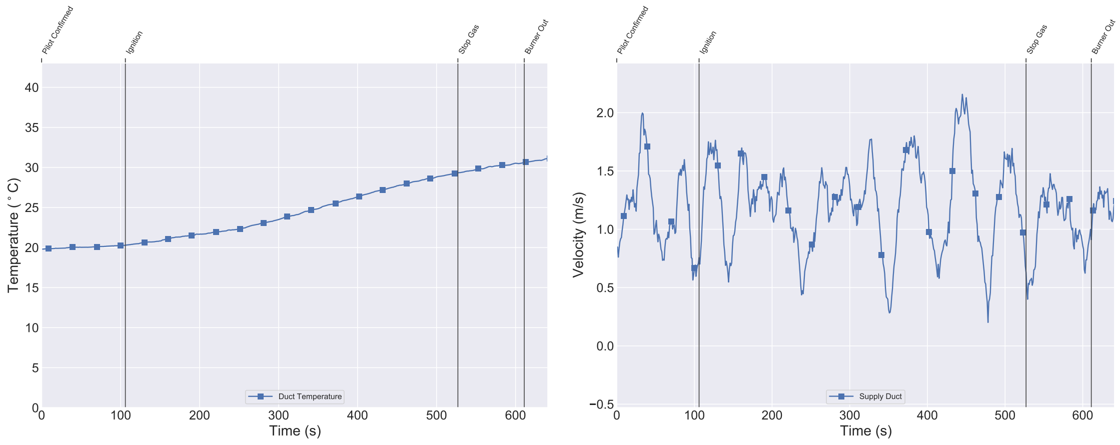

(a) Supply Duct Temperature

(b) Supply Duct Velocity

Figure 5.60: Experiment 10 Supply Duct Temperature and Velocity

5.1.11 Experiment 11

Gas Burner Experiment 11 was performed with the HVAC system off. The flow rate of propane to the gas burner was controlled so that a heat release rate of approximately 121 kW was produced. The doors to bedroom 1 and 3 were closed. The door to bedroom 2 and the staircase door to the basement were open. All exterior vents to the structure were closed. A louver vent was installed above the bedroom 1 and bedroom 3 doors. For this experiment, gas concentration measurements on the first floor were taken in bedroom 1, bedroom 2, and bedroom 3. Experiment 11 was performed to observe how heat and fire gases were transported throughout the structure with the addition of an additional louver vent above the closed bedroom 3 door. Comparisons will be made to Experiments 8 and 10. The events of interest for Experiment 10 are presented in Table 5.11.

Table 5.11: Experiment 11 Events

Event	Elapsed Time (seconds)
Pilot Confirmed	0
Ignition	122
Stop Gas	543
Burner Out	618

The time versus temperature graphs for the first floor are provided in Figure 5.61. Temperature trends across the first floor were similar to those reported in Experiment 10. The addition of the louver vent in bedroom 3 enabled a more direct route for convective heating into the closed bedroom. Temperatures in bedroom 3 rose quicker than in other experiments because of the addition of the louver vent.

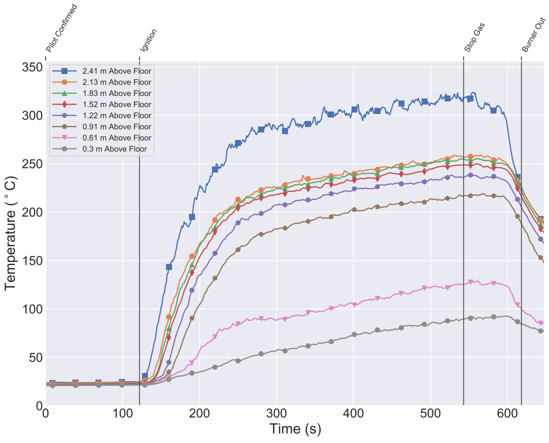

(a) Bedroom 1 Temperatures

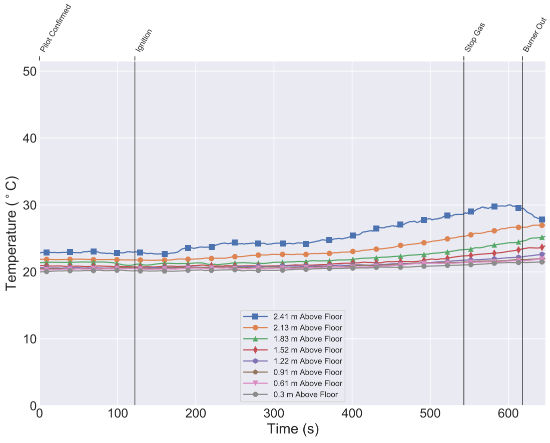

(b) Bedroom 2 Temperatures

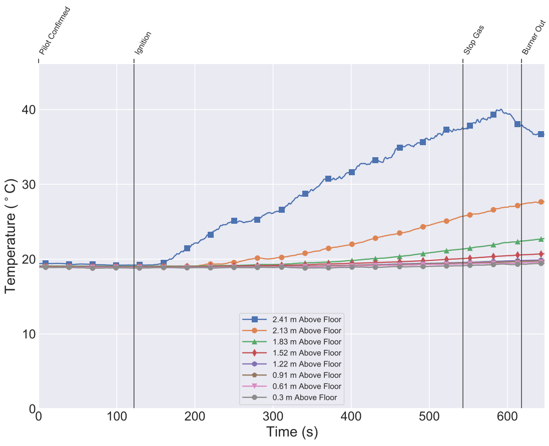

(c) Bedroom 3 Temperatures

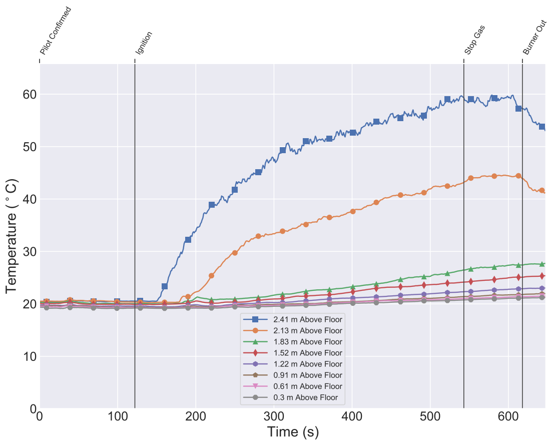

(d) Living Room Temperatures

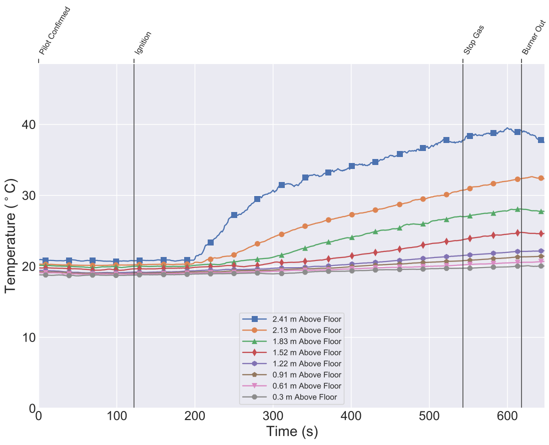

(e) Dining Room Temperatures

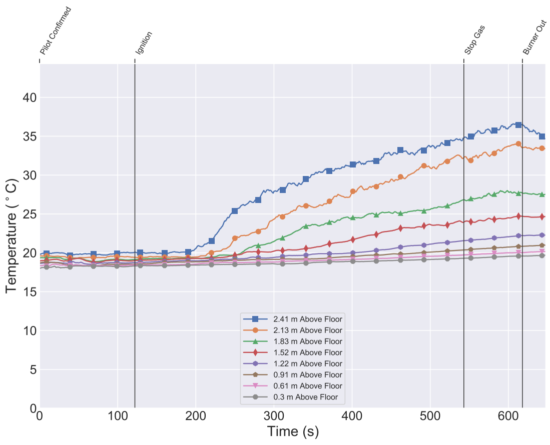

(f) Kitchen Temperatures

Figure 5.61: Experiment 11 First Floor Temperatures

The time versus temperature graphs for the four basement quadrants are provided in Figure 5.62. No notable temperature changes occurred in the basement during this experiment.

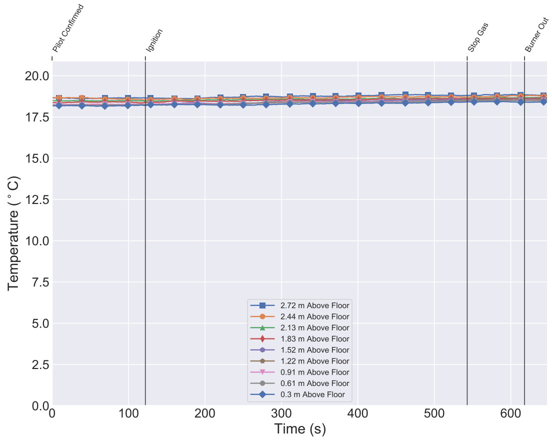

(a) Basement A Quadrant Temperatures

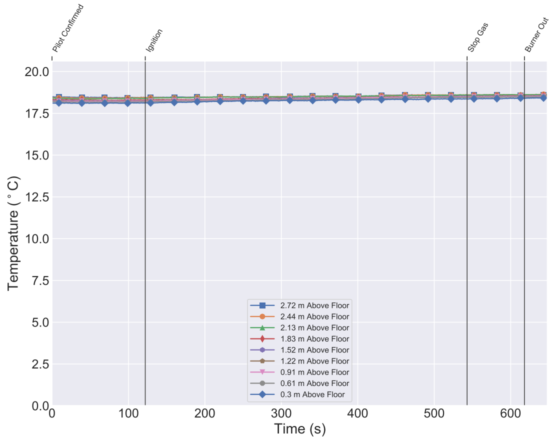

(b) Basement B Quadrant Temperatures

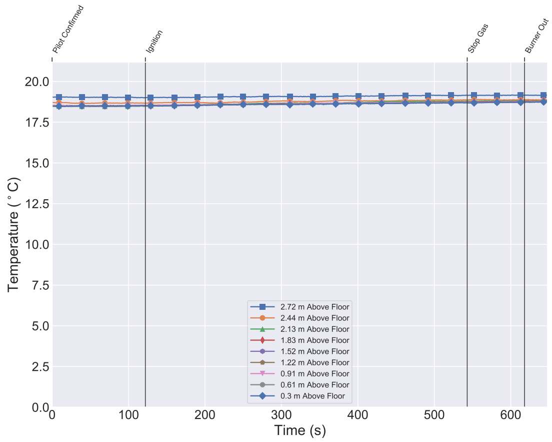

(c) Basement C Quadrant Temperatures

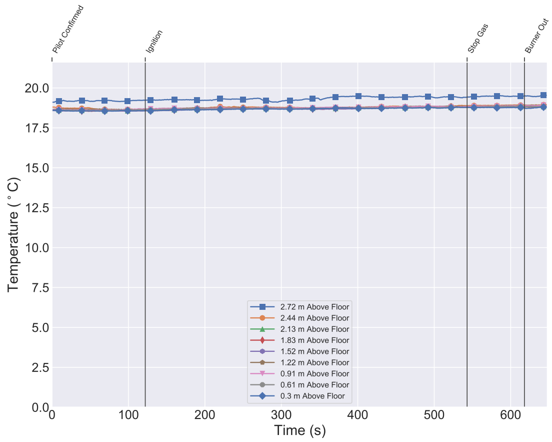

(d) Basement D Quadrant Temperatures

Figure 5.62: Experiment 11 Basement Temperatures

The pressures within each room are shown in Figure 5.63. In contrast to Experiments 8 and 10, there was no recorded pressure spike following ignition. Pressures at the 2.13 m height held steady around 6 Pa during the experiment. At the 1.2 m

height pressures remained between 0 and 2 Pa. at 0.3 m above the floor the pressure remained between 2 and 4 Pa below the ambient.

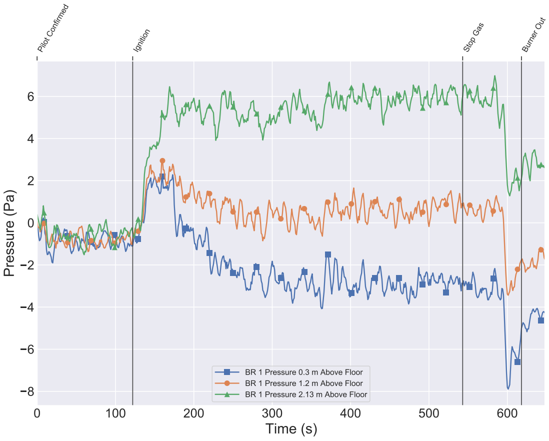

(a) Bedroom 1 Pressures

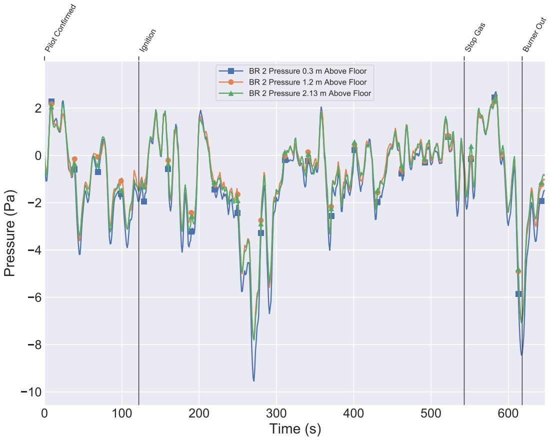

(b) Bedroom 2 Pressures

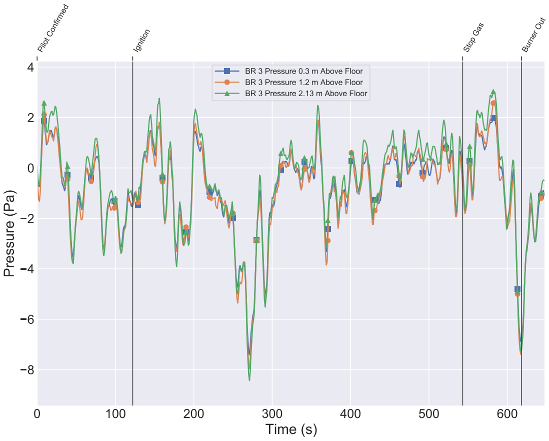

(c) Bedroom 3 Pressures

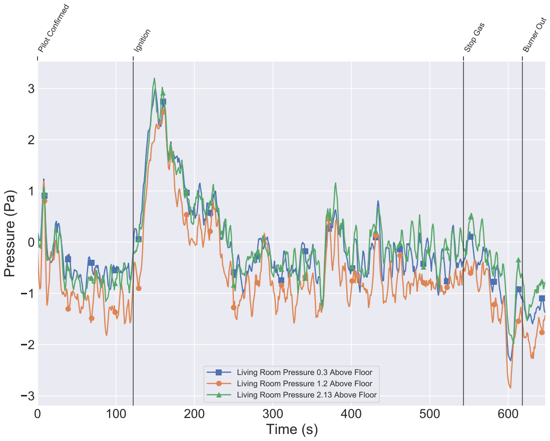

(d) Living Room Pressures

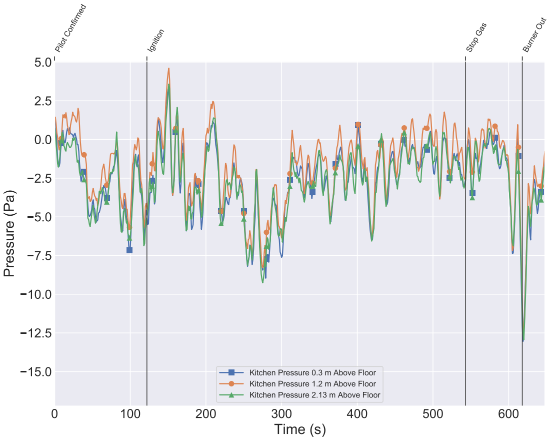

(e) Kitchen Pressures

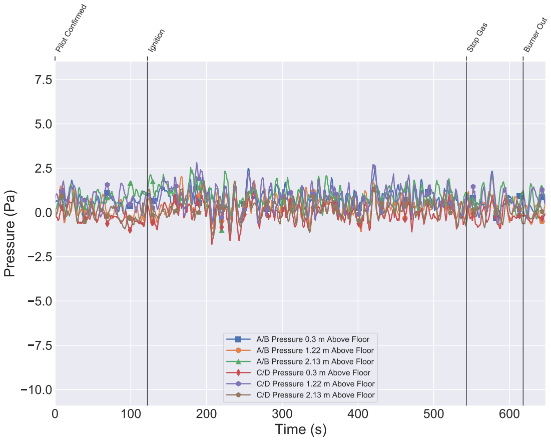

(f) Basement Pressures

Figure 5.63: Experiment 11 Pressures

The concentrations of oxygen and carbon dioxide are provided for the three bedrooms and the basement in Figure 5.64. The data presented here behaves in a similar manner to that presented for Experiments 8 and 10.

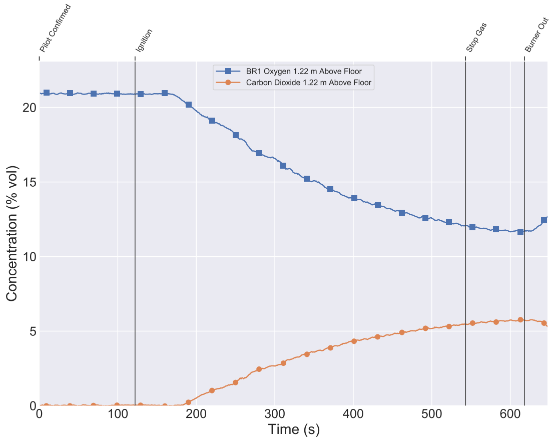

(a) Bedroom 1 Gases

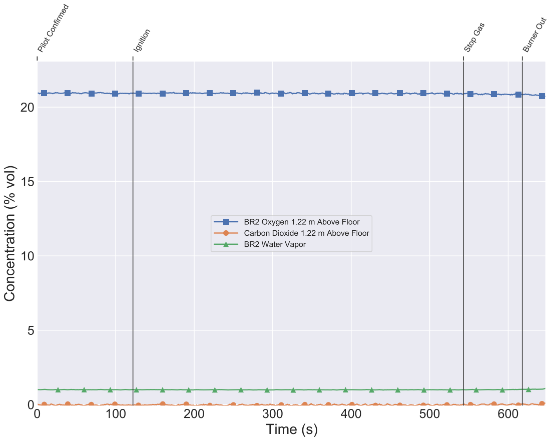

(b) Bedroom 2 Gases

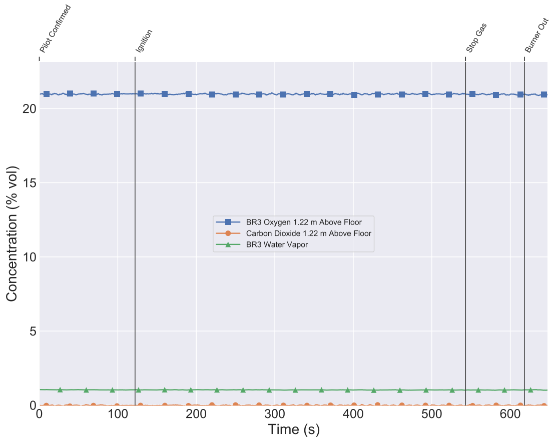

(c) Bedroom 3 Gases

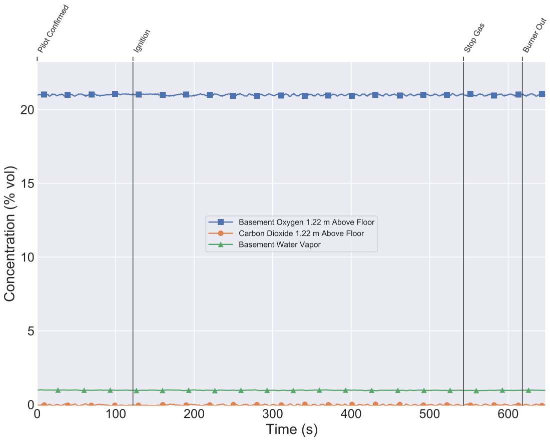

(d) Basement Gases

Figure 5.64: Experiment 11 Gases

Figure 5.65 shows the temperatures of the supply and return vents in the structure. Even with the inclusion of the louver vent into bedroom 3, the vent temperatures for all the bedrooms displayed similar data trends to that of Experiment

10. With the exception of the living room front door supply vent temperature, all supply and return vents in the structure behaved in the same manner as those of Experiments 8 and 10. The front door supply vent temperature did not show any effect from the living room temperature.

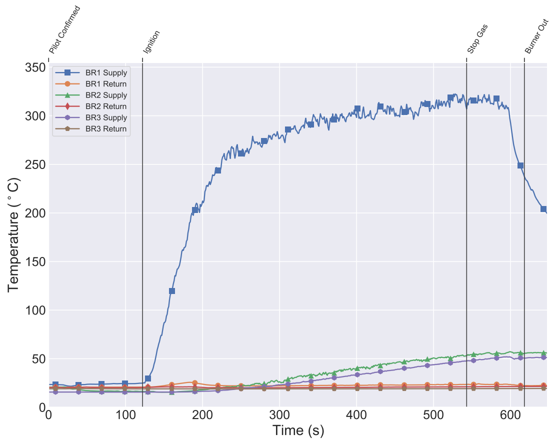

(a) Bedroom Vent Temperatures

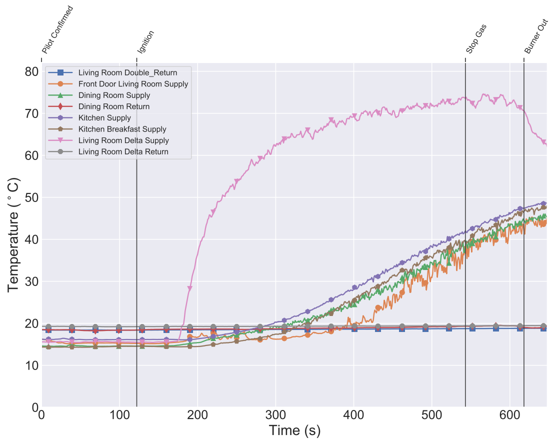

(b) First Floor Vent Temperatures

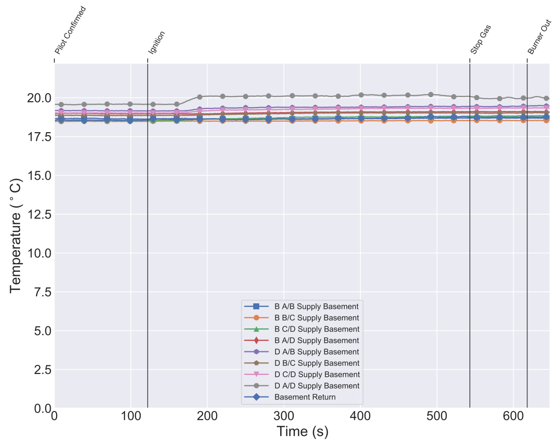

(c) Basement Vent Temperatures

Figure 5.65: Experiment 11 Vent Temperatures

The temperature and velocity within the supply duct for this experiment are provided in Figure 5.66. From Figure 5.66(a) it can be seen that the temperature

within the duct follows a similar trend to Experiments 8 and 10. The supply vent velocity appears to show a constant flow of air in the supply vent from the first floor to the basement. While the magnitude of this flow varies, it appears to fluctuate mostly between 0 and 1.5 m/s.

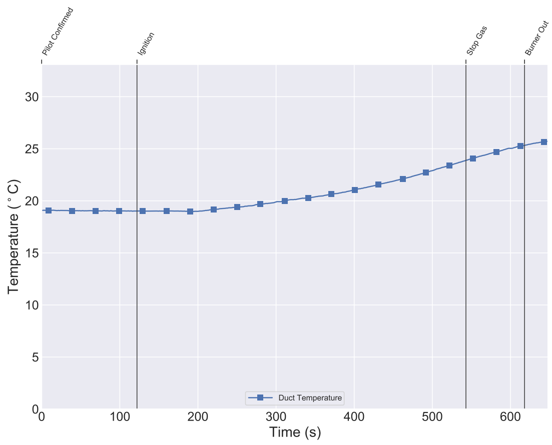

(a) Supply Duct Temperature

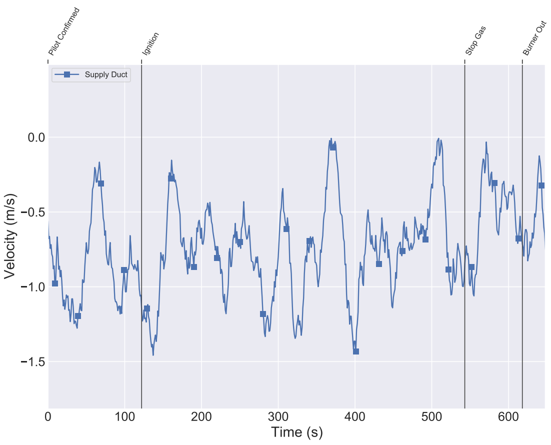

(b) Supply Duct Velocity

Figure 5.66: Experiment 11 Supply Duct Temperature and Velocity

5.1.12 Experiment 12

Gas Burner Experiment 12 was performed with the HVAC system off. The flow rate of propane to the gas burner was controlled so that a heat release rate of approximately 121 kW was produced. The door to bedroom 3 was closed. The doors to bedroom 1, bedroom 2 and the staircase door to the basement were open. In this experiment, the front door was the only exertior vent that was open. Gas concentration measurements on the first floor were taken in bedroom 1, bedroom 2, and bedroom 3. Experiment 12 was performed to observe the differences in how heat and fire gases were transported within the structure as a result of the front door

being open. Comparisons will be made to Experiment 1 to discuss any differences. The events of interest for Experiment 12 are presented in Table 5.11.

Table 5.12: Experiment 12 Events

Event	Elapsed Time (seconds)
Pilot Confirmed	0
Ignition	97
Stop Gas	999
Burner Out	1071

The time versus temperature graphs for the first floor are provided in Figure 5.67. Comparison of first floor temperatures to those recorded in Experiment 1 show no effects from the front door being open.

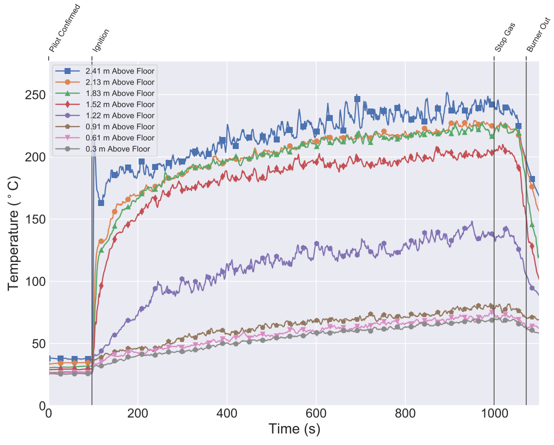

(a) Bedroom 1 Temperatures

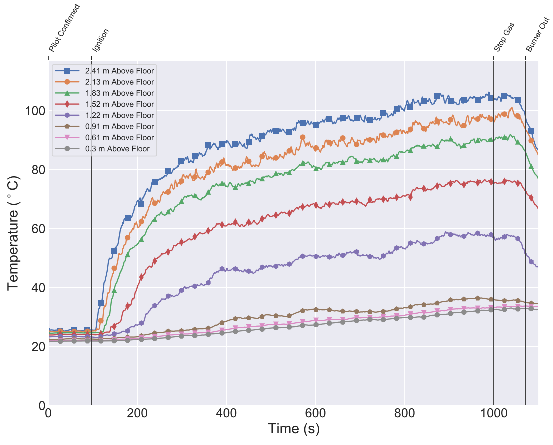

(b) Bedroom 2 Temperatures

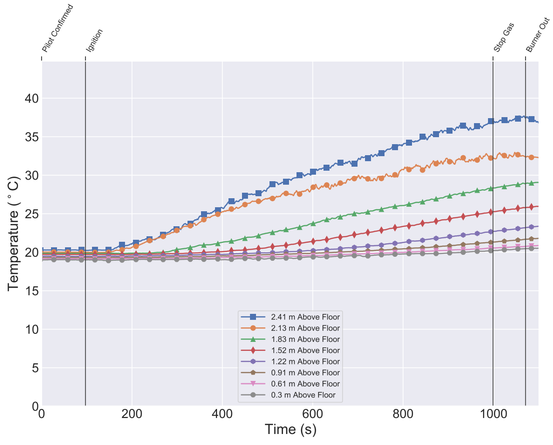

(c) Bedroom 3 Temperatures

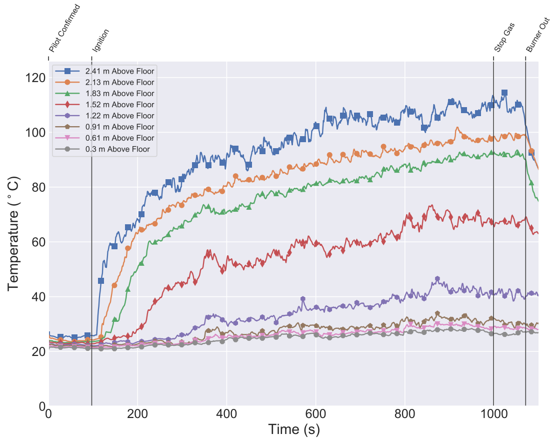

(d) Living Room Temperatures

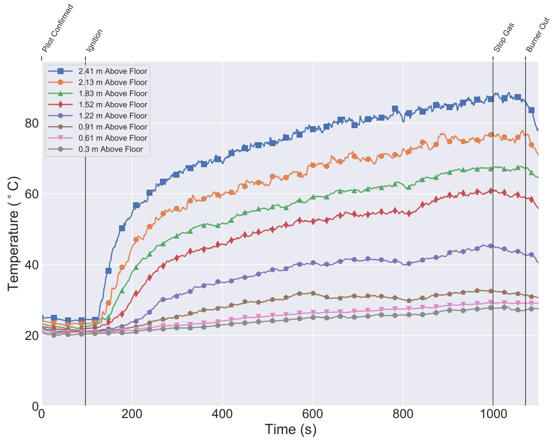

(e) Dining Room Temperatures

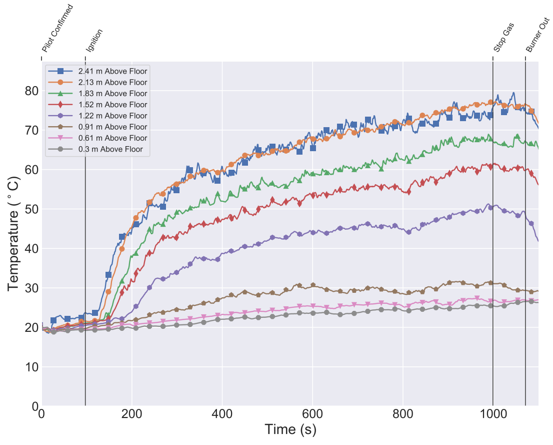

(f) Kitchen Temperatures

Figure 5.67: Experiment 12 First Floor Temperatures

The time versus temperature graphs for the four basement quadrants are provided in Figure 5.68. No notable temperature changes occurred in the basement during this experiment.

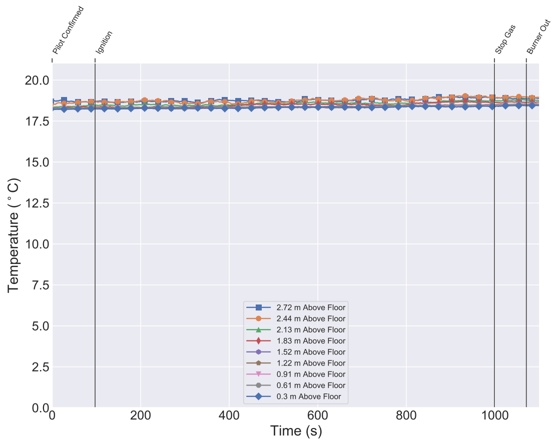

(a) Basement A Quadrant Temperatures

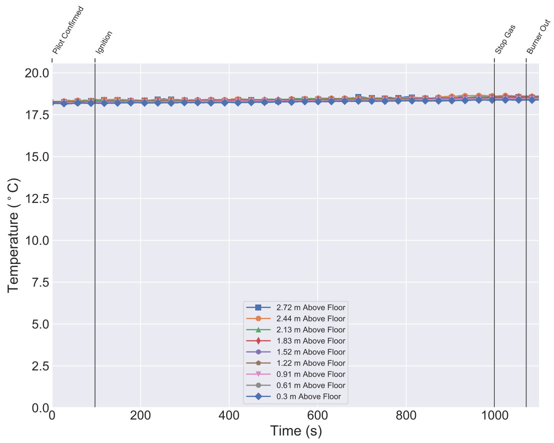

(b) Basement B Quadrant Temperatures

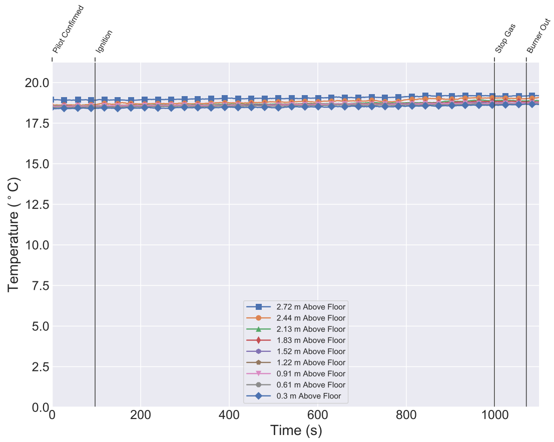

(c) Basement C Quadrant Temperatures

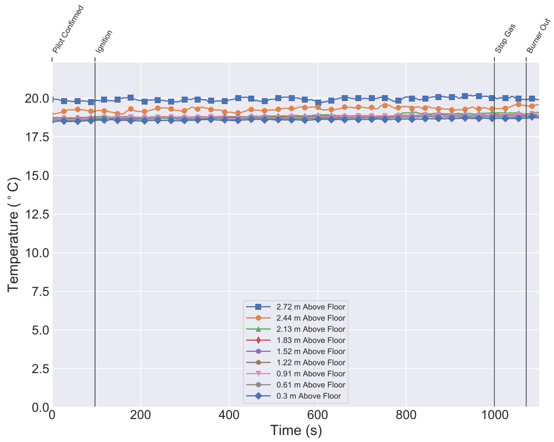

(d) Basement D Quadrant Temperatures

Figure 5.68: Experiment 12 Basement Temperatures

The pressures within each room are shown in Figure 5.69. Pressures on the first floor demonstrated a larger fluctation during the experiment than those in Experiment 1. While pressures in bedroom 1 had similar trends to that of Experiment

1, the pressures in bedrooms 2 and 3 fluctuated between 5 Pa below the ambient and 10 Pa above, with a spike greater than 15 Pa above the ambient. Pressures in the living room at the 2.13 m height remained between 2 and 4 Pa during the experiment with a spike up to 8 Pa towards the end of the experiment. Pressures at the 0.3 m and 1.2 m heights remained around ambient for the majority of the experiment. Pressures in the kitchen behaved similar to bedrooms 2 and 3. Pressures in the basement fluctuated mostly between 2 Pa above and below the ambient.

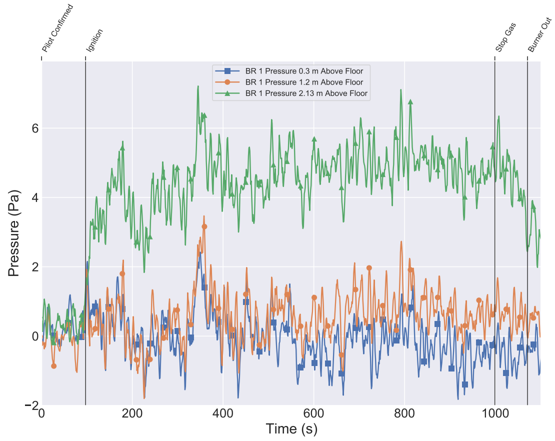

(a) Bedroom 1 Pressures

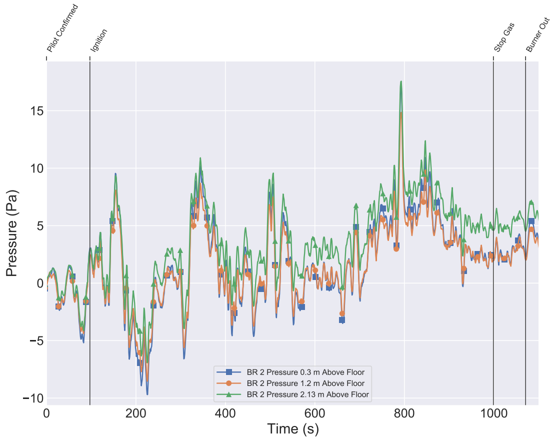

(b) Bedroom 2 Pressures

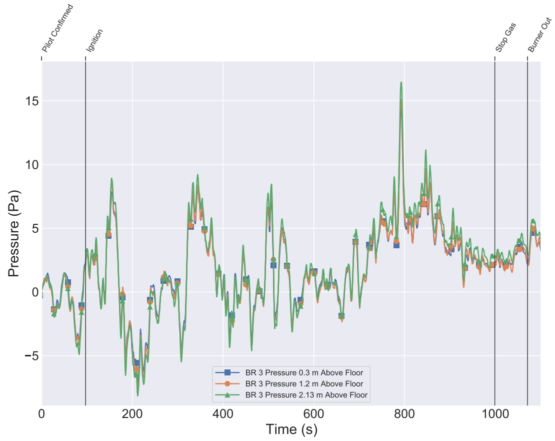

(c) Bedroom 3 Pressures

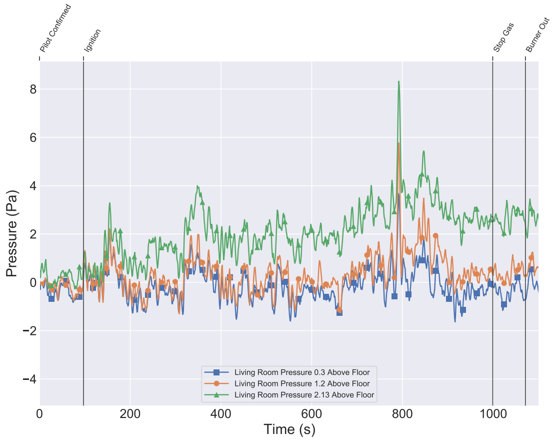

(d) Living Room Pressures

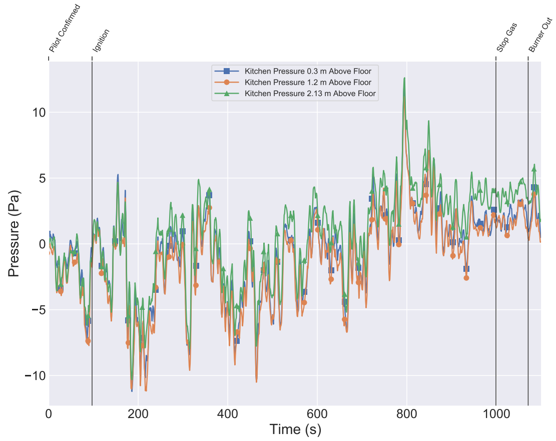

(e) Kitchen Pressures

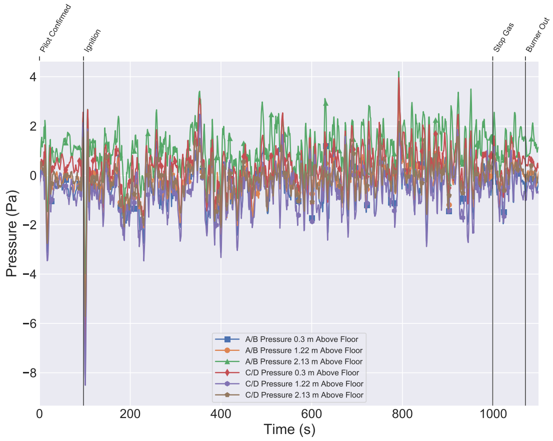

(f) Basement Pressures

Figure 5.69: Experiment 12 Pressures

The concentrations of oxygen and carbon dioxide are provided for the three bedrooms and the basement in Figure 5.70. Water vapor concentrations are included for bedroom 2, bedroom 3 and the basement. Oxygen concentrations in bedroom 1 only decreased by 1% during this experiment. The introduction of fresh air into the structure via the front door enabled the oxygen levels in the fire room to remain near normal levels. Carbon dioxide levels in bedroom 1 also remained near normal levels of 0%. Oxygen concentrations in bedroom 2 dropped to approximately 18% while carbon dioxide levels rose to 2%. Water vapor concentrations in bedroom 2 fluctuated with increases between 1% to 4%. Concentrations in bedroom 3 and the basement behaved the same as in Experiment 1.

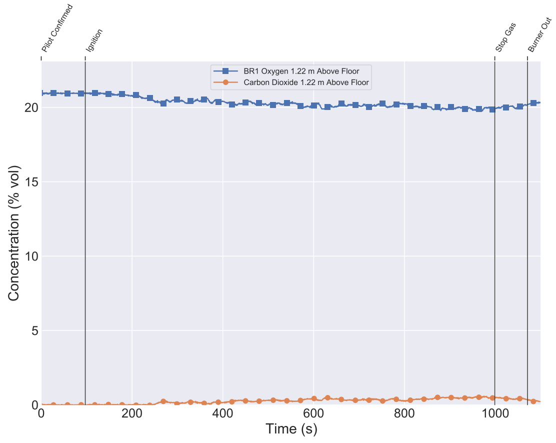

(a) Bedroom 1 Gases

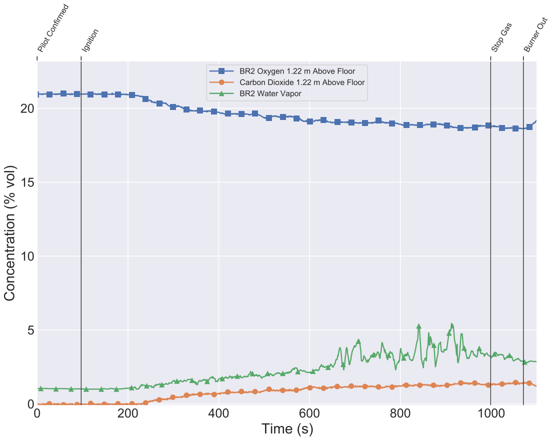

(b) Bedroom 2 Gases

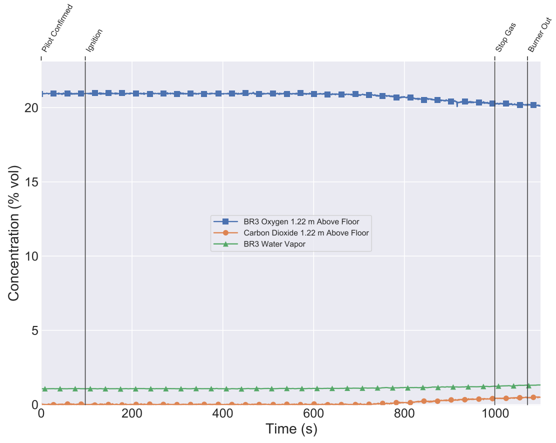

(c) Bedroom 3 Gases

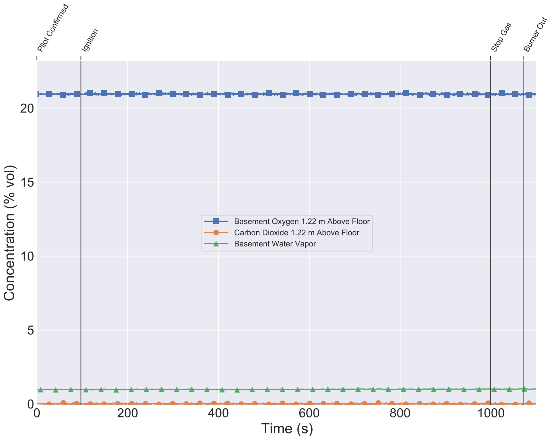

(d) Basement Gases

Figure 5.70: Experiment 12 Gases

Figure 5.71 shows the temperatures of the supply and return vents in the structure. Vent temperatures throughout the structure did not display any different trends than those identified in Experiment 1.

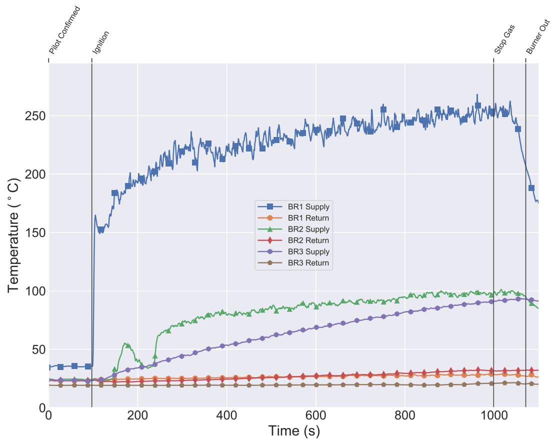

(a) Bedroom Vent Temperatures

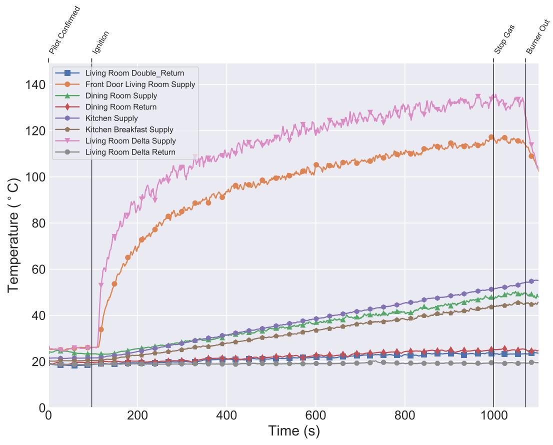

(b) First Floor Vent Temperatures

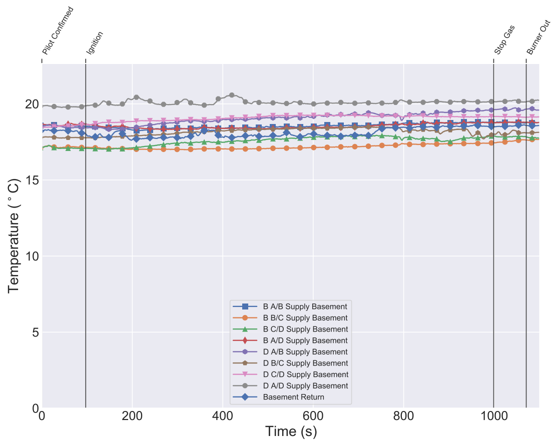

(c) Basement Vent Temperatures

Figure 5.71: Experiment 12 Vent Temperatures

The temperature and velocity within the supply duct for this experiment are provided in Figure 5.72. The temperature within the supply duct trended with the data provided in Experiment 1. Velocity within the duct fluctuated in a similar manner with Experiment 1.

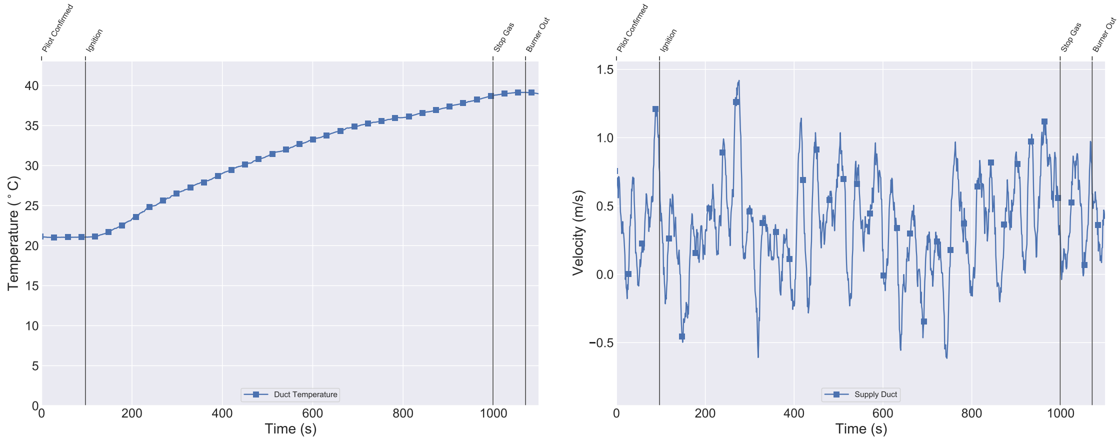

(a) Supply Duct Temperature

(b) Supply Duct Velocity

Figure 5.72: Experiment 12 Supply Duct Temperature and Velocity

5.1.13 Experiment 13

Gas Burner Experiment 13 was performed with the HVAC system set to A/C. The flow rate of propane to the gas burner was controlled so that a heat release rate of approximately 121 kW was produced. The doors to bedroom 1, bedroom 2 and the basement were open. The door to bedroom 3 was closed. The front door was the only exterior vent open for this experiment. Gas concentration measurements on the first floor were taken in bedroom 1, bedroom 2, and bedroom 3. Experiment 13 was performed to observe the differences in how heat and fire gases were transported within the structure as a result of the front door being open and the HVAC system set to A/C. Comparisons will be made to Experiments 2 and 12 to highlight effects of the open front door. The events of interest for Experiment 13 are presented in Table 5.13.

Table 5.13: Experiment 13 Events

Event	Elapsed Time (seconds)
Pilot Confirmed	0
Ignition	123
Stop Gas	1024
Burner Out	1107

The time versus temperature graphs for the first floor are provided in Figure 5.73. Comparison of first floor temperatures to Experiment 2 temperatures yields similar trends and values. Temperatures also trended with the data observed in Experiment 12 with the exception of bedroom 3 which was held at a lower temperature as a result of the A/C.

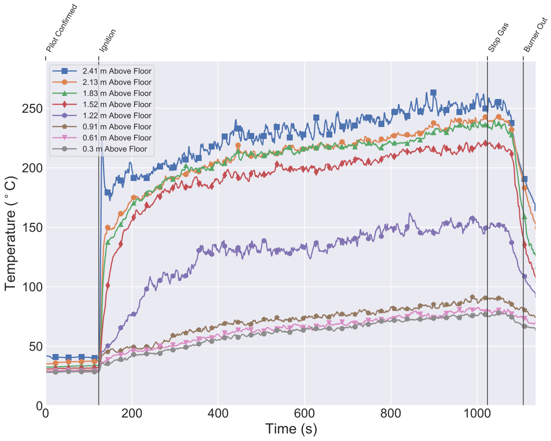

(a) Bedroom 1 Temperatures

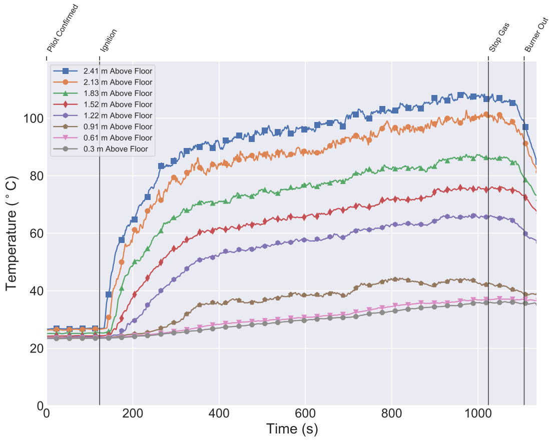

(b) Bedroom 2 Temperatures

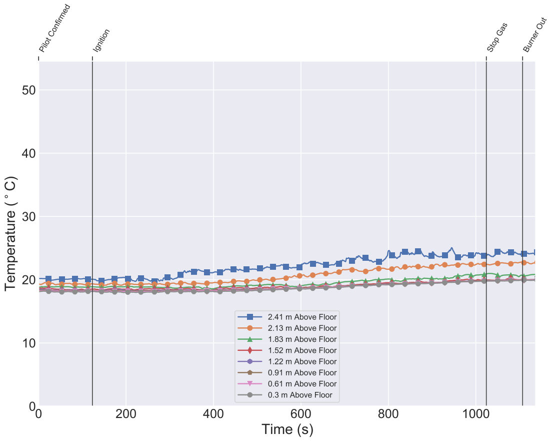

(c) Bedroom 3 Temperatures

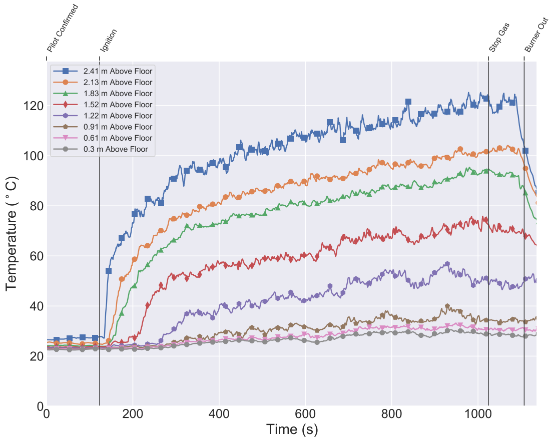

(d) Living Room Temperatures

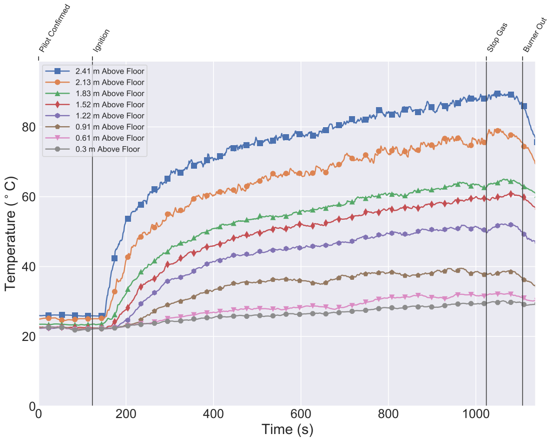

(e) Dining Room Temperatures

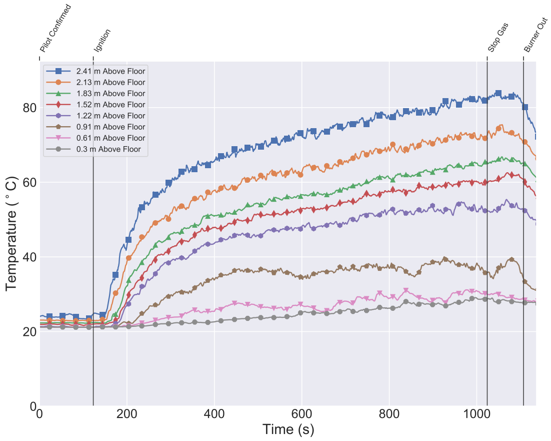

(f) Kitchen Temperatures

Figure 5.73: Experiment 13 First Floor Temperatures

The time versus temperature graphs for the four basement quadrants are provided in Figure 5.74. No notable temperature changes occurred in the basement during this experiment.

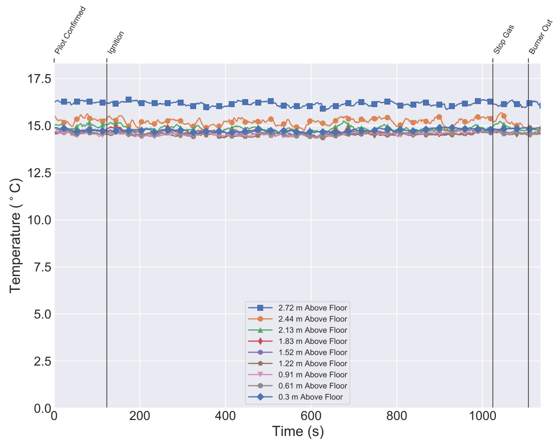

(a) Basement A Quadrant Temperatures

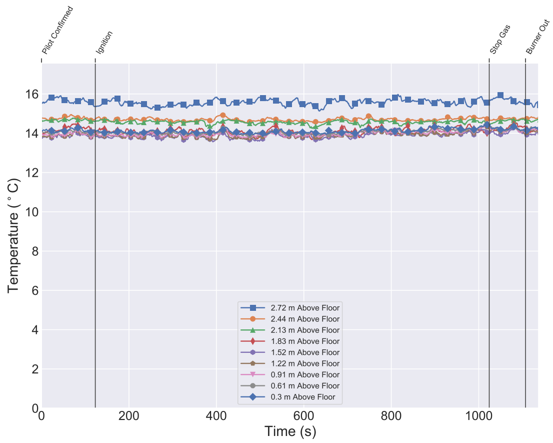

(b) Basement B Quadrant Temperatures

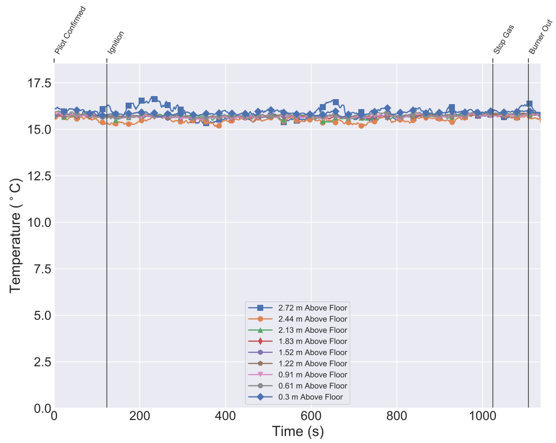

(c) Basement C Quadrant Temperatures

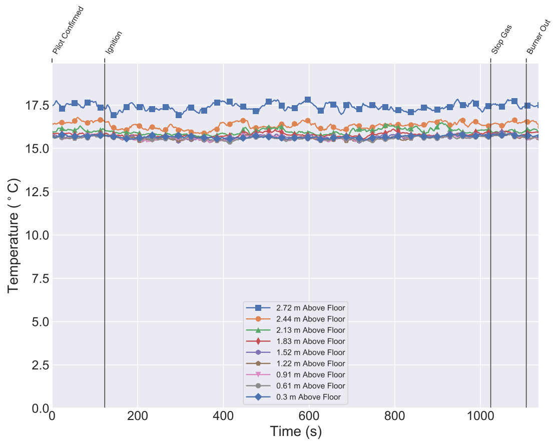

(d) Basement D Quadrant Temperatures

Figure 5.74: Experiment 13 Basement Temperatures

The pressures within each room are shown in Figure 5.75. Pressures in bedroom 1 behaved in a similar manner to that of Experiments 2 and 12. In contrast to those experiments, the pressures in bedroom 2 showed continuous positive pressure

increases up to 10 Pa during the experiment. Pressures in bedroom 3 fluctuated between 2.5 Pa and 10 Pa during the experiment. Pressures in the living room displayed similar trends to those seen in Experiment 12. Pressures in the kitchen exceeded the ambient by approximately 10 to 15 Pa. Pressures in the basement mainly hovered around 2 to 4 Pa during the experiment.

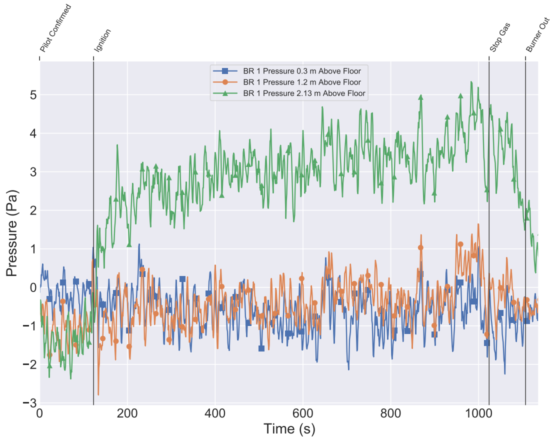

(a) Bedroom 1 Pressures

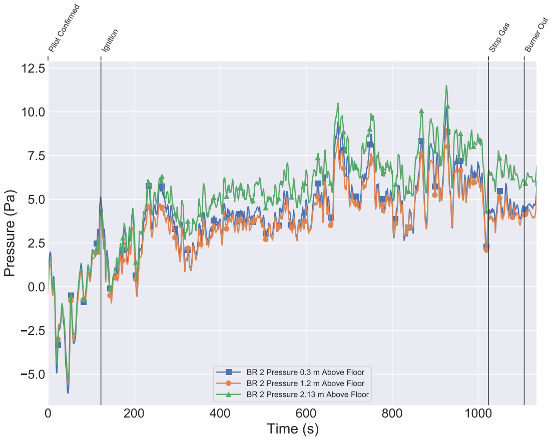

(b) Bedroom 2 Pressures

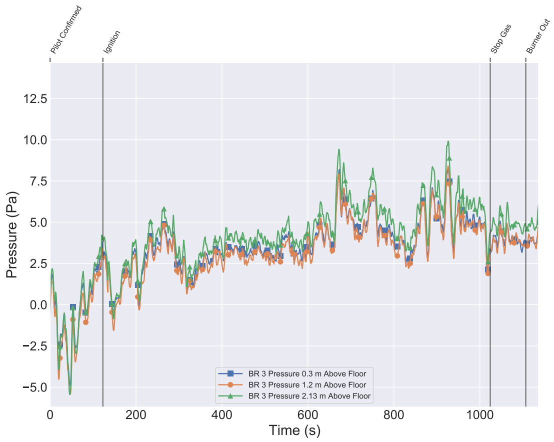

(c) Bedroom 3 Pressures

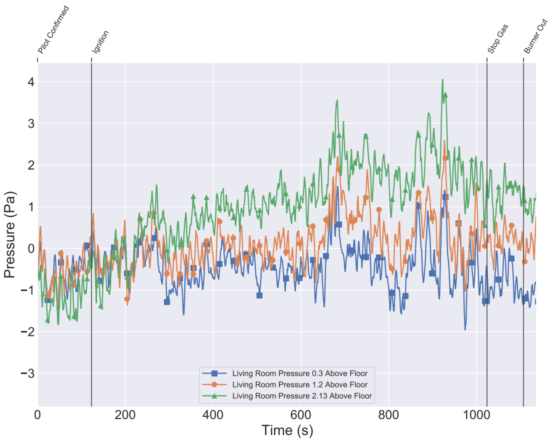

(d) Living Room Pressures

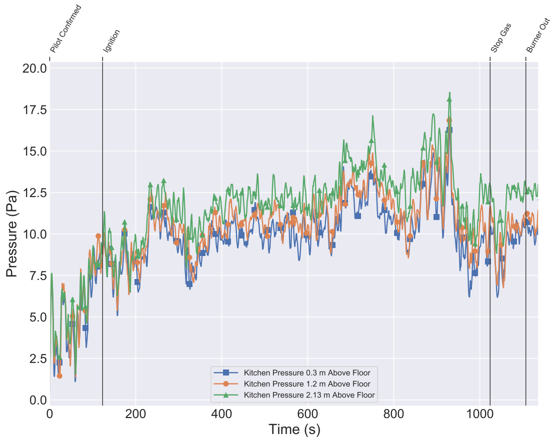

(e) Kitchen Pressures

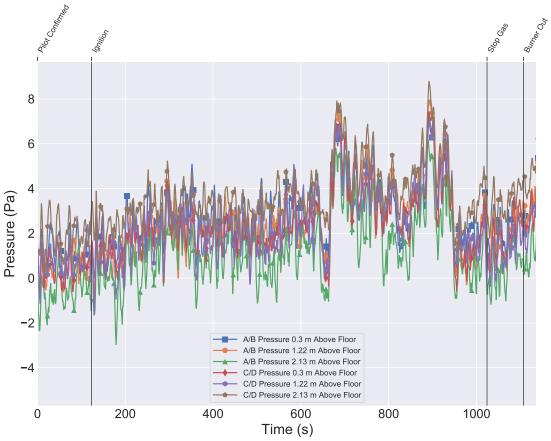

(f) Basement Pressures

Figure 5.75: Experiment 13 Pressures

The concentrations of oxygen and carbon dioxide are provided for the three bedrooms and the basement in Figure 5.76. Water vapor concentrations are included for bedroom 2, bedroom 3 and the basement. Gas and water vapor concentrations observed in Experiment 13 are similar to the trends and values seen in Experiment 12.

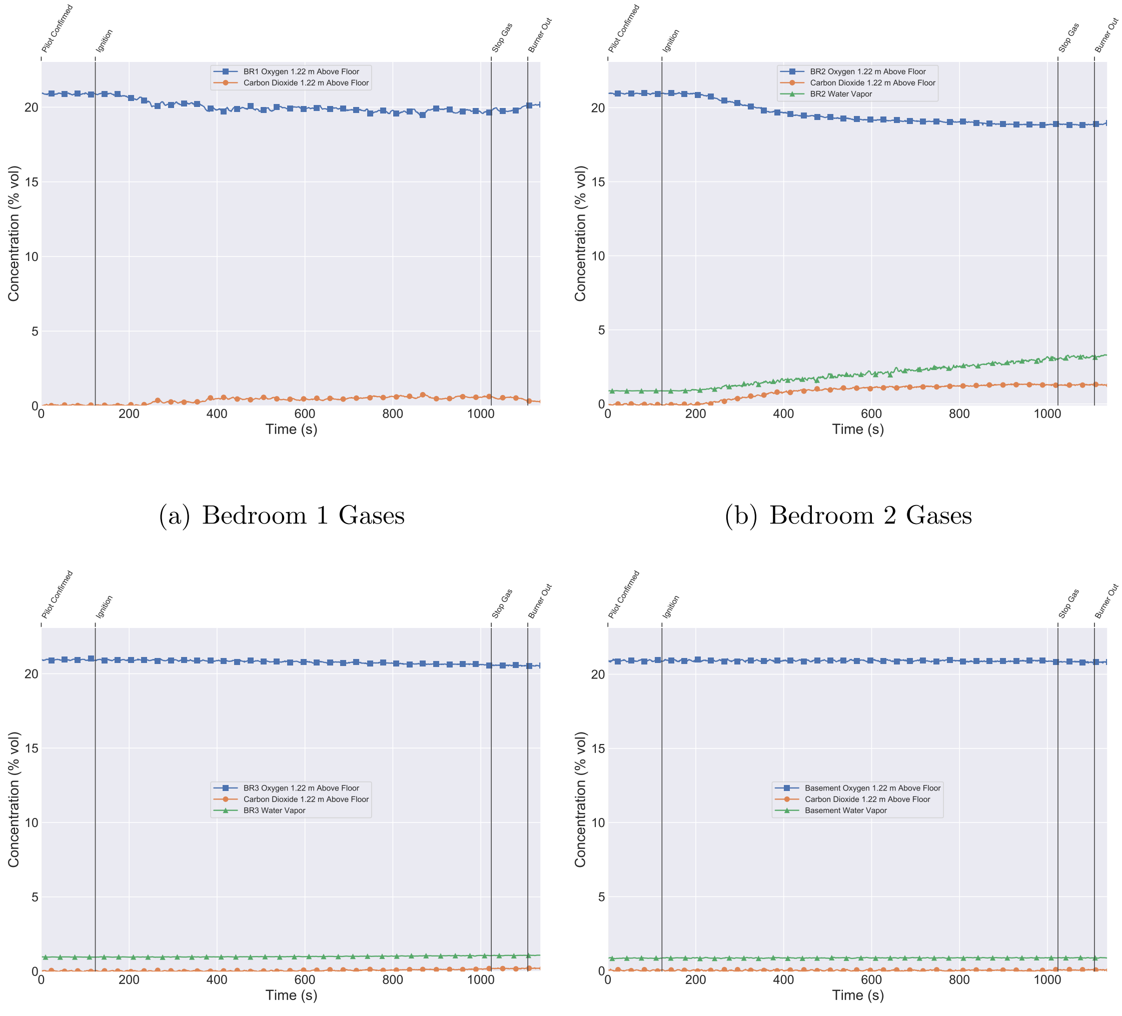

(a) Bedroom 1 Gases

(b) Bedroom 2 Gases

(c) Bedroom 3 Gases

(d) Basement Gases

Figure 5.76: Experiment 13 Gases

Figure 5.77 shows the temperatures of the supply and return vents in the

structure. Figure 5.77(a) shows the vent temperatures for the bedrooms. In contrast to what was observed in Experiment 2, the supply vent temperatures never experienced a sharp increase in temperature. A possible reason for this is that the pressures generated within the HVAC supply duct were never surpassed by the pressures being generated in the structure. The open front door could have allowed for this to occur. This behavior is seen in all first floor supply vent locations, thus supporting the conclusion that the HVAC set to A/C was able to limit the heat transfer through the supply ducts. Similar to the other experiments, there was no notable temperature change at the basement vents.

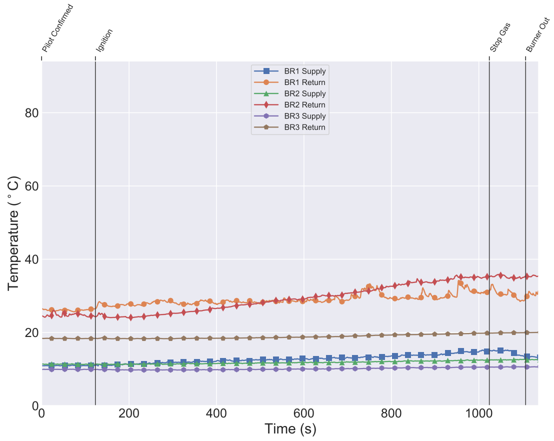

(a) Bedroom Vent Temperatures

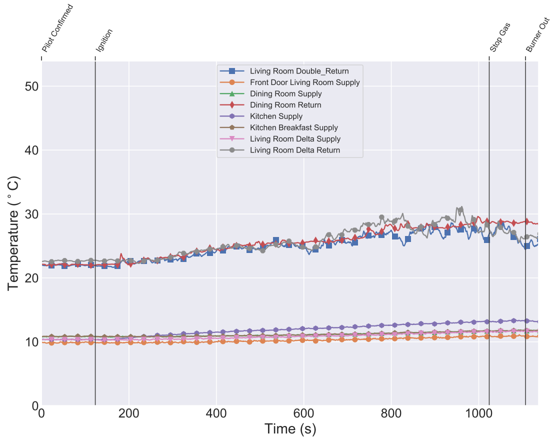

(b) First Floor Vent Temperatures

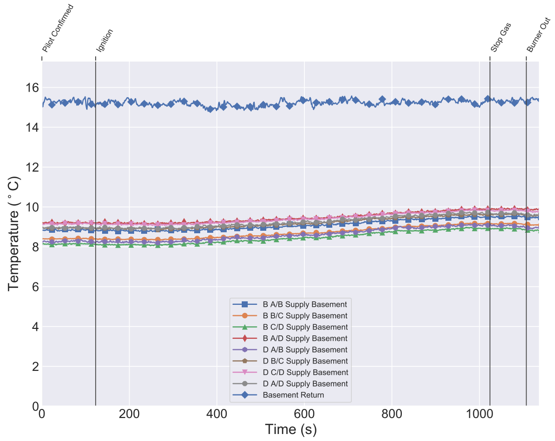

(c) Basement Vent Temperatures

Figure 5.77: Experiment 13 Vent Temperatures

The temperature and velocity within the supply duct for this experiment are provided in Figure 5.78. From Figure 5.78(a), it can be seen that the temperature within the supply duct showed minimal change. The velocity within the supply duct fluctuated between 0 and 1 m/s both from the basement to the first floor and the other way during the experiment.

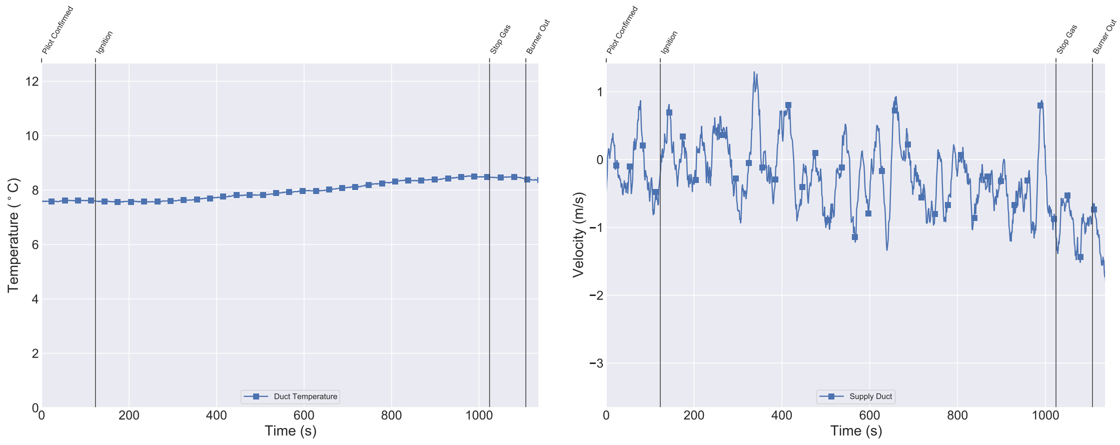

(a) Supply Duct Temperature

(b) Supply Duct Velocity

Figure 5.78: Experiment 13 Supply Duct Temperature and Velocity

5.1.14 Experiment 14

Gas Burner Experiment 14 was performed with the HVAC system set to A/C with a higher activating temperature for cooling. The flow rate of propane to the gas burner was controlled so that a heat release rate of approximately 121 kW was produced. The doors to bedroom 1, bedroom 2 and the basement were open. The door to bedroom 3 was closed. All exterior vents to the structure were closed. For this experiment, gas concentration measurements on the first floor were taken in bedroom 1, bedroom 2, and bedroom 3. Experiment 14 was performed to observe if there were any effects of having the HVAC cooling system set to activate when a higher temperature setpoint was reached. Comparisons will be drawn to Experiment 2. The events of interest for Experiment 14 are presented in Table 5.14.

Table 5.14: Experiment 14 Events

Event	Elapsed Time (seconds)
Pilot Confirmed	0
Ignition	102
Stop Gas	823
Burner Out	904

The time versus temperature graphs for the first floor are provided in Figure 5.79. Temperatures throughout the first floor trended similar to the data of Experiment 2. By observation of Figure 5.79(c), it can be seen that there is a slight temperature increase followed by a decrease when the HVAC A/C was able to build up enough pressure to push cooler air through the ductwork.

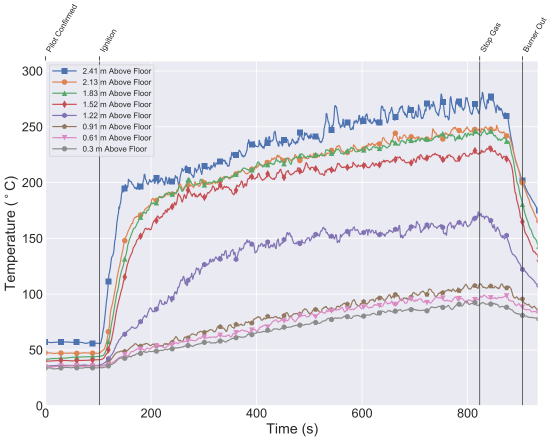

(a) Bedroom 1 Temperatures

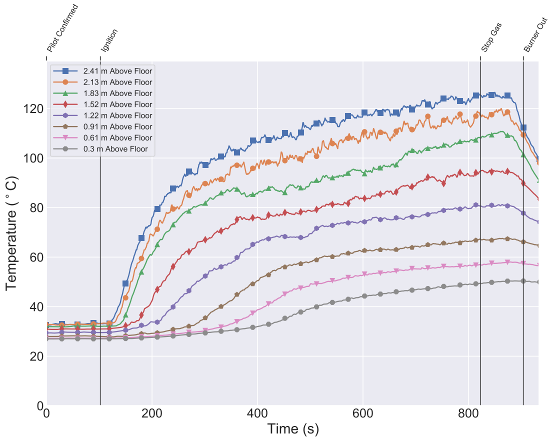

(b) Bedroom 2 Temperatures

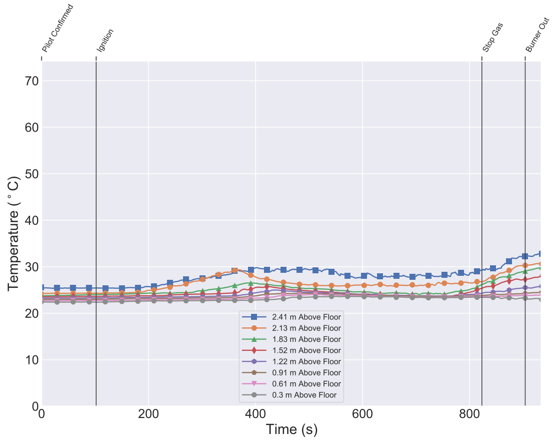

(c) Bedroom 3 Temperatures

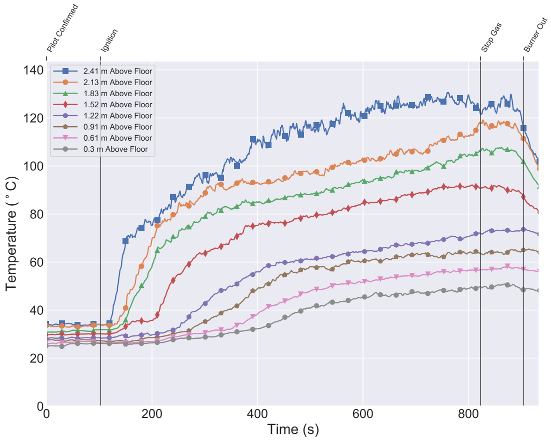

(d) Living Room Temperatures

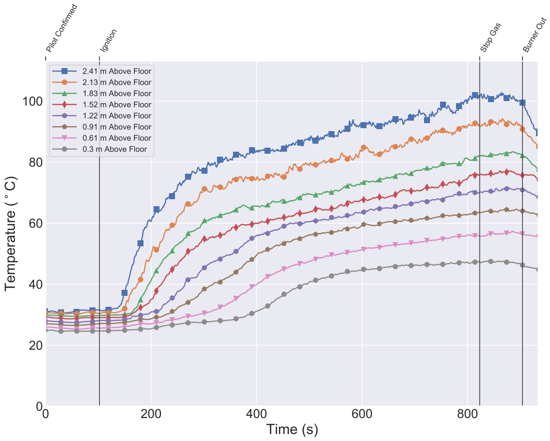

(e) Dining Room Temperatures

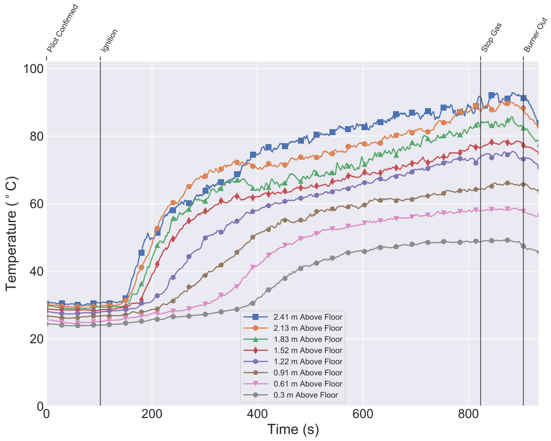

(f) Kitchen Temperatures

Figure 5.79: Experiment 14 First Floor Temperatures

The time versus temperature graphs for the four basement quadrants are provided in Figure 5.80. A slight temperature decrease can be seen when the A/C was able to cool the air in the supply ducts.

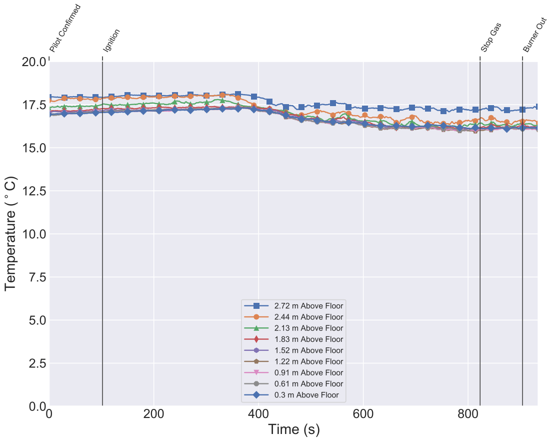

(a) Basement A Quadrant Temperatures

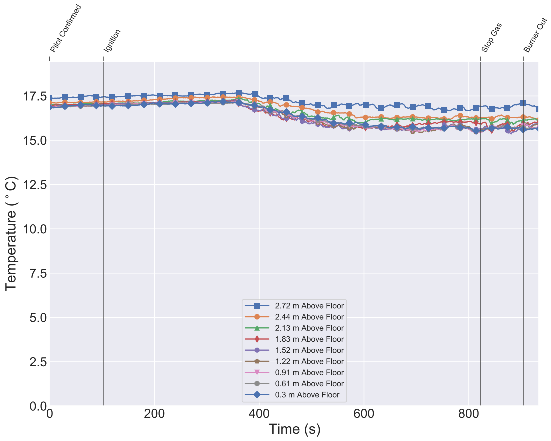

(b) Basement B Quadrant Temperatures

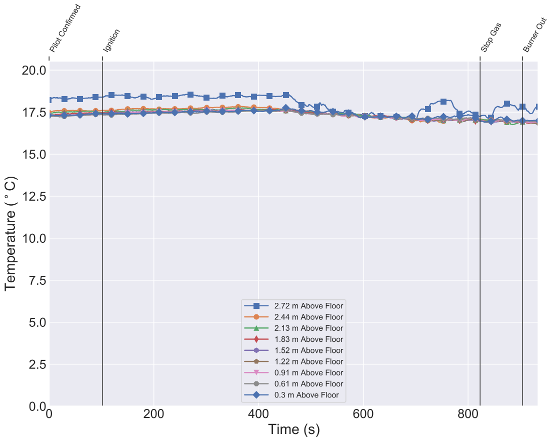

(c) Basement C Quadrant Temperatures

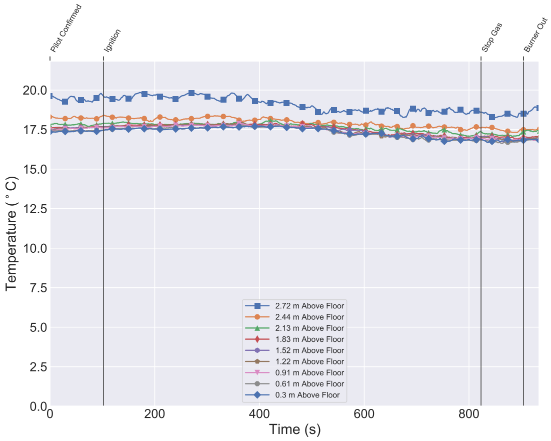

(d) Basement D Quadrant Temperatures

Figure 5.80: Experiment 14 Basement Temperatures

The pressures within each room are shown in Figure 5.81. Pressures in the bedrooms all trended similar to those observed in Experiment 2 with the exception of larger pressure spikes observed in bedrooms 2 and 3 following ignition. Similarly,

pressures in the living room and kitchen followed similar trends to Experiment 2 with larger post ignition pressure spikes. Pressures in the basement showed an increase from around 0 Pa to around 8 Pa after the HVAC system was activated.

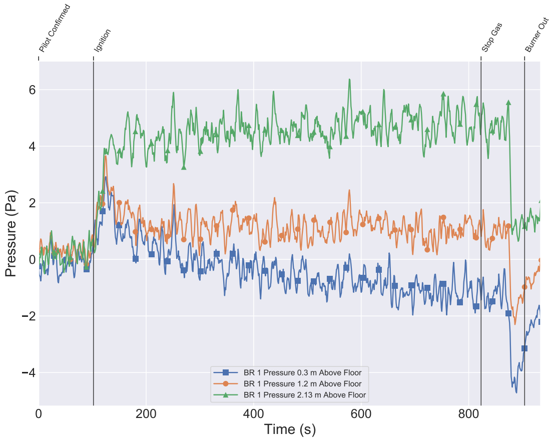

(a) Bedroom 1 Pressures

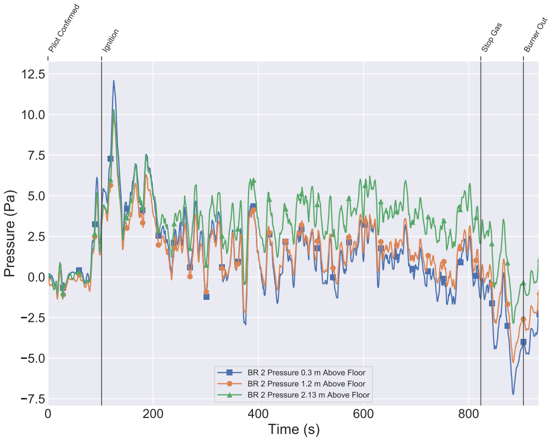

(b) Bedroom 2 Pressures

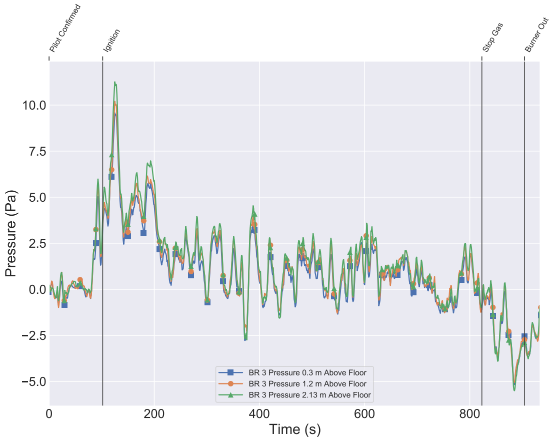

(c) Bedroom 3 Pressures

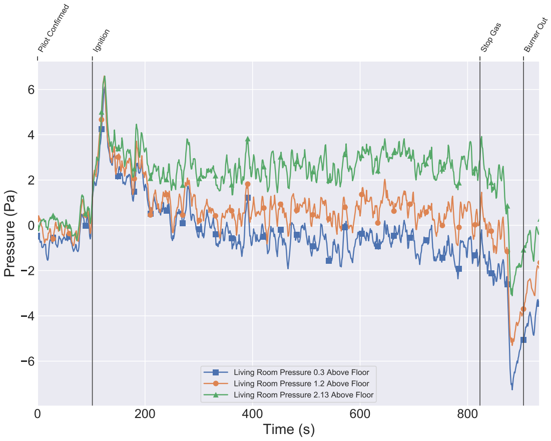

(d) Living Room Pressures

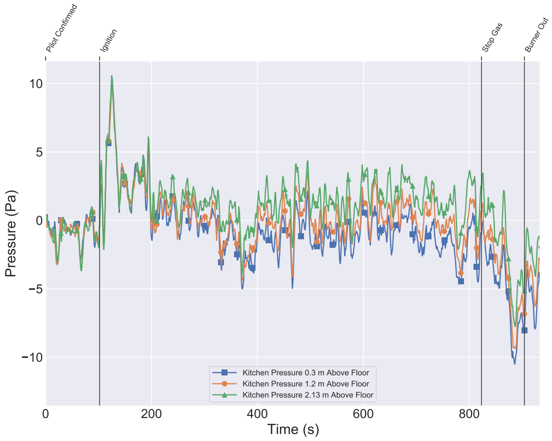

(e) Kitchen Pressures

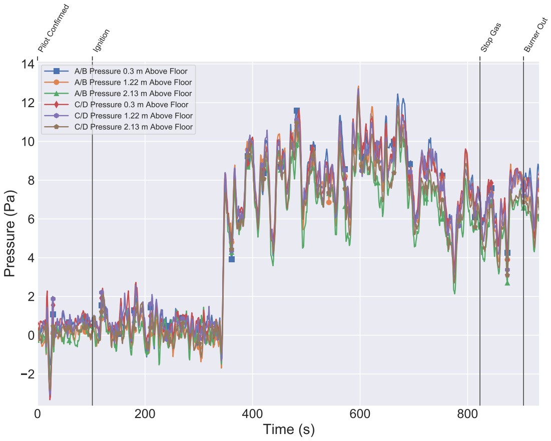

(f) Basement Pressures

Figure 5.81: Experiment 14 Pressures

The concentrations of oxygen and carbon dioxide are provided for the three bedrooms and the basement in Figure 5.82. Water vapor concentrations are included for bedroom 2, bedroom 3 and the basement. Data collected in Experiment 14 was similar to the trends and values observed in Experiment 2.

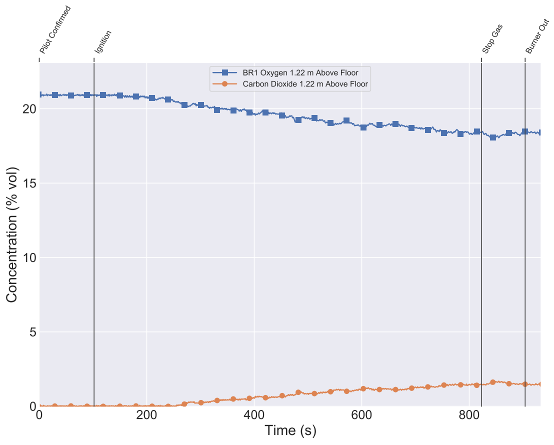

(a) Bedroom 1 Gases

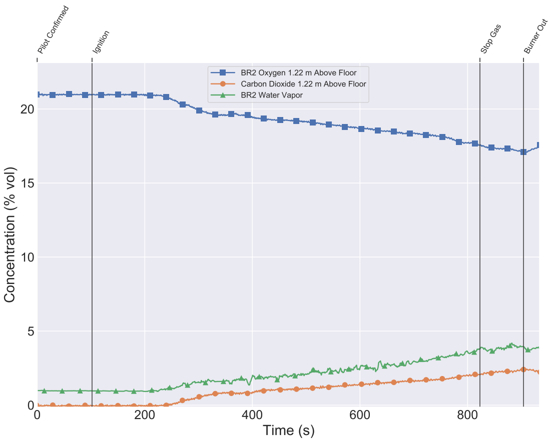

(b) Bedroom 2 Gases

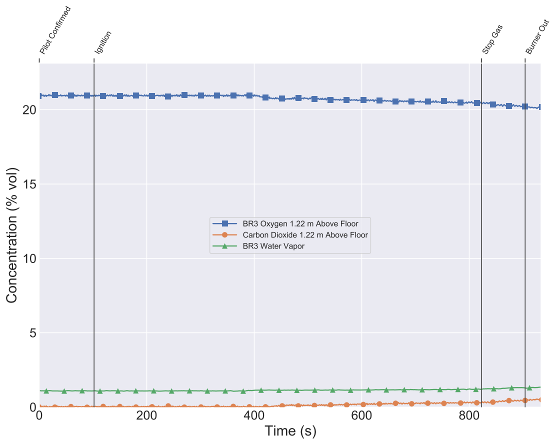

(c) Bedroom 3 Gases

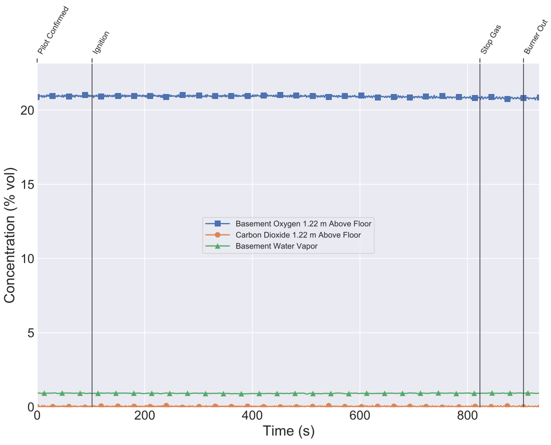

(d) Basement Gases

Figure 5.82: Experiment 14 Gases

Figure 5.83 shows the temperatures of the supply and return vents in the structure. Figure 5.83(a) displays the bedroom vent temperatures. From this figure

it can easily be seen that the temperature at the supply vent in bedroom 1 increased until the HVAC system was able to overcome the pressure being generated by fire. The HVAC system was able to maintain a greater pressure than bedroom 1 for a couple hundred seconds before the fire was able to heat the supply vent again. A similar trend can be seen in bedrooms 2 and 3. By observation of Figure 5.83(b), a similar trend can be seen for the living room supply vents. Observation of Figure 5.83(c) shows both when the HVAC system initially activated and when the system tried to compensate for the continual heat rise past the set point.

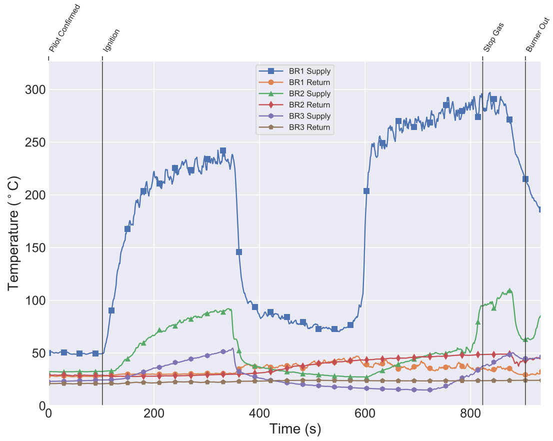

(a) Bedroom Vent Temperatures

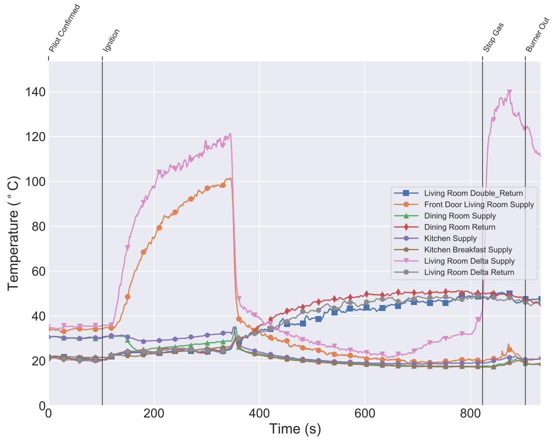

(b) First Floor Vent Temperatures

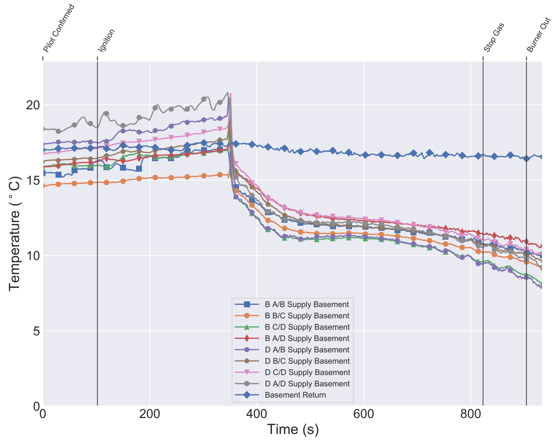

(c) Basement Vent Temperatures

Figure 5.83: Experiment 14 Vent Temperatures

The temperature and velocity within the supply duct for this experiment are provided in Figure 5.84. From Figure 5.84(a) it can be seen that the temperature within the supply duct increased until the HVAC system activated, which caused a steep decline in temperature. The supply vent velocity shows a sharp positive increase when the HVAC system activated and cool air was being pushed up to the first floor.

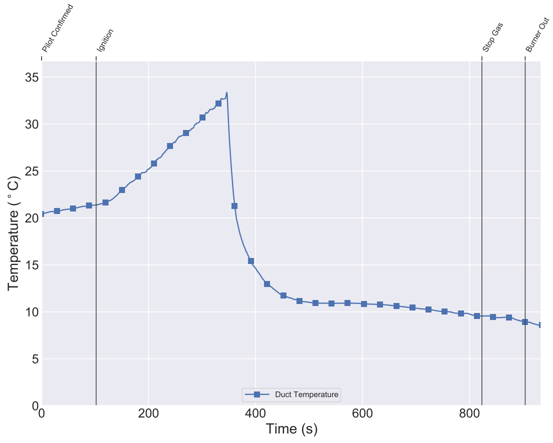

(a) Supply Duct Temperature

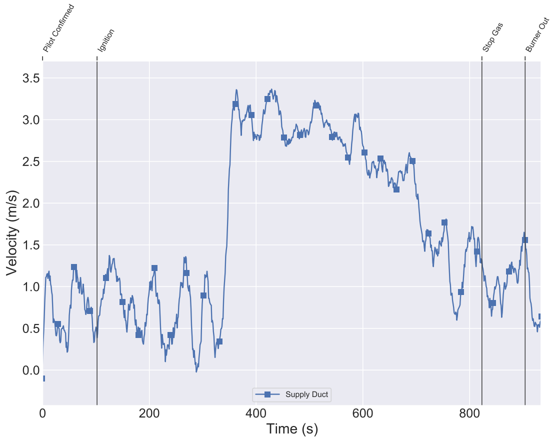

(b) Supply Duct Velocity

Figure 5.84: Experiment 14 Supply Duct Temperature and Velocity

5.2 Living Room Experiments

Gas Burner Experiments 15 through 21 were performed with the gas burner located in the living room. For all living room experiments, the door to bedroom 1 and all exterior vents were closed and the door to bedroom 2 was open. Three gas concentration measurements were obtained on the first floor and one measurement in the basement. The basement gas concentration measurement was located on the D side of the structure. For experiments 20 and 21, louver vents were installed above the doorways to bedrooms 1 and 3. To protect the windows in the living room from failure due to repeated heat exposure and to reduce rehab time between experiments, durarock was placed inside the structure to protect the windows immediately exposed to the burner. The durarock was replaced as needed to protect the windows during all living room experiments.

5.2.1 Experiment 15

Gas Burner Experiment 15 was performed with the HVAC system off. The flow rate of propane to the gas burner was controlled so that a heat release rate of approximately 121 kW was produced. The stairwell door to the basement was open during this experiment. Gas concentration measurements on the first floor were taken in bedroom 2, bedroom 3 and the living room. Experiment 15 was performed as a baseline for how heat and fire gases were transported within the HVAC ductwork with the system turned off and a fire in the living room. The events of interest for Experiment 15 are presented in Table 5.15.

Table 5.15: Experiment 15 Events

Event	Elapsed Time (seconds)
Pilot Confirmed	0
Ignition	94
Stop Gas	1774
Burner Out	1860

The time versus temperature graphs for the first floor are provided in Figure 5.85. Temperatures in the living room increased approximately 150 °C at the 2.13 m height and approximately 20 °C at the 0.3 m height. Temperatures in bedroom 2 increased approximately 80 °C at the 2.13 m height and approximately 30 °C at the 0.3 m height. Temperatures in bedroom 1 rose approximately 50 °C at the 2.13 m height and approximately 15 °C at the 0.3 m height. In contrast to the temperatures in bedroom 1, the temperatures in bedroom 3 increased approximately 25 °C at the 2.13 m height and approximately 5 °C at the 0.3 m height. Tempera-

tures in the Dining Room increased approximately 100 °C at the 2.13 m height and approximately 40 °C at the 0.3 m height. Temperatures in the Kitchen increased approximately 90 °C at the 2.13 m height and approximately 40 °C at the 0.3 m height.

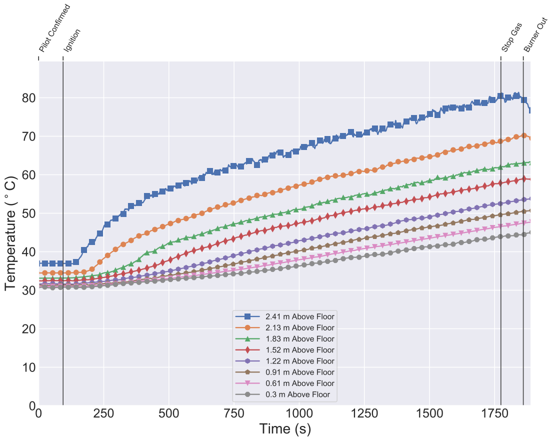

(a) Bedroom 1 Temperatures

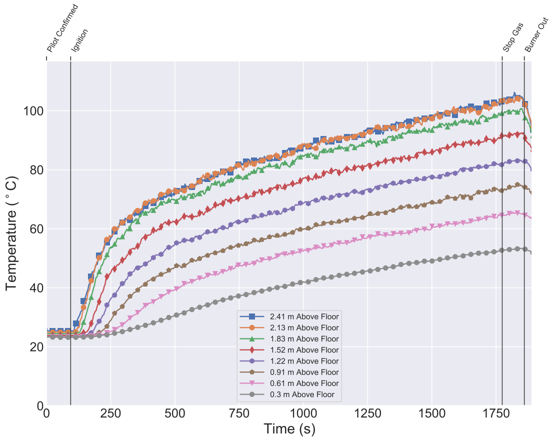

(b) Bedroom 2 Temperatures

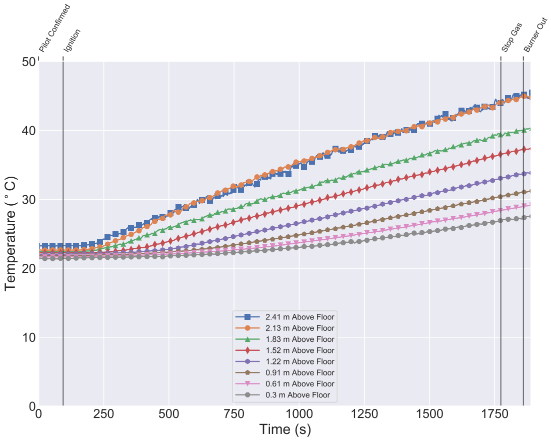

(c) Bedroom 3 Temperatures

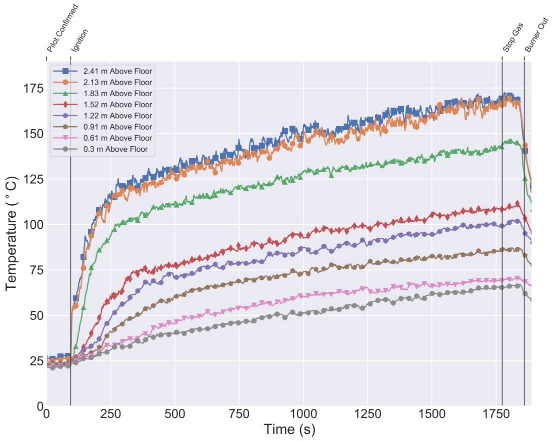

(d) Living Room Temperatures

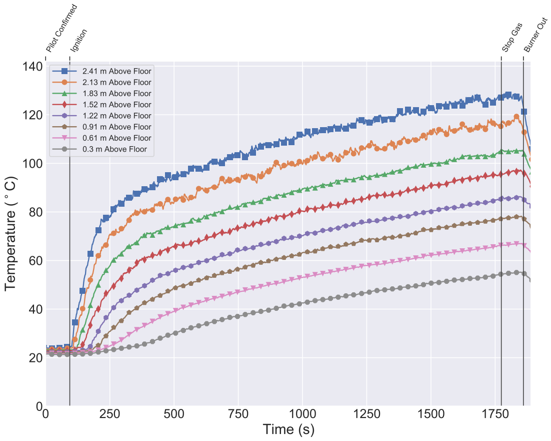

(e) Dining Room Temperatures

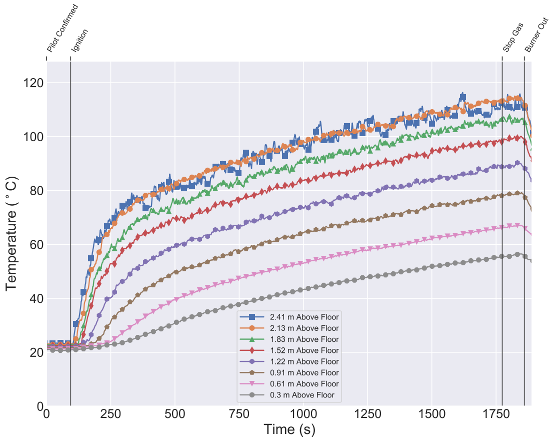

(f) Kitchen Temperatures

Figure 5.85: Experiment 15 First Floor Temperatures

The time versus temperature graphs for the four basement quadrants are provided in Figure 5.86. As was the case with other first floor burner experiments, there was very little change to temperatures in the basement. Over the course of Experiment 15, a slight positive slope can be seen, resulting in an increase of only a degree celcius.

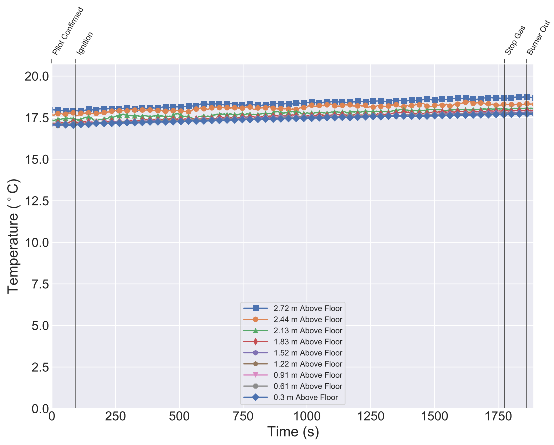

(a) Basement A Quadrant Temperatures

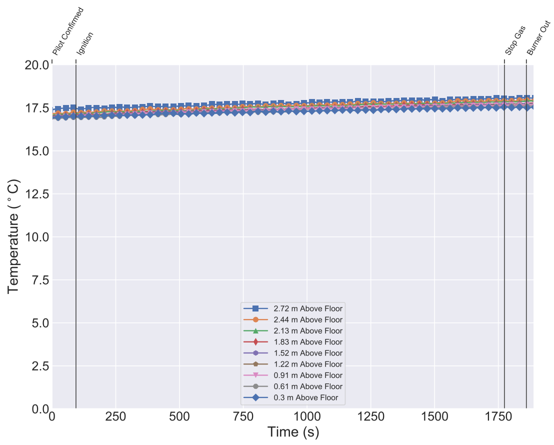

(b) Basement B Quadrant Temperatures

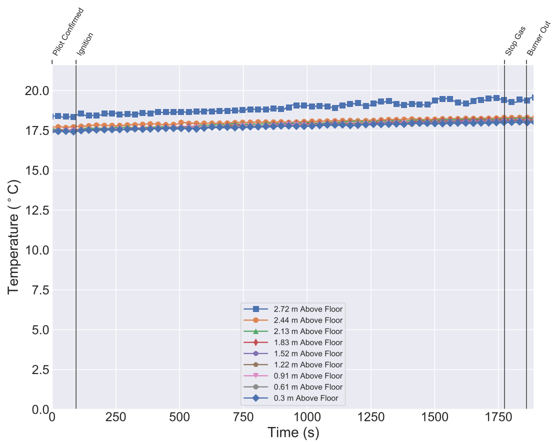

(c) Basement C Quadrant Temperatures

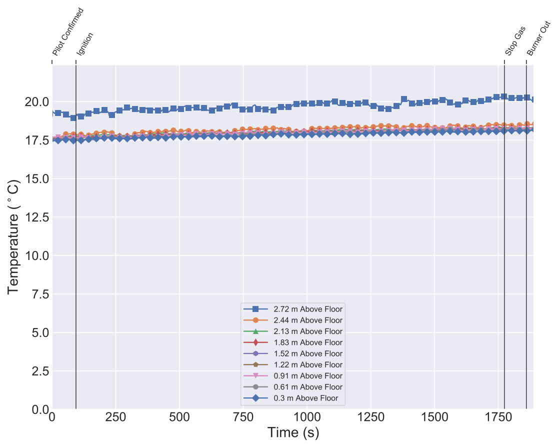

(d) Basement D Quadrant Temperatures

Figure 5.86: Experiment 15 Basement Temperatures

The pressures within each room are shown in Figure 5.87. Pressure in bedroom

1 at 2.13 m above the floor exceeded the ambient pressure by approximately 2 to 3 Pa while the burner was on. At 1.2 m above the floor pressure exceeded the ambient by approximately 1 to 2 Pa. At 0.3 m above the floor pressure remained at approximately ambient with a small spike following ignition. Pressure in bedroom 2 at 2.13 m above the floor exceeded the ambient pressure by approximately 4 to 6 Pa. At 1.2 m above the floor pressure exceeded the ambient by approximately 1 to 4 Pa. At 0.3 m above the floor pressure trended similar to that at the 1.2 m height. In bedroom 3 pressures fluctuated between 0 and 3 Pa above the ambient at all heights with a spike following ignition. Pressure in the living room at 2.13 m above the floor exceeded the ambient pressure by approximately 5 Pa while the burner was on. Pressure at the 1.2 m height fluctuated around 1 to 2 Pa above the ambient. Pressure at the 0.3 m height fluctuated around 0 to -1 Pa relative to the ambient pressure. Pressure in the kitchen at the 2.13 m height fluctuated mostly between 0 and 3 Pa with a high spike up to 4 Pa and a low spike down to -2 Pa. Pressures at the 1.2 m height and the 0.3 m height fluctuated between 1 and -2 Pa relative to the ambient with spikes as low as -5 Pa. Pressures in the basement held relatively constant around -1 pascal throughout the experiment.

The steady increase in the pressure at the 2.13 m height in bedroom 1 in conjunction with the greater temperature increase at the ceiling of bedroom 1 could indicate that there was more leakage into bedroom 1 than bedroom 3 from the living room. By observation of the pressure graphs it appears that the pressures in bedroom 1 are more similar to bedroom 2, which had an open bedroom door.

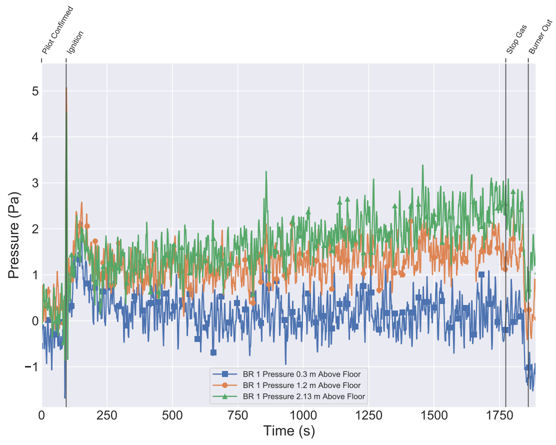

(a) Bedroom 1 Pressures

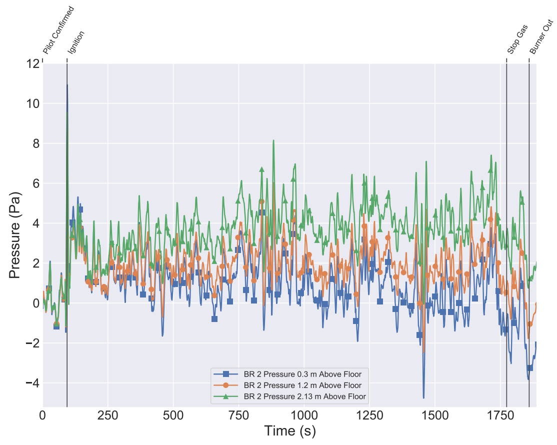

(b) Bedroom 2 Pressures

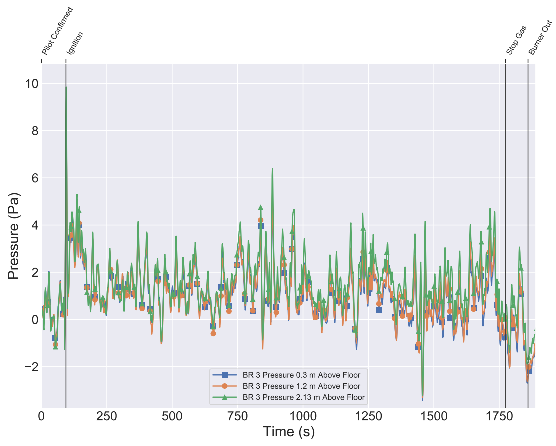

(c) Bedroom 3 Pressures

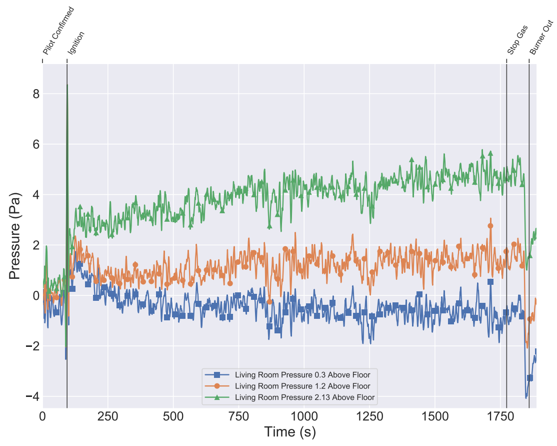

(d) Living Room Pressures

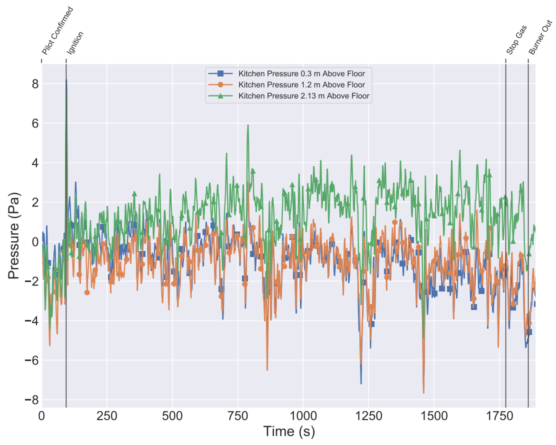

(e) Kitchen Pressures

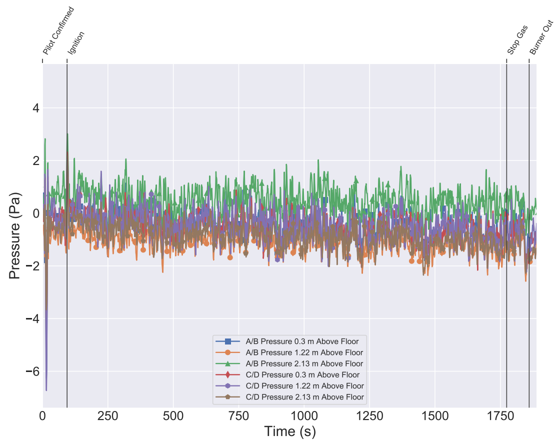

(f) Basement Pressures

Figure 5.87: Experiment 15 Pressures

The gas concentrations of oxygen and carbon dioxide are provided for the living room, bedroom 2 and bedroom 3 in Figure 5.88. Water vapor concentrations are provided for bedroom 2, bedroom 3 and the basement. Oxygen concentrations in the living room slowly dropped to between 18 and 19% while the burner was on. Carbon dioxide levels in the living room increased by less than 1% during this experiment. Bedroom 2 oxygen concentrations dropped to approximately 16% and carbon dioxide increased to approximately 3%. Bedroom 3 oxygen levels dropped to approximately 18% and carbon dioxide increased to approximately 2%. Oxygen levels decreased the most in bedroom 2 due to the open bedroom door, however there was a significant drop in oxygen concentrations behind the closed door in bedroom 3. The decrease in oxygen in bedroom 3 could be due to gas flow through the HVAC supply ducts from the living room into the bedroom. Oxygen concentrations in the living room did not drop as much as the bedrooms possibly due to larger volumes of air being pulled towards the living room burner from the basement and other rooms on the first floor. No notable changes to gas concentrations in the basement were noted in Figure 5.88(d).

Water vapor concentrations in bedroom 2 increased approximately 5% while the burner was on. Water vapor concentrations in bedroom 3 increased approximately 2% during this experiment. Water vapor concentrations in the basement did not show any significant change. The larger increase in water vapor in bedroom 2 could be attributed to a larger volume of smoke that was able to reach bedroom 2 as compared to bedroom 3.

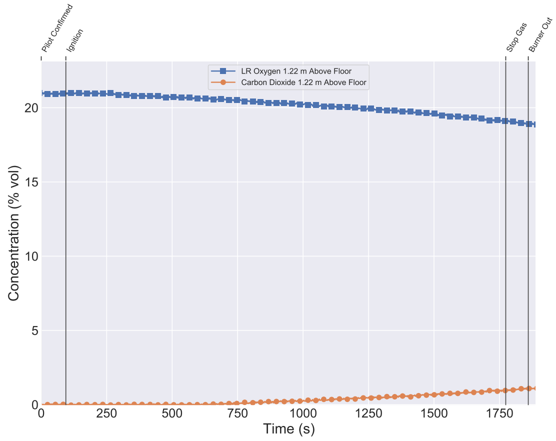

(a) Living Room Gases

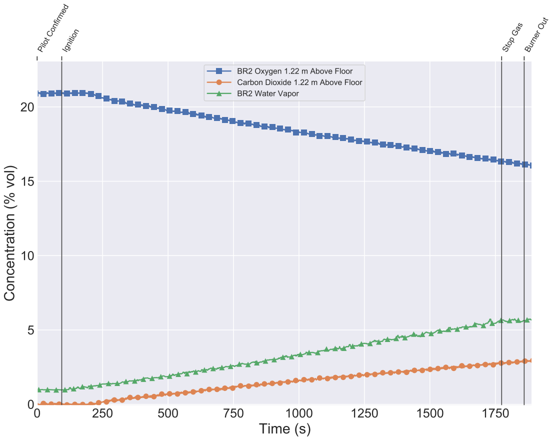

(b) Bedroom 2 Gases

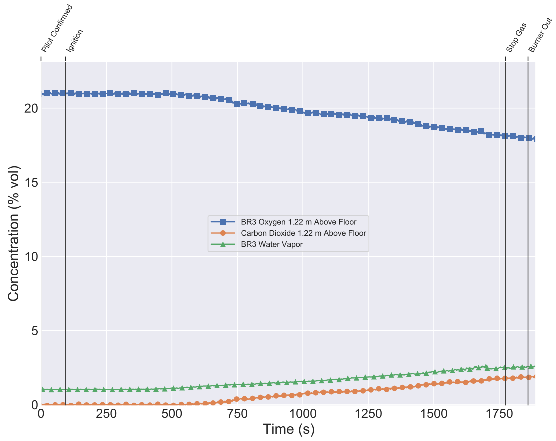

(c) Bedroom 3 Gases

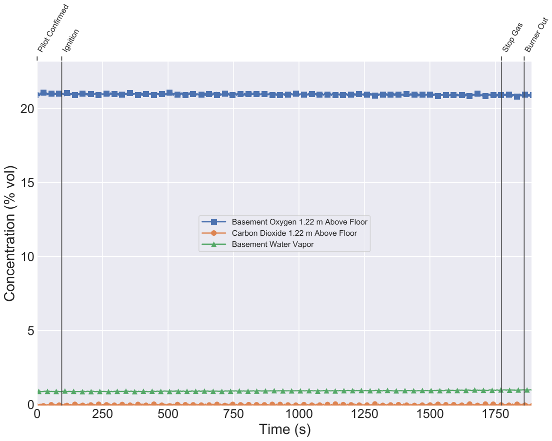

(d) Basement Gases

Figure 5.88: Experiment 15 Gases

Figure 5.89 shows the temperatures of the supply and return vents in the structure. The bedroom 1 supply vent was excluded from this chart due to a fault in the thermocoulple wire. Bedroom 3 vent temperatures reached approximately 90 °C for the supply vent and around 30 °C for the return. Bedroom 2 vent temperatures rose to approximately 70 °C at the supply vent and 40 °C at the return. The return vent in bedroom 1 reached a temperature around 45 °C. Comparing the data found

in this chart to the bedroom temperatures from Figure 5.85, it can be seen that the temperature increase in bedroom 3 appears to come from the supply vent, as that temperature is significantly greater than the temperatures in the rest of the room. The return vent temperature follows the trend of the 0.3 m thermocouple, thus showing heating from the room and not the return system ductwork. In bedroom 2 the supply vent temperature does not trend with the ceiling temperature, this could possibly be due to cooler air in the supply duct being pushed out at the end of the D side branch. The return vent temperature trends slightly lower than the temperature at the 0.3 m level but does not differ by a large temperature. The bedroom 1 return temperature trends with the temperature at 0.3 m in bedroom 1.

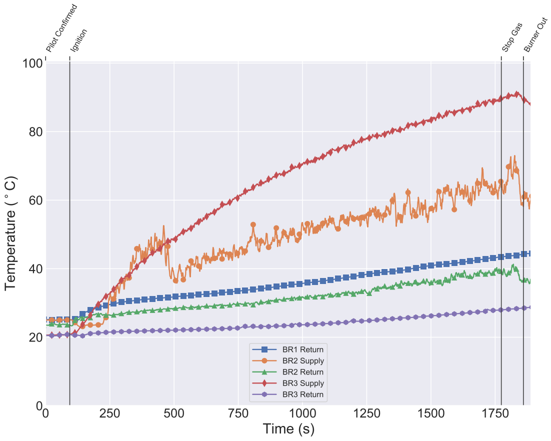

(a) Bedroom Vent Temperatures

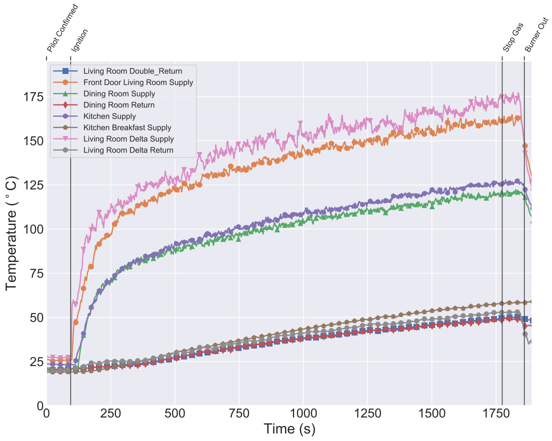

(b) First Floor Vent Temperatures

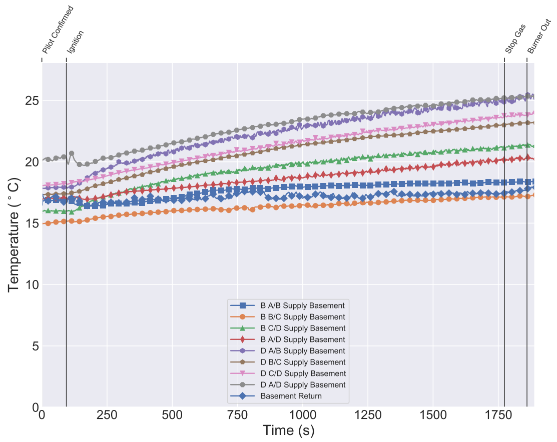

(c) Basement Vent Temperatures

Figure 5.89: Experiment 15 Vent Temperatures

First floor vent temperatures are provided in Figure 5.89(b) and appear to trend with the temperatures recorded in their respective rooms with the exception of the kitchen breakfast supply vent. Basement vent temperatures are shown in Figure 5.89(c) and show a slight increase in temperatures during this experiment.

The temperature and velocity within the supply duct are provided in Figure 5.90. From this figure it can be seen that there is a temperature increase within

the supply duct of approximately 40 °C while the gas burner was on. The increase in temperature is consistent with what was observed from the supply vent temperatures in the basement but with greater magnitude. Hot temperatures in the supply duct could drive temperature increases in the basement in conjunction with the open stairwell door. The supply vent velocity appears to show that there is a constant flow of air in the supply vent from the first floor to the basement.

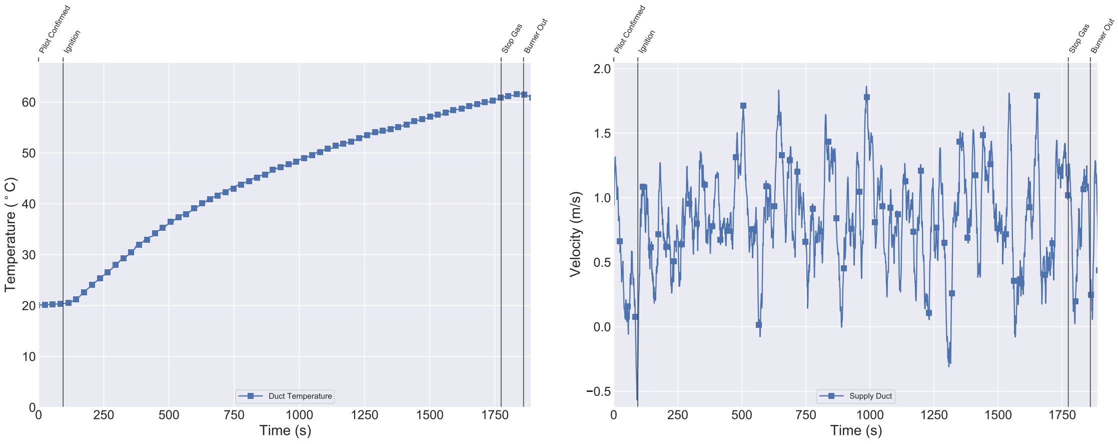

(a) Supply Duct Temperature

(b) Supply Duct Velocity

Figure 5.90: Experiment 15 Supply Duct Temperature and Velocity

5.2.2 Experiment 16

Gas Burner Experiment 16 was performed with the HVAC system off. The flow rate of propane to the gas burner was controlled so that a heat release rate of approximately 121 kW was produced. The stairwell door to the basement was closed during this experiment. Gas concentration measurements on the first floor were taken in bedroom 2, bedroom 3 and the living room. Experiment 16 was performed to compare the results to Experiment 15 but with the basement door

closed. The events of interest for Experiment 16 are presented in Table 5.16.

Table 5.16: Experiment 16 Events

Event	Elapsed Time (seconds)
Pilot Confirmed	0
Ignition	85
Stop Gas	1765
Burner Out	1857

The time versus temperature graphs from the first floor are provided in Figure 5.91. Temperatures in the living room increased approximately 150 °C at the 2.4 m height and approximately 40 °C at the 0.3 m height. Temperatures in bedroom 1 rose approximately 55 °C at the 2.4 m height and approximately 15 °C at the 0.3 m height. Temperatures in bedroom 2 increased approximately 90 °C at the 2.4 m height and approximately 35 °C at the 0.3 m height. The temperatures in bedroom 3 increased approximately 25 °C at the 2.4 m height and approximately 5 °C at the 0.3 m height. Temperatures in the dining room increased approximately 120 °C at the 2.4 m height and approximately 40 °C at the 0.3 m height. Temperatures in the kitchen increased approximately 100 °C at the 2.4 m height and approximately 40 °C at the 0.3 m height. These temperatures trend with extreme similarity to the temperatures seen in Experiment 15 (see Figure 5.85). Both bedroom 1 and bedroom 3 were behind closed doors, yet the temperatures in bedroom 1 increased far more than bedroom 3 in both Experiment 15 and 16.

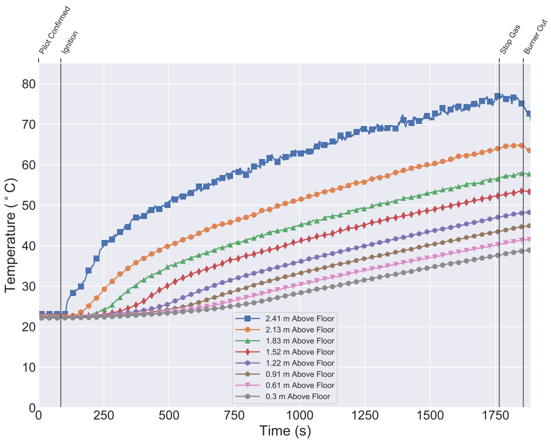

(a) Bedroom 1 Temperatures

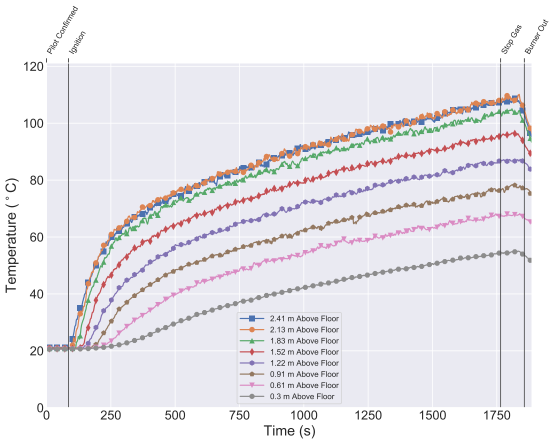

(b) Bedroom 2 Temperatures

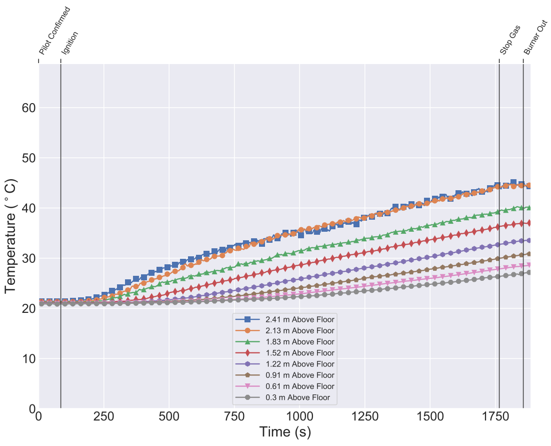

(c) Bedroom 3 Temperatures

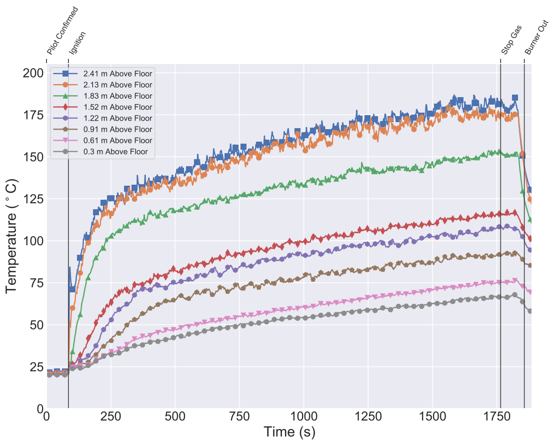

(d) Living Room Temperatures

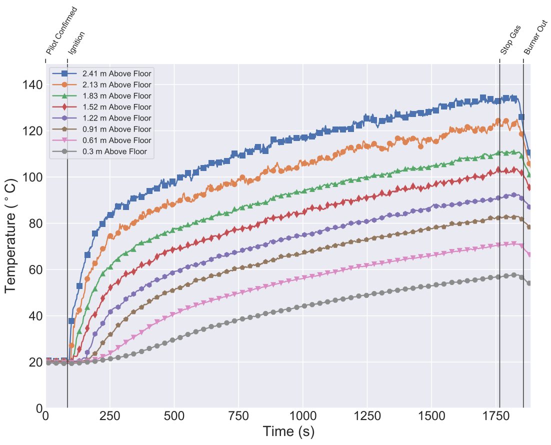

(e) Dining Room Temperatures

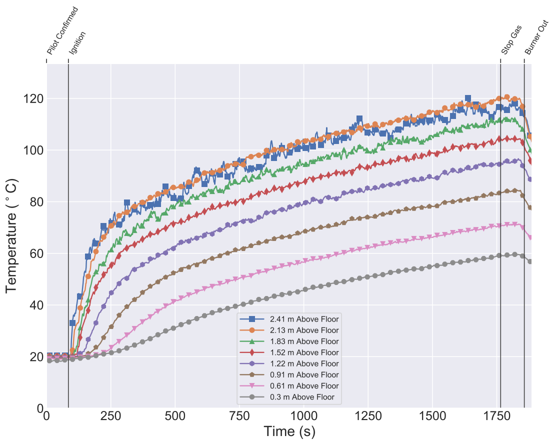

(f) Kitchen Temperatures

Figure 5.91: Experiment 16 First Floor Temperatures

The time versus temperature graphs for the four basement quadrants are provided in Figure 5.92. As was the case with Experiment 15, there was very almost no notable change in basement temperatures.

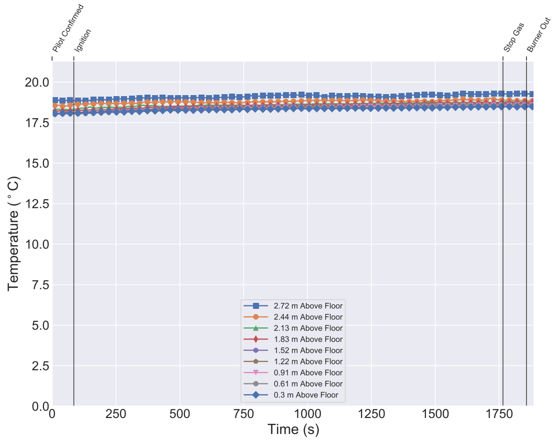

(a) Basement A Quadrant Temperatures

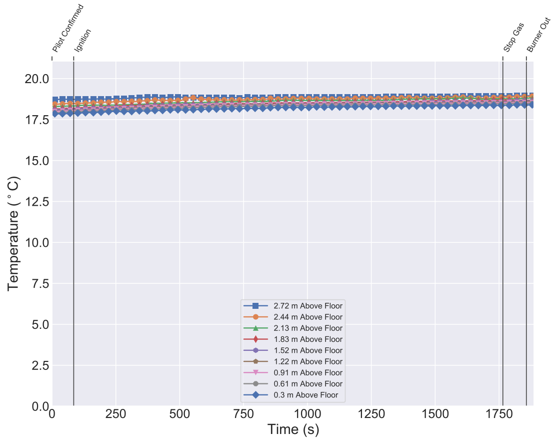

(b) Basement B Quadrant Temperatures

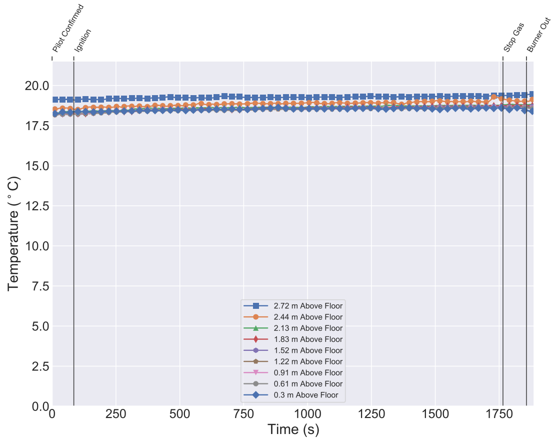

(c) Basement C Quadrant Temperatures

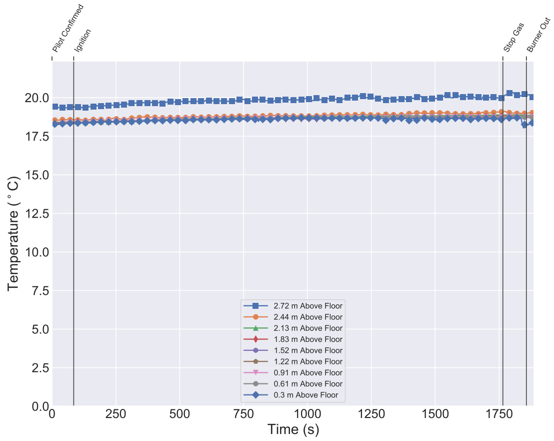

(d) Basement D Quadrant Temperatures

Figure 5.92: Experiment 16 Basement Temperatures

The pressures within each room are shown in Figure 5.93. Pressure in bedroom 1 at 2.13 m above the floor exceeded the ambient pressure by approximately 2 Pa while the burner was on. At 1.2 m above the floor pressure exceeded the ambient by

approximately 2 Pa. At 0.3 m above the floor pressure remained at approximately ambient. Following ignition there was a spike in pressure in bedroom 1 up to between 4 and 5 Pa above the ambient. Pressures in bedroom 2 at the 2.13 m height exceeded the ambient by approximately 4 Pa. At the 1.2 m height the pressure reached approximately 2 Pa. At the 0.3 m height pressures remained apporoximately equal to the ambient pressure. Pressures following ignition spiked to almost 10 Pa above the ambient. Pressures in bedroom 3 were similar at all height measurements with a peak of 3 to 4 Pa above ambient shortly after ignition. Pressures in bedroom 3 remained mostly steady between 0 and 1 pascal above the ambient pressure while the gas burner was on. Pressure spikes following ignition reached approximately 7 to 8 Pa above the ambient. Pressures in the basement did not show any significant variations from the ambient pressure including immediately after ignition. Pressure in the living room at the 2.13 m height reached between 4 and 5 Pa above the ambient while the burner was on. At the 1.2 m height pressures hovered between 1 to 2 Pa above the ambient. At the 0.3 m height pressures remained slightly negative between 0 and -1 Pa relative to the ambient pressure. Spikes following ignition reached approximately 8 Pa above the ambient in the living room. Pressures in the kitchen followed a similar trend to the pressures in the living room with a spike following ignition around 9 Pa above the ambient.

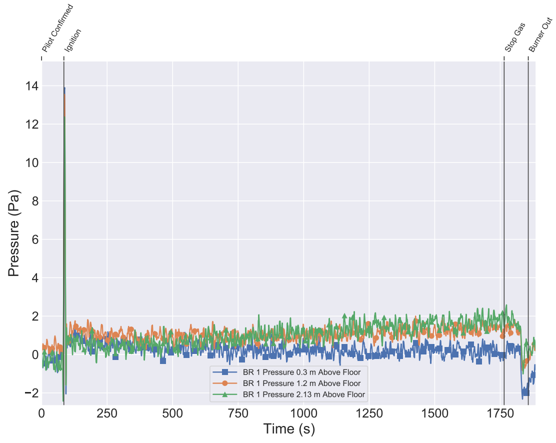

(a) Bedroom 1 Pressures

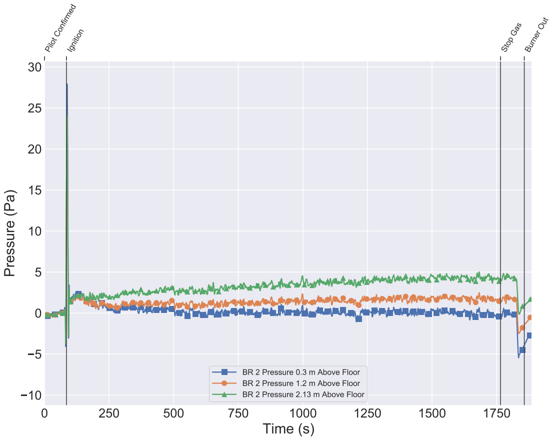

(b) Bedroom 2 Pressures

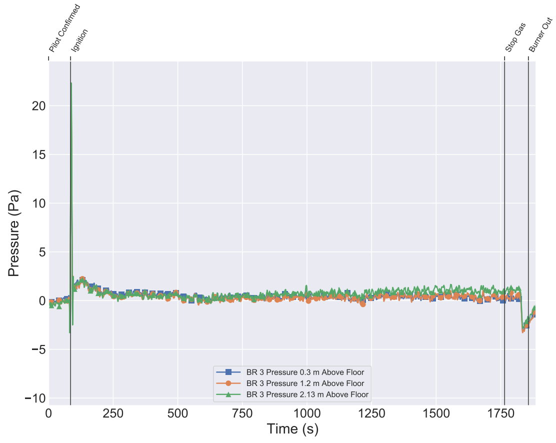

(c) Bedroom 3 Pressures

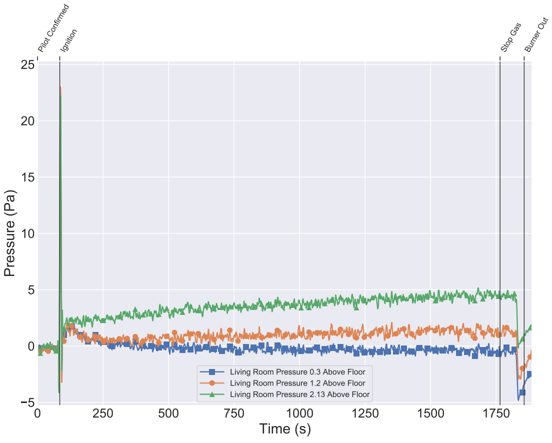

(d) Living Room Pressures

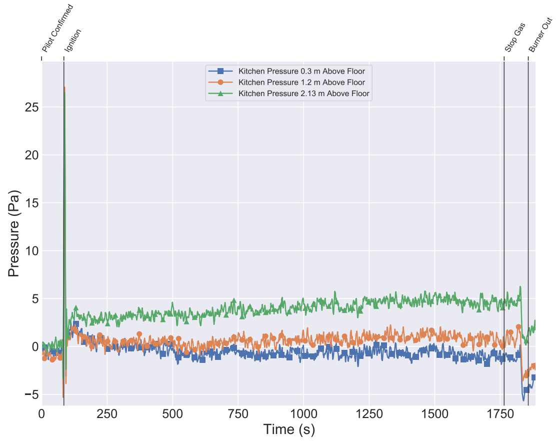

(e) Kitchen Pressures

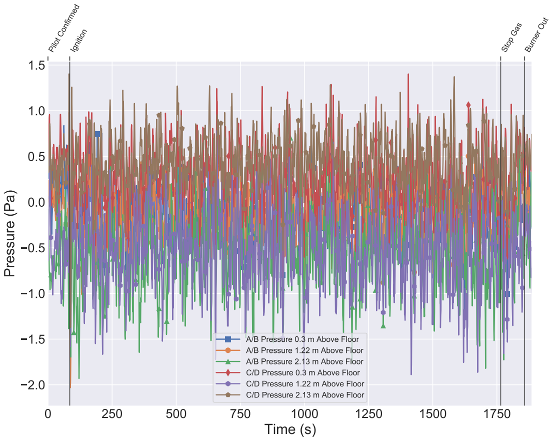

(f) Basement Pressures

Figure 5.93: Experiment 16 Pressures

The gas concentrations of oxygen and carbon dioxide are provided for the living room, bedroom 2, bedroom 3 and the basement in Figure 5.94. Water vapor concentrations are provided for bedroom 2, bedroom 3 and the basement. Oxygen concentrations in the living room slowly dropped to between 18 and 19% while the burner was on. Carbon dioxide levels in the living room increased by less than 1% during this experiment. Bedroom 2 oxygen concentrations dropped to approximately 16% and carbon dioxide increased to approximately 3 to 4%. Bedroom 3 gas levels did not appear to change during this experiment. Gas levels in the basement did not appear to be affected during this experiment. In contrast to Experiment 15, the gas concentrations in bedroom 3 did not change.

Water vapor concentrations in bedroom 2 increased approximately 5% during this experiment. Water vapor concentrations in bedroom 3 increased approximately 2% while the burner was on. Water vapor concentrations in the basement did not show any notable changes during this experiment. While bedroom 3 gas concentrations behaved differently than that of Experiment 15, the water vapor concentrations for this experiment were similar to that of the previous experiment.

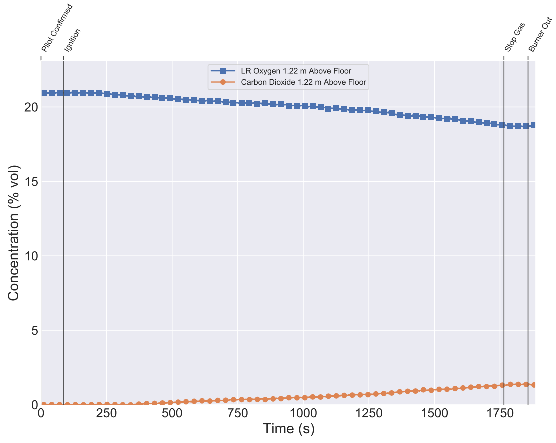

(a) Living Room Gases

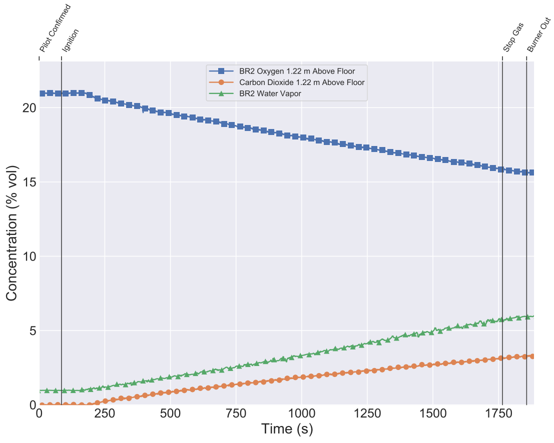

(b) Bedroom 2 Gases

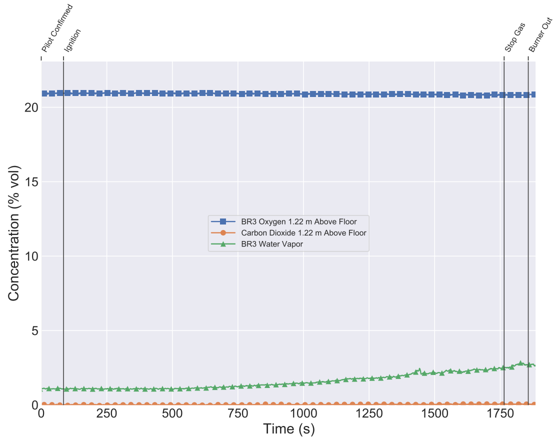

(c) Bedroom 3 Gases

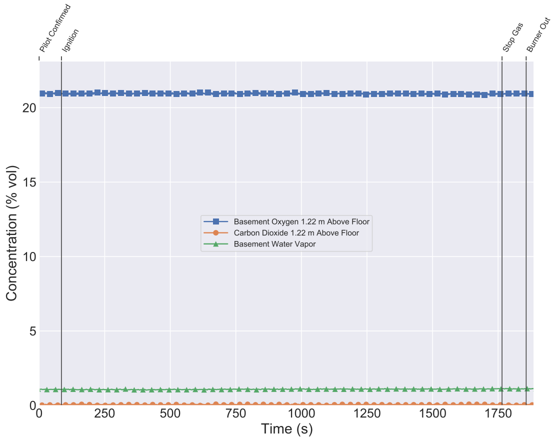

(d) Basement Gases

Figure 5.94: Experiment 16 Bedroom Gases

Figure 5.95 shows the temperatures of the supply and return vents in the structure. As was the case in Experiment 15, the bedroom 1 supply vent was excluded from this chart due to a fault in the thermocoulple wire. Bedroom 3 vent temperatures reached approximately 90 °C for the supply vent and around 30 °C for the return. Bedroom 2 vent temperatures rose to approximately 70 °C at the supply vent and 35 °C at the return. The return vent in bedroom 1 reached a

temperature around 40 °C. Comparing the data found in this chart to the bedroom temperatures from Figure 5.91, the temperature increase in bedroom 3 appears to come from the supply vent, as that temperature is significantly greater than the temperatures in the rest of the room. The return vent temperature follows the trend of the 0.3 m thermocouple, thus showing heating from the room and not the return system ductwork. In bedroom 2 the supply vent temperature does not trend with the ceiling temperature, this could possibly be due to cooler air in the supply duct being pushed out at the end of the D side branch. The return vent temperature trends slightly lower than the temperature at the 0.3 m level but does not differ by a large temperature. The bedroom 1 return temperature trends with the temperature at 0.3 m in bedroom 1.

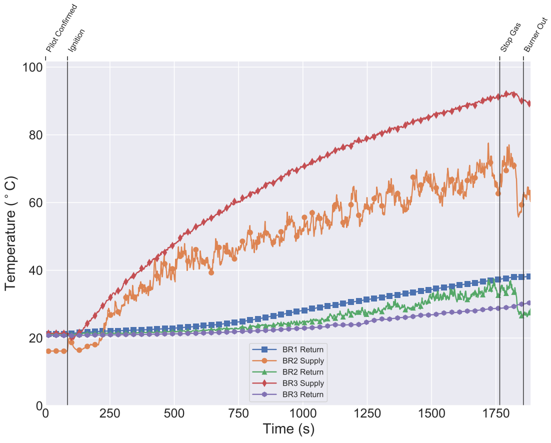

(a) Bedroom Vent Temperatures

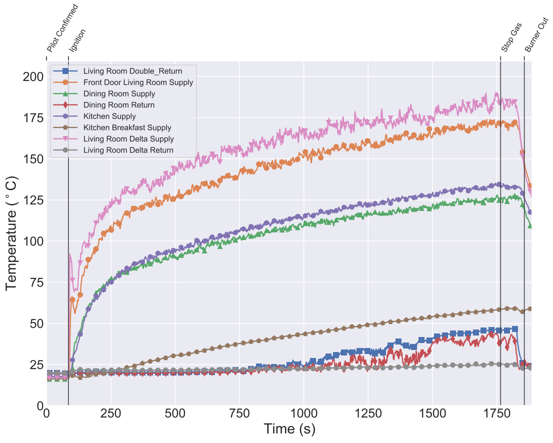

(b) First Floor Vent Temperatures

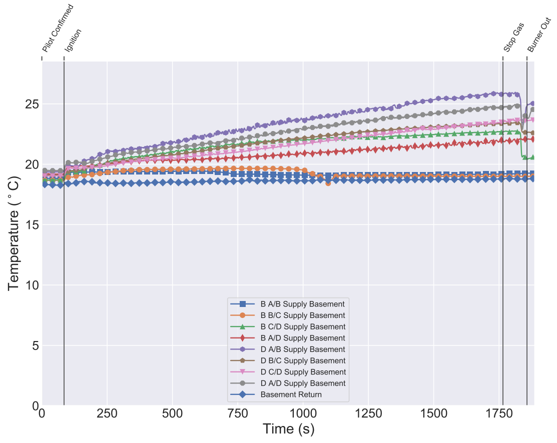

(c) Basement Vent Temperatures

Figure 5.95: Experiment 16 Vent Temperatures

First floor vent temperatures are provided in Figure 5.95(b) and appear to trend with the temperatures recorded in their respective rooms with the exception of the kitchen breakfast supply vent. Basement vent temperatures are shown in Figure 5.95(c) and show a slight increase in temperatures during this experiment for most of the supply vents.

The temperature and velocity within the supply duct are provided in Fig-

ure 5.96. From this figure it can be seen that there is a temperature increase within the supply duct of approximately 40 °C while the gas burner was on. The increase in temperature is consistent with what was observed from the supply vent temperatures in the basement but with greater magnitude. Hot temperatures in the supply duct could drive temperature increases in the basement and would be the path of least resistance with the closed stairwell door. The supply vent velocity appears to show that there is a constant flow of air in the supply vent from the first floor to the basement.

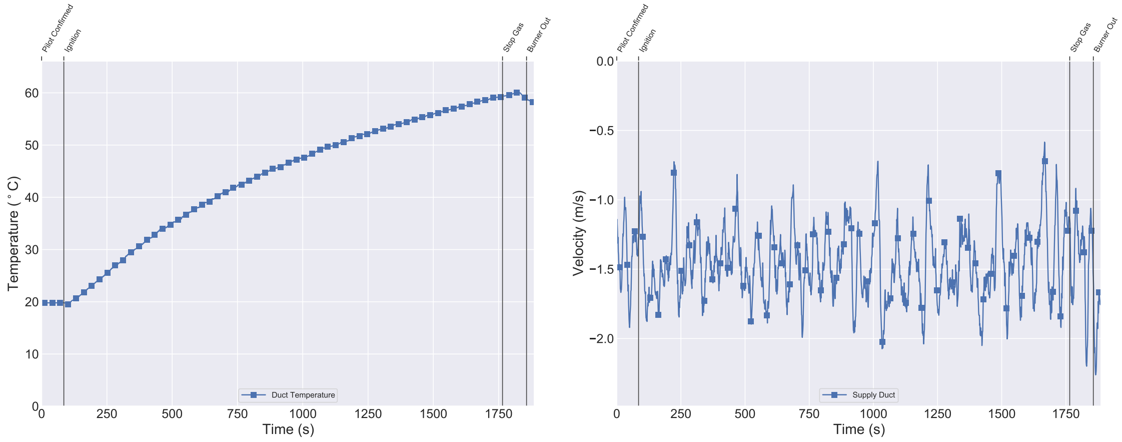

(a) Supply Duct Temperature

(b) Supply Duct Velocity

Figure 5.96: Experiment 16 Supply Duct Temperature and Velocity

5.2.3 Experiment 17

Gas Burner Experiment 17 was performed with the HVAC system set to A/C. The flow rate of propane to the gas burner was controlled so that a heat release rate of approximately 121 kW was produced. The doors to bedroom 1, bedroom 3 and the stairwell door to the basement were closed during this experiment. All exterior

vents were closed. Gas concentration measurements on the first floor were taken in bedroom 2, bedroom 3 and the living room. Experiment 17 was performed to compare the results to Experiment 16 to see the effect of the HVAC system being set to A/C with a fire in the living room. The events of interest for Experiment 17 are presented in Table 5.17.

Table 5.17: Experiment 17 Events

Event	Elapsed Time (seconds)
Pilot Confirmed	0
Ignition	129
Stop Gas	910
Burner Out	983

The time versus temperature graphs from the first floor are provided in Figure 5.97. The temperatures in bedroom 1 and bedroom 2 did not show any difference in trends and values when compared to Experiment 16. With the HVAC set to A/C, the increase in temperature in bedroom 3 was held to only a few degree increase during the experiment. Temperatures in the dining room and the kitchen did not show any difference from the data gathered in Experiment 16.

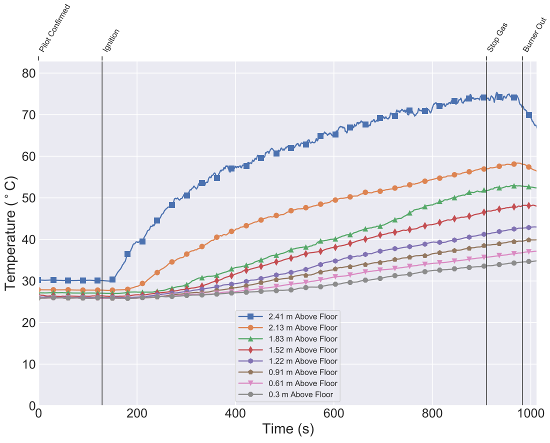

(a) Bedroom 1 Temperatures

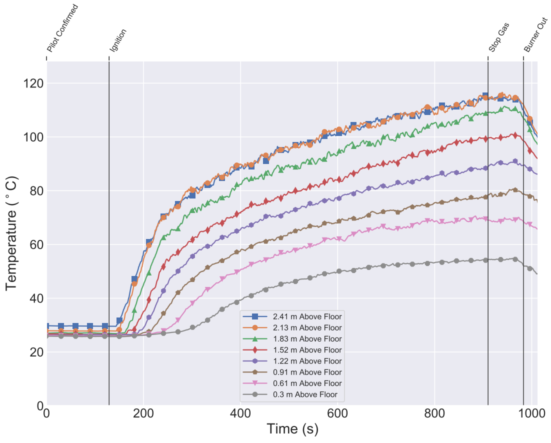

(b) Bedroom 2 Temperatures

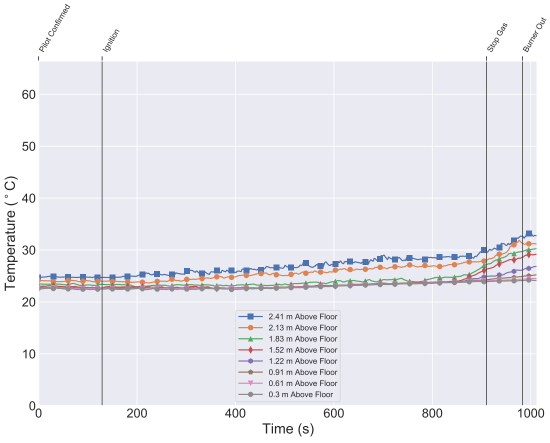

(c) Bedroom 3 Temperatures

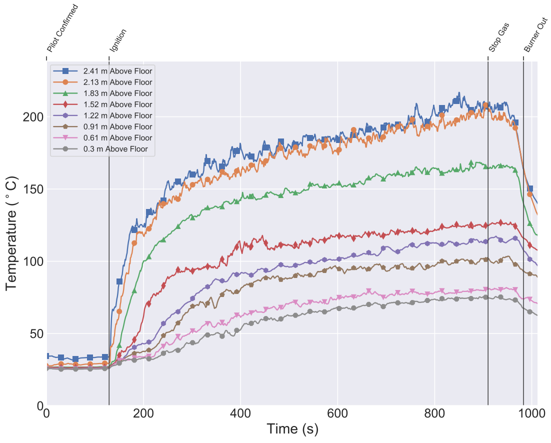

(d) Living Room Temperatures

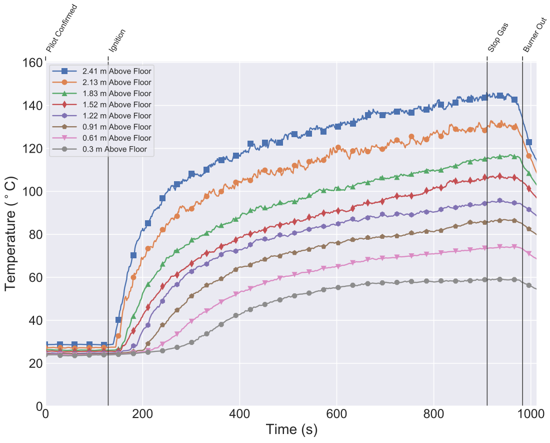

(e) Dining Room Temperatures

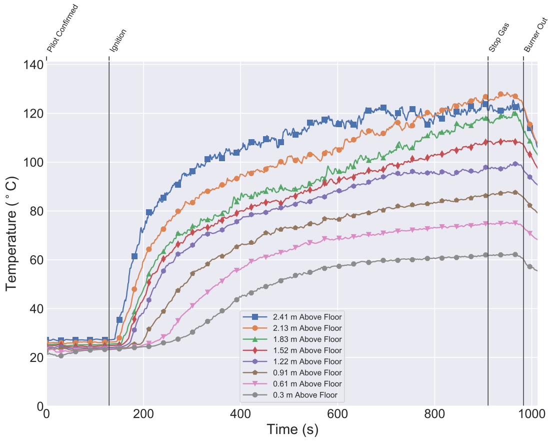

(f) Kitchen Temperatures

Figure 5.97: Experiment 17 First Floor Temperatures

The time versus temperature graphs for the four basement quadrants are provided in Figure 5.98. No notable change in basement temperatures was observed in this experiment.

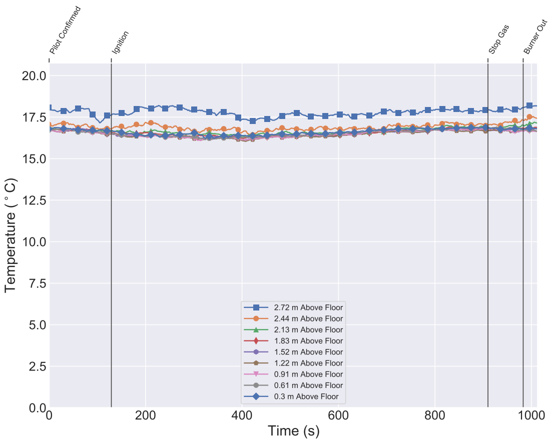

(a) Basement A Quadrant Temperatures

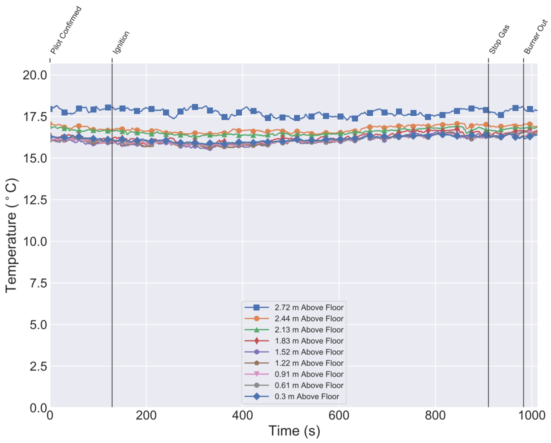

(b) Basement B Quadrant Temperatures

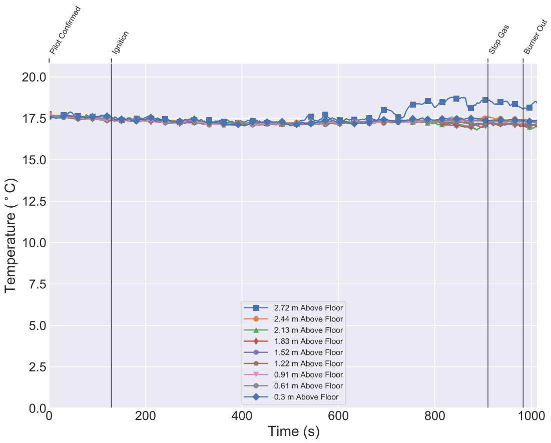

(c) Basement C Quadrant Temperatures

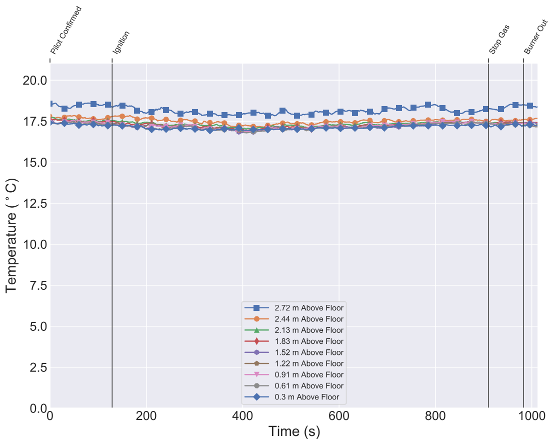

(d) Basement D Quadrant Temperatures

Figure 5.98: Experiment 17 Basement Temperatures

The pressures within each room are shown in Figure 5.99. Pressures in bedrooms 1 and 2 followed a similar trend to that seen in Experiment 16. In bedroom 3, the pressures started in the positive range but slowly progressed into a negative

pressure region. This differed from the data collected in Experiment 16 where the pressure remained constant slightly above 0 Pa. Pressures in the basement fluctuated with an average slightly below the ambient pressure. Pressures in the living room followed the same trend as that recorded in Experiment 16. Pressures in the kitchen were similar at the 1.2 m and 2.13 m heights to that of the previous experiment. Pressure at the 0.3 m height was slightly lower than that of Experiment 16 and reached a negative pressure of approximately 5 Pa.

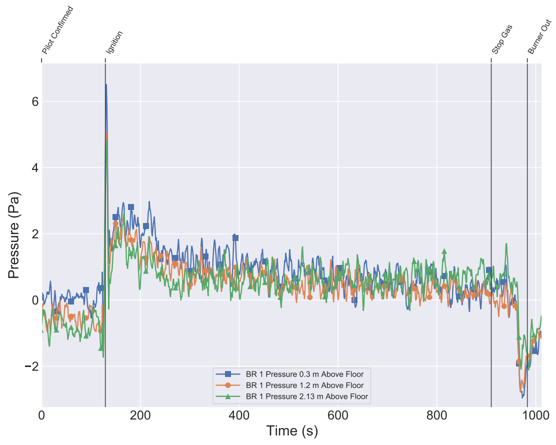

(a) Bedroom 1 Pressures

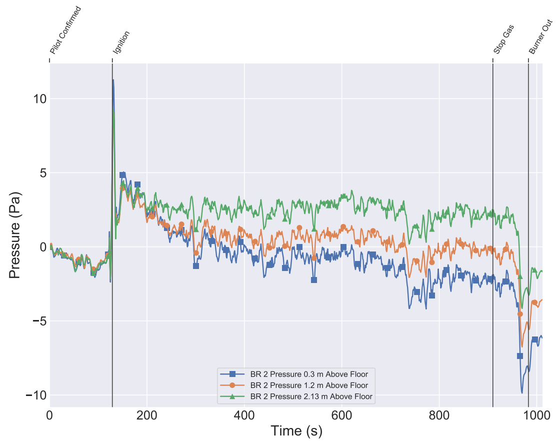

(b) Bedroom 2 Pressures

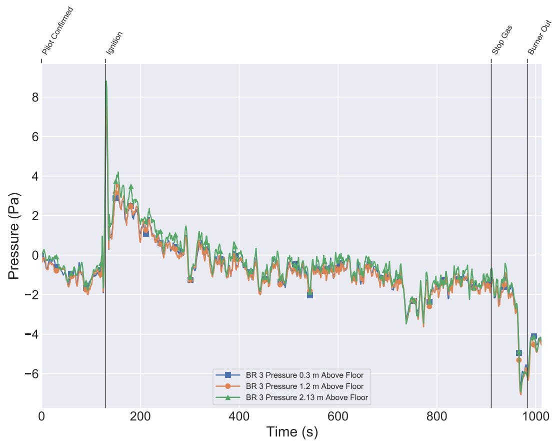

(c) Bedroom 3 Pressures

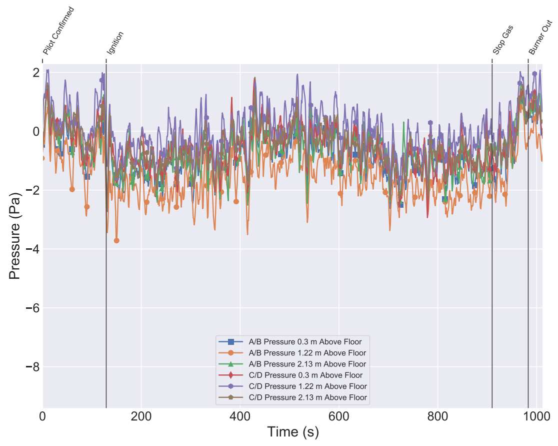

(d) Basement Pressures

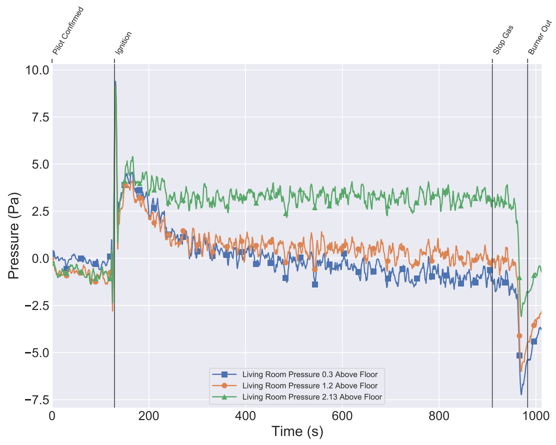

(e) Living Room Pressures

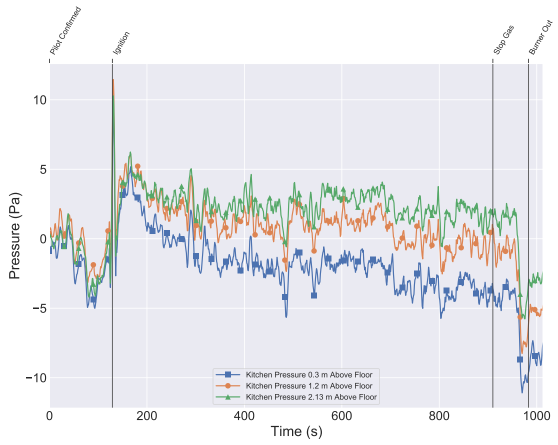

(f) Kitchen Pressures

Figure 5.99: Experiment 17 Pressures

The gas concentrations of oxygen and carbon dioxide are provided for the living room, bedroom 2, bedroom 3 and the basement in Figure 5.100. Water vapor concentrations are provided for bedroom 2, bedroom 3 and the basement. Oxygen concentrations in the living room remained higher than those observed in Experiment 16 and carbon dioxide concentrations were lower as well. In bedroom 2, the oxygen concentration dropped to approximately 17%, water vapor increased approximately 4% and carbon dioxide increased approximately 3%. Concentrations in bedroom 3 and the basement remained constant throughout the duration of this experiment.

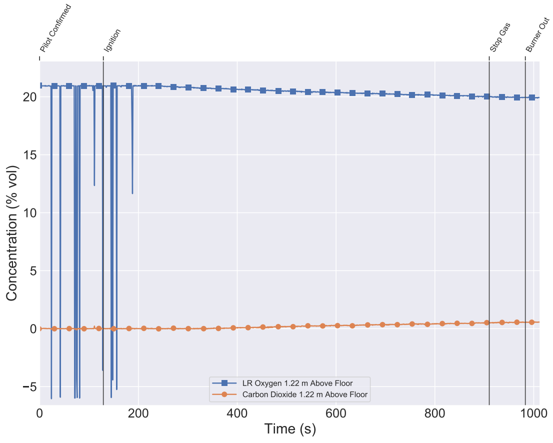

(a) Living Room Gases

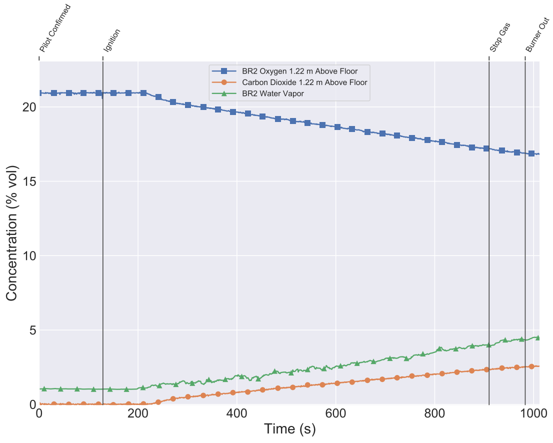

(b) Bedroom 2 Gases

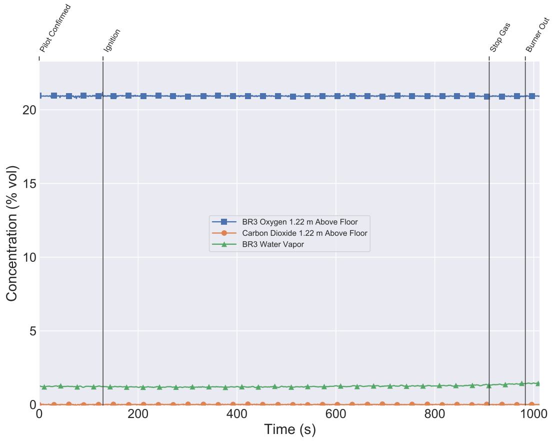

(c) Bedroom 3 Gases

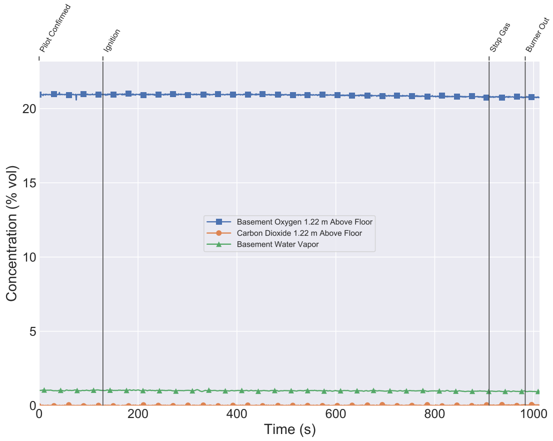

(d) Basement Gases

Figure 5.100: Experiment 17 Bedroom Gases

Figure 5.101 shows the temperatures of the supply and return vents in the structure. From Figure 5.101(a), it can be seen that the HVAC system set to A/C was able to keep the bedroom supply vents cool for the majority of the experiment. Approximately 600 seconds following ignition, temperature increases can be observed in the bedroom supply vents. The bedroom 2 supply vent had a sharp increase in temperature when the pressure in bedroom 2 was able to overpower that

of the pressure within the HVAC supply ducts.

Figure 5.101(b) shows that the HVAC supply system was able to keep the rest of the supply vents on the first floor cool for several hundred seconds following ignition. Prior to the end of the experiment, pressures in the living room and kitchen were able to overpower the pressure generated within the supply ducts. Return vents trended with the temperatures near the floor for their respective locations. Vent temperatures in the basement followed similar trends to those observed in previous experiments where the HVAC was set to A/C.

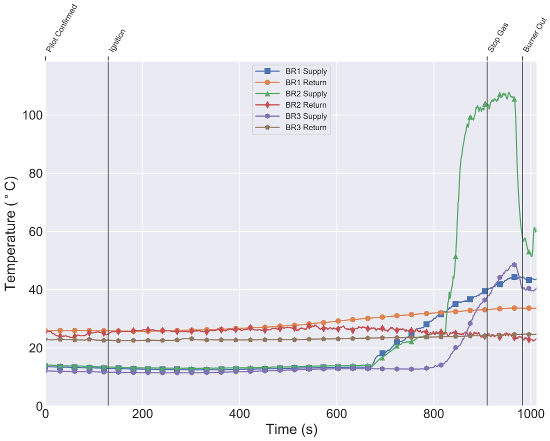

(a) Bedroom Vent Temperatures

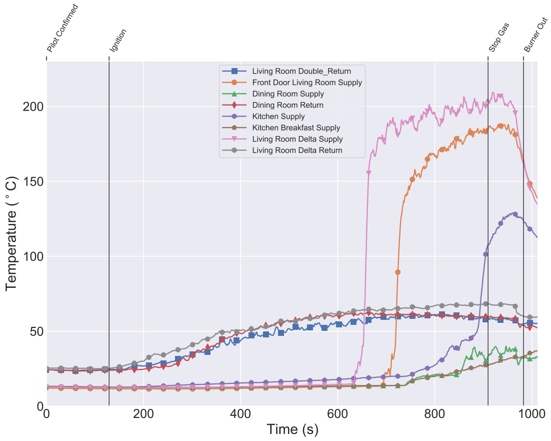

(b) First Floor Vent Temperatures

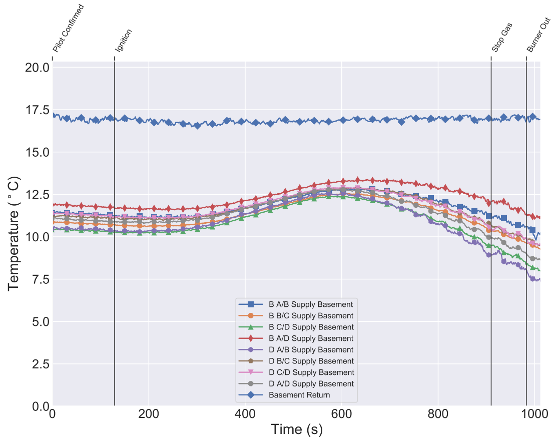

(c) Basement Vent Temperatures

Figure 5.101: Experiment 17 Vent Temperatures

The temperature and velocity within the supply duct are provided in Figure 5.102. The temperature in the supply duct is held constant by the conditioned air being pushed up to the first floor.

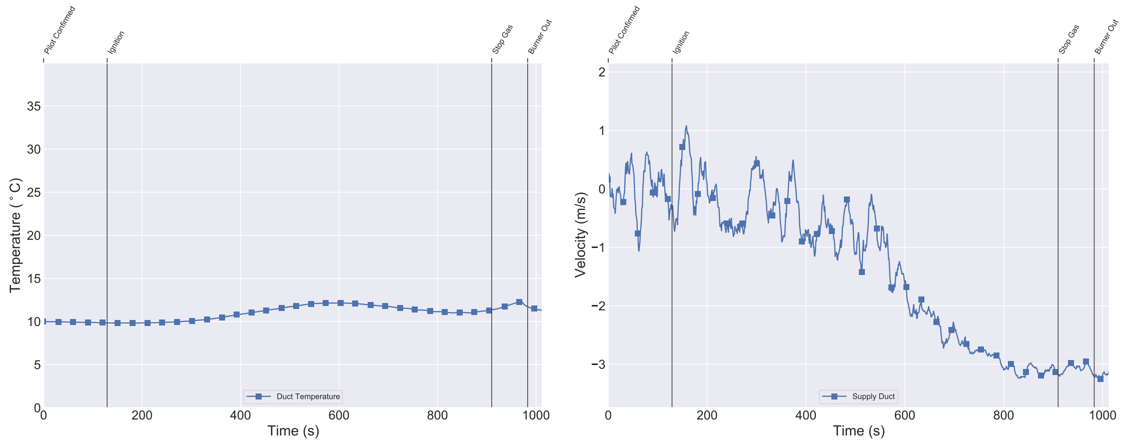

(a) Supply Duct Temperature

(b) Supply Duct Velocity

Figure 5.102: Experiment 17 Supply Duct Temperature and Velocity

5.2.4 Experiment 18

Gas Burner Experiment 18 was performed with the HVAC system off. The flow rate of propane to the gas burner was controlled so that an initial heat release rate of approximately 121 kW was produced and then ramped up to 302 kW. The doors to bedroom 1, bedroom 3 and the stairwell door to the basement were closed during this experiment. All exterior vents were closed. Gas concentration measurements on the first floor were taken in bedroom 2, bedroom 3 and the living room. Experiment 18 was performed to compare the results to Experiment 16 to see the effects of a higher heat release rate on heat and gas spread with the HVAC system off. The events of interest for Experiment 18 are presented in Table 5.18.

Table 5.18: Experiment 18 Events

Event	Elapsed Time (seconds)
Pilot Confirmed	0
Ignition	108
Increased HRR	250
Stop Gas	904

The time versus temperature graphs from the first floor are provided in Figure 5.103. As was the case with previous experiments where a higher HRR was utilized, the temperature trends identified in Experiment 16 are similar, but the magnitude of temperature increase is greater at all locations on the first floor.

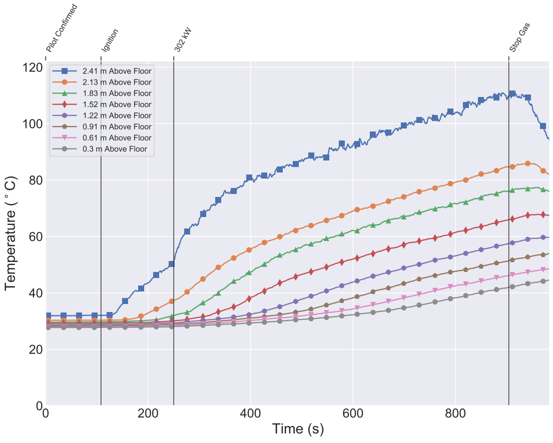

(a) Bedroom 1 Temperatures

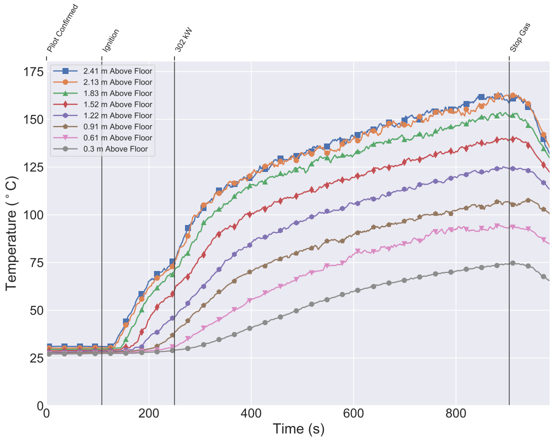

(b) Bedroom 2 Temperatures

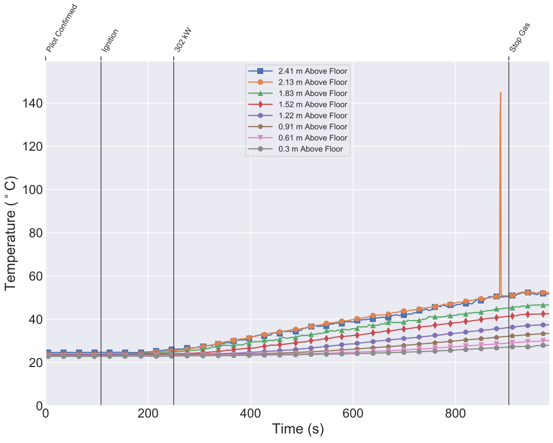

(c) Bedroom 3 Temperatures

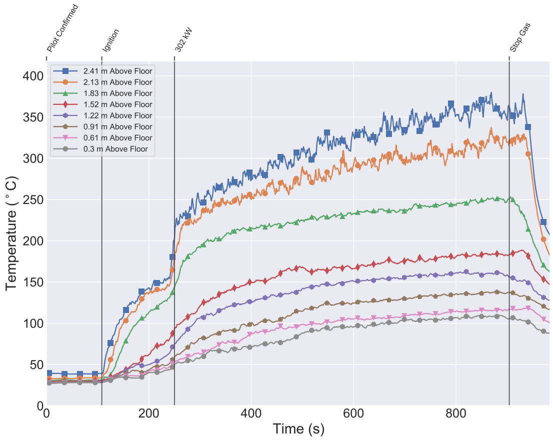

(d) Living Room Temperatures

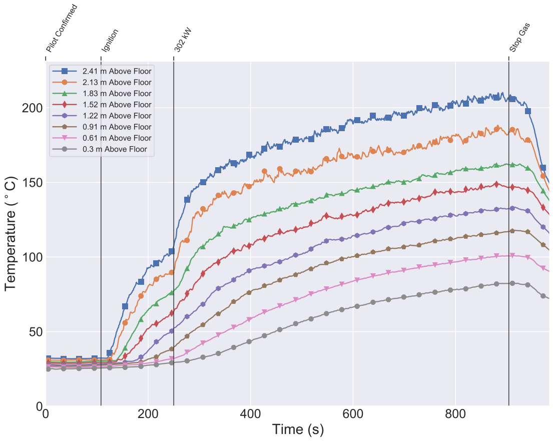

(e) Dining Room Temperatures

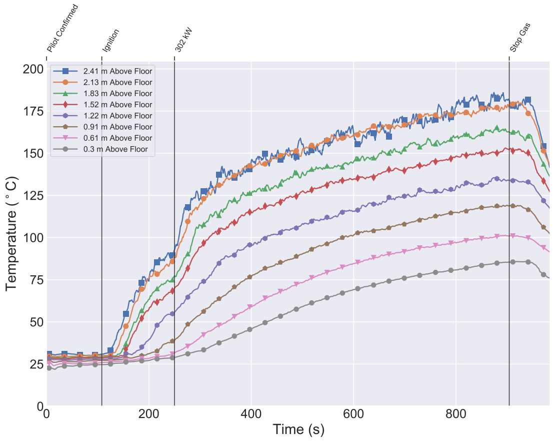

(f) Kitchen Temperatures

Figure 5.103: Experiment 18 First Floor Temperatures

The time versus temperature graphs for the four basement quadrants are provided in Figure 5.104. Similar to Experiment 16, only a slight temperature increase can be seen during the experiment.

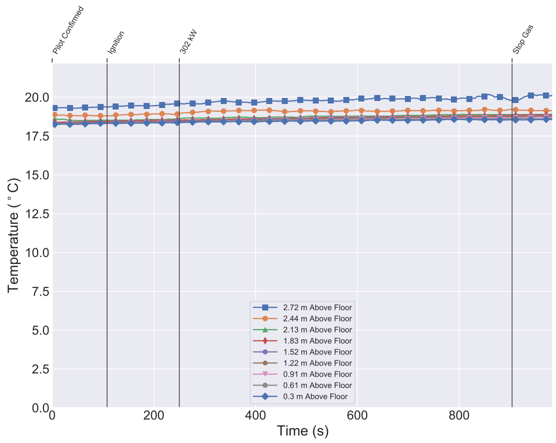

(a) Basement A Quadrant Temperatures

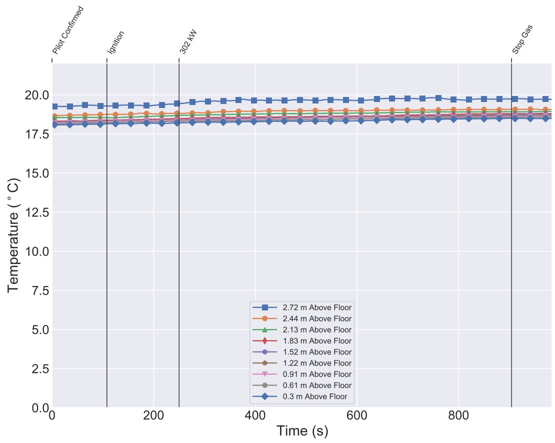

(b) Basement B Quadrant Temperatures

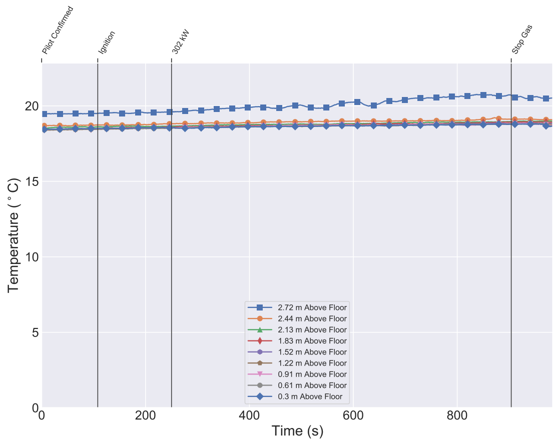

(c) Basement C Quadrant Temperatures

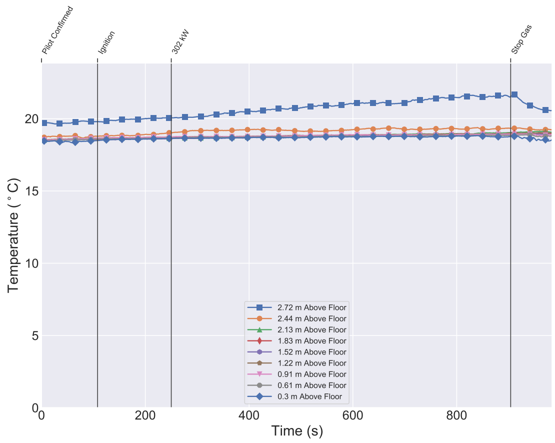

(d) Basement D Quadrant Temperatures

Figure 5.104: Experiment 18 Basement Temperatures

The pressures within each room are shown in Figure 5.105. Pressures in bedroom 1 and 2 are slightly greater than those of Experiment 16 following a second pressure spike when the HRR was increased. Pressures in bedroom 3 returned to

near ambient levels following the second spike in pressure. Pressures in the basement fluctuated around ambient with a greater amplitude following the second pressure spike. Pressures in the living room and kitchen were similar to Experiment 16 with a higher pressure recorded at the 2.13 m height.

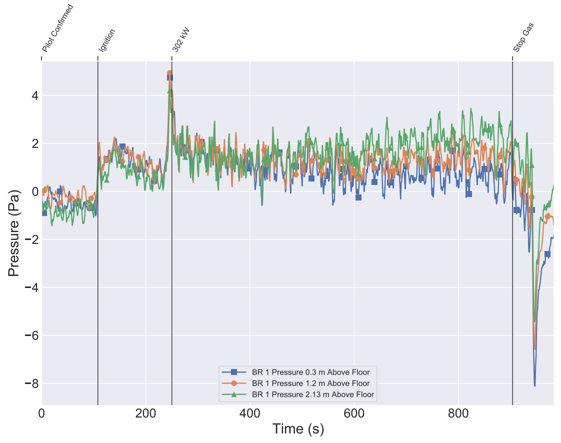

(a) Bedroom 1 Pressures

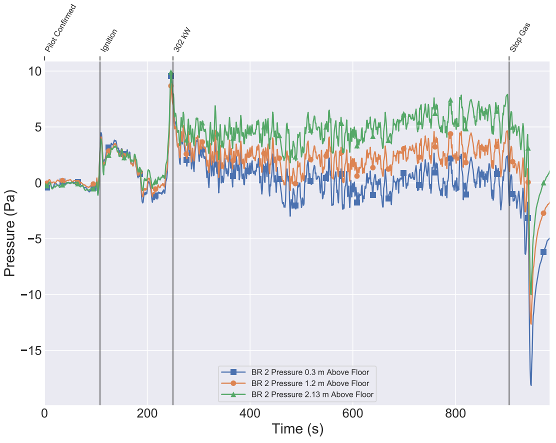

(b) Bedroom 2 Pressures

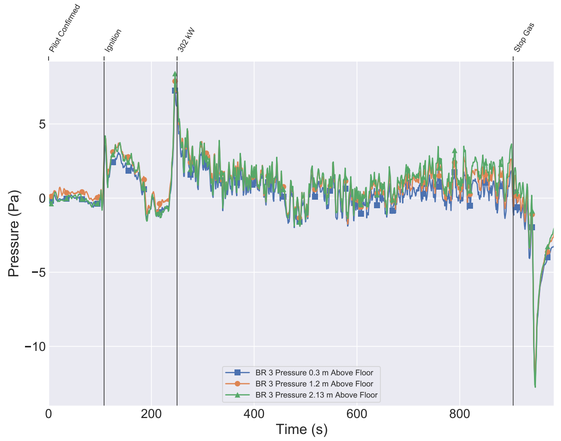

(c) Bedroom 3 Pressures

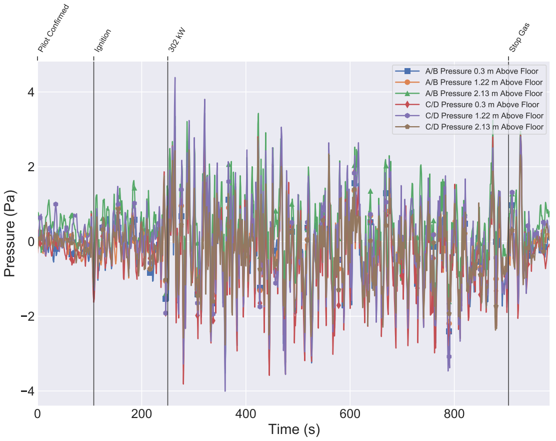

(d) Basement Pressures

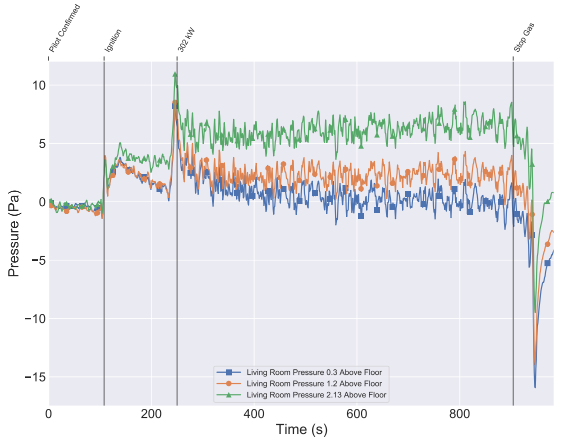

(e) Living Room Pressures

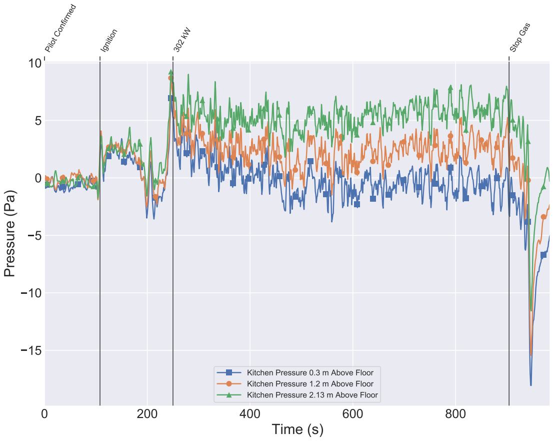

(f) Kitchen Pressures

Figure 5.105: Experiment 18 Pressures

The gas concentrations of oxygen and carbon dioxide are provided for the living room, bedroom 2, bedroom 3 and the basement in Figure 5.106. Water vapor concentrations are provided for bedroom 2, bedroom 3 and the basement. Gas concentrations in the living room changed less than they did in Experiment 16. In bedroom 2, oxygen concentrations dropped to approximately 13%, carbon dioxide levels increased approximately 5% and water vapor concentrations increased approximately 9% during the experiment. Gas concentrations in bedroom 3 and the basement were similar to those recorded in Experiment 16.

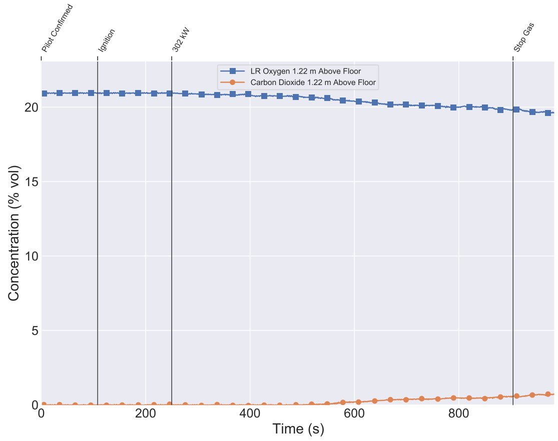

(a) Living Room Gases

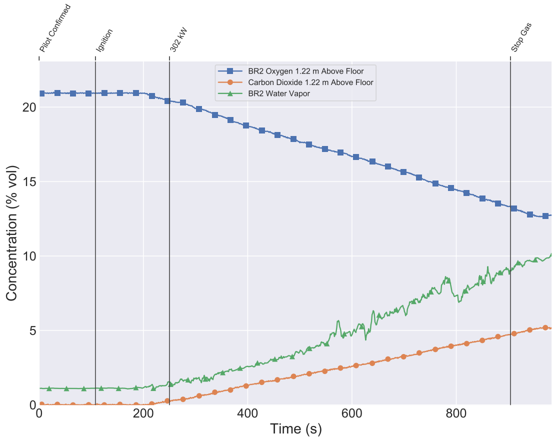

(b) Bedroom 2 Gases

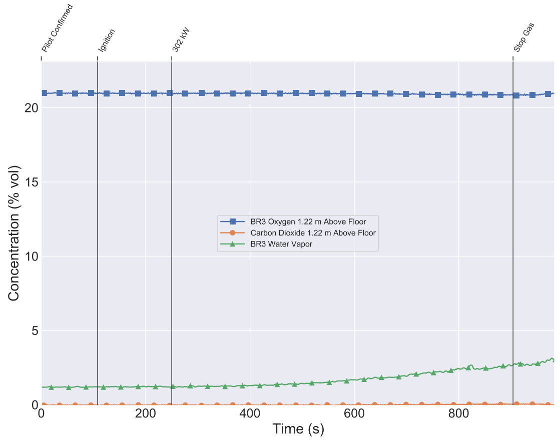

(c) Bedroom 3 Gases

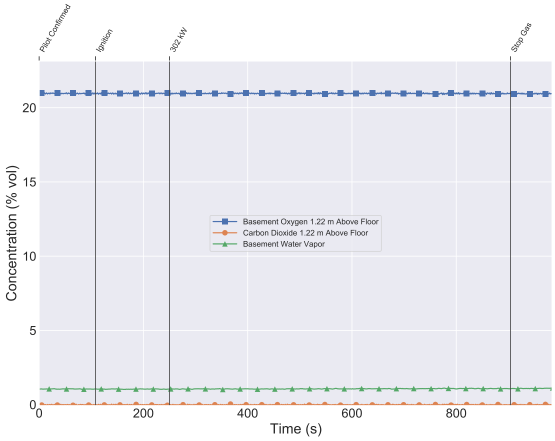

(d) Basement Gases

Figure 5.106: Experiment 18 Bedroom Gases

Figure 5.107 shows the temperatures of the supply and return vents in the structure. Similar to the temperatures reported on the first floor, the temperature trends recorded at the vents throughout the structure were similar to those of Experiment 16 but with greater temperatures being reached.

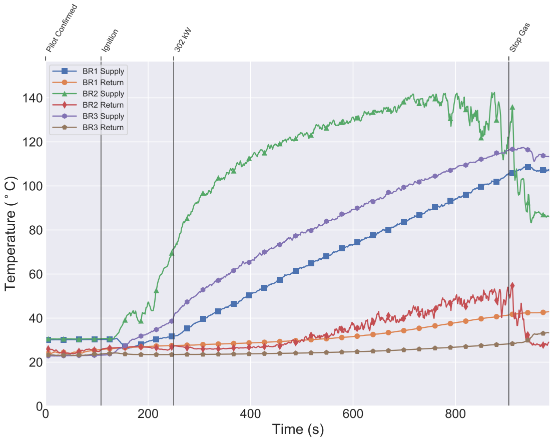

(a) Bedroom Vent Temperatures

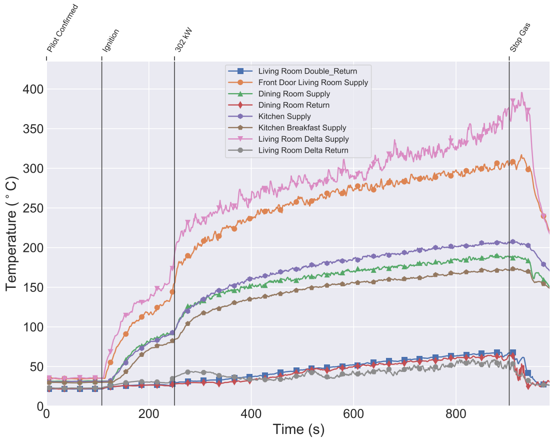

(b) First Floor Vent Temperatures

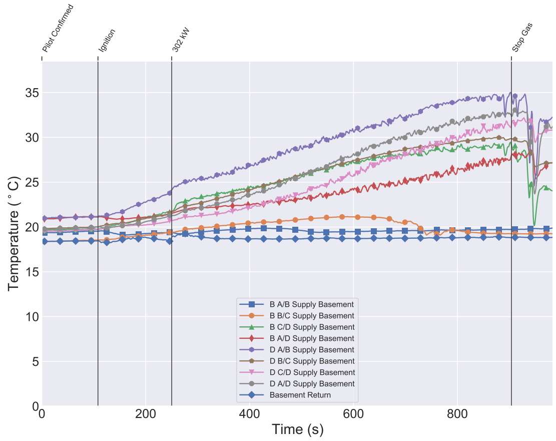

(c) Basement Vent Temperatures

Figure 5.107: Experiment 18 Vent Temperatures

The temperature and velocity within the supply duct are provided in Figure 5.108. A greater temperature increase was recorded within the supply duct than was seen in Experiment 16 because of the greater HRR of this experiment.

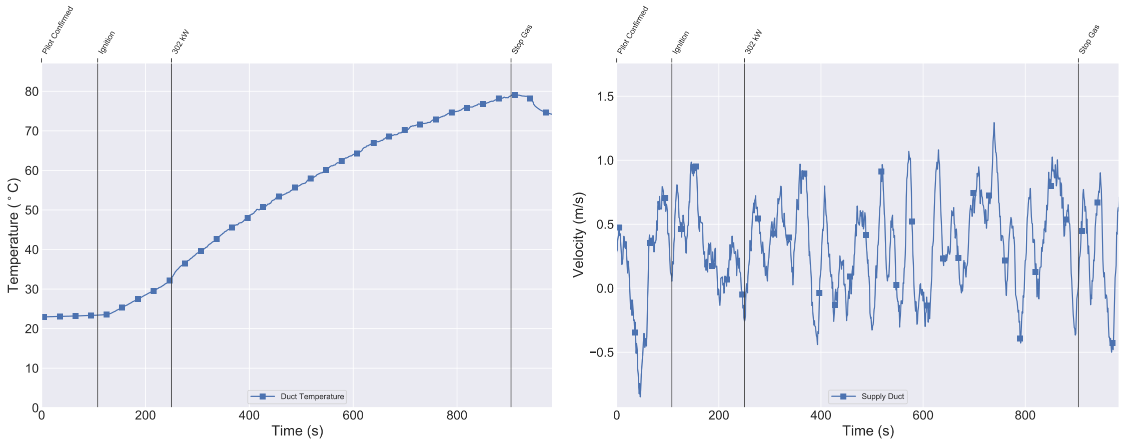

(a) Supply Duct Temperature

(b) Supply Duct Velocity

Figure 5.108: Experiment 18 Supply Duct Temperature and Velocity

5.2.5 Experiment 19

Gas Burner Experiment 19 was performed with the HVAC system set to A/C. The flow rate of propane to the gas burner was controlled so that an initial heat release rate of approximately 121 kW was produced and then ramped up to 302 kW. The doors to bedroom 1, bedroom 3 and the stairwell door to the basement were closed during this experiment. All exterior vents were closed. Gas concentration measurements on the first floor were taken in bedroom 1, bedroom 2 and bedroom 3. Experiment 19 was performed to compare the results to Experiment 18 to see the effect of the HVAC system with a higher HRR. The events of interest for Experiment 19 are presented in Table 5.19.

Table 5.19: Experiment 19 Events

Event	Elapsed Time (seconds)
Pilot Confirmed	0
Ignition	111
Increased HRR	279
Stop Gas	730
Burner Out	787

The time versus temperature graphs from the first floor are provided in Figure 5.109. Temperatures in bedroom 1 were able to be kept approximately 20 °C cooler with the HVAC system set to A/C than with the system off. Temperatures in bedrooms 2 and 3 also increased less because of the HVAC system being on. Temperatures in the living room, kitchen and dining room all behaved similarly to the data recorded in Experiment 18.

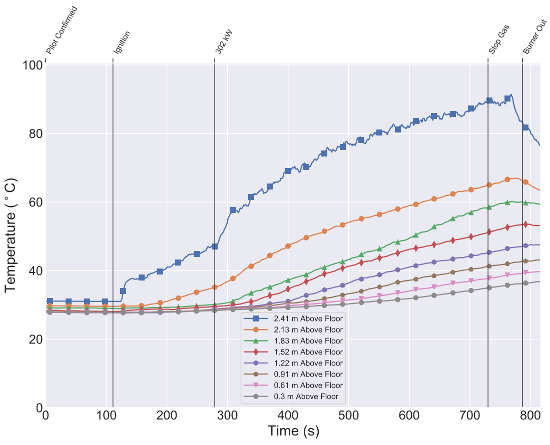

(a) Bedroom 1 Temperatures

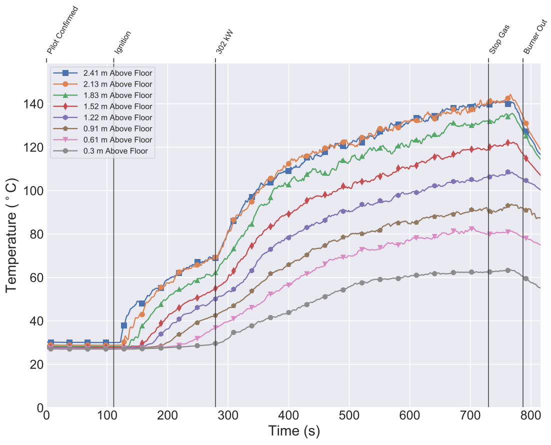

(b) Bedroom 2 Temperatures

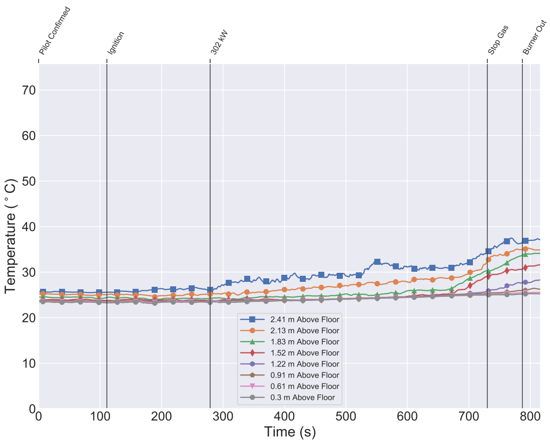

(c) Bedroom 3 Temperatures

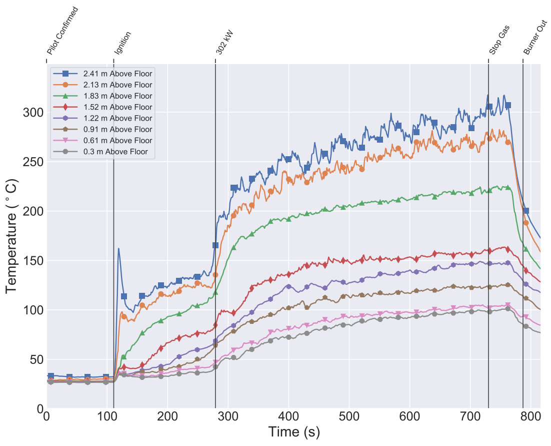

(d) Living Room Temperatures

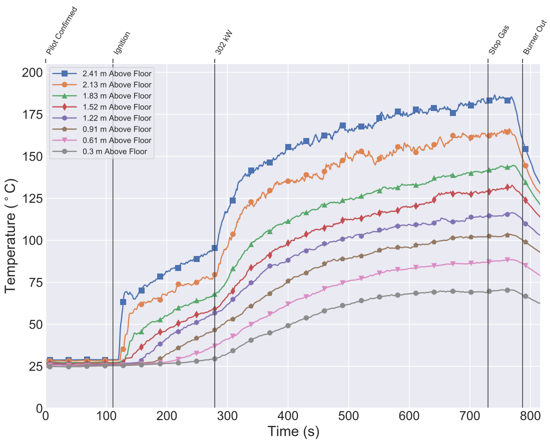

(e) Dining Room Temperatures

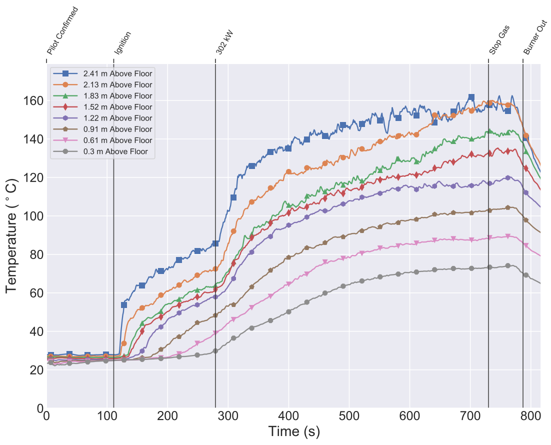

(f) Kitchen Temperatures

Figure 5.109: Experiment 19 First Floor Temperatures

The time versus temperature graphs for the four basement quadrants are provided in Figure 5.110. No notable change in basement temperatures occurred.

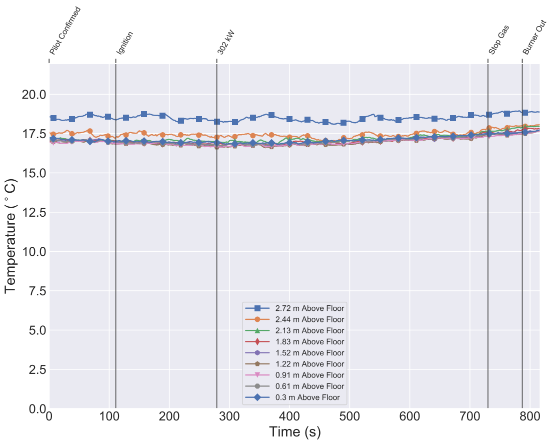

(a) Basement A Quadrant Temperatures

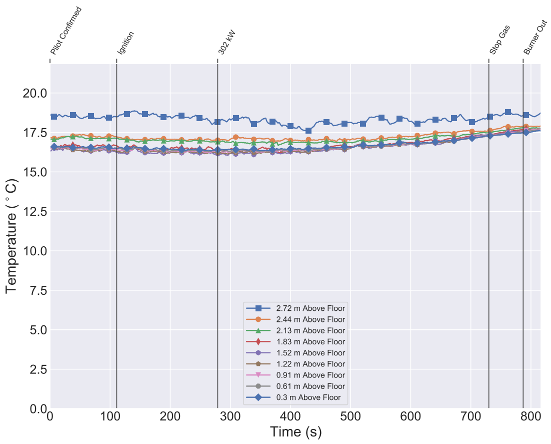

(b) Basement B Quadrant Temperatures

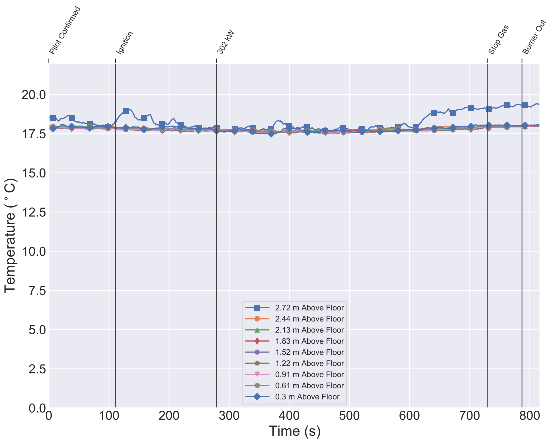

(c) Basement C Quadrant Temperatures

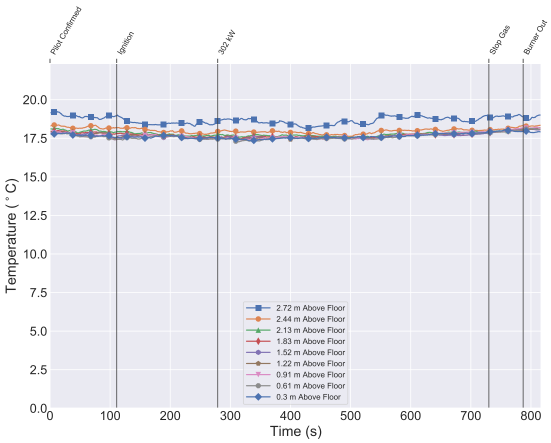

(d) Basement D Quadrant Temperatures

Figure 5.110: Experiment 19 Basement Temperatures

The pressures within each room are shown in Figure 5.111. With the exception of a greater pressure spike following ignition, pressures in the bedrooms followed similar trends to the data recorded in Experiment 18. Pressures in the basement were similar to those reported in the previous experiment. Similar to the bedrooms,

pressures in the living room and kitchen experienced a large spike following ignition and then returned to similar values as those of Experiment 18.

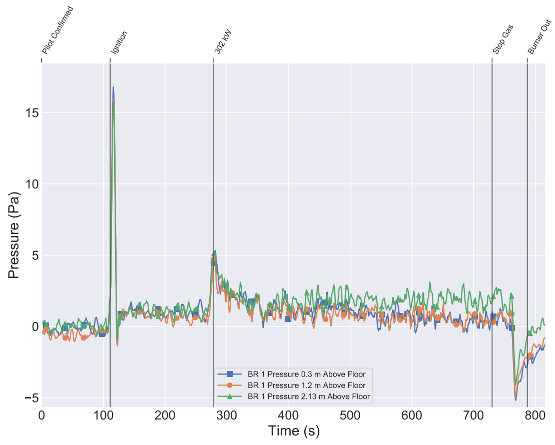

(a) Bedroom 1 Pressures

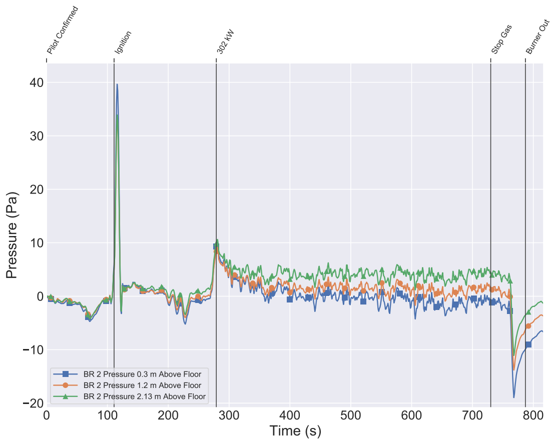

(b) Bedroom 2 Pressures

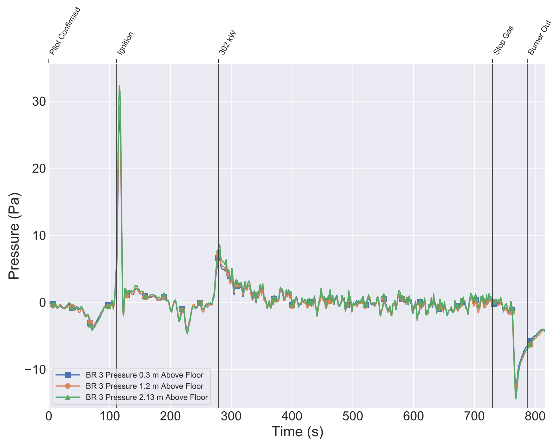

(c) Bedroom 3 Pressures

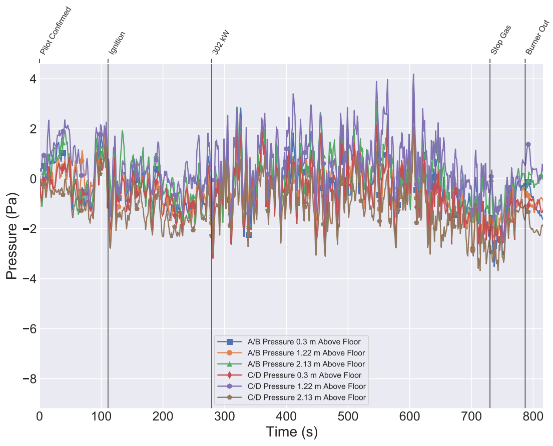

(d) Basement Pressures

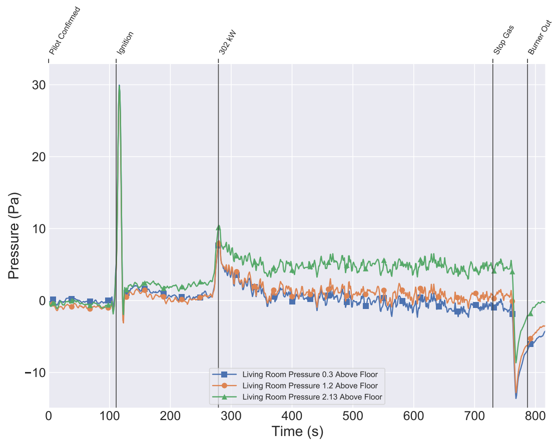

(e) Living Room Pressures

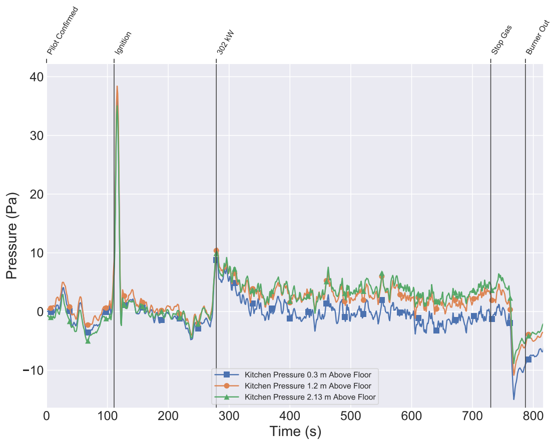

(f) Kitchen Pressures

Figure 5.111: Experiment 19 Pressures

The gas concentrations of oxygen and carbon dioxide are provided for the living room, bedroom 2, bedroom 3 and the basement in Figure 5.112. Water vapor concentrations are provided for bedroom 2, bedroom 3 and the basement. Gas concentrations in bedroom 1 showed similar changes as those recorded in Experiment 18. Gas and vapor concentrations in bedroom 2 followed similar trends as Experiment 18 and were trending towards the same final values had the experiment duration been the same. Gas and vapor concentrations in bedroom 3 and the basement were unchanged during this experiment.

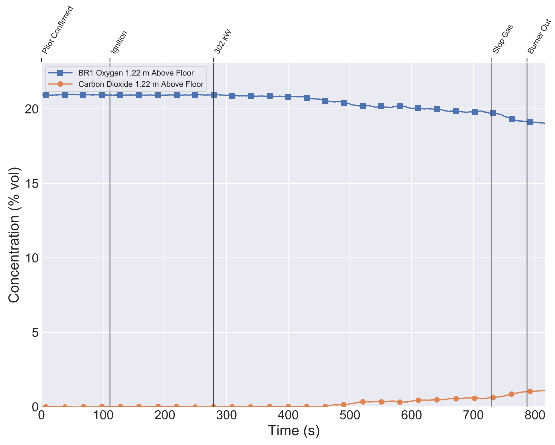

(a) Bedroom 1 Gases

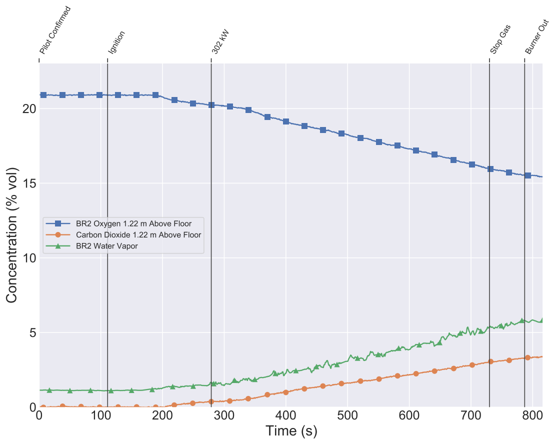

(b) Bedroom 2 Gases

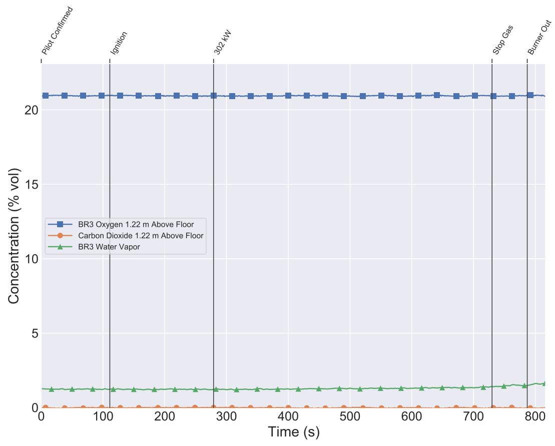

(c) Bedroom 3 Gases

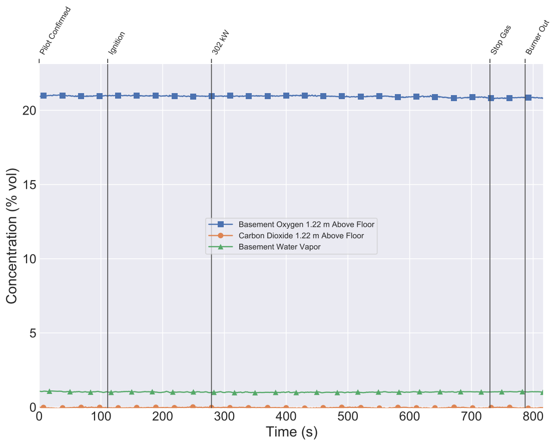

(d) Basement Gases

Figure 5.112: Experiment 19 Bedroom Gases

Figure 5.113 shows the temperatures of the supply and return vents in the structure. Similar to other Experiments with HVAC set to A/C, the temperatures at the supply vents on the first floor were able to be held at cooler temperatures for several hundred seconds following ignition. Once pressures outside the HVAC supply ductwork overpowered the internal pressures, the temperatures at those vents would sharply increase to the temperature in the room. From Figure 5.113(c) it can be seen

that the HVAC system attempted to compensate for the increasing temperature, as was noted in previous experiments.

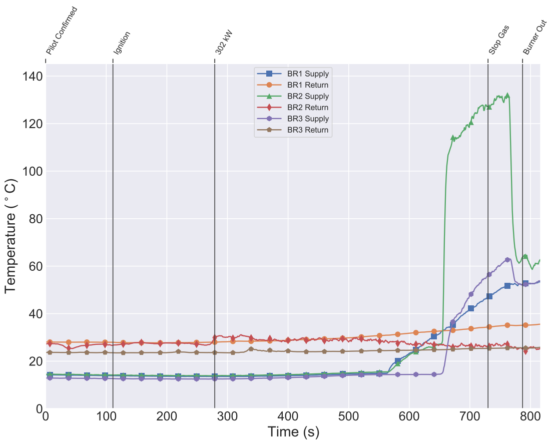

(a) Bedroom Vent Temperatures

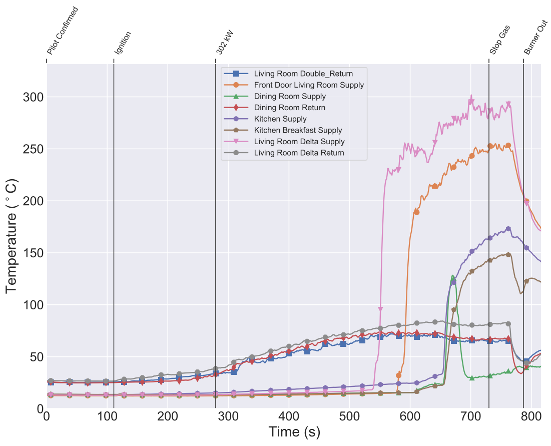

(b) First Floor Vent Temperatures

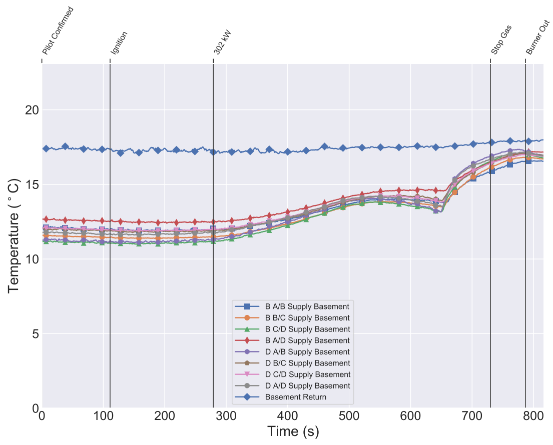

(c) Basement Vent Temperatures

Figure 5.113: Experiment 19 Vent Temperatures

The temperature and velocity within the supply duct are provided in Figure 5.114. Temperatures within the supply duct were able to remain at consistently cool temperatures for the majority of the experiment but began to increase sharply prior to termination of the gas burner. Similarly, it can be seen that the velocity

within the supply duct had a steep decrease to a negative flowrate indicating flow of air from the first floor into the basement.

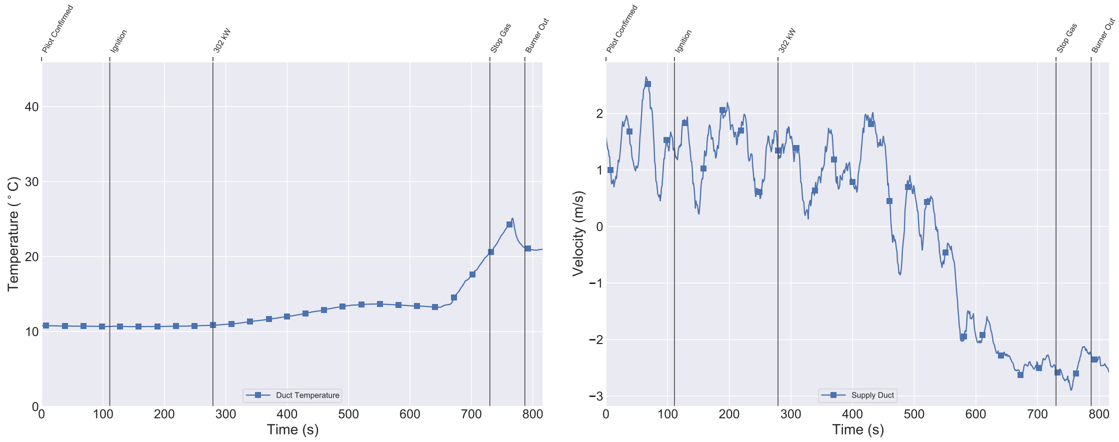

(a) Supply Duct Temperature (b) Supply Duct Velocity

Figure 5.114: Experiment 19 Supply Duct Temperature and Velocity

5.2.6 Experiment 20

Gas Burner Experiment 20 was performed with the HVAC system off. The flow rate of propane to the gas burner was controlled so that an initial heat release rate of approximately 121 kW was produced and then ramped up to 393 kW. The doors to bedroom 1, bedroom 3 and the stairwell door to the basement were closed during this experiment. All exterior vents were closed. Louver vents were installed above the doorways to bedroom 1 and bedroom 3. Gas concentration measurements on the first floor were taken in bedroom 1, bedroom 2 and bedroom 3. Experiment 20 was performed to observe the effects of a higher HRR on heat movement through the structure with louver vents above the closed bedroom doors. Comparisons will be made with Experiment 18. The events of interest for Experiment 20 are presented

in Table 5.20.

Table 5.20: Experiment 20 Events

Event	Elapsed Time (seconds)
Pilot Confirmed	0
Ignition	101
Increased HRR	244
Stop Gas	792
Burner Out	861

The time versus temperature graphs from the first floor are provided in Figure 5.115. Even with a higher HRR and the installation of the louver vents above the closed bedroom doors, temperatures on the first floor were similar in trends and values to those recorded in Experiment 18.

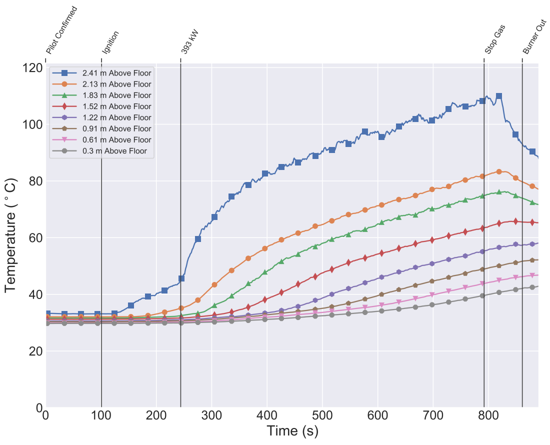

(a) Bedroom 1 Temperatures

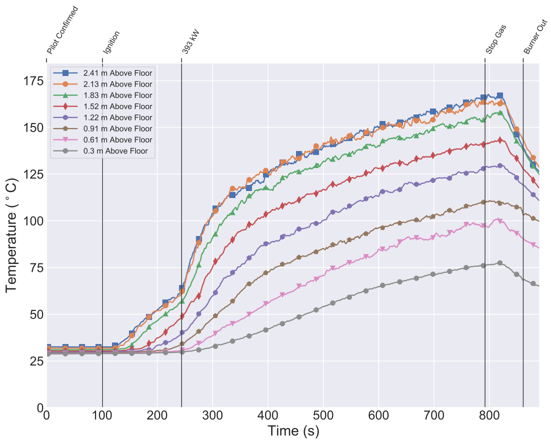

(b) Bedroom 2 Temperatures

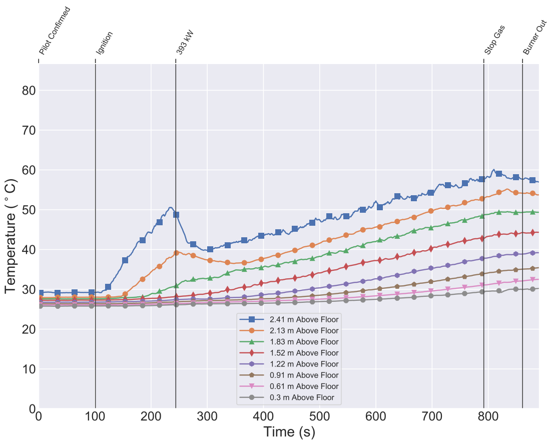

(c) Bedroom 3 Temperatures

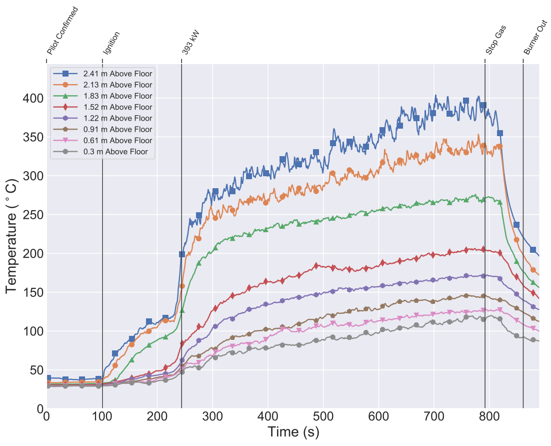

(d) Living Room Temperatures

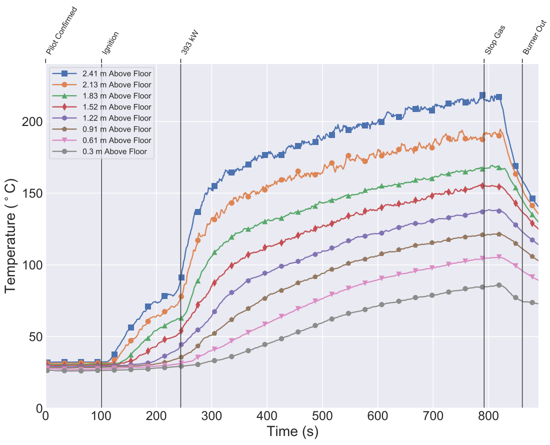

(e) Dining Room Temperatures

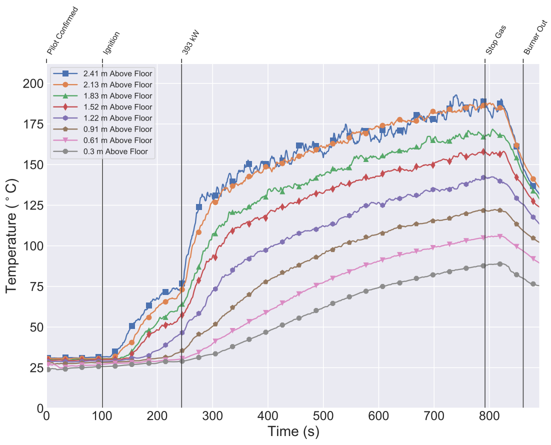

(f) Kitchen Temperatures

Figure 5.115: Experiment 20 First Floor Temperatures

The time versus temperature graphs for the four basement quadrants are provided in Figure 5.116. As was the case with Experiment 88, there was minimal change in basement temperatures.

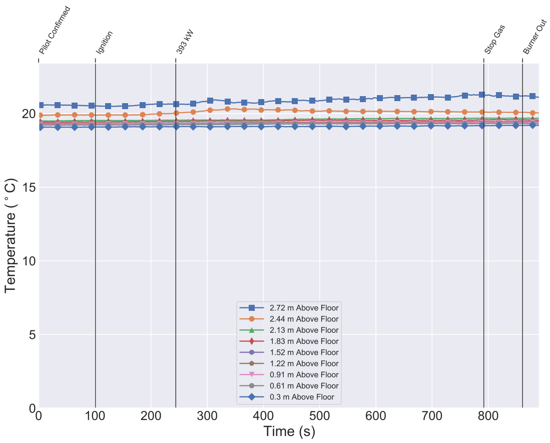

(a) Basement A Quadrant Temperatures

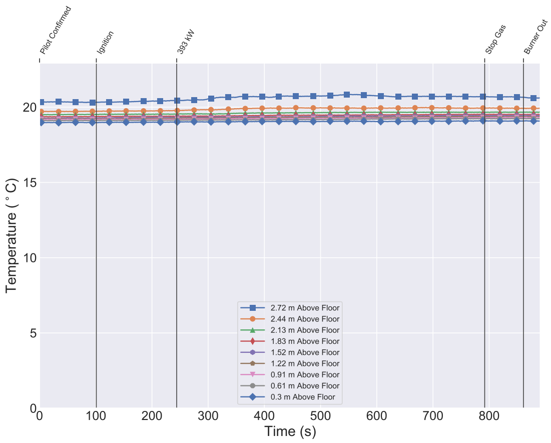

(b) Basement B Quadrant Temperatures

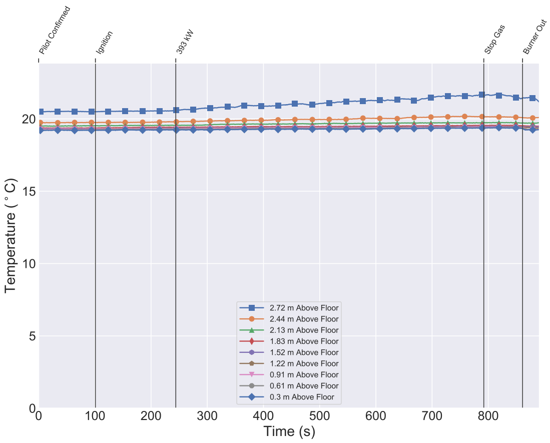

(c) Basement C Quadrant Temperatures

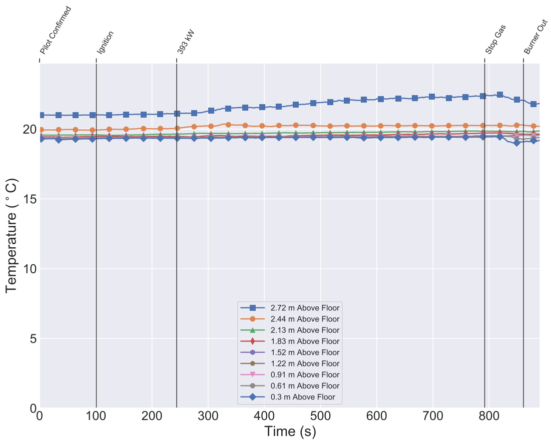

(d) Basement D Quadrant Temperatures

Figure 5.116: Experiment 20 Basement Temperatures

The pressures within each room are shown in Figure 5.117. Pressures in this experiment behaved similar to those recorded in Experiment 18.

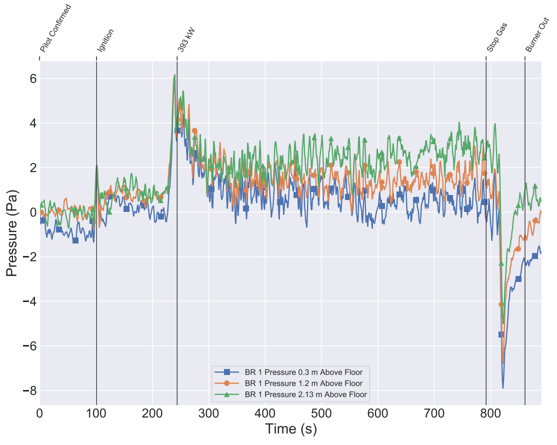

(a) Bedroom 1 Pressures

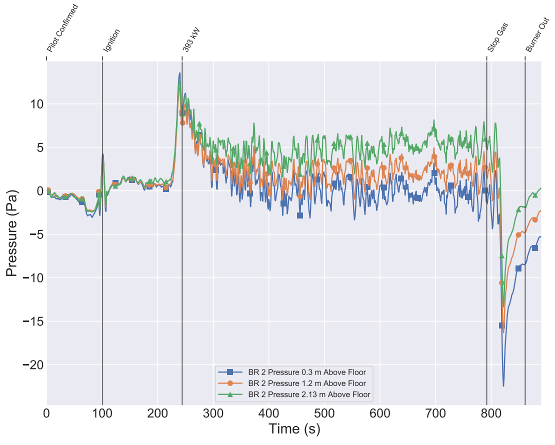

(b) Bedroom 2 Pressures

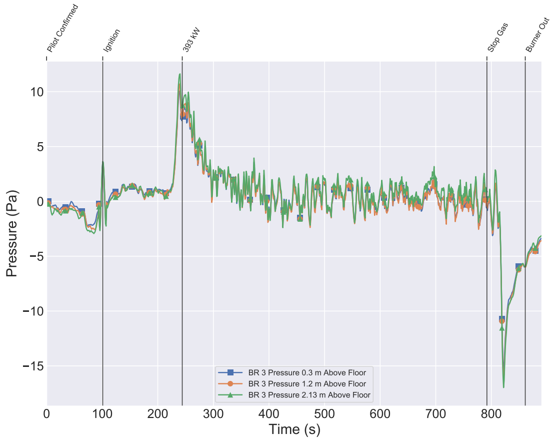

(c) Bedroom 3 Pressures

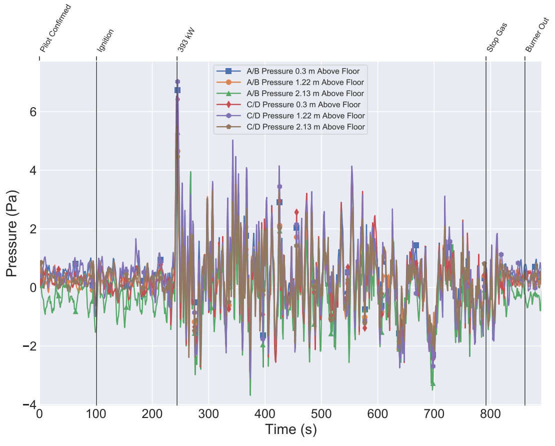

(d) Basement Pressures

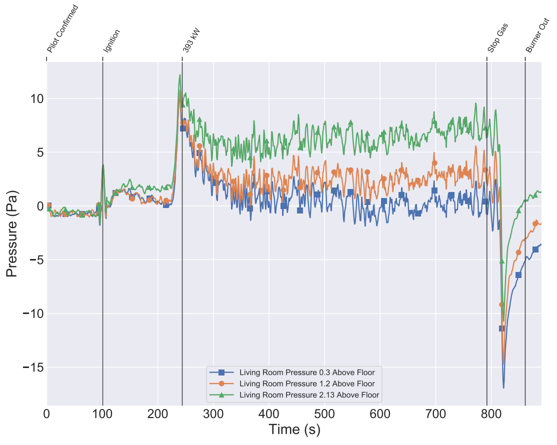

(e) Living Room Pressures

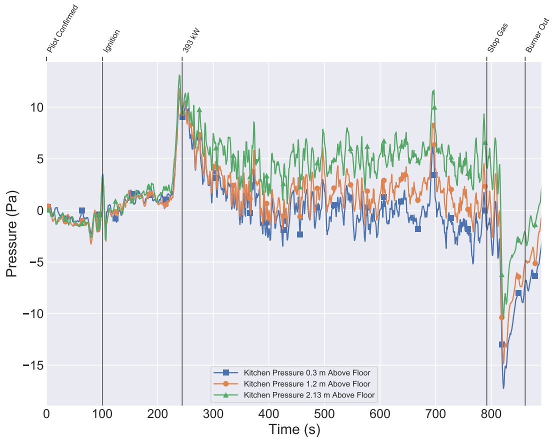

(f) Kitchen Pressures

Figure 5.117: Experiment 20 Pressures

The gas concentrations of oxygen and carbon dioxide are provided for bedroom 1, bedroom 2, bedroom 3 and the basement in Figure 5.118. Water vapor concentrations are provided for bedroom 2, bedroom 3 and the basement. Oxygen concentrations in bedroom 1 dropped to approximately 17% during the experiment. Carbon dioxide levels in bedroom 1 increased approximately 2% during the experiment. In bedroom 2, oxygen concentrations dropped to approximately 14%, carbon dioxide increased approximately 5% and water vapor increased approximately 7%. Gas concentrations in bedroom 3 remained steady and water vapor increased by only 1% during the experiment. Gas and vapor concentrations in the basement did not change during this experiment. The difference in gas concentration behavior between bedroom 1 and bedroom 3 suggests that there is more leakage into bedroom 1 than bedroom 3 with both having their doorway closed and a louver vent above it.

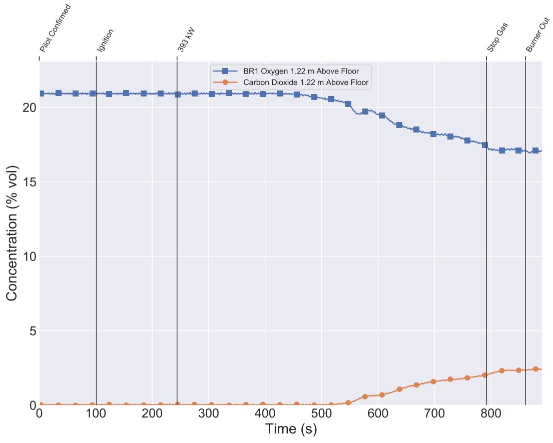

(a) Bedroom 1 Gases

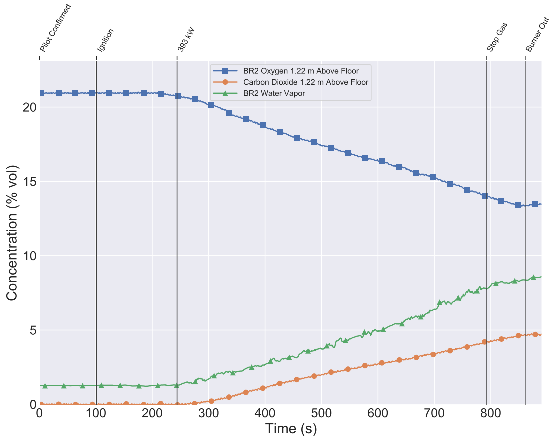

(b) Bedroom 2 Gases

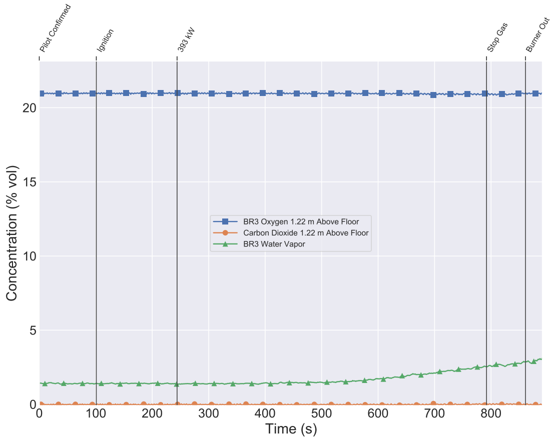

(c) Bedroom 3 Gases

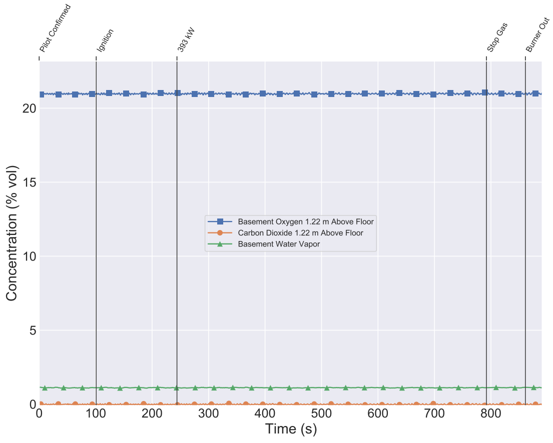

(d) Basement Gases

Figure 5.118: Experiment 20 Bedroom Gases

Figure 5.119 shows the temperatures of the supply and return vents in the structure. Vent temperatures throughout the structure behaved in a similar manner to the data recorded in Experiment 18.

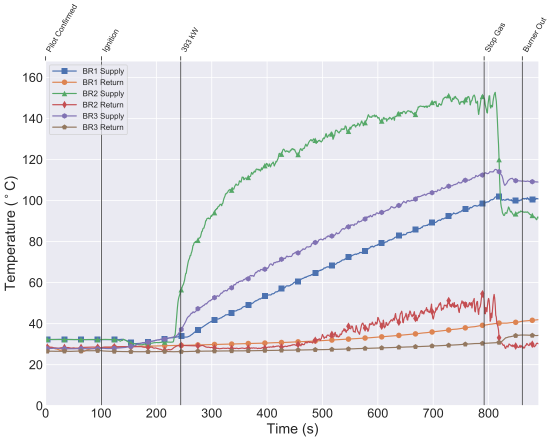

(a) Bedroom Vent Temperatures

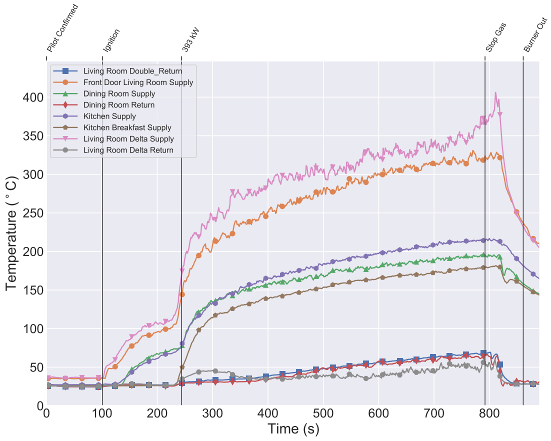

(b) First Floor Vent Temperatures

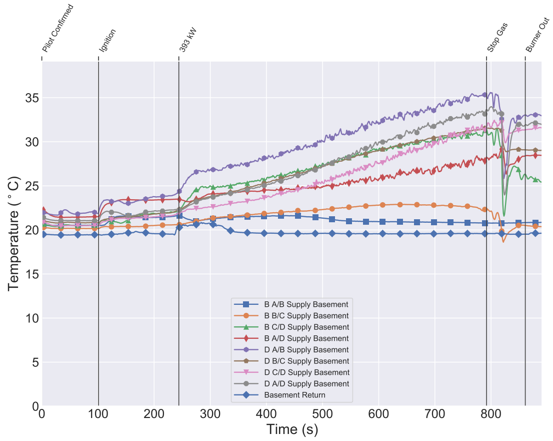

(c) Basement Vent Temperatures

Figure 5.119: Experiment 20 Vent Temperatures

The temperature and velocity within the supply duct are provided in Figure 5.120. Temperatures and velocities within the supply duct were similar between this experiment and Experiment 18.

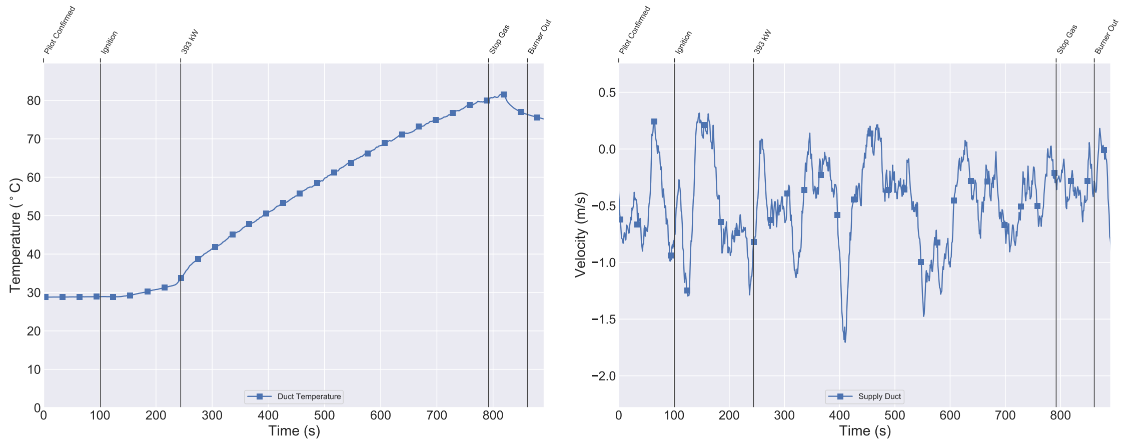

(a) Supply Duct Temperature

(b) Supply Duct Velocity

Figure 5.120: Experiment 20 Supply Duct Temperature and Velocity

5.2.7 Experiment 21

Gas Burner Experiment 21 was performed with the HVAC system set to A/C. The flow rate of propane to the gas burner was controlled so that an initial heat release rate of approximately 121 kW was produced and then ramped up to 302 kW. The doors to bedroom 1, bedroom 3 and the stairwell door to the basement were closed during this experiment. All exterior vents were closed. Louver vents were installed above the doorways to bedroom 1 and bedroom 3. Gas concentration measurements on the first floor were taken in bedroom 1, bedroom 2 and bedroom 3. Experiment 21 was performed to compare the results to Experiment 20 to see the effects of the HVAC system being set to A/C with a higher HRR. The events of interest for Experiment 21 are presented in Table 5.21.

Table 5.21: Experiment 21 Events

Event	Elapsed Time (seconds)
Pilot Confirmed	0
Ignition	178
Increased HRR	318
Stop Gas	709
Burner Out	756

The time versus temperature graphs from the first floor are provided in Figure 5.121. Similar to Experiment 19, The HVAC system set to A/C was able to keep the temperatures in the bedrooms cooler by approximately 10 °C than the temperatures in Experiment 20. Temperatures in the living room, dining room and kitchen were unaffected by the HVAC system.

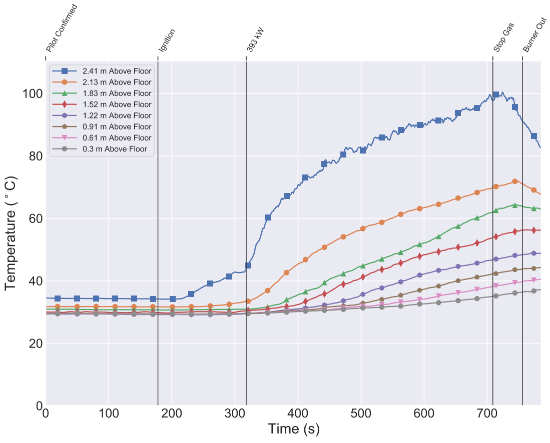

(a) Bedroom 1 Temperatures

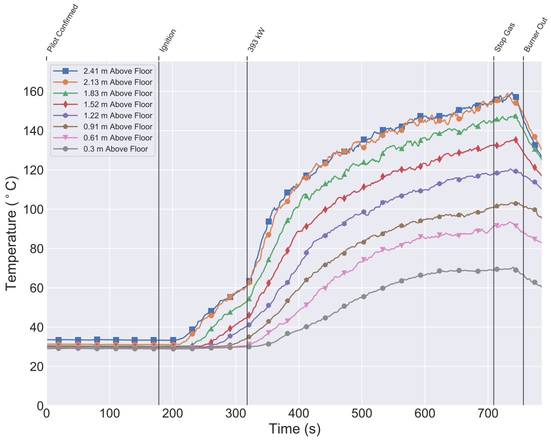

(b) Bedroom 2 Temperatures

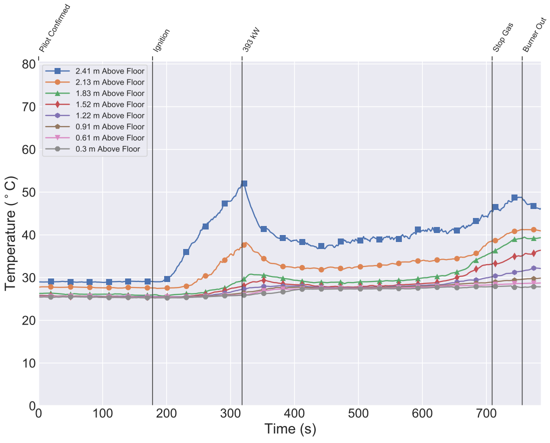

(c) Bedroom 3 Temperatures

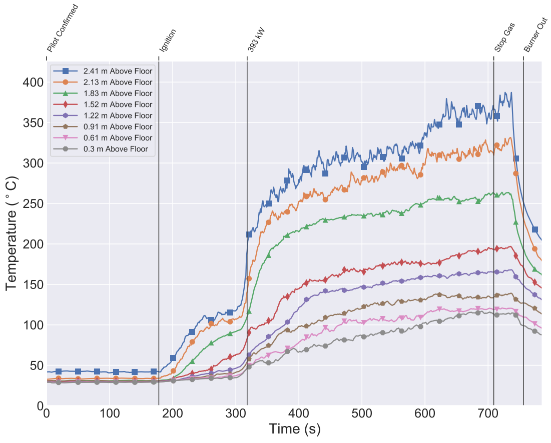

(d) Living Room Temperatures

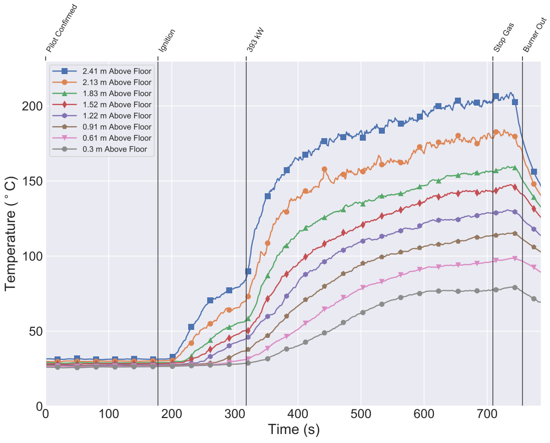

(e) Dining Room Temperatures

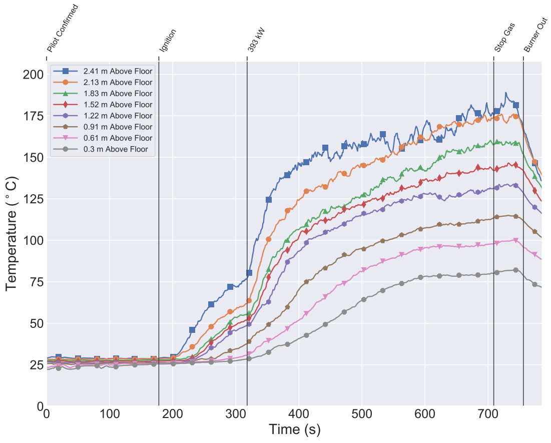

(f) Kitchen Temperatures

Figure 5.121: Experiment 21 First Floor Temperatures

The time versus temperature graphs for the four basement quadrants are provided in Figure 5.122. Temperatures in the basement remained relatively constant throughout the experiment.

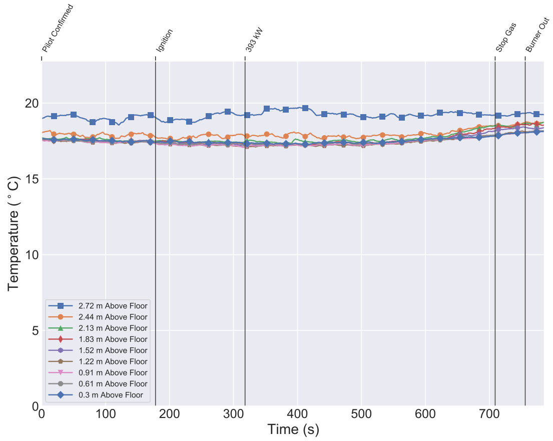

(a) Basement A Quadrant Temperatures

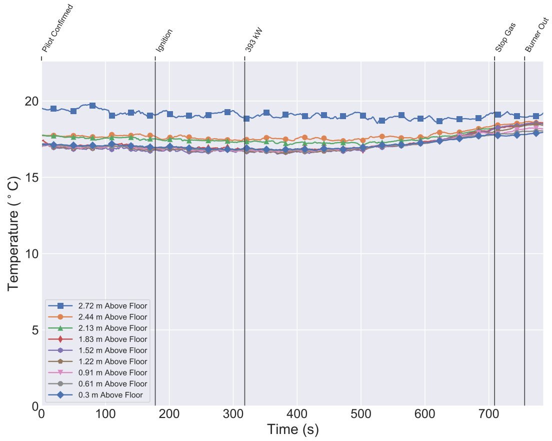

(b) Basement B Quadrant Temperatures

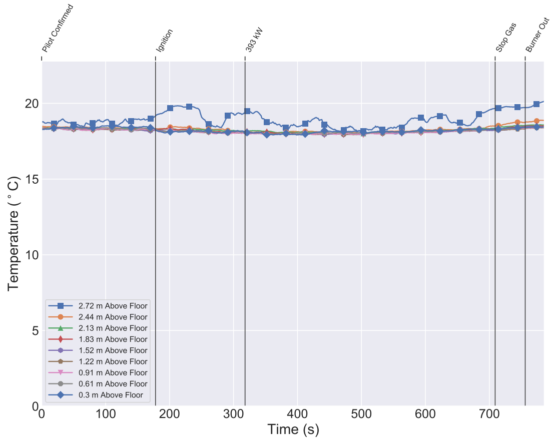

(c) Basement C Quadrant Temperatures

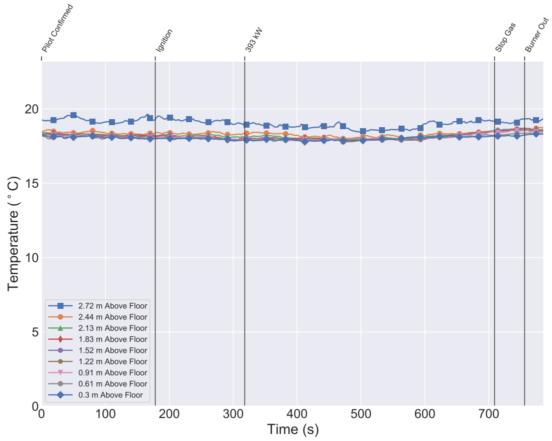

(d) Basement D Quadrant Temperatures

Figure 5.122: Experiment 21 Basement Temperatures

The pressures within each room are shown in Figure 5.123. Pressures in the structure were not affected by the HVAC system when comparing between this experiment and the previous experiment.

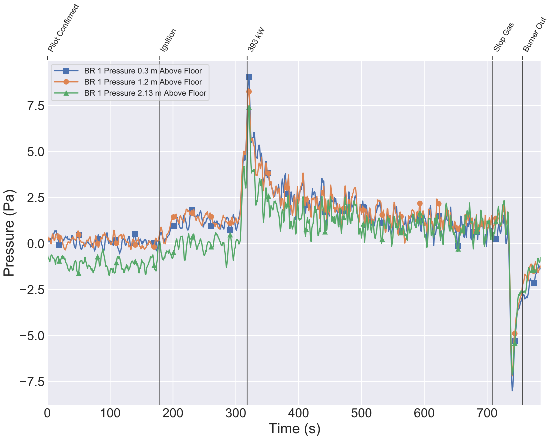

(a) Bedroom 1 Pressures

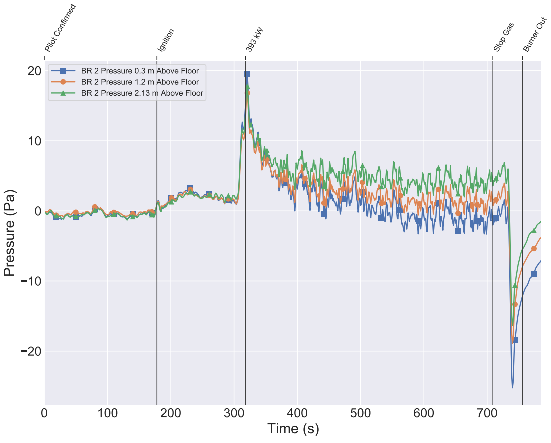

(b) Bedroom 2 Pressures

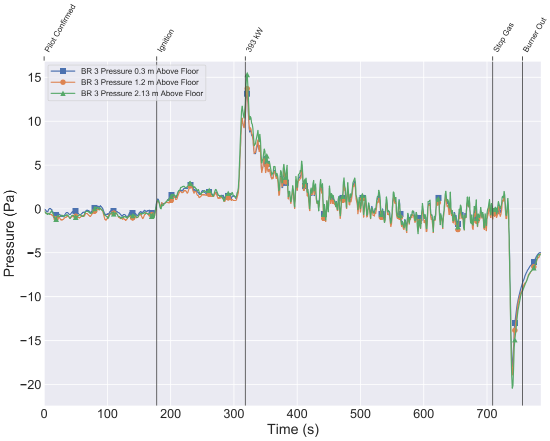

(c) Bedroom 3 Pressures

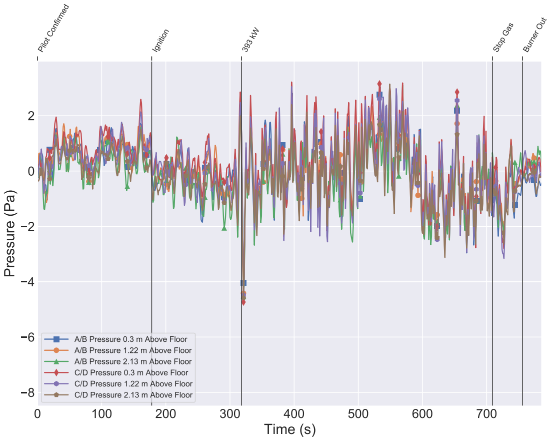

(d) Basement Pressures

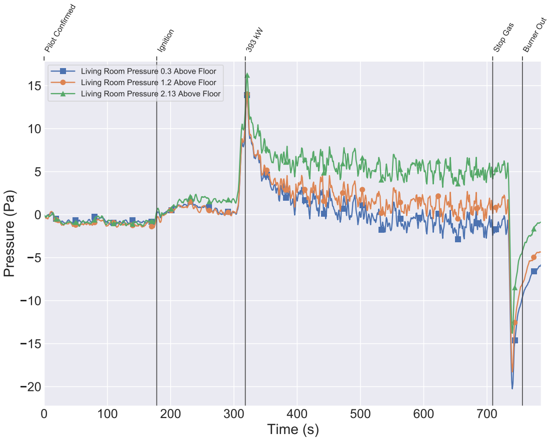

(e) Living Room Pressures

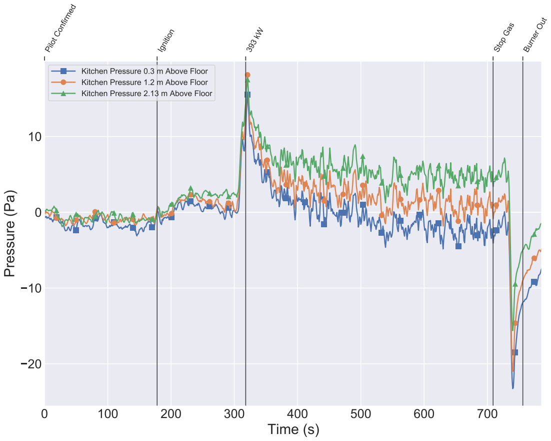

(f) Kitchen Pressures

Figure 5.123: Experiment 21 Pressures

The gas concentrations of oxygen and carbon dioxide are provided for the bedroom 1, bedroom 2, bedroom 3 and the basement in Figure 5.124. Water vapor concentrations are provided for bedroom 2, bedroom 3 and the basement. Gas concentrations in bedroom 1 were affected less with the HVAC system set to A/C than they were with the HVAC system turned off. Oxygen levels in bedroom 1 decreased only by approximately 1% during the experiment. Bedroom 2 gas and vapor concentrations behaved in a similar manner to that of Experiment 20. Gas and vapor concentrations in bedroom 3 and the basement remained steady and unchanged throughout the experiment.

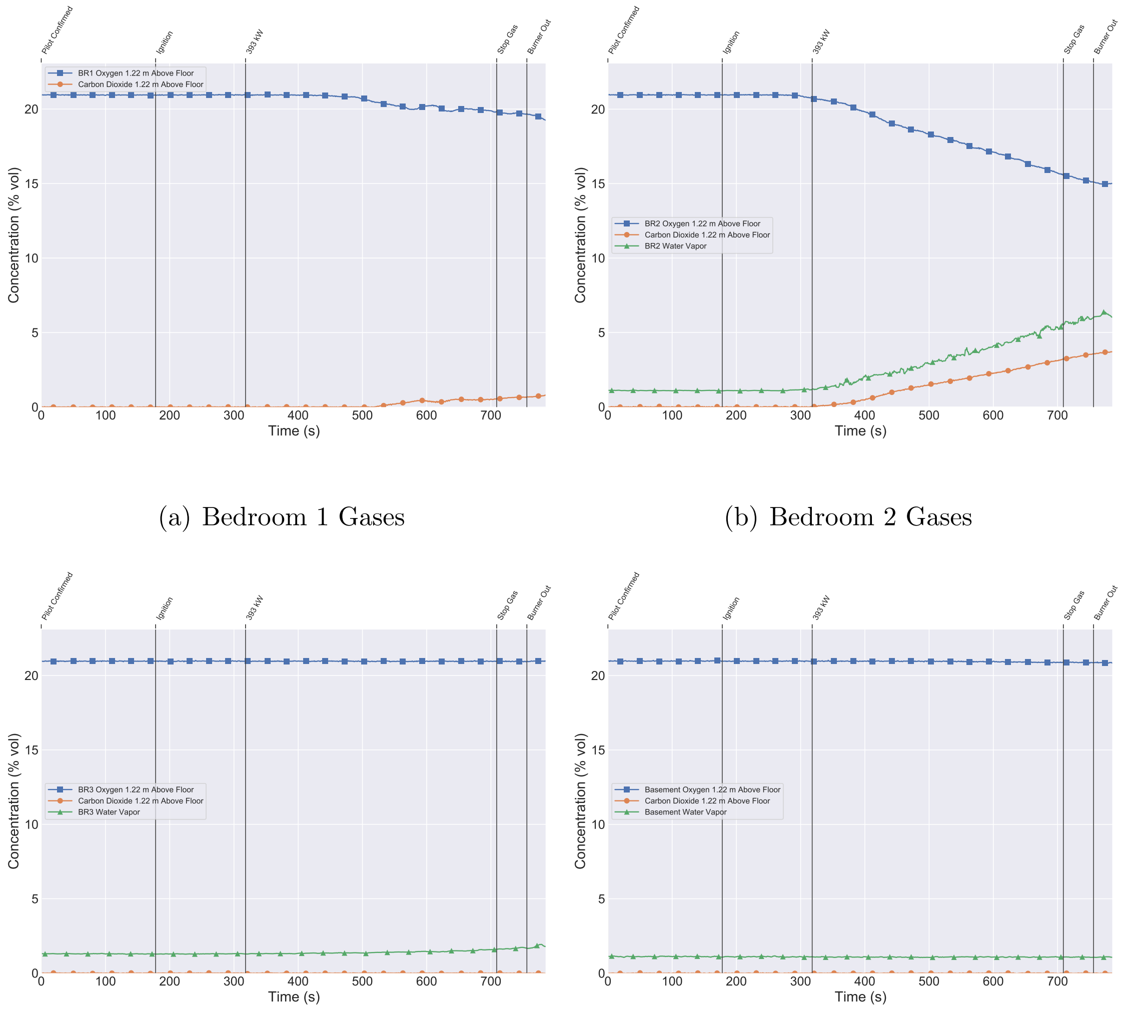

(a) Bedroom 1 Gases

(b) Bedroom 2 Gases

(c) Bedroom 3 Gases

(d) Basement Gases

Figure 5.124: Experiment 21 Bedroom Gases

Figure 5.125 shows the temperatures of the supply and return vents in the structure. Supply vent temperatures across the first floor were held at cooler temperatures for several hundred seconds until the pressures within their respective rooms were able to overcome the pressure generated within the HVAC supply ducts. Return vent temperatures were similar to the temperatures at a similar height in their respective rooms. Basement supply vent temperatures displayed similar trends

to that identified in Experiment 19.

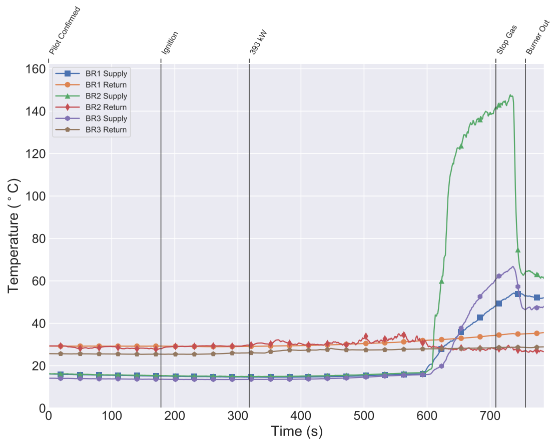

(a) Bedroom Vent Temperatures

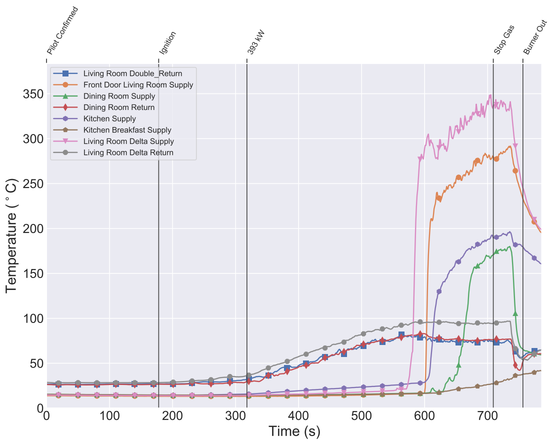

(b) First Floor Vent Temperatures

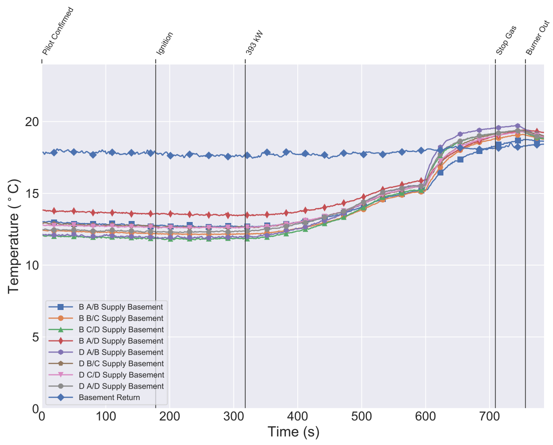

(c) Basement Vent Temperatures

Figure 5.125: Experiment 21 Vent Temperatures

The temperature and velocity within the supply duct are provided in Figure 5.126. Supply duct temperatures remained relatively constant throughout most of the experiment with an increasing slope towards the end of the experiment. Similarly, the velocity in the duct showed an increased negative velocity as the experiment progressed.

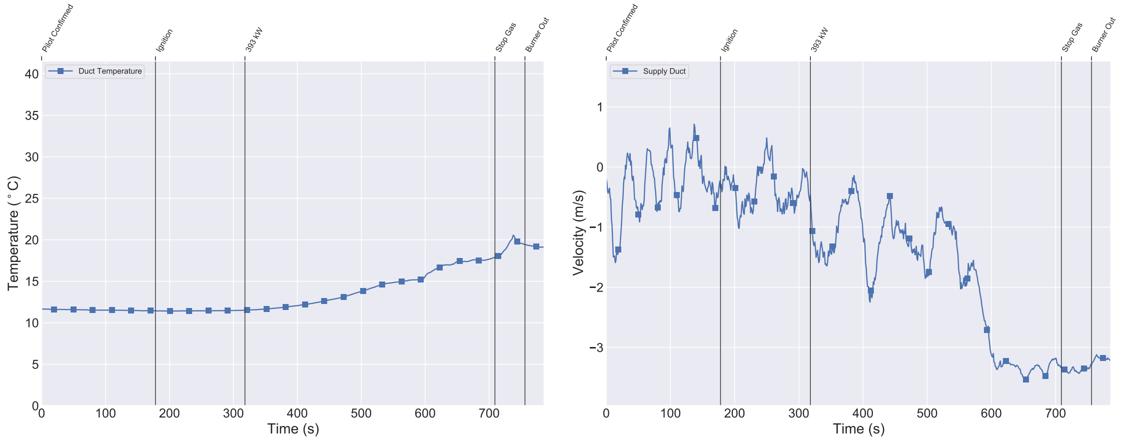

(a) Supply Duct Temperature

(b) Supply Duct Velocity

Figure 5.126: Experiment 21 Supply Duct Temperature and Velocity

5.3 Basement Experiments

Gas Burner Experiments 23 through 29 were performed with the gas burner located in the basement on the D side of the structure. For all basement experiments louver vents were installed above the doorways to bedroom 1 and bedroom 3. The door to bedroom 3 was closed for all experiments along with all exterior vents. The doorway to bedroom 2 was open for all experiments. Three gas concentration measurements were obtained on the first floor and one measurement in the basement. Gas measurements on the first floor were obtained in bedrooms 1, 2 and 3. The basement gas concentration measurement was located on the B side of the structure near the sliding door. Durarock was secured to the ceiling above the burner to prevent fire from spreading into the floor joists. Gas burner Experiment 22 was excluded from analysis because the experiment was terminated due to instrumentation issues.

Over the course of the basement fire experiments, it was noted that the HVAC filters would be clogged by soot deposition during experiments where the HVAC system was on. The clogged filters would cause reduced airflow into the HVAC fan that would create a negative pressure in the system. In experiments where this change was noted, the gas flow would be terminated due to concern of damaging the system. The termination of gas flow correlates to the 'Stop Gas' event.

5.3.1 Experiment 23

Gas Burner Experiment 23 was performed with the HVAC system off. The flow rate of propane to the gas burner was controlled so that a heat release rate of approximately 302 kW was produced. The stairwell door to the basement was open during this experiment. The doorway to bedroom 1 was closed for this experiment. Experiment 23 was performed to see how smoke and fire gases moved through the structure via the HVAC system. The events of interest for Experiment 23 are presented in Table 5.22.

Table 5.22: Experiment 23 Events

Event	Elapsed Time (seconds)
Pilot Confirmed	0
Ignition	94
Stop Gas	1235
Burner Out	1279

The time versus temperature graphs for Experiment 23 are provided below. Figure 5.127 displays the basement temperatures and Figure 5.128 displays the first floor temperatures.

Temperatures in the A/B corner of the basement increased approximately 150 °C at the 2.7 m height and increased approximately 30 °C at the 0.3 m height. Temperatures in the B/C corner of the basement increased approximately 125 °C at the 2.7 m height and approximately 25 °C at the 0.3 m height. Temperatures in the C/D corner of the basement increased approximately 275 °C at the 2.7 m height and approximately 50 °C at the 0.3 m height. Temperatures in the A/D corner of the basement increased approximately 210 °C at the 2.7 m height and approximately 40 °C at 0.3 m while the burner was on. Temperatures in the mechanical room, located on the D side of the basement near the gas burner, increased approximately 25 °C at the 2.7 m height and approximately 15 °C at the 0.3 m height.

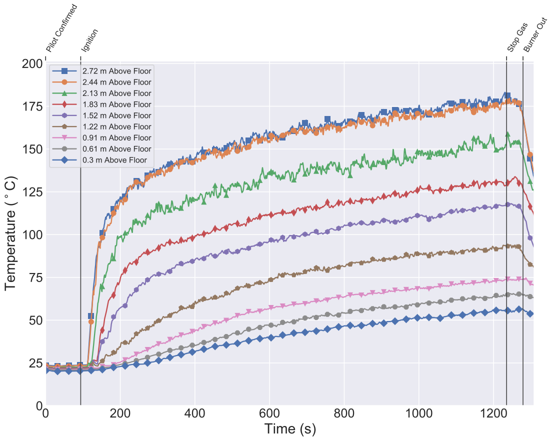

(a) Basement A/B Corner Temperatures

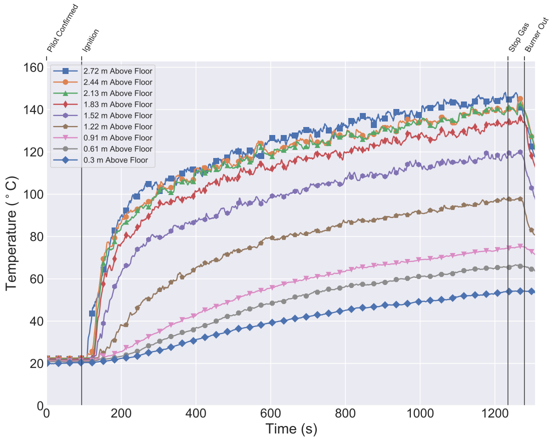

(b) Basement B/C Corner Temperatures

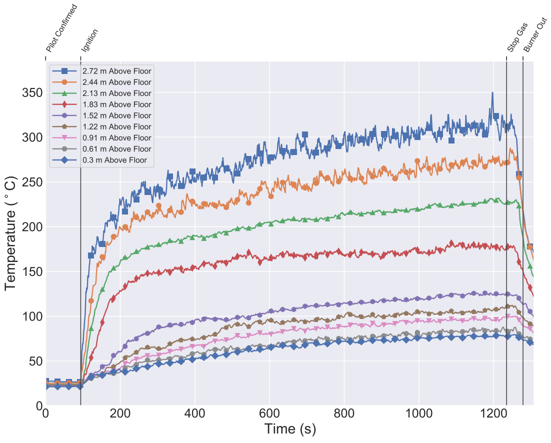

(c) Basement C/D Corner Temperatures

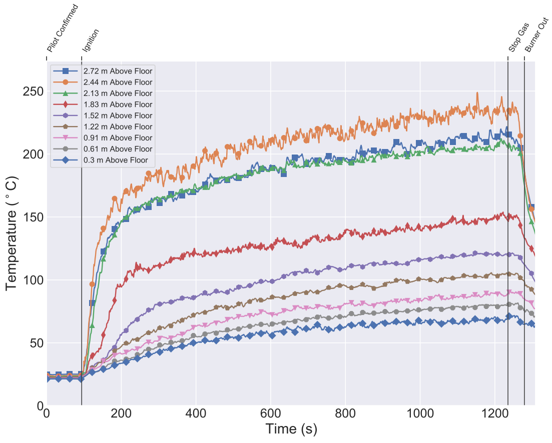

(d) Basement A/D Corner Temperatures

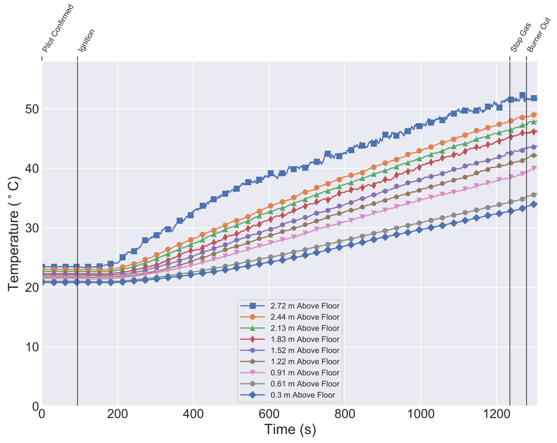

(e) Basement Mechanical Room Temperatures

Figure 5.127: Experiment 23 Basement Temperatures

Temperatures in Bedroom 1 at the 2.4 m height increased to approximately 40 °C while the burner was on and temperatures at the 0.3 m height increased by only a few degrees celcius. Temperatures in bedroom 2 increased to approximately 55 °C at the 2.4 m height and to approximately 40 °C at the 0.3 m height while the burner was on. Temperatures in bedroom 3 increased to approximately 45 °C at the 2.4 m height and increased by approximately 5 °C at the 0.3 m height. Temperatures in the living room at the 2.4 m height reached approximately 70 °C while the burner was on and increased to approximately 50 °C at the 0.3 m height. Temperatures in the dining room at the 2.4 m height reached approximately 80 °C while the burner was on and increased to approximately 40 °C at the 0.3 m height. Temperatures in the kitchen at the 2.4 m height reached approximately 95 °C while the burner was on and increased to approximately 45 °C at the 0.3 m height. Temperature increases in the living room, dining room, kitchen and bedroom 2 appear to be due to convective heating through the open stairwell door and openings on the first floor. Temperatures increases in bedrooms 1 and 3 could be due to heating through the HVAC ductwork or possibly though the louver vents installed above their doors.

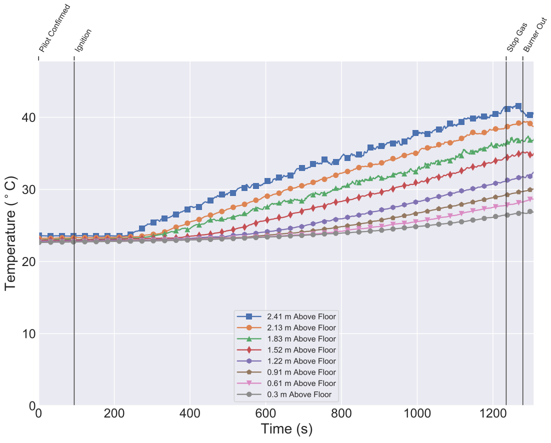

(a) Bedroom 1 Temperatures

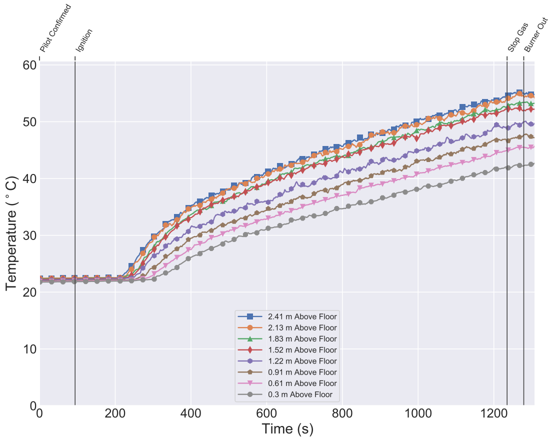

(b) Bedroom 2 Temperatures

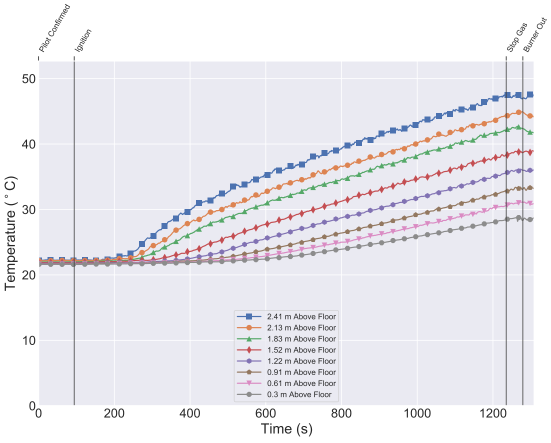

(c) Bedroom 3 Temperatures

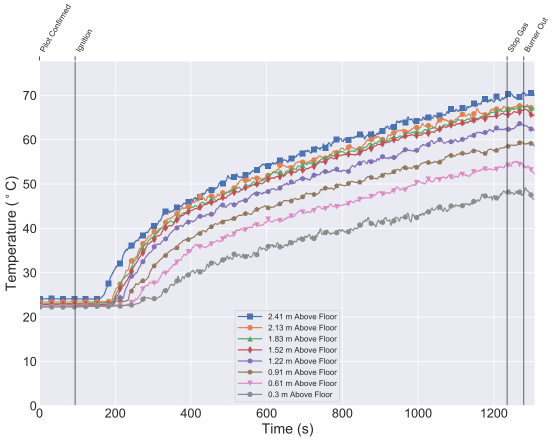

(d) Living Room Temperatures

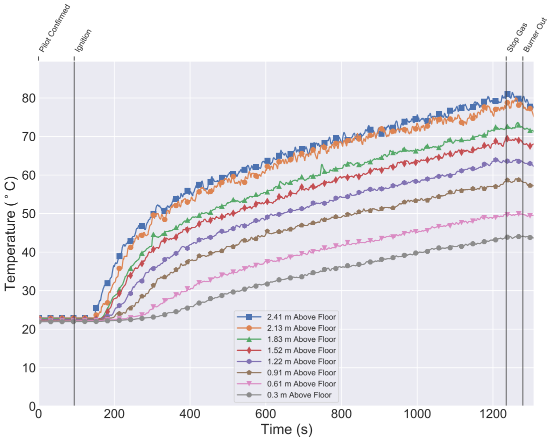

(e) Dining Room Temperatures

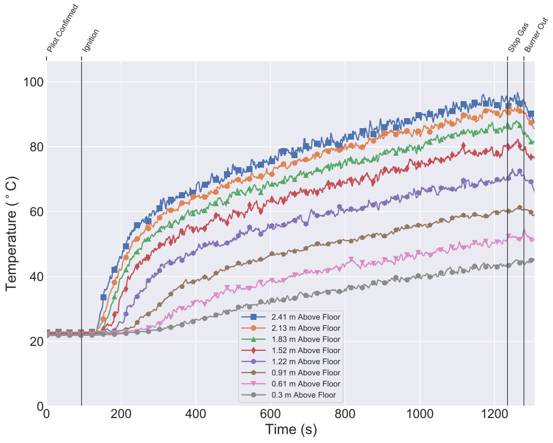

(f) Kitchen Temperatures

Figure 5.128: Experiment 23 First Floor Temperatures

The pressures within each room are shown in Figure 5.129. Pressure in bedroom 1 exceeded the ambient pressure by approximately 1 to 3 Pa at all heights while the burner was on with a spike of approximately 15 Pa following ignition. Pressures in bedroom 2 exceeded the ambient pressure by approximately 3 Pa at all heights and spiked to approximately 20 Pa following ignition. Pressures in bedroom 3 were similar at all heights to that of bedroom 2. Pressures in the basement at the 2.13 m height hovered around 10 Pa above the ambient pressure. Pressures at the 1.2 m varied from the ambient pressure by approximately -15 to -20 Pa. At the 0.3 m height pressures trended negative to a pressure of approximately -30 Pa below the ambient. Pressure in the living room exceeded the ambient pressure by approximately 1 to 5 Pa with a spike of approximately 18 Pa following ignition. Pressures in the kitchen followed a similar trend to the pressures in the living room with a spike following ignition around 20 Pa above the ambient.

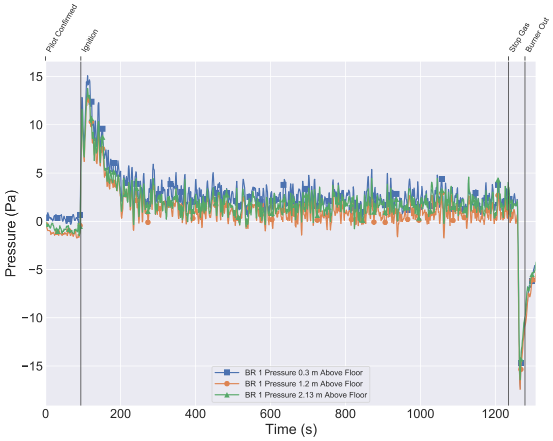

(a) Bedroom 1 Pressures

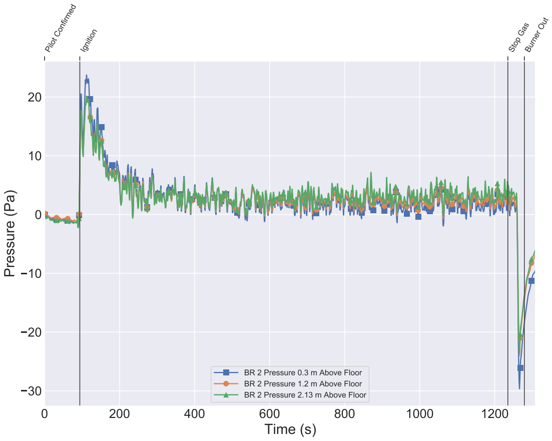

(b) Bedroom 2 Pressures

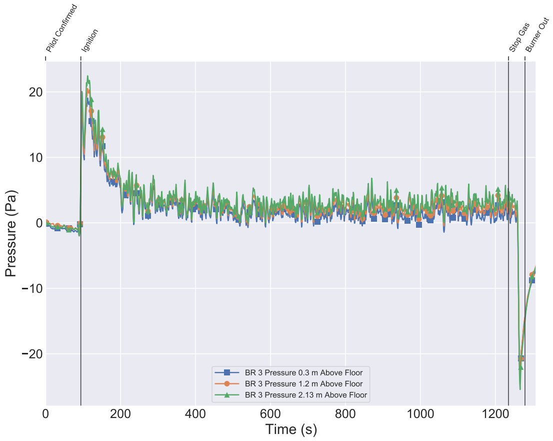

(c) Bedroom 3 Pressures

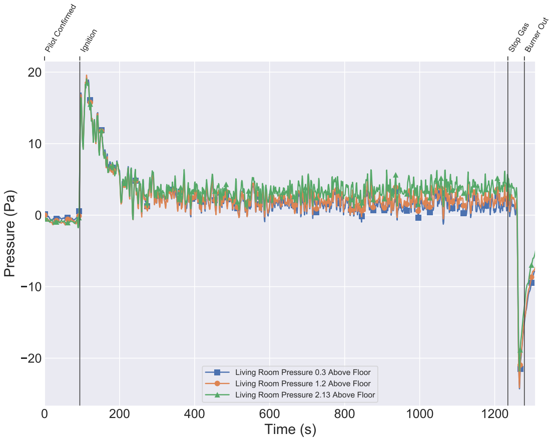

(d) Living Room Pressures

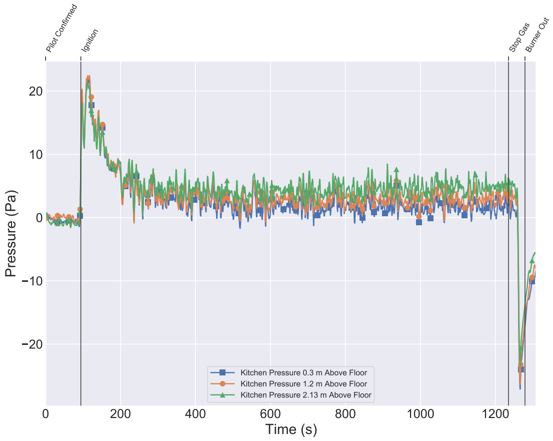

(e) Kitchen Pressures

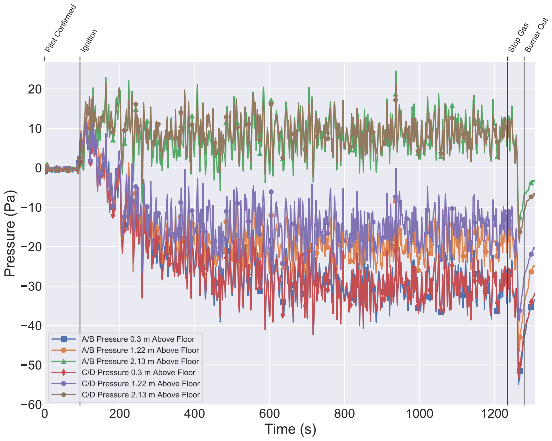

(f) Basement Pressures

Figure 5.129: Experiment 23 Pressures

The gas concentrations of oxygen and carbon dioxide are provided for bedroom 1, bedroom 2, bedroom 3 and the basement in Figure 5.130. Water vapor concentrations are provided for bedroom 2, bedroom 3 and the basement. Oxygen concentrations in bedroom 1 decreased to approximately 18% while the burner was on. Carbon dioxide concentrations in bedroom 1 increased to approximately 2% during this experiment. Oxygen concentrations in bedroom 2 dropped to approximately 16% while the burner was on. Carbon dioxide concentrations in bedroom 2 rose by 3% while the gas burner was on. Oxygen concentrations in bedroom 3 dropped slightly towards the end of the experiment but never dropped below 20%. Carbon dioxide levels appeared to have a slight increase towards the end of the experiment but did not show any significant change. Oxygen levels in the basement dropped to approximately 17% while the gas burner was on. Carbon dioxide levels increased by approximately 3% in the basement during the experiment.

Water vapor concentrations in bedroom 2 increased by approximately 3% during this experiment. Water vapor concentrations in bedroom 3 showed a slight increase of approximately 1% while the gas burner was on. Water vapor concentrations in the basement appeared to increase by approximately 5% but had some spikes of almost 10% during the experiment. At multiple points during this experiment the basement water vapor instrumentation was not able to produce usable data points. This could have been due to an excessive amounts of smoke around the laser sensor or water condensation interferring with the sensor windows.

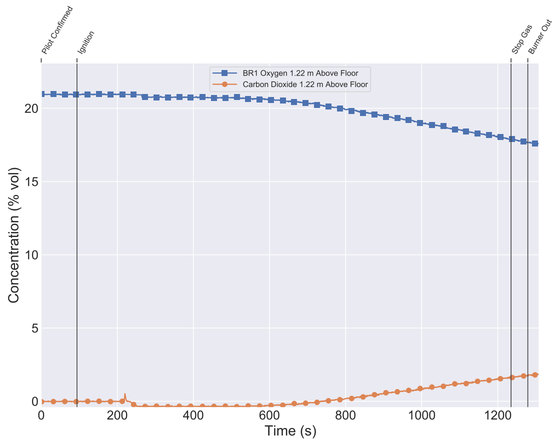

(a) Bedroom 1 Gases

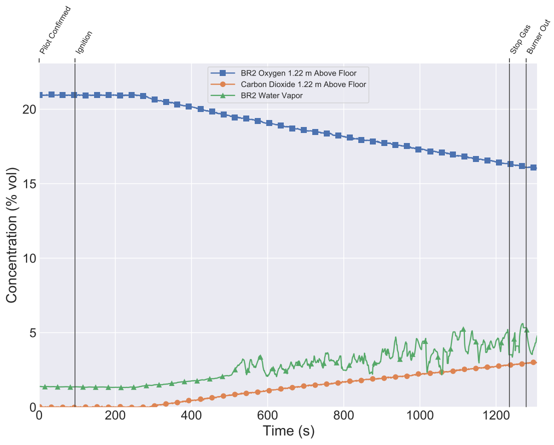

(b) Bedroom 2 Gases

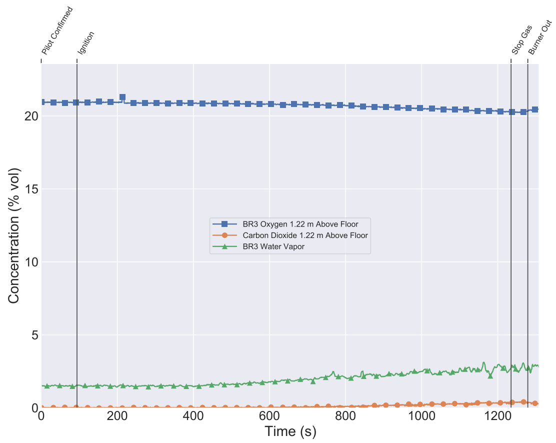

(c) Bedroom 3 Gases

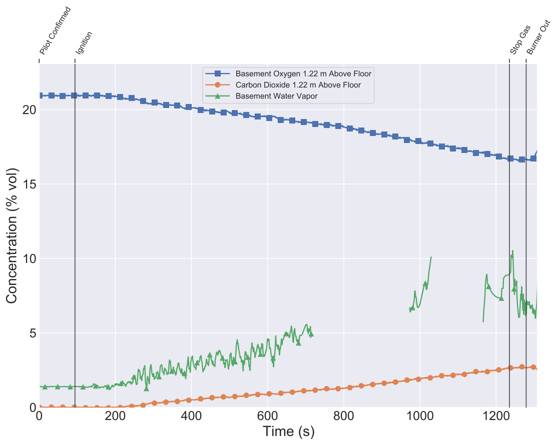

(d) Basement Gases

Figure 5.130: Experiment 23 Bedroom Gases

Figure 5.131 shows the temperatures of the supply and return vents in the structure. From Figure 5.131(a) it can be seen that the supply vent in bedroom 3 is consistantly warmer than the rest of the temperatures in bedroom 3. This indicates that heating of bedroom 3 is due to heat transfer through the supply ducts. The bedroom 3 return vent temperature follows the trend of the thermocouple in bedroom 3 at that height, thus indicating that heat transfer to bedroom 3 is not

occurring through the return ducts. The same trends noted for bedroom 3 also occurred in bedroom 1 during this experiment. The supply and return vents in bedroom 2 appear to follow the temperature trends observed at the thermocouple tree located in bedroom 2, thus indicating that heating of bedroom 2 is by convectional currents through the structure and not via the HVAC ductwork.

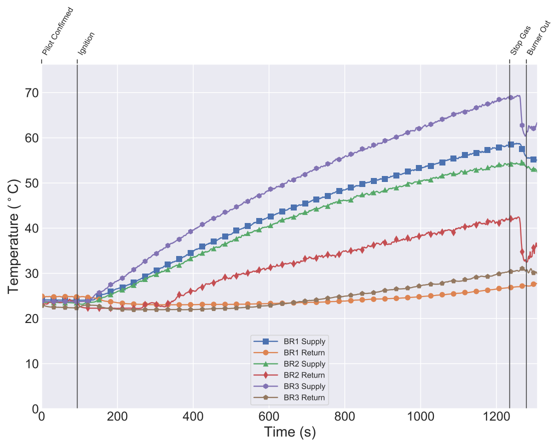

(a) Bedroom Vent Temperatures

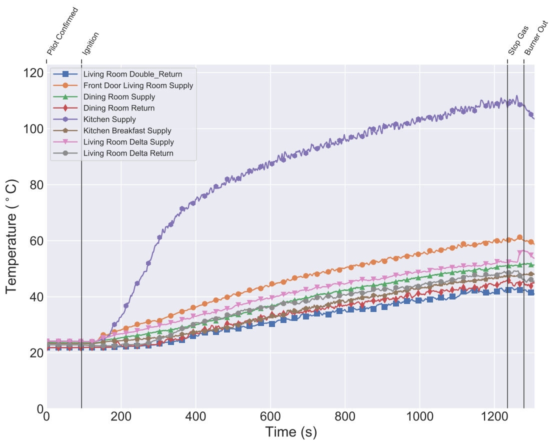

(b) First Floor Vent Temperatures

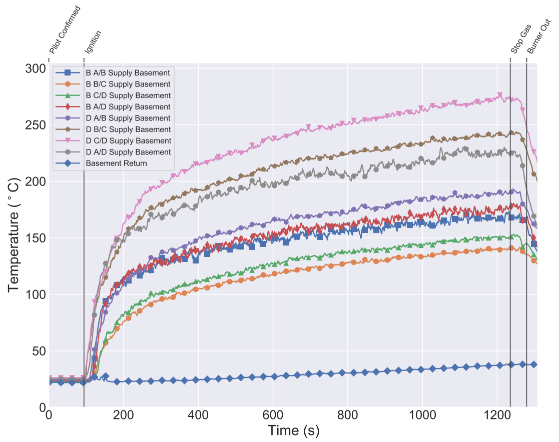

(c) Basement Vent Temperatures

Figure 5.131: Experiment 23 Vent Temperatures

From Figure 5.131(b) it appears that heating of all the other vents on the first

floor are due to convective heat coming up the staircase and spreading throughout the first floor of the structure. The kitchen supply vent located near the top of the staircase reached the highest temperature of vents on the first floor and its temperature was similar to that of the thermocouple at the 2.4 m height in the kitchen. Figure 5.131(c) shows the temperatures for the vents located in the basement. The highest temperature for a supply vent in the basement was approximately 275 °C recorded at supply vent S-07 in the C/D corner of the D side of the basement. Temperatures for all vents in the basement followed the trends of the thermocouple trees located in their respective areas.

The temperature and velocity within the supply duct are provided in Figure 5.132. From this figure it can be seen that there is a temperature increase within the supply duct of approximately 70 °C while the gas burner was on. The increase in temperature is consistent with what was observed from the supply vent temperatures on the first floor. The supply ducts appear to be the means of heating for bedrooms 1 and 3 behind their closed doors. The supply vent velocity appears to show that there is a constant flow of air in the supply vent from the basement to the first floor, however this remains relatively constant from before ignition to after the burner is out.

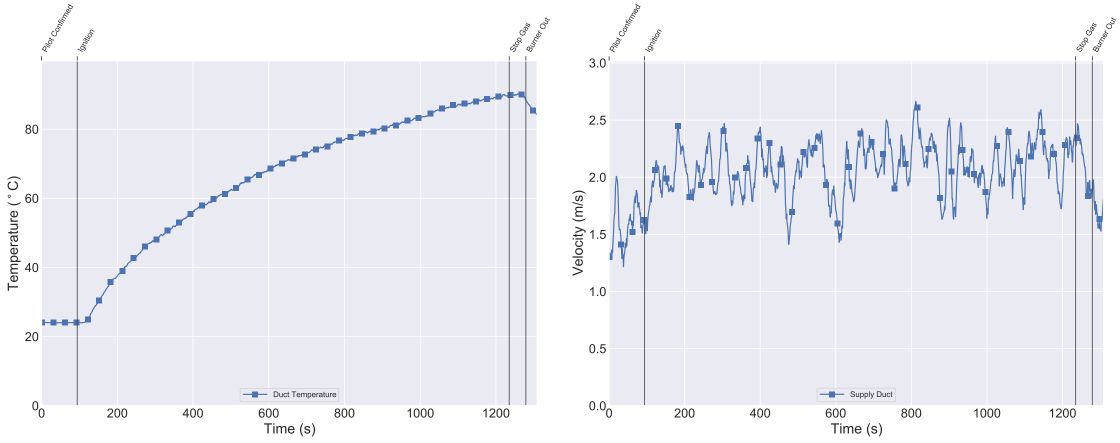

(a) Supply Duct Temperature

(b) Supply Duct Velocity

Figure 5.132: Experiment 23 Supply Duct Temperature and Velocity

5.3.2 Experiment 24

Gas Burner Experiment 24 was performed with the HVAC system set to A/C. The flow rate of propane to the gas burner was controlled so that a heat release rate of approximately 302 kW was produced. The stairwell door to the basement was open during this experiment. The doorway to bedroom 1 and all exterior vents were closed for this experiment. Experiment 24 was performed to see how smoke and fire gases moved through the structure with the HVAC system set to A/C. Comparions will be made to Experiment 23. The events of interest for Experiment 24 are presented in Table 5.23.

Table 5.23: Experiment 24 Events

Event	Elapsed Time (seconds)
Pilot Confirmed	0
Ignition	71
Stop Gas	462
Burner Out	519

The time versus temperature graphs for Experiment 24 are provided below. Figure 5.133 displays the basement temperatures and Figure 5.134 displays the first floor temperatures.

Temperatures in the basement followed a similar trend at all locations. It is important to note the different time scales between Experiment 23 and Experiment 24. Experiment 24 ran for less than half the duration of Experiment 23. The reason this experiment was shorter than the previous experiment is because the HVAC air filter was being clogged by soot as the air handler tried to pull soot polluted air through the conditioning chamber (see Figure). It is worth noting that prior to this experiment, the HVAC air filter had not contributed to the termination of an experiment. The reason that soot played a major role in this scenario is because of the HVAC return in the basement. The basement return vent was responsible for pulling in approximately half of the air that was conditioned as it went through the AHU. This was not an issue when the fire was on the first floor. One possible reason is that there was not enough soot pulled through the first floor returns to affect the AHU. Another possibility is that soot was being deposited along the return ductwork as it travelled from the first floor to the AHU. The basement return was located the closest to the AHU, thus allowing less time for heat and soot dissipation.

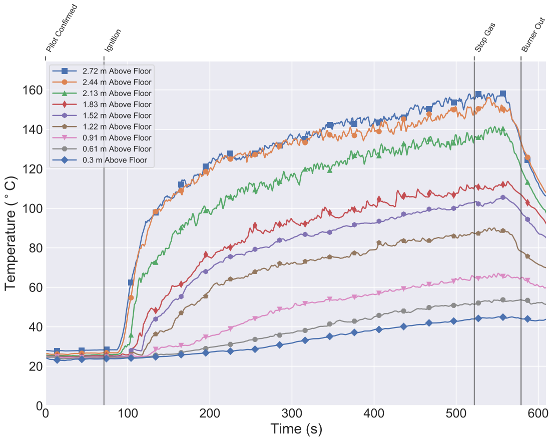

(a) Basement A/B Corner Temperatures

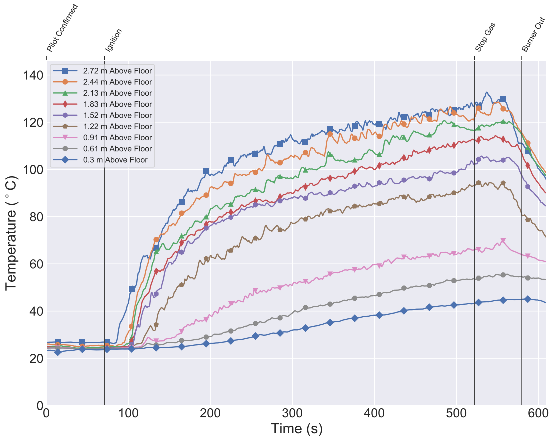

(b) Basement B/C Corner Temperatures

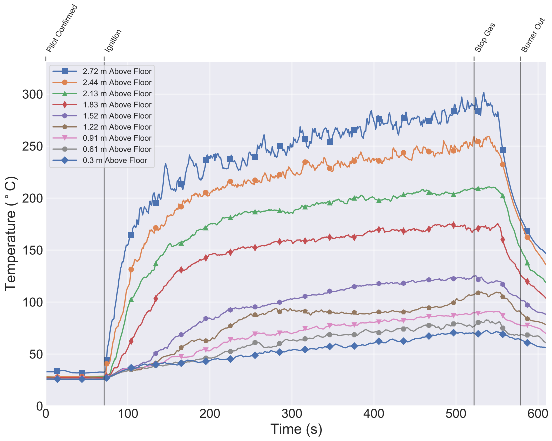

(c) Basement C/D Corner Temperatures

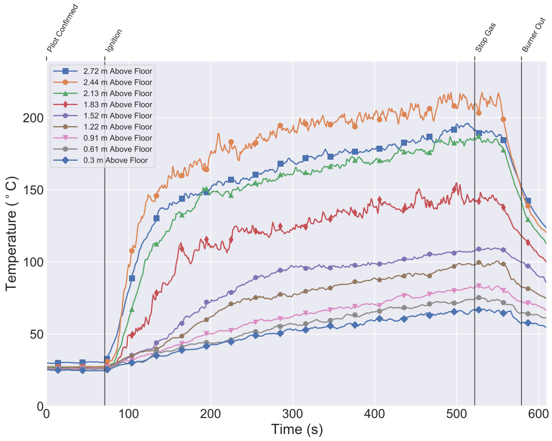

(d) Basement A/D Corner Temperatures

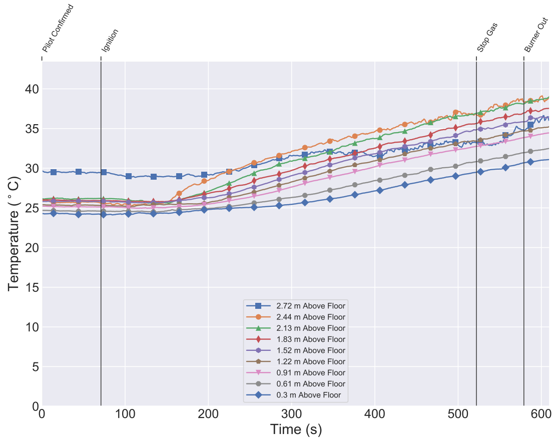

(e) Basement Mechanical Room Temperatures

Figure 5.133: Experiment 24 Basement Temperatures

From the data collected in this experiment, it is hard to determine if the temperatures in bedroom 1 and bedroom 3 were affected by having the HVAC system set to A/C. The louver vents installed above the bedroom doors could allow enough heat to enter the bedrooms to overpower any cooling that could have been provided by the HVAC system. Temperatures at all other locations on the first floor appeared to follow the trends established in Experiment 23.

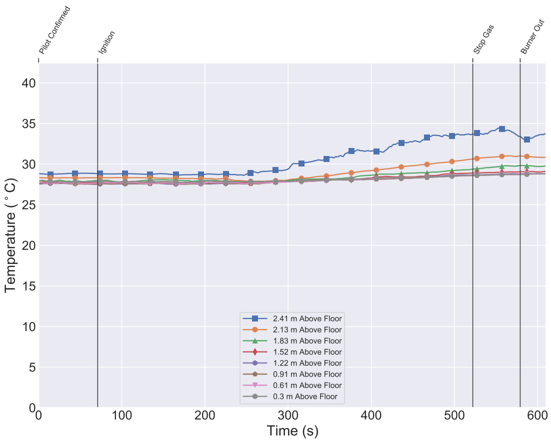

(a) Bedroom 1 Temperatures

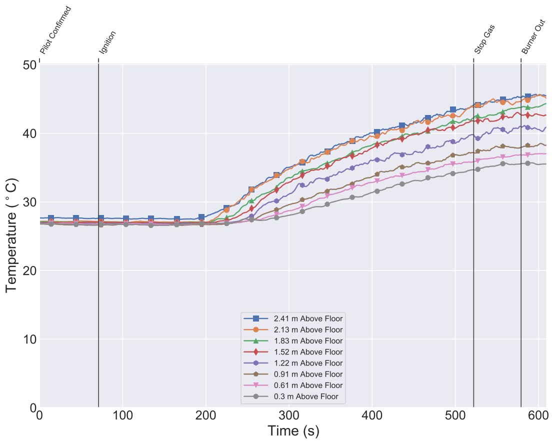

(b) Bedroom 2 Temperatures

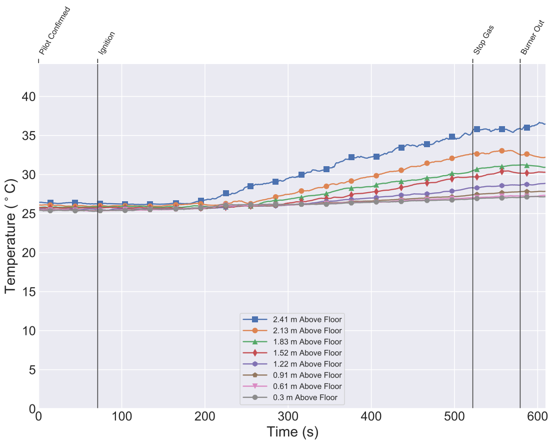

(c) Bedroom 3 Temperatures

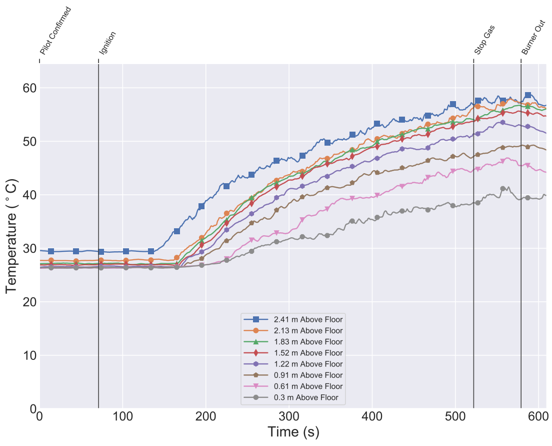

(d) Living Room Temperatures

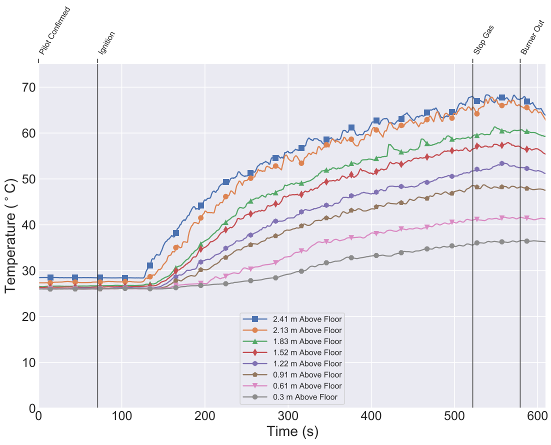

(e) Dining Room Temperatures

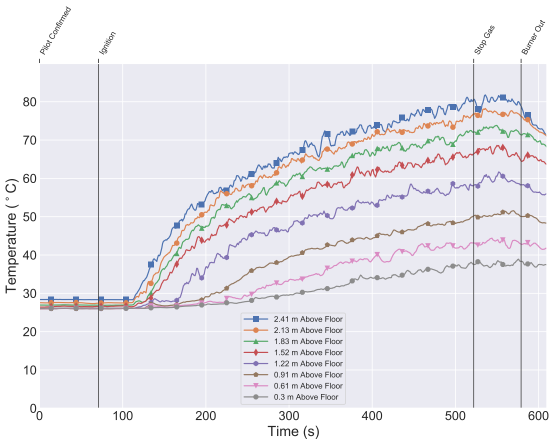

(f) Kitchen Temperatures

Figure 5.134: Experiment 24 First Floor Temperatures

The pressures within each room are shown in Figure 5.135. Pressures within the structure followed a similar trend to the data presented in Experiment 23 with similar values.

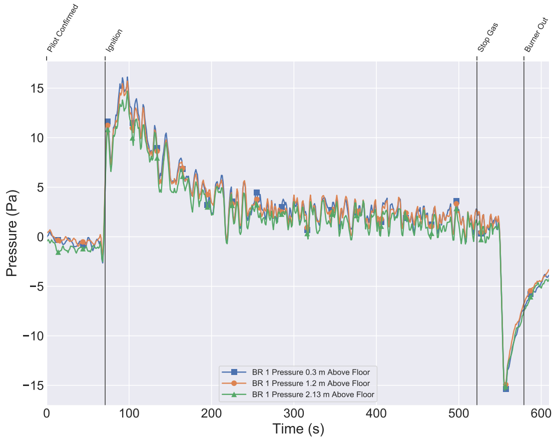

(a) Bedroom 1 Pressures

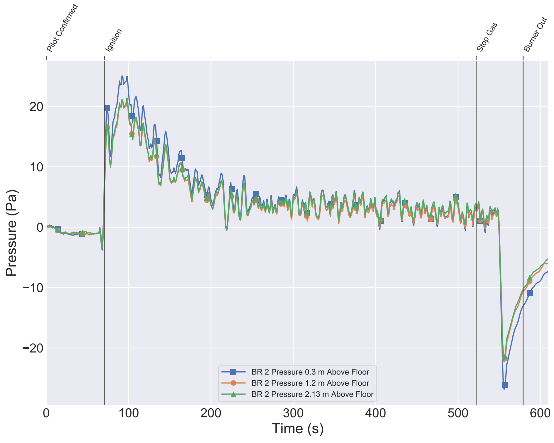

(b) Bedroom 2 Pressures

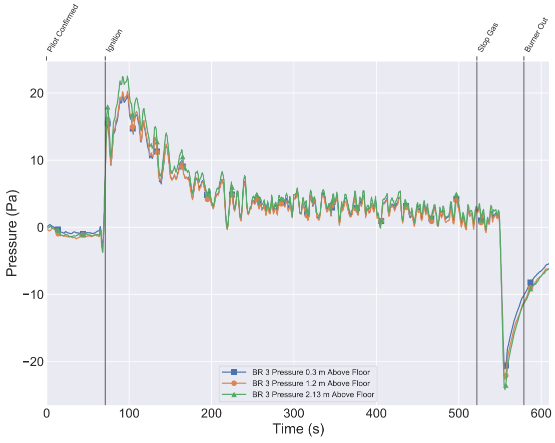

(c) Bedroom 3 Pressures

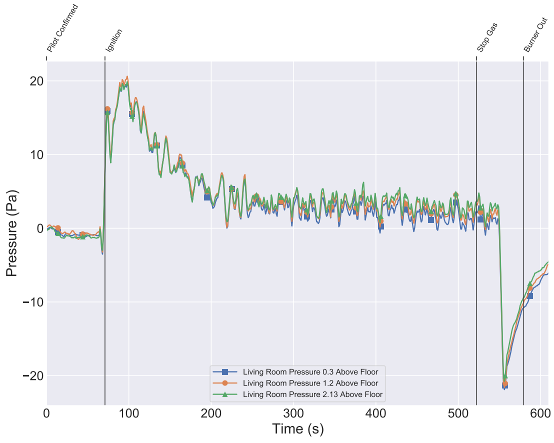

(d) Living Room Pressures

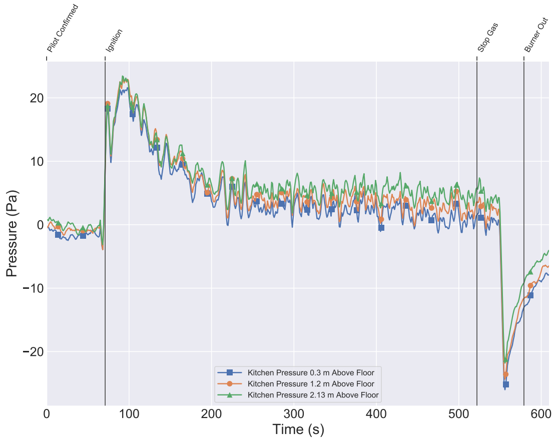

(e) Kitchen Pressures

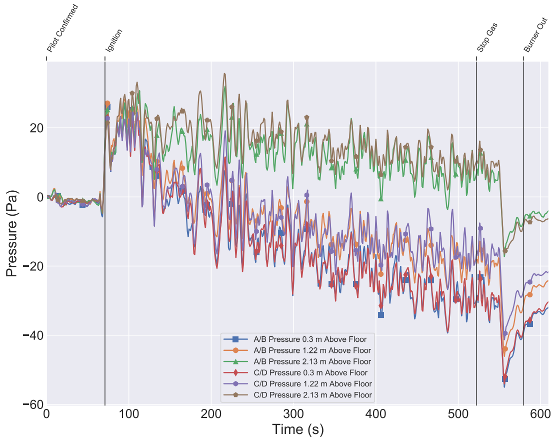

(f) Basement Pressures

Figure 5.135: Experiment 24 Pressures

The gas concentrations of oxygen and carbon dioxide are provided for bedroom 1, bedroom 2, bedroom 3 and the basement in Figure 5.136. Water vapor concentrations are provided for bedroom 2, bedroom 3 and the basement. While gas concentrations in bedrooms 1 and 3 did not appear to change in this experiment, it is possible that with a longer experiment duration that the gas concentrations would have trended like those in Experiment 23. Gas and vapor concentrations in bedroom 2 and the basement follow a similar trend as the data presented in Experiment 23.

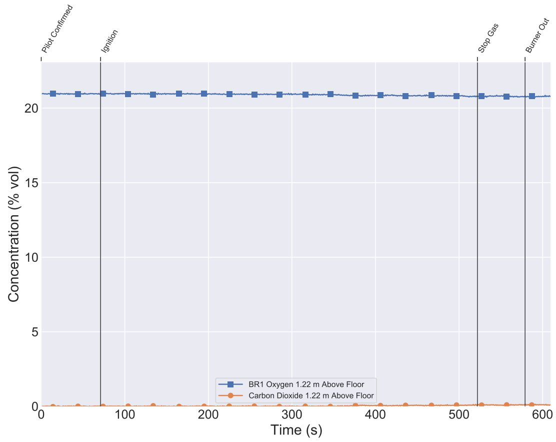

(a) Bedroom 1 Gases

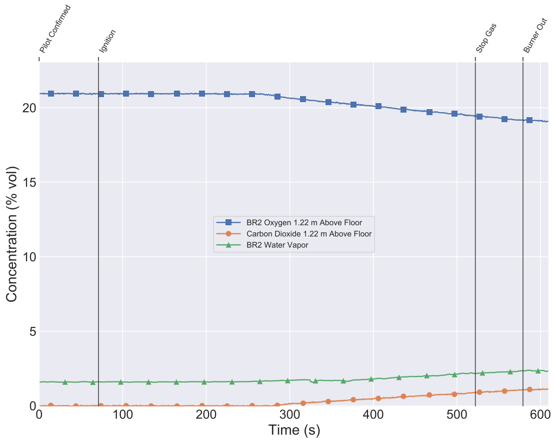

(b) Bedroom 2 Gases

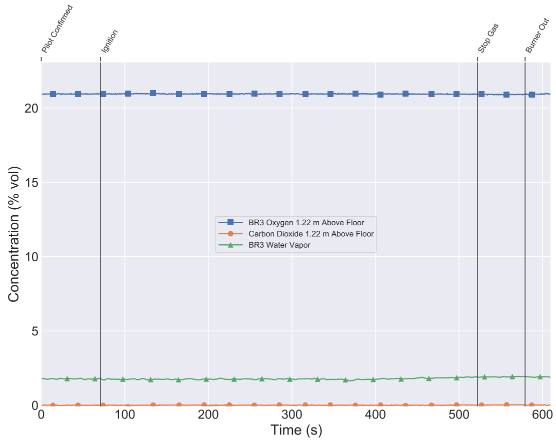

(c) Bedroom 3 Gases

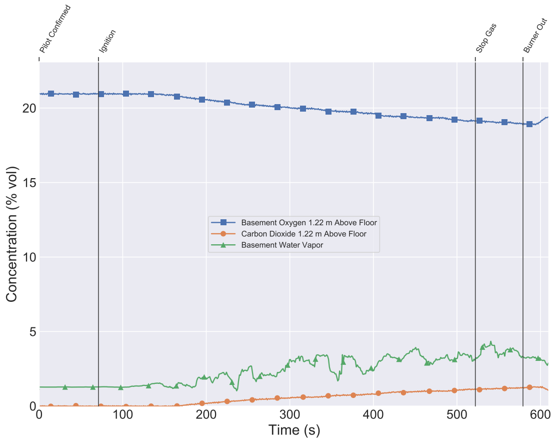

(d) Basement Gases

Figure 5.136: Experiment 24 Bedroom Gases

Figure 5.137 shows the temperatures of the supply and return vents in the structure. It can be seen that the supply vent temperatures in the bedrooms are held at a consideribly lower temperature during this experiment than in Experiment 23. With the exception of the supply vents located in the kitchen and the living room delta supply, all other supply vents on the first floor were maintained at a lower temperature. The supply vents in the kitchen were helded at a lower temperature for a few hundred seconds following ignition before being heated by the air around them. Similarly, the living room delta supply was helt at a cool temperature for over 400 seconds before being heated by the surrounding air. Return vents on the first floor behaved similarly to the temperature in the room at their respective heights.

Figure 5.137(c) shows the temperatures of the supply and return vents located in the basement. From the figure it can be seen that the HVAC system set to A/C was able to maintain cooler temperatures at all the supply vents for this experiment. What is interesting to note is that the return vent temperature was exceptionally higher and more turbulent in this experiment than in Experiment 23. A possible cause of this could be that with the supply vents pushing air out at the ceiling, the thermal layer was disrupted, specifically in the area near the return vent, thus causing mixed temperature air to enter the vent.

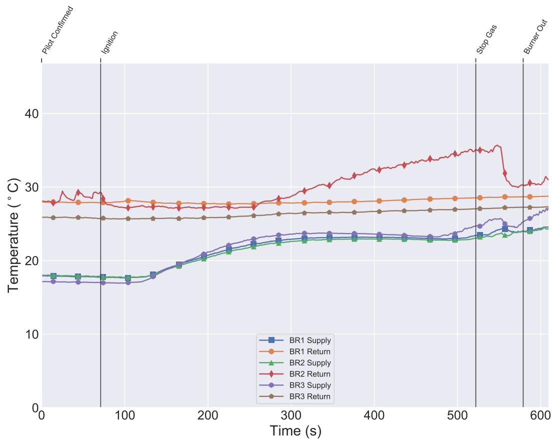

(a) Bedroom Vent Temperatures

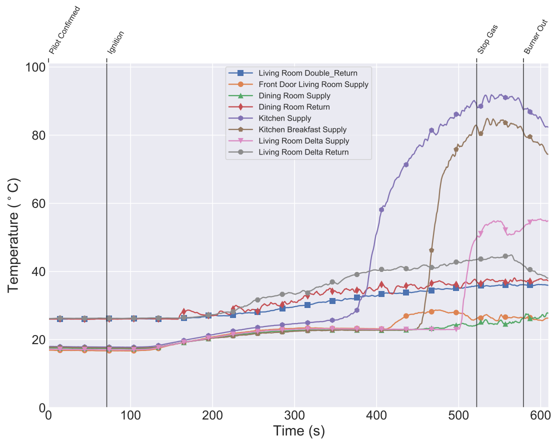

(b) First Floor Vent Temperatures

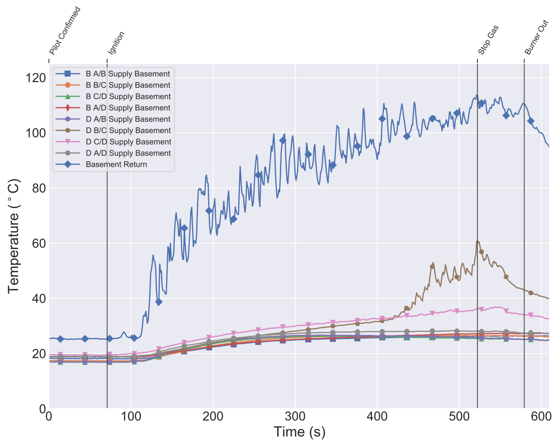

(c) Basement Vent Temperatures

Figure 5.137: Experiment 24 Vent Temperatures

The temperature and velocity within the supply duct are provided in Figure 5.138. Temperatures within the supply duct were held at a significantly cooler temperature for the duration of this experiment. The supply duct velocity indicated an increased negative flow, but it is more probable that the probe just sensed a lower velocity positive flow.

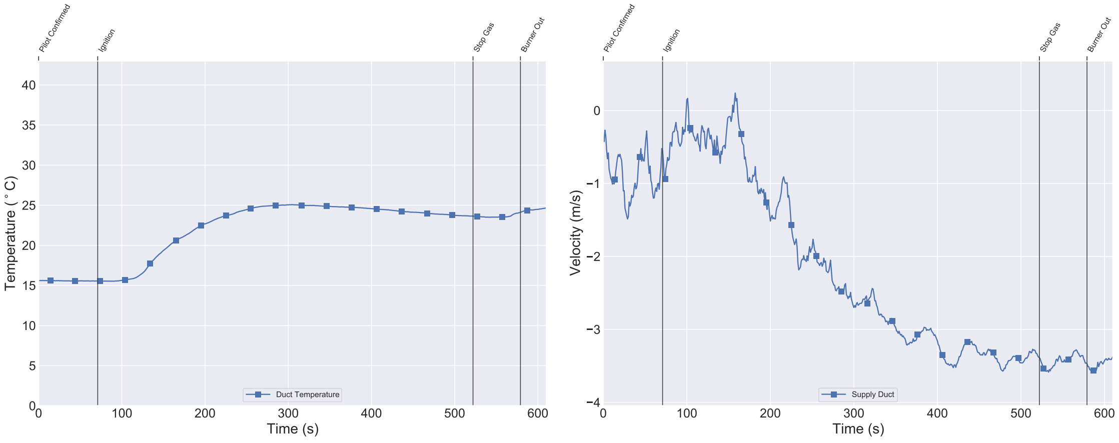

(a) Supply Duct Temperature

(b) Supply Duct Velocity

Figure 5.138: Experiment 24 Supply Duct Temperature and Velocity

5.3.3 Experiment 25

Gas Burner Experiment 25 was performed with the HVAC system off. The flow rate of propane to the gas burner was controlled so that a heat release rate of approximately 302 kW was produced. The stairwell door to the basement and bedroom 1 were closed during this experiment. Experiment 25 was performed as a comparison to experiment 23 to see how smoke and fire gases moved through the structure with the gas burner located in the basement and the basement door closed. The events of interest for Experiment 25 are presented in Table 5.24.

Table 5.24: Experiment 25 Events

Event	Elapsed Time (seconds)
Pilot Confirmed	0
Ignition	97
Stop Gas	998
Burner Out	1048

The time versus temperature graphs for Experiment 25 are provided below. Figure 5.139 displays the basement temperatures and Figure 5.140 displays the first floor temperatures.

Temperatures in the A/B corner of the basement increased approximately 170 °C at the 2.7 m height and increased approximately 50 °C at the 0.3 m height. Temperatures in the B/C corner of the basement increased approximately 130 °C at the 2.7 m height and approximately 60 °C at the 0.3 m height. Temperatures in the C/D corner of the basement increased approximately 300 °C at the 2.7 m height and approximately 75 °C at the 0.3 m height. Temperatures in the A/D corner of the basement increased approximately 225 °C at the 2.7 m height and approximately 50 °C at 0.3 m while the burner was on. Temperatures in the mechanical room increased approximately 35 °C at the 2.7 m height and approximately 10 °C at the 0.3 m height.

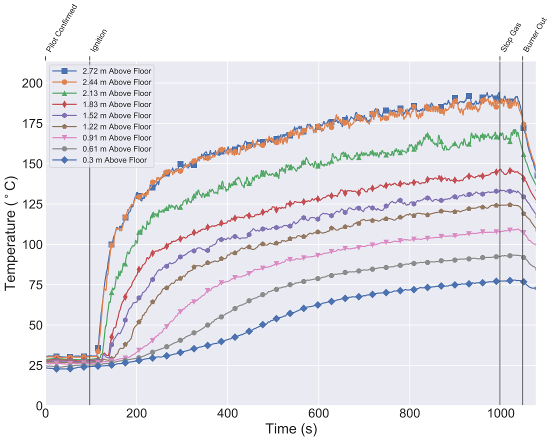

(a) Basement A/B Corner Temperatures

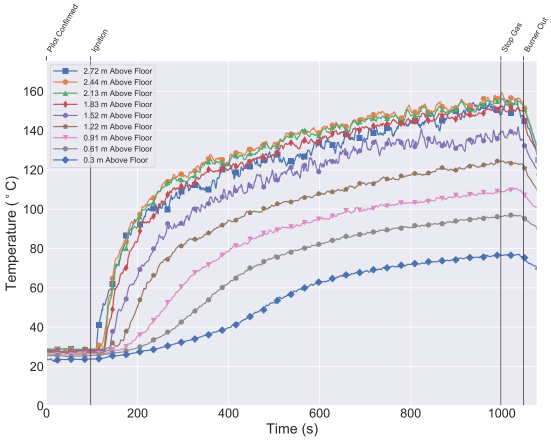

(b) Basement B/C Corner Temperatures

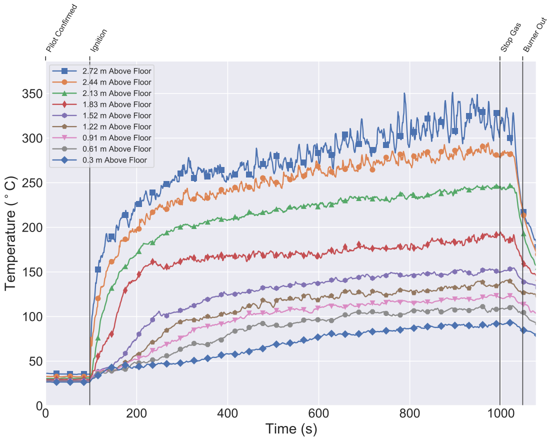

(c) Basement C/D Corner Temperatures

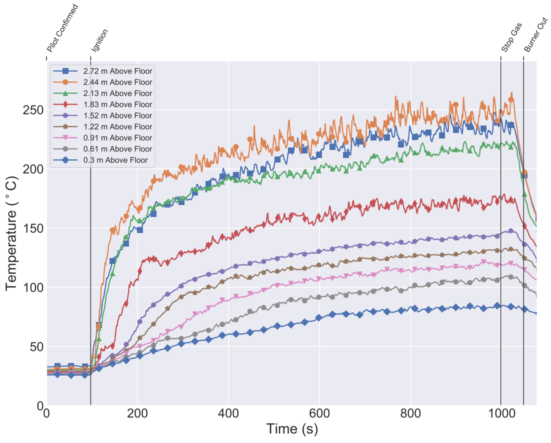

(d) Basement A/D Corner Temperatures

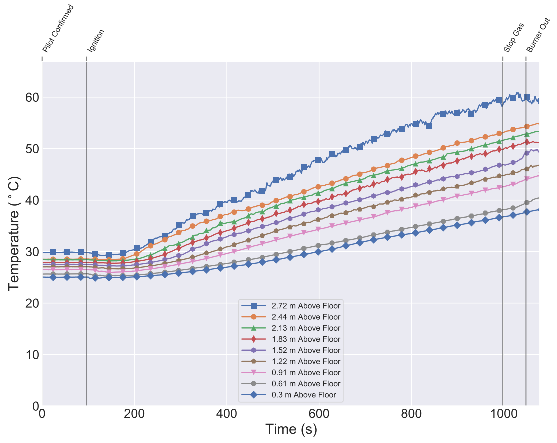

(e) Basement Mechanical Room Temperatures

Figure 5.139: Experiment 25 Basement Temperatures

Temperatures in Bedroom 1 at the 2.4 m height increased approximately 5 °C while the burner was on and temperatures at the 0.3 m height did not appear to change. Temperatures in bedroom 2 at the 2.4 m height increased approximately 10 °C. Temperatures in bedroom 3 increased approximately 13 °C at the 2.4 m height while the burner was on. Temperatures in the living room at the 2.4 m height increased approximately 10 °C while the burner was on. Temperatures in the dining room at the 2.4 m height increased approximately 10 °C while the burner was on. Temperatures in the kitchen at the 2.4 m height increased approximately 15 °C while the burner was on. For all rooms on the first floor, the temperatures at the 0.3 m height increased by less than 5 °C while the gas burner was on. Temperature increases on the first floor appear to follow a similar trend regardless of room location. This could indicate that heat transport through the ductwork spreads evenly across the first floor with little effect from how far a supply vent is from the main supply duct riser.

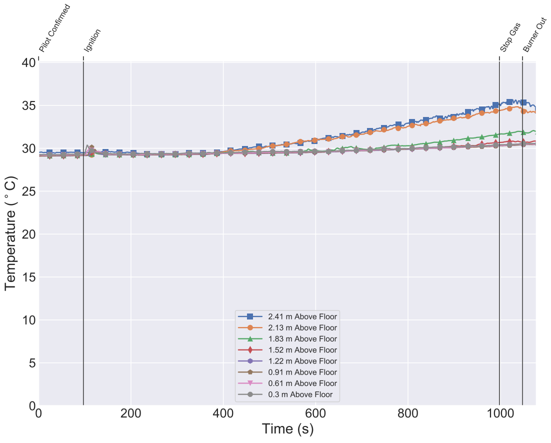

(a) Bedroom 1 Temperatures

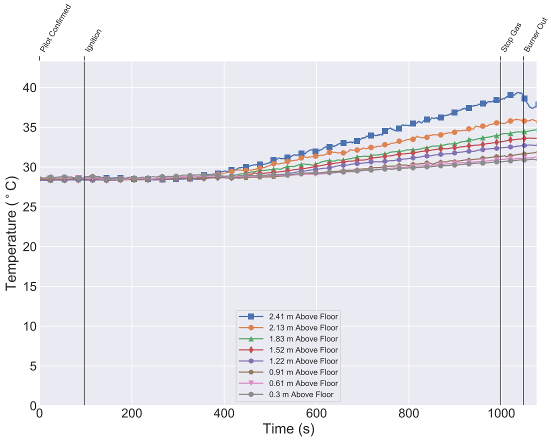

(b) Bedroom 2 Temperatures

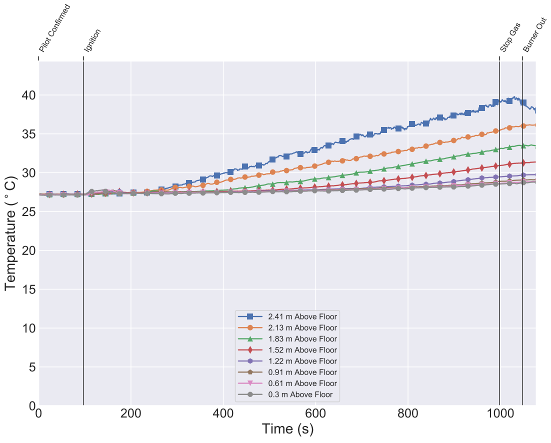

(c) Bedroom 3 Temperatures

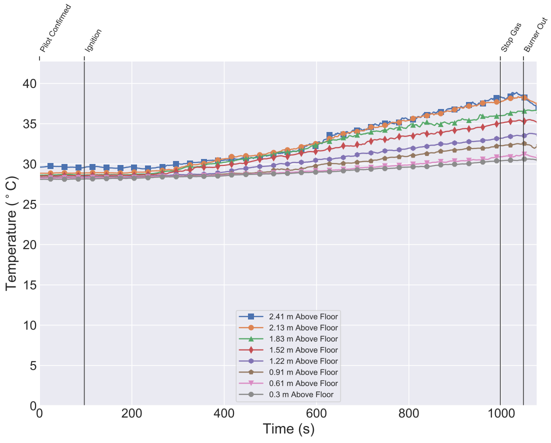

(d) Living Room Temperatures

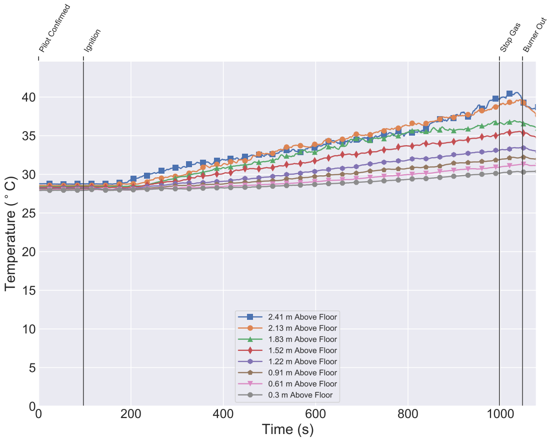

(e) Dining Room Temperatures

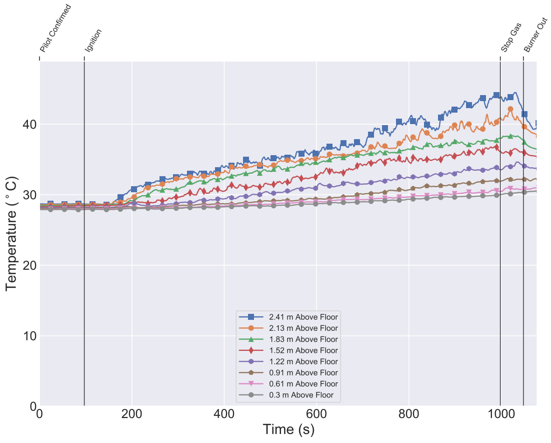

(f) Kitchen Temperatures

Figure 5.140: Experiment 25 First Floor Temperatures

The pressures within each room are shown in Figure 5.141. Pressure in bedroom 1 exceeded the ambient pressure by approximately 1 Pa at all heights while the burner was on with a spike above 25 Pa following ignition. Pressures in bedroom 2 exceeded the ambient pressure by approximately the same amount as bedroom 1 at all heights and spiked to approximately 45 Pa following ignition. Pressures in bedroom 3 were similar at all heights to that of bedroom 2 with a similar spike following ignition. Pressures in the living room and kitchen followed a similar trend to bedroom 2 at all heights with spikes of approximately 55 Pa and 45 Pa, respectively. Pressures in the basement at the 2.13 m height ranged between 10 and 25 Pa above the ambient pressure. Pressures at the 1.2 m ranged between -5 to -20 Pa below the ambient. At the 0.3 m height pressures ranged between -20 to -40 Pa below the ambient.

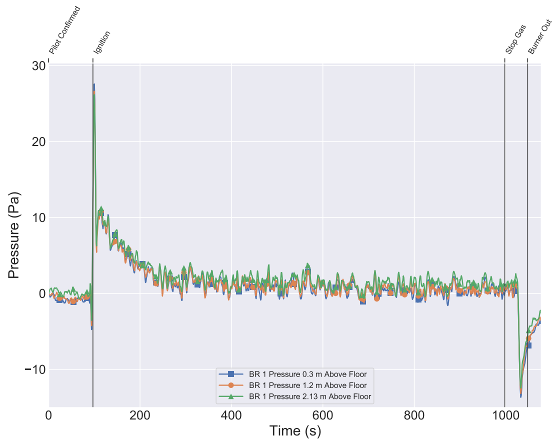

(a) Bedroom 1 Pressures

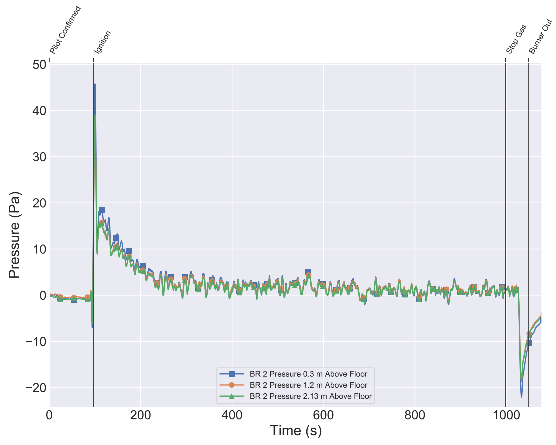

(b) Bedroom 2 Pressures

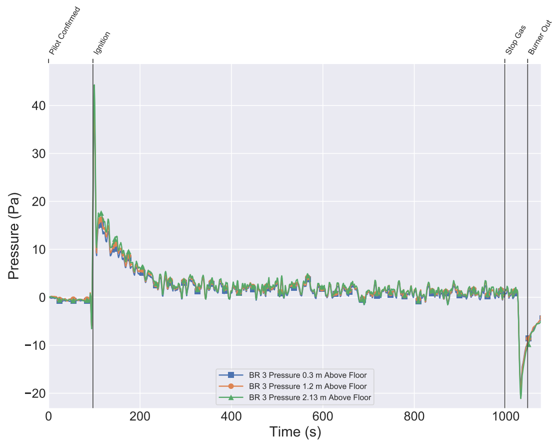

(c) Bedroom 3 Pressures

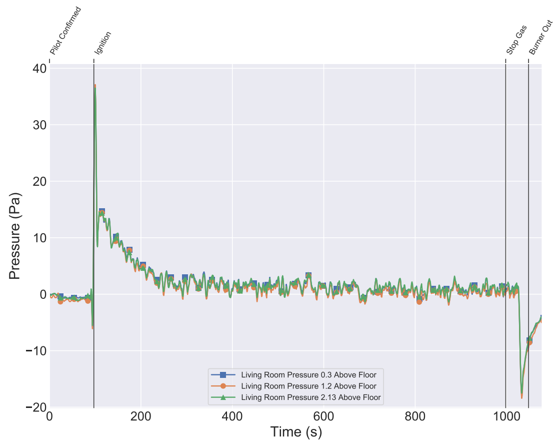

(d) Living Room Pressures

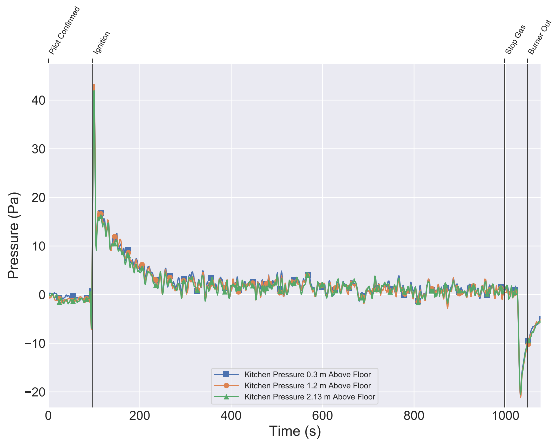

(e) Kitchen Pressures

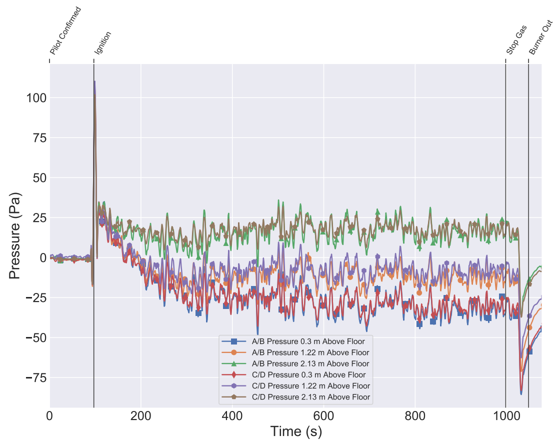

(f) Basement Pressures

Figure 5.141: Experiment 25 Pressures

The gas concentrations of oxygen and carbon dioxide are provided for bedroom 1, bedroom 2, bedroom 3 and the basement in Figure 5.142. Water vapor concentrations are provided for bedroom 2, bedroom 3 and the basement. Oxygen concentrations in bedrooms 1 and 3 appeared to show a slight decrease towards the end of the experiment, but ultimately did not show any significant changes while the gas burner was on. Carbon dioxide levels in bedrooms 1 and 3 remained constant during this experiment. In bedroom 2 oxygen concentrations dropped to approximately 19% while the burner was on. Carbon dioxide levels in bedroom 2 increased approximately 1 to 2% during the experiment. In the basement, oxygen concentrations decreased to approximately 15% prior to the gas burner being turned off. Carbon dioxide concentrations increased by less than 1% during this experiment.

Water vapor concentrations in both bedrooms 2 and 3 appeared to remain constant showing less than a 1% increase during this experiment. Water vapor concentrations in the basement increased by approximately 8% while the gas burner was on. Comparing the water vapor results from this experiment to experiment 23 show that by closing the stairwell door, less water vapor was able to travel through open doorways to the bedroom 2 water vapor measurement laser.

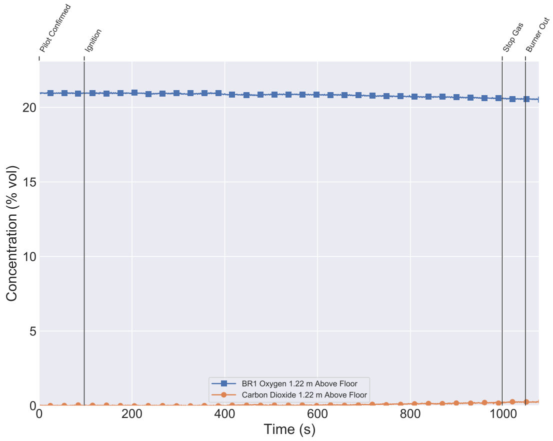

(a) Bedroom 1 Gases

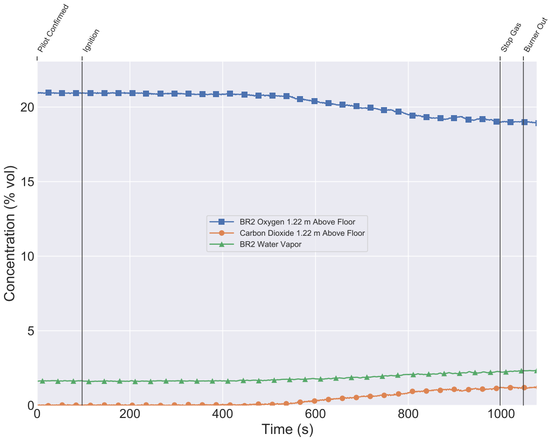

(b) Bedroom 2 Gases

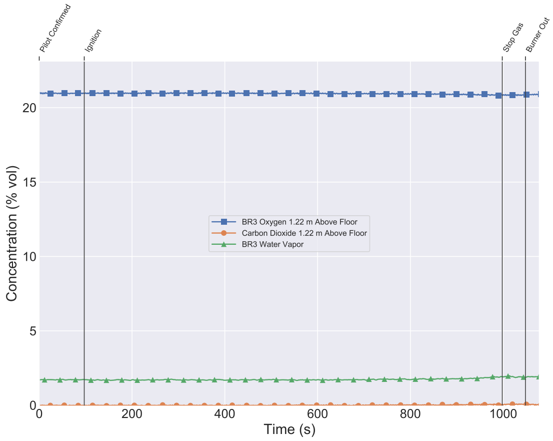

(c) Bedroom 3 Gases

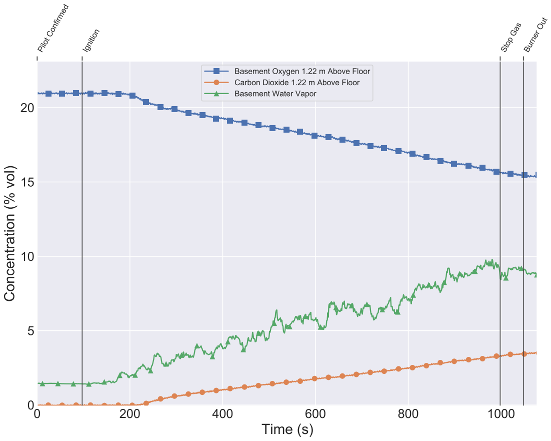

(d) Basement Gases

Figure 5.142: Experiment 25 Bedroom Gases

Figure 5.143 shows the temperatures of the supply and return vents in the structure. Figure 5.143(a) shows the bedroom vent temperatures from this experiment. From the supply vent temperatures recorded in this figure it appears that heat transfer into the bedrooms is occurring through the supply ducts because the supply vent temperatures are consistently greater than the temperatures at the 2.4 m height in those rooms. For all three bedrooms the temperatures of the return

vents remain relatively constant throughout the experiment. The temperature at the bedroom 3 supply vent is consistantly higher than those of bedrooms 2 and 3. This could be due to bedroom 3's proximity to the main supply duct riser.

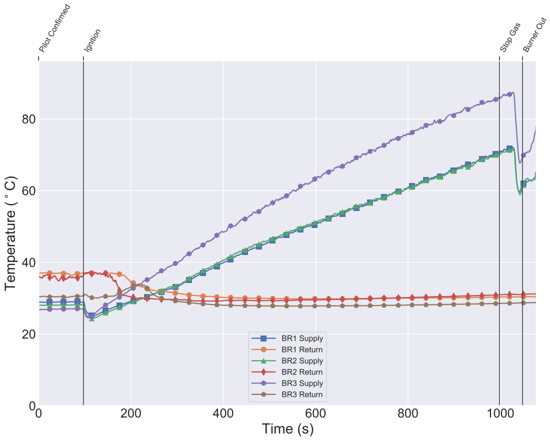

(a) Bedroom Vent Temperatures

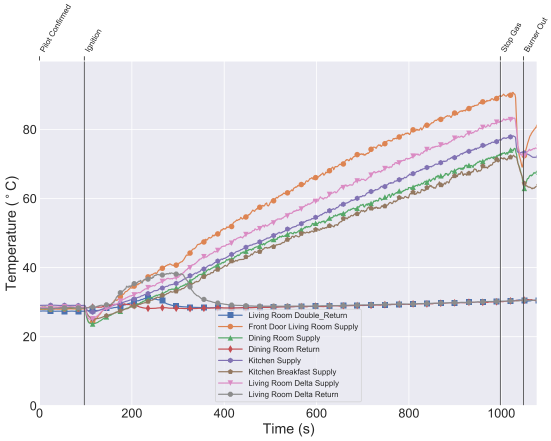

(b) First Floor Vent Temperatures

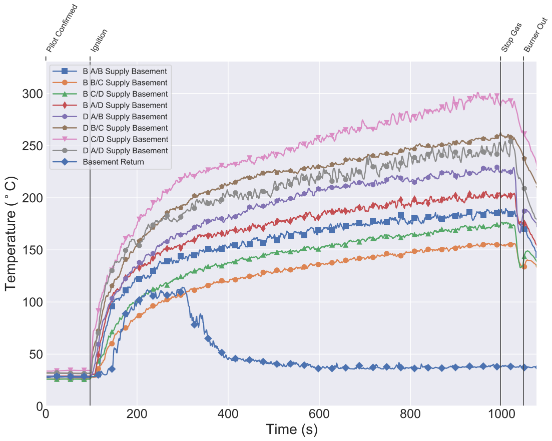

(c) Basement Vent Temperatures

Figure 5.143: Experiment 25 Vent Temperatures

From Figure 5.143(b) it appears that heating of all the other vents on the first floor are due to heat travelling through the HVAC supply system. Similar to the bedrooms, the return vents for the first floor remain relatively constant throughout

the experiment. Figure 5.143(c) shows the temperatures for the vents located in the basement. The highest temperature for a supply vent in the basement was approximately 300 °C recorded at supply vent S-07 in the C/D corner of the D side of the basement. Temperatures for all vents in the basement followed the trends of the thermocouple trees located in their respective areas. With the exception of the return vent in the basement, all vent temperatures trended upwards as expected. The return vent in the basement initially followed an increasing temperature trend similar to the supply vents but then around the 300 second mark the temperature at the return vent decreased by approximately 70 °C to a final constant temperature of approximately 40 °C. One possible cause of this behavior is that for the first 200 seconds following ignition the return vent was in a turbulent region of gas mixing. It is possible that once the HGL was established the return vent ended up below the height of that layer.

The temperature and velocity within the supply duct are provided in Figure 5.144. From this figure it can be seen that there is a temperature increase within the supply duct to approximately 120 °C while the gas burner was on. The increase in temperature is consistent with what was observed from the supply vent temperatures on the first floor. All first floor vents could be receiving heat through the HVAC supply duct network and then distribute that heat throughout the first floor. The supply ducts appear to be the means of heating for bedroom 3 behind the closed door. The supply vent velocity appears to show that there is an increasing flow of air in the supply vent from the basement to the first floor while the gas burner is on. This velocity decreases after the gas burner is shut off, thus supporting

an increased flow to the first floor driven by the fire.

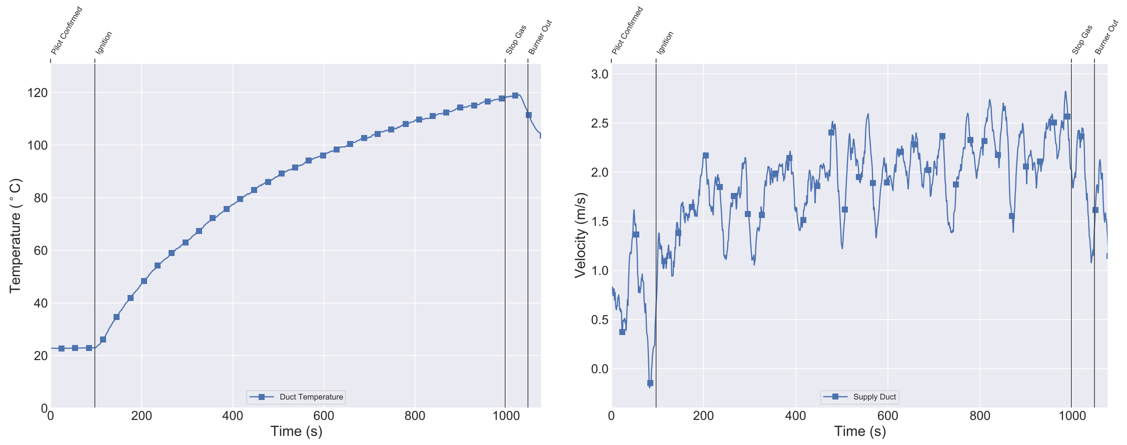

(a) Supply Duct Temperature (b) Supply Duct Velocity

Figure 5.144: Experiment 25 Supply Duct Temperature and Velocity

5.3.4 Experiment 26

Gas Burner Experiment 26 was performed with the HVAC system set to A/C. The flow rate of propane to the gas burner was controlled so that a heat release rate of approximately 302 kW was produced. The stairwell door to the basement was closed during this experiment. The doorway to bedroom 1 and all exterior vents were closed for this experiment. Experiment 26 was performed to copmare how smoke and fire gases moved through the structure via the HVAC system with the system set to A/C and the stairwell door closed. Comparisons will be made to Experiment 24 and 25. The events of interest for Experiment 26 are presented in Table 5.25.

Table 5.25: Experiment 26 Events

Event	Elapsed Time (seconds)
Pilot Confirmed	0
Ignition	96
Stop Gas	578
Burner Out	629

The time versus temperature graphs for Experiment 26 are provided below. Figure 5.145 displays the basement temperatures and Figure 5.146 displays the first floor temperatures.

Temperatures in the basement do not show any variations in temperature when compared to Experiments 24 and 25.

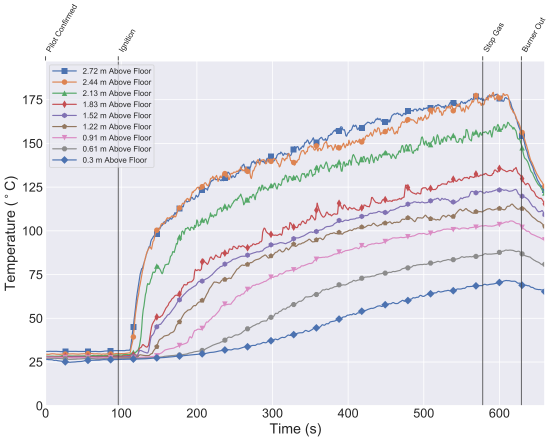

(a) Basement A/B Corner Temperatures

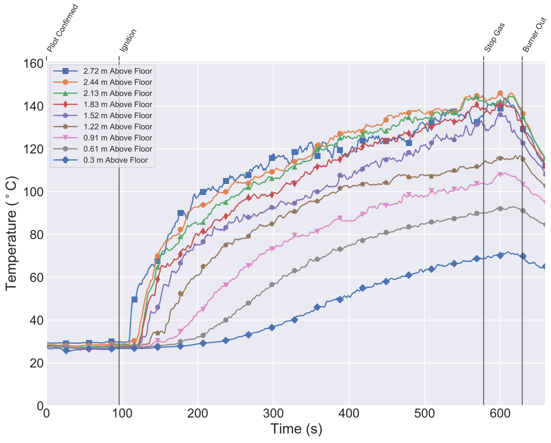

(b) Basement B/C Corner Temperatures

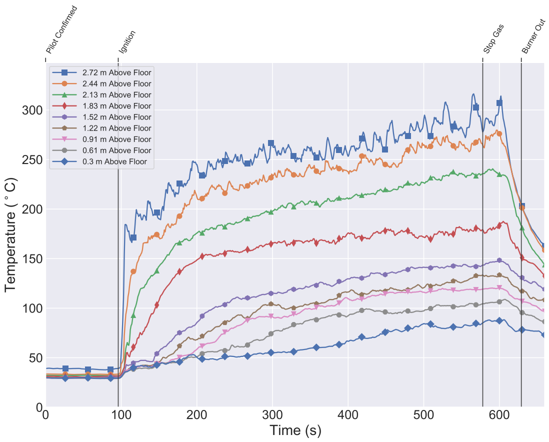

(c) Basement C/D Corner Temperatures

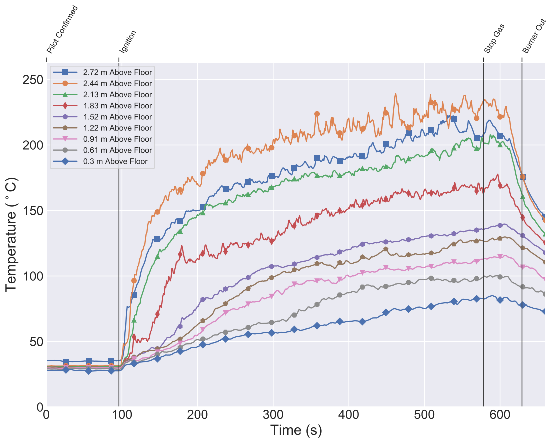

(d) Basement A/D Corner Temperatures

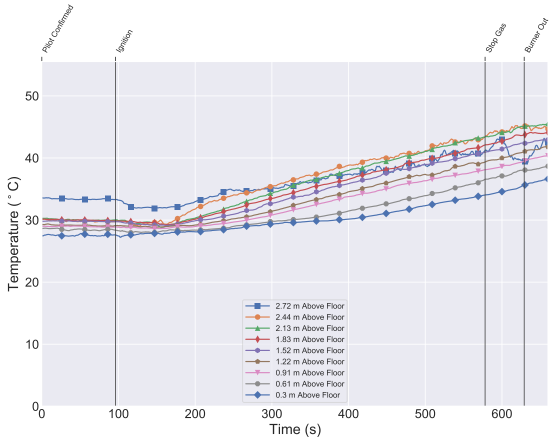

(e) Basement Mechanical Room Temperatures

Figure 5.145: Experiment 26 Basement Temperatures

Comparison of first floor temperatures between Experiment 24 and this experiment show that the closed door to the basement limited the spread of heat to the first floor. Comparison to Experiment 25 shows that the bedroom temperatures remained at a relatively constant temperature for the duration of this experiment. Temperatures in the living room, dining room and kitchen showed temperature increases proportional to their proximity to the basement staircase. The greater temperature increase closer to the staircase indicates that there is still some heat leakage around the basement staircase door. If the driving heat transfer occurred through the HVAC system, the greatest heat increase would be expected in the living room because that location has the shortest travel distance in the HVAC ductwork.

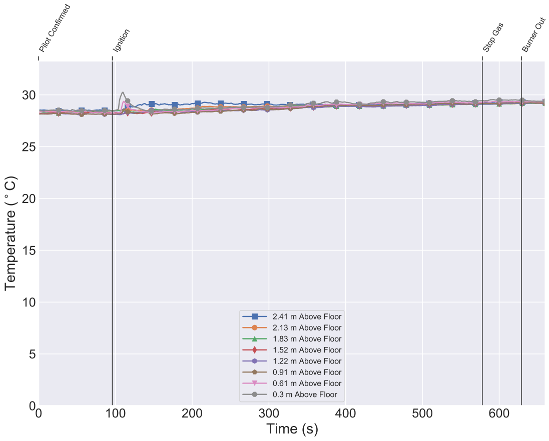

(a) Bedroom 1 Temperatures

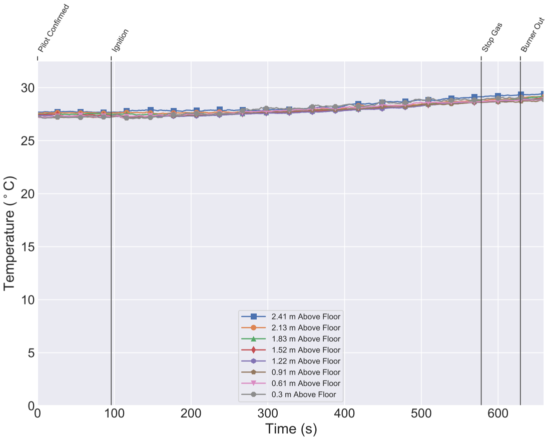

(b) Bedroom 2 Temperatures

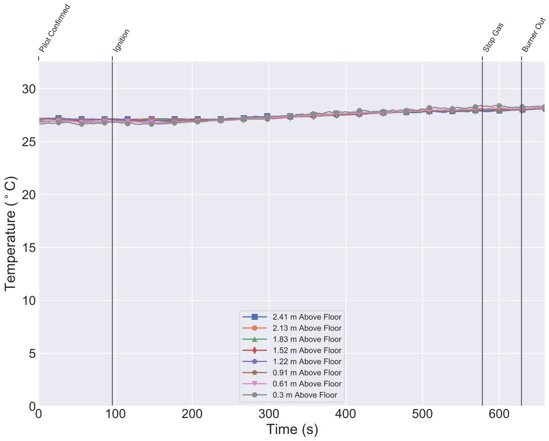

(c) Bedroom 3 Temperatures

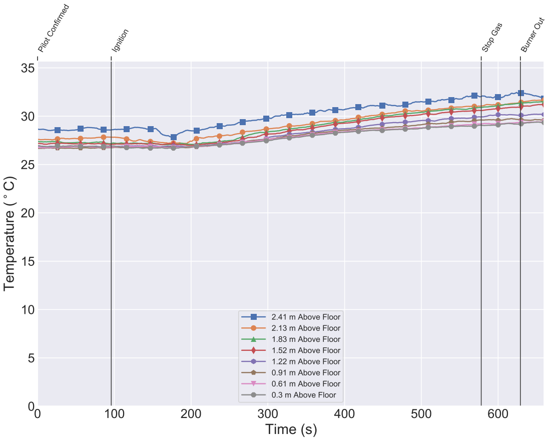

(d) Living Room Temperatures

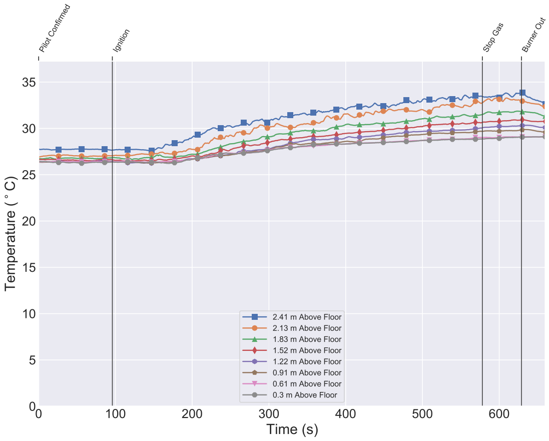

(e) Dining Room Temperatures

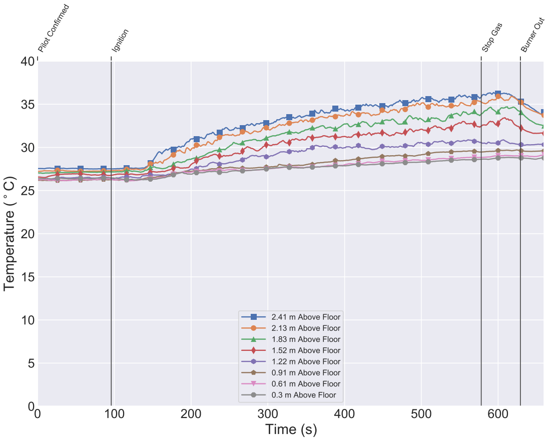

(f) Kitchen Temperatures

Figure 5.146: Experiment 26 First Floor Temperatures

The pressures within each room are shown in Figure 5.147. Pressures in this experiment are similar to those observed in Experiment 25. Post ignition spike values exceed those observed in Experiment 24 by twice the amount or greater, however, the steady pressure values recorded after that trend similar to both Experiments 24 and 25.

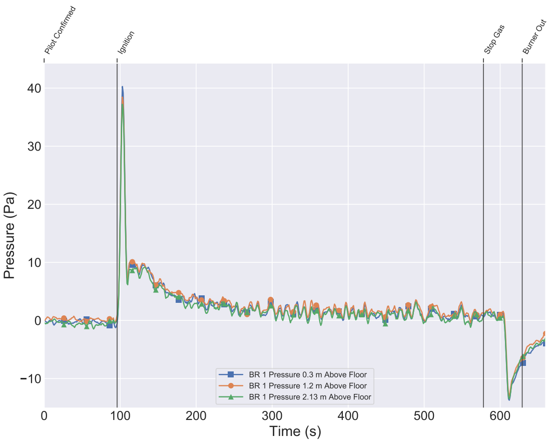

(a) Bedroom 1 Pressures

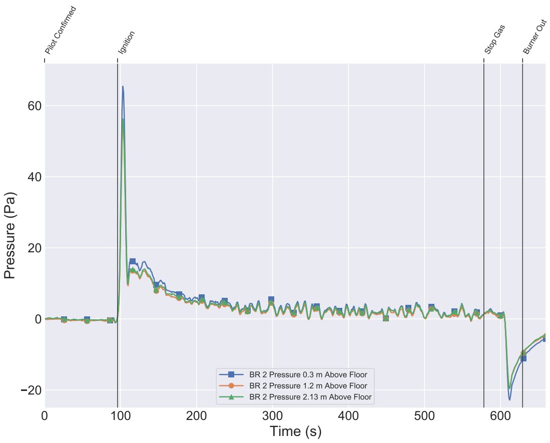

(b) Bedroom 2 Pressures

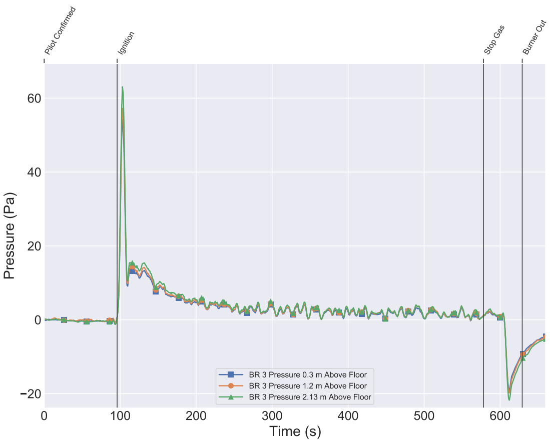

(c) Bedroom 3 Pressures

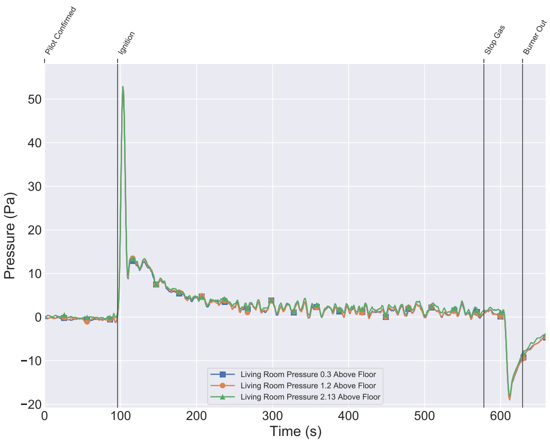

(d) Living Room Pressures

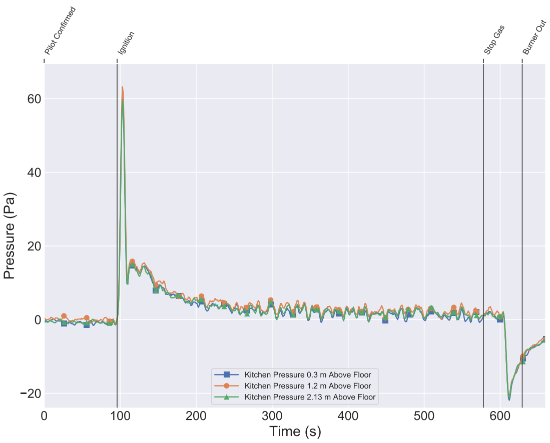

(e) Kitchen Pressures

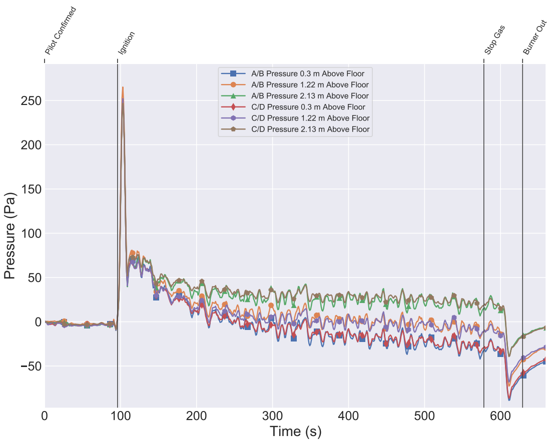

(f) Basement Pressures

Figure 5.147: Experiment 26 Pressures

The gas concentrations of oxygen and carbon dioxide are provided for bedroom 1, bedroom 2, bedroom 3 and the basement in Figure 5.148. Water vapor concentrations are provided for bedroom 2, bedroom 3 and the basement. Oxygen concentrations in all three bedrooms showed minimal change during this experiment. For the same duration of time in Experiment 25, this behavior is similar, including the gas and water vapor concentrations in the basement. Bedroom 2 gas and water vapor concentrations differ between Experiment 24 and this experiment. With the staircase door open in Experiment 24, more fire gases were able to extend to bedroom 2.

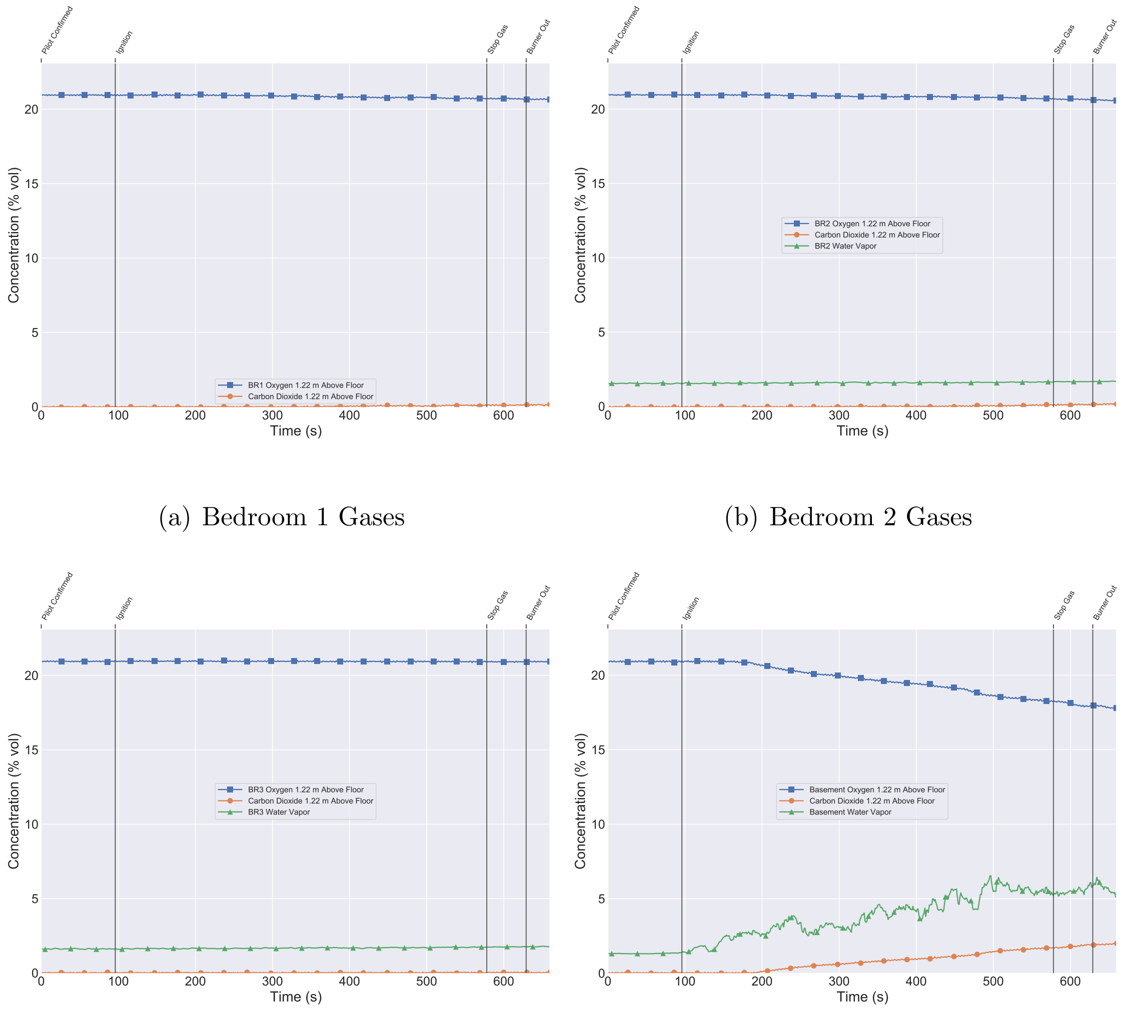

(a) Bedroom 1 Gases

(b) Bedroom 2 Gases

(c) Bedroom 3 Gases

(d) Basement Gases

Figure 5.148: Experiment 26 Bedroom Gases

Figure 5.149 shows the temperatures of the supply and return vents in the structure. From Figure 5.149(a) it can be seen that immediately following ignition, there was a spike in temperature at the return vents bedrooms 1 and 2. Shortly after that there is a small increase in the supply vent temperatures. The distinct increase in return vent temperatures was not observed in either Experiment 24 or Experiment 25. The sharp increase was follwed by a slow and steady decrease as the

temperatures in those bedrooms trended towards those of the supply vents. From this chart it can be seen that the ignition caused a surge of reverse air movement through the HVAC return system from the basement up to the first floor. Closing the staircase door and having positive pressure in the HVAC supply system caused the return system to be the path of least resistance for the pressure buildup that followed ignition. In contrast to Experiment 25, the supply vent temperatures in the bedrooms did not increase during the experiment because the HVAC supply system was able to condition the air being distributed on the first floor.

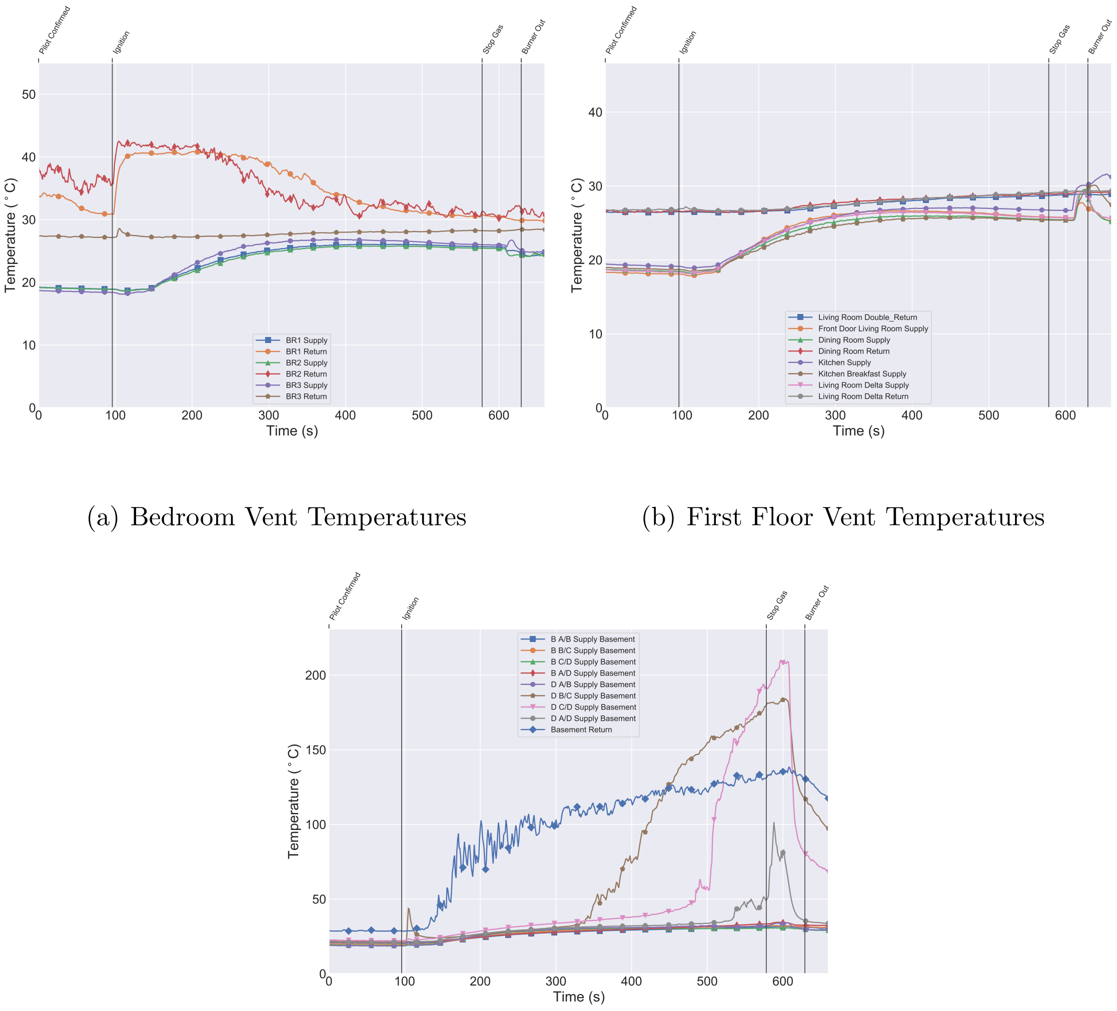

(a) Bedroom Vent Temperatures

(b) First Floor Vent Temperatures

(c) Basement Vent Temperatures

Figure 5.149: Experiment 26 Vent Temperatures

From Figure 5.149(b) it can be seen that the remaining first floor supply vents experienced slight heating following ignition but were able to maintain cool temperatures throughout the experiment. Both Experiments 24 and 25 showed some heating of their supply vent temperatures which did not occur in this experiment.

Figure 5.149(c) shows the temperatures for the vents located in the basement. The basement vent temperatures were similar to those observed in experiment 24

with the exception of the delta side supply vents. By closing the staircase door, the supply vents on the delta side were unable to maintain a higher pressure than basement, thus resulting in increased supply vent temperatures.

The temperature and velocity within the supply duct are provided in Figure 5.150. Similar to Experiment 24, the temperature in the supply duct and the velocity followed the same trends.

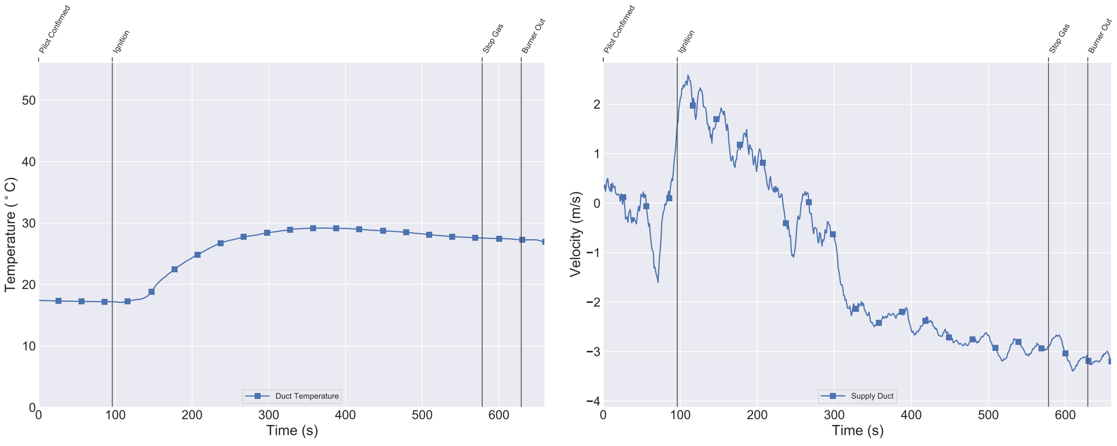

(a) Supply Duct Temperature (b) Supply Duct Velocity

Figure 5.150: Experiment 26 Supply Duct Temperature and Velocity

5.3.5 Experiment 27

Gas Burner Experiment 27 was performed with the HVAC system set to heat. The flow rate of propane to the gas burner was controlled so that a heat release rate of approximately 302 kW was produced. The stairwell door to the basement was closed during this experiment. The doorway to bedroom 1 and all exterior vents were closed for this experiment. Experiment 27 was performed to see how smoke and fire gases moved through the structure via the HVAC system with the system

set to heat. Comparisons were made to Experiments 25 and 26. The events of interest for Experiment 27 are presented in Table 5.26.

Table 5.26: Experiment 27 Events

Event	Elapsed Time (seconds)
Pilot Confirmed	0
Ignition	89
Stop Gas	629
Burner Out	689

The time versus temperature graphs for Experiment 27 are provided below. Figure 5.151 displays the basement temperatures and Figure 5.152 displays the first floor temperatures.

Temperatures in the basement did not reach the same peak temperatures observed in Experiments 25 and 26 but followed similar increasing trends.

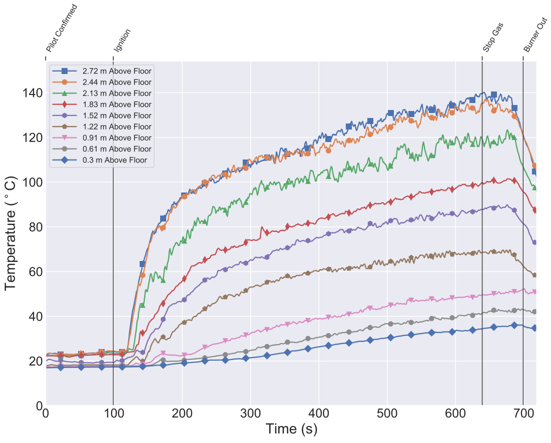

(a) Basement A/B Corner Temperatures

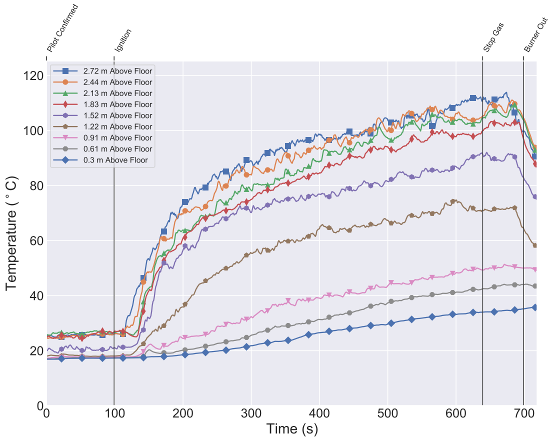

(b) Basement B/C Corner Temperatures

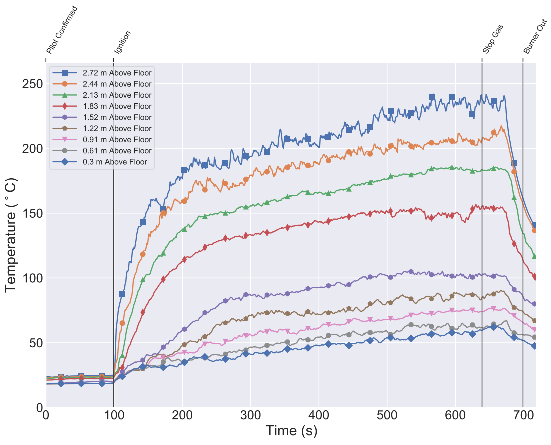

(c) Basement C/D Corner Temperatures

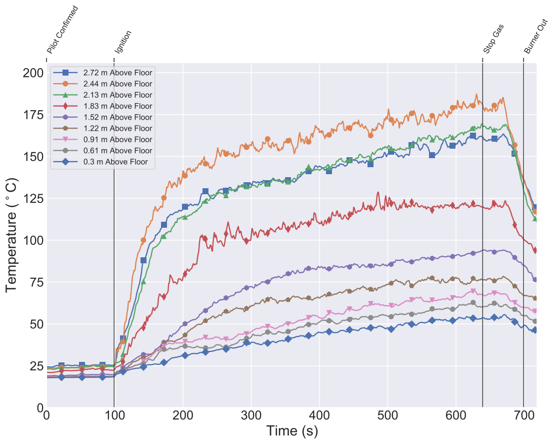

(d) Basement A/D Corner Temperatures

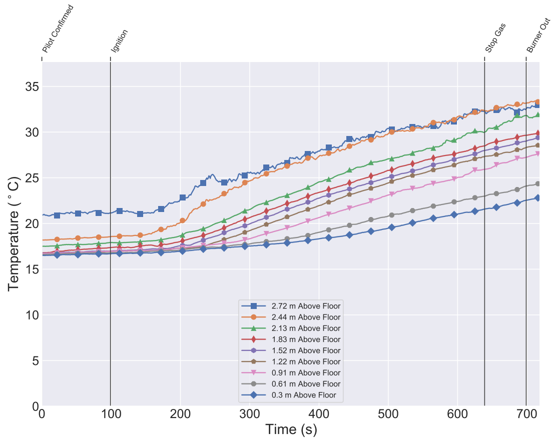

(e) Basement Mechanical Room Temperatures

Figure 5.151: Experiment 27 Basement Temperatures

Temperatures on the first floor in the bedrooms followed increasing trends observed in Experiment 25. Temperatures outside the bedrooms on the first floor exceeded Experiments 25 and 26 in increasing rate and temperatures.

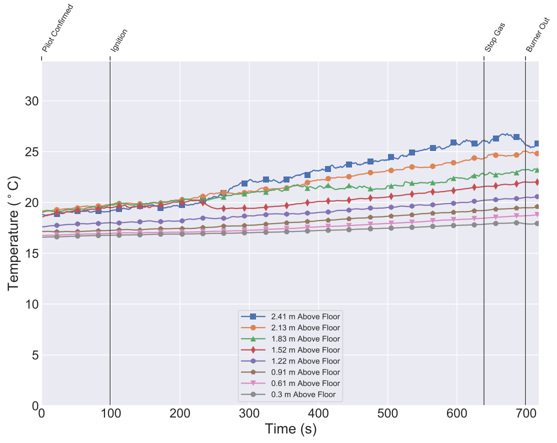

(a) Bedroom 1 Temperatures

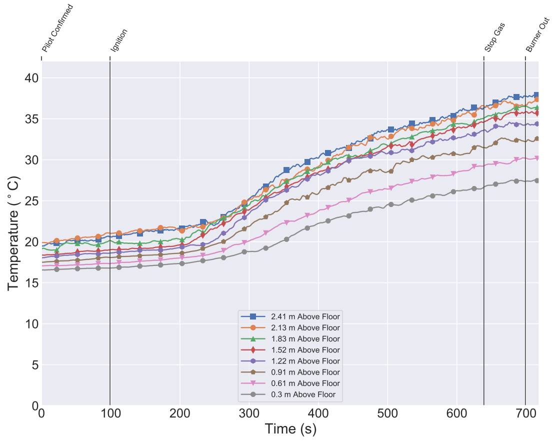

(b) Bedroom 2 Temperatures

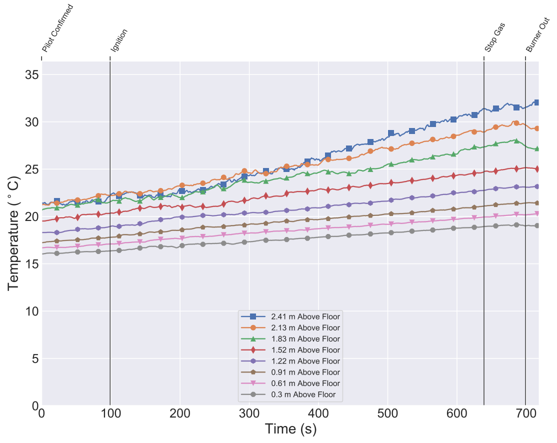

(c) Bedroom 3 Temperatures

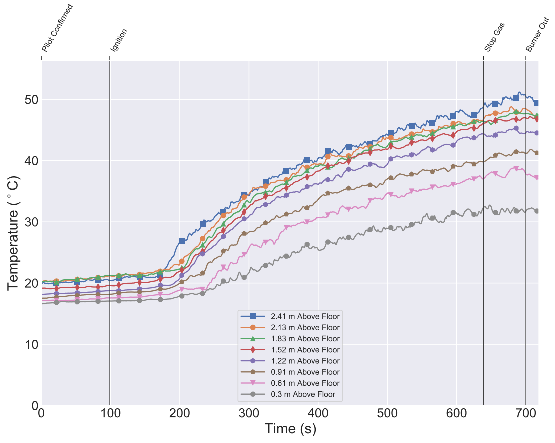

(d) Living Room Temperatures

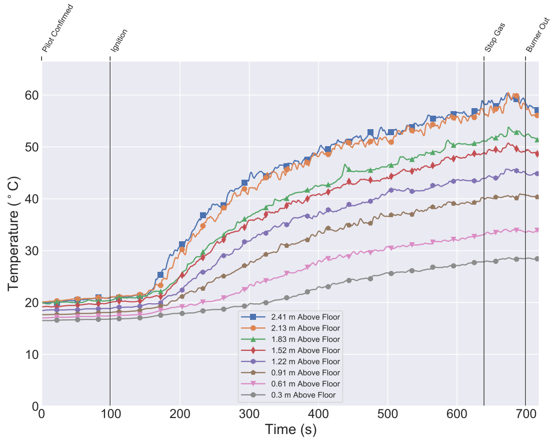

(e) Dining Room Temperatures

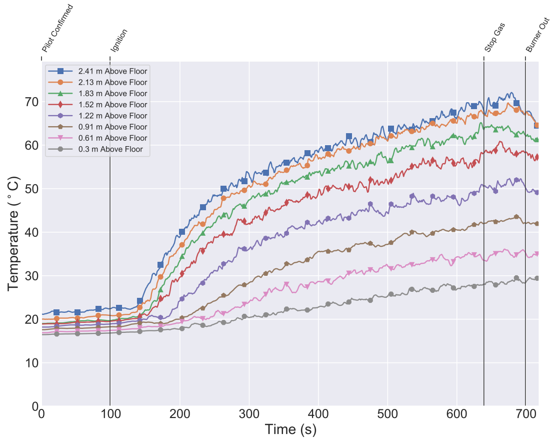

(f) Kitchen Temperatures

Figure 5.152: Experiment 27 First Floor Temperatures

The pressures within each room are shown in Figure 5.153. Steady pressures in this experiment exceeded that of Experiment 25 by a few pascals in all locations except the basement. Pressure spikes following ignition were less than those of Experiment 26 by almost half in all locations. Similar to Experiment 25, the steady pressures in this experiment exceeded those of Experiment 26 by a few pascals in all locations.

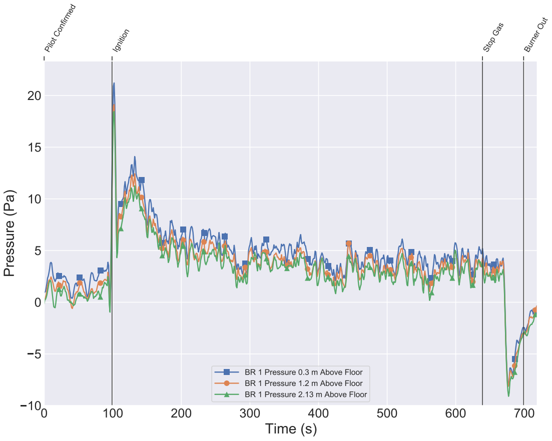

(a) Bedroom 1 Pressures

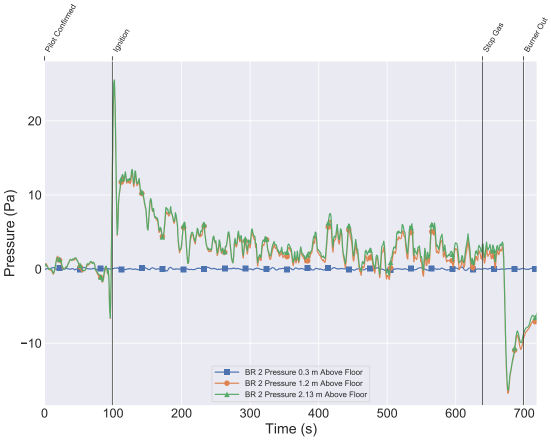

(b) Bedroom 2 Pressures

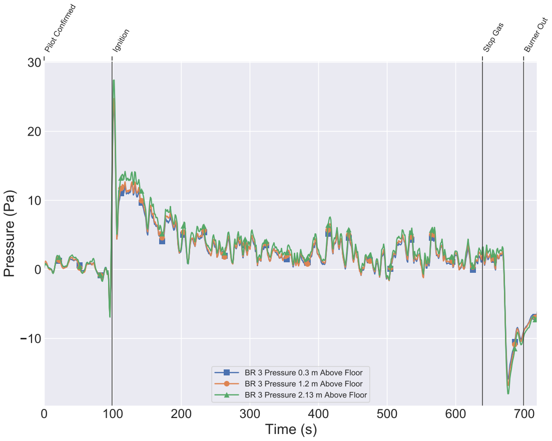

(c) Bedroom 3 Pressures

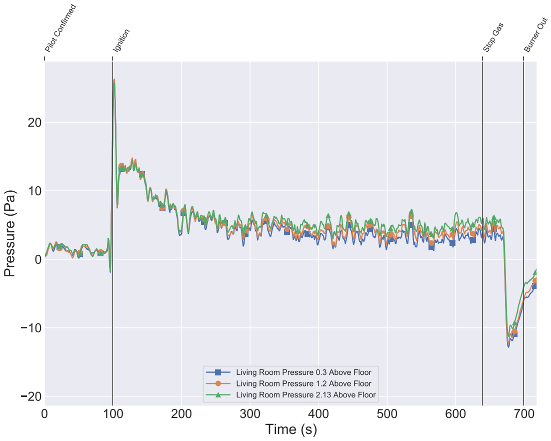

(d) Living Room Pressures

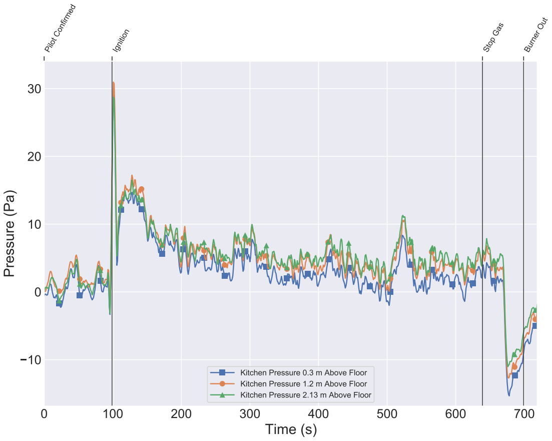

(e) Kitchen Pressures

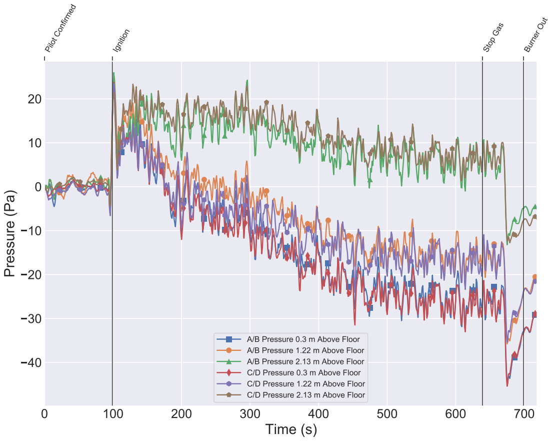

(f) Basement Pressures

Figure 5.153: Experiment 27 Pressures

The gas concentrations of oxygen and carbon dioxide are provided for bedroom 1, bedroom 2, bedroom 3 and the basement in Figure 5.154. Gas concentrations in the bedrooms followed a similar trend between this experiment and Experiments 25 and 26. Basement gas concentrations changed by a smaller percentage during this experiment than the others. Oxygen concentrations dropped by only 1% to 2% in this experiment. Similarly, the Carbon Monoxide levels only increased by approximately 1% during this experiment.

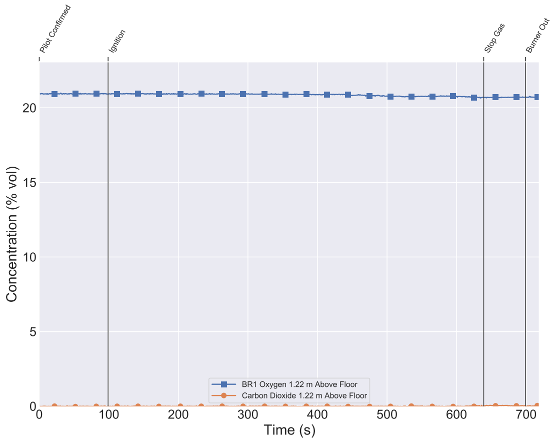

(a) Bedroom 1 Gases

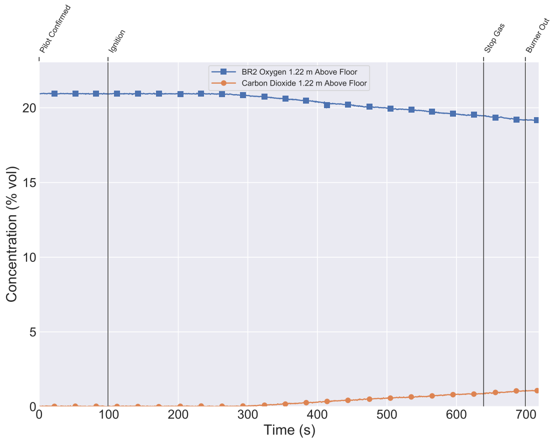

(b) Bedroom 2 Gases

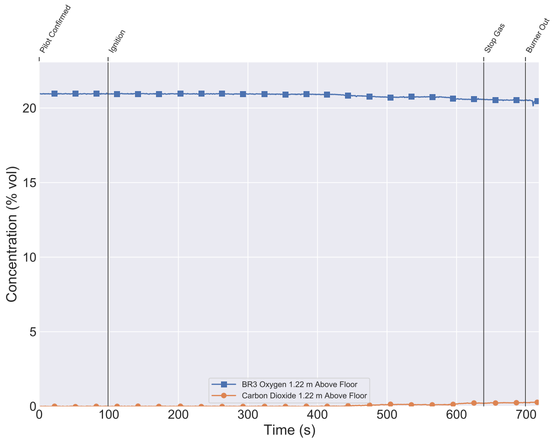

(c) Bedroom 3 Gases

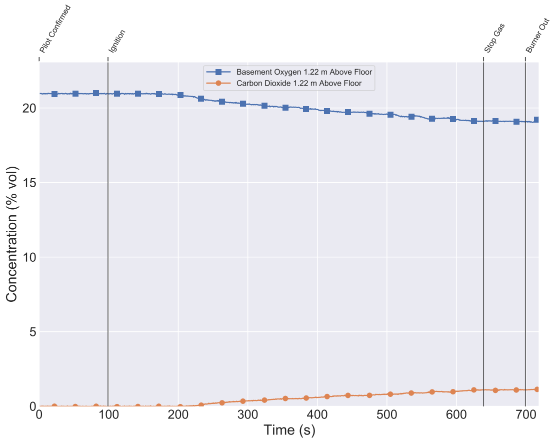

(d) Basement Gases

Figure 5.154: Experiment 27 Bedroom Gases

Figure 5.155 shows the temperatures of the supply and return vents in the structure. Bedroom supply vents experienced a small temperature increase during the experiment but remained at relatively stable temperatures. Bedroom return vents trended with the room temperatures at their respective heights. First floor supply and return vents behaved in a similar manner to those in the bedroom. Basement supply vents maintained relatively constant temperatures for the majority of the experiment. Supply vents on the same side as the gas burner maintained a constant temperature for several hundred seconds before showing sharp increases, similar to Experiment 26. The return vent in the basement behaved in a similar manner to that of Experiment 26.

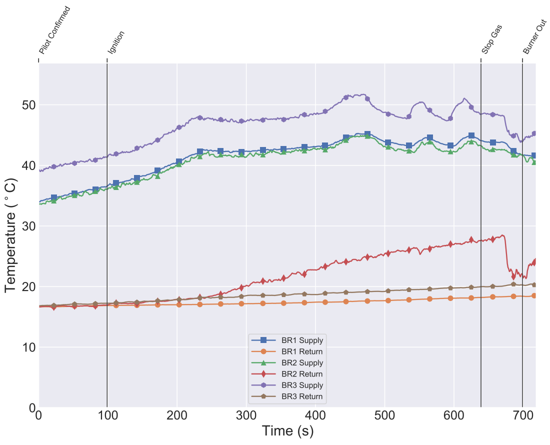

(a) Bedroom Vent Temperatures

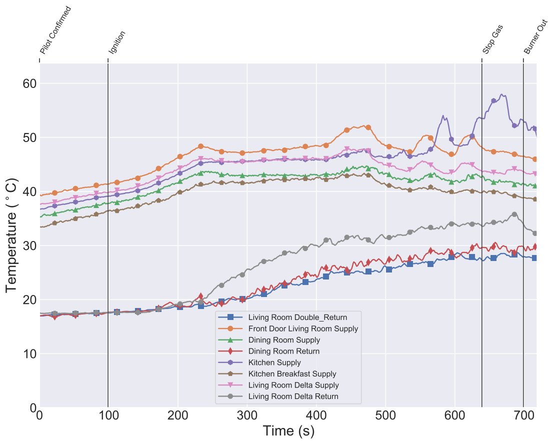

(b) First Floor Vent Temperatures

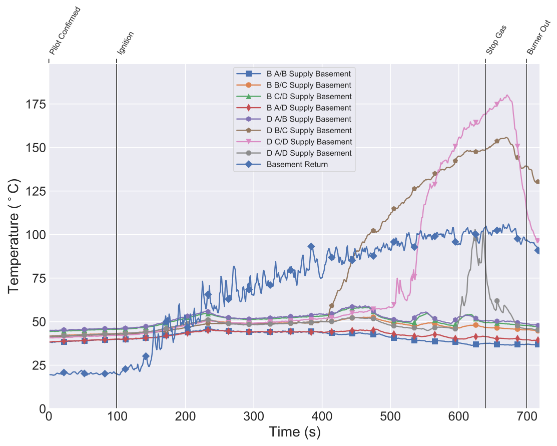

(c) Basement Vent Temperatures

Figure 5.155: Experiment 27 Vent Temperatures

The temperature and velocity within the supply duct are provided in Figure 5.156. Temperatures in the supply duct showed only a small increase in temperature during the experiment. Conditioning of the air appeared to continue throughout the experiment because the increase in temperature is far less in slope and value than that of Experiment 25. The velocity in the supply duct behaved similarly to the trend identified in Experiment 26.

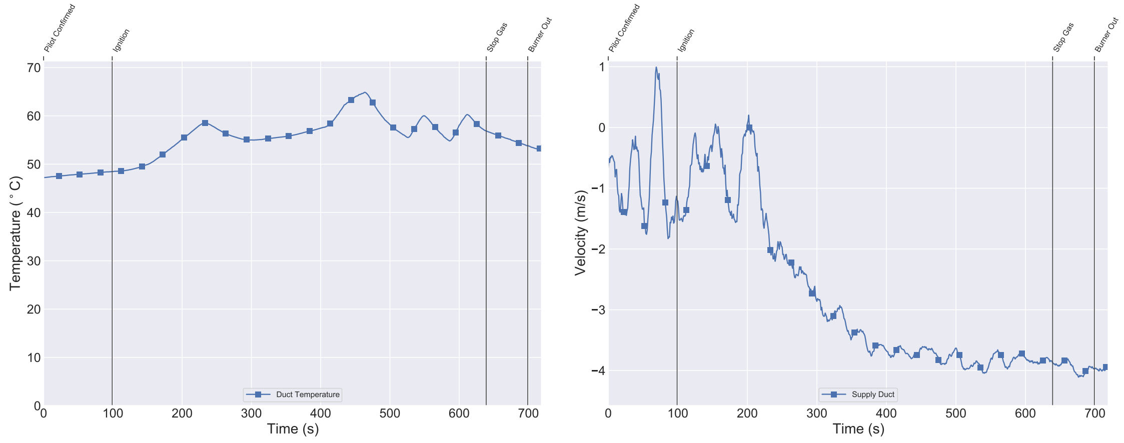

(a) Supply Duct Temperature

(b) Supply Duct Velocity

Figure 5.156: Experiment 27 Supply Duct Temperature and Velocity

5.3.6 Experiment 28

Gas Burner Experiment 28 was performed with the HVAC system off. The flow rate of propane to the gas burner was controlled so that a heat release rate of approximately 393 kW was produced. The stairwell door to the basement was closed during this experiment. The doorway to bedroom 1 and all exterior vents were closed for this experiment. Experiment 28 was performed to see how smoke and fire gases moved through the structure via the HVAC system with a higher HRR. Comparisons were made to Experiment 25. The events of interest for Experiment 28 are presented in Table 5.27.

Table 5.27: Experiment 28 Events

Event	Elapsed Time (seconds)
Pilot Confirmed	0
Ignition	120
Stop Gas	1012
Burner Out	1064

The time versus temperature graphs for Experiment 28 are provided below. Figure 5.157 displays the basement temperatures and Figure 5.158 displays the first floor temperatures.

Temperatures in the basement displayed similar increase trends but with greater values than those of Experiment 25. This trend is similar to other experiments where an increased HRR was the only variation to the experiment.

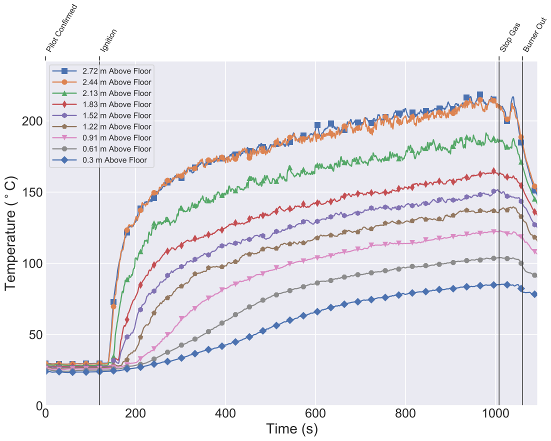

(a) Basement A/B Corner Temperatures

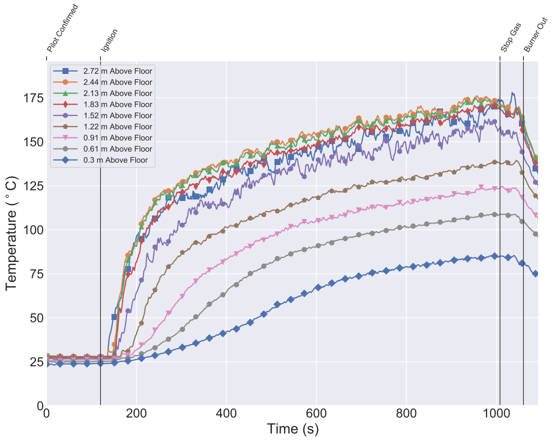

(b) Basement B/C Corner Temperatures

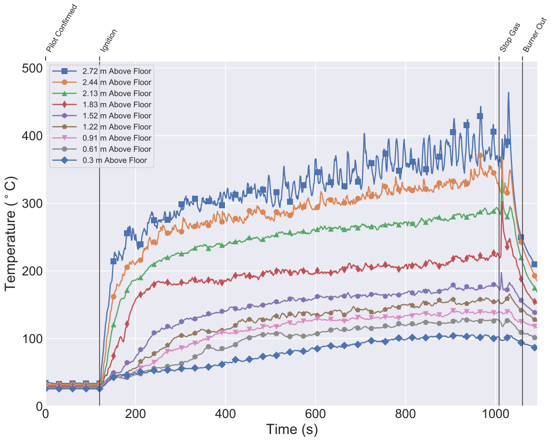

(c) Basement C/D Corner Temperatures

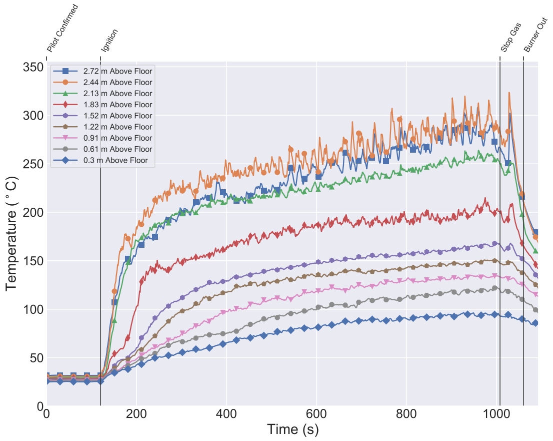

(d) Basement A/D Corner Temperatures

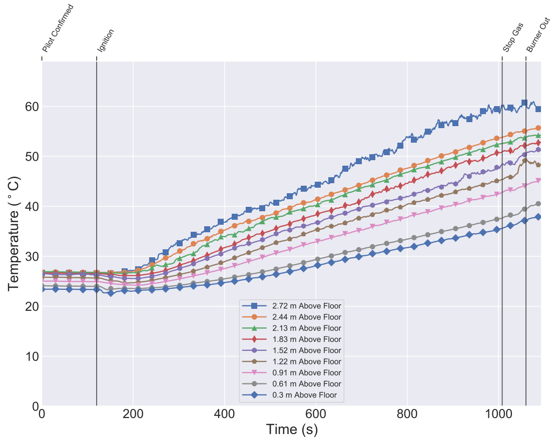

(e) Basement Mechanical Room Temperatures

Figure 5.157: Experiment 28 Basement Temperatures

Temperatures on the first floor behaved similar in trends and values to those reported in Experiment 25.

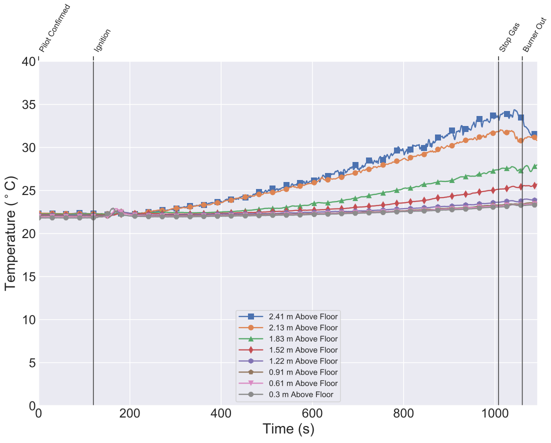

(a) Bedroom 1 Temperatures

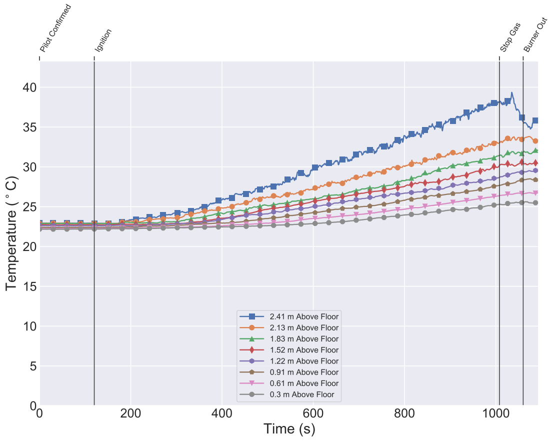

(b) Bedroom 2 Temperatures

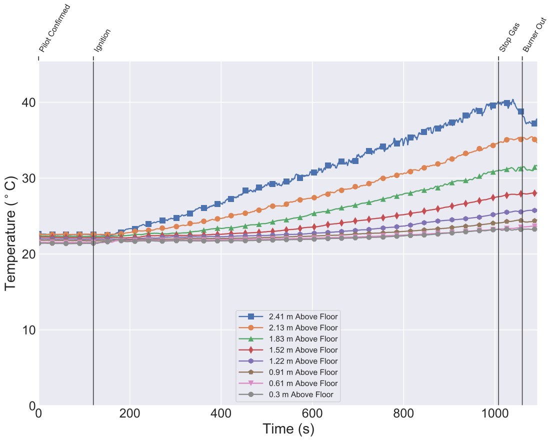

(c) Bedroom 3 Temperatures

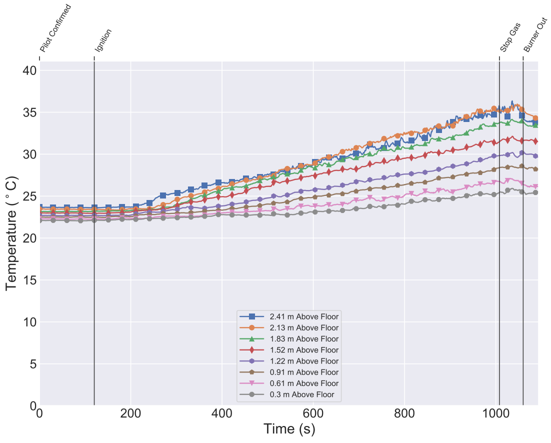

(d) Living Room Temperatures

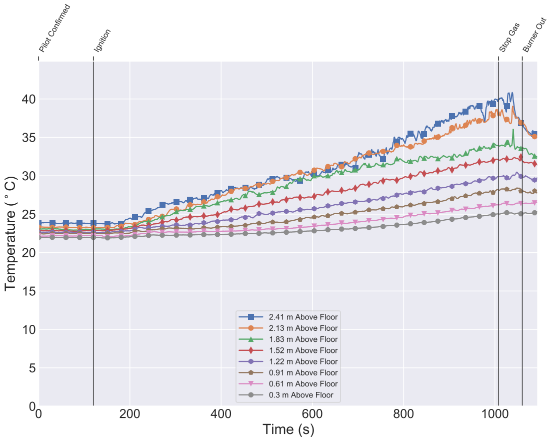

(e) Dining Room Temperatures

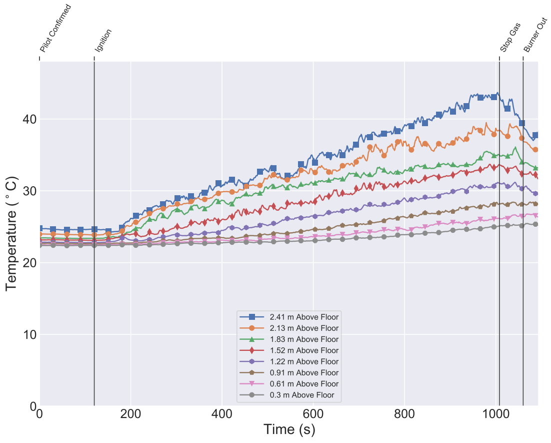

(f) Kitchen Temperatures

Figure 5.158: Experiment 28 First Floor Temperatures

The pressures within each room are shown in Figure 5.159. Pressures throughout the structure were higher than those of Experiment 25 and less steady in several locations.

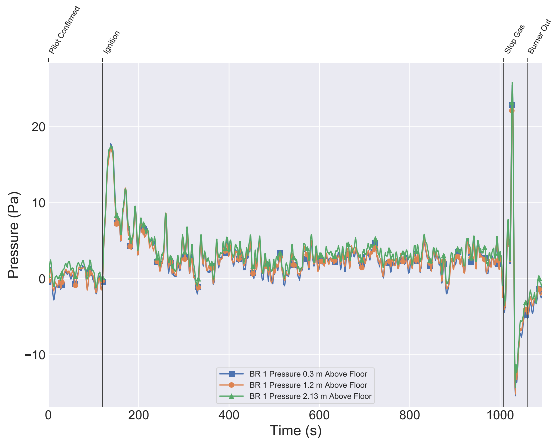

(a) Bedroom 1 Pressures

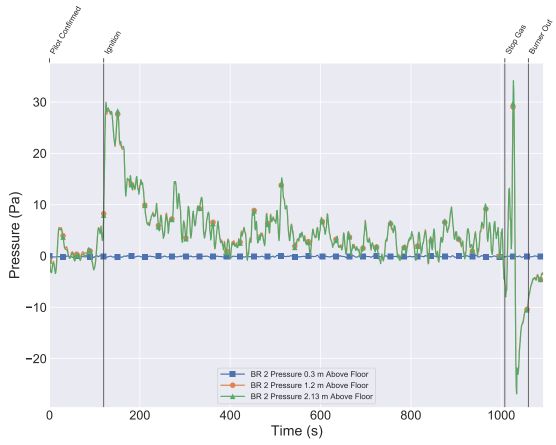

(b) Bedroom 2 Pressures

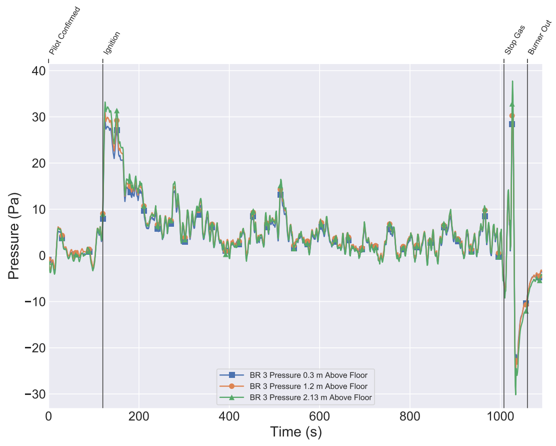

(c) Bedroom 3 Pressures

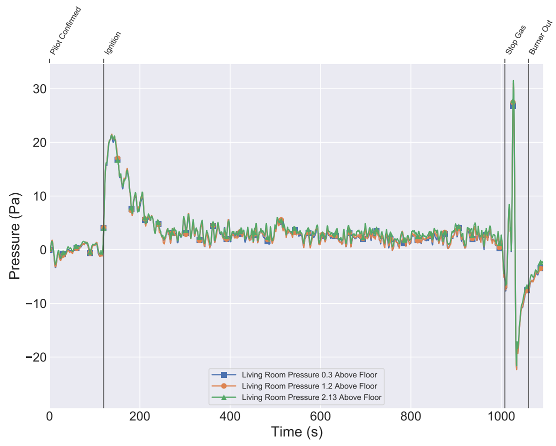

(d) Living Room Pressures

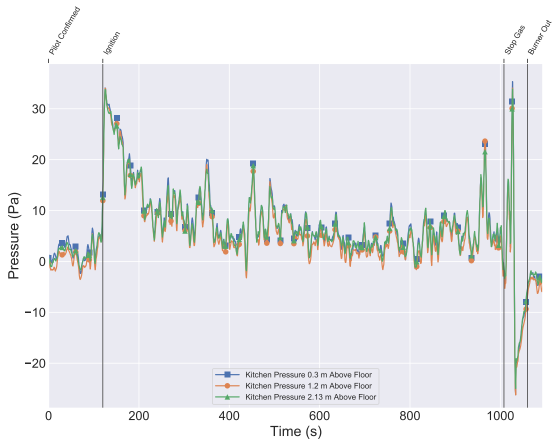

(e) Kitchen Pressures

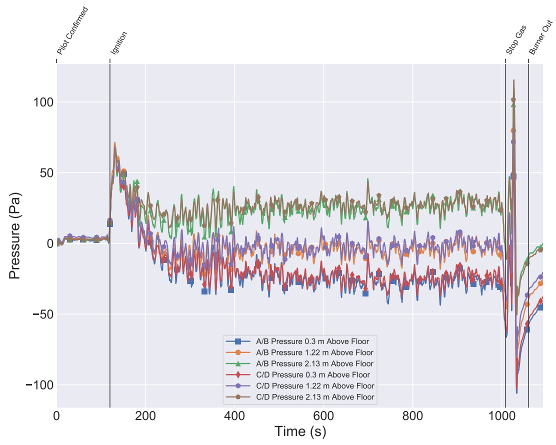

(f) Basement Pressures

Figure 5.159: Experiment 28 Pressures

The gas concentrations of oxygen and carbon dioxide are provided for bedroom 1, bedroom 2, bedroom 3 and the basement in Figure 5.160. Gas concentration trends were similar in the structure with the exception of bedroom 3. Whereas in Experiment 25 the gas concentrations in bedroom 3 were steady and unchanging, in this experiment a decrease of oxygen can be observed towards the end of the experiment. Basement oxygen concentrations were depleated more during this experiment because the larger fire required more oxygen for combustion.

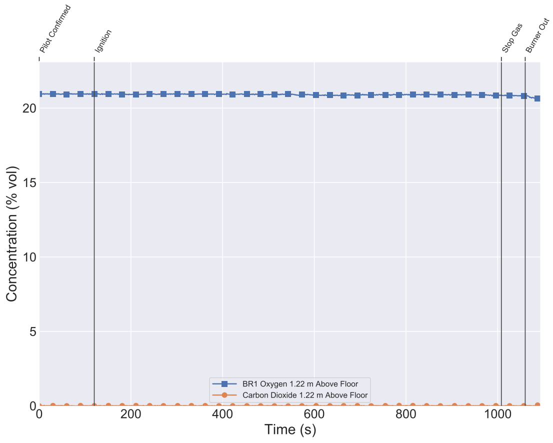

(a) Bedroom 1 Gases

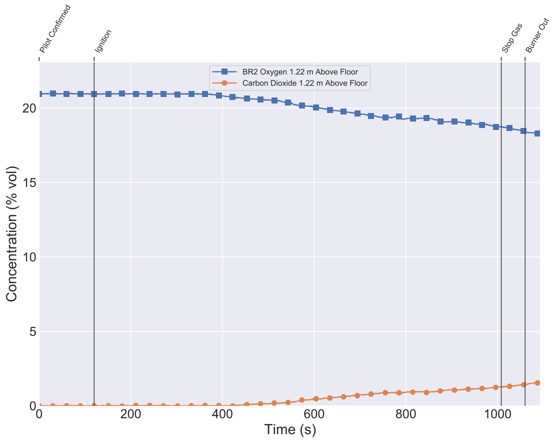

(b) Bedroom 2 Gases

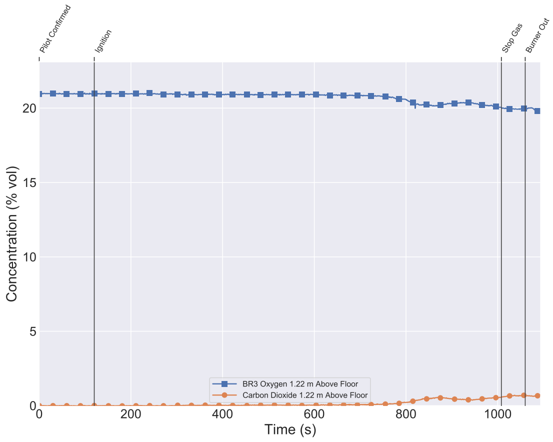

(c) Bedroom 3 Gases

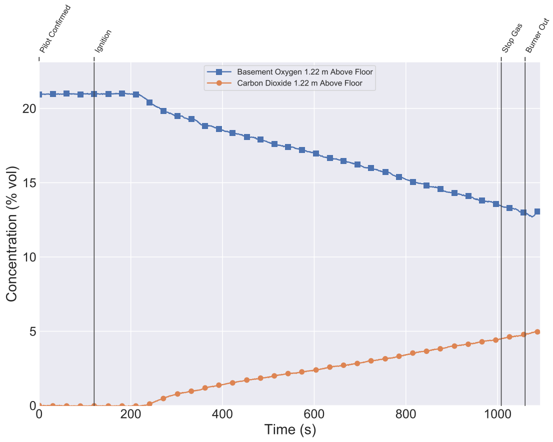

(d) Basement Gases

Figure 5.160: Experiment 28 Bedroom Gases

Figure 5.161 shows the temperatures of the supply and return vents in the structure. Vent temperatures throughout the structure behaved in a similar manner but with greater values than those recorded in Experiment 25.

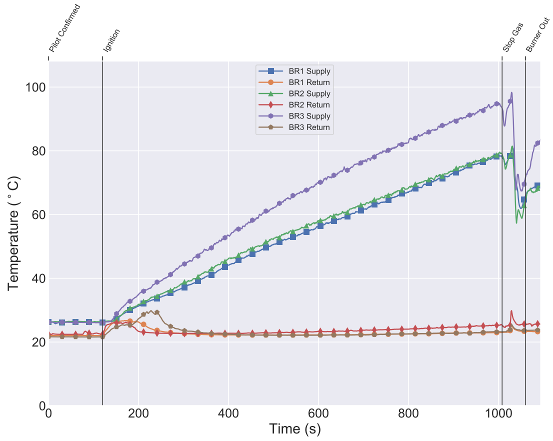

(a) Bedroom Vent Temperatures

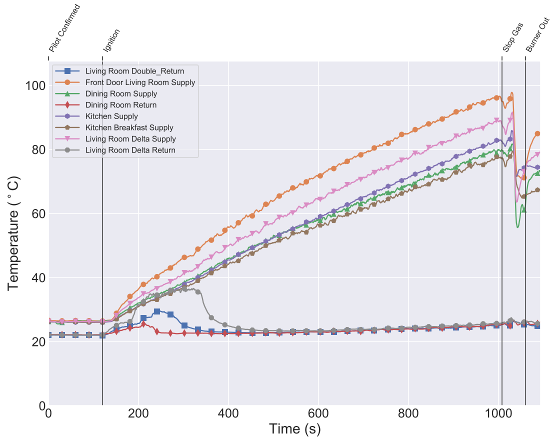

(b) First Floor Vent Temperatures

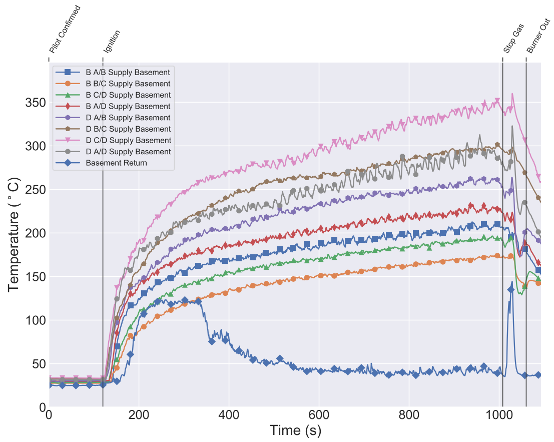

(c) Basement Vent Temperatures

Figure 5.161: Experiment 28 Vent Temperatures

The temperature and velocity within the supply duct are provided in Figure 5.162. Supply duct temperatures and velocities were similar between this experiment and Experiment 25.

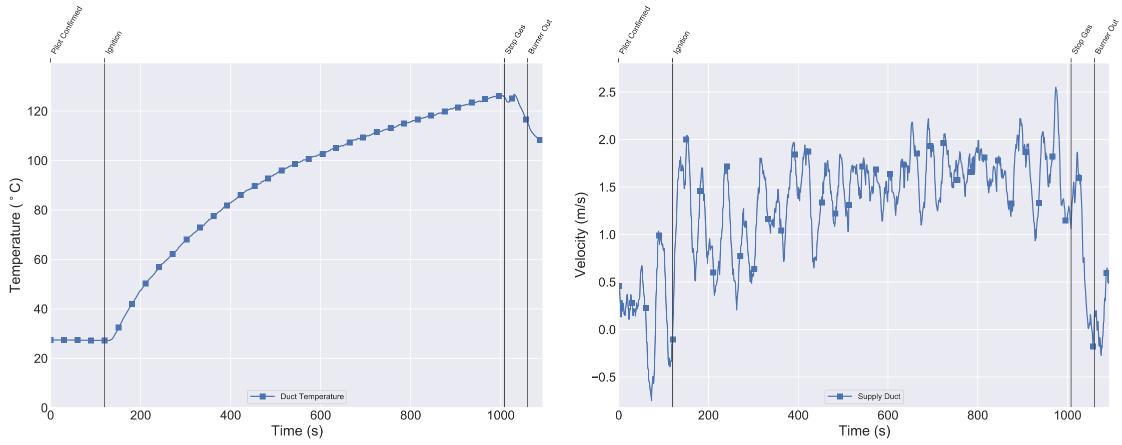

(a) Supply Duct Temperature

(b) Supply Duct Velocity

Figure 5.162: Experiment 28 Supply Duct Temperature and Velocity

5.3.7 Experiment 29

Gas Burner Experiment 29 was performed with the HVAC system off. The flow rate of propane to the gas burner was controlled so that a heat release rate of approximately 393 kW was produced. The stairwell door to the basement was open during this experiment. The doorway to bedroom 1 and all exterior vents were closed for this experiment. Experiment 29 was performed to see how smoke and fire gases moved through the structure via the HVAC system with a higher HRR and the basement stair door open. Comparisons were made to Experiment 28. The events of interest for Experiment 29 are presented in Table 5.28.

Table 5.28: Experiment 29 Events

Event	Elapsed Time (seconds)
Pilot Confirmed	0
Ignition	131
Stop Gas	1212
Burner Out	1279

The time versus temperature graphs for Experiment 29 are provided below. Figure 5.163 displays the basement temperatures and Figure 5.164 displays the first floor temperatures.

Temperatures in the basement behaved similar to the trends and values of Experiment 28.

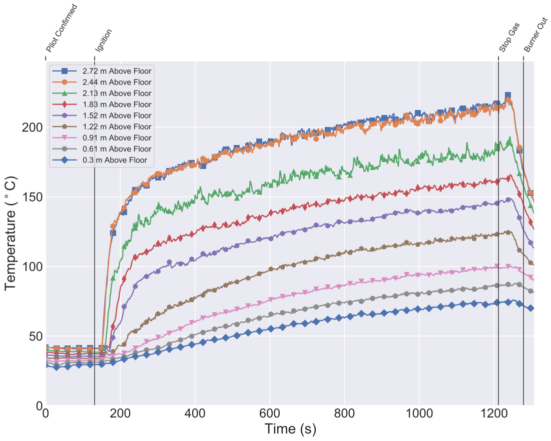

(a) Basement A/B Corner Temperatures

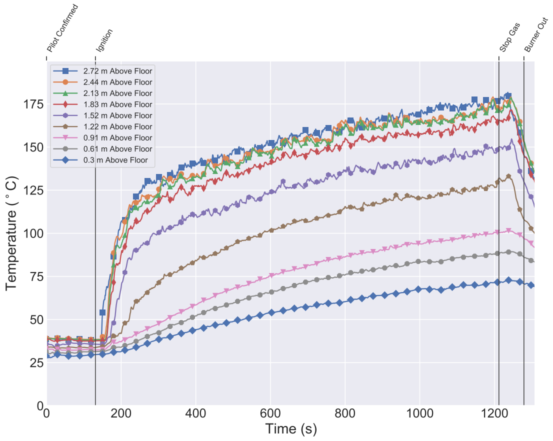

(b) Basement B/C Corner Temperatures

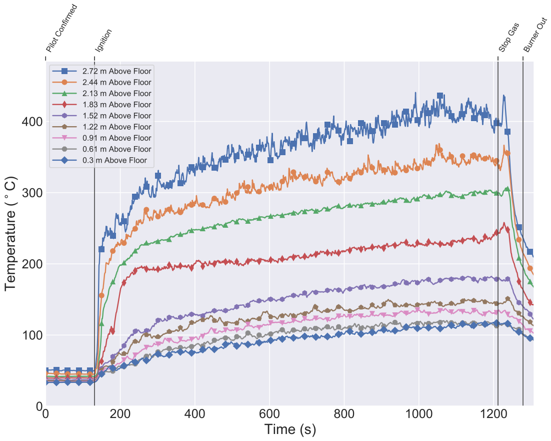

(c) Basement C/D Corner Temperatures

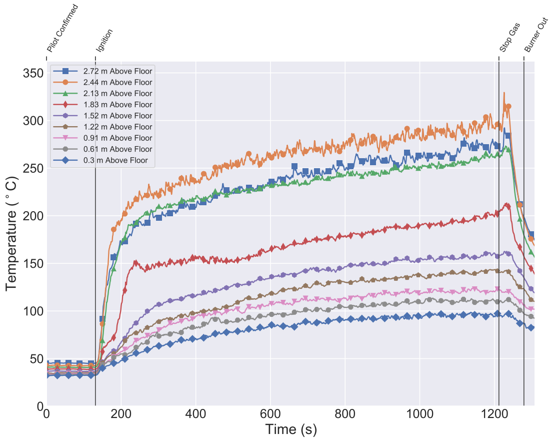

(d) Basement A/D Corner Temperatures

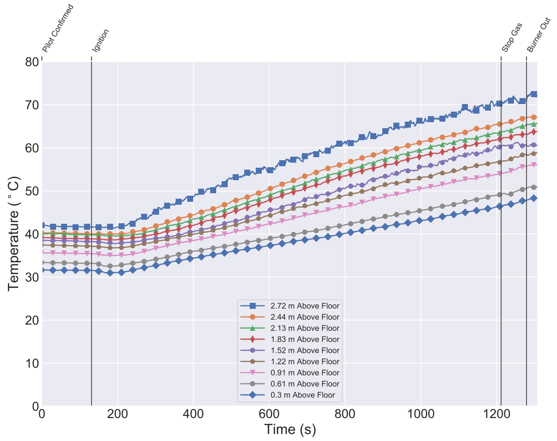

(e) Basement Mechanical Room Temperatures

Figure 5.163: Experiment 29 Basement Temperatures

Temperatures on the first floor were greater in this experiment than in Experiment 28 because of the open staircase door.

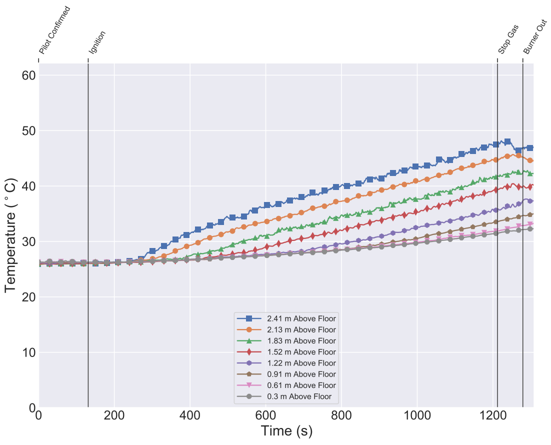

(a) Bedroom 1 Temperatures

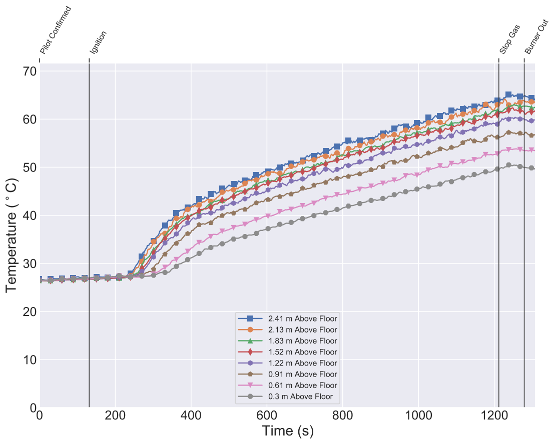

(b) Bedroom 2 Temperatures

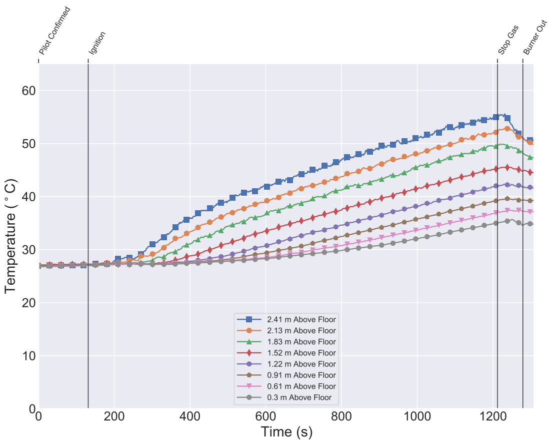

(c) Bedroom 3 Temperatures

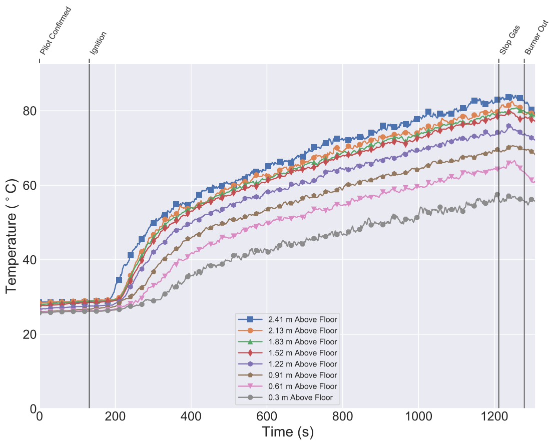

(d) Living Room Temperatures

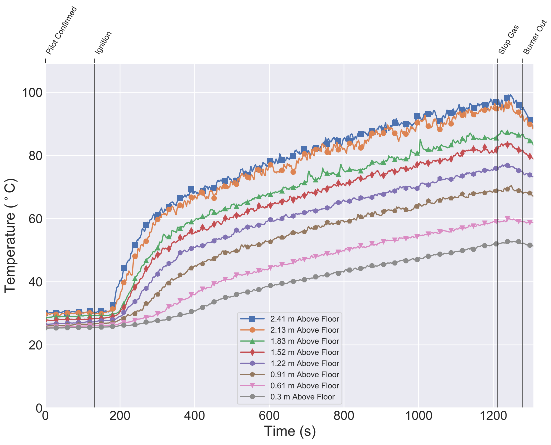

(e) Dining Room Temperatures

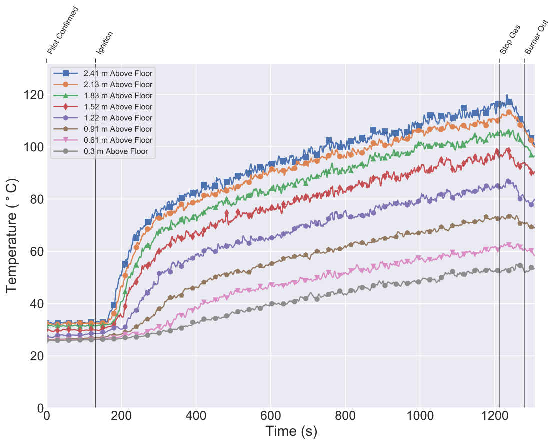

(f) Kitchen Temperatures

Figure 5.164: Experiment 29 First Floor Temperatures

The pressures within each room are shown in Figure 5.165. Pressures throughout the structure experienced a greater post ignition pressure spike in this experiment by nearly two times the values recorded in Experiment 28. The steady pressures which followed the spikes were similar in values to those of the previous experiment.

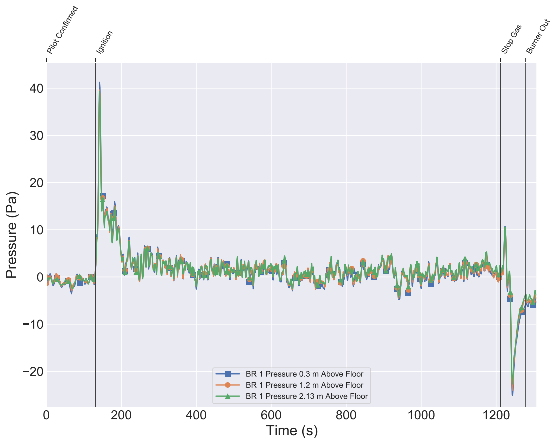

(a) Bedroom 1 Pressures

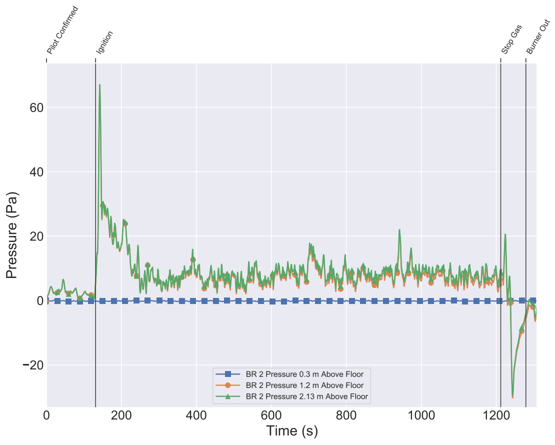

(b) Bedroom 2 Pressures

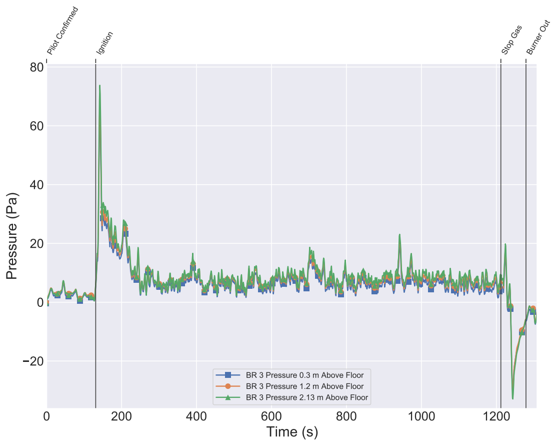

(c) Bedroom 3 Pressures

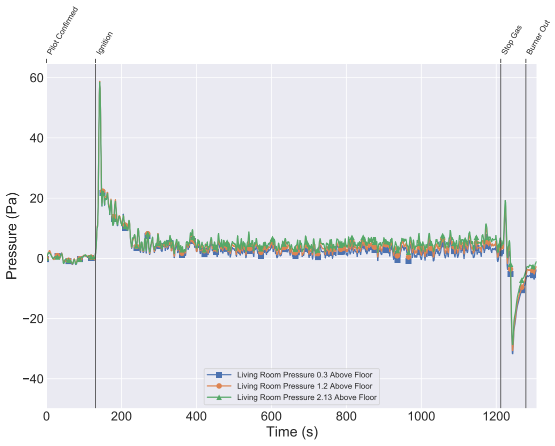

(d) Living Room Pressures

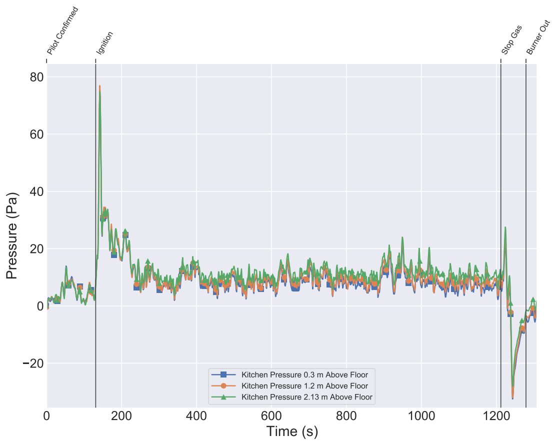

(e) Kitchen Pressures

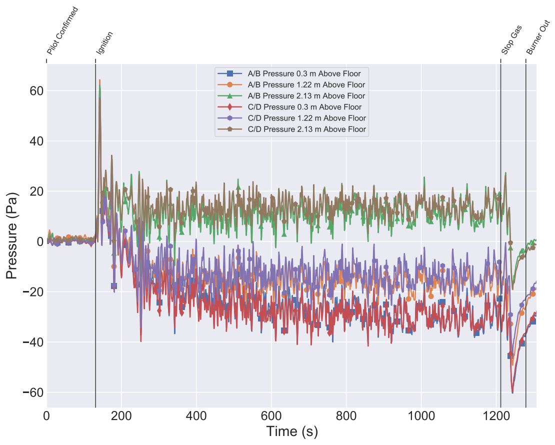

(f) Basement Pressures

Figure 5.165: Experiment 29 Pressures

The gas concentrations of oxygen and carbon dioxide are provided for bedroom 1, bedroom 2, bedroom 3 and the basement in Figure 5.166. Gas concentration changes were greater in all the bedrooms as a result of the staircase door being open. Oxygen concentrations dropped to approximately 17%, 15% and 16% in bedrooms 1, 2 and 3, respectively. Similarly, Carbon Monoxide levels increased approximately 2%, 4% and 3% in bedrooms 1, 2 and 3, respectively. Gas concentrations in the basement behaved the same as the data recorded in Experiment 28.

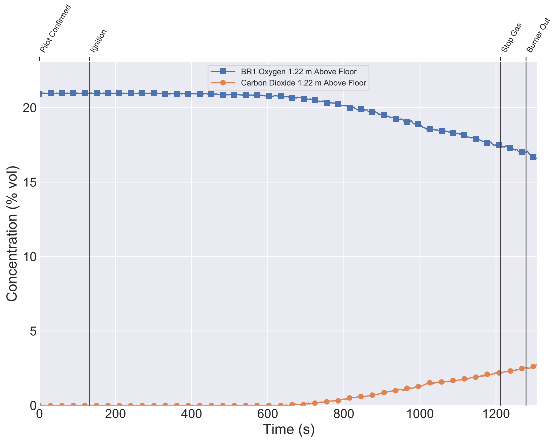

(a) Bedroom 1 Gases

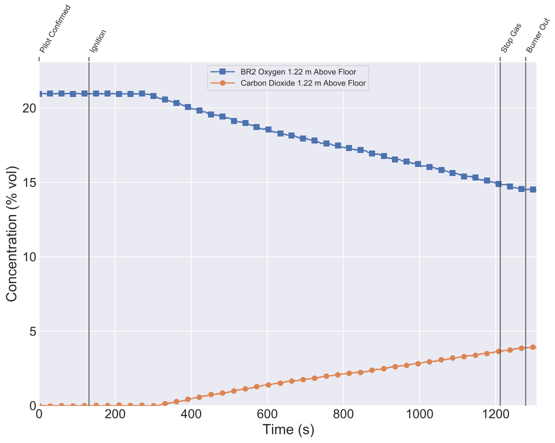

(b) Bedroom 2 Gases

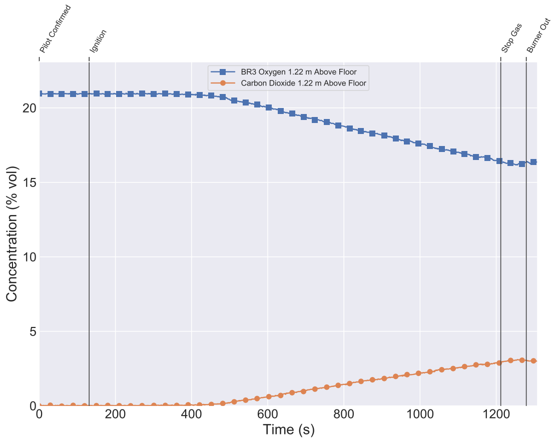

(c) Bedroom 3 Gases

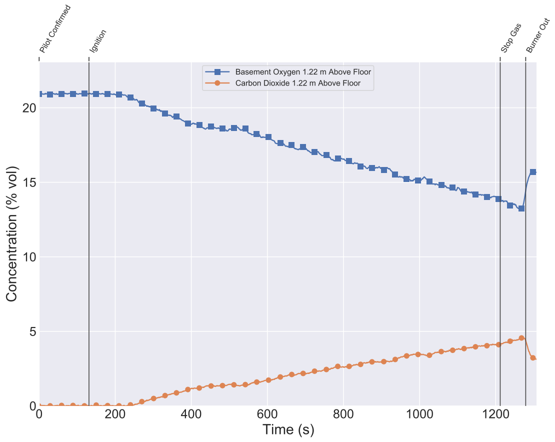

(d) Basement Gases

Figure 5.166: Experiment 29 Bedroom Gases

Figure 5.167 shows the temperatures of the supply and return vents in the structure. Supply vent temperatures on the first floor behaved similarly to those of Experiment 28 but remained approximately 10 °C less outside the bedrooms. Return vent temperatures on the first floor increased more during this experiment than in Experiment 28. Basement supply vent temperatures behaved similarly between the two experiments but the return vent temperature was steady and relatively unchanged throughout this experiment.

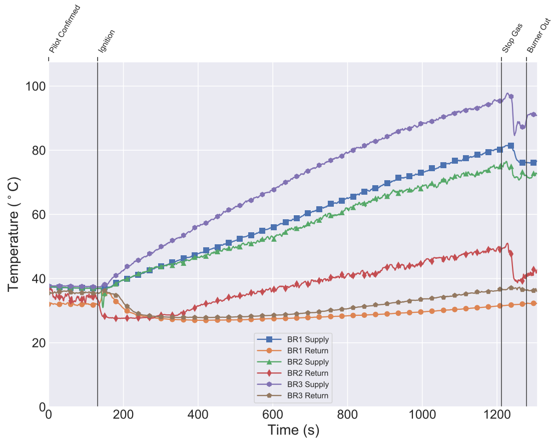

(a) Bedroom Vent Temperatures

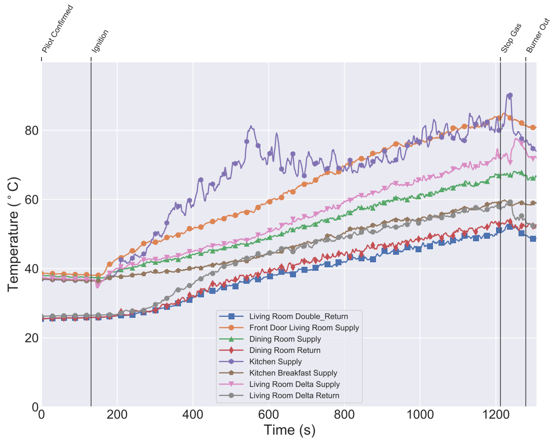

(b) First Floor Vent Temperatures

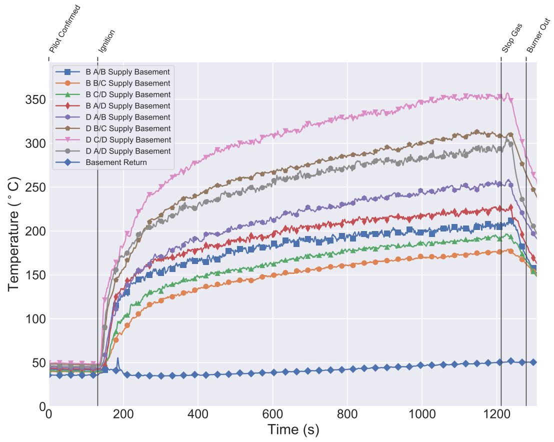

(c) Basement Vent Temperatures

Figure 5.167: Experiment 29 Vent Temperatures

The temperature and velocity within the supply duct are provided in Figure 5.168. The temperature and velocity are similar between this experiment and Experiment 28.

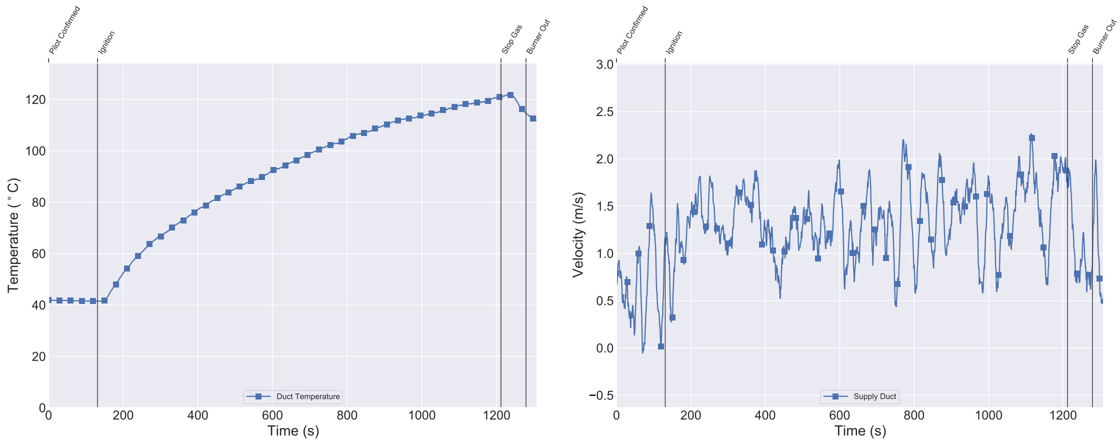

(a) Supply Duct Temperature

(b) Supply Duct Velocity

Figure 5.168: Experiment 29 Supply Duct Temperature and Velocity

5.4 HVAC Status Comparison

Displayed in the tables below are four comparisons made at 200 second time intervals following the pilot ignition. The locations for the comparison points are as follows:

- Fire room ceiling temperature in degrees Celcius
- Fire room supply vent temperature in degrees Celcius
- Bedroom 3 ceiling temperature in degrees Celcius
- Bedroom 3 supply vent temperature in degrees Celcius

- HVAC riser duct temperature in degrees Celcius
- Fire room oxygen concentration
- Bedroom 3 oxygen concentration

In Table 5.29, three experiments were compared. Experiment 1 with a passive HVAC setting, Experiment 2 with the HVAC set to A/C, and Experiment 4 with the HVAC set to heat. All three experiments were executed with the same parameters, with the exception of the HVAC status.

Table 5.29: Bedroom 1 HVAC Status Comparison

Description<br>HVAC status	Exp 1<br>Passive	Exp 2<br>A/C	Exp 4<br>Heat
Time = 200 seconds			
Fire Room Ceiling Temp	167	197	192
Fire Room Supply Temp	149	10.9	38.5
BR3 Ceiling Temp	21.7	23.1	26.2
BR3 Supply Temp	22.9	9.9	43.3
HVAC Temp	20.6	8.4	48.4
Fire Room O2	20.8%	20.4%	20.1%
BR3 O2	20.9%	20.9%	20.9%
Time = 400 seconds			
Fire Room Ceiling Temp	208	225	218
Fire Room Supply Temp	225	11.6	214
BR3 Ceiling Temp	27.2	25.1	28.7
BR3 Supply Temp	50.3	9.9	38.2
HVAC Temp	28.8	8.7	36.5
Fire Room O2	20.0%	19.5%	19.6%
BR3 O2	20.9%	20.8%	20.8%
Time = 600 seconds			
Fire Room Ceiling Temp	223	260	225
Fire Room Supply Temp	237	212	247
BR3 Ceiling Temp	33.2	27.6	33.6
BR3 Supply Temp	71.9	10.8	68.9
HVAC Temp	35.1	10.0	44.1
Fire Room O2	19.6%	18.9%	19.0%
BR3 O2	20.8%	20.7%	20.9%
Time = 800 seconds			
Fire Room Ceiling Temp	252	272	245
Fire Room Supply Temp	260	273	249
BR3 Ceiling Temp	39.3	29.8	38.7
BR3 Supply Temp	88.5	14.1	87.1
HVAC Temp	40.0	9.9	47.7
Fire Room O2	18.2%	18.5%	18.1%
BR3 O2	20.3%	20.5%	20.7%

*Temperatures are in degrees Celcius

From observation of the fire room ceiling and supply vent temperatures, over the course of the time steps it can be seen that the HVAC setting can impact the

temperature at that specific location. At 200 seconds, the fire room supply vent is different for all three experiments because of the HVAC setting. At 400 seconds, the A/C setting in Experiment 2 is still able to keep the supply vent in the fire room at a similar temperature to that observed at 200 seconds. Bedroom 3 ceiling and supply vent temperatures display the path of heat transfer into bedroom 3. With the exception of the A/C experiment, the HVAC system is providing a source of temperature increase in bedroom 3. The HVAC temperature shows the penetration of heat into the supply duct riser over the course of these experiments. The oxygen readings show that the HVAC setting did not have a significant impact during these time steps.

In Table 5.30, four experiments were compared. Experiment 16 with a passive HVAC setting, Experiment 17 with the HVAC set to A/C, Experiment 18 with a passive HVAC setting and a higher HRR, and Experiment 19 with the HVAC set to A/C and a higher HRR. All four experiments were executed with the same parameters, with the exception of the HVAC status and HRR. In Experiment 19, the gas burner was shut off prior to the 800 second time step. Data was still included to show trends within Experiment 19, but not for equal comparison to Experiment 18.

Table 5.30: Living Room HVAC Status Comparison

Description<br>HVAC status	Exp 16<br>Passive	Exp 17<br>A/C	Exp 18<br>Passive	Exp 19<br>A/C
Time = 200 seconds				
Fire Room Ceiling Temp	119	126	141	124
Fire Room Supply Temp	111	12.3	137	13.1
BR3 Ceiling Temp	21.8	25.1	24.9	26.3
BR3 Supply Temp	27.6	11.5	33.4	12.5
HVAC Temp	23.5	9.9	28.7	10.7
Fire Room O2	20.9%	20.9%	20.9%	20.6%
BR3 O2	20.8%	20.9%	20.9%	20.9%
Time = 400 seconds				
Fire Room Ceiling Temp	135	176	278	255
Fire Room Supply Temp	130	13.1	270	15.2
BR3 Ceiling Temp	26.2	25.9	31.8	29.0
BR3 Supply Temp	42.2	11.9	66.3	13.1
HVAC Temp	31.8	10.9	48.8	12.0
Fire Room O2	20.7%	20.6%	20.7%	20.5%
BR3 O2	20.9%	20.9%	20.9%	20.9%
Time = 600 seconds				
Fire Room Ceiling Temp	145	184	335	271
Fire Room Supply Temp	146	14.5	287	253
BR3 Ceiling Temp	30.2	27.2	39.2	31.1
BR3 Supply Temp	53.4	12.9	89.5	14.4
HVAC Temp	38.1	12.2	64.2	13.4
Fire Room O2	20.4%	20.3%	20.4%	20.6%
BR3 O2	20.9%	20.9%	20.9%	20.9%
Time = 800 seconds				
Fire Room Ceiling Temp	154	200	348	**182
Fire Room Supply Temp	162	191	336	**177
BR3 Ceiling Temp	33.2	28.4	46.7	**37.0
BR3 Supply Temp	62.9	13.6	109	**52.6
HVAC Temp	43.5	11.1	75.2	**20.9
Fire Room O2	20.2%	20.1%	20.0%	**19.7%
BR3 O2	20.9%	20.9%	20.8%	**20.9%

*Temperatures are in degrees Celcius
**Gas burner shut off prior to time step

Observation of the data presented for the living room experiments yield similar comparisons to those made from the bedroom 1 experiments. The higher HRR

experiments did not show any significant differences.

In Table 5.31, three experiments were compared. Experiment 25 with a passive HVAC setting, Experiment 26 with the HVAC set to A/C, and Experiment 27 with the HVAC set to heat. All three experiments were executed with the same parameters, with the exception of the HVAC status. In experiments 26 and 27, the gas burner was shut off prior to the 800 second time step. Data was still included for these experiments to show trends within their data sets, but not for equal comparisons to other experiments.

Table 5.31: Basement HVAC Status Comparison

Description<br>HVAC status	Exp 25<br>Passive	Exp 26<br>A/C	Exp 27<br>Heat
Time = 200 seconds			
Fire Room Ceiling Temp	149	150	103
Fire Room Supply Temp	155	26.8	45.4
BR3 Ceiling Temp	27.4	27.1	22.0
BR3 Supply Temp	32.6	22.8	43.5
HVAC Temp	47.5	24.4	50.8
Fire Room O2	20.8%	20.6%	20.9%
BR3 O2	20.9%	20.9%	20.9%
Time = 400 seconds			
Fire Room Ceiling Temp	194	191	135
Fire Room Supply Temp	203	32.1	49.3
BR3 Ceiling Temp	29.9	27.6	25.0
BR3 Supply Temp	48.4	26.8	47.8
HVAC Temp	77.5	29.1	56.1
Fire Room O2	19.3%	19.3%	20.0%
BR3 O2	20.9%	20.9%	20.9%
Time = 600 seconds			
Fire Room Ceiling Temp	216	207	150
Fire Room Supply Temp	219	72.2	46.4
BR3 Ceiling Temp	32.9	27.9	29.8
BR3 Supply Temp	64.0	26.0	49.2
HVAC Temp	96.7	27.4	57.8
Fire Room O2	18.0%	18.0%	19.2%
BR3 O2	20.9%	20.9%	20.7%
Time = 800 seconds			
Fire Room Ceiling Temp	227	**101	**94.3
Fire Room Supply Temp	232	**32.9	**44.3
BR3 Ceiling Temp	36.2	**28.2	**31.4
BR3 Supply Temp	75.7	**24.2	**46.2
HVAC Temp	110	**25.0	**54.5
Fire Room O2	16.9%	**18.1%	**19.6%
BR3 O2	20.9%	**20.9%	**20.4%

*Temperatures are in degrees Celcius
**Gas burner shut off prior to time step

Observation of the basement A/C experiment shows a significant increase in the HVAC supply duct temperature when compared to the other fire locations. The

increased temperature could be caused by warmer air being drawn in through the basement return vent, which accounts for a large portion of air supplied to the furnace.

Chapter 6: Discussion of Modeling Results

6.1 Cold Flow Analysis

The first modeling analysis performed was used to establish the losses through the HVAC ductwork. Cold flow data was collected with the HVAC system running with the system fan on and the structure closed. A flowhood was utilized to collect the flowrates of air into all returns and out of all supplies. The collected values then became the target values for simulation outputs with an appropriate error range.

In order to run initial simulations to compare duct flow outputs, an initial value for these losses was determined. Utilizing the 2017 ASHRAE Handbook for Fundamentals [29], an initial minor loss was calculated for each duct section. Based on Table 3 in chapter 3 of the handbook, estimated minor losses for each duct segment were calculated based on the number of turns the duct segment made and the connection tees that airflow would traverse. FDS requires that all loss values be combined into a single loss for the entire duct section between nodes. For a duct segment that made a 90 ° turn, a loss of 0.6, 0.9 or 1.3 was assessed based on if the curve was a long radius, short radius or mitered corner, respectively. For airflow through a connection tee, if the airflow changed direction in the tee a loss of 1.8 was assessed. If the airflow continued straight through the tee then a loss of 0.5 was

assessed. The first simulation utilizing the calculated values was called ASHRAE 1. The results of ASHRAE 1 flow rates were compared to the values collected in the DELCO structure. Based on the collected data, losses were adjusted in a specific manner to direct more or less airflow to specific vents and parts of the structure.

A total of eight ASHRAE experiments, ASHRAE 1 to ASHRAE7, were modeled with ASHRAE 4 being performed at two grid size resolutions. ASHRAE 1 to ASHRAE 3 were performed with a grid resolution of 20 cm x 20 cm to obtain rough estimates on flowrates while reducing simulation time. In the first ASHRAE 4, 20 cm x 20 cm resolution, a majority of the flowrates converged on the target values. ASHRAE 4 was then executed again with the grid resolution decreased to 10 cm x 10 cm. Reducing the grid resolution size increased the precision of calculated flow values but also increased the simulation computational time. The refined ASHRAE 4 was then analyzed and losses were adjusted for ASHRAE 5. ASHRAE 5, 6 and 7 were performed with a resolution of 10 cm x 10 cm. Upon completion of ASHRAE 7, the simulated flowrates all were within the acceptable range of values established by the flowhood values.

The successful comparison of simulation values to experimental values was an important first step to take because it provides confidence that the HVAC submodel is successfully replicating the airflow through the ductwork. Being that the rest of the models executed were predicated on the proper modeling of the HVAC system, the ASHRAE models were a crucial task that was successfully executed. For further discussion on the cold flow analysis, see Appendix A.

6.2 Experimental Simulation Analysis

In this section the experiments modeled using FDS will be compared to the experimental data presented in Chapter 5.

6.2.1 Experiment 1

The time versus temperature graphs from the three bedrooms, the living room, the dining room and the kitchen are provided in Figure 6.1. From analysis of bedroom 1, it can be seen that the temperature trends identified in Experiment 1 were modeled with a good degree of accuracy. Maximum temperatures reached in bedroom 1 were approximately 250 °C in both the simulation and the experiment. Temperatures near the floor increased to approximately 75 °C in both as well. The thermal layer between hot upper gases and cooler lower gas layers is lower in the simulation than it is in the model for bedroom 1. This is evident by the higher temperatures recorded at the 0.91 m and 1.22 m height. Temperatures beyond the 'Gas Off' event will differ because the simulation gas burner was terminated at the 'Burner Off' event.

Similar to bedroom 1, the modeled temperatures in bedroom 2 trend with the experimental data for bedroom 2. The simulated maximum temperature in bedroom 2 was exceeded by the experimental maximum by approximately 25 °C at the ceiling while the temperature at the floor was slightly more than the experimental temperature. In the simulation, there was a shorter time delay between ignition and the increase of temperature in bedroom 2.

The bedroom 3 simulation temperature exceeded the experimental maximum temperature by approximately 20 °C at the ceiling and 5 °C at the floor. The greater temperature in bedroom 3 is due to more heat being transferred through the HVAC supply ductwork in the FDS HVAC submodel. It is stated in the FDS Users Manual that the current HVAC solver does not account for heat loss in the HVAC systems. Therefore all models should over-predict heat transfer through the HVAC system. It is to be expected that at any location where heat transfer was identified as primarily from the HVAC system, that the temperature predicted by FDS will be greater in value than in the experiment.

Comparison of the living room experiments shows good agreement between temperatures at the ceiling and the floor. At both of these locations temperatures were within 20 °C between experimental and simulated results. Experimental temperatures were slightly higher at the ceiling and slightly lower at the floor. Similar to the results in the living room, the dining room temperatures and trends agreed well between the experimental and simulated results. Temperatures were within 20 °C with the experimental temperature at the ceiling being slightly higher than the simulated and at the floor slightly lower. In the kitchen the maximum temperature was approximately 80 °C in both the simulation and the experimental data. The temperature at the floor in the simulation exceeded the experimental temperature by approximately 10 °C.

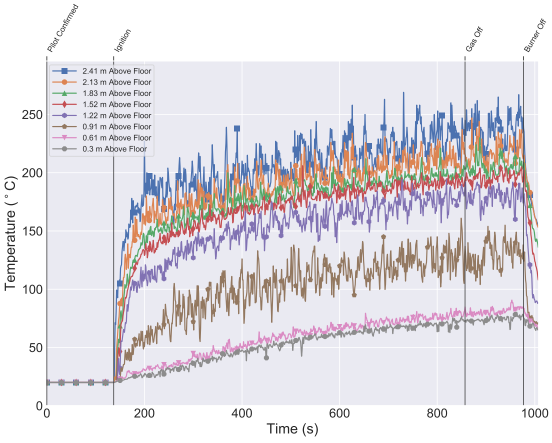

(a) Bedroom 1 Temperatures

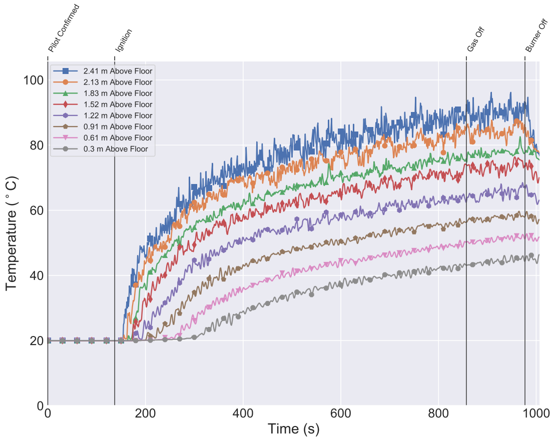

(b) Bedroom 2 Temperatures

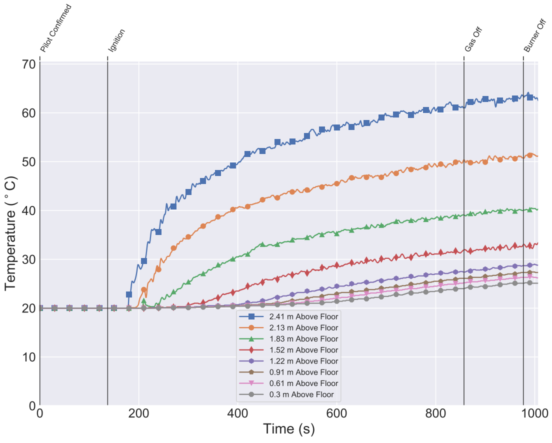

(c) Bedroom 3 Temperatures

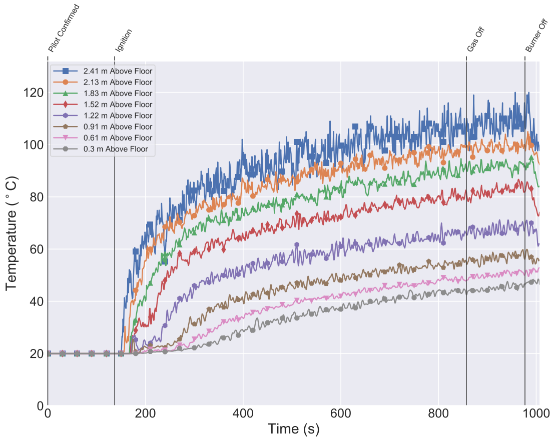

(d) Living Room Temperatures

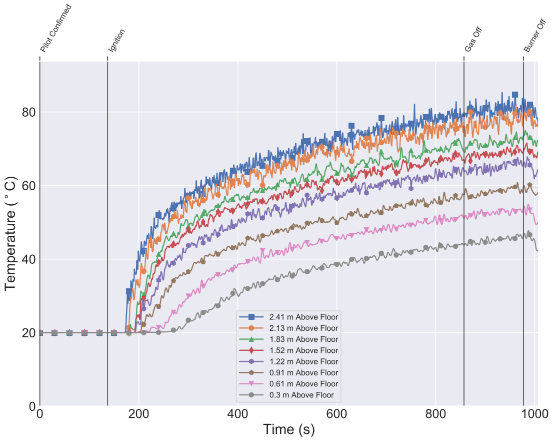

(e) Dining Room Temperatures

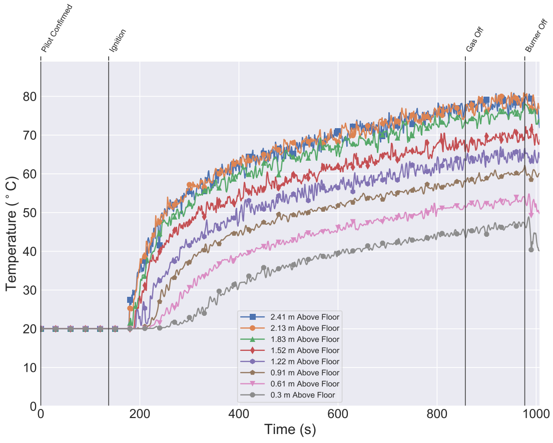

(f) Kitchen Temperatures

Figure 6.1: FDS Experiment 1 First Floor Temperatures

The time versus temperature graphs for the four basement quadrants are provided in Figure 6.2. From the figures it can be seen that with a simulated fire in bedroom 1, there was minimal temperature increase measured in the basement. This slight temperature change is less than 1 °C but could have been caused by the increased heat transfer through the HVAC ductwork.

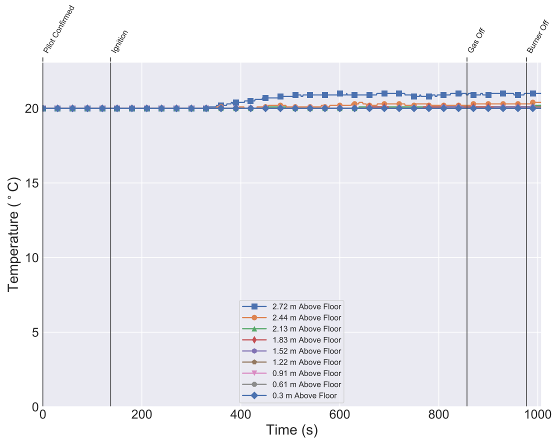

(a) Basement A Quadrant Temperatures

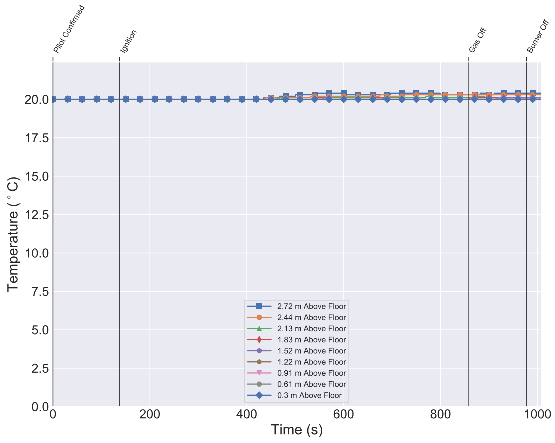

(b) Basement B Quadrant Temperatures

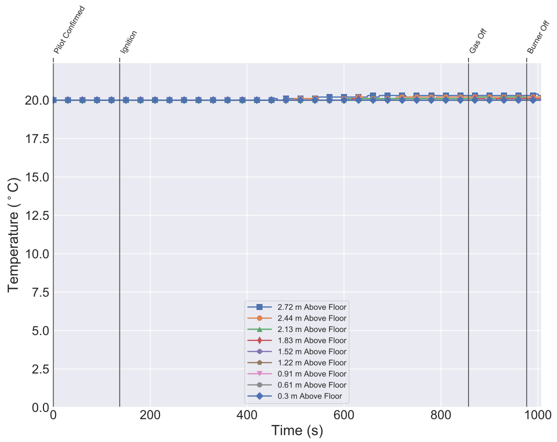

(c) Basement C Quadrant Temperatures

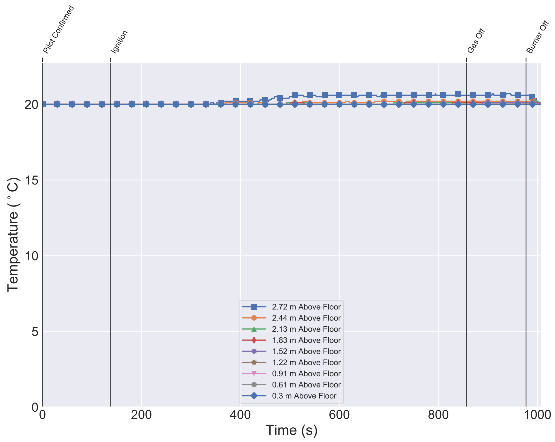

(d) Basement D Quadrant Temperatures

Figure 6.2: FDS Experiment 1 Basement Temperatures

The pressures within each room are shown in Figure 6.3. The pressure charts

generated by the simulation vary greatly to those of Experiment 1. In the simulation, at all locations following ignition there was a pressure spike of almost 50 Pa. Following the pressure spike, pressures all returned to lower readings. Pressures in bedroom 1 following the spike leveled out with steady pressures only a few pascals greater than what was recorded in the experiment. A similar trend was identified for all of the pressure measurements throughout the structure. In the simulation, a more uniform pressure was recorded throughout the structure. All simulation pressures remained relatively steady with readings slightly greater than the ambient pressure.

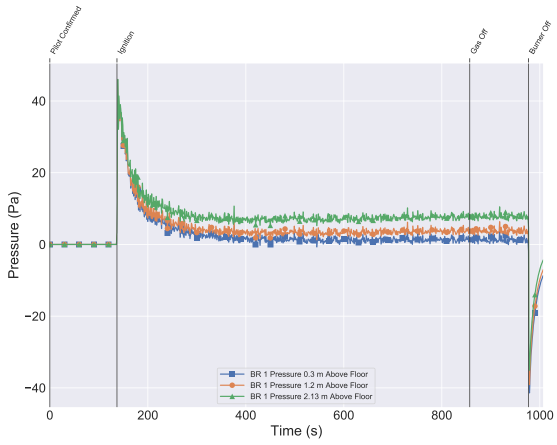

(a) Bedroom 1 Pressures

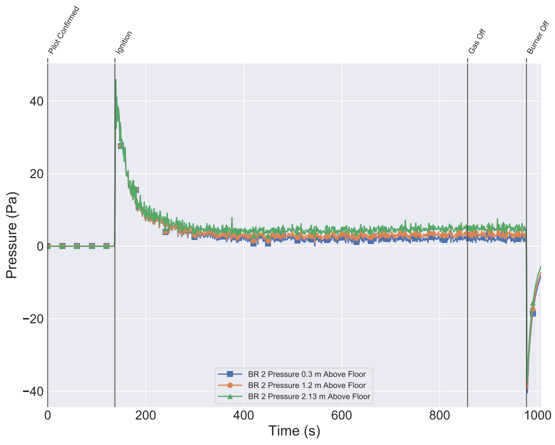

(b) Bedroom 2 Pressures

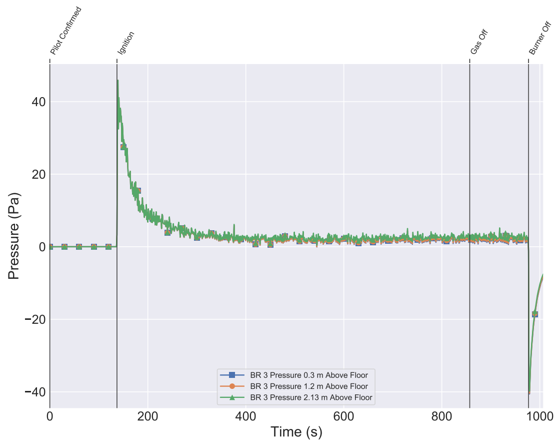

(c) Bedroom 3 Pressures

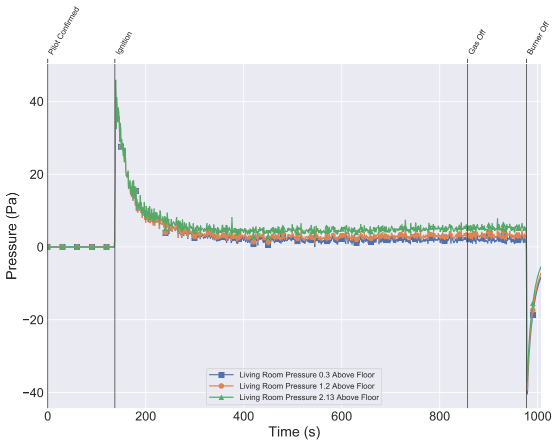

(d) Living Room Pressures

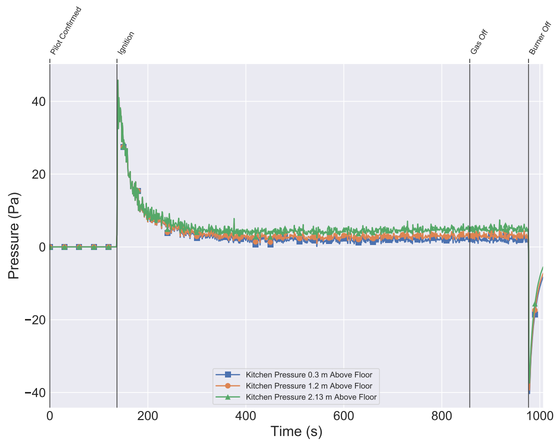

(e) Kitchen Pressures

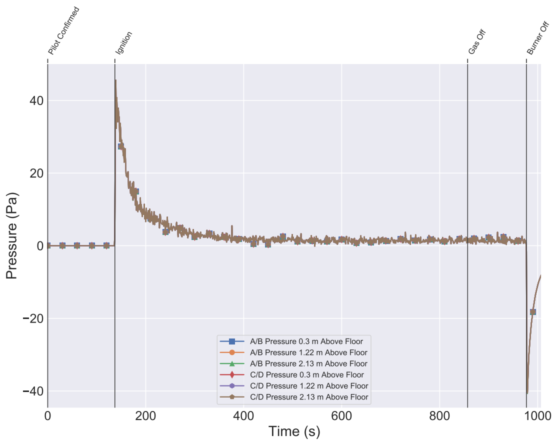

(f) Basement Pressures

Figure 6.3: FDS Experiment 1 Pressures

The concentrations of oxygen and carbon dioxide are provided for the three bedrooms and the basement in Figure 6.4. Water vapor concentrations are included for bedroom 2, bedroom 3 and the basement. Comparison of oxygen concentrations in bedroom 1 show that the simulation had a quicker response to oxygen consumption than the experimental data and that more oxygen was consumed, thus lowering the oxygen concentration in bedroom 1 to approximately 16%. Oxygen concentrations in bedroom 2 followed a similar trend with the simulation showing a drop in oxygen earlier than the experiment. Oxygen concentrations at the 'Gas Off' event were similar between the experimental and simulation data. Similarly in bedroom 2, the water vapor concentrations follwed a similar trend with an equal rise in concentration. In bedroom 3 the simulated oxygen and water vapor concentrations followed a similar trend as the experimental data. In the basement, the simulated data matched the experimental data showing no change in measured concentrations.

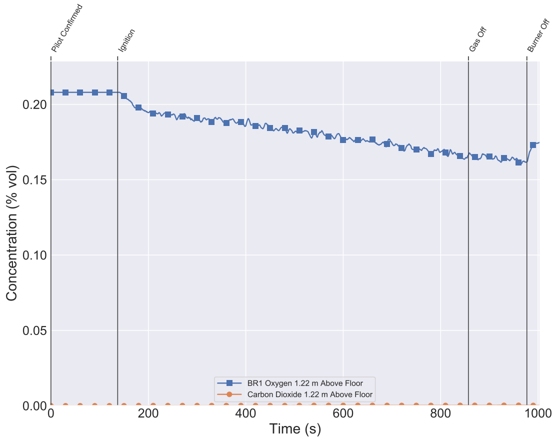

(a) Bedroom 1 Gases

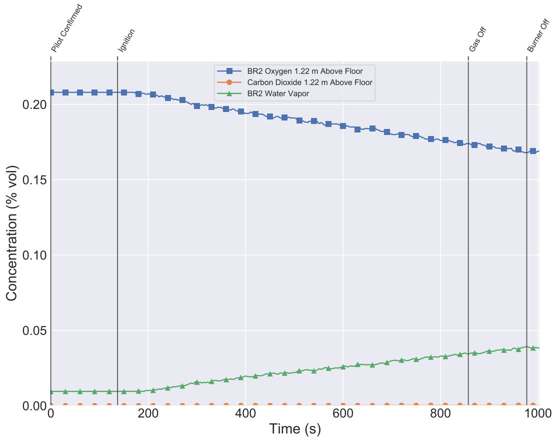

(b) Bedroom 2 Gases

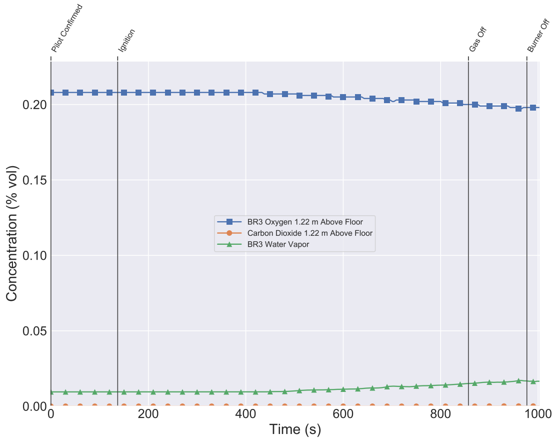

(c) Bedroom 3 Gases

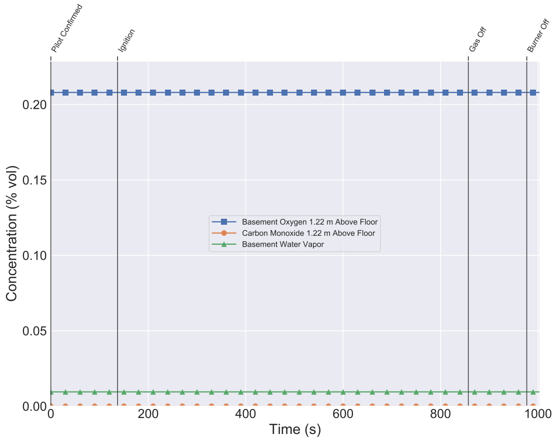

(d) Basement Gases

Figure 6.4: FDS Experiment 1 Gases

Figure 6.5 shows the temperatures of the supply and return vents in the structure. As was stated when analysing bedroom temperatures, the HVAC solver in FDS does not account for heat loss within the ducts, thus the temperatures recorded in the ductwork and at vents should be expected to be higher than the experimental results. From comparison of Figure 6.5(a) to the bedroom vent temperatures in Experiment 1, it can be seen that the simulated supply vent temperatures in bedrooms

2 and 3 exceed the experimental data by 40 °C and 70 °C, respectively. The greater heat transfer through the ducts was also visible in the higher simulated bedroom 3 temperatures. The return vent temperatures presented in the simulation are similar to those seen in the experimental data.

Figure 6.5(b) shows the rest of the vents on the first floor. With the exception of the living room supply vents, all other supply vents on the first floor exceeded their experimental values by approximately 50 °C. Similarly, the return vents throughout the first floor exceeded the experimental values by approximately 15 °C. Vent temperatures in the basement behaved similarly between experimental and simulated results.

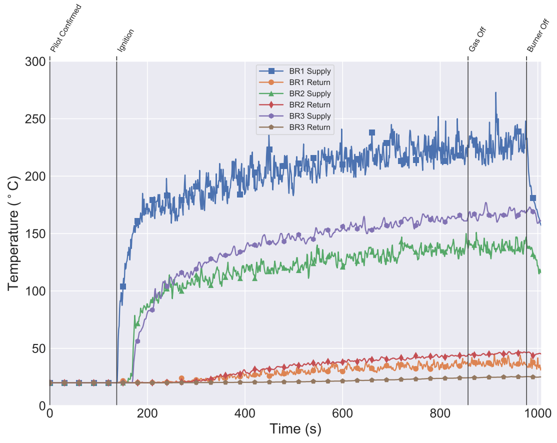

(a) Bedroom Vent Temperatures

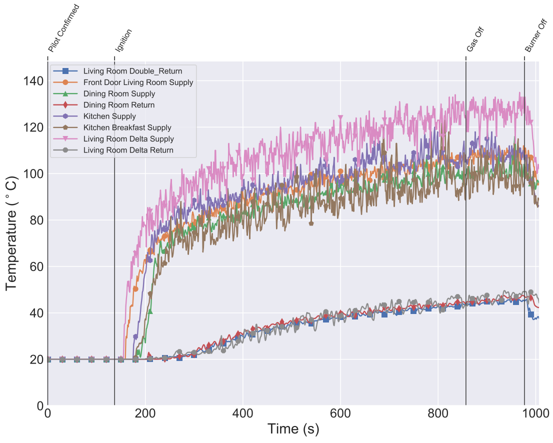

(b) First Floor Vent Temperatures

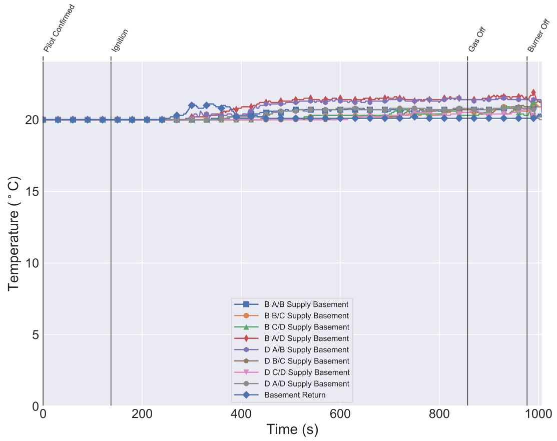

(c) Basement Vent Temperatures

Figure 6.5: FDS Experiment 1 Vent Temperatures

Table 6.1 below shows a comparison of fire room data and bedroom 3 data collected from experimental and simulated data. Based on the data presented, the experimental and simulated data showed good agreement with the exception of the bedroom 3 ceiling temperature with a differential at 52.1%. As was expected based on the lack of heat loss within the FDS HVAC ductwork, the simulated bedroom 3 temperature is greater than the experimental data.

Table 6.1: Experiment 1 Comparisons

Description	Experimental Value	FDS Value	Difference
Max Fire Room Ceiling Temperature	267.0	269.0	0.7%
Max Fire Room Floor Temperature	67.6	80.3	18.8%
Min Fire Room O2	17.9%	16.0%	-10.6%
Max BR3 Ceiling Temperature	42.2	64.2	52.1%
Max BR3 Floor Temperature	22.6	25.2	11.5%
Min BR3 O2	18.8%	19.7%	4.8%

6.2.2 Experiment 12

The time versus temperature graphs from the three bedrooms, the living room, the dining room and the kitchen are provided in Figure 6.6. Comparison of bedroom 1 temperatures shows that the experimental thermal hot gas layer was slightly higher than the FDS model. This is supported by a consistently higher temperature at the 0.91 m height. With the exception of the HGL, the data collected in bedroom 1 was similar between the experimental and simulated data. Experimental bedroom 2 temperatrues exceeded the simulated values by approximately 20 °C above the 1.22 m height. Simulated temperatures in bedroom 3 exceeded the experimental celing temperature by approximately 20 °C and displayed a rapid initial temperature increase that was not present in the experimental data. This increased heat transfer into bedroom 3 is attributed to the overprediction of heat through the HVAC supply system.

Temperatures in the living room, dining room, and kitchen all displayed similar trends and values between the experimental and simulated data.

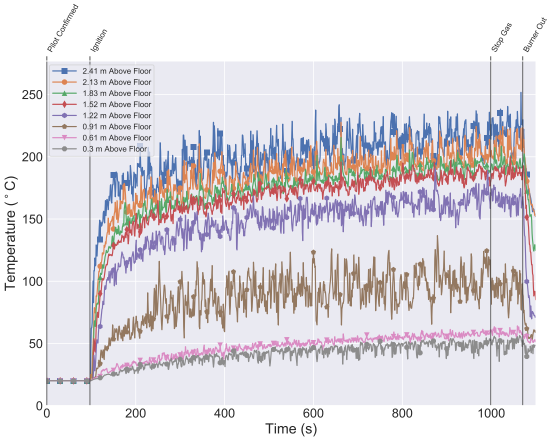

(a) Bedroom 1 Temperatures

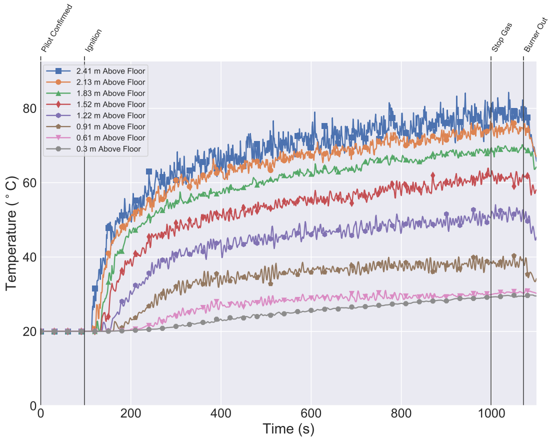

(b) Bedroom 2 Temperatures

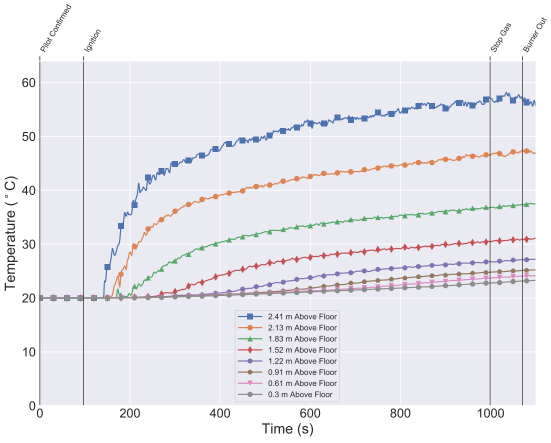

(c) Bedroom 3 Temperatures

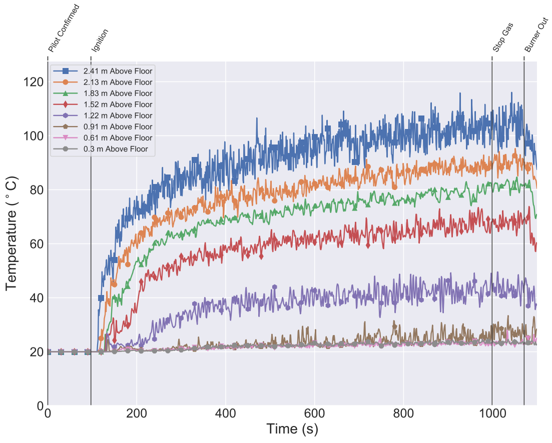

(d) Living Room Temperatures

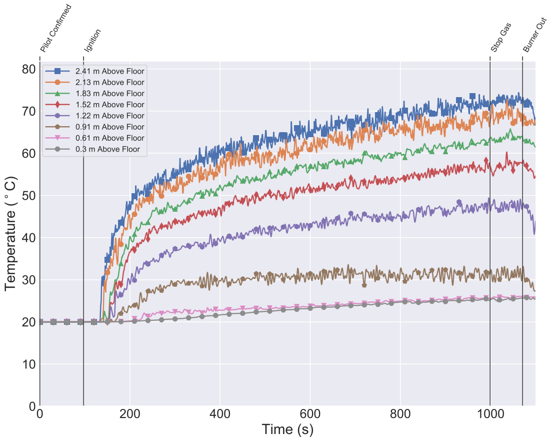

(e) Dining Room Temperatures

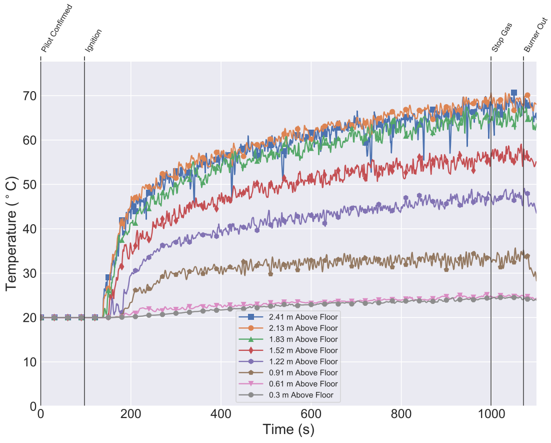

(f) Kitchen Temperatures

Figure 6.6: FDS Experiment 12 First Floor Temperatures

The time versus temperature graphs for the four basement quadrants are provided in Figure 6.7. Similar to the simulated fire in bedroom 1, there was minimal temperature increase measured in the basement.

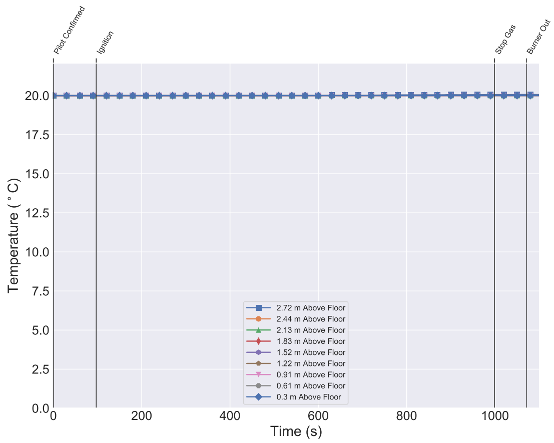

(a) Basement A Quadrant Temperatures

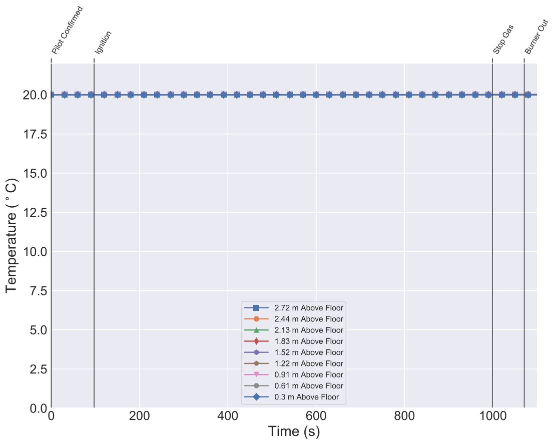

(b) Basement B Quadrant Temperatures

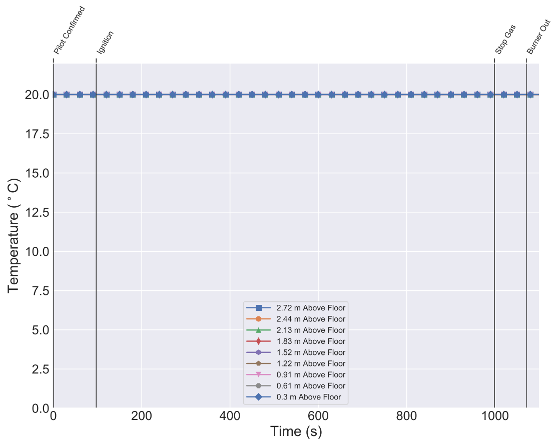

(c) Basement C Quadrant Temperatures

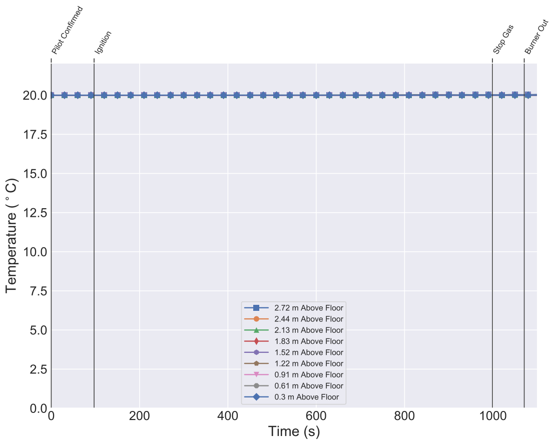

(d) Basement D Quadrant Temperatures

Figure 6.7: FDS Experiment 12 Basement Temperatures

The pressures within each room are shown in Figure 6.8. Pressure predictions throughout the structure varied between the experimental and simulated values. Bedroom 1 values were the most similar with the simulated values at 0.3 m being

steady around 1 Pa below ambient, 1.2 m slightly below 1 Pa above the ambient and 2.13 m reaching almost 5 Pa above the ambient. Pressures in bedrooms 2 and 3 varied greatly from the experimental values which fluctuated at a scale 10 times greater than the simulated values. Predicted values in the living room and dining room were similar to eachother but less so to the experimental values. Basement pressures displayed similar values that trended within 1 Pa of ambient on average.

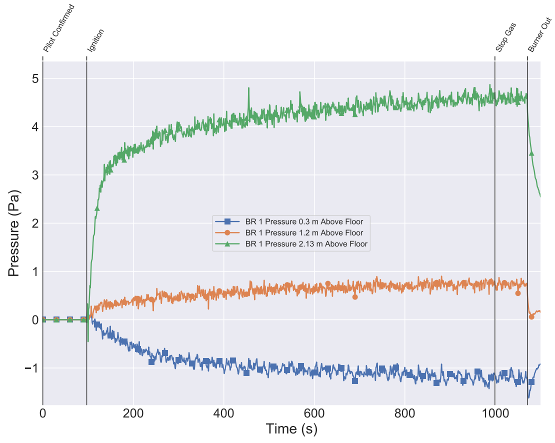

(a) Bedroom 1 Pressures

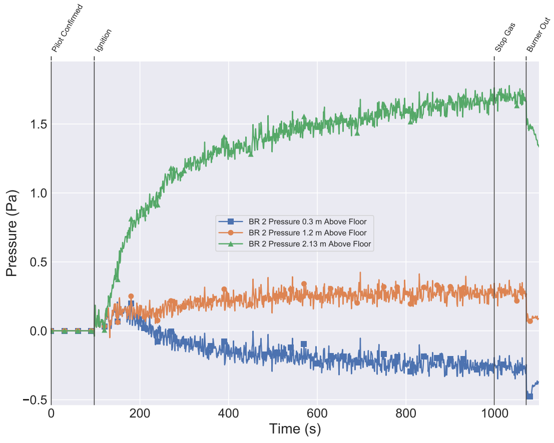

(b) Bedroom 2 Pressures

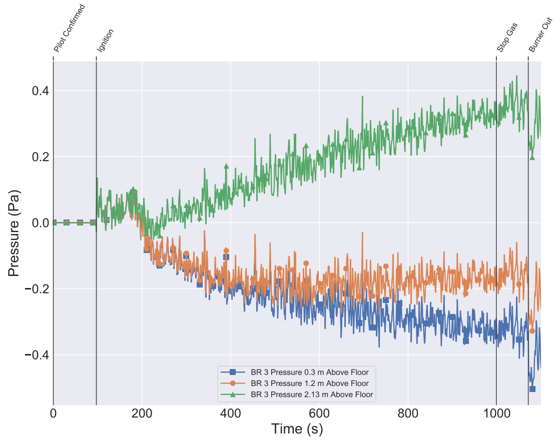

(c) Bedroom 3 Pressures

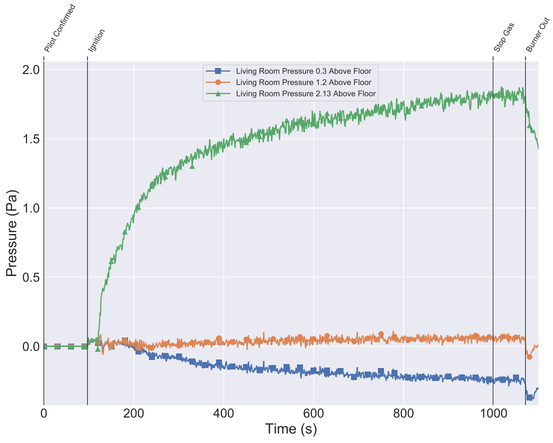

(d) Living Room Pressures

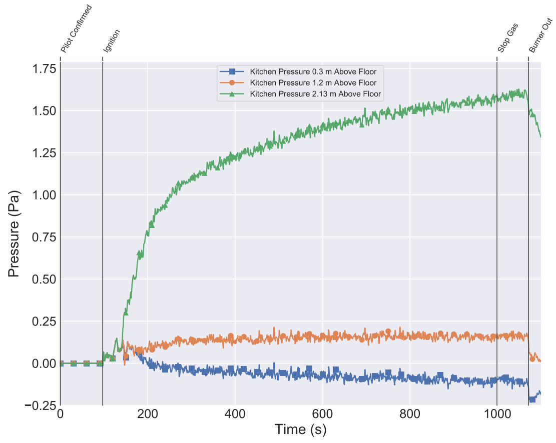

(e) Kitchen Pressures

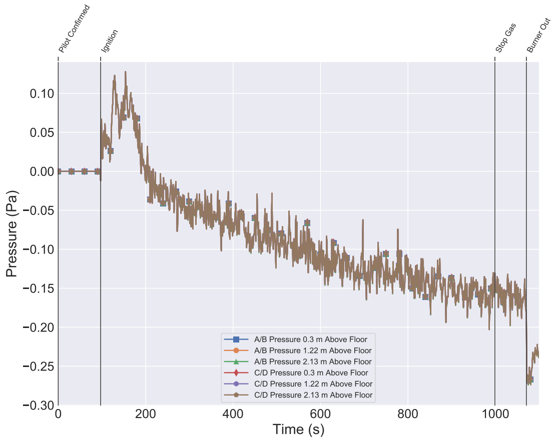

(f) Basement Pressures

Figure 6.8: FDS Experiment 12 Pressures

The concentrations of oxygen and carbon dioxide are provided for the three bedrooms and the basement in Figure 6.9. Water vapor concentrations are included for bedroom 2, bedroom 3 and the basement. Predicted oxygen concentrations in bedroom 1 remained constant approximately 1% less than the experimental value. Oxygen concentrations in bedroom 2, bedroom 3 and the basement were similar between the experimental and simulated values. Water vapor data in bedroom 2 and the basement were similar between the experimental and simulated data sets. Water vapor concentrations in bedroom 3 were slightly greater in the simulation than in the experimental data.

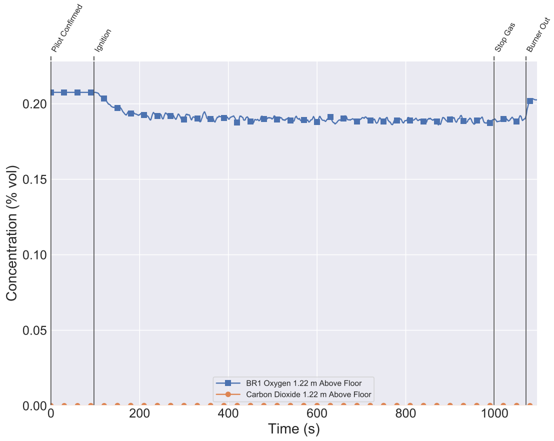

(a) Bedroom 1 Gases

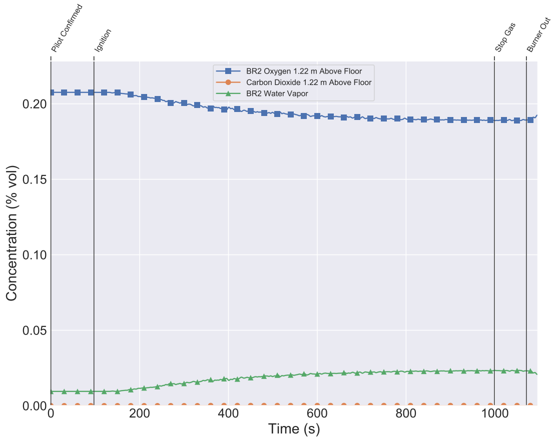

(b) Bedroom 2 Gases

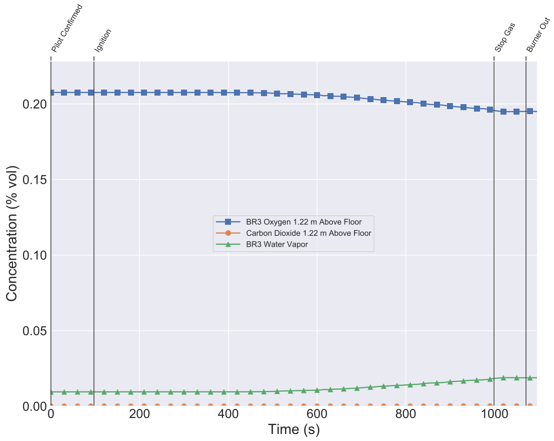

(c) Bedroom 3 Gases

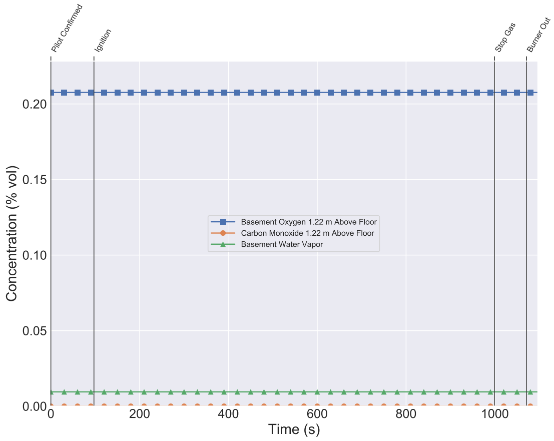

(d) Basement Gases

Figure 6.9: FDS Experiment 12 Gases

Figure 6.10 shows the temperatures of the supply and return vents in the structure. Supply vent temperatures in bedrooms 1 and 2 were similar between experimental and simulated data because of convective heating being the primary route of heat transfer through the open doorways. Simulated supply vent temperatures in bedroom 3 increased quicker and to a greater value than the experimental data. This is attributed to the lack of heat loss through the HVAC ductwork. Return

vent temperatures were similar to the experimental data.

Figure 6.10(b) shows the rest of the vents on the first floor. Living room supply vent temperatures were similar between the experimental and simulated data. Supply vent temperatures for the dining room and the kitchen were greatly overestimated due to convective heat transfer through the HVAC supply system. Return vent temperatures behaved similar to the experimental data and room temperatures at their respective heights. Simulated vent temperatures in the basement showed no change, similar to the experimental data.

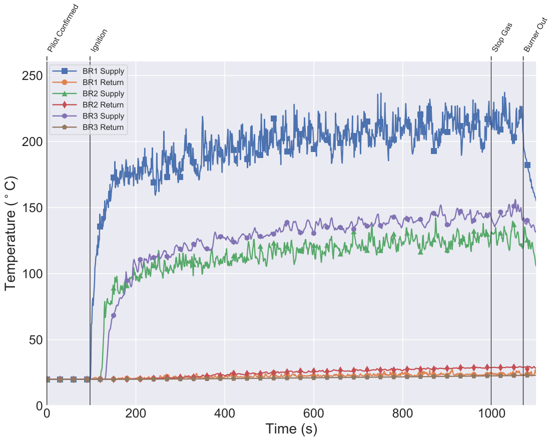

(a) Bedroom Vent Temperatures

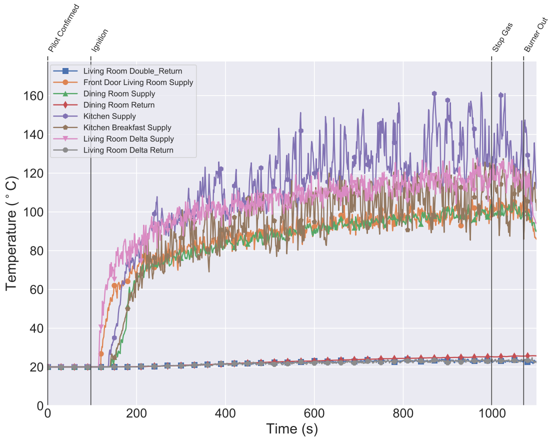

(b) First Floor Vent Temperatures

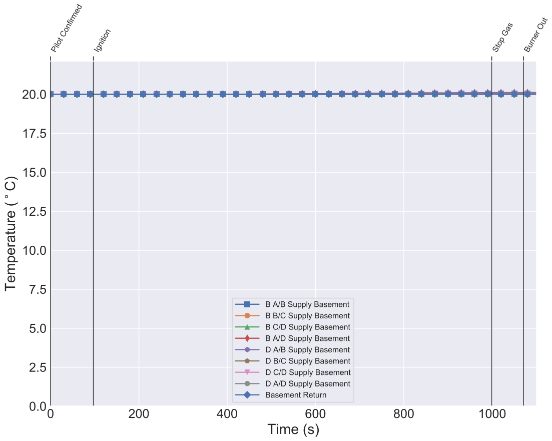

(c) Basement Vent Temperatures

Figure 6.10: FDS Experiment 12 Vent Temperatures

The temperature and velocity within the supply duct are provided in Figure 6.11. Simulated temperatures in the supply duct increased to a similar temperature as the experimental data but had a more rapid increase. Simulated velocity in the supply duct remained steady at approximately 0 m/s.

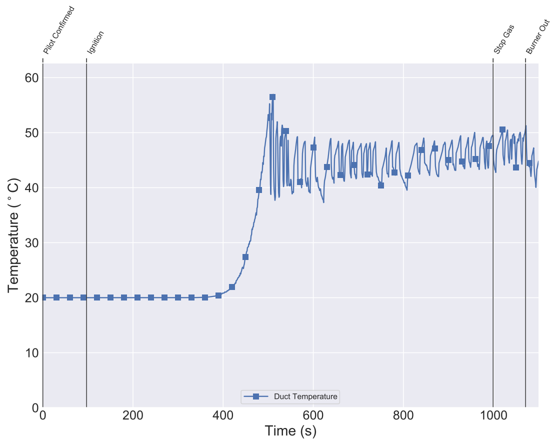

(a) Supply Duct Temperature

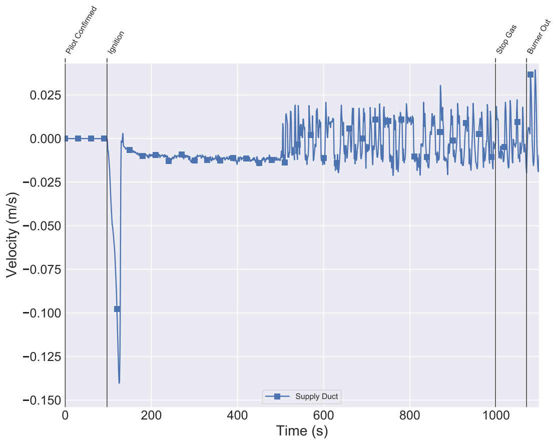

(b) Supply Duct Velocity

Figure 6.11: FDS Experiment 12 Supply Duct Temperature and Velocity

Table 6.2 below shows a comparison of fire room data and bedroom 3 data collected from experimental and simulated data. The data points identified below show a wider difference for the specified locations as compared to simulation 1. The bedroom 3 ceiling temperature was exceeded by over 54%, or approximately 21 °C. The greatest differential occurring here is expected based on heat transfer through the ductwork.

Table 6.2: Experiment 12 Comparisons

Description	Experimental Value	FDS Value	Difference
Max Fire Room Ceiling Temperature	252.0	252.0	0.0%
Max Fire Room Floor Temperature	70.6	56.4	-20.1%
Min Fire Room O2	19.6%	18.3%	-6.6%
Max BR3 Ceiling Temperature	37.7	58.2	54.4%
Max BR3 Floor Temperature	20.5	23.3	13.7%
Min BR3 O2	19.6%	19.5%	-0.5%

6.2.3 Experiment 15

The time versus temperature graphs from the three bedrooms, the living room, the dining room and the kitchen are provided in Figure 6.12. The simulated temperatures in bedroom 1 from this experiment increased by only 30 °C, in contrast to the experimental increase of approximately 40 °C. Temperature increase at the 0.3 m height is similar between the simulated and experimental data. For this experiment, it is probable that there was extra heat leakage into bedroom 1 that was not included in the model. Sources of this heat leakage could have been from around the closed door to bedroom 1 or through the common space wall that the gas line was fed into the living room through.

Analysis of the bedroom 2 data shows similar trends between the simulated and experimental data. The maximum temperatures at the ceiling and the floor were approximately 20 °C higher for the simulated data than what was observed in the experimental data.

As was the case in previous simulated experiments, the temperature in bedroom 3 was higher than the experimental data. Simulated temperature increase in bedroom 3 increased at a greater rate and with a shorter delay following ignition than was observed in the experimental data. Temperatures at the ceiling in bedroom 3 were approximately 15 °C greater in the simulation than recorded during the experiment. Temperatures near the floor displayed a similar trend.

Temperatures in the living room were similar between the simulation and the experiment, with simulated temperatures at the ceiling exceeding the experimental

temperatures by approximately 25 °C. Simulated temperatures at the floor exceeded the experimental data by by approximately 15 °C. Simulated temperatures in the kitchen and dining room exceeded the experimental data by approximately 10 °C at the ceiling and approximately 20 °C at the floor.

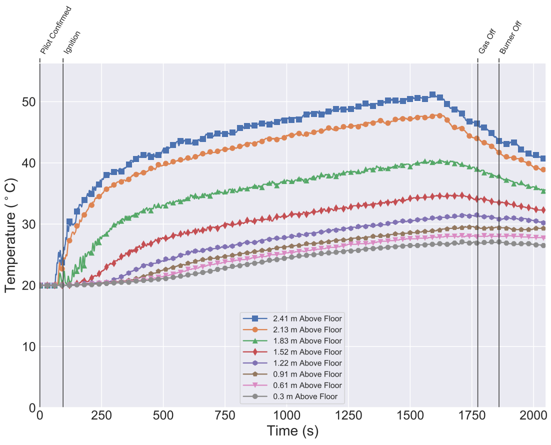

(a) Bedroom 1 Temperatures

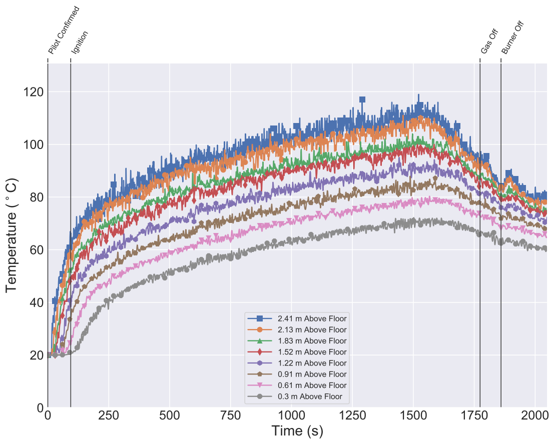

(b) Bedroom 2 Temperatures

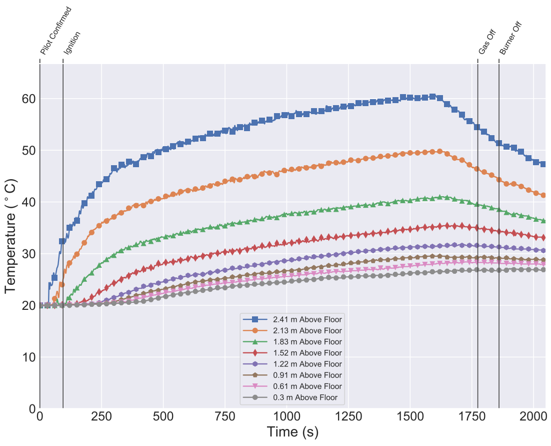

(c) Bedroom 3 Temperatures

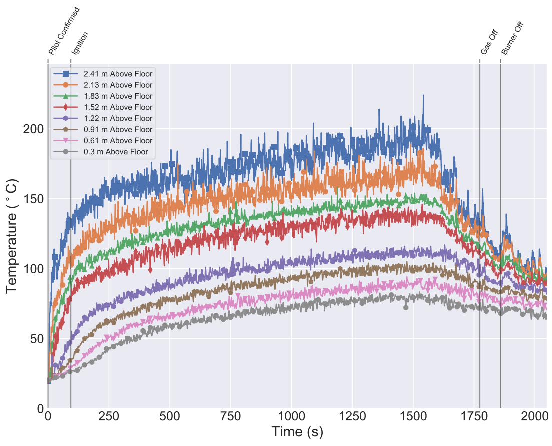

(d) Living Room Temperatures

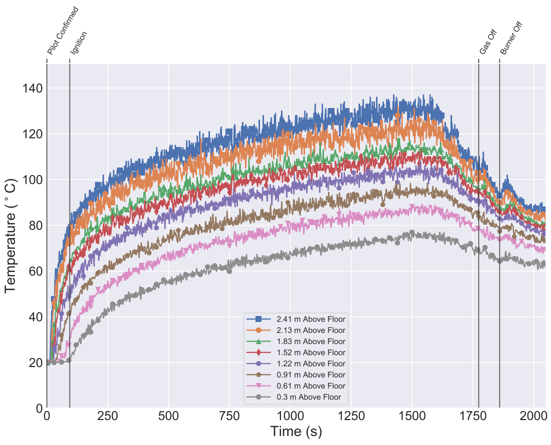

(e) Dining Room Temperatures

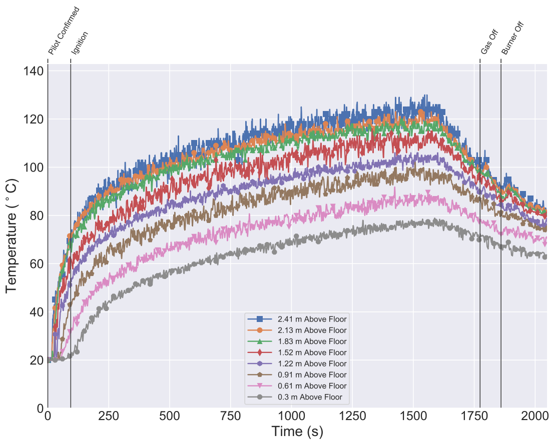

(f) Kitchen Temperatures

Figure 6.12: FDS Experiment 15 First Floor Temperatures

The time versus temperature graphs for the four basement quadrants are provided in Figure 6.13. From the figures it can be seen that with a simulated fire in the living room, there was minimal temperature increase measured in the basement.

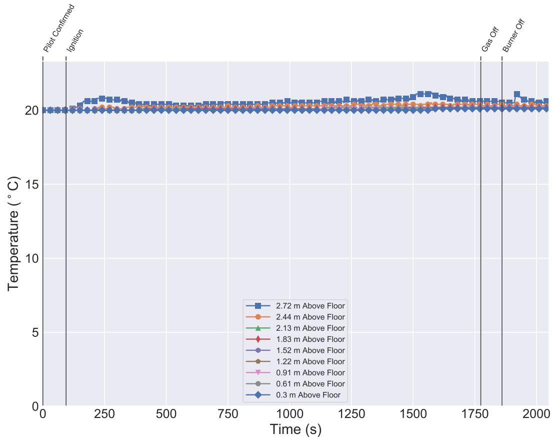

(a) Basement A Quadrant Temperatures

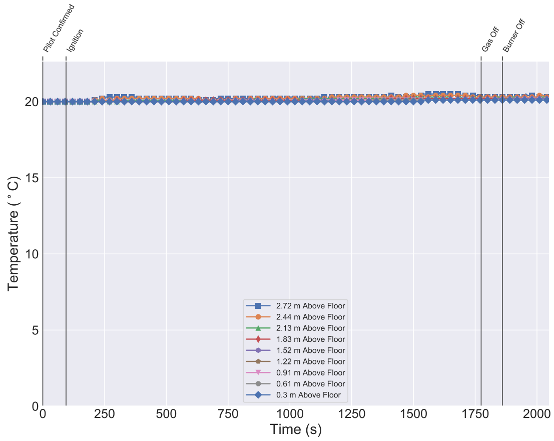

(b) Basement B Quadrant Temperatures

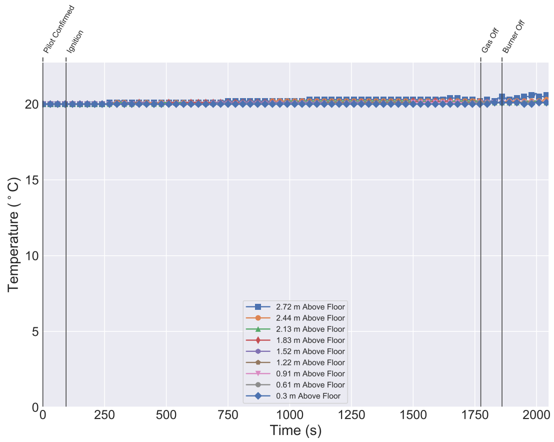

(c) Basement C Quadrant Temperatures

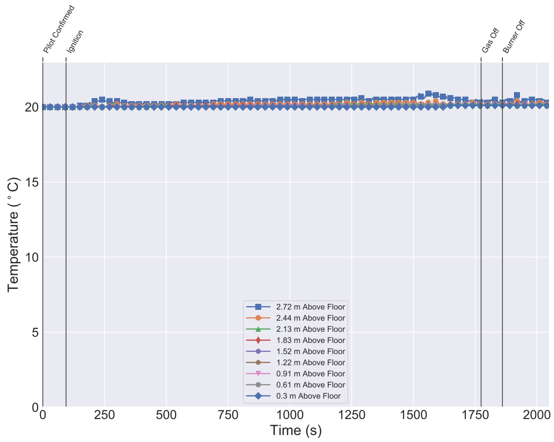

(d) Basement D Quadrant Temperatures

Figure 6.13: FDS Experiment 15 Basement Temperatures

The pressures within each room are shown in Figure 6.14. The pressure charts generated by the simulation vary greatly compared to the experimental results. In the simulation, at all locations following ignition there was a pressure spike of ap-

proximately 50 Pa. Following the pressure spike, pressures all returned to lower readings. Pressures in bedroom 1 following the spike leveled out with steady pressures approximately equal with what was recorded in the experiment at the 2.13 m height. Simulated pressures in bedroom 2 displayed a similar stratification as that recorded in the experiment but a few pascals higher at each height. Simulated pressures in bedroom 3 were steady at a similar pressure to that recorded in the experiment. Simulated living room and kitchen pressures both showed distinct stratification with consistent pressures a few pascals above the experimental data. Simulated pressures in the basement remained steady at a few pascals above the ambient and above the experimental data.

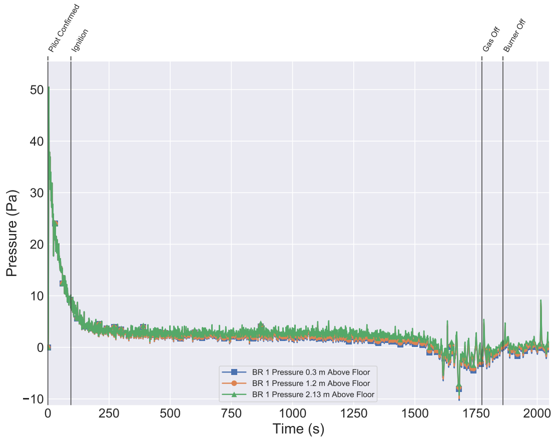

(a) Bedroom 1 Pressures

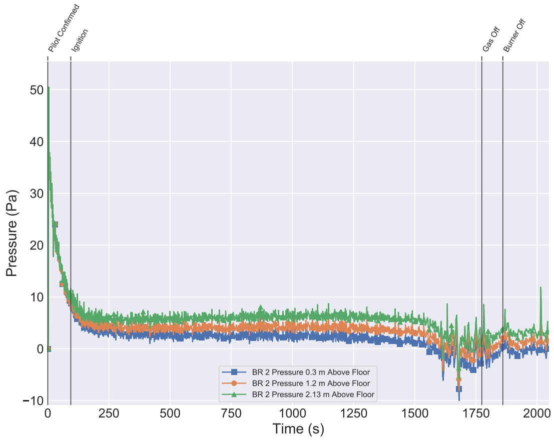

(b) Bedroom 2 Pressures

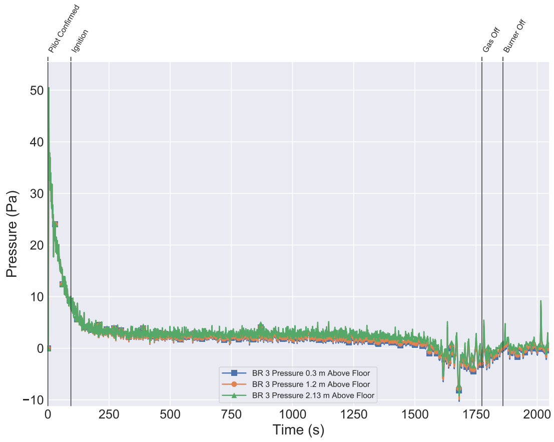

(c) Bedroom 3 Pressures

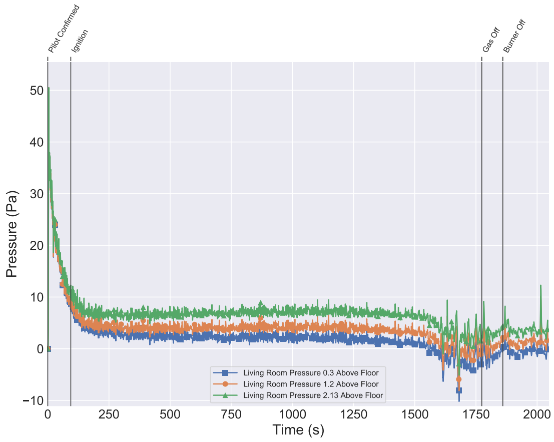

(d) Living Room Pressures

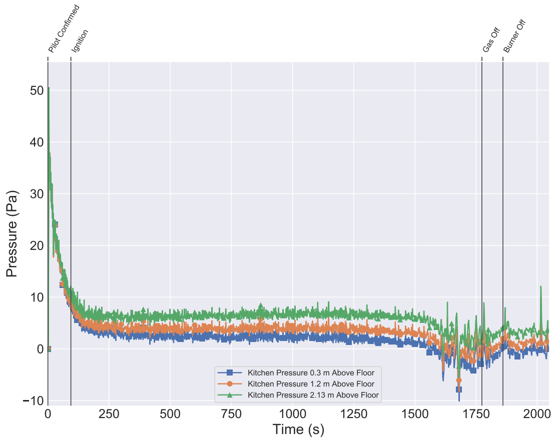

(e) Kitchen Pressures

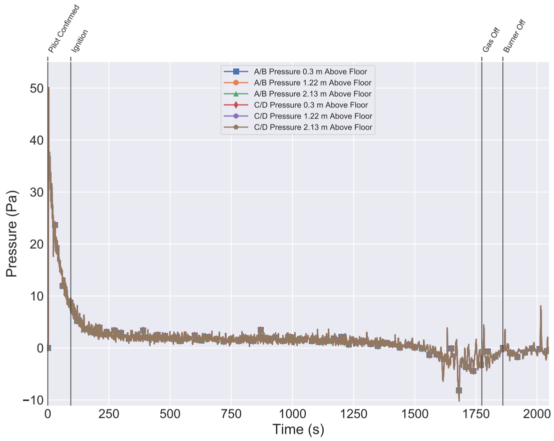

(f) Basement Pressures

Figure 6.14: FDS Experiment 15 Pressures

The concentrations of oxygen and carbon dioxide are provided for the three bedrooms and the basement in Figure 6.15. Water vapor concentrations are included for bedroom 2, bedroom 3 and the basement. A quick comparison of the simulated versus experimental living room gas concentrations shows that there was a smaller supply of oxygen available for combustion in the simulated experiment. The simulated oxygen concentration dropped to approximately 13% while the experimental oxygen concentration barely dropped below 20% during the experiment. It is likely that there was more leakage into the structure than was accounted for in the modeling setup. Comparison of the slope of simulated oxygen decrease in bedroom 2 is similar to the slope of the experimental oxygen decrease in bedroom 2. The simulated slope is slightly greater than the experimental slope with the final simulated oxygen concentration approaching 13%. Water vapor in bedroom 2 increased more during the simulated experiment by approximately 2%. Oxygen concentrations in bedroom 3 decreased more in the simulated experiment by approximately 2%, while water concentrations in bedroom 3 increased by approximately 3% more in the simulated experiment. Basement gas concentrations did not differ between simulated data and experimental data.

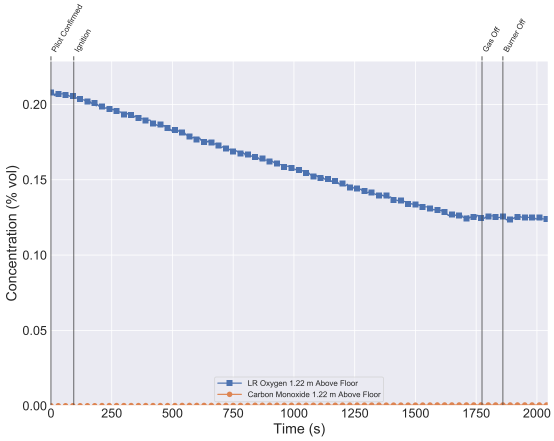

(a) Living Room Gases

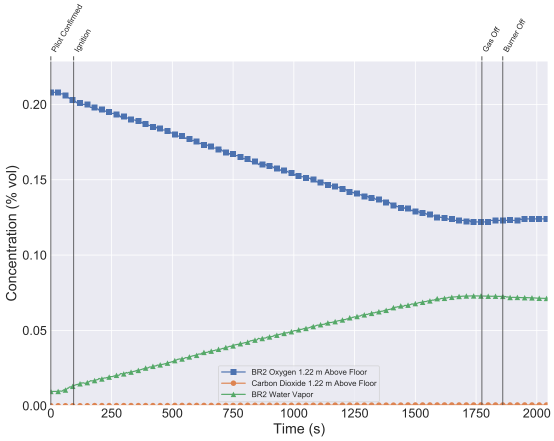

(b) Bedroom 2 Gases

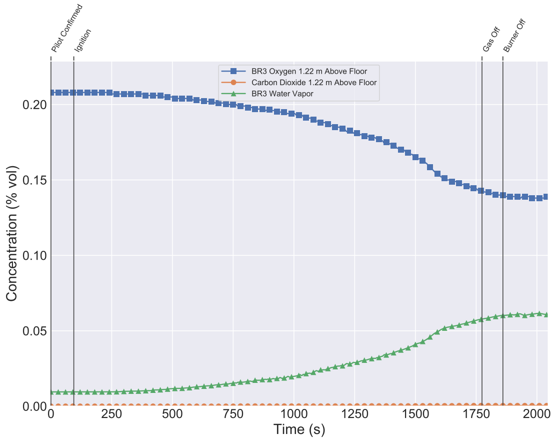

(c) Bedroom 3 Gases

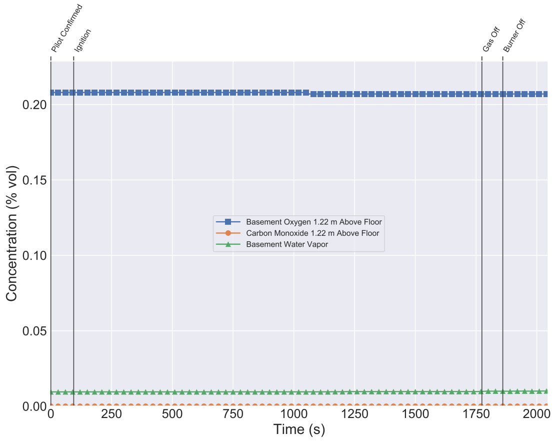

(d) Basement Gases

Figure 6.15: FDS Experiment 15 Gases

Figure 6.16 shows the temperatures of the supply and return vents in the structure. From Figure 6.16(a) it can be seen that the bedroom 2 and 3 supply vent temperatures increased more during the simulation by approximately 40 °C. The slope of increase in bedroom 2 was simillar between the simulation and experimental data. The simulated return vent temperature for bedroom 1 increased at a rate similar to bedroom 3, while the experimental bedroom 1 vent increased at a greater

rate and reached a temperature approximately 20 °C greater than the simulated temperature. The simulated bedroom 2 return vent temperature increased at a greater rate than the experimental data and reached a temperature approximately 40 °C greater than the experimental data. The bedroom 3 return vent behaved similar between the simulated data and the experimental data.

Figure 6.16(b) shows the rest of the vents on the first floor. The living room supply vents behaved similar between the simulated and the experimental data because they were being affected directly by the gas burner. The kitchen supply and the dining room supply also behaved similar between the simulation and the experiment. The simulation return vent temperatures exceeded the experimental values by approximately 25 °C. The only supply vent temperature that does not agree between the simulation and the experiment is that of the kitchen breakfast supply vent. The experimental data had the breakfast supply vent following a similar trend to the data observed from the return vents. The simulated data had the breakfast supply vent display similar trends to the kitchen supply and dining room supply vents. Comparison of basement vent temperatures yields similar agreement between the simulated and experimental data.

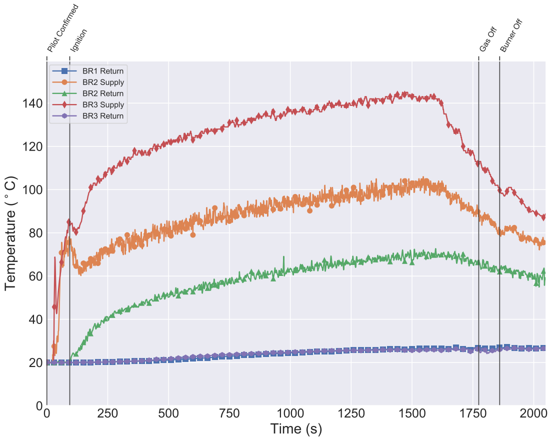

(a) Bedroom Vent Temperatures

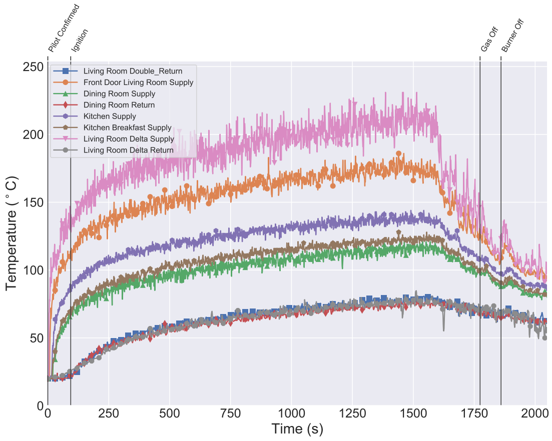

(b) First Floor Vent Temperatures

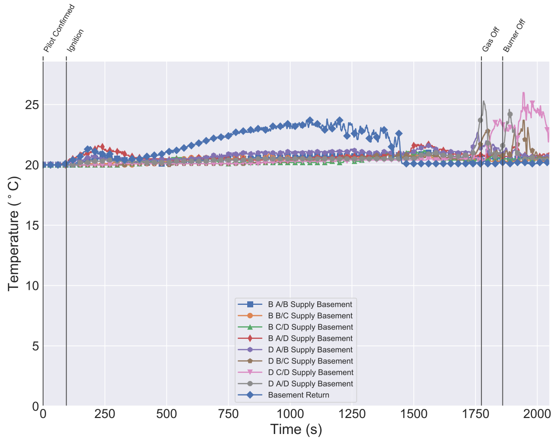

(c) Basement Vent Temperatures

Figure 6.16: FDS Experiment 15 Vent Temperatures

The temperature and velocity within the supply duct are provided in Figure 6.17. The simulated supply duct temperature varies widely with several 60 °C variations. The maximum simulated supply duct temperature exceeds the experimental data by approximately 60 °C. The simulated supply duct velocity holds constant at 0 m/s for the experiment while the experimental velocity varies with an average above 0 m/s.

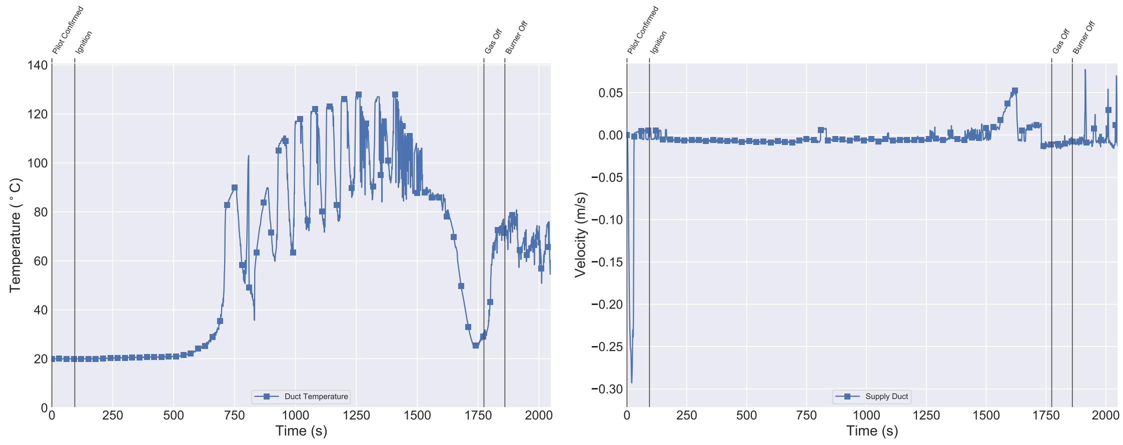

(a) Supply Duct Temperature

(b) Supply Duct Velocity

Figure 6.17: FDS Experiment 15 Supply Duct Temperature and Velocity

Table 6.3 below shows a comparison of fire room data and bedroom 3 data collected from experimental and simulated data. Greater discrepancies were noted in the living room experiment as compared to the bedroom simulations. Simulated data overestimated the temperatures reached in the fire room by almost 30% and the ceiling temperature in bedroom 3 by over 33%. The overestimation of oxygen loss within the structure is important to note. The over consumption could be due to additional leaks being present in the experimental structure that were not accounted for in the simulation model.

Table 6.3: Experiment 15 Comparisons

Description	Experimental Value	FDS Value	Difference
Max Fire Room Ceiling Temperature	173.0	224.0	29.5%
Max Fire Room Floor Temperature	67.1	83.5	24.4%
Min Fire Room O2	19.0%	12.3%	-33.9%
Max BR3 Ceiling Temperature	45.4	60.7	33.7%
Max BR3 Floor Temperature	27.3	27.1	-0.7%
Min BR3 O2	17.0%	13.8%	-20.7%

6.2.4 Experiment 16

The time versus temperature graphs from the three bedrooms, the living room, the dining room and the kitchen are provided in Figure 6.18. Temperatures in bedroom 1 were less in the simulated data than in the experimental data by approximately 20 °C at most heights. This trend differs from those identified in other simulated experiments where the primary source of heat transfer into bedroom 1 should theoretically be through the HVAC supply system. It is possible that greater experimental heat transfer into bedroom 1 could be due to leaks through a common wall or doorway. Simulated temperatures in bedroom 2, the living room, the dining room and the kitchen were similar to the experimental data collected. Simulated temperatures in bedroom 3 were greater than the experimental temperatures by approximately 15 °C at the ceiling.

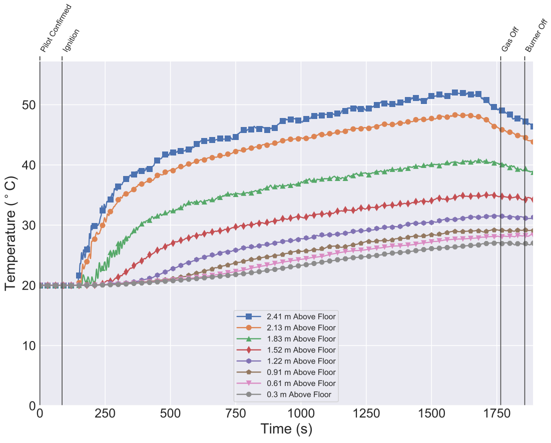

(a) Bedroom 1 Temperatures

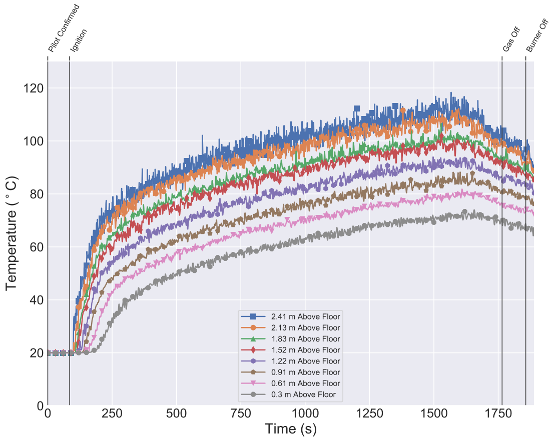

(b) Bedroom 2 Temperatures

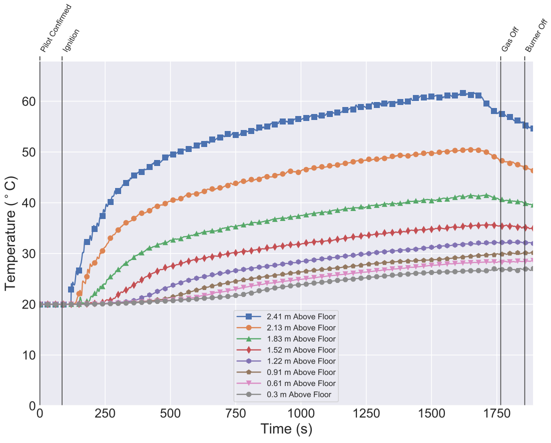

(c) Bedroom 3 Temperatures

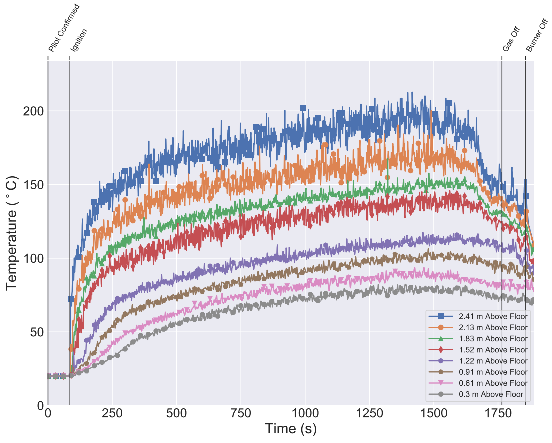

(d) Living Room Temperatures

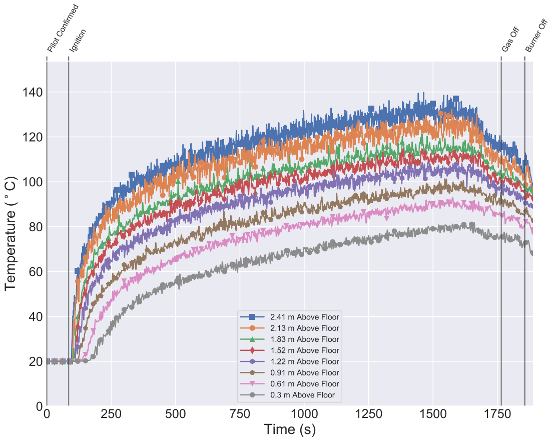

(e) Dining Room Temperatures

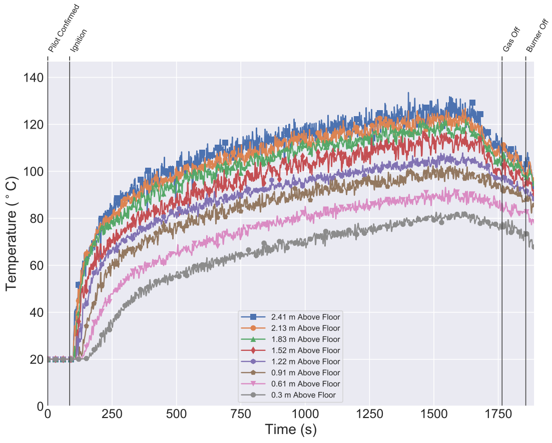

(f) Kitchen Temperatures

Figure 6.18: FDS Experiment 16 First Floor Temperatures

The time versus temperature graphs for the four basement quadrants are provided in Figure 6.19. Minimal temperature changes were noted in the simulated data, similar to the experimental data.

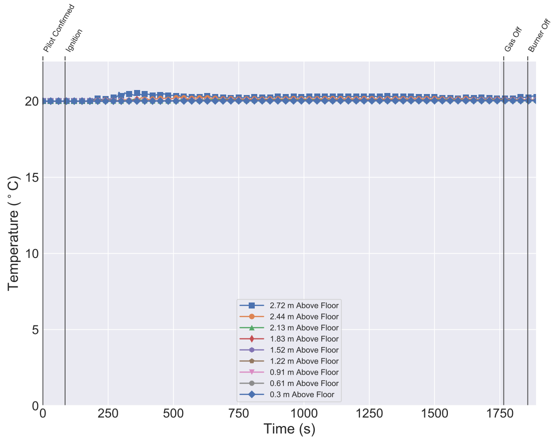

(a) Basement A Quadrant Temperatures

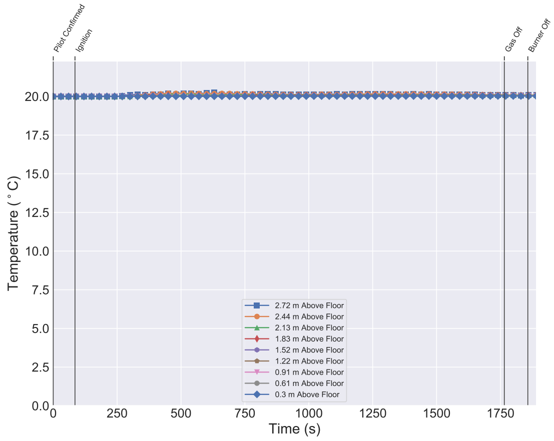

(b) Basement B Quadrant Temperatures

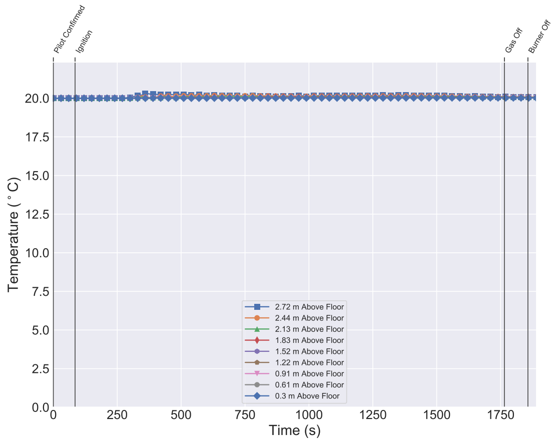

(c) Basement C Quadrant Temperatures

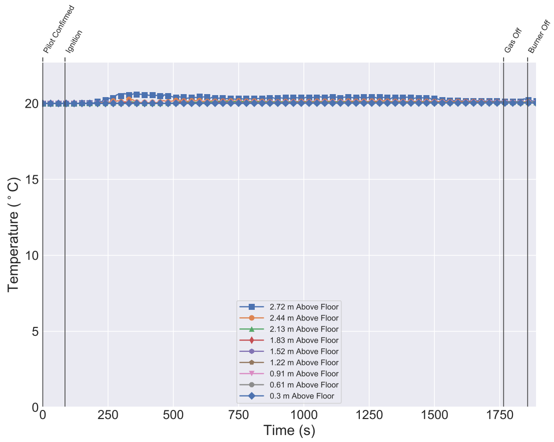

(d) Basement D Quadrant Temperatures

Figure 6.19: FDS Experiment 16 Basement Temperatures

The pressures within each room are shown in Figure 6.20. Simulated pressures in bedroom 1 were similar to the constant experimental values with a simulated spike that was approximately half of the experimental spike. Bedroom 2 steady pressures

were also similar to the experimental values but with a simulated spike less than a quarter of the experimental spike. Bedroom 3 pressures were similar with the same lower spike as noted in the other bedrooms. Simulated living room and kitchen pressures were slightly greater than the experimental pressures by approximately 2 Pa at all heights. Simulated pressures in the basement remained steady around 0.5 Pa above the ambient while the experimental pressure varied between 1 Pa above and below the ambient.

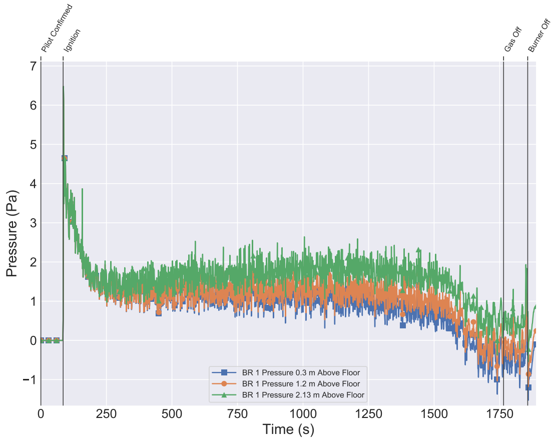

(a) Bedroom 1 Pressures

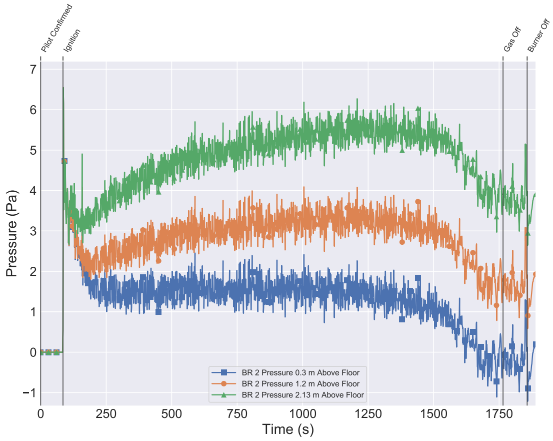

(b) Bedroom 2 Pressures

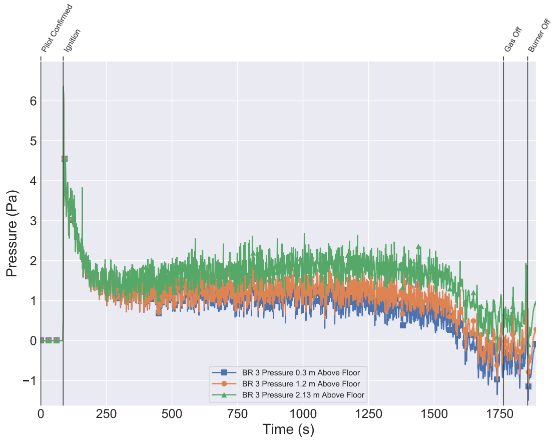

(c) Bedroom 3 Pressures

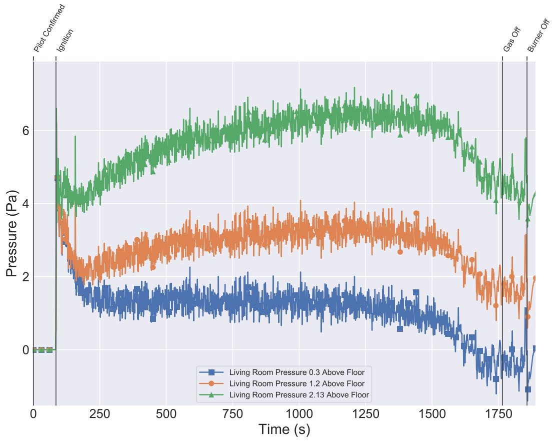

(d) Living Room Pressures

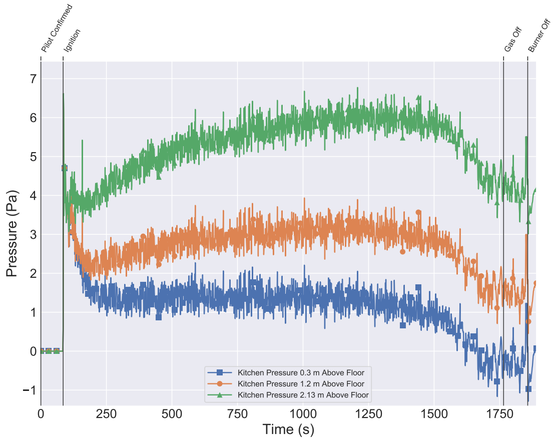

(e) Kitchen Pressures

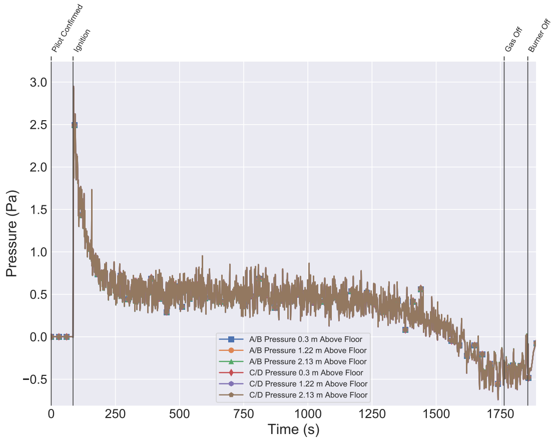

(f) Basement Pressures

Figure 6.20: FDS Experiment 16 Pressures

The concentrations of oxygen and carbon dioxide are provided for the three bedrooms and the basement in Figure 6.21. Water vapor concentrations are included for bedroom 2, bedroom 3 and the basement. Simulated oxygen consumption in bedroom 2 was greater than the experimental data by approximately 4%. Simulated oxygen concentrations in bedroom 3 were lower than the experimental values by approximately 5% at the termination of the experiment. Simulated oxygen concentrations in the living room decreased at a greater rate and to a lower value than the experimental data collected. Oxygen concentrations in the living room decreased to the point that combustion of the propane gas was no longer possible, thus causing the flames to go out in the simulation. Gas concentrations in the basement were similar between the experimental and simulated data. Water vapor concentrations in bedroom 2 and the basement were similar between the simulated and experimental data. Water vapor concentrations in bedroom 3 were greater than the experimental data by approximately 2%.

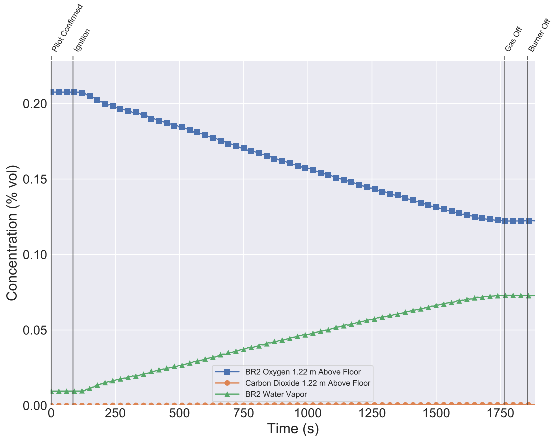

(a) Bedroom 2 Gases

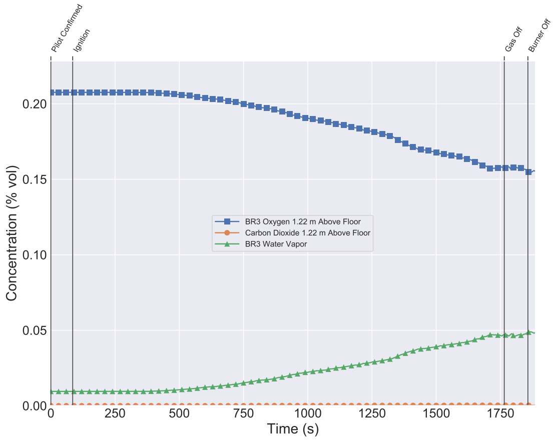

(b) Bedroom 3 Gases

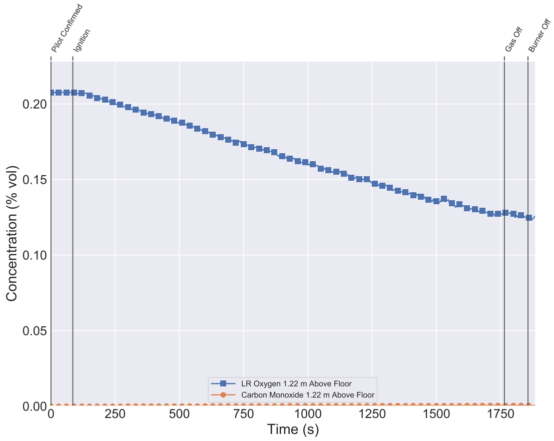

(c) Living Room Gases

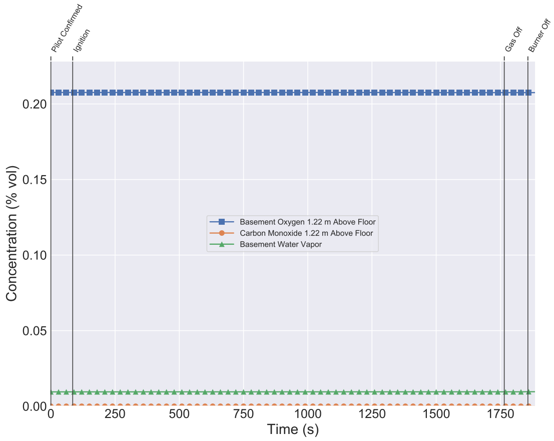

(d) Basement Gases

Figure 6.21: FDS Experiment 16 Gases

Figure 6.22 shows the temperatures of the supply and return vents in the structure. Simulated supply vent temperatures in bedrooms 2 and 3 were greater than the experimental data. The supply vent in bedroom 2 was heated by convection through the air in bedroom 2 while bedroom 3 was heated by convection through the HVAC supply system. Return vent temperatures in the bedrooms were slightly underpredicted by the simulated data.

Figure 6.22(b) shows the rest of the vents on the first floor. Predicted supply vent temperatures were similar between the simulated and experimental data with the exception of the kitchen breakfast supply which was overpredicted by the simulation. Return vent temperatures on the first floor were also overpredicted by the simulated data. In the simulated basement data, the basement return vent experienced a slight temperature spike early in the experiment and then had a slight steady increase further into the experiment. Simulated data shows that heat transfer to the basement was occurring via the return vent. This is different than the experimental data which had a slow but steady increase in several supply vent temperatures in the basement during the experiment.

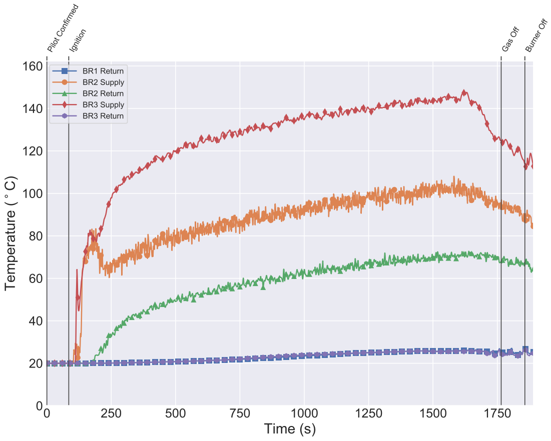

(a) Bedroom Vent Temperatures

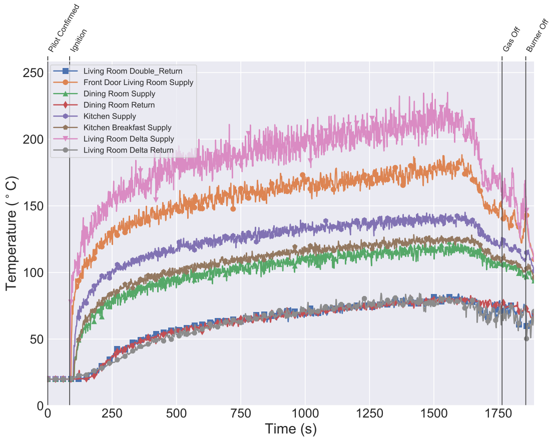

(b) First Floor Vent Temperatures

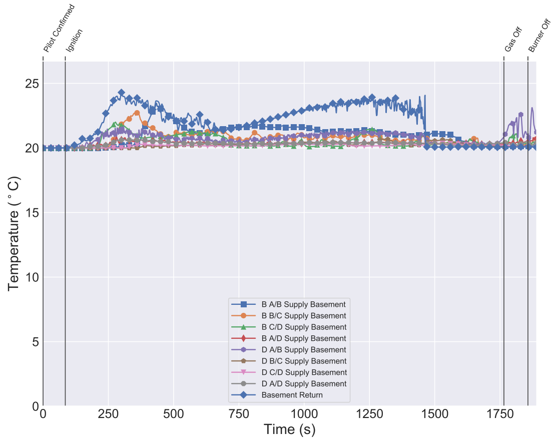

(c) Basement Vent Temperatures

Figure 6.22: FDS Experiment 16 Vent Temperatures

The temperature and velocity within the supply duct are provided in Figure 6.23. Simulated temperatures in the supply duct are rather unsteady and spontaneous as compared to the steady increase of temperature in the experimental data. While the simulated temperature reaches a higher peak value, the short duration of increased heat limits the heat transferred throught the supply duct to the basement in the simulated experiment. Simulated velocity in the supply duct remains steady

at 0 m/s for the majority of the experiment.

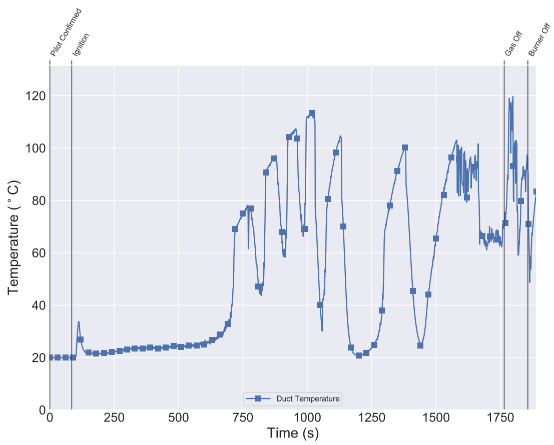

(a) Supply Duct Temperature

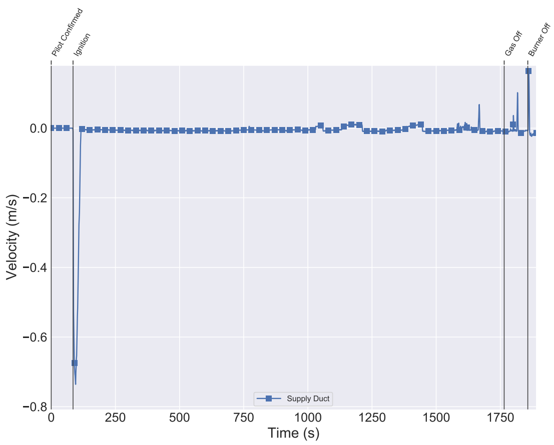

(b) Supply Duct Velocity

Figure 6.23: FDS Experiment 16 Supply Duct Temperature and Velocity

Table 6.4 below shows a comparison of fire room data and bedroom 3 data collected from experimental and simulated data. In comparison to simulated Experiment 15, predicted values did not differ greatly. The temperature at the ceiling in bedroom 3 was over predicted by approximately 37%, or 16 °C higher than the experimental data. Oxygen consumption predictions were still greater than the experimental consumption, but the differential was similar to the previous simulation.

Table 6.4: Experiment 16 Comparisons

Description	Experimental Value	FDS Value	Difference
Max Fire Room Ceiling Temperature	187.0	213.0	13.9%
Max Fire Room Floor Temperature	67.9	82.4	21.4%
Min Fire Room O2	18.4%	12.3%	-33.2%
Max BR3 Ceiling Temperature	45.2	61.8	36.7%
Max BR3 Floor Temperature	27.0	27.1	0.4%
Min BR3 O2	20.7%	15.5%	-25.1%

6.2.5 Experiment 23

The results for the FDS simulation of Experiment 23 are provided in this section. The time versus temperature graphs for the four basement quadrants are provided in Figure 6.24. Comparison of these figures to those of gas burner Experiment 23 show similiar temperature increase trends in all locations but with less temperature stratification. FDS consistently overpredicted temperature increases close to the floor in the basement at all measurement locations, with the exception of the mechanical room. Temperture increases in the mechanical room were greater than simulated by 5 °C to 15 °C.

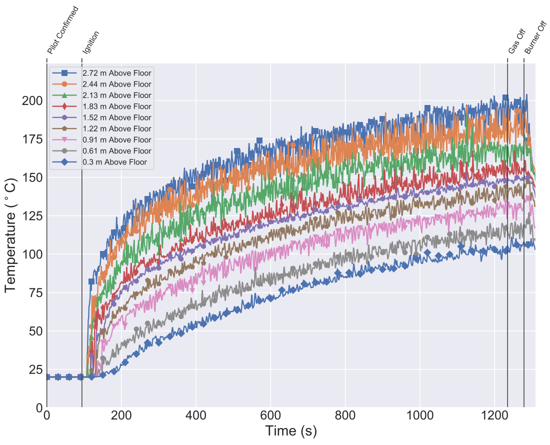

(a) Basement A Quadrant Temperatures

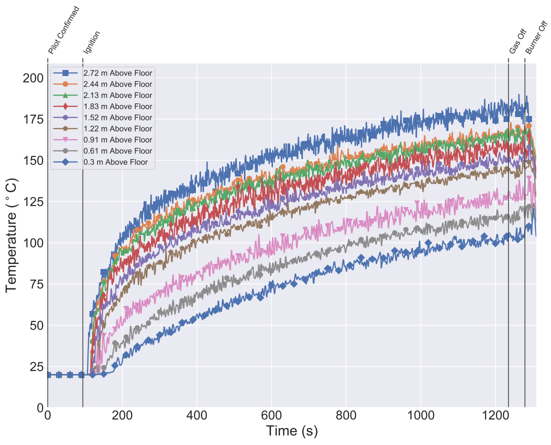

(b) Basement B Quadrant Temperatures

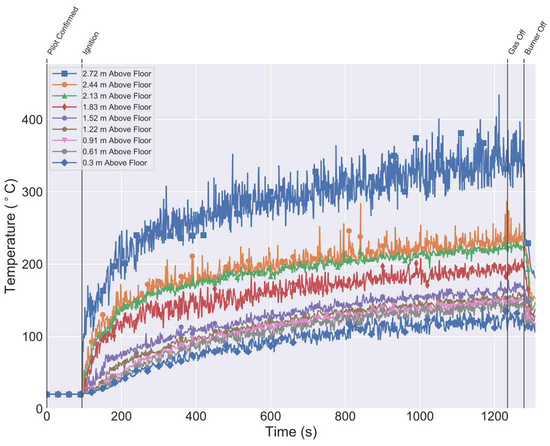

(c) Basement C Quadrant Temperatures

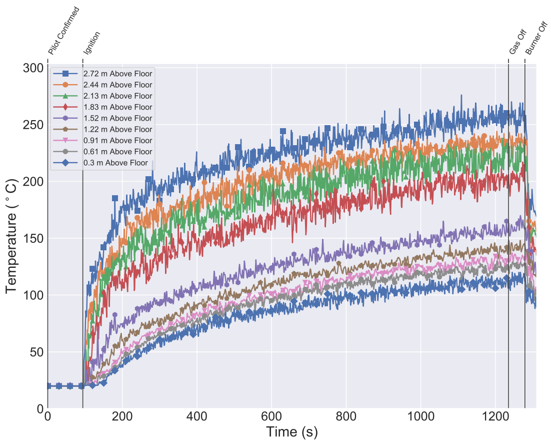

(d) Basement D Quadrant Temperatures

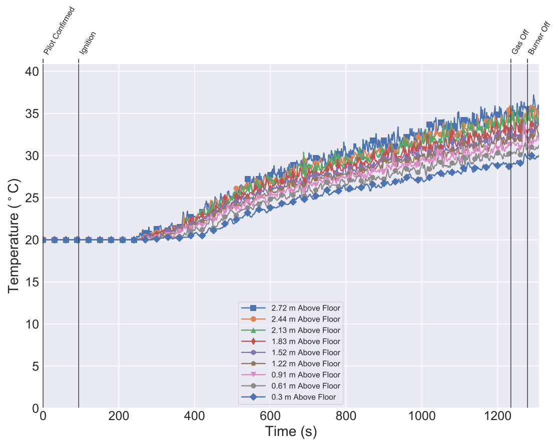

(e) Basement Mechanical Room

Figure 6.24: FDS Experiment 23 Basement Temperatures

The time versus temperature graphs from the three bedrooms, the living room, the dining room and the kitchen are provided in Figure 6.25. Temperatures on the first floor were overpredicted in all measurement locations. Rooms that did not have a closed door between their measurement location and the fire in the basement displayed temperatures that trended closer to their measured values in Experiment 23. Temperatures in bedroom 1 and bedroom 3 showed a quicker and greater initial temperature than those observed in the gas burner experiment. Following the initial temperature spikes, the slope of temperature increase was similar between the gas burner experiment and the FDS simulation. The FDS simulation displayed similar temperature stratification to that seen in the gas burner experiment.

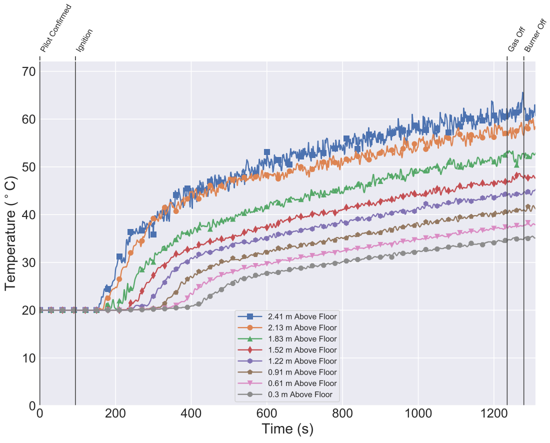

(a) Bedroom 1 Temperatures

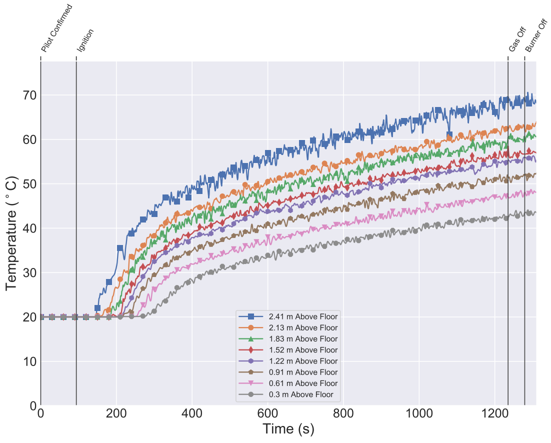

(b) Bedroom 2 Temperatures

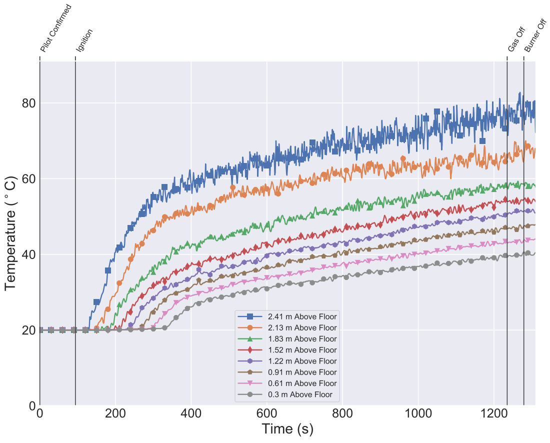

(c) Bedroom 3 Temperatures

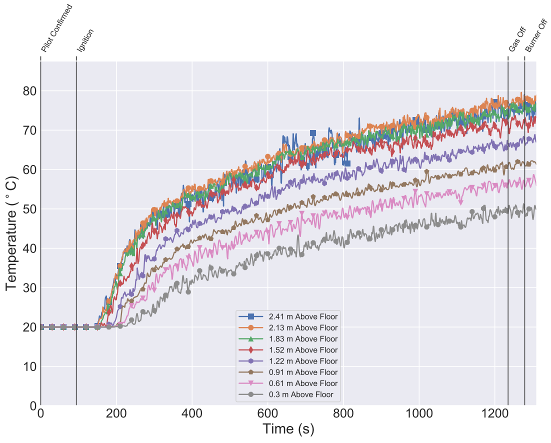

(d) Living Room Temperatures

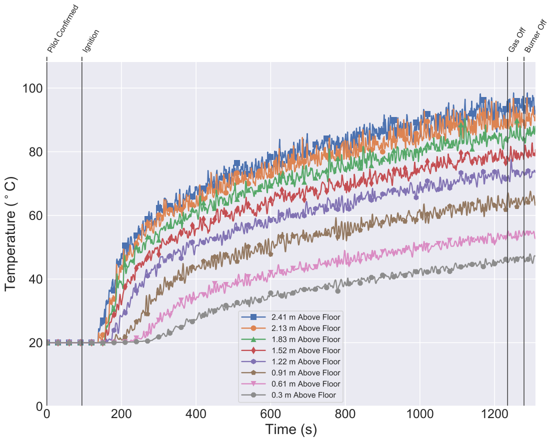

(e) Dining Room Temperatures

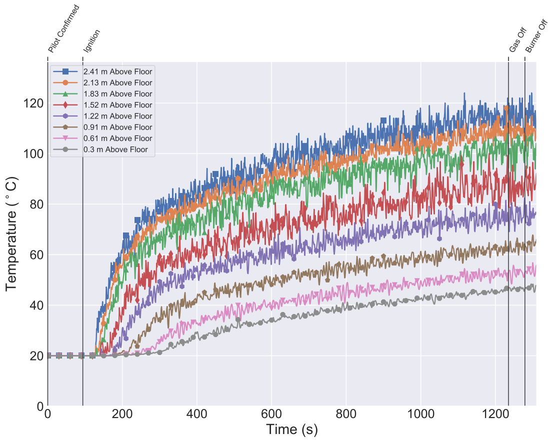

(f) Kitchen Temperatures

Figure 6.25: FDS Experiment 23 First Floor Temperatures

The pressures within each room are shown in Figure 6.26. The pressure charts generated by the simulation vary greatly to those of Experiment 23. FDS pressures spiked a few hundred pascals following ignition and then fluctuated 10s of pascals higher than the experimental data.

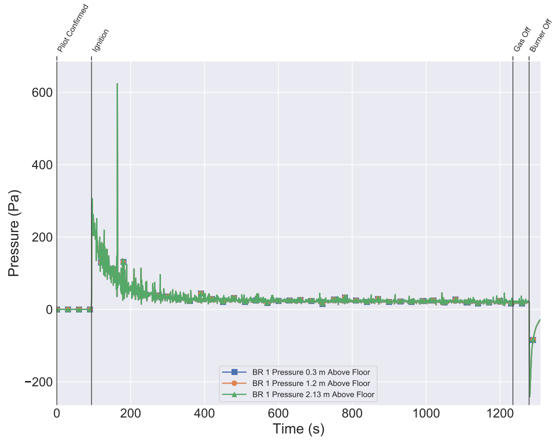

(a) Bedroom 1 Pressures

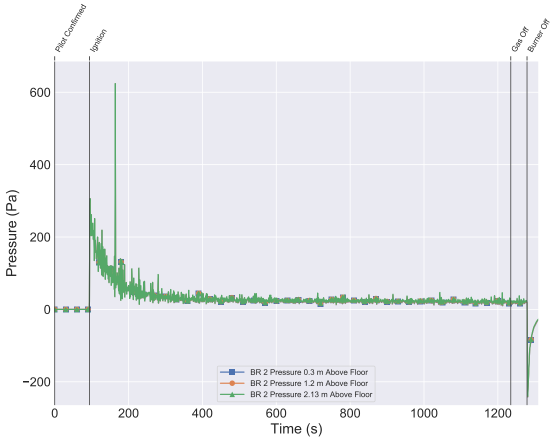

(b) Bedroom 2 Pressures

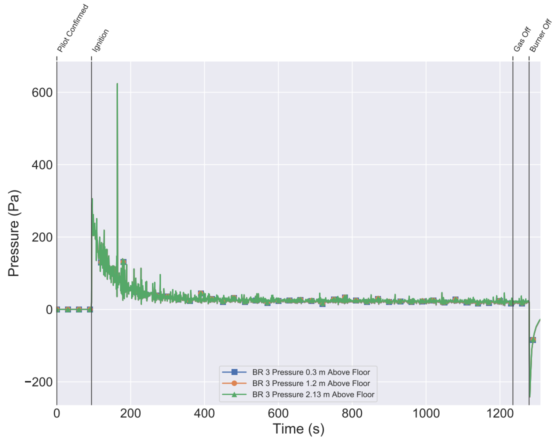

(c) Bedroom 3 Pressures

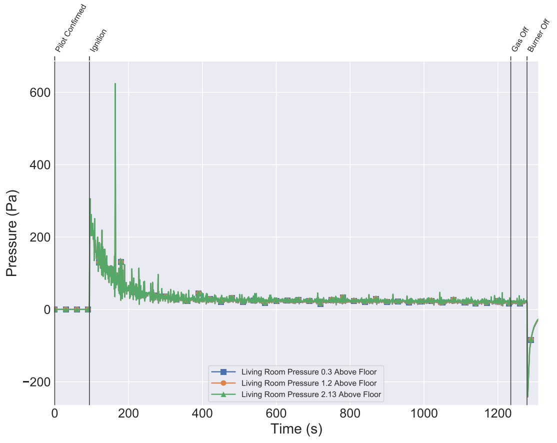

(d) Living Room Pressures

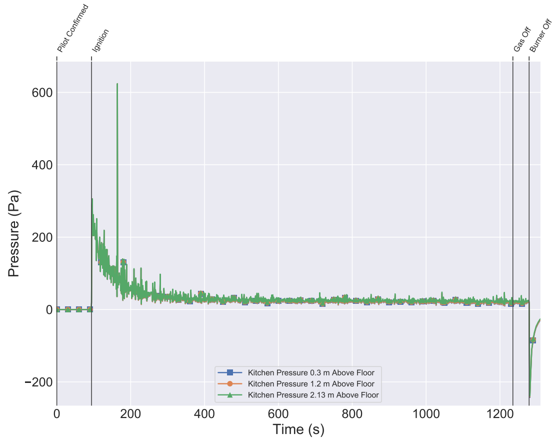

(e) Kitchen Pressures

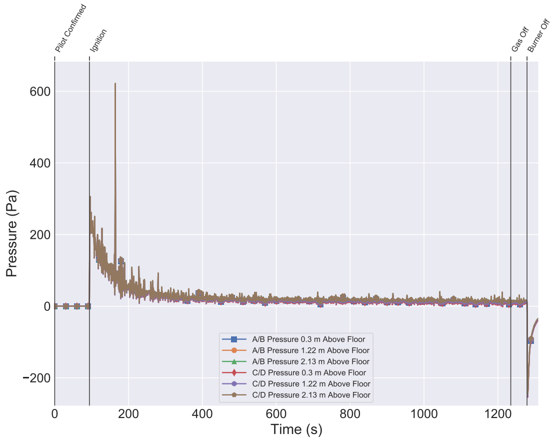

(f) Basement Pressures

Figure 6.26: FDS Experiment 23 Pressures

The concentrations of oxygen and carbon dioxide are provided for the three bedrooms and the basement in Figure 6.27. Water vapor concentrations are included for bedroom 2, bedroom 3 and the basement. Losses in oxygen concentrations are overpredicted in FDS by varying degrees depending on the room. In bedrooms 1 and 2, the predicted drop of oxygen begins earlier than recorded in the experiments which results in a greater loss over time on a similar slope. Experimental oxygen losses in bedroom 3 were minimal while FDS predicted a decrease of nearly 6%. Greater losses were predicted in the basement by nearly 3% over the course of the experimental timeframe. Water vapor trends were similar in bedroom 2 and the basement and were overpredicted in bedroom 3.

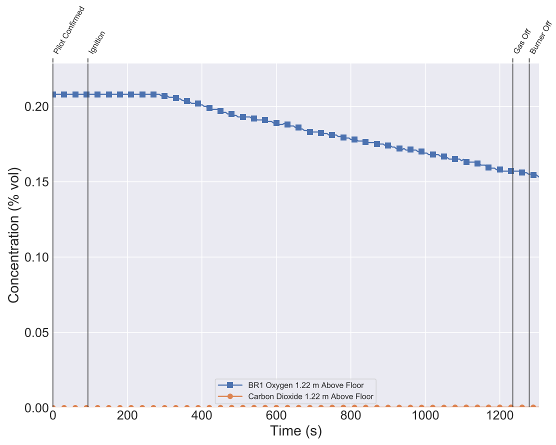

(a) Bedroom 1 Gases

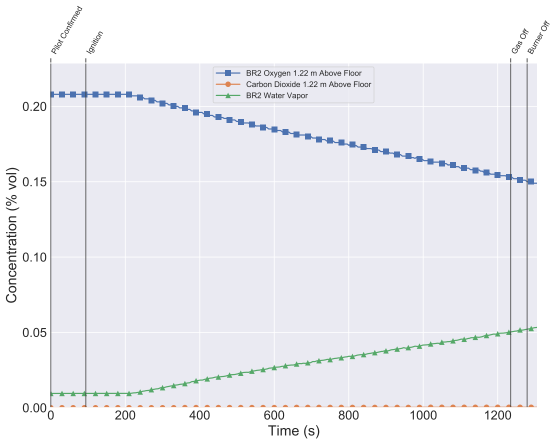

(b) Bedroom 2 Gases

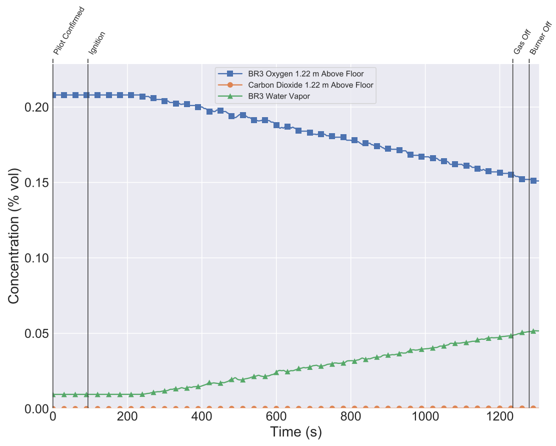

(c) Bedroom 3 Gases

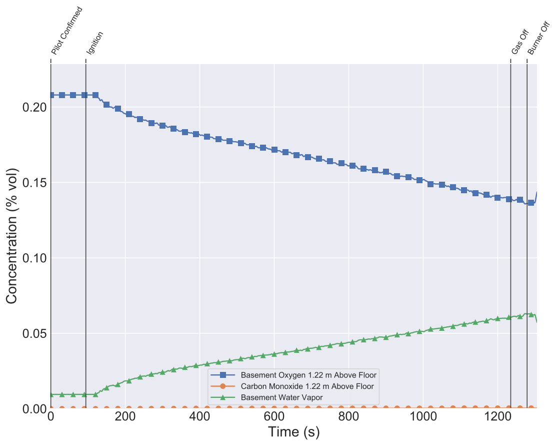

(d) Basement Gases

Figure 6.27: FDS Experiment 23 Gases

Figure 6.28 shows the temperatures of the supply and return vents in the structure. As noted in previous sections, the FDS simulation drastically overpredicts the heat transfer through the HVAC system. The predicted supply vent temperatures greatly exceed those recorded at all first floor locations, with the exception of the kitchen supply which is primarily heated by convection up the open staircase. Predicted return vent temperatures on the first floor trend similarly with the exper-

imental data. Basement vent temperatures trend similarly between FDS and the experimental data due to convective heating across the basement being the primary source of heat transfer to these locations.

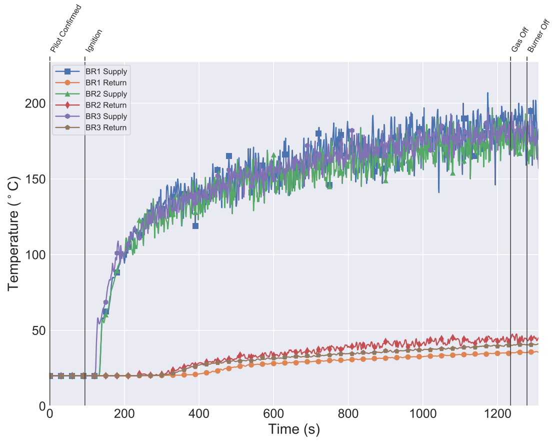

(a) Bedroom Vent Temperatures

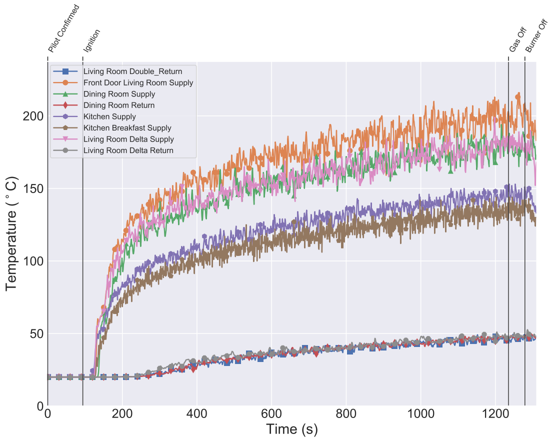

(b) First Floor Vent Temperatures

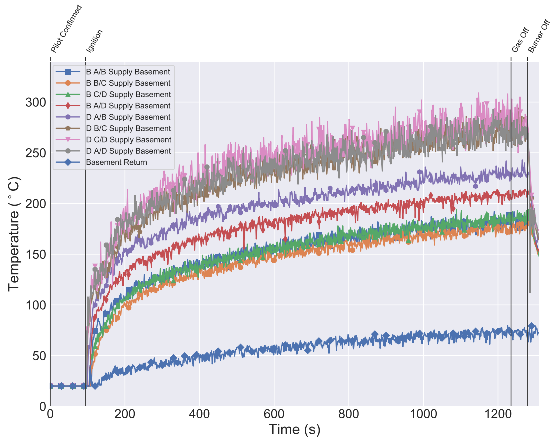

(c) Basement Vent Temperatures

Figure 6.28: FDS Experiment 23 Vent Temperatures

The temperature and velocity within the supply duct are provided in Figure 6.29. Predicted temperatures in the supply duct far exceed the experimental data, but the predicted velocity is similar to that recorded. The constant positive

velocity indicates a continuous flow of heat from the basement to the first floor.

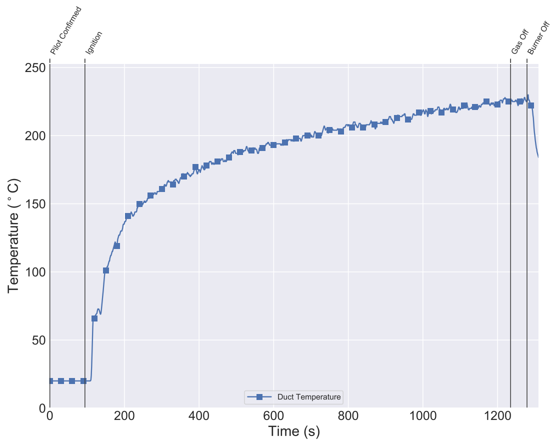

(a) Supply Duct Temperature

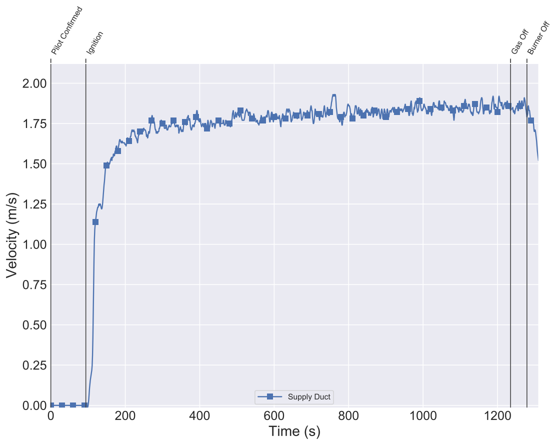

(b) Supply Duct Velocity

Figure 6.29: FDS Experiment 23 Supply Duct Temperature and Velocity

Table 6.5 below shows a comparison of fire room data and bedroom 3 data collected from experimental and simulated data. Differentials between simulated temperatures and experimental data was greater for this basement experiment than for the previous living room experiments, consideribly so at the lowest temperature heights. Predicted fire room temperatures exceeded the experimental data by approximately 50 °C at the ceiling and the floor, resulting in almost a 70% over prediction at the floor level. Bedroom 3 temperature predictions also exceeded the experimental values by a considerable percentage. The bedroom 3 ceiling temperature was over predicted by almost 75%, approximately 35 °C greater than the experimental value. Greater oxygen consumption was also noted during this experiment in the basement and in bedroom 3.

Table 6.5: Experiment 23 Comparisons

Description	Experimental Value	FDS Value	Difference
Max Fire Room Ceiling Temperature	222.0	276.0	24.3%
Max Fire Room Floor Temperature	71.7	120.0	67.4%
Min Fire Room O2	16.5%	13.5%	-18.2%
Max BR3 Ceiling Temperature	47.7	82.8	73.6%
Max BR3 Floor Temperature	28.8	40.6	41.0%
Min BR3 O2	20.2%	15.1%	-25.2%

6.2.6 Experiment 25

The time versus temperature graphs for the four basement quadrants are provided in Figure 6.30. Comparison of experimental results to the simulation results show that predicted temperatures near the ceiling were much closer to the experimental values than those predicted near the floor. Temperature stratification was similar between the experimental and modeled data. Heat transfer into the mechanical room was underpredicted in the simulation.

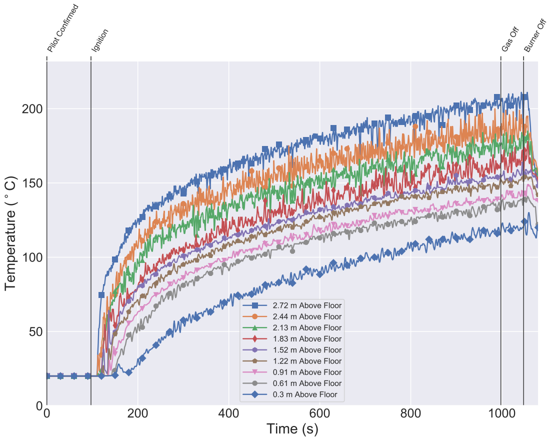

(a) Basement A Quadrant Temperatures

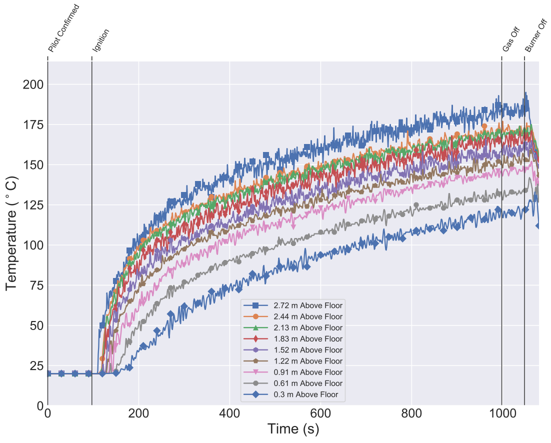

(b) Basement B Quadrant Temperatures

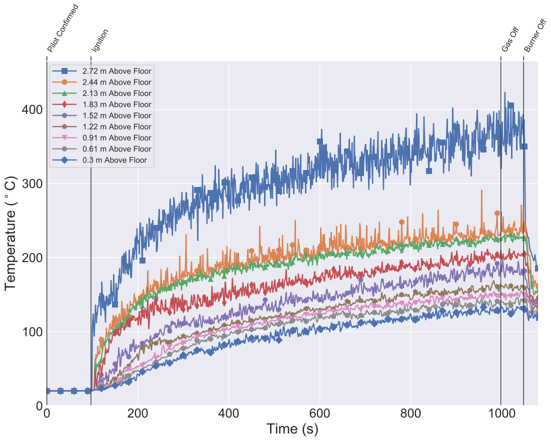

(c) Basement C Quadrant Temperatures

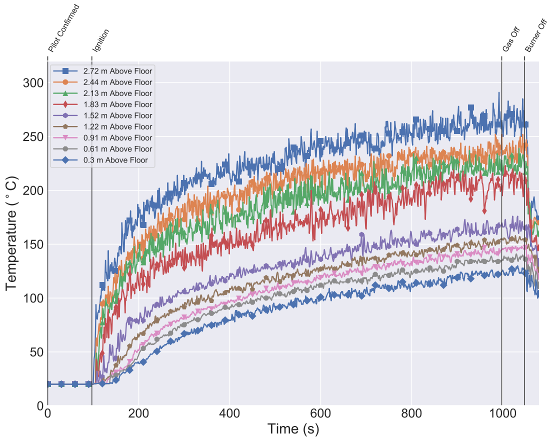

(d) Basement D Quadrant Temperatures

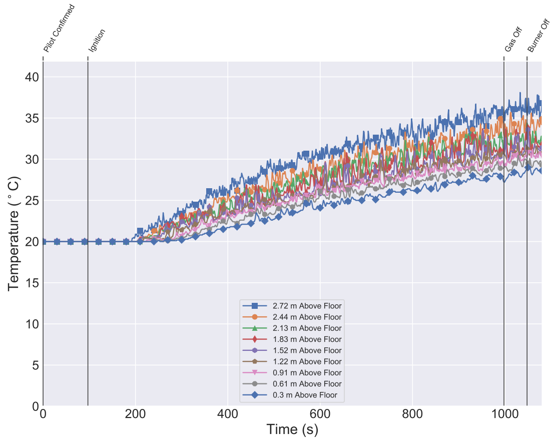

(e) Basement Mechanical Room

Figure 6.30: FDS Experiment 25 Basement Temperatures

The time versus temperature graphs from the three bedrooms, the living room, the dining room and the kitchen are provided in Figure 6.31. Heat transfer to the first floor was grossly overpredicted by the simulation. Actual temperature increases in bedrooms 1, 2 and 3 were in the range of 5 °C to 15 °C, while simulation results showed increases of 30 °C or greater. Similarly, temperature increases on the first floor were gradual in the experiment but showed sharp increases in the model. The sharp spikes are due to overpredicted heat transfer through the modeled ductwork. As was the case in the bedrooms, temperatures elsewhere on the first floor were overpredicted and had rapid temperature increases in the model. A brief comparison to FDS Experiment 23 shows that the closed staircase door did result in a smaller temperature increase in all rooms on the first floor.

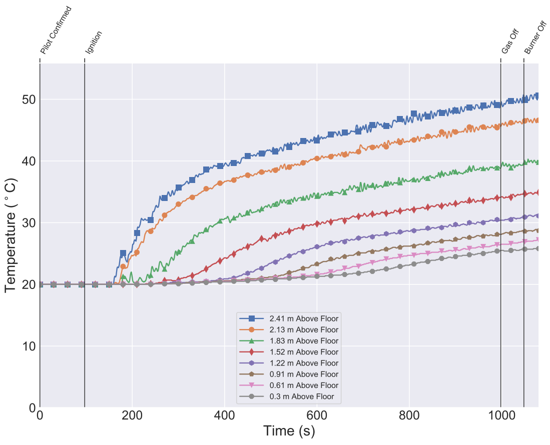

(a) Bedroom 1 Temperatures

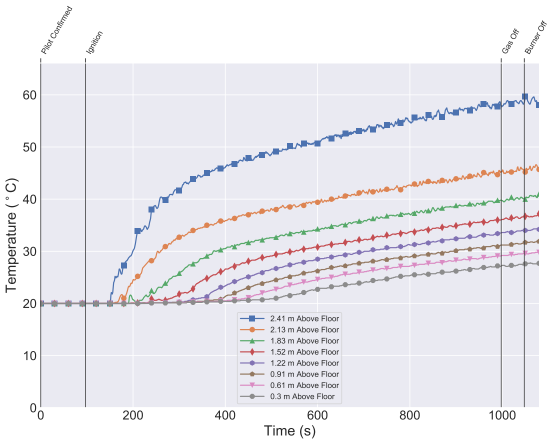

(b) Bedroom 2 Temperatures

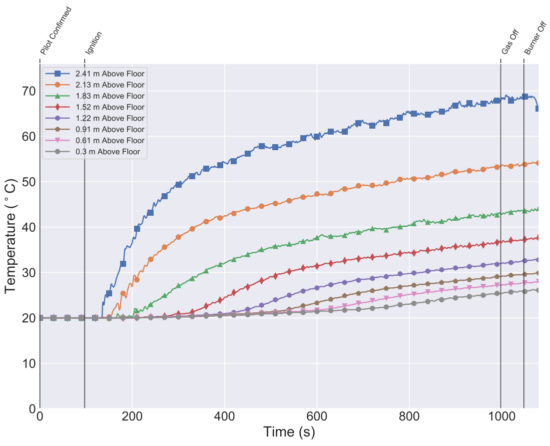

(c) Bedroom 3 Temperatures

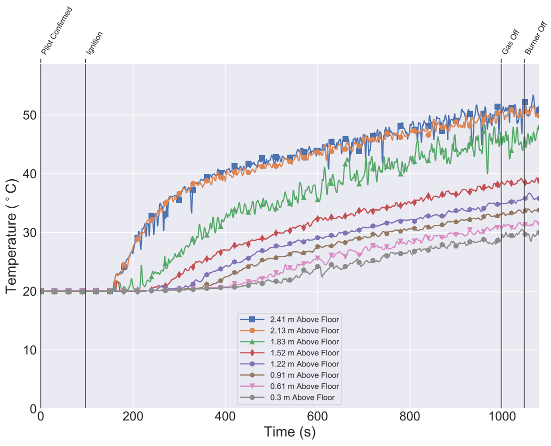

(d) Living Room Temperatures

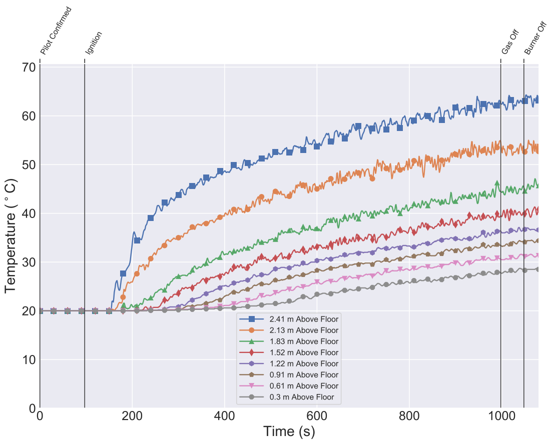

(e) Dining Room Temperatures

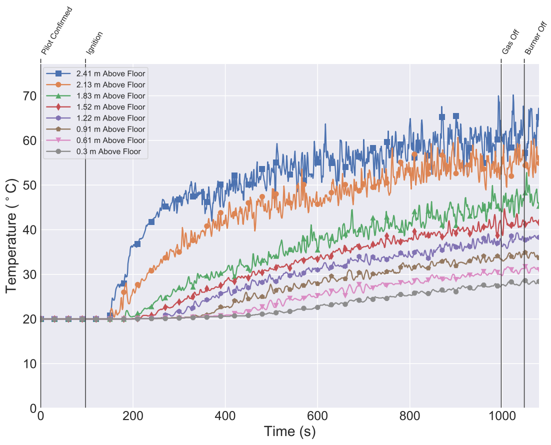

(f) Kitchen Temperatures

Figure 6.31: FDS Experiment 25 First Floor Temperatures

The pressures within each room are shown in Figure 6.32. The pressure charts generated by the simulation vary greatly to those of Experiment 25, similar to other FDS experiments. FDS pressures spiked a couple hundred pascals following ignition and then fluctuated 10s of pascals higher than the experimental data. While the scales of pressure spikes vary between experimental and simulated, the general shape of the pressure graphs are similar for first floor pressures.

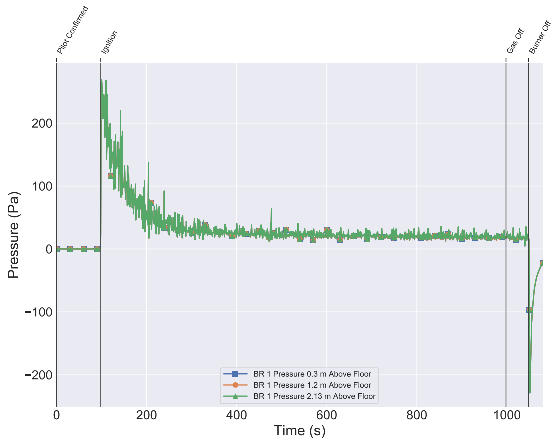

(a) Bedroom 1 Pressures

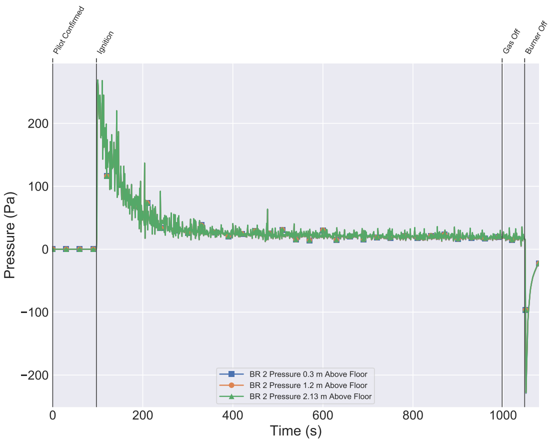

(b) Bedroom 2 Pressures

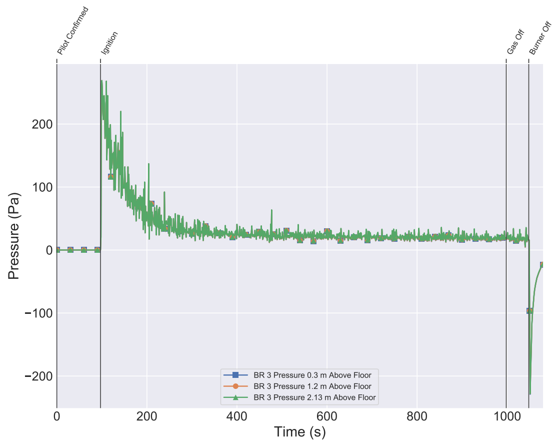

(c) Bedroom 3 Pressures

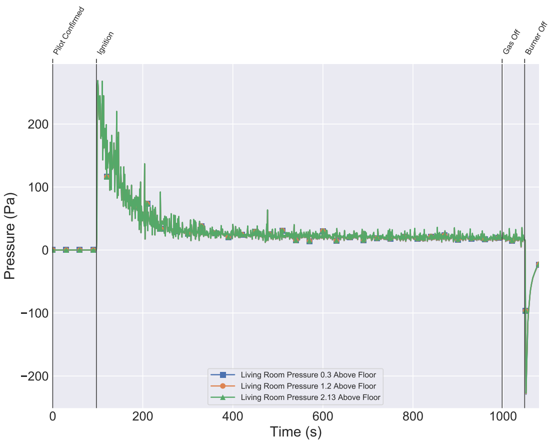

(d) Living Room Pressures

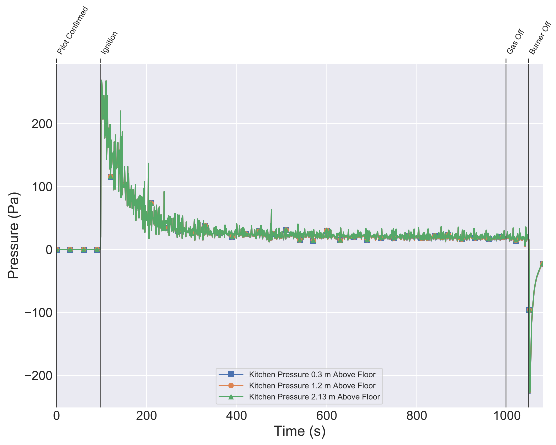

(e) Kitchen Pressures

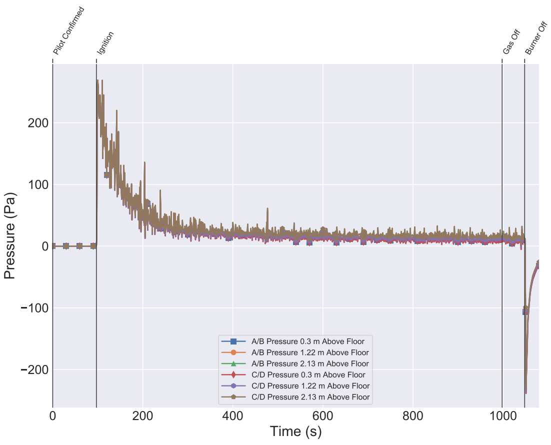

(f) Basement Pressures

Figure 6.32: FDS Experiment 25 Pressures

The concentrations of oxygen and carbon dioxide are provided for the three bedrooms and the basement in Figure 6.33. Water vapor concentrations are included for bedroom 2, bedroom 3 and the basement. Similar to FDS Experiment 23, oxygen concentrations drop more in the modeled experiment than in the actual gas burner experiment, for all measured locations. In the experimental results, the oxygen concentrations in bedroom 1 and bedroom 3 were almost unchanged, but in the simulation results both rooms dropped approximately 3% in oxygen concentrations. In bedroom 2 and the basement, the oxygen concentration drop was overpredicted by approximately 2%. Water vapor trends were similar in the basement and were overpredicted in bedrooms 2 and 3.

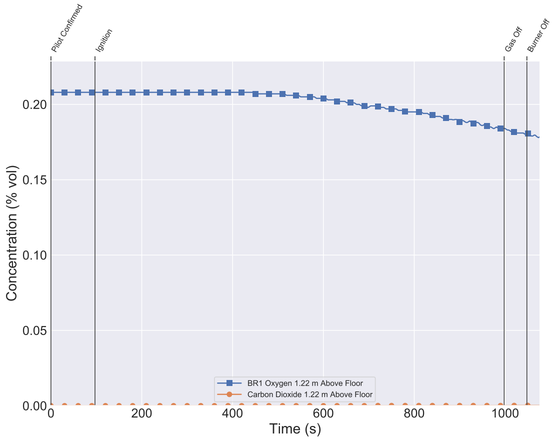

(a) Bedroom 1 Gases

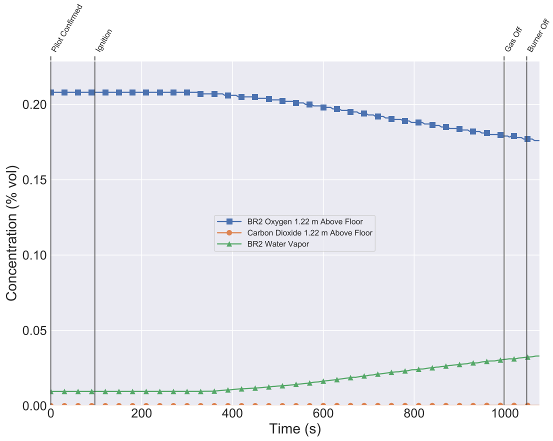

(b) Bedroom 2 Gases

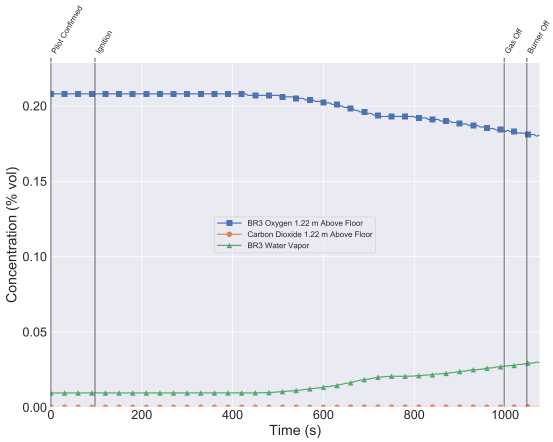

(c) Bedroom 3 Gases

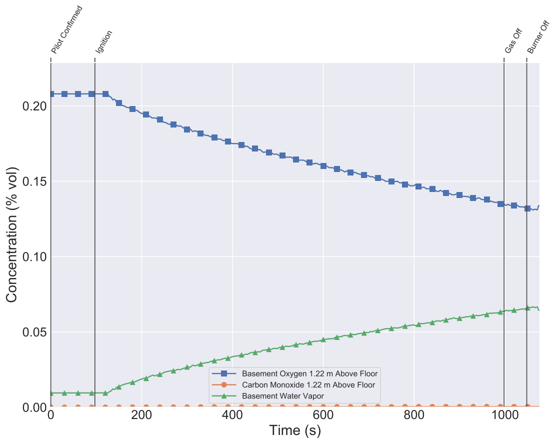

(d) Basement Gases

Figure 6.33: FDS Experiment 25 Gases

Figure 6.34 shows the temperatures of the supply and return vents in the structure. As was the case in previous simulated experiments, supply vent temperatures on the first floor are grossly overpredicted by the simulation. Return vent temperatures are similar to those recorded in the experiments. Basement vent temperatures are similar to the experimental results with the exception of the return vent in the basement being overpredicted.

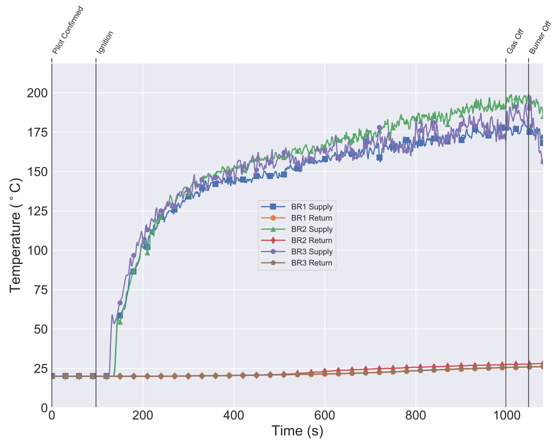

(a) Bedroom Vent Temperatures

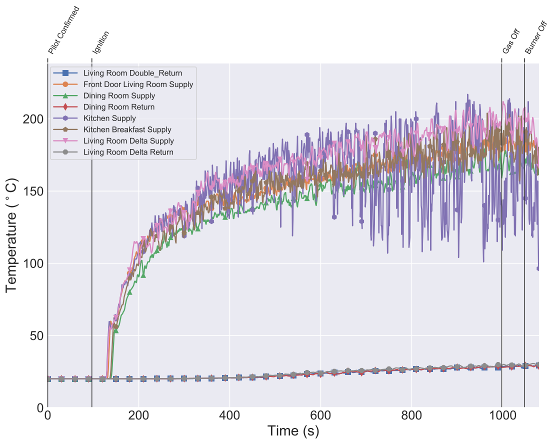

(b) First Floor Vent Temperatures

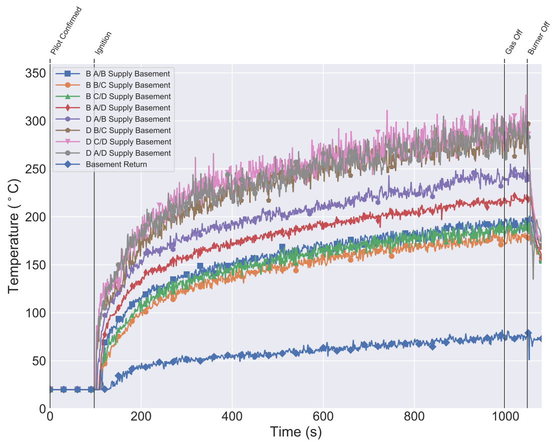

(c) Basement Vent Temperatures

Figure 6.34: FDS Experiment 25 Vent Temperatures

The temperature and velocity within the supply duct are provided in Figure 6.35. Temperature in the supply vent is overpredicted by approximately 100 °C in the simulated experiment. Recorded velocity within the supply duct is similar between the simulated and experimental data.

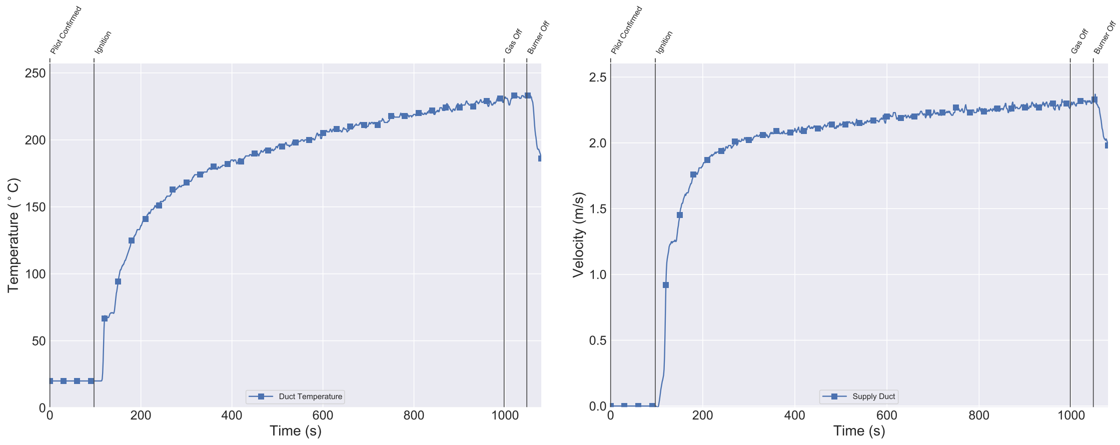

(a) Supply Duct Temperature (b) Supply Duct Velocity

Figure 6.35: FDS Experiment 25 Supply Duct Temperature and Velocity

Table 6.6 below shows a comparison of fire room data and bedroom 3 data collected from experimental and simulated data. Simulated temperatures in the fire room over predicted the experimental data by approximately 50 °C at the ceiling and the floor, as was noted in the previous basement fire simulation. The bedroom 3 ceiling temperature was again over predicted by almost 75%. In contrast to the previous simulation, the simulated bedroom 3 temperature at floor was much closer to the experimental value. Oxygen consumption in the structure was again greater than the experimental values but not greater than a 15% differential at either location.

Table 6.6: Experiment 25 Comparisons

Description	Experimental Value	FDS Value	Difference
Max Fire Room Ceiling Temperature	248.0	291.0	17.3%
Max Fire Room Floor Temperature	84.9	130.0	53.1%
Min Fire Room O2	15.3%	13.1%	-14.4%
Max BR3 Ceiling Temperature	39.8	69.1	73.6%
Max BR3 Floor Temperature	28.7	26.2	-8.7%
Min BR3 O2	20.7%	18.0%	-13.0%

Chapter 7: Conclusions

From the data collected in the gas burner experiments, an initial understanding could be developed for how products of combustion are transported through a structure by means of a residential HVAC system. The experimental data displayed heat transfer through the HVAC system that bypassed closed doorways between rooms and floors. Increased heat and fire gases were detected in the closed bedroom with the HVAC system being the easiest path of transport. The experimental data also showed the effect that an active HVAC system could have on limiting heat and gas transfer to other parts of the structure, so long as the pressures within the HVAC system were able to overcome those generated by the fire. An HVAC system set to A/C was able to prevent transport through the HVAC system the longest, but a system set to heat was effective until the system shut off due to the desired temperature being reached at the thermostat.

From the first set of experiments conducted in bedroom 1, it was observed that products of combustion had little extension into the basement of the structure, regardless of doorway openings and the status of the HVAC system. Small amounts of heat transfer could be noted in vent temperatures in the basement and in the supply vent ccnnecting the two levels. Heat and fire gases were able to be transported across

the first floor via open doorways and through the HVAC supply system. Similar conclusions were drawn from analysis of the living room experiments conducted.

From the basement gas burner experiments conducted, heat transfer up to the first floor was more notable as compared to downward flow. Heat and fire gases were able to transverse the HVAC ductwork to reach sensors behind closed doorways. In basement gas burner experiments with the HVAC system on, large deposits of soot would build up on the HVAC filter and cause restricted airflow into the HVAC fan. The notable increase in soot deposition was attributed to the basement HVAC return vent accounting for approximately half of the return air to the furnace.

Comparison of gas burner experiments to FDS simulations yielded comparison data presented in Table 7.1. A negative percentage indicates that the FDS simulation value was less than the experimental data. FDS heat transfer predictions through the HVAC system to bedroom 3 yielded temperatures that were greater than the gas burner experiment data in all simulations. This conclusion is expected because the FDS HVAC submodel does not account for heat loss in the ductwork. Oxygen concentration predictions in FDS yielded results that were lower than the experimental data for the majority of locations and experiments. Greater oxygen values in the experimental data could be due to greater leakage in the experimental structure than was accounted for in FDS.

Table 7.1: FDS Simulation Differential Comparisons

Description	Exp 1	Exp 12	Exp 15	Exp 16	Exp 23	Exp 25
Max FR Ceiling Temp	0.7%	0.0%	29.5%	13.9%	24.3%	17.3%
Max FR Floor Temp	18.8%	-20.1%	24.4%	21.4%	67.4%	53.1%
Min FR O2	-10.6%	-6.6%	-33.9%	-33.2%	-18.2%	-14.4%
Max BR3 Ceiling Temp	52.1%	54.4%	33.7%	36.7%	73.6%	73.6%
Max BR3 Floor Temp	11.5%	13.7%	-0.7%	0.4%	41.0%	-8.7%
Min BR3 O2	4.8%	-0.5%	-20.7%	-25.1%	-25.2%	-13.0%

*Calculations based on degrees Celcius temperature scale
**FR stands for Fire Room

7.1 Future Work

As with most research, the possibilities for future work are plentiful. One point of interest to help further an understanding of heat transfer within an HVAC system would be to instrument all junction points and duct segments within an HVAC system to then compare to HVAC submodel node and segment data that can be output by FDS. Instrumentation within the ductwork would help to track heat transfer through the system at a finer resolution than monitoring vent locations only. Additionally, experimental data sets were collected during these experiments to analyze heat transfer with the HVAC system active, however no FDS simulations were conducted in this report to compare to that data. Further simulation and experimental data comparisons would be able to identify other trends or support trends identified in this report.

Another area that should be researched in future work is that of heat loss through the ductwork. As mentioned in this report, FDS does not currently account for heat loss within the duct system. One possible approach for this would be to

calculate losses using heat transfer within the ducts, and then apply that to the aircoil feature within the FDS HVAC submodel. While we do not yet know the effect that heat loss within the ducts would have, based on our data, any simulated losses would help narrow the gap between experimental and simulated temperatures recorded in our structure.

Future work should also be conducted to examine how grid sensitivity affects heat transfer through the HVAC submodel. While a brief comparison was made during the ASHRAE cold flow experiments, no analysis was performed to evaluate the effect of grid resolution on heat transfer within the ductwork.

Chapter A: Cold Flow Losses

In the process of developing the HVAC component of the FDS model a separate set of modeling experiments needed to be performed. This separate model was utilized to compare collected flowrates from the DELCO structure's HVAC supply and return vents to the FDS model output. The fan curve input function of FDS was utilized and duct characteristics such as length and area were input into the model. An additional aspect needed to model the ductwork was the losses incurred within each duct segment. This loss value is the dimensionless minor losses within the duct caused by turns, junctions and control devices [8]. Prior to the start of the propane gas burner experiments, data was collected on the flow rates in and out of all the vents in the DELCO structure. To determine the flowrates, a flow hood [30, 31] was used to measure flow rates out of each vent while the HVAC fan was set in the 'on' setting. These measured values were the targets for the series of fan curve simulations performed. An accepted value range was formed based on these measured values and the provided accuracy range from the flow hood users guide. On the high end the flow value could be an additional three percent of the measured value plus 7 cfm. On the low end the flow value could be a reduction of three percent minus 7 cfm. The target flows along with the error range, plus or

minus, can be seen in Tables A.1 and A.2 below.

Table A.1: Flow Hood Supply Vent Results

Vent	Target Flows (cfm)	Error Range (+/-)
S-01	110	9.4
S-02	115	9.1
S-03	105	9.7
S-04	85	11.2
S-05	70	13.0
S-06	65	13.8
S-07	90	10.8
S-08	100	10.0
S-09	55	15.7
S-10	50	17.0
S-11	55	15.7
S-12	55	15.7
S-13	50	17.0
S-14	55	15.7
S-15	65	13.8
S-16	50	17.0

Table A.2: Flow Hood Return Vent Results

Vent	Target Flows (cfm)	Error Range (+/-)
R-01/02	525	4.3
R-03	65	13.8
R-04/05	130	8.4
R-06	75	12.3
R-07	55	15.7
R-08	30	26.3
R-09	30	26.3

In order to run initial simulations to compare duct flow outputs, an initial value for these losses needed to be determined. Utilizing the 2017 ASHRAE Handbook for Fundamentals, an initial minor loss was calculated for each duct section. Based on Table 3 in the third chapter, estimated minor losses for each duct segment were calculated based on the number of turns the duct segment made and the connection tees that airflow would traverse. For a duct segment that made a 90 ° turn, a loss of 0.6, 0.9 or 1.3 was assessed based on if the curve was a long radius, short radius or mitered corner, respectively. For airflow through a connection tee, if the airflow changed direction in the tee a loss of 1.8 was assessed. If the airflow continued straight through the tee then a loss of 0.5 was assessed. The minor loss coefficients estimated initially are shown in Tables A.3 and A.4 and were used in the FDS model to complete the first cold flow simulation, ASHRAE 1 [29]. The results of ASHRAE

1 flow rates were compared to the values collected in the DELCO structure. Based on the collected data, losses were tweeked in specific ways to direct more or less flow to certain vents and parts of the structure.

Table A.3: ASHRAE 1 Supply System

Duct Segment	Minor Losses
R-FURNACE to S-FURNACE	1.3
ST-FURNACE to ST-01	0.0
ST-01 to ST-02	2.9
ST-01 to ST-06	3.2
ST-01 to ST-10	2.3
ST-02 to S-05	4.5
ST-02 to ST-03	0.5
ST-03 to S-06	4.5
ST-03 to ST-04	1.1
ST-04 to S-07	3.6
ST-04 to S-08	4.1
ST-06 to S-04	3.6
ST-06 to ST-07	0.5
ST-07 to S-03	3.6
ST-07 to ST-08	0.5
ST-08 to S-02	3.6
ST-08 to S-01	4.1
ST-10 to ST-11	1.8
ST-11 to S-13	3.6
ST-11 to ST-12	0.5
ST-12 to S-14	3.6
ST-12 to ST-13	0.5
ST-13 to S-15	3.6
ST-13 to S-16	4.1
ST-10 to ST-15	1.8
ST-15 to S-12	3.6
ST-15 to ST-16	0.5
ST-16 to S-11	3.6
ST-16 to ST-17	0.5
ST-17 to S-09	4.1
ST-17 to S-10	3.6

Table A.4: ASHRAE 1 Return System

Duct Segment	Minor Losses
R-01 to RT-09	0.9
R-02 to RT-09	1.4
RT-09 to RT-10	1.8
R-03 to RT-02	5.4
R-04 to RT-02	4.9
RT-02 to RT-03	0.5
R-05 to RT-03	4.9
RT-03 to RT-04	1.8
R-09 to RT-07	5.4
R-08 to RT-07	4.9
RT-07 to RT-06	0.5
R-07 to RT-06	4.9
RT-06 to RT-05	0.5
R-06 to RT-05	4.9
RT-05 to RT-04	1.8
RT-04 to RT-10	1.4
RT-10 to R-FURNACE	0.9

Table A.5: ASHRAE 1 Supply Results

Vent	Model (cfm)	Target (cfm)	Difference (%)	Accepted Range (+/-)
S-01	82.0	110	-25.5	9.4
S-02	94.6	115	-17.8	9.1
S-03	97.4	105	-7.2	9.7
S-04	97.2	85	14.3	11.2
S-05	98.2	70	40.2	13.0
S-06	92.3	65	42.0	13.8
S-07	96.0	90	6.7	10.8
S-08	91.7	100	-8.3	10.0
S-09	49.9	55	-9.2	15.7
S-10	53.9	50	7.9	17.0
S-11	54.3	55	-1.2	15.7
S-12	58.0	55	5.5	15.7
S-13	58.7	50	17.4	17.0
S-14	55.0	55	0.0	15.7
S-15	53.4	65	-17.9	13.8
S-16	51.2	50	2.3	17.0

Table A.6: ASHRAE 1 Return Results

Vent	Model (cfm)	Target (cfm)	Difference (%)	Accepted Range (+/-)
R-01/02	443.0	525	-15.6	4.3
R-03	72.9	65	12.2	13.8
R-04/05	165.4	130	27.2	8.4
R-06	83.0	75	10.6	12.3
R-07	66.5	55	21.0	15.7
R-08	55.6	30	85.5	26.3
R-09	53.2	30	77.5	26.3

Upon completion of ASHRAE 1, an analysis of the output flows through the simulated HVAC system was performed. The simulated results were then compared to the target flows obtained. The percent difference for each vent was calculated and compared to the target flow data. A negative percent difference value indicated that the modeled flow for that vent was less than the target flow value. The comparison can be seen in Tables A.5 and A.6. In locations where the flow percentage difference was outside the acceptable range, adjustments needed to be made to the minor losses. Based on whether it was a single vent in a branch that was off or several, adjustments were made to different parts of the duct system. If an entire branch had too much flow through the vents, such was the case with all first floor returns in ASHRAE 1, the minor losses in the main branch sections were increased in arbitrary increments of 0.5 or 1.0. Increasing the minor losses along a section of ductwork

would decrease the flow through vents off of that duct. Decreasing the flow through some vents would inadvertently increase the flows elsewhere. If only the flow of one or two vents off of a main duct branch needed to be adjusted then minor losses were increased in the ducts that led from the main branch to the vent.

Once changes were made to the minor losses to adjust flowrates, the model was executed again as ASHRAE 2. The adjusted losses of ASHRAE 2 are compared to ASHRAE 1 in Tables A.7 and A.8.

Table A.7: Minor Supply Losses ASHRAE 1 & 2

Duct Segment	ASHRAE 1	ASHRAE 2
R-FURNACE to S-FURNACE	1.3	1.3
ST-FURNACE to ST-01	0.0	0.0
ST-01 to ST-02	2.9	2.9
ST-01 to ST-06	3.2	2.7
ST-01 to ST-10	2.3	2.3
ST-02 to S-05	4.5	5.5
ST-02 to ST-03	0.5	0.5
ST-03 to S-06	4.5	5.5
ST-03 to ST-04	1.1	1.1
ST-04 to S-07	3.6	3.6
ST-04 to S-08	4.1	4.1
ST-06 to S-04	3.6	3.6
ST-06 to ST-07	0.5	0.5
ST-07 to S-03	3.6	3.6
ST-07 to ST-08	0.5	0.5
ST-08 to S-02	3.6	3.6
ST-08 to S-01	4.1	4.1
ST-10 to ST-11	1.8	1.8
ST-11 to S-13	3.6	4.1
ST-11 to ST-12	0.5	0.5
ST-12 to S-14	3.6	3.1
ST-12 to ST-13	0.5	0.5
ST-13 to S-15	3.6	3.6
ST-13 to S-16	4.1	4.1
ST-10 to ST-15	1.8	1.8
ST-15 to S-12	3.6	3.6
ST-15 to ST-16	0.5	0.5
ST-16 to S-11	3.6	3.6
ST-16 to ST-17	0.5	0.5
ST-17 to S-09	4.1	4.1
ST-17 to S-10	3.6	3.6

Table A.8: Minor Return Losses ASHRAE 1 & 2

Duct Segment	ASHRAE 1	ASHRAE 2
R-01 to RT-09	0.9	0.9
R-02 to RT-09	1.4	1.4
RT-09 to RT-10	1.8	1.8
R-03 to RT-02	5.4	5.4
R-04 to RT-02	4.9	4.9
RT-02 to RT-03	0.5	0.5
R-05 to RT-03	4.9	4.9
RT-03 to RT-04	1.8	2.3
R-09 to RT-07	5.4	5.4
R-08 to RT-07	4.9	4.9
RT-07 to RT-06	0.5	1.5
R-07 to RT-06	4.9	4.9
RT-06 to RT-05	0.5	1.0
R-06 to RT-05	4.9	4.9
RT-05 to RT-04	1.8	1.8
RT-04 to RT-10	1.4	1.4
RT-10 to R-FURNACE	0.9	0.9

Table A.9: ASHRAE 2 Supply Results

Vent	Model (cfm)	Target (cfm)	Difference (%)	Accepted Range (+/-)
S-01	85.1	110	-22.6	9.4
S-02	98.1	115	-14.7	9.1
S-03	101.2	105	-3.7	9.7
S-04	100.9	85	18.7	11.2
S-05	90.3	70	29.0	13.0
S-06	85.4	65	31.4	13.8
S-07	98.0	90	8.9	10.8
S-08	93.6	100	-6.4	10.0
S-09	49.6	55	-9.9	15.7
S-10	53.5	50	7.1	17.0
S-11	54.0	55	-1.9	15.7
S-12	57.6	55	4.8	15.7
S-13	55.0	50	10.0	17.0
S-14	58.1	55	5.6	15.7
S-15	52.9	65	-18.6	13.8
S-16	50.7	50	1.5	17.0

Table A.10: ASHRAE 2 Return Results

Vent	Model (cfm)	Target (cfm)	Difference (%)	Accepted Range (+/-)
R-01/02	450.4	525	-14.2	4.3
R-03	70.2	65	8.0	13.8
R-04/05	159.1	130	22.4	8.4
R-06	93.7	75	24.9	12.3
R-07	68.3	55	24.2	15.7
R-08	49.2	30	64.0	26.3
R-09	47.1	30	56.9	26.3

Changes made to minor losses between ASHRAE 1 and ASHRAE 2 caused flows to shift in the intended direction, however some flow changes were too much while others were not enough. Tables A.9 and A.10 show the flow results from ASHRAE 2 compared to the target flows. Some vents, such as S-14, experienced inadvertent changes to flow as a result of adjustments made to other parts of the HVAC system. Minor losses were again altered by arbitrary amounts of 0.5 or 1.0 between ASHRAE 2 and ASHRAE 3 to drive the modeled flows closer to the target flows. The adjusted losses for ASHRAE 3 can be found in Tables A.11 and A.12.

Table A.11: Minor Supply Losses ASHRAE 1, 2, & 3

Duct Segment	ASHRAE 1	ASHRAE 2	ASHRAE 3
R-FURNACE to S-FURNACE	1.3	1.3	1.3
ST-FURNACE to ST-01	0.0	0.0	0.0
ST-01 to ST-02	2.9	2.9	2.9
ST-01 to ST-06	3.2	2.7	2.2
ST-01 to ST-10	2.3	2.3	2.3
ST-02 to S-05	4.5	5.5	6.0
ST-02 to ST-03	0.5	0.5	0.5
ST-03 to S-06	4.5	5.5	6.0
ST-03 to ST-04	1.1	1.1	1.1
ST-04 to S-07	3.6	3.6	3.6
ST-04 to S-08	4.1	4.1	4.1
ST-06 to S-04	3.6	3.6	4.1
ST-06 to ST-07	0.5	0.5	0.5
ST-07 to S-03	3.6	3.6	3.6
ST-07 to ST-08	0.5	0.5	0.5
ST-08 to S-02	3.6	3.6	3.6
ST-08 to S-01	4.1	4.1	4.1
ST-10 to ST-11	1.8	1.8	1.8
ST-11 to S-13	3.6	4.1	4.1
ST-11 to ST-12	0.5	0.5	0.5
ST-12 to S-14	3.6	3.1	3.6
ST-12 to ST-13	0.5	0.5	0.5
ST-13 to S-15	3.6	3.6	3.1
ST-13 to S-16	4.1	4.1	4.1
ST-10 to ST-15	1.8	1.8	1.8
ST-15 to S-12	3.6	3.6	3.6
ST-15 to ST-16	0.5	0.5	0.5
ST-16 to S-11	3.6	3.6	3.6
ST-16 to ST-17	0.5	0.5	0.5
ST-17 to S-09	4.1	4.1	4.1
ST-17 to S-10	3.6	3.6	3.6

Table A.12: Minor Return Losses ASHRAE 1, 2, & 3

Duct Segment	ASHRAE 1	ASHRAE 2	ASHRAE 3
R-01 to RT-09	0.9	0.9	0.9
R-02 to RT-09	1.4	1.4	1.4
RT-09 to RT-10	1.8	1.8	1.8
R-03 to RT-02	5.4	5.4	5.4
R-04 to RT-02	4.9	4.9	4.9
RT-02 to RT-03	0.5	0.5	0.5
R-05 to RT-03	4.9	4.9	4.9
RT-03 to RT-04	1.8	2.3	1.8
R-09 to RT-07	5.4	5.4	5.4
R-08 to RT-07	4.9	4.9	4.9
RT-07 to RT-06	0.5	1.5	2.0
R-07 to RT-06	4.9	4.9	4.9
RT-06 to RT-05	0.5	1.0	1.0
R-06 to RT-05	4.9	4.9	4.9
RT-05 to RT-04	1.8	1.8	2.8
RT-04 to RT-10	1.4	1.4	1.4
RT-10 to R-FURNACE	0.9	0.9	0.9

The same analysis process that was used to analyze ASHRAE 1 & 2 results was done for ASHRAE 3. Alterations to minor losses were made once again before

executing ASHRAE 4.

Table A.13: Minor Supply Losses ASHRAE 4

Duct Segment	Minor Losses
R-FURNACE to S-FURNACE	1.3
ST-FURNACE to ST-01	0.0
ST-01 to ST-02	3.4
ST-01 to ST-06	2.2
ST-01 to ST-10	2.3
ST-02 to S-05	6.0
ST-02 to ST-03	0.5
ST-03 to S-06	6.0
ST-03 to ST-04	1.1
ST-04 to S-07	3.6
ST-04 to S-08	4.1
ST-06 to S-04	4.6
ST-06 to ST-07	0.5
ST-07 to S-03	3.6
ST-07 to ST-08	0.5
ST-08 to S-02	3.1
ST-08 to S-01	3.6
ST-10 to ST-11	1.8
ST-11 to S-13	4.1
ST-11 to ST-12	0.5
ST-12 to S-14	3.6
ST-12 to ST-13	0.5
ST-13 to S-15	2.6
ST-13 to S-16	4.1
ST-10 to ST-15	1.8
ST-15 to S-12	3.6
ST-15 to ST-16	0.5
ST-16 to S-11	3.6
ST-16 to ST-17	0.5
ST-17 to S-09	4.1
ST-17 to S-10	3.6

Table A.14: Minor Return Losses ASHRAE 4

Duct Segment	Minor Losses
R-01 to RT-09	0.9
R-02 to RT-09	1.4
RT-09 to RT-10	1.8
R-03 to RT-02	5.4
R-04 to RT-02	4.9
RT-02 to RT-03	0.5
R-05 to RT-03	4.9
RT-03 to RT-04	2.3
R-09 to RT-07	5.4
R-08 to RT-07	4.9
RT-07 to RT-06	2.5
R-07 to RT-06	4.9
RT-06 to RT-05	1.0
R-06 to RT-05	4.9
RT-05 to RT-04	2.8
RT-04 to RT-10	1.4
RT-10 to R-FURNACE	0.9

After the completion of the ASHRAE 4 simulation and analysis of the vent flows, it could be seen that all but a few vents were flowing within the acceptable

range. At this point the simulation mesh size was reduced from a 20cm x 20cm grid to a 10cm x 10cm grid. Initial trials were performed in the larger grid size to facilitate quicker simulation times that would yield useful results. ASHRAE 4 was then simulated again with the same losses but a reduced mesh size. A comparison between the different mesh size results can be seen in Tables A.15 and A.16. This table shows that a change in mesh size, without changing any minor loss values, has a small but noticable impact on the flow rates. The reduced mesh size allows FDS to complete more precise calculations along flow boundaries and within the modeled field. Upon completion of ASHRAE 4 with a reduced mesh size, the modeled flows for approximately 75 percent of the vents were within the acceptable range. An iteration based on the results was performed to adjust the losses for ASHRAE 5. ASHRAE 5 was then initiated with a grid scale of 10cm x 10cm to maintain more precise results.

Table A.15: ASHRAE 4 Supply Results

Vent	20cm Grid (cfm)	10cm Grid (cfm)
S-01	94.5	95.2
S-02	111.5	112.9
S-03	107.7	109.3
S-04	96.2	97.1
S-05	86.0	87.7
S-06	81.1	82.4
S-07	96.6	97.8
S-08	96.2	94.6
S-09	50.0	50.5
S-10	54.1	54.6
S-11	54.6	55.1
S-12	58.4	59.1
S-13	55.5	56.3
S-14	54.8	55.4
S-15	60.5	61.1
S-16	50.7	51.3

Table A.16: ASHRAE 4 Return Results

Vent	20cm Grid (cfm)	10cm Grid (cfm)
R-01/02	531.0	538.0
R-03	67.7	67.7
R-04/05	152.5	153.9
R-06	78.5	79.2
R-07	59.3	59.7
R-08	37.5	38.0
R-09	36.0	36.3

Table A.17: Minor Supply Losses ASHRAE 5

Duct Segment	ASHRAE 5 Losses
R-FURNACE to S-FURNACE	1.3
ST-FURNACE to ST-01	0.0
ST-01 to ST-02	3.4
ST-01 to ST-06	2.2
ST-01 to ST-10	2.3
ST-02 to S-05	7.0
ST-02 to ST-03	0.5
ST-03 to S-06	7.0
ST-03 to ST-04	1.1
ST-04 to S-07	4.1
ST-04 to S-08	4.1
ST-06 to S-04	5.1
ST-06 to ST-07	0.5
ST-07 to S-03	3.6
ST-07 to ST-08	0.5
ST-08 to S-02	3.1
ST-08 to S-01	3.6
ST-10 to ST-11	1.8
ST-11 to S-13	4.1
ST-11 to ST-12	0.5
ST-12 to S-14	3.6
ST-12 to ST-13	0.5
ST-13 to S-15	2.6
ST-13 to S-16	4.1
ST-10 to ST-15	1.8
ST-15 to S-12	3.6
ST-15 to ST-16	0.5
ST-16 to S-11	3.6
ST-16 to ST-17	0.5
ST-17 to S-09	4.1
ST-17 to S-10	3.6

Table A.18: Minor Return Losses ASHRAE 5

Duct Segment	ASHRAE 5 Losses
R-01 to RT-09	0.9
R-02 to RT-09	1.4
RT-09 to RT-10	1.8
R-03 to RT-02	5.4
R-04 to RT-02	5.4
RT-02 to RT-03	0.5
R-05 to RT-03	5.4
RT-03 to RT-04	2.3
R-09 to RT-07	5.4
R-08 to RT-07	4.9
RT-07 to RT-06	3.0
R-07 to RT-06	4.9
RT-06 to RT-05	1.0
R-06 to RT-05	4.9
RT-05 to RT-04	2.8
RT-04 to RT-10	1.4
RT-10 to R-FURNACE	0.9

Upon completion of ASHRAE 5 all but three vents were within their allowable flow range. Minor loss alterations were made to specific branches to adjust the

flowrates out of the vents. ASHRAE 6 was executed based on the minor losses in Tables A.19 and A.20, but did not change the flows enough to satisfy the error range. Once again minor changes were made to specific branches to adjust the minor losses as needed.

Table A.19: Minor Supply Losses ASHRAE 6

Duct Segment	ASHRAE 6 Losses
R-FURNACE to S-FURNACE	1.3
ST-FURNACE to ST-01	0.0
ST-01 to ST-02	3.4
ST-01 to ST-06	2.2
ST-01 to ST-10	2.3
ST-02 to S-05	8.0
ST-02 to ST-03	0.5
ST-03 to S-06	8.0
ST-03 to ST-04	1.1
ST-04 to S-07	4.1
ST-04 to S-08	4.1
ST-06 to S-04	5.1
ST-06 to ST-07	0.5
ST-07 to S-03	3.6
ST-07 to ST-08	0.5
ST-08 to S-02	3.1
ST-08 to S-01	3.6
ST-10 to ST-11	1.8
ST-11 to S-13	4.1
ST-11 to ST-12	0.5
ST-12 to S-14	3.6
ST-12 to ST-13	0.5
ST-13 to S-15	2.6
ST-13 to S-16	4.1
ST-10 to ST-15	1.8
ST-15 to S-12	3.6
ST-15 to ST-16	0.5
ST-16 to S-11	3.6
ST-16 to ST-17	0.5
ST-17 to S-09	4.1
ST-17 to S-10	3.6

and A.22.

Table A.20: Minor Return Losses ASHRAE 6

Duct Segment	ASHRAE 6 Losses
R-01 to RT-09	0.9
R-02 to RT-09	1.4
RT-09 to RT-10	1.8
R-03 to RT-02	5.4
R-04 to RT-02	6.4
RT-02 to RT-03	0.5
R-05 to RT-03	6.4
RT-03 to RT-04	2.3
R-09 to RT-07	5.4
R-08 to RT-07	4.9
RT-07 to RT-06	3.0
R-07 to RT-06	4.9
RT-06 to RT-05	1.0
R-06 to RT-05	4.9
RT-05 to RT-04	2.8
RT-04 to RT-10	1.4
RT-10 to R-FURNACE	0.9

ASHRAE 7 was executed with the minor loss values presented in Tables A.21 and A.22.

Table A.21: Minor Supply Losses ASHRAE 7

Duct Segment	ASHRAE 7 Losses
R-FURNACE to S-FURNACE	1.3
ST-FURNACE to ST-01	0.0
ST-01 to ST-02	3.9
ST-01 to ST-06	2.2
ST-01 to ST-10	2.3
ST-02 to S-05	8.0
ST-02 to ST-03	0.5
ST-03 to S-06	8.0
ST-03 to ST-04	1.1
ST-04 to S-07	4.1
ST-04 to S-08	4.1
ST-06 to S-04	5.1
ST-06 to ST-07	0.5
ST-07 to S-03	3.6
ST-07 to ST-08	0.5
ST-08 to S-02	3.1
ST-08 to S-01	3.6
ST-10 to ST-11	1.8
ST-11 to S-13	4.1
ST-11 to ST-12	0.5
ST-12 to S-14	3.6
ST-12 to ST-13	0.5
ST-13 to S-15	2.6
ST-13 to S-16	4.1
ST-10 to ST-15	1.8
ST-15 to S-12	3.6
ST-15 to ST-16	0.5
ST-16 to S-11	3.6
ST-16 to ST-17	0.5
ST-17 to S-09	4.1
ST-17 to S-10	3.6

Table A.22: Minor Return Losses ASHRAE 7

Duct Segment	ASHRAE 7 Losses
R-01 to RT-09	0.9
R-02 to RT-09	1.4
RT-09 to RT-10	1.8
R-03 to RT-02	5.4
R-04 to RT-02	6.4
RT-02 to RT-03	0.5
R-05 to RT-03	6.4
RT-03 to RT-04	2.8
R-09 to RT-07	5.4
R-08 to RT-07	4.9
RT-07 to RT-06	3.0
R-07 to RT-06	4.9
RT-06 to RT-05	1.0
R-06 to RT-05	4.9
RT-05 to RT-04	2.8
RT-04 to RT-10	1.4
RT-10 to R-FURNACE	0.9

Upon the completion of ASHRAE 7, flow analysis was once again performed. Upon inspection of modeled vent flows, all of the vents in the model were within

the acceptable range of target values. Tables A.23 and A.24 show the flow rates obtained from ASHRAE 7 and a comparison to the target values.

Table A.23: ASHRAE 7 Supply Results

Vent	Model (cfm)	Target (cfm)	Difference (%)	Accepted Range (+/-)
S-01	102.8	110	-6.5	9.4
S-02	114.4	115	-0.5	9.1
S-03	111.1	105	5.8	9.7
S-04	94.4	85	11.1	11.2
S-05	78.0	70	11.4	13.0
S-06	73.8	65	13.5	13.8
S-07	95.1	90	5.7	10.8
S-08	97.7	100	-2.3	10.0
S-09	51.5	55	-6.4	15.7
S-10	55.7	50	11.4	17.0
S-11	56.2	55	2.2	15.7
S-12	60.2	55	9.4	15.7
S-13	57.4	50	14.8	17.0
S-14	56.5	55	2.7	15.7
S-15	62.3	65	-4.1	13.8
S-16	52.2	50	4.5	17.0

Table A.24: ASHRAE 7 Return Results

Vent	Model (cfm)	Target (cfm)	Difference (%)	Accepted Range (+/-)
R-01/02	543.7	525	3.6	4.3
R-03	69.6	65	7.1	13.8
R-04/05	139.0	130	7.0	8.4
R-06	81.9	75	9.2	12.3
R-07	62.3	55	13.3	15.7
R-08	37.8	30	25.9	26.3
R-09	36.2	30	20.6	26.3

Figure A.1 below provide a comparison of the minor losses across all the ASHRAE models and show the percent difference in value as compared to the initial calculated minor losses utilized in ASHRAE 1. Minor loss changes are highlighted in a blue accent for a reduction in minor loss value and in a gold accent for an increase in value. A yellow highlight in the percent change column indicates an increase in minor loss value when compared to the original calculated value and a blue highlight indicates a decrease in value compared to the original.

Figure A.2 provides a comparison of the supply vent flows for all ASHRAE models and their percent difference from the target supply flows. Figure A.3 provides a comparison of the return vent flows for all ASHRAE models and their percent difference from the target return flows. A cell that is highlighted in a dark blue accent indicates that the flow from that vent is less than the low acceptable range

limit. A light blue accent means it is less than the target flow but within the acceptable range. A light gold accent means the flow is above the target flow but within the acceptable range. A dark gold accent means that the flow is greater than the high acceptable range limit. In this table the flow rate is also provided in $\frac{m^3}{s}$ because that is how the values were calculated and presented in the FDS. The conversion was made from $\frac{m^3}{s}$ to cfm using equation A.1 below.

$$cfm = \frac{m^3}{s} * 60 \frac{s}{min} * 35.3147 \frac{ft^3}{m^3} \tag{A.1}$$

Duct_ID	ASHRAE 1	ASHRAE 2	ASHRAE 3	ASHRAE 4	ASHRAE 5	ASHRAE 6	ASHRAE 7	Percent Change
R-FURNACE_S-FURNACE	1.3	1.3	1.3	1.3	1.3	1.3	1.3	0.0
S-Furnace_ST-01	0.0	0.0	0.0	0.0	0.0	0.0	0.0	0.0
ST-01_ST-02	2.9	2.9	2.9	3.4	3.4	3.4	3.9	17.2
ST-01_ST-06	3.2	2.7	2.2	2.2	2.2	2.2	2.2	-31.3
ST-01_ST-10	2.3	2.3	2.3	2.3	2.3	2.3	2.3	0.0
ST-02_S-05	4.5	5.5	6.0	6.0	7.0	8.0	8.0	55.6
ST-02_ST-03	0.5	0.5	0.5	0.5	0.5	0.5	0.5	0.0
ST-03_S-06	4.5	5.5	6.0	6.0	7.0	8.0	8.0	55.6
ST-03_ST-04	1.1	1.1	1.1	1.1	1.1	1.1	1.1	0.0
ST-04_S-07	3.6	3.6	3.6	3.6	4.1	4.1	4.1	13.9
ST-04_S-08	4.1	4.1	4.1	4.1	4.1	4.1	4.1	0.0
ST-06_S-04	3.6	3.6	4.1	4.6	5.1	5.1	5.1	41.7
ST-06_ST-07	0.5	0.5	0.5	0.5	0.5	0.5	0.5	0.0
ST-07_S-03	3.6	3.6	3.6	3.6	3.6	3.6	3.6	0.0
ST-07_ST-08	0.5	0.5	0.5	0.5	0.5	0.5	0.5	0.0
ST-08_S-02	3.6	3.6	3.6	3.1	3.1	3.1	3.1	-13.9
ST-08_S-01	4.1	4.1	4.1	3.6	3.1	3.1	3.1	-24.4
ST-10_ST-11	1.8	1.8	1.8	1.8	1.8	1.8	1.8	0.0
ST-11_S-13	3.6	4.1	4.1	4.1	4.1	4.1	4.1	13.9
ST-11_ST-12	0.5	0.5	0.5	0.5	0.5	0.5	0.5	0.0
ST-12_S-14	3.6	3.1	3.6	3.6	3.6	3.6	3.6	0.0
ST-12_ST-13	0.5	0.5	0.5	0.5	0.5	0.5	0.5	0.0
ST-13_S-15	3.6	3.6	3.1	2.6	2.6	2.6	2.6	-27.8
ST-13_S-16	4.1	4.1	4.1	4.1	4.1	4.1	4.1	0.0
ST-10_ST-15	1.8	1.8	1.8	1.8	1.8	1.8	1.8	0.0
ST-15_S-12	3.6	3.6	3.6	3.6	3.6	3.6	3.6	0.0
ST-15_ST-16	0.5	0.5	0.5	0.5	0.5	0.5	0.5	0.0
ST-16_S-11	3.6	3.6	3.6	3.6	3.6	3.6	3.6	0.0
ST-16_ST-17	0.5	0.5	0.5	0.5	0.5	0.5	0.5	0.0
ST-17_S-09	4.1	4.1	4.1	4.1	4.1	4.1	4.1	0.0
ST-17_S-10	3.6	3.6	3.6	3.6	3.6	3.6	3.6	0.0
R-01_RT-09	0.9	0.9	0.9	0.9	0.9	0.9	0.9	0.0
R-02_RT-09	1.4	1.4	1.4	1.4	1.4	1.4	1.4	0.0
RT-09_RT-10	1.8	1.8	1.8	0.8	0.8	0.8	0.8	-55.6
R-03_RT-02	5.4	5.4	5.4	5.4	5.4	5.4	5.4	0.0
R-04_RT-02	4.9	4.9	4.9	4.9	5.4	6.4	6.4	10.2
RT-02_RT-03	0.5	0.5	0.5	0.5	0.5	0.5	0.5	0.0
R-05_RT-03	4.9	4.9	4.9	4.9	5.4	6.4	6.4	10.2
RT-03_RT-04	1.8	2.3	1.8	2.3	2.3	2.3	2.8	27.8
R-09_RT-07	5.4	5.4	5.4	5.4	5.4	5.4	5.4	0.0
R-08_RT-07	4.9	4.9	4.9	4.9	4.9	4.9	4.9	0.0
RT-07_RT-06	0.5	1.5	2.0	2.5	3.0	3.0	3.0	500.0
R-07_RT-06	4.9	4.9	4.9	4.9	4.9	4.9	4.9	0.0
RT-06_RT-05	0.5	1.0	1.0	1.0	1.0	1.0	1.0	100.0
R-06_RT-05	4.9	4.9	4.9	4.9	4.9	4.9	4.9	0.0
RT-05_RT-04	1.8	1.8	2.8	2.8	2.8	2.8	2.8	55.6
RT-04_RT-10	1.4	1.4	1.4	1.4	1.4	1.4	1.4	0.0
RT-10_R-FURNACE	0.9	0.9	0.9	0.9	0.9	0.9	0.9	0.0

Figure A.1: ASHRAE Losses

	Vent	S-01	S-02	S-03	S-04	S-05	S-06	S-07	S-08	S-09	S-10	S-11	S-12	S-13	S-14	S-15	S-16
Measured Cold Flow in Structure	ft^3/min	110	115	105	85	70	65	90	100	55	50	55	55	50	55	65	50
	m^3/min	3.1149	3.2564	2.9733	2.4069	1.9822	1.8406	2.5485	2.8317	1.5574	1.4158	1.5574	1.5574	1.4158	1.5574	1.8406	1.4158
	m^3/s	0.1	0.1	0.0	0.0	0.0	0.0	0.0	0.0	0.0	0.0	0.0	0.0	0.0	0.0	0.0	0.0
	Calculated m/s	1.3	1.3	1.2	1.0	0.8	0.6	0.8	1.1	1.0	0.9	1.0	1.0	0.9	1.0	1.2	0.9
Error Range	High Range (+3%+7)	120.3	125.5	115.2	94.6	79.1	74.0	99.7	110.0	63.7	58.5	63.7	63.7	58.5	63.7	74.0	58.5
	%Diff	9.4	9.1	9.7	11.2	13.0	13.8	10.8	10.0	15.7	17.0	15.7	15.7	17.0	15.7	13.8	17.0
	Low Range (-3%-7)	99.7	104.6	94.9	75.5	60.9	56.1	80.3	90.0	46.4	41.5	46.4	46.4	41.5	46.4	56.1	41.5
	%Diff	-9.4	-9.1	-9.7	-11.2	-13.0	-13.8	-10.8	-10.0	-15.7	-17.0	-15.7	-15.7	-17.0	-15.7	-13.8	-17.0
ASHRAE 1 20cm x 20cm	m^3/s	0.03869	0.04463	0.04599	0.04587	0.04633	0.04356	0.04532	0.04329	0.02356	0.02545	0.02565	0.02739	0.02770	0.02595	0.02520	0.02415
	ft^3/min	82.0	94.6	97.4	97.2	98.2	92.3	96.0	91.7	49.9	53.9	54.3	58.0	58.7	55.0	53.4	51.2
	Percent Difference	-25.5	-17.8	-7.2	14.3	40.2	42.0	6.7	-8.3	-9.2	7.9	-1.2	5.5	17.4	0.0	-17.9	2.3
	Within Error Range	No	No	Yes	No	No	No	Yes	Yes	Yes	Yes	Yes	Yes	No	Yes	No	Yes
ASHRAE 2 20cm x 20cm	m^3/s	0.04017	0.04632	0.04774	0.04762	0.04263	0.04032	0.04624	0.04418	0.02339	0.02527	0.02547	0.02720	0.02595	0.02742	0.02497	0.02394
	ft^3/min	85.1	98.1	101.2	100.9	90.3	85.4	98.0	93.6	49.6	53.5	54.0	57.6	55.0	58.1	52.9	50.7
	Percent Difference	-22.6	-14.7	-3.7	18.7	29.0	31.4	8.9	-6.4	-9.9	7.1	-1.9	4.8	10.0	5.6	-18.6	1.5
	Within Error Range	No	No	Yes	No	No	No	Yes	Yes	Yes	Yes	Yes	Yes	Yes	Yes	No	Yes
ASHRAE 3 20cm x 20cm	m^3/s	0.04207	0.04849	0.05000	0.04711	0.04081	0.03869	0.04629	0.04424	0.02317	0.02503	0.02523	0.02694	0.02569	0.02549	0.02633	0.02368
	ft^3/min	89.1	102.7	105.9	99.8	86.5	82.0	98.1	93.7	49.1	53.0	53.5	57.1	54.4	54.0	55.8	50.2
	Percent Difference	-19.0	-10.7	0.9	17.4	23.5	26.1	9.0	-6.3	-10.7	6.1	-2.8	3.8	8.9	-1.8	-14.2	0.4
	Within Error Range	No	No	Yes	No	No	No	Yes	Yes	Yes	Yes	Yes	Yes	Yes	Yes	No	Yes
ASHRAE 4 20cm x 20cm	m^3/s	0.04459	0.05264	0.05081	0.04540	0.04057	0.03827	0.04559	0.04540	0.02362	0.02553	0.02575	0.02754	0.02617	0.02585	0.02857	0.02392
	ft^3/min	94.5	111.5	107.7	96.2	86.0	81.1	96.6	96.2	50.0	54.1	54.6	58.4	55.5	54.8	60.5	50.7
	Percent Difference	-14.1	-3.0	2.5	13.2	22.8	24.8	7.3	-3.8	-9.0	8.2	-0.8	6.1	10.9	-0.4	-6.9	1.4
	Within Error Range	No	Yes	Yes	No	No	No	Yes	Yes	Yes	Yes	Yes	Yes	Yes	Yes	Yes	Yes
ASHRAE 4 10cm x 10cm	m^3/s	0.04493	0.05326	0.05158	0.04582	0.04141	0.03889	0.04617	0.04464	0.02383	0.02579	0.02601	0.02787	0.02657	0.02615	0.02885	0.02419
	ft^3/min	95.2	112.9	109.3	97.1	87.7	82.4	97.8	94.6	50.5	54.6	55.1	59.1	56.3	55.4	61.1	51.3
	Percent Difference	-13.5	-1.9	4.1	14.2	25.3	26.8	8.7	-5.4	-8.2	9.3	0.2	7.4	12.6	0.7	-6.0	2.5
	Within Error Range	No	Yes	Yes	No	No	No	Yes	Yes	Yes	Yes	Yes	Yes	Yes	Yes	Yes	Yes
ASHRAE 5 10cm x 10cm	m^3/s	0.04787	0.05330	0.05174	0.04398	0.03957	0.03756	0.04513	0.04635	0.02398	0.02594	0.02617	0.02803	0.02674	0.02631	0.02902	0.02433
	ft^3/min	101.4	112.9	109.6	93.2	83.8	79.6	95.6	98.2	50.8	55.0	55.5	59.4	56.7	55.7	61.5	51.6
	Percent Difference	-7.8	-1.8	4.4	9.6	19.8	22.4	6.3	-1.8	-7.6	9.9	0.8	8.0	13.3	1.4	-5.4	3.1
	Within Error Range	Yes	Yes	Yes	Yes	No	No	Yes	Yes	Yes	Yes	Yes	Yes	Yes	Yes	Yes	Yes
ASHRAE 6 10cm x 10cm	m^3/s	0.04806	0.05350	0.05194	0.04415	0.03773	0.03571	0.04603	0.04727	0.02407	0.02604	0.02627	0.02814	0.02684	0.02641	0.02913	0.02442
	ft^3/min	101.8	113.4	110.1	93.5	79.9	75.7	97.5	100.2	51.0	55.2	55.7	59.6	56.9	56.0	61.7	51.7
	Percent Difference	-7.4	-1.4	4.8	10.1	14.2	16.4	8.4	0.2	-7.3	10.4	1.2	8.4	13.7	1.7	-5.0	3.5
	Within Error Range	Yes	Yes	Yes	Yes	No	No	Yes	Yes	Yes	Yes	Yes	Yes	Yes	Yes	Yes	Yes
ASHRAE 7 10cm x 10cm	m^3/s	0.04852	0.05401	0.05243	0.04457	0.03679	0.03482	0.04488	0.04610	0.02430	0.02629	0.02652	0.02841	0.02710	0.02666	0.02941	0.02465
	ft^3/min	102.8	114.4	111.1	94.4	78.0	73.8	95.1	97.7	51.5	55.7	56.2	60.2	57.4	56.5	62.3	52.2
	Percent Difference	-6.5	-0.5	5.8	11.1	11.4	13.5	5.7	-2.3	-6.4	11.4	2.2	9.4	14.8	2.7	-4.1	4.5
	Within Error Range	Yes	Yes	Yes	Yes	Yes	Yes	Yes	Yes	Yes	Yes	Yes	Yes	Yes	Yes	Yes	Yes

Figure A.2: Supply Flow Comparison

	Vent	R-01/02	R-03	R-04/05	R-06	R-07	R-08	R-09
Measured Cold Flow in Structure	ft^3/min	-525	-65	-130	-75	-55	-30	-30
	m^3/min	-14.8663	-1.8406	-3.6812	-2.1238	-1.5574	-0.8495	-0.8495
	m^3/s	-0.2	0.0	-0.1	0.0	0.0	0.0	0.0
	Calculated m/s	-2.0	-0.5	-0.5	-0.6	-0.4	-0.2	-0.2
Error Range	High Range (+3%+7)	-502.3	-56.1	-119.1	-65.8	-46.4	-22.1	-22.1
	%Diff	-4.3	-13.8	-8.4	-12.3	-15.7	-26.3	-26.3
	Low Range (-3%-7)	-547.8	-74.0	-140.9	-84.3	-63.7	-37.9	-37.9
	%Diff	4.3	13.8	8.4	12.3	15.7	26.3	26.3
ASHRAE 1 20cm x 20cm	m^3/s	-0.20909	-0.03442	-0.07804	-0.03916	-0.03140	-0.02626	-0.02513
	ft^3/min	-443.0	-72.9	-165.4	-83.0	-66.5	-55.6	-53.2
	Percent Difference	-15.6	12.2	27.2	10.6	21.0	85.5	77.5
	Within Error Range	No	Yes	No	Yes	No	No	No
ASHRAE 2 20cm x 20cm	m^3/s	-0.21256	-0.03312	-0.07508	-0.04421	-0.03223	-0.02322	-0.02222
	ft^3/min	-450.4	-70.2	-159.1	-93.7	-68.3	-49.2	-47.1
	Percent Difference	-14.2	8.0	22.4	24.9	24.2	64.0	56.9
	Within Error Range	No	Yes	No	No	No	No	No
ASHRAE 3 20cm x 20cm	m^3/s	-0.21525	-0.03678	-0.08327	-0.03929	-0.02925	-0.01964	-0.01881
	ft^3/min	-456.1	-77.9	-176.4	-83.3	-62.0	-41.6	-39.9
	Percent Difference	-13.1	19.9	35.7	11.0	12.7	38.7	32.9
	Within Error Range	No	No	No	Yes	Yes	No	No
ASHRAE 4 20cm x 20cm	m^3/s	-0.25060	-0.03174	-0.07197	-0.03705	-0.02799	-0.01771	-0.01697
	ft^3/min	-531.0	-67.3	-152.5	-78.5	-59.3	-37.5	-36.0
	Percent Difference	1.1	3.5	17.3	4.7	7.8	25.1	19.9
	Within Error Range	Yes	Yes	No	Yes	Yes	Yes	Yes
ASHRAE 4 10cm x 10cm	m^3/s	-0.25390	-0.03196	-0.07263	-0.03740	-0.02819	-0.01792	-0.01715
	ft^3/min	-538.0	-67.7	-153.9	-79.2	-59.7	-38.0	-36.3
	Percent Difference	2.5	4.2	18.4	5.7	8.6	26.6	21.1
	Within Error Range	Yes	Yes	No	Yes	Yes	No	Yes
ASHRAE 5 10cm x 10cm	m^3/s	-0.25410	-0.03285	-0.07124	-0.03779	-0.02878	-0.01744	-0.01669
	ft^3/min	-538.4	-69.6	-150.9	-80.1	-61.0	-37.0	-35.4
	Percent Difference	2.6	7.1	16.1	6.8	10.9	23.2	17.9
	Within Error Range	Yes	Yes	No	Yes	Yes	Yes	Yes
ASHRAE 6 10cm x 10cm	m^3/s	-0.25450	-0.03438	-0.06865	-0.03794	-0.02889	-0.01751	-0.01676
	ft^3/min	-539.3	-72.8	-145.5	-80.4	-61.2	-37.1	-35.5
	Percent Difference	2.7	12.1	11.9	7.2	11.3	23.7	18.4
	Within Error Range	Yes	Yes	No	Yes	Yes	Yes	Yes
ASHRAE 7 10cm x 10cm	m^3/s	-0.25660	-0.03286	-0.06562	-0.03865	-0.02940	-0.01783	-0.01707
	ft^3/min	-543.7	-69.6	-139.0	-81.9	-62.3	-37.8	-36.2
	Percent Difference	3.6	7.1	7.0	9.2	13.3	25.9	20.6
	Within Error Range	Yes	Yes	Yes	Yes	Yes	Yes	Yes

Figure A.3: Return Flow Comparison

Chapter B: DelCo Test Structure

To assist with modeling the test structure utilized at the Delaware County Emergency Services Training Center (DelCo), Revit 2019 was used for floor plans and HVAC display. Revit is a three dimensional building information modeling software that is capable of creating detailed floor plans and HVAC systems. For the purposes of our project, Revit was more than sufficient to create a detailed model of our structure including all interior and exterior vents as well as the HVAC system. Revit's three dimensional viewing capabilities provided graphics to better display the HVAC system than could be obtained through photos of the structure. Figures B.1 through B.4 show detailed plans and images of the basement while Figures B.5 through B.8 provide details and images of the first floor.

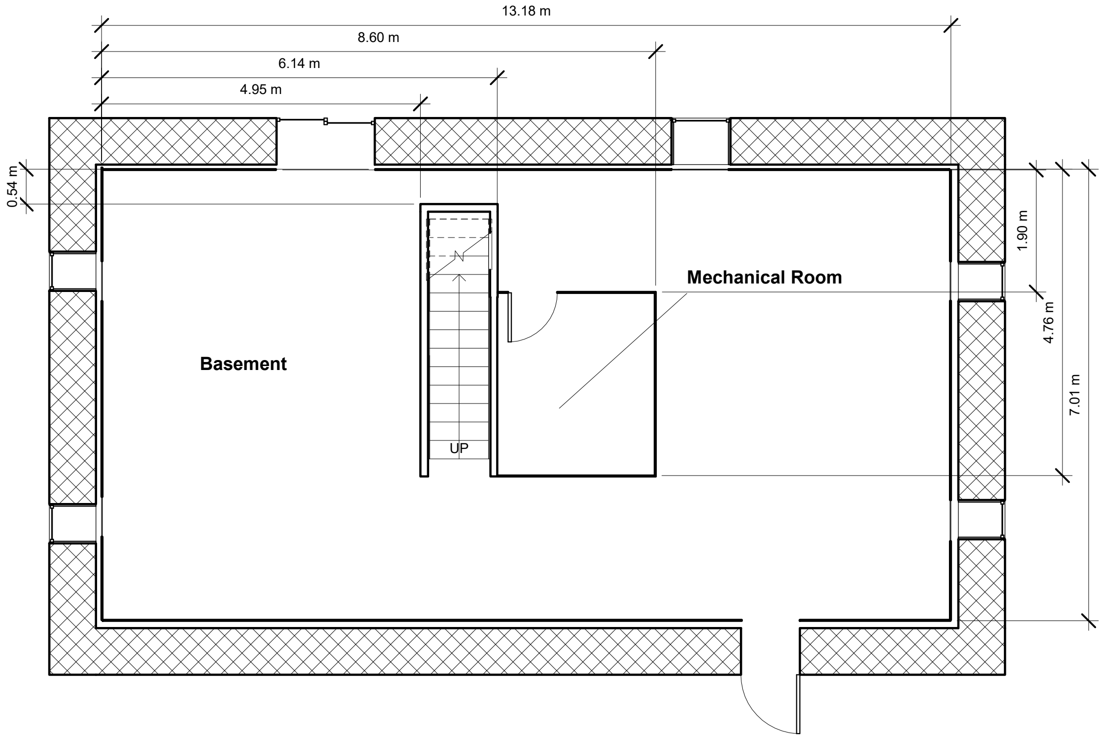

Figure B.1: Basement Plan

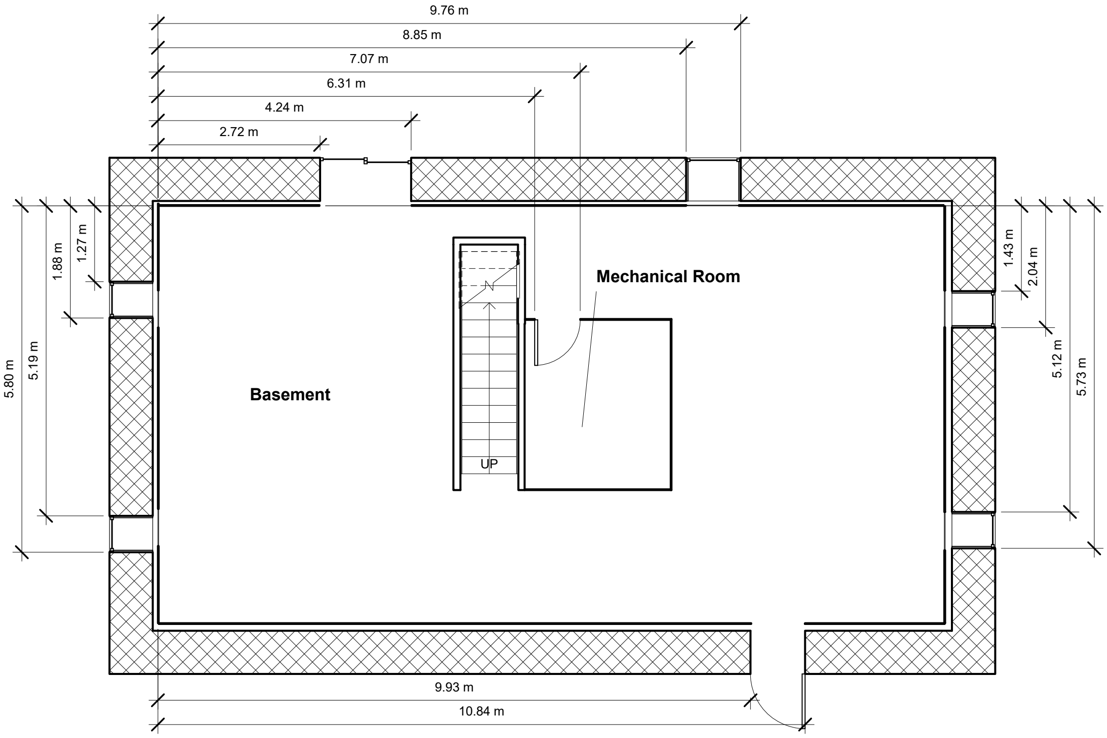

Figure B.2: Basement Vents Detailed

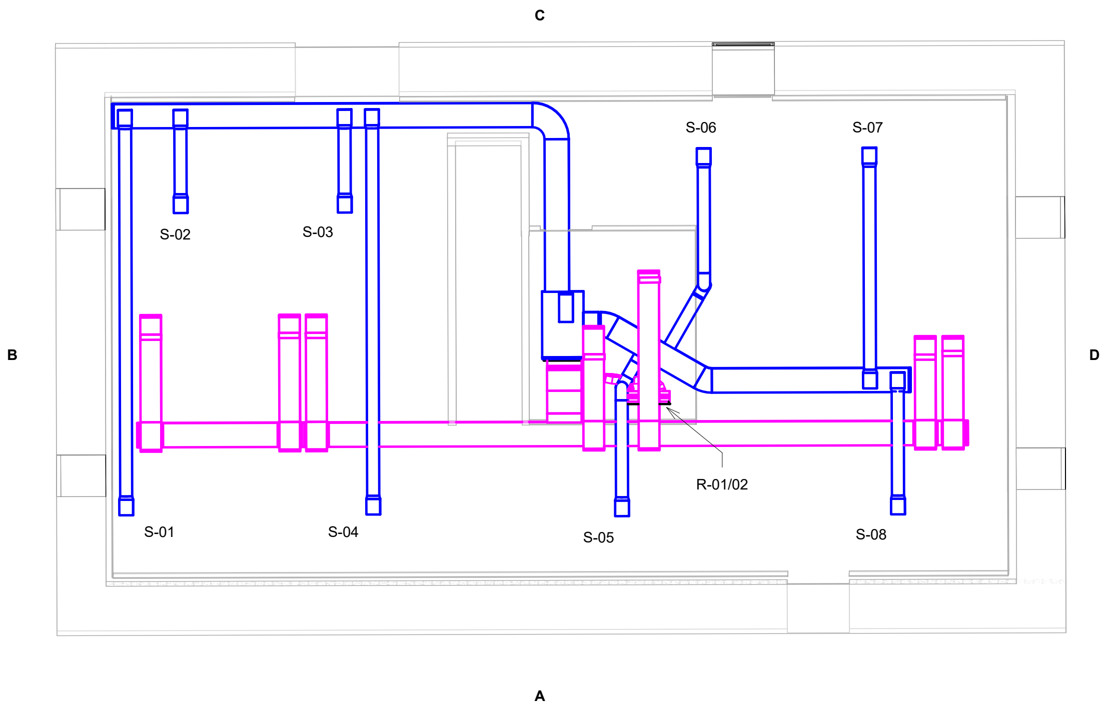

Figure B.3: Basement HVAC Plan

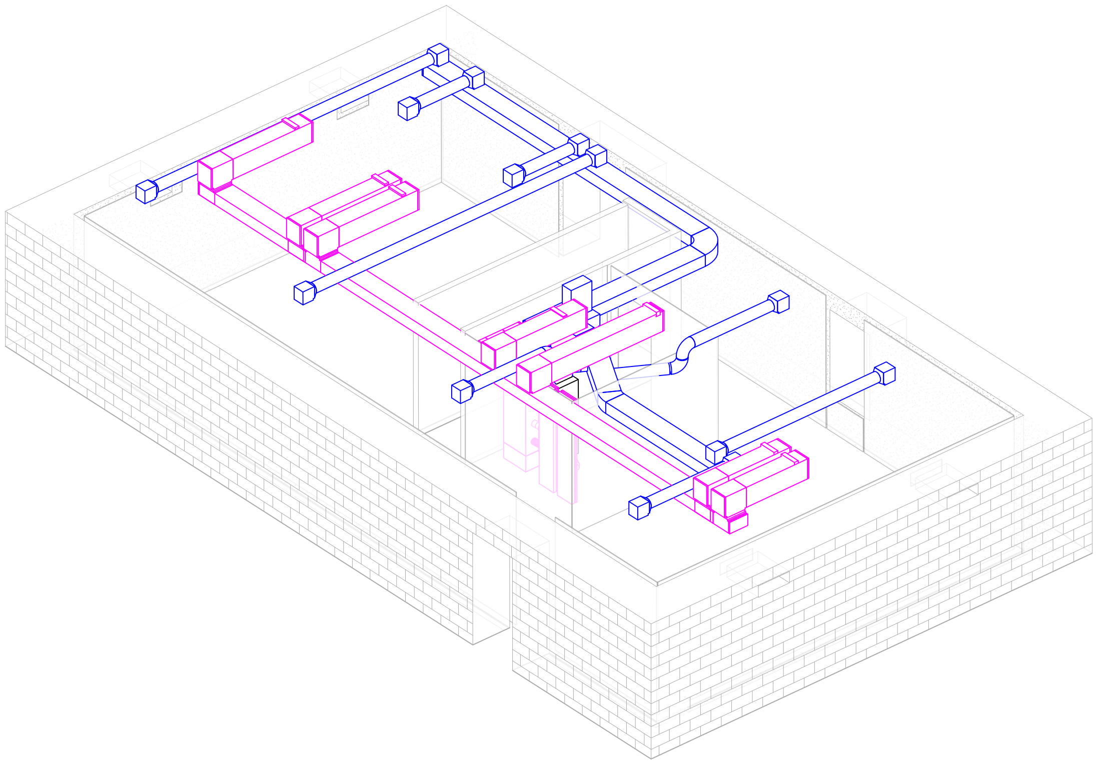

Figure B.4: Basement HVAC Isometric

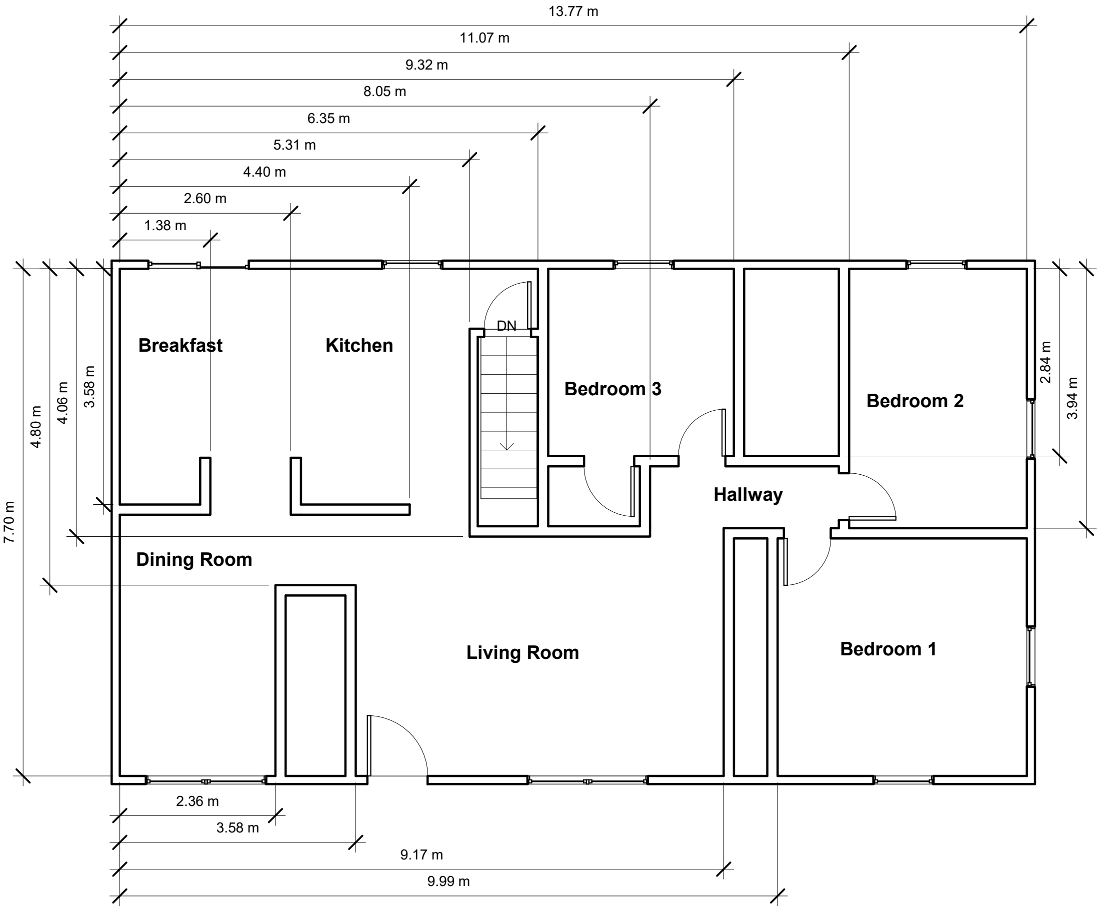

Figure B.5: First Floor Plan

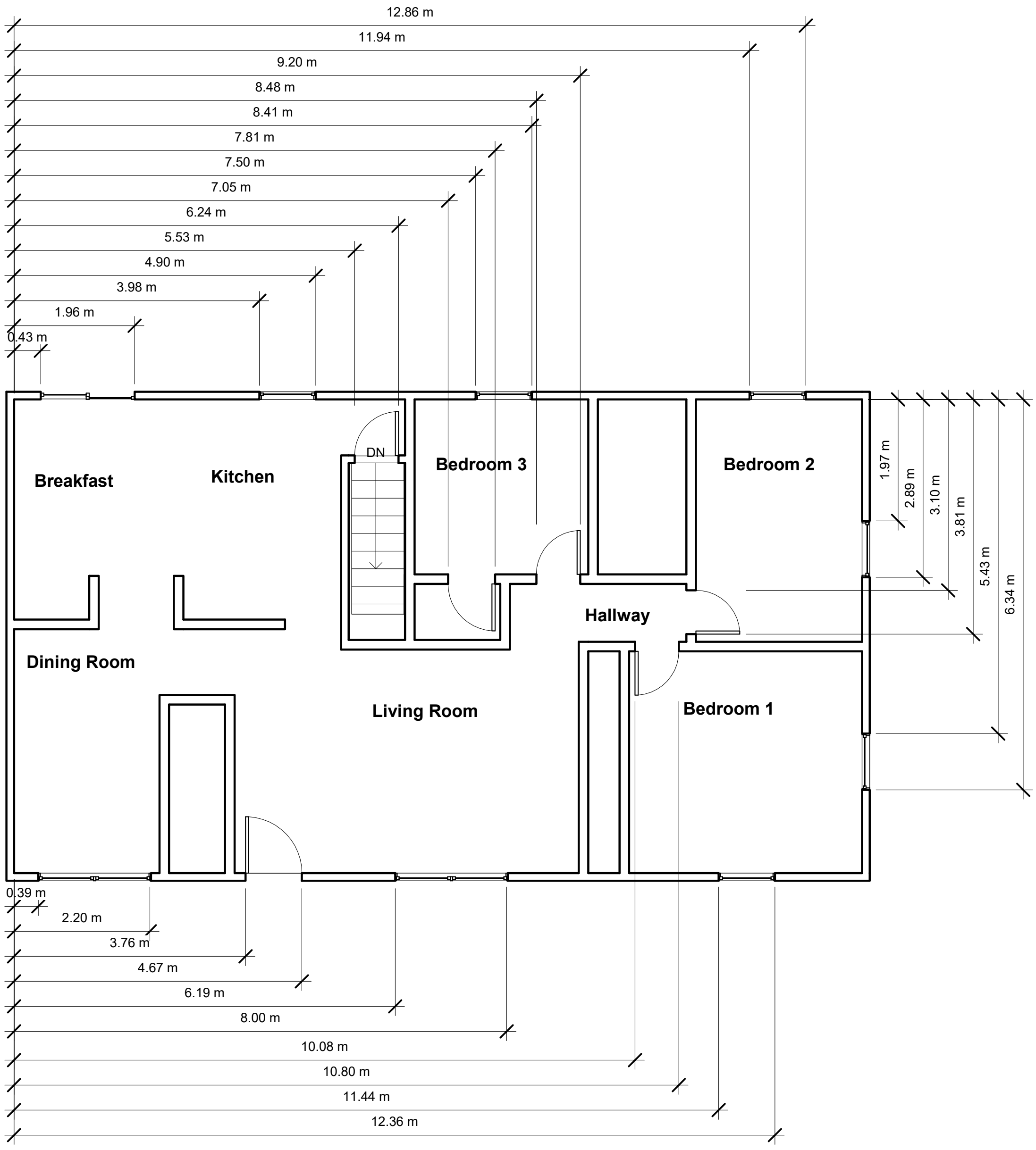

Figure B.6: First Floor Vents

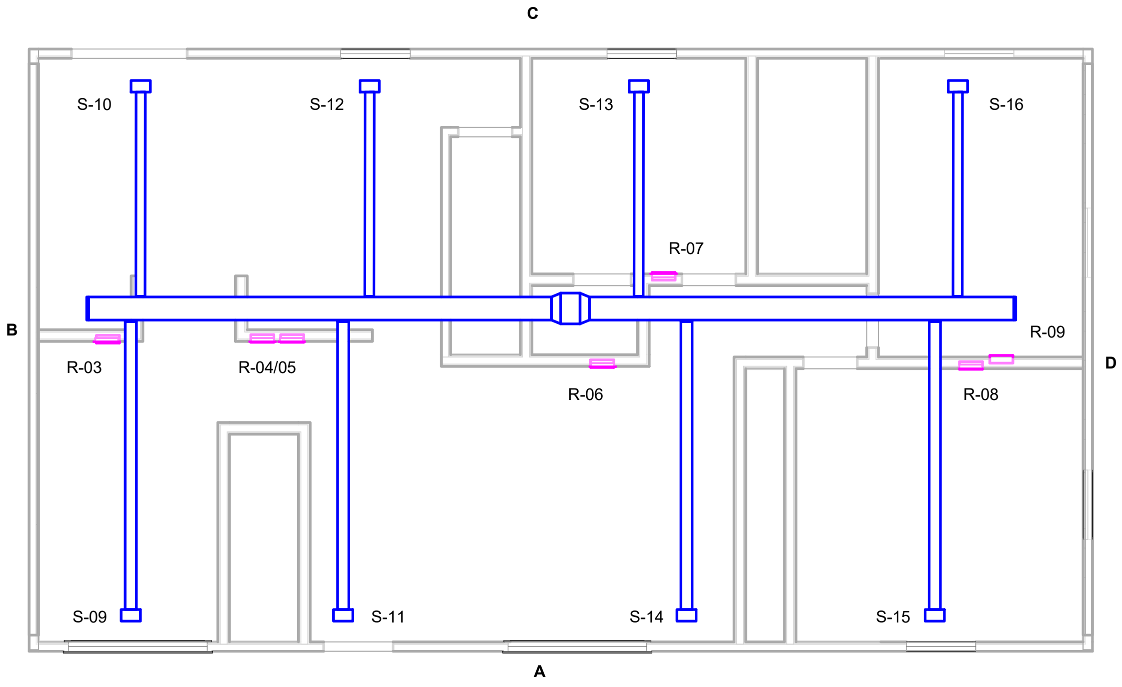

Figure B.7: First Floor HVAC Plan

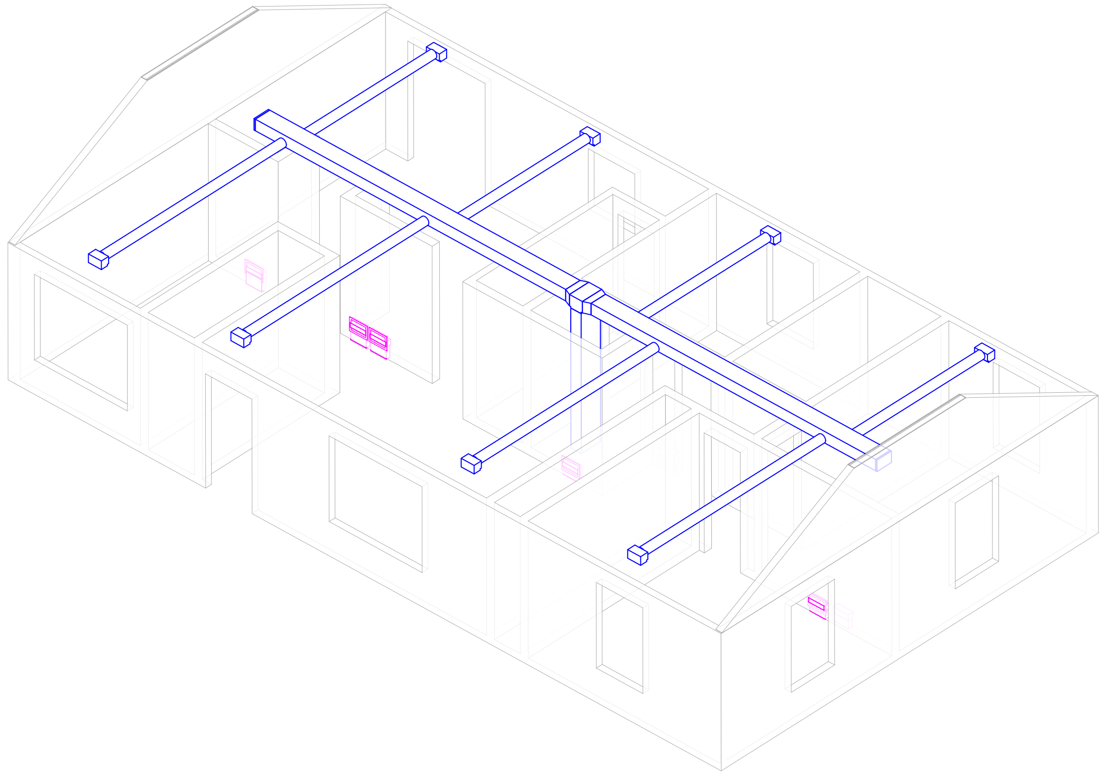

Figure B.8: First Floor HVAC Isometric

Table B.1 provides a list of all the thermalcouple (TC) trees with their channel name and location. A TC tree with a top height of 2.72 meters is comprised of nine thermalcouples at heights of 0.30 m (xTC1), 0.61 m, 0.91 m, 1.22 m, 1.52 m, 1.83 m, 2.13 m, 2.44 m and 2.72 m (xTC9). A TC tree with a top height of 2.41 meters is comprised of eight thermalcouples at heights of 0.30 m (xTC1), 0.61 m, 0.91 m, 1.22 m, 1.52 m, 1.83 m, 2.13 m and 2.41 m (xTC8). 11TC, located in the attic, consisted of eight probes, one at the top of the closet in Bedroom 3 (11TC1), one nested within the attic insulation, 0.3 m above the insulation, 0.61 m, 0.91 m, 1.22 m, 1.52 m, and 1.83 m (11TC8). Table B.2 provides the channel and location for all the single thermocouple measurements.

Table B.1: TC Tree List

Channel	Type	Top Height	Location
1TC	Temperature	2.72 m Above Floor	Basement A/B Corner
2TC	Temperature	2.72 m Above Floor	Basement B/C Corner
3TC	Temperature	2.72 m Above Floor	Basement C/D Corner
4TC	Temperature	2.72 m Above Floor	Basement A/D Corner
5TC	Temperature	2.41 m Above Floor	Living Room
6TC	Temperature	2.41 m Above Floor	Dining Room
7TC	Temperature	2.41 m Above Floor	Kitchen
8TC	Temperature	2.41 m Above Floor	Bedroom 3
9TC	Temperature	2.41 m Above Floor	Bedroom 2
10TC	Temperature	2.41 m Above Floor	Bedroom 1
11TC	Temperature	1.83 m Above Floor	Attic
12TC	Temperature	2.72 m Above Floor	Mechanical Room

Table B.2: Single Thermalcouple List

Channel	Type	Location
TCS-1	Temperature	Living Room Double Return
TCS-2	Temperature	Front Door Supply
TCS-3	Temperature	Dining Room Supply
TCS-4	Temperature	Dining Room Return
TCS-5	Temperature	Kitchen Window Supply
TCS-6	Temperature	Kitchen Slider Supply
TCS-7	Temperature	Bedroom 1 Supply
TCS-8	Temperature	Bedroom 1 Return
TCS-9	Temperature	Bedroom 2 Supply
TCS-10	Temperature	Bedroom 2 Return
TCS-11	Temperature	Bedroom 3 Supply
TCS-12	Temperature	Bedroom 3 Return
TCS-13	Temperature	Living Room Delta Supply
TCS-14	Temperature	Living Room Delta Return
TCS-15	Temperature	Basement B Side A/B Corner Supply
TCS-16	Temperature	Basement B Side B/C Corner Supply
TCS-17	Temperature	Basement B Side C/D Corner Supply
TCS-18	Temperature	Basement B Side A/D Corner Supply
TCS-19	Temperature	Basement D Side A/B Corner Supply
TCS-20	Temperature	Basement D Side B/C Corner Supply
TCS-21	Temperature	Basement D Side C/D Corner Supply
TCS-22	Temperature	Basement D Side A/D Corner Supply
TCS-23	Temperature	Basement Double Return
TC-Therm	Temperature	First Floor Thermostat

Table B.3 provides a list of all the gas measurements collected. The channel, gas measurement and location are provided. Each gas measurement was collected at a height of approximately 1.22 meters above the floor.

Table B.3: Gas Concentration List

Channel	Type	Location
1GASO2	Oxygen	Basement
1GASCO	Carbon Monoxide	Basement
1GASCO2	Carbon Dioxide	Basement
2GASO2	Oxygen	Living Room
2GASCO	Carbon Monoxide	Living Room
2GASCO2	Carbon Dioxide	Living Room
3GASO2	Oxygen	Kitchen
3GASCO	Carbon Monoxide	Kitchen
3GASCO2	Carbon Dioxide	Kitchen
4GASO2	Oxygen	Bedroom 3
4GASCO	Carbon Monoxide	Bedroom 3
4GASCO2	Carbon Dioxide	Bedroom 3
5GASO2	Oxygen	Bedroom 2
5GASCO	Carbon Monoxide	Bedroom 2
5GASCO2	Carbon Dioxide	Bedroom 2
6GASO2	Oxygen	Bedroom 1
6GASCO	Carbon Monoxide	Bedroom 1
6GASCO2	Carbon Dioxide	Bedroom 1

Table B.4 provides a list of all the bi-directional probe channels, measure-

ment type and location. 1BDP consisted of six probes located at heights of 0.99 m (1BDPT1/1BDPV1), 1.27 m, 1.55 m, 1.88 m, 2.18 m and 2.49 m (1BDPT6/1BDPV6). 2BDP and 3BDP were comprised of five probes located at heights of 0.10 m (xB-DPT1/xBDPV1), 0.56 m, 1.02 m, 1.47 m and 1.93 m (xBDPT5/xBDPV5).

Table B.4: Bi-Directional Probe List

Channel	Type	Location
1BDPT	Temperature	Basement Stairwell
1BDPV	Velocity	Basement Stairwell
2BDPT	Temperature	Top Stairwell
2BDPV	Velocity	Top Stairwell
3BDPT	Temperature	Front Door
3BDPV	Velocity	Front Door
4BDPT	Temperature	In Duct
4BDPV	Velocity	In Duct

Table B.5 provides a list of all the pressure tap channels and locations. Each pressure tap location contained three evaluation heights: 0.3 m (xPT1), 1.2 m and 2.13 m (xPT3).

Table B.5: Pressure Tap List

Channel	Type	Location
1PT	Pressure	Basement B Side
2PT	Pressure	Basement D Side
3PT	Pressure	Bedroom 3
4PT	Pressure	Bedroom 2
5PT	Pressure	Bedroom 1
6PT	Pressure	Living Room
7PT	Pressure	Dining Room
8PT	Pressure	Kitchen

Chapter C: FDS Experiment 1 Script

```
&Head CHID='Test_1_7_1', TITLE='Test 1 7_1 Fine Full'  /

&MISC HVAC_MASS_TRANSPORT=.TRUE. /

&PRES SOLVER='UGLMAT' /

&DUMP VELOCITY_ERROR_FILE=.TRUE. /

&DUMP DT_DEVC=1. /
&DUMP DT_BNDF=5. /

MESH FIELD SPECIFICATION

MULTI MESH 10cm x 10cm element size
&MESH IJK=54,50,40, XB=-0.5,4.9,-0.4,4.6,-0.2,3.8 /
&MESH IJK=54,50,40, XB=4.9,10.3,-0.4,4.6,-0.2,3.8 /
```

```
&MESH IJK=54,50,40, XB=10.3,15.7,-0.4,4.6,-0.2,3.8 /

&MESH IJK=54,50,40, XB=-0.5,4.9,4.6,9.6,-0.2,3.8 /

&MESH IJK=54,50,40, XB=4.9,10.3,4.6,9.6,-0.2,3.8 /

&MESH IJK=54,50,40, XB=10.3,15.7,4.6,9.6,-0.2,3.8 /

&MESH IJK=54,50,40, XB=-0.5,4.9,-0.4,4.6,3.8,7.8 /

&MESH IJK=54,50,40, XB=4.9,10.3,-0.4,4.6,3.8,7.8 /

&MESH IJK=54,50,40, XB=10.3,15.7,-0.4,4.6,3.8,7.8 /

&MESH IJK=54,50,40, XB=-0.5,4.9,4.6,9.6,3.8,7.8 /

&MESH IJK=54,50,40, XB=4.9,10.3,4.6,9.6,3.8,7.8 /

&MESH IJK=54,50,40, XB=10.3,15.7,4.6,9.6,3.8,7.8 /


%%%%%%%%%%%%%%%%%%%%%%%%%%%%%%%%%%%%%%%%


TIME SPECIFICATION


&TIME T_END=1007. /


FIRE SPECIFICATION


&SURF ID             = 'BURNER'

    THICKNESS        = 0.01

    HRRPUA           = 287

    RAMP_Q           = 'FIRE_RAMPING' / 237=100 kW, 287=121 kW,
```

592=250 kW, 715=302 kW, 930=393 kW, 947=400 kW FOR 0.65 X 0.65 BURNER

&RAMP ID= 'FIRE_RAMPING', T = 0.0, F = 0.0 / Pilot Ignition

&RAMP ID= 'FIRE_RAMPING', T = 136.0 F = 0.0 / 136

&RAMP ID= 'FIRE_RAMPING', T = 137.0, F = 1.0 / Ignition

&RAMP ID= 'FIRE_RAMPING', T = 976.0, F = 1.0 / 976

&RAMP ID= 'FIRE_RAMPING', T = 977.0, F = 0.0 / Burner Out

&HVAC TYPE_ID='FAN', ID='FAN', VOLUME_FLOW=0., LOSS=2. /RAMP_ID='FAN CURVE', 0.472 m^3/sec=1000CFM, 0.708=1500CFM, 0.944=2000CFM

BURNER LOCATION

BASEMENT BURNER

AIT INIT XB= 11.8, 12.85, 4.15, 5.2, 0.5, 2.4 /

OBST XB= 12.0, 12.65, 4.35, 5.0, 0.0, 0.5, SURF_ID6='CONCRETE FLOOR', 'CONCRETE FLOOR', 'CONCRETE FLOOR', 'CONCRETE FLOOR', 'CONCRETE FLOOR', 'BURNER' /

BEDROOM 1 BURNER

AIT INIT XB= 11.8, 12.85, 1.8, 2.85, 3.2, 5.2 /

```
&OBST XB= 12.0, 12.65, 2.0, 2.65, 3.0, 3.5, SURF_ID6='CONCRETE
FLOOR', 'CONCRETE FLOOR', 'CONCRETE FLOOR', 'CONCRETE FLOOR',
'CONCRETE FLOOR', 'BURNER' /

LIVING ROOM BURNER
AIT INIT XB= 7.8, 8.85, 1.8, 2.85, 3.2, 5.2 /
OBST XB= 8.0, 8.65, 2.0, 2.65, 3.0, 3.5, SURF_ID6='CONCRETE FLOOR',
'CONCRETE FLOOR', 'CONCRETE FLOOR', 'CONCRETE FLOOR', 'CONCRETE
FLOOR', 'BURNER' /

FUEL SPECIFICATION

&REAC FUEL          = 'PROPANE'
    SOOT_YIELD      = 0.024
    CO_YIELD        = 0.02
    AUTO_IGNITION_TEMPERATURE = 450.
    AIT_EXCLUSION_ZONE=11.8, 12.85, 1.8, 2.85, 3.2, 5.2 / BR1 BURNER
    EXCLUSION ZONE

%%%%%%%%%%%%%%%%%%%%%%%%%%%%%%%%%%%%%%%%%%%%%%%%%%

MATERIAL AND SURFACE SPECIFICATIONS
```

```
&MATL ID            = 'GYPSUM BOARD'
    CONDUCTIVITY    = 0.16
    SPECIFIC_HEAT   = 1.0
    DENSITY         = 480.0 /

&MATL ID            = 'ZEKE BLOCK'
    FYI            = 'ZEKE BLOCK'
    DENSITY         = 2400.
    SPECIFIC_HEAT   = 0.96
    CONDUCTIVITY    = 0.01 /

&MATL ID            = 'CONCRETE'
    FYI             = 'BASEMENT FLOOR'
    DENSITY         = 2400.
    SPECIFIC_HEAT   = 0.96
    CONDUCTIVITY    = 0.01 /

&SURF ID            = 'DRYWALL'
    COLOR           = 'SLATE GRAY'
    MATL_ID         = 'GYPSUM BOARD'
    THICKNESS       = 0.012 /

&SURF ID            = 'BASEMENT WALL'
```

```
    COLOR           = 'GRAY'
    MATL_ID         = 'ZEKE BLOCK'
    THICKNESS       = 0.7239 /

&SURF ID            = 'INTERIOR WALL'
    COLOR           = 'DIM GRAY'
    MATL_ID         = 'GYPSUM BOARD'
    THICKNESS       = 0.1556 /

&SURF ID            = 'CONCRETE FLOOR'
    COLOR           = 'TAN'
    MATL_ID         = 'CONCRETE'
    THICKNESS       = 0.15 /

&SURF ID            = 'LEVEL ONE FLOOR'
    COLOR           = 'GRAY'
    MATL_ID         = 'GYPSUM BOARD'
    THICKNESS       = 0.0127 /

ESTABLISHES OPEN FIELD

&VENT MB='XMIN', SURF_ID='OPEN' /
&VENT MB='XMAX', SURF_ID='OPEN' /
```

&VENT MB='YMIN', SURF_ID='OPEN' /

&VENT MB='YMAX', SURF_ID='OPEN' /

&VENT MB='ZMAX', SURF_ID='OPEN' /

&VENT MB='ZMIN', SURF_ID='OPEN' /

BASEMENT LAYOUT

BASEMENT LAYOUT

&OBST XB= 0, 14.8, 0, 0.8, 0, 2.8, SURF_ID='BASEMENT WALL' / EXTERIOR ALPHA WALL

&OBST XB= 0, 0.8, 0, 8.8, 0, 2.8, SURF_ID='BASEMENT WALL' / EXTERIOR BRAVO WALL

&OBST XB= 0, 14.8, 8.0, 8.8, 0, 2.8, SURF_ID='BASEMENT WALL' / EXTERIOR CHARLIE WALL

&OBST XB= 14.0, 14.8, 0, 8.8, 0, 2.8, SURF_ID='BASEMENT WALL' / EXTERIOR DELTA WALL

&OBST XB= 0.8, 14.0, .8, 8.0, 2.8, 3.0, SURF_ID='DRYWALL' / BASEMENT CEILING

&OBST XB= -1, 16, -1, 10, -0.2, 0, SURF_ID='CONCRETE FLOOR' / CONCRETE PAD

&OBST XB= 5.8, 6.0, 3.0, 7.4, 0, 2.8, SURF_ID='INTERIOR WALL' / STAIR WALL BRAVO SIDE

```
&OBST XB= 6.8, 7.0, 3.0, 7.4, 0, 2.8, SURF_ID='INTERIOR WALL' /
STAIR WALL DELTA SIDE
&OBST XB= 5.8, 7.0, 7.4, 7.6, 0, 2.8, SURF_ID='INTERIOR WALL' /
STAIR WALL CHARLIE SIDE
&OBST XB= 7.0, 10.2, 3.0, 3.2, 0, 2.8, SURF_ID='INTERIOR WALL' /
MECH ROOM ALPHA SIDE
&OBST XB= 10.0, 10.2, 3.0, 6.2, 0, 2.8, SURF_ID='INTERIOR WALL' /
MECH ROOM DELTA SIDE
&OBST XB= 7.0, 10.2, 6.0, 6.2, 0, 2.8, SURF_ID='INTERIOR WALL' /
MECH ROOM CHARLIE SIDE


&MULT ID='STAIRS', DXB=0.0, 0.0, 0.17854, 0.17854, 0.163367,
0.163367, N_LOWER=0, N_UPPER=14 / STAIR STEP
MULT ID='STAIRS_CEILING', DXB=0.0, 0.0, 0.026781, 0.026781,
0.028385, 0.028385, N_LOWER=0, N_UPPER=99 /
&MULT ID='STAIRS_CEILING', DXB=0.0, 0.0, 0.2, 0.2, 0.2, 0.2,
N_LOWER=0, N_UPPER=14 / STAIR STEP
OBST XB= 6.0008, 6.7278, 4.6101, 4.78864, 0.0, 0.163367,
MULT_ID='STAIRS', SURF_ID='INTERIOR WALL' / STAIRS
&OBST XB= 6.0, 6.8, 4.6, 4.8, 0.0, 0.163367, MULT_ID='STAIRS',
SURF_ID='INTERIOR WALL' / STAIRS
OBST XB= 6.0008, 6.7278, 4.6101, 4.636881, 2.4384, 2.466785,
MULT_ID='STAIRS_CEILING', SURF_ID='DRYWALL' / STAIRS CEILING
```

&OBST XB= 6.0, 6.8, 4.0, 4.4, 2.8, 3.0, MULT_ID='STAIRS_CEILING',

SURF_ID='DRYWALL' / STAIRS CEILING

HOLE XB= 9.8, 10.8, 0, 0.8, 0, 2.0 / ALPHA WALL DOOR

HOLE XB= 0, 2.0, 1.6, 2.2, 2.0, 2.2 / BRAVO WALL WINDOW ALPHA SIDE

HOLE XB= 0, 2.0, 5.2, 5.8, 2.0, 2.2 / BRAVO WALL WINDOW CHARLIE SIDE

HOLE XB= 1.6, 3.4, 8.0, 8.8, 0, 2.0 / CHARLIE WALL SLIDER DOOR

HOLE XB= 9.4, 10.4, 8.0, 8.8, 0.6, 2.2 / CHARLIE WALL WINDOW

HOLE XB= 14.0, 14.8, 1.6, 2.2, 2.0, 2.2 / DELTA WALL WINDOW ALPHA

SIDE

HOLE XB= 14.0, 14.8, 5.2, 5.8, 2.0, 2.2 / DELTA WALL WINDOW CHARLIE

SIDE

&HOLE XB= 6.0, 6.8, 4.6, 7.2, 2.6, 3.2 / CEILING HOLE STAIRS

&HOLE XB= 7.2, 8.0, 6.0, 6.2, 0.0, 0.2 / MECH ROOM DOOR UNDERCUT 0.1

&HOLE XB= 0.0, 1.0, 1.0, 1.2, 0.0, 0.2 / PRESSURE EQUALIZATION HOLE

FOR BASEMENT

&HOLE XB= 6.8, 7.0, 5.6, 5.8, 0.0, 0.4 / BENEATH STAIR PRESSURE

RELIEF

LEVEL ONE LAYOUT

&OBST XB= 0.4, 14.4, 0.4, 8.4, 2.8, 3.0, SURF_ID='INTERIOR WALL' /

VOID AREA WITHIN LEVELS

&OBST XB= 0.4, 14.4, 0.4, 0.6, 2.8, 5.4, SURF_ID='INTERIOR WALL' /

EXTERIOR ALPHA WALL

&OBST XB= 0.4, 0.6, 0.4, 8.4, 2.8, 5.4, SURF_ID='INTERIOR WALL' /

EXTERIOR BRAVO WALL

&OBST XB= 0.4, 14.4, 8.2, 8.4, 2.8, 5.4, SURF_ID='INTERIOR WALL' /

EXTERIOR CHARLIE WALL

&OBST XB= 14.2, 14.4, 0.4, 8.4, 2.8, 5.4, SURF_ID='INTERIOR WALL' /

EXTERIOR DELTA WALL

&OBST XB= 0.6, 14.2, 0.4, 8.2, 2.8, 3.0, SURF_ID='LEVEL ONE FLOOR' /

LEVEL ONE FLOOR

&OBST XB= 0.6, 14.2, 0.4, 8.2, 5.4, 5.6, SURF_ID='DRYWALL' / LEVEL

ONE CEILING

&OBST XB= 0.4, 1.8, 4.4, 4.8, 3.0, 5.4, SURF_ID='INTERIOR WALL' /

DR-BREAKFAST-HORIZ WALL

&OBST XB= 1.6, 1.8, 4.4, 5.4, 3.0, 5.4, SURF_ID='INTERIOR WALL' /

DR-BREAKFAST-VERT WALL

&OBST XB= 3.0, 3.2, 4.4, 5.4, 3.0, 5.4, SURF_ID='INTERIOR WALL' /

DR-KIT-VERT WALL

&OBST XB= 3.0, 4.6, 4.4, 4.8, 3.0, 5.4, SURF_ID='INTERIOR WALL' /

DR-KIT-HORIZ WALL

&OBST XB= 5.8, 6.0, 4.0, 7.4, 3.0, 5.4, SURF_ID='INTERIOR WALL' /

KITCHEN DELTA WALL

&OBST XB= 5.8, 8.4, 4.0, 4.2, 3.0, 5.4, SURF_ID='INTERIOR WALL' / LR

CHARLIE WALL

&OBST XB= 5.8, 6.8, 7.2, 7.4, 3.0, 5.4, SURF_ID='INTERIOR WALL' /

STAIR DOOR WALL

&OBST XB= 6.8, 7.0, 4.0, 8.4, 3.0, 5.4, SURF_ID='INTERIOR WALL' /

BR3 BRAVO WALL

&OBST XB= 8.2, 8.4, 4.0, 5.4, 3.0, 5.4, SURF_ID='INTERIOR WALL' /

HALL BRAVO WALL

&OBST XB= 6.8, 11.6, 5.2, 5.4, 3.0, 5.4, SURF_ID='INTERIOR WALL' /

HALL CHARLIE WALL

&OBST XB= 9.8, 10.0, 5.2, 8.4, 3.0, 5.4, SURF_ID='INTERIOR WALL' /

BR3 DELTA WALL

&OBST XB= 11.4, 11.6, 4.2, 8.4, 3.0, 5.4, SURF_ID='INTERIOR WALL' /

BR2 BRAVO WALL

&OBST XB= 9.6, 9.8, 0.4, 4.2, 3.0, 5.4, SURF_ID='INTERIOR WALL' / LR

DELTA WALL

&OBST XB= 10.4, 10.6, 0.4, 4.2, 3.0, 5.4, SURF_ID='INTERIOR WALL' /

BR1 BRAVO WALL

&OBST XB= 9.6, 14.4, 4.0, 4.2, 3.0, 5.4, SURF_ID='INTERIOR WALL' /

BR1 CHARLIE WALL

&OBST XB= 2.8, 3.0, 0.4, 3.4, 3.0, 5.4, SURF_ID='INTERIOR WALL' / DR

DELTA WALL

&OBST XB= 2.8, 4.2, 3.2, 3.4, 3.0, 5.4, SURF_ID='INTERIOR WALL' /

```
DR-LR HALL ALPHA WALL
&OBST XB= 4.0, 4.2, 0.4, 3.4, 3.0, 5.4, SURF_ID='INTERIOR WALL' / LR
BRAVO WALL

HOLE XB= 1.0, 2.8, 0.4, 0.6, 3.4, 5.0 /  ALPHA BRAVO WINDOW
HOLE XB= 4.2, 5.2, 0.4, 0.6, 3.0, 5.0 /  ALPHA SIDE DOOR
HOLE XB= 6.2, 8.8, 0.4, 0.6, 3.4, 5.0 / ALPHA WINDOW
HOLE XB= 12.0, 12.8, 0.4, 0.6, 3.4, 5.0 / ALPHA DELTA WINDOW
HOLE XB= 14.2, 14.4, 1.8, 2.8, 3.4, 5.0 / DELTA ALPHA WINDOW
HOLE XB= 14.2, 14.4, 5.2, 6.2, 3.4, 5.0 / DELTA CHARLIE WINDOW
HOLE XB= 12.4, 13.4, 8.2, 8.4, 3.4, 5.0 / CHARLIE DELTA WINDOW
HOLE XB= 8.0, 8.8, 8.2, 8.4, 3.4, 5.0 / CHARLIE WINDOW
HOLE XB= 4.2, 5.0, 8.2, 8.4, 3.4, 4.8 / CHARLIE BRAVO WINDOW
HOLE XB= 1.0, 2.6, 8.2, 8.4, 3.0, 5.0 / CHARLIE SLIDER
HOLE XB= 9.0, 9.6, 5.2, 5.4, 3.1, 5.0 / BR THREE DOOR
&HOLE XB= 9.0, 9.6, 5.2, 5.4, 3.0, 3.2 / BR THREE DOOR UNDERCUT 3.1
HOLE XB= 7.49, 8.12, 5.2, 5.4, 3.10, 5.0 / BR THREE CLOSET DOOR
&HOLE XB= 7.49, 8.12, 5.2, 5.4, 3.0, 3.20 / BR THREE CLOSET DOOR
UNDERCUT 3.1
&HOLE XB= 10.6, 11.2, 4.0, 4.2, 3.0, 3.20 / BR ONE DOOR UNDERCUT 3.1
&HOLE XB= 6.0, 6.74, 7.2, 7.4, 3.0, 3.10 / STAIR DOOR UNDERCUT
&HOLE XB= 11.4, 11.7, 4.4, 5.0, 3.0, 5.0 / BR TWO DOOR
&HOLE XB= 6.0, 6.8, 4.6, 7.2, 2.6, 3.2 / FLOOR HOLE STAIRS
```

```
&HOLE XB= 3.4, 3.8, 0.3, 0.7, 3.1, 4.8 / ALPHA INSTRUMENT ROOM DOOR
OPEN
&HOLE XB= 9.9, 10.3, 0.3, 0.7, 3.1, 3.6 / ALPHA/DELTA INSTRUMENT
ROOM WALL HOLE
&HOLE XB= 10.5, 10.9, 8.1, 8.5, 3.1, 4.8 / CHARLIE INSTRUMENT ROOM
DOOR OPEN

%%%%%%%%%%%%%%%%%%%%%%%%%%%%%
%%%%%%%%%%%%%%%%%%%%%%%%%%%%%

&HOLE XB= 10.6, 11.2, 4.0, 4.2, 3.1, 5.0 / BR ONE DOOR
&HOLE XB= 6.0, 6.74, 7.2, 7.4, 3.10, 5.0 / STAIR DOOR

# &HOLE XB= 14.2, 14.4, 6.2, 6.4, 3.0, 3.2 / FIRST FLOOR PRESSURE
EQUALIZATION
# &HOLE XB= 10.4, 10.6, 0.6, 1.0, 3.0, 3.4 / EXTRA BR1 PRESSURE
RELIEF HOLE
# &HOLE XB= 8.6, 8.8, 8.2, 8.4, 3.2, 3.4 / EXTRA BR3 PRESSURE RELIEF
HOLE

%%%%%%%%%%%%%%%%%%%%%%%%%%%%%
%%%%%%%%%%%%%%%%%%%%%%%%%%%%%
```

ROOF LEVEL

```
&MULT ID='ALPHA_ROOF', DXB=0.0, 0.0, 0.10283, 0.10283, 0.059035,
0.059035, N_LOWER=0, N_UPPER=39 /
&MULT ID='CHARLIE_ROOF', DXB=0.0, 0.0, -0.10283, -0.10283, 0.059035,
0.059035, N_LOWER=0, N_UPPER=39 /
&MULT ID='ROOF_WALL', DXB=0.0, 0.0, 0.10033, -0.10033,
0.057785,0.057785, N_LOWER=0, N_UPPER=39 /
&OBST XB= 0.2620, 14.4828, 0.24930, 0.35213, 5.32690, 5.385935,
MULT_ID='ALPHA_ROOF', COLOR='TAN' /
&OBST XB= 0.2620, 14.4828, 8.37287, 8.47570, 5.32690, 5.385935,
MULT_ID='CHARLIE_ROOF', COLOR='TAN' /
&OBST XB= 0.3620, 0.5176, 0.3493, 8.3757, 5.27690, 5.334685,
MULT_ID='ROOF_WALL', SURF_ID='INTERIOR WALL' / BRAVO ROOF WALL
&OBST XB= 14.2272, 14.3828, 0.3493, 8.3757, 5.27690, 5.334685,
MULT_ID='ROOF_WALL', SURF_ID='INTERIOR WALL' / DELTA ROOF WALL
HOLE XB= 6.8834, 8.3789, 4.6101, 5.1792, 5.1213, 5.2769 / MECHANICAL
SHAFT

&HOLE XB= 6.2, 6.6, 5.4, 6.0, 5.2, 5.8 / PRESSURE RELIEF ABOVE STAIRS
&HOLE XB= 0.2, 0.6, 4.4, 4.8, 6.4, 6.8 / ATTIC PRESSURE RELIEF HOLE
```

ROOF LEVEL

```
&MULT ID='ALPHA_ROOF', DXB=0.0, 0.0, 0.10283, 0.10283, 0.059035,
0.059035, N_LOWER=0, N_UPPER=39 /
&MULT ID='CHARLIE_ROOF', DXB=0.0, 0.0, -0.10283, -0.10283, 0.059035,
0.059035, N_LOWER=0, N_UPPER=39 /
&MULT ID='ROOF_WALL', DXB=0.0, 0.0, 0.10033, -0.10033,
0.057785,0.057785, N_LOWER=0, N_UPPER=39 /
&OBST XB= 0.2620, 14.4828, 0.24930, 0.35213, 5.32690, 5.385935,
MULT_ID='ALPHA_ROOF', COLOR='TAN' /
&OBST XB= 0.2620, 14.4828, 8.37287, 8.47570, 5.32690, 5.385935,
MULT_ID='CHARLIE_ROOF', COLOR='TAN' /
&OBST XB= 0.3620, 0.5176, 0.3493, 8.3757, 5.27690, 5.334685,
MULT_ID='ROOF_WALL', SURF_ID='INTERIOR WALL' / BRAVO ROOF WALL
&OBST XB= 14.2272, 14.3828, 0.3493, 8.3757, 5.27690, 5.334685,
MULT_ID='ROOF_WALL', SURF_ID='INTERIOR WALL' / DELTA ROOF WALL
HOLE XB= 6.8834, 8.3789, 4.6101, 5.1792, 5.1213, 5.2769 / MECHANICAL
SHAFT
```

PRESSURE ZONES

```
&ZONE XYZ= 6.0,2.0,1.0, LEAK_AREA (0) = 0.137 / PRESSURE ZONE
DEFINED BY POINT
```

HVAC

HVAC VENTS; RED IS RETURN(R), BLUE IS SUPPLY(S)

BASEMENT

```
&VENT XB=1.0,1.2,1.8,2.0,2.8,2.8,      SURF_ID='HVAC', ID='S-01',
COLOR='BLUE', IOR=-3 /
&VENT XB=1.8,2.0,6.4,6.6,2.8,2.8,      SURF_ID='HVAC', ID='S-02',
COLOR='BLUE', IOR=-3 /
&VENT XB=4.2,4.4,6.4,6.6,2.8,2.8,      SURF_ID='HVAC', ID='S-03',
COLOR='BLUE', IOR=-3 /
&VENT XB=4.6,4.8,1.8,2.0,2.8,2.8,      SURF_ID='HVAC', ID='S-04',
COLOR='BLUE', IOR=-3 /
&VENT XB=8.2,8.4,1.8,2.0,2.8,2.8,      SURF_ID='HVAC', ID='S-05',
COLOR='BLUE', IOR=-3 /
&VENT XB=9.4,9.6,7.0,7.2,2.8,2.8,      SURF_ID='HVAC', ID='S-06',
COLOR='BLUE', IOR=-3 /
&VENT XB=11.8,12.0,7.0,7.2,2.8,2.8,    SURF_ID='HVAC', ID='S-07',
COLOR='BLUE', IOR=-3 /
&VENT XB=12.2,12.4,1.8,2.0,2.8,2.8,    SURF_ID='HVAC', ID='S-08',
COLOR='BLUE', IOR=-3 /

&VENT XB=8.2,8.6,3.0,3.0,1.6,1.8,      SURF_ID='HVAC', ID='R-01',
```

```
COLOR='RED', IOR=-2 /
&VENT XB=8.6,9.0,3.0,3.0,1.6,1.8,      SURF_ID='HVAC', ID='R-02',
COLOR='RED', IOR=-2 /

&VENT XB=8.6,9.0,3.2,3.2,1.8,2.0,       SURF_ID='HVAC',
ID='R-EXTRA',   COLOR='RED',    IOR=2 /

LEVEL 1
&VENT XB=1.6,2.0,0.8,1.0,5.4,5.4,      SURF_ID='HVAC', ID='S-09',
COLOR='BLUE', IOR=-3 /
&VENT XB=1.6,2.0,7.8,8.0,5.4,5.4,      SURF_ID='HVAC', ID='S-10',
COLOR='BLUE', IOR=-3 /
&VENT XB=4.2,4.6,0.8,1.0,5.4,5.4,      SURF_ID='HVAC', ID='S-11',
COLOR='BLUE', IOR=-3 /
&VENT XB=4.6,5.0,7.8,8.0,5.4,5.4,      SURF_ID='HVAC', ID='S-12',
COLOR='BLUE', IOR=-3 /
&VENT XB=8.2,8.6,7.8,8.0,5.4,5.4,      SURF_ID='HVAC', ID='S-13',
COLOR='BLUE', IOR=-3 /
&VENT XB=8.8,9.2,0.8,1.0,5.4,5.4,      SURF_ID='HVAC', ID='S-14',
COLOR='BLUE', IOR=-3 /
&VENT XB=12.0,12.4,0.8,1.0,5.4,5.4,    SURF_ID='HVAC', ID='S-15',
COLOR='BLUE', IOR=-3 /
&VENT XB=12.4,12.8,7.8,8.0,5.4,5.4,    SURF_ID='HVAC', ID='S-16',
```

```
COLOR='BLUE', IOR=-3 /

&VENT XB=1.2,1.6,4.4,4.4,3.2,3.4,      SURF_ID='HVAC', ID='R-03',
COLOR='RED', IOR=-2 /
&VENT XB=3.2,3.6,4.4,4.4,3.2,3.4,      SURF_ID='HVAC', ID='R-04',
COLOR='RED', IOR=-2 /
&VENT XB=3.6,4.0,4.4,4.4,3.2,3.4,      SURF_ID='HVAC', ID='R-05',
COLOR='RED', IOR=-2 /
&VENT XB=7.6,8.0,4.0,4.0,3.2,3.4,      SURF_ID='HVAC', ID='R-06',
COLOR='RED', IOR=-2 /
&VENT XB=8.4,8.8,5.4,5.4,3.2,3.4,      SURF_ID='HVAC', ID='R-07',
COLOR='RED', IOR=2 /
&VENT XB=12.6,13.0,4.0,4.0,3.2,3.4,    SURF_ID='HVAC', ID='R-08',
COLOR='RED', IOR=-2 /
&VENT XB=13.0,13.4,4.2,4.2,3.2,3.4,    SURF_ID='HVAC', ID='R-09',
COLOR='RED', IOR=2 /

%%%%%%%%%%%%%%%%%%%%%%%%%%%%%
%%%%%%%%%%%%%%%%%%%%%%%%%%%%%

LOUVER VENTS
BR1 LOUVER VENT
VENT XB=10.7, 11.1, 4.0, 4.0, 5.1, 5.3,    SURF_ID='HVAC',
```

```
ID='BR1_LOUVER', COLOR='GREEN', IOR=-2 /
VENT XB=10.7, 11.1, 4.2, 4.2, 5.1, 5.3,    SURF_ID='HVAC',
ID='HALLWAY1',   COLOR='GREEN', IOR=2 /
HVAC TYPE_ID='NODE',   ID='BR1_LOUVER',
DUCT_ID='BR1_LOUVER_HALLWAY1',           VENT_ID='BR1_LOUVER' / DUCT
FROM VENT R-01 TO RETURN TEE 10
HVAC TYPE_ID='NODE',   ID='HALLWAY1',
DUCT_ID='BR1_LOUVER_HALLWAY1',            VENT_ID='HALLWAY1' / DUCT
FROM VENT R-01 TO RETURN TEE 10
HVAC TYPE_ID='DUCT', ID='BR1_LOUVER_HALLWAY1', NODE_ID='BR1_LOUVER',
'HALLWAY1',   AREA=0.0903,      LOSS=1.3,1.3,   LENGTH=0.2,
ROUGHNESS=0.00001,      DUCT_INTERP_TYPE='NODE2',   N_CELLS=2 /

BR3 LOUVER VENT
VENT XB=9.1, 9.5, 5.4, 5.4, 5.1, 5.3,    SURF_ID='HVAC',
ID='BR3_LOUVER', COLOR='GREEN', IOR=2 /
VENT XB=9.1, 9.5, 5.2, 5.2, 5.1, 5.3,    SURF_ID='HVAC',
ID='HALLWAY3',   COLOR='GREEN', IOR=-2 /
HVAC TYPE_ID='NODE',   ID='BR3_LOUVER',
DUCT_ID='BR3_LOUVER_HALLWAY3',           VENT_ID='BR1_LOUVER' / DUCT
FROM VENT R-01 TO RETURN TEE 10
HVAC TYPE_ID='NODE',   ID='HALLWAY3',
DUCT_ID='BR3_LOUVER_HALLWAY3',            VENT_ID='HALLWAY1' / DUCT
```

FROM VENT R-01 TO RETURN TEE 10

HVAC TYPE_ID='DUCT', ID='BR3_LOUVER_HALLWAY3', NODE_ID='BR3_LOUVER',

'HALLWAY3', AREA=0.0903, LOSS=1.3,1.3, LENGTH=0.2,

ROUGHNESS=0.00001, DUCT_INTERP_TYPE='NODE2', N_CELLS=2 /

%%%%%%%%%%%%%%%%%%%%%%%%%%%%%%%

%%%%%%%%%%%%%%%%%%%%%%%%%%%%%%%

HVAC NODES

BASEMENT

&HVAC TYPE_ID='NODE', ID='S-FURNACE',

DUCT_ID='R-FURNACE_S-FURNACE', 'S-FURNACE_ST-01', XYZ=7.4,4.6,1.2

/ S-FURNACE

&HVAC TYPE_ID='NODE', ID='S-01',

DUCT_ID='ST-08_S-01', VENT_ID='S-01' / DUCT FROM SUPPLY

TEE 08 TO VENT S-01

&HVAC TYPE_ID='NODE', ID='S-02',

DUCT_ID='ST-08_S-02', VENT_ID='S-02' / DUCT FROM SUPPLY

TEE 08 TO VENT S-02

&HVAC TYPE_ID='NODE', ID='S-03',

```
DUCT_ID='ST-07_S-03',           VENT_ID='S-03' / DUCT FROM SUPPLY
TEE 07 TO VENT S-03
&HVAC TYPE_ID='NODE',   ID='S-04',
DUCT_ID='ST-06_S-04',           VENT_ID='S-04' / DUCT FROM SUPPLY
TEE 06 TO VENT S-04
&HVAC TYPE_ID='NODE',   ID='S-05',
DUCT_ID='ST-02_S-05',           VENT_ID='S-05' / DUCT FROM SUPPLY
TEE 02 TO VENT S-05
&HVAC TYPE_ID='NODE',   ID='S-06',
DUCT_ID='ST-03_S-06',           VENT_ID='S-06' / DUCT FROM SUPPLY
TEE 03 TO VENT S-06
&HVAC TYPE_ID='NODE',   ID='S-07',
DUCT_ID='ST-04_S-07',           VENT_ID='S-07' / DUCT FROM SUPPLY
TEE 04 TO VENT S-07
&HVAC TYPE_ID='NODE',   ID='S-08',
DUCT_ID='ST-04_S-08',           VENT_ID='S-08' / DUCT FROM SUPPLY
TEE 04 TO VENT S-08

SUPPLY TEES
&HVAC TYPE_ID='NODE',  ID='ST-01',  DUCT_ID='S-FURNACE_ST-01',
'ST-01_ST-02', 'ST-01_ST-06', 'ST-01_ST-10', XYZ=7.4,5.0,2.8 /
DELTA SIDE
&HVAC TYPE_ID='NODE',  ID='ST-02',  DUCT_ID='ST-01_ST-02',
```

```
'ST-02_ST-03', 'ST-02_S-05',                    XYZ=8.6,4.2,2.8 /
&HVAC TYPE_ID='NODE',  ID='ST-03',  DUCT_ID='ST-02_ST-03',
'ST-03_ST-04', 'ST-03_S-06',                    XYZ=8.8,4.0,2.8 /
&HVAC TYPE_ID='NODE',  ID='ST-04',  DUCT_ID='ST-03_ST-04',
'ST-04_S-07', 'ST-04_S-08',                     XYZ=11.0,3.8,2.8 /
BRAVO SIDE
&HVAC TYPE_ID='NODE',  ID='ST-06',  DUCT_ID='ST-01_ST-06',
'ST-06_S-04', 'ST-06_ST-07',                    XYZ=4.6,7.6,2.8 /
&HVAC TYPE_ID='NODE',  ID='ST-07',  DUCT_ID='ST-06_ST-07',
'ST-07_S-03', 'ST-07_ST-08',                    XYZ=4.2,7.6,2.8 /
&HVAC TYPE_ID='NODE',  ID='ST-08',  DUCT_ID='ST-07_ST-08',
'ST-08_S-02', 'ST-08_S-01',                     XYZ=1.8,7.6,2.8 /

&HVAC TYPE_ID='NODE',   ID='R-01', DUCT_ID='R-01_RT-09',
      VENT_ID='R-01' / DUCT FROM VENT R-01 TO RETURN TEE 10
&HVAC TYPE_ID='NODE',   ID='R-02', DUCT_ID='R-02_RT-09',
      VENT_ID='R-02' / DUCT FROM VENT R-02 TO RETURN TEE 09
&HVAC TYPE_ID='NODE',   ID='R-03', DUCT_ID='R-03_RT-02',
      VENT_ID='R-03' / DUCT FROM VENT R-03 TO RETURN TEE 02
&HVAC TYPE_ID='NODE',   ID='R-04', DUCT_ID='R-04_RT-02',
      VENT_ID='R-04' / DUCT FROM VENT R-04 TO RETURN TEE 02
&HVAC TYPE_ID='NODE',   ID='R-05', DUCT_ID='R-05_RT-03',
      VENT_ID='R-05' / DUCT FROM VENT R-05 TO RETURN TEE 03
```

```
&HVAC TYPE_ID='NODE',   ID='R-06', DUCT_ID='R-06_RT-05',
      VENT_ID='R-06' / DUCT FROM VENT R-06 TO RETURN TEE 05
&HVAC TYPE_ID='NODE',   ID='R-07', DUCT_ID='R-07_RT-06',
      VENT_ID='R-07' / DUCT FROM VENT R-07 TO RETURN TEE 06
&HVAC TYPE_ID='NODE',   ID='R-08', DUCT_ID='R-08_RT-07',
      VENT_ID='R-08' / DUCT FROM VENT R-08 TO RETURN TEE 07
&HVAC TYPE_ID='NODE',   ID='R-09', DUCT_ID='R-09_RT-07',
     VENT_ID='R-09' / DUCT FROM VENT R-09 TO RETURN TEE 07
&HVAC TYPE_ID='NODE',   ID='R-EXTRA',   DUCT_ID='R-EXTRA_R-FURNACE'
VENT_ID='R-EXTRA' / EXTRA DUCT TO ACCOUNT FOR RETURN LEAKS
RETURN TEES
&HVAC TYPE_ID='NODE',   ID='RT-02', DUCT_ID='R-03_RT-02',
'R-04_RT-02', 'RT-02_RT-03',       XYZ=3.4,3.0,2.8 /
&HVAC TYPE_ID='NODE',   ID='RT-03', DUCT_ID='RT-02_RT-03',
'R-05_RT-03', 'RT-03_RT-04',      XYZ=3.8,3.0,2.8 /
&HVAC TYPE_ID='NODE',   ID='RT-04', DUCT_ID='RT-03_RT-04',
'RT-05_RT-04', 'RT-04_RT-10',     XYZ=7.4,3.0,2.8 /
&HVAC TYPE_ID='NODE',   ID='RT-05', DUCT_ID='RT-06_RT-05',
'R-06_RT-05', 'RT-05_RT-04',      XYZ=8.0,3.0,2.8 /
&HVAC TYPE_ID='NODE',   ID='RT-06', DUCT_ID='RT-07_RT-06',
'R-07_RT-06', 'RT-06_RT-05',      XYZ=8.8,3.0,2.8 /
&HVAC TYPE_ID='NODE',   ID='RT-07', DUCT_ID='R-09_RT-07',
'R-08_RT-07', 'RT-07_RT-06',       XYZ=12.8,3.0,2.8 /
```

```
&HVAC TYPE_ID='NODE',   ID='RT-09', DUCT_ID='R-02_RT-09',
'R-01_RT-09', 'RT-09_RT-10',       XYZ=7.4,3.8,1.0 /
&HVAC TYPE_ID='NODE',   ID='RT-10', DUCT_ID='RT-09_RT-10',
'RT-04_RT-10', 'RT-10_R-FURNACE', XYZ=7.4,3.8,0.6 /

&HVAC TYPE_ID='NODE',   ID='R-FURNACE', DUCT_ID='RT-10_R-FURNACE',
'R-EXTRA_R-FURNACE', 'R-FURNACE_S-FURNACE',  XYZ=7.4,4.0,0.6 /

LEVEL 1
&HVAC TYPE_ID='NODE',   ID='S-09', DUCT_ID='ST-17_S-09',
VENT_ID='S-09' / DUCT FROM SUPPLY TEE 17 TO VENT S-09
&HVAC TYPE_ID='NODE',   ID='S-10', DUCT_ID='ST-17_S-10',
VENT_ID='S-10' / DUCT FROM SUPPLY TEE 17 TO VENT S-10
&HVAC TYPE_ID='NODE',   ID='S-11', DUCT_ID='ST-16_S-11',
VENT_ID='S-11' / DUCT FROM SUPPLY TEE 16 TO VENT S-11
&HVAC TYPE_ID='NODE',   ID='S-12', DUCT_ID='ST-15_S-12',
VENT_ID='S-12' / DUCT FROM SUPPLY TEE 15 TO VENT S-12
&HVAC TYPE_ID='NODE',   ID='S-13', DUCT_ID='ST-11_S-13',
VENT_ID='S-13' / DUCT FROM SUPPLY TEE 11 TO VENT S-13
&HVAC TYPE_ID='NODE',   ID='S-14', DUCT_ID='ST-12_S-14',
VENT_ID='S-14' / DUCT FROM SUPPLY TEE 12 TO VENT S-14
&HVAC TYPE_ID='NODE',   ID='S-15', DUCT_ID='ST-13_S-15',
VENT_ID='S-15' / DUCT FROM SUPPLY TEE 13 TO VENT S-15
```

```
&HVAC TYPE_ID='NODE',   ID='S-16', DUCT_ID='ST-13_S-16',
VENT_ID='S-16' / DUCT FROM SUPPLY TEE 14 TO VENT S-16


&HVAC TYPE_ID='NODE',   ID='ST-10', DUCT_ID='ST-01_ST-10',
'ST-10_ST-11', 'ST-10_ST-15',     XYZ=7.4,5.0,5.4 /
&HVAC TYPE_ID='NODE',   ID='ST-11', DUCT_ID='ST-10_ST-11',
'ST-11_ST-12', 'ST-11_S-13',      XYZ=8.4,5.0,5.4 /
&HVAC TYPE_ID='NODE',   ID='ST-12', DUCT_ID='ST-11_ST-12',
'ST-12_ST-13', 'ST-12_S-14',      XYZ=9.0,5.0,5.4 /
&HVAC TYPE_ID='NODE',   ID='ST-13', DUCT_ID='ST-12_ST-13',
'ST-13_S-15', 'ST-13_S-16',       XYZ=12.2,5.0,5.4 /
&HVAC TYPE_ID='NODE',   ID='ST-15', DUCT_ID='ST-10_ST-15',
'ST-15_ST-16', 'ST-15_S-12',      XYZ=5.0,5.0,5.4 /
&HVAC TYPE_ID='NODE',   ID='ST-16', DUCT_ID='ST-15_ST-16',
'ST-16_ST-17', 'ST-16_S-11',      XYZ=4.6,5.0,5.4 /
&HVAC TYPE_ID='NODE',   ID='ST-17', DUCT_ID='ST-16_ST-17',
'ST-17_S-09', 'ST-17_S-10',       XYZ=1.8,5.0,5.4 /


HVAC DUCTWORK
 BASEMENT
 &HVAC TYPE_ID='DUCT', ID='R-FURNACE_S-FURNACE',
 NODE_ID='R-FURNACE', 'S-FURNACE', AREA=0.2,      LOSS=1.3,1.3,
 LENGTH=1.0, ROUGHNESS=0.00001,      FAN_ID='FAN',
```

```
DUCT_INTERP_TYPE='NODE2',  N_CELLS=10 /

&HVAC TYPE_ID='DUCT', ID='S-FURNACE_ST-01',    NODE_ID='S-FURNACE',
'ST-01',       AREA=0.2,       LOSS=0.0,0.0,   LENGTH=0.2,
ROUGHNESS=0.00001,      DUCT_INTERP_TYPE='NODE2',   N_CELLS=2 /
&HVAC TYPE_ID='DUCT', ID='ST-01_ST-10',        NODE_ID='ST-01',
'ST-10',           AREA=0.072,     LOSS=2.3,2.3,   LENGTH=3.0,
ROUGHNESS=0.00001,      DUCT_INTERP_TYPE='NODE2',   N_CELLS=30 /
BRAVO SIDE
&HVAC TYPE_ID='DUCT', ID='ST-01_ST-06',        NODE_ID='ST-01',
'ST-06',           AREA=0.072,     LOSS=2.2,2.2,   LENGTH=5.6,
ROUGHNESS=0.00001,      DUCT_INTERP_TYPE='NODE2',   N_CELLS=56 /
DECREASED LOSSES BY 1.0
&HVAC TYPE_ID='DUCT', ID='ST-06_S-04',         NODE_ID='ST-06',
'S-04',            AREA=0.0248,    LOSS=5.1,5.1,  LENGTH=5.6,
ROUGHNESS=0.00001,      DUCT_INTERP_TYPE='NODE2',   N_CELLS=56 /
INCREASED LOSSES BY 1.5
&HVAC TYPE_ID='DUCT', ID='ST-06_ST-07',        NODE_ID='ST-06',
'ST-07',           AREA=0.072,     LOSS=0.5,0.5,   LENGTH=0.4,
ROUGHNESS=0.00001,      DUCT_INTERP_TYPE='NODE2',   N_CELLS=4 /
&HVAC TYPE_ID='DUCT', ID='ST-07_S-03',         NODE_ID='ST-07',
'S-03',            AREA=0.0248,    LOSS=3.6,3.6,  LENGTH=1.2,
ROUGHNESS=0.00001,      DUCT_INTERP_TYPE='NODE2',   N_CELLS=12 /
```

```
&HVAC TYPE_ID='DUCT', ID='ST-07_ST-08',        NODE_ID='ST-07',
'ST-08',           AREA=0.072,     LOSS=0.5,0.5,   LENGTH=2.4,
ROUGHNESS=0.00001,      DUCT_INTERP_TYPE='NODE2',   N_CELLS=24 /
&HVAC TYPE_ID='DUCT', ID='ST-08_S-02',         NODE_ID='ST-08',
'S-02',            AREA=0.0248,    LOSS=3.1,3.1,  LENGTH=1.2,
ROUGHNESS=0.00001,      DUCT_INTERP_TYPE='NODE2',   N_CELLS=12 /
 DECREASED LOSSES BY 0.5
&HVAC TYPE_ID='DUCT', ID='ST-08_S-01',         NODE_ID='ST-08',
'S-01',            AREA=0.0248,    LOSS=3.1,3.1,  LENGTH=6.4,
ROUGHNESS=0.00001,      DUCT_INTERP_TYPE='NODE2',   N_CELLS=64 /
DECREASED LOSSES BY 1.0
DELTA SIDE
&HVAC TYPE_ID='DUCT', ID='ST-01_ST-02',        NODE_ID='ST-01',
'ST-02',           AREA=0.072,     LOSS=3.9,3.9,   LENGTH=1.4,
ROUGHNESS=0.00001,      DUCT_INTERP_TYPE='NODE2',   N_CELLS=14 /
INCREASED LOSSES BY 1.0
&HVAC TYPE_ID='DUCT', ID='ST-02_S-05',         NODE_ID='ST-02',
'S-05',            AREA=0.0248,    LOSS=8.0,8.0,  LENGTH=2.2,
ROUGHNESS=0.00001,      DUCT_INTERP_TYPE='NODE2',   N_CELLS=22 /
INCREASED LOSSES BY 3.5
&HVAC TYPE_ID='DUCT', ID='ST-02_ST-03',        NODE_ID='ST-02',
'ST-03',           AREA=0.072,     LOSS=0.5,0.5,   LENGTH=0.4,
ROUGHNESS=0.00001,      DUCT_INTERP_TYPE='NODE2',   N_CELLS=4 /
```

```
&HVAC TYPE_ID='DUCT', ID='ST-03_S-06',         NODE_ID='ST-03',
'S-06',            AREA=0.0248,    LOSS=8.0,8.0,  LENGTH=3.0,
ROUGHNESS=0.00001,      DUCT_INTERP_TYPE='NODE2',   N_CELLS=30 /
INCREASED LOSSES BY 3.5
&HVAC TYPE_ID='DUCT', ID='ST-03_ST-04',        NODE_ID='ST-03',
'ST-04',           AREA=0.072,     LOSS=1.1,1.1,   LENGTH=0.8,
ROUGHNESS=0.00001,      DUCT_INTERP_TYPE='NODE2',   N_CELLS=8 /
&HVAC TYPE_ID='DUCT', ID='ST-04_S-07',         NODE_ID='ST-04',
'S-07',            AREA=0.0248,    LOSS=4.1,4.1,  LENGTH=3.4,
ROUGHNESS=0.00001,      DUCT_INTERP_TYPE='NODE2',   N_CELLS=38 /
INCREASED LOSSES BY 0.5
&HVAC TYPE_ID='DUCT', ID='ST-04_S-08',         NODE_ID='ST-04',
'S-08',            AREA=0.0248,    LOSS=4.1,4.1,  LENGTH=1.8,
ROUGHNESS=0.00001,      DUCT_INTERP_TYPE='NODE2',   N_CELLS=22 /

RETURN NETWORK
&HVAC TYPE_ID='DUCT', ID='R-03_RT-02',         NODE_ID='R-03',
'RT-02',            AREA=0.062, LOSS=5.4,5.4,      LENGTH=4.6,
ROUGHNESS=0.00001,      DUCT_INTERP_TYPE='NODE2',   N_CELLS=46 /
&HVAC TYPE_ID='DUCT', ID='R-04_RT-02',         NODE_ID='R-04',
'RT-02',            AREA=0.062, LOSS=6.4,6.4,      LENGTH=2.2,
ROUGHNESS=0.00001,      DUCT_INTERP_TYPE='NODE2',   N_CELLS=22 /
INCREASED LOSSES BY 1.5
```

```
&HVAC TYPE_ID='DUCT', ID='RT-02_RT-03',        NODE_ID='RT-02',
'RT-03',          AREA=0.072, LOSS=0.5,0.5,       LENGTH=0.4,
ROUGHNESS=0.00001,      DUCT_INTERP_TYPE='NODE2',   N_CELLS=4 /
&HVAC TYPE_ID='DUCT', ID='R-05_RT-03',         NODE_ID='R-05',
'RT-03',           AREA=0.062, LOSS=6.4,6.4,      LENGTH=2.2,
ROUGHNESS=0.00001,      DUCT_INTERP_TYPE='NODE2',   N_CELLS=22 /
INCREASED LOSSES BY 1.5
&HVAC TYPE_ID='DUCT', ID='RT-03_RT-04',        NODE_ID='RT-03',
'RT-04',          AREA=0.072, LOSS=2.8,2.8,       LENGTH=3.4,
ROUGHNESS=0.00001,      DUCT_INTERP_TYPE='NODE2',   N_CELLS=34 /
INCREASED LOSSES BY 1.0
&HVAC TYPE_ID='DUCT', ID='RT-05_RT-04',        NODE_ID='RT-05',
'RT-04',          AREA=0.072, LOSS=2.8,2.8,       LENGTH=0.4,
ROUGHNESS=0.00001,      DUCT_INTERP_TYPE='NODE2',   N_CELLS=4 /
INCREASED LOSSES BY 1.0
&HVAC TYPE_ID='DUCT', ID='R-06_RT-05',         NODE_ID='R-06',
'RT-05',           AREA=0.062, LOSS=4.9,4.9,      LENGTH=2.4,
ROUGHNESS=0.00001,      DUCT_INTERP_TYPE='NODE2',   N_CELLS=24 /
&HVAC TYPE_ID='DUCT', ID='RT-06_RT-05',        NODE_ID='RT-06',
'RT-05',          AREA=0.072, LOSS=1.0,1.0,       LENGTH=0.8,
ROUGHNESS=0.00001,      DUCT_INTERP_TYPE='NODE2',   N_CELLS=8 /
INCREASED LOSSES BY 0.5
&HVAC TYPE_ID='DUCT', ID='R-07_RT-06',         NODE_ID='R-07',
```

```
'RT-06',            AREA=0.062, LOSS=4.9,4.9,      LENGTH=3.0,
ROUGHNESS=0.00001,      DUCT_INTERP_TYPE='NODE2',   N_CELLS=30 /
&HVAC TYPE_ID='DUCT', ID='RT-07_RT-06',        NODE_ID='RT-07',
'RT-06',           AREA=0.072, LOSS=3.0,3.0,       LENGTH=4.2,
ROUGHNESS=0.00001,      DUCT_INTERP_TYPE='NODE2',   N_CELLS=42 /
INCREASED LOSSES BY 2.5
&HVAC TYPE_ID='DUCT', ID='R-08_RT-07',         NODE_ID='R-08',
'RT-07',            AREA=0.062, LOSS=4.9,4.9,      LENGTH=2.2,
ROUGHNESS=0.00001,      DUCT_INTERP_TYPE='NODE2',   N_CELLS=22 /
&HVAC TYPE_ID='DUCT', ID='R-09_RT-07',         NODE_ID='R-09',
'RT-07',            AREA=0.062, LOSS=5.4,5.4,      LENGTH=2.2,
ROUGHNESS=0.00001,      DUCT_INTERP_TYPE='NODE2',   N_CELLS=22 /
&HVAC TYPE_ID='DUCT', ID='RT-04_RT-10',        NODE_ID='RT-04',
'RT-10',           AREA=0.129, LOSS=1.4,1.4,       LENGTH=1.6,
ROUGHNESS=0.00001,      DUCT_INTERP_TYPE='NODE2',   N_CELLS=16 /
&HVAC TYPE_ID='DUCT', ID='R-02_RT-09',         NODE_ID='R-02',
'RT-09',            AREA=0.062, LOSS=1.4,1.4,      LENGTH=0.2,
ROUGHNESS=0.00001,      DUCT_INTERP_TYPE='NODE2',   N_CELLS=12 /
&HVAC TYPE_ID='DUCT', ID='RT-09_RT-10',        NODE_ID='RT-09',
'RT-10',           AREA=0.1,   LOSS=0.8,0.8,       LENGTH=0.4,
ROUGHNESS=0.00001,      DUCT_INTERP_TYPE='NODE2',   N_CELLS=4 /
INCREASED AREA FROM 0.072 TO 0.1, REDUCED LOSSES BY 1.0
&HVAC TYPE_ID='DUCT', ID='R-01_RT-09',         NODE_ID='R-01',
```

```
'RT-09',            AREA=0.062, LOSS=0.9,0.9,      LENGTH=0.2,
ROUGHNESS=0.00001,      DUCT_INTERP_TYPE='NODE2',   N_CELLS=10 /
&HVAC TYPE_ID='DUCT', ID='RT-10_R-FURNACE',    NODE_ID='RT-10',
'R-FURNACE',       AREA=0.129, LOSS=0.9,0.9,       LENGTH=0.6,
ROUGHNESS=0.00001,      DUCT_INTERP_TYPE='NODE2',   N_CELLS=6 /

&HVAC TYPE_ID='DUCT', ID='R-EXTRA_R-FURNACE',  NODE_ID='R-EXTRA',
'R-FURNACE',     AREA=0.04,   LOSS=2.5,2.5,      LENGTH=0.5,
 ROUGHNESS=0.00001,      DUCT_INTERP_TYPE='NODE2',   N_CELLS=5 /

LEVEL 1
DELTA BRANCH
&HVAC TYPE_ID='DUCT',  ID='ST-10_ST-11',       NODE_ID='ST-10',
'ST-11',        AREA=0.06,  LOSS=1.8,1.8,   LENGTH=0.8,
ROUGHNESS=0.00001,      DUCT_INTERP_TYPE='NODE2',   N_CELLS=8 /
&HVAC TYPE_ID='DUCT',  ID='ST-11_S-13',        NODE_ID='ST-11',
'S-13',         AREA=0.018, LOSS=4.1,4.1,  LENGTH=2.8,
ROUGHNESS=0.00001,      DUCT_INTERP_TYPE='NODE2',   N_CELLS=28 /
INCREASED LOSSES BY 0.5
&HVAC TYPE_ID='DUCT',  ID='ST-11_ST-12',       NODE_ID='ST-11',
'ST-12',        AREA=0.06,  LOSS=0.5,0.5,   LENGTH=0.6,
ROUGHNESS=0.00001,      DUCT_INTERP_TYPE='NODE2',   N_CELLS=6 /
&HVAC TYPE_ID='DUCT',  ID='ST-12_S-14',        NODE_ID='ST-12',
```

```
  'S-14',         AREA=0.018, LOSS=3.6,3.6,  LENGTH=3.8,
  ROUGHNESS=0.00001,      DUCT_INTERP_TYPE='NODE2',   N_CELLS=38 /
&HVAC TYPE_ID='DUCT',  ID='ST-12_ST-13',       NODE_ID='ST-12',
  'ST-13',        AREA=0.06,  LOSS=0.5,0.5,   LENGTH=3.4,
  ROUGHNESS=0.00001,      DUCT_INTERP_TYPE='NODE2',   N_CELLS=34 /
&HVAC TYPE_ID='DUCT',  ID='ST-13_S-15',        NODE_ID='ST-13',
'S-15',         AREA=0.018, LOSS=2.6,2.6,  LENGTH=3.8,
ROUGHNESS=0.00001,      DUCT_INTERP_TYPE='NODE2',   N_CELLS=38 /
DECREASED LOSSES BY 1.0
&HVAC TYPE_ID='DUCT',  ID='ST-13_S-16',        NODE_ID='ST-13',
'S-16',         AREA=0.018, LOSS=4.1,4.1,  LENGTH=3.0,
ROUGHNESS=0.00001,      DUCT_INTERP_TYPE='NODE2',   N_CELLS=30 /
BRAVO BRANCH
&HVAC TYPE_ID='DUCT',  ID='ST-10_ST-15',       NODE_ID='ST-10',
'ST-15',        AREA=0.06,  LOSS=1.8,1.8,   LENGTH=2.6,
ROUGHNESS=0.00001,      DUCT_INTERP_TYPE='NODE2',   N_CELLS=26 /
&HVAC TYPE_ID='DUCT',  ID='ST-15_S-12',        NODE_ID='ST-15',
'S-12',         AREA=0.018, LOSS=3.6,3.6,  LENGTH=2.8,
ROUGHNESS=0.00001,      DUCT_INTERP_TYPE='NODE2',   N_CELLS=28 /
&HVAC TYPE_ID='DUCT',  ID='ST-15_ST-16',       NODE_ID='ST-15',
'ST-16',        AREA=0.06,  LOSS=0.5,0.5,   LENGTH=0.4,
ROUGHNESS=0.00001,      DUCT_INTERP_TYPE='NODE2',   N_CELLS=4 /
&HVAC TYPE_ID='DUCT',  ID='ST-16_S-11',        NODE_ID='ST-16',
```

```
'S-11',         AREA=0.018, LOSS=3.6,3.6,  LENGTH=3.8,
ROUGHNESS=0.00001,      DUCT_INTERP_TYPE='NODE2',   N_CELLS=38 /
&HVAC TYPE_ID='DUCT',  ID='ST-16_ST-17',       NODE_ID='ST-16',
'ST-17',        AREA=0.06,  LOSS=0.5,0.5,   LENGTH=2.8,
ROUGHNESS=0.00001,      DUCT_INTERP_TYPE='NODE2',   N_CELLS=28 /
&HVAC TYPE_ID='DUCT',  ID='ST-17_S-09',        NODE_ID='ST-17',
'S-09',         AREA=0.018, LOSS=4.1,4.1,  LENGTH=3.8,
ROUGHNESS=0.00001,      DUCT_INTERP_TYPE='NODE2',   N_CELLS=38 /
&HVAC TYPE_ID='DUCT',  ID='ST-17_S-10',        NODE_ID='ST-17',
'S-10',         AREA=0.018, LOSS=3.6,3.6,  LENGTH=2.8,
ROUGHNESS=0.00001,      DUCT_INTERP_TYPE='NODE2',   N_CELLS=28 /
```

DEVICE LOCATIONS

```
&DEVC XYZ=3.09,2.55,0.3,        QUANTITY='TEMPERATURE',     ID='1TC1' /
&DEVC XYZ=3.09,2.55,0.61,       QUANTITY='TEMPERATURE',     ID='1TC2' /
&DEVC XYZ=3.09,2.55,0.91,       QUANTITY='TEMPERATURE',     ID='1TC3' /
&DEVC XYZ=3.09,2.55,1.22,       QUANTITY='TEMPERATURE',     ID='1TC4' /
&DEVC XYZ=3.09,2.55,1.52,       QUANTITY='TEMPERATURE',     ID='1TC5' /
&DEVC XYZ=3.09,2.55,1.83,       QUANTITY='TEMPERATURE',     ID='1TC6' /
&DEVC XYZ=3.09,2.55,2.13,       QUANTITY='TEMPERATURE',     ID='1TC7' /
&DEVC XYZ=3.09,2.55,2.44,       QUANTITY='TEMPERATURE',     ID='1TC8' /
&DEVC XYZ=3.09,2.55,2.72,       QUANTITY='TEMPERATURE',     ID='1TC9' /
```

```
&DEVC XYZ=3.11,6.11,0.3,        QUANTITY='TEMPERATURE',     ID='2TC1' /
&DEVC XYZ=3.11,6.11,0.61,       QUANTITY='TEMPERATURE',     ID='2TC2' /
&DEVC XYZ=3.11,6.11,0.91,       QUANTITY='TEMPERATURE',     ID='2TC3' /
&DEVC XYZ=3.11,6.11,1.22,       QUANTITY='TEMPERATURE',     ID='2TC4' /
&DEVC XYZ=3.11,6.11,1.52,       QUANTITY='TEMPERATURE',     ID='2TC5' /
&DEVC XYZ=3.11,6.11,1.83,       QUANTITY='TEMPERATURE',     ID='2TC6' /
&DEVC XYZ=3.11,6.11,2.13,       QUANTITY='TEMPERATURE',     ID='2TC7' /
&DEVC XYZ=3.11,6.11,2.44,       QUANTITY='TEMPERATURE',     ID='2TC8' /
&DEVC XYZ=3.11,6.11,2.72,       QUANTITY='TEMPERATURE',     ID='2TC9' /

&DEVC XYZ=11.66,5.85,0.3,       QUANTITY='TEMPERATURE',     ID='3TC1' /
&DEVC XYZ=11.66,5.85,0.61,      QUANTITY='TEMPERATURE',     ID='3TC2' /
&DEVC XYZ=11.66,5.85,0.91,      QUANTITY='TEMPERATURE',     ID='3TC3' /
&DEVC XYZ=11.66,5.85,1.22,      QUANTITY='TEMPERATURE',     ID='3TC4' /
&DEVC XYZ=11.66,5.85,1.52,      QUANTITY='TEMPERATURE',     ID='3TC5' /
&DEVC XYZ=11.66,5.85,1.83,      QUANTITY='TEMPERATURE',     ID='3TC6' /
&DEVC XYZ=11.66,5.85,2.13,      QUANTITY='TEMPERATURE',     ID='3TC7' /
&DEVC XYZ=11.66,5.85,2.39,      QUANTITY='TEMPERATURE',     ID='3TC8' /
&DEVC XYZ=11.66,5.85,2.72,      QUANTITY='TEMPERATURE',     ID='3TC9' /

&DEVC XYZ=11.66,2.1,0.3,        QUANTITY='TEMPERATURE',     ID='4TC1' /
&DEVC XYZ=11.66,2.1,0.61,       QUANTITY='TEMPERATURE',     ID='4TC2' /
```

```
&DEVC XYZ=11.66,2.1,0.91,       QUANTITY='TEMPERATURE',     ID='4TC3' /
&DEVC XYZ=11.66,2.1,1.22,       QUANTITY='TEMPERATURE',     ID='4TC4' /
&DEVC XYZ=11.66,2.1,1.52,       QUANTITY='TEMPERATURE',     ID='4TC5' /
&DEVC XYZ=11.66,2.1,1.83,       QUANTITY='TEMPERATURE',     ID='4TC6' /
&DEVC XYZ=11.66,2.1,2.13,       QUANTITY='TEMPERATURE',     ID='4TC7' /
&DEVC XYZ=11.66,2.1,2.39,       QUANTITY='TEMPERATURE',     ID='4TC8' /
&DEVC XYZ=11.66,2.1,2.68,       QUANTITY='TEMPERATURE',     ID='4TC9' /

&DEVC XYZ=7.05,3.65,3.3,        QUANTITY='TEMPERATURE',     ID='5TC1' /
&DEVC XYZ=7.05,3.65,3.61,       QUANTITY='TEMPERATURE',     ID='5TC2' /
&DEVC XYZ=7.05,3.65,3.91,       QUANTITY='TEMPERATURE',     ID='5TC3' /
&DEVC XYZ=7.05,3.65,4.22,       QUANTITY='TEMPERATURE',     ID='5TC4' /
&DEVC XYZ=7.05,3.65,4.52,       QUANTITY='TEMPERATURE',     ID='5TC5' /
&DEVC XYZ=7.05,3.65,4.83,       QUANTITY='TEMPERATURE',     ID='5TC6' /
&DEVC XYZ=7.05,3.65,5.13,       QUANTITY='TEMPERATURE',     ID='5TC7' /
&DEVC XYZ=7.05,3.65,5.39,       QUANTITY='TEMPERATURE',     ID='5TC8' /

&DEVC XYZ=1.67,2.63,3.3,        QUANTITY='TEMPERATURE',     ID='6TC1' /
&DEVC XYZ=1.67,2.63,3.61,       QUANTITY='TEMPERATURE',     ID='6TC2' /
&DEVC XYZ=1.67,2.63,3.91,       QUANTITY='TEMPERATURE',     ID='6TC3' /
&DEVC XYZ=1.67,2.63,4.22,       QUANTITY='TEMPERATURE',     ID='6TC4' /
&DEVC XYZ=1.67,2.63,4.52,       QUANTITY='TEMPERATURE',     ID='6TC5' /
&DEVC XYZ=1.67,2.63,4.83,       QUANTITY='TEMPERATURE',     ID='6TC6' /
```

```
&DEVC XYZ=1.67,2.63,5.13,        QUANTITY='TEMPERATURE',     ID='6TC7' /
&DEVC XYZ=1.67,2.63,5.39,        QUANTITY='TEMPERATURE',     ID='6TC8' /

&DEVC XYZ=2.48,6.4,3.3,          QUANTITY='TEMPERATURE',     ID='7TC1' /
&DEVC XYZ=2.48,6.4,3.61,         QUANTITY='TEMPERATURE',     ID='7TC2' /
&DEVC XYZ=2.48,6.4,3.91,         QUANTITY='TEMPERATURE',     ID='7TC3' /
&DEVC XYZ=2.48,6.4,4.22,         QUANTITY='TEMPERATURE',     ID='7TC4' /
&DEVC XYZ=2.48,6.4,4.52,         QUANTITY='TEMPERATURE',     ID='7TC5' /
&DEVC XYZ=2.48,6.4,4.83,         QUANTITY='TEMPERATURE',     ID='7TC6' /
&DEVC XYZ=2.48,6.4,5.13,         QUANTITY='TEMPERATURE',     ID='7TC7' /
&DEVC XYZ=2.48,6.4,5.39,         QUANTITY='TEMPERATURE',     ID='7TC8' /

&DEVC XYZ=8.48,6.73,3.3,         QUANTITY='TEMPERATURE',     ID='8TC1' /
&DEVC XYZ=8.48,6.73,3.61,        QUANTITY='TEMPERATURE',     ID='8TC2' /
&DEVC XYZ=8.48,6.73,3.91,        QUANTITY='TEMPERATURE',     ID='8TC3' /
&DEVC XYZ=8.48,6.73,4.22,        QUANTITY='TEMPERATURE',     ID='8TC4' /
&DEVC XYZ=8.48,6.73,4.52,        QUANTITY='TEMPERATURE',     ID='8TC5' /
&DEVC XYZ=8.48,6.73,4.83,        QUANTITY='TEMPERATURE',     ID='8TC6' /
&DEVC XYZ=8.48,6.73,5.13,        QUANTITY='TEMPERATURE',     ID='8TC7' /
&DEVC XYZ=8.48,6.73,5.39,        QUANTITY='TEMPERATURE',     ID='8TC8' /

&DEVC XYZ=12.83,6.23,3.3,        QUANTITY='TEMPERATURE',     ID='9TC1' /
&DEVC XYZ=12.83,6.23,3.61,       QUANTITY='TEMPERATURE',     ID='9TC2' /
```

```
&DEVC XYZ=12.83,6.23,3.91,      QUANTITY='TEMPERATURE',     ID='9TC3' /
&DEVC XYZ=12.83,6.23,4.22,      QUANTITY='TEMPERATURE',     ID='9TC4' /
&DEVC XYZ=12.83,6.23,4.52,      QUANTITY='TEMPERATURE',     ID='9TC5' /
&DEVC XYZ=12.83,6.23,4.83,      QUANTITY='TEMPERATURE',     ID='9TC6' /
&DEVC XYZ=12.83,6.23,5.13,      QUANTITY='TEMPERATURE',     ID='9TC7' /
&DEVC XYZ=12.83,6.23,5.39,      QUANTITY='TEMPERATURE',     ID='9TC8' /


&DEVC XYZ=12.4,3.55,3.3,        QUANTITY='TEMPERATURE',     ID='10TC1' /
&DEVC XYZ=12.4,3.55,3.61,       QUANTITY='TEMPERATURE',     ID='10TC2' /
&DEVC XYZ=12.4,3.55,3.91,       QUANTITY='TEMPERATURE',     ID='10TC3' /
&DEVC XYZ=12.4,3.55,4.22,       QUANTITY='TEMPERATURE',     ID='10TC4' /
&DEVC XYZ=12.4,3.55,4.52,       QUANTITY='TEMPERATURE',     ID='10TC5' /
&DEVC XYZ=12.4,3.55,4.83,       QUANTITY='TEMPERATURE',     ID='10TC6' /
&DEVC XYZ=12.4,3.55,5.13,       QUANTITY='TEMPERATURE',     ID='10TC7' /
&DEVC XYZ=12.4,3.55,5.39,       QUANTITY='TEMPERATURE',     ID='10TC8' /


&DEVC XYZ=7.72,4.2,5.31,        QUANTITY='TEMPERATURE',     ID='11TC8' /
&DEVC XYZ=7.72,4.2,6.62,        QUANTITY='TEMPERATURE',     ID='11TC7' /
&DEVC XYZ=7.72,4.2,5.92,        QUANTITY='TEMPERATURE',     ID='11TC6' /
&DEVC XYZ=7.72,4.2,6.23,        QUANTITY='TEMPERATURE',     ID='11TC5' /
&DEVC XYZ=7.72,4.2,6.53,        QUANTITY='TEMPERATURE',     ID='11TC4' /
&DEVC XYZ=7.72,4.2,6.84,        QUANTITY='TEMPERATURE',     ID='11TC3' /
&DEVC XYZ=7.72,4.2,7.14,        QUANTITY='TEMPERATURE',     ID='11TC2' /
```

```
&DEVC XYZ=7.72,4.2,7.45,        QUANTITY='TEMPERATURE',     ID='11TC1' /


&DEVC XYZ=8.34,4.04,0.30,       QUANTITY='TEMPERATURE',     ID='12TC1' /

&DEVC XYZ=8.34,4.04,0.61,       QUANTITY='TEMPERATURE',     ID='12TC2' /

&DEVC XYZ=8.34,4.04,0.91,       QUANTITY='TEMPERATURE',     ID='12TC3' /

&DEVC XYZ=8.34,4.04,1.22,       QUANTITY='TEMPERATURE',     ID='12TC4' /

&DEVC XYZ=8.34,4.04,1.52,       QUANTITY='TEMPERATURE',     ID='12TC5' /

&DEVC XYZ=8.34,4.04,1.83,       QUANTITY='TEMPERATURE',     ID='12TC6' /

&DEVC XYZ=8.34,4.04,2.13,       QUANTITY='TEMPERATURE',     ID='12TC7' /

&DEVC XYZ=8.34,4.04,2.44,       QUANTITY='TEMPERATURE',     ID='12TC8' /

&DEVC XYZ=8.34,4.04,2.74,       QUANTITY='TEMPERATURE',     ID='12TC9' /


&DEVC XYZ=3.4,4.39,3.30,        QUANTITY='TEMPERATURE',     ID='TCS_1' /

&DEVC XYZ=4.40,0.90,5.39,       QUANTITY='TEMPERATURE',     ID='TCS_2' /

&DEVC XYZ=1.80,0.90,5.39,       QUANTITY='TEMPERATURE',     ID='TCS_3' /

&DEVC XYZ=1.40,4.39,3.30,       QUANTITY='TEMPERATURE',     ID='TCS_4' /

&DEVC XYZ=4.80,7.9,5.39,        QUANTITY='TEMPERATURE',     ID='TCS_5' /

&DEVC XYZ=1.80,7.9,5.39,        QUANTITY='TEMPERATURE',     ID='TCS_6' /

&DEVC XYZ=12.2,0.90,5.39,       QUANTITY='TEMPERATURE',     ID='TCS_7' /

&DEVC XYZ=12.8,3.99,3.30,       QUANTITY='TEMPERATURE',     ID='TCS_8' /

&DEVC XYZ=12.6,7.90,5.39,       QUANTITY='TEMPERATURE',     ID='TCS_9' /

&DEVC XYZ=13.2,4.21,3.30,       QUANTITY='TEMPERATURE',     ID='TCS_10' /

&DEVC XYZ=8.40,7.9,5.39,        QUANTITY='TEMPERATURE',     ID='TCS_11' /
```

```
&DEVC XYZ=8.6,5.41,3.30,        QUANTITY='TEMPERATURE',     ID='TCS_12' /

&DEVC XYZ=9.0,0.90,5.39,        QUANTITY='TEMPERATURE',     ID='TCS_13' /

&DEVC XYZ=7.8,3.99,3.30,        QUANTITY='TEMPERATURE',     ID='TCS_14' /

&DEVC XYZ=1.10,1.90,2.79,       QUANTITY='TEMPERATURE',     ID='TCS_15' /

&DEVC XYZ=1.90,6.50,2.79,       QUANTITY='TEMPERATURE',     ID='TCS_16' /

&DEVC XYZ=4.30,6.50,2.79,       QUANTITY='TEMPERATURE',     ID='TCS_17' /

&DEVC XYZ=4.70,1.90,2.79,       QUANTITY='TEMPERATURE',     ID='TCS_18' /

&DEVC XYZ=8.30,1.90,2.79,       QUANTITY='TEMPERATURE',     ID='TCS_19' /

&DEVC XYZ=9.5,7.10,2.79,        QUANTITY='TEMPERATURE',     ID='TCS_20' /

&DEVC XYZ=11.9,7.10,2.79,       QUANTITY='TEMPERATURE',     ID='TCS_21' /

&DEVC XYZ=12.3,1.90,2.79,       QUANTITY='TEMPERATURE',     ID='TCS_22' /

&DEVC XYZ=8.40,2.99,1.70,       QUANTITY='TEMPERATURE',     ID='TCS_23' /

&DEVC XYZ=8.41,4.19,4.30,       QUANTITY='TEMPERATURE',     ID='TC_Therm' /


&DEVC XYZ=5.65,6.67,0.25,       QUANTITY='PRESSURE', ID='1PT1' /

&DEVC XYZ=5.65,6.67,1.19,       QUANTITY='PRESSURE', ID='1PT4' /

&DEVC XYZ=5.65,6.67,2.16,       QUANTITY='PRESSURE', ID='1PT7' /

&DEVC XYZ=10.35,5.68,0.28,      QUANTITY='PRESSURE', ID='2PT1' /

&DEVC XYZ=10.35,5.68,1.19,      QUANTITY='PRESSURE', ID='2PT4' /

&DEVC XYZ=10.35,5.68,2.16,      QUANTITY='PRESSURE', ID='2PT7' /

&DEVC XYZ=4.35,2.58,3.33,       QUANTITY='PRESSURE', ID='6PT1' /

&DEVC XYZ=4.35,2.58,4.22,       QUANTITY='PRESSURE', ID='6PT4' /

&DEVC XYZ=4.35,2.58,5.11,       QUANTITY='PRESSURE', ID='6PT7' /
```

```
&DEVC XYZ=2.65,2.56,3.30,       QUANTITY='PRESSURE', ID='7PT1' /

&DEVC XYZ=2.65,2.56,4.19,       QUANTITY='PRESSURE', ID='7PT4' /

&DEVC XYZ=2.65,2.56,5.11,       QUANTITY='PRESSURE', ID='7PT7' /

&DEVC XYZ=0.75,7.92,3.30,       QUANTITY='PRESSURE', ID='8PT1' /

&DEVC XYZ=0.75,7.92,4.19,       QUANTITY='PRESSURE', ID='8PT4' /

&DEVC XYZ=0.75,7.92,5.11,       QUANTITY='PRESSURE', ID='8PT7' /

&DEVC XYZ=9.65,7.31,3.33,       QUANTITY='PRESSURE', ID='3PT1' /

&DEVC XYZ=9.65,7.31,4.22,       QUANTITY='PRESSURE', ID='3PT4' /

&DEVC XYZ=9.65,7.31,5.11,       QUANTITY='PRESSURE', ID='3PT7' /

&DEVC XYZ=11.75,7.31,3.33,      QUANTITY='PRESSURE', ID='4PT1' /

&DEVC XYZ=11.75,7.31,4.24,      QUANTITY='PRESSURE', ID='4PT4' /

&DEVC XYZ=11.75,7.31,5.11,      QUANTITY='PRESSURE', ID='4PT7' /

&DEVC XYZ=10.75,1.82,3.30,      QUANTITY='PRESSURE', ID='5PT1' /

&DEVC XYZ=10.75,1.82,4.22,      QUANTITY='PRESSURE', ID='5PT4' /

&DEVC XYZ=10.75,1.82,5.13,      QUANTITY='PRESSURE', ID='5PT7' /


&DEVC XYZ=6.4,3.40,0.99,        QUANTITY='VELOCITY', ID='1BDPV1' /

&DEVC XYZ=6.4,3.40,1.27,        QUANTITY='VELOCITY', ID='1BDPV2' /

&DEVC XYZ=6.4,3.40,1.55,        QUANTITY='VELOCITY', ID='1BDPV3' /

&DEVC XYZ=6.4,3.40,1.88,        QUANTITY='VELOCITY', ID='1BDPV4' /

&DEVC XYZ=6.4,3.40,2.18,        QUANTITY='VELOCITY', ID='1BDPV5' /

&DEVC XYZ=6.4,3.40,2.49,        QUANTITY='VELOCITY', ID='1BDPV6' /

&DEVC XYZ=6.4,7.0,3.10,         QUANTITY='VELOCITY', ID='2BDPV1' /
```

```
&DEVC XYZ=6.4,7.0,3.56,        QUANTITY='VELOCITY', ID='2BDPV2' /
&DEVC XYZ=6.4,7.0,4.02,        QUANTITY='VELOCITY', ID='2BDPV3' /
&DEVC XYZ=6.4,7.0,4.47,        QUANTITY='VELOCITY', ID='2BDPV4' /
&DEVC XYZ=6.4,7.0,4.93,        QUANTITY='VELOCITY', ID='2BDPV5' /
&DEVC XYZ=4.70,0.39,3.36,      QUANTITY='VELOCITY', ID='3BDPV1' /
&DEVC XYZ=4.70,0.39,3.71,      QUANTITY='VELOCITY', ID='3BDPV2' /
&DEVC XYZ=4.70,0.39,4.09,      QUANTITY='VELOCITY', ID='3BDPV3' /
&DEVC XYZ=4.70,0.39,4.35,      QUANTITY='VELOCITY', ID='3BDPV4' /
&DEVC XYZ=4.70,0.39,4.68,      QUANTITY='VELOCITY', ID='3BDPV5' /

&DEVC XYZ=6.4,3.40,0.99,       QUANTITY='TEMPERATURE', ID='1BDPT1' /
&DEVC XYZ=6.4,3.40,1.27,       QUANTITY='TEMPERATURE', ID='1BDPT2' /
&DEVC XYZ=6.4,3.40,1.55,       QUANTITY='TEMPERATURE', ID='1BDPT3' /
&DEVC XYZ=6.4,3.40,1.88,       QUANTITY='TEMPERATURE', ID='1BDPT4' /
&DEVC XYZ=6.4,3.40,2.18,       QUANTITY='TEMPERATURE', ID='1BDPT5' /
&DEVC XYZ=6.4,3.40,2.49,       QUANTITY='TEMPERATURE', ID='1BDPT6' /
&DEVC XYZ=6.4,7.0,3.10,        QUANTITY='TEMPERATURE', ID='2BDPT1' /
&DEVC XYZ=6.4,7.0,3.56,        QUANTITY='TEMPERATURE', ID='2BDPT2' /
&DEVC XYZ=6.4,7.0,4.02,        QUANTITY='TEMPERATURE', ID='2BDPT3' /
&DEVC XYZ=6.4,7.0,4.47,        QUANTITY='TEMPERATURE', ID='2BDPT4' /
&DEVC XYZ=6.4,7.0,4.93,        QUANTITY='TEMPERATURE', ID='2BDPT5' /
&DEVC XYZ=4.70,0.39,3.36,      QUANTITY='TEMPERATURE', ID='3BDPT1' /
&DEVC XYZ=4.70,0.39,3.71,      QUANTITY='TEMPERATURE', ID='3BDPT2' /
```

```
&DEVC XYZ=4.70,0.39,4.09,       QUANTITY='TEMPERATURE', ID='3BDPT3' /

&DEVC XYZ=4.70,0.39,4.35,       QUANTITY='TEMPERATURE', ID='3BDPT4' /

&DEVC XYZ=4.70,0.39,4.68,       QUANTITY='TEMPERATURE', ID='3BDPT5' /


&DEVC XYZ=3.04,6.32,1.22,       QUANTITY='VOLUME FRACTION',

SPEC_ID='OXYGEN',           ID='1GASO2' /

&DEVC XYZ=3.04,6.32,1.22,       QUANTITY='VOLUME FRACTION', SPEC_ID='CARBON

MONOXIDE',  ID='1GASCO' /

&DEVC XYZ=3.04,6.32,1.22,       QUANTITY='VOLUME FRACTION', SPEC_ID='CARBON

DIOXIDE',   ID='1GASCO2' /

&DEVC XYZ=3.04,6.32,1.22,       QUANTITY='VOLUME FRACTION', SPEC_ID='WATER

VAPOR',   ID='B_W4' /


%%%%%%%%%%%%%%%%%%%%%%%%%%%%%%%%%%%%%%%%%%%%%%%%%%%

%%%%%%%%%%%%%%%%%%%%%%%%%%%%%%%%%%%%%%%%%%%%%%%%%%%


DEVC XYZ=3.04,6.32,1.22,        QUANTITY='VOLUME FRACTION',

SPEC_ID='OXYGEN',           ID='1GASO2' / BASEMENT GAS TRAP DURING BASEMENT

BURNER EXPERIMENTS

DEVC XYZ=3.04,6.32,1.22,        QUANTITY='VOLUME FRACTION', SPEC_ID='CARBON

MONOXIDE',  ID='1GASCO' / BASEMENT GAS TRAP DURING BASEMENT BURNER

EXPERIMENTS

DEVC XYZ=3.04,6.32,1.22,        QUANTITY='VOLUME FRACTION', SPEC_ID='CARBON
```

```
DIOXIDE',   ID='1GASCO2' / BASEMENT GAS TRAP DURING BASEMENT BURNER
EXPERIMENTS
DEVC XYZ=3.04,6.32,1.22,        QUANTITY='VOLUME FRACTION', SPEC_ID='WATER
VAPOR',   ID='B_W4' / BASEMENT GAS TRAP DURING BASEMENT BURNER EXPERIMENTS

%%%%%%%%%%%%%%%%%%%%%%%%%%%%%%%%%%%%%%%%%%%%%%%%%%%%%%%%%%%%
%%%%%%%%%%%%%%%%%%%%%%%%%%%%%%%%%%%%%%%%%%%%%%%%%%%%%%%%%%%%

&DEVC XYZ=7.0,3.65,4.24,        QUANTITY='VOLUME FRACTION',
SPEC_ID='OXYGEN',           ID='2GASO2' /
&DEVC XYZ=7.0,3.65,4.24,        QUANTITY='VOLUME FRACTION', SPEC_ID='CARBON
MONOXIDE',  ID='2GASCO' /
&DEVC XYZ=7.0,3.65,4.24,        QUANTITY='VOLUME FRACTION', SPEC_ID='CARBON
DIOXIDE',   ID='2GASCO2' /
&DEVC XYZ=7.0,3.65,4.24,        QUANTITY='VOLUME FRACTION', SPEC_ID='WATER
VAPOR',   ID='LR_W4' /
&DEVC XYZ=8.43,6.80,4.24,       QUANTITY='VOLUME FRACTION',
SPEC_ID='OXYGEN',           ID='4GASO2' /
&DEVC XYZ=8.43,6.80,4.24,       QUANTITY='VOLUME FRACTION', SPEC_ID='CARBON
MONOXIDE',  ID='4GASCO' /
&DEVC XYZ=8.43,6.80,4.24,       QUANTITY='VOLUME FRACTION', SPEC_ID='CARBON
DIOXIDE',   ID='4GASCO2' /
&DEVC XYZ=8.43,6.80,4.24,       QUANTITY='VOLUME FRACTION', SPEC_ID='WATER
```

```
VAPOR',   ID='BR3_W4' /
&DEVC XYZ=12.85,6.22,4.22,      QUANTITY='VOLUME FRACTION',
SPEC_ID='OXYGEN',           ID='5GASO2' /
&DEVC XYZ=12.85,6.22,4.22,      QUANTITY='VOLUME FRACTION', SPEC_ID='CARBON
MONOXIDE',  ID='5GASCO' /
&DEVC XYZ=12.85,6.22,4.22,      QUANTITY='VOLUME FRACTION', SPEC_ID='CARBON
DIOXIDE',   ID='5GASCO2' /
&DEVC XYZ=12.85,6.22,4.22,      QUANTITY='VOLUME FRACTION', SPEC_ID='WATER
VAPOR',   ID='BR2_W4' /
&DEVC XYZ=12.42,3.55,4.19,      QUANTITY='VOLUME FRACTION',
SPEC_ID='OXYGEN',           ID='6GASO2' /
&DEVC XYZ=12.42,3.55,4.19,      QUANTITY='VOLUME FRACTION', SPEC_ID='CARBON
MONOXIDE',  ID='6GASCO' /
&DEVC XYZ=12.42,3.55,4.19,      QUANTITY='VOLUME FRACTION', SPEC_ID='CARBON
DIOXIDE',   ID='6GASCO2' /
&DEVC XYZ=12.42,3.55,4.19,      QUANTITY='VOLUME FRACTION', SPEC_ID='WATER
VAPOR',   ID='BR1_W4' /

IN-DUCT DEVICES

&DEVC QUANTITY='DUCT VELOCITY',     DUCT_ID='ST-01_ST-10',
  ID='4BDPV1'/
&DEVC QUANTITY='NODE TEMPERATURE',     NODE_ID='ST-01',
```

```
ID='4BDPT1'/

&SLCF PBY=1.90,  QUANTITY='VELOCITY',        VECTOR=.TRUE.,
CELL_CENTERED=.TRUE. /
&SLCF PBY=7.10,  QUANTITY='VELOCITY',        VECTOR=.TRUE.,
CELL_CENTERED=.TRUE. /
&SLCF PBZ=2.79,  QUANTITY='VELOCITY',        VECTOR=.TRUE.,
CELL_CENTERED=.TRUE. /
&SLCF PBZ=3.30,  QUANTITY='VELOCITY',        VECTOR=.TRUE.,
CELL_CENTERED=.TRUE. /
&SLCF PBZ=5.39,  QUANTITY='VELOCITY',        VECTOR=.TRUE.,
CELL_CENTERED=.TRUE. /
&SLCF PBY=1.90,  QUANTITY='PRESSURE',        VECTOR=.TRUE.,
CELL_CENTERED=.TRUE. /
&SLCF PBY=7.10,  QUANTITY='PRESSURE',        VECTOR=.TRUE.,
CELL_CENTERED=.TRUE. /
&SLCF PBZ=2.79,  QUANTITY='PRESSURE',        VECTOR=.TRUE.,
CELL_CENTERED=.TRUE. /
&SLCF PBZ=3.30,  QUANTITY='PRESSURE',        VECTOR=.TRUE.,
CELL_CENTERED=.TRUE. /
&SLCF PBZ=5.39,  QUANTITY='PRESSURE',        VECTOR=.TRUE.,
CELL_CENTERED=.TRUE. /
&SLCF PBY=1.90,  QUANTITY='TEMPERATURE',     VECTOR=.TRUE.,
```

```
CELL_CENTERED=.TRUE. /
&SLCF PBY=7.10,  QUANTITY='TEMPERATURE',     VECTOR=.TRUE.,
CELL_CENTERED=.TRUE. /
&SLCF PBZ=2.79,  QUANTITY='TEMPERATURE',     VECTOR=.TRUE.,
CELL_CENTERED=.TRUE. /
&SLCF PBZ=3.30,  QUANTITY='TEMPERATURE',     VECTOR=.TRUE.,
CELL_CENTERED=.TRUE. /
&SLCF PBZ=5.39,  QUANTITY='TEMPERATURE',     VECTOR=.TRUE.,
CELL_CENTERED=.TRUE. /
&SLCF PBY=1.90,  QUANTITY='VOLUME FRACTION', SPEC_ID='OXYGEN',
CELL_CENTERED=.TRUE. /
&SLCF PBY=7.10,  QUANTITY='VOLUME FRACTION', SPEC_ID='OXYGEN',
CELL_CENTERED=.TRUE. /
&SLCF PBZ=2.79,  QUANTITY='VOLUME FRACTION', SPEC_ID='OXYGEN',
CELL_CENTERED=.TRUE. /
&SLCF PBZ=3.30,  QUANTITY='VOLUME FRACTION', SPEC_ID='OXYGEN',
CELL_CENTERED=.TRUE. /
&SLCF PBZ=5.39,  QUANTITY='VOLUME FRACTION', SPEC_ID='OXYGEN',
CELL_CENTERED=.TRUE. /

&BNDF       QUANTITY='VELOCITY ERROR' /
```

```
&TAIL /
```

Bibliography

[1] D. Madrzykowski and C. Weinschenk. Understanding and Fighting Basement Fires. Technical report, UL Firefighter Safety Research Institute, Columbia, Maryland, March 2018.

[2] R. Zevotek and S. Kerber. Study of the Effectiveness of Fire Service Positive Pressure Ventilation During Fire Attack in Single Family Homes Incorporating Modern Construction Practices. Technical report, UL Firefighter Safety Research Institute, Columbia, MD, May 2016.

[3] S. Marsar. SURVIVABILITY PROFILING: ARE THE VICTIMS SAVABLE? *Fire Engineering*, 162(12):69, December 2009.

[4] J. Harwood. HVAC: A Brief History, August 2016.

[5] K. McGrattan, S. Hostikka, R. McDermott, J. Floyd, and M. Vanella. *Fire Dynamics Simulator, Technical Reference Guide, Volume 3: Validation*. National Institute of Standards and Technology, Gaithersburg, Maryland, USA, and VTT Technical Research Centre of Finland, Espoo, Finland, sixth edition, October 2019.

[6] S.C. Sugarman. *HVAC Fundamentals*. Fairmont Press, Inc., Lilburn, GA, 2016.

[7] K. McGrattan, S. Hostikka, R. McDermott, J. Floyd, and M. Vanella. *Fire Dynamics Simulator, Technical Reference Guide*. National Institute of Standards and Technology, Gaithersburg, Maryland, USA, and VTT Technical Research Centre of Finland, Espoo, Finland, sixth edition, October 2019. Vol. 1: Mathematical Model; Vol. 2: Verification Guide; Vol. 3: Validation Guide; Vol. 4: Configuration Management Plan.

[8] K. McGrattan, S. Hostikka, R. McDermott, J. Floyd, and M. Vanella. *Fire Dynamics Simulator, User's Guide*. National Institute of Standards and Technology, Gaithersburg, Maryland, USA, and VTT Technical Research Centre of Finland, Espoo, Finland, sixth edition, October 2019.

[9] K. McGrattan, S. Hostikka, R. McDermott, J. Floyd, and M. Vanella. *Fire Dynamics Simulator, Technical Reference Guide, Volume 2: Verification*. National Institute of Standards and Technology, Gaithersburg, Maryland, USA, and VTT Technical Research Centre of Finland, Espoo, Finland, sixth edition, October 2019.

[10] K. McGrattan, C. Bouldin, and G. Forney. Federal Building and Fire Safety Investigation of the World Trade Center Disaster: Computer Simulation of the Fires in the WTC Towers. NIST NCSTAR 1-5F, National Institute of Standards and Technology, Gaithersburg, Maryland, September 2005.

[11] W.L. Grosshandler, N. Bryner, D. Madrzykowski, and K. Kuntz. Report of the Technical Investigation of The Station Nightclub Fire. NIST NCSTAR 2, National Institute of Standards and Technology, Gaithersburg, MD, June 2005.

[12] N. Bryner, S.P. Fuss, B.W. Klein, and A.D. Putorti. Technical Study of the Sofa Super Store Fire, South Carolina, June 18, 2007. NIST Special Publication 1118, National Institute of Standards and Technology, Gaithersburg, MD, March 2011.

[13] C.G. Weinschenk, K.J. Overholt, and D. Madrzykowski. Simulation of an Attic Fire in a Wood Frame Residential Structure – Chicago, IL. NIST Technical Note 1838, National Institute of Standards and Technology, Gaithersburg, Maryland, 2014.

[14] K.J. Overholt, C.G. Weinschenk, and D. Madrzykowski. Simulation of a Fire in a Hillside Residential Structure – San Francisco, CA. NIST Technical Note 1856, National Institute of Standards and Technology, Gaithersburg, Maryland, 2014.

[15] D. Madrzykowski, G.P. Forney, and W.D. Walton. Simulation of the Dynamics of a Fire in a Two-Story Duplex, Iowa, December 22, 1999. NISTIR 6854, National Institute of Standards and Technology, Gaithersburg, Maryland, 2002.

[16] B. Ralph and R. Carvel. Coupled hybrid modelling in fire safety engineering; a literature review. *Fire Safety Journal*, 100:157–170, 2018.

[17] R.O. Gauntt, RK. Cole, C.M. Erickson, R. Gido, R.D. Gasser, S.B. Rodriguez, and M.F. Young. Melcor computer code manuals: Reference manuals version 1.8.5. 2, 2000.

[18] J. Floyd, S. Hunt, F. Williams, and P. Tatem. Fire and smoke simulator (fssim) version 1: Theory manual. 2004.

[19] ASTM International. *Standard E779-10: Standard Test Method for Determining Air Leakage Rate by Fan Pressurization*, 2018.

[20] A.K. Persilly. Airtightness of Commercial and Institutional Buildings: Blowing Holes in the Myth of Tight Buildings. In *Thermal Envelopes VII Conference*, pages 829–837, Clearwater, FL, 1998.

[21] ThermalZone, Philadelphia, PA. *Air Handlers TZHSLT/TZHDLT-High Efficiency*, 2019.

[22] ThermalZone, Philadelphia, PA. *Heat Pumps TZPLS-14 Series*, 2018.

[23] L.G. Blevins. Behavior of bare and aspirated thermocouples in compartment fires. In *National Heat Transfer Conference, 33rd Proceedings*, pages 15–17, 1999.

[24] W.M. Pitts, E. Braun, R. Peacock, H. Mitler, E. Johnson, P. Reneke, and L.G. Blevins. Temperature uncertainties for bare-bead and aspirated thermocouple measurements in fire environments. *ASTM Special Technical Publication*, 1427:3–15, 2003.

[25] R.A. Bryant. A comparison of gas velocity measurements in a full-scale enclosure fire. *Fire Safety Journal*, 44:793–800, 2009.

[26] M. Bundy, A. Hamins, E.L. Johnsson, S.C. Kim, G.H. Ko, and D.B. Lenhart. Measurements of Heat and Combustion Products in Reduced-Scale Ventilated-Limited Compartment Fires. NIST Technical Note 1483, National Institute of Standards and Technology, Gaithersburg, Maryland, 2007.

[27] A. Lock, M. Bundy, E.L. Johnsson, A. Hamins, G.H. Ko, C. Hwang, P. Fuss, and R. Harris. Experimental study of the effects of fuel type, fuel distribution, and vent size on full-scale underventilated compartment fires in an ISO 9705 room. NIST Technical Note 1603, National Institute of Standards and Technology, Gaithersburg, Maryland, 2008.

[28] S. Kerber and D. Madrzykowski. Evaluating Positive Pressure Ventilation In Large Structures: School Pressure and Fire Experiments. NIST Technical Note 1498, National Institute of Standards and Technology, Gaithersburg, Maryland, 2008.

[29] M.S. Owen. *2017 ASHRAE Handbook: Fundamentals*. American Society of Heating, Refrigerating, and Air-Conditioning Engineers, Inc., Atlanta, GA, 2017.

[30] Testo, West Chester, PA. *Testo 420 Flow Hood: Instruction Manual.*

[31] Testo, West Chester, PA. *Testo 420 Flow Hood: Data Sheet.*
